# The Causal Role of the Gut Microbiota–Plasma Metabolome Axis in Myeloproliferative Neoplasm Pathogenesis: A Mendelian Randomization and Mediation Analysis

**DOI:** 10.3390/metabo15080501

**Published:** 2025-07-28

**Authors:** Hao Kan, Ka Zhang, Aiqin Mao, Li Geng

**Affiliations:** 1 Wuxi School of Medicine, Jiangnan University, Wuxi 214125, China; 2 School of Food Science and Technology, Jiangnan University, Wuxi 214125, China

**Keywords:** myeloproliferative neoplasm, gut microbiome, plasma metabolites, mendelian randomization analysis, mediation analysis

## Abstract

Background: Myeloproliferative neoplasms (MPN), a group of chronic hematologic neoplasms, are driven by inflammatory mechanisms that influence disease initiation and progression. Emerging evidence highlights the gut microbiome and plasma metabolome as pivotal immunomodulators, yet their causal roles in MPN pathogenesis remain uncharacterized. Methods: We conducted a two-sample Mendelian randomization (MR) analysis to systematically evaluate causal relationships between 196 gut microbial taxa, 526 plasma metabolites, and MPN risk. Instrumental variables were derived from genome-wide association studies (GWASs) of microbial/metabolite traits. Validation utilized 16S rRNA sequencing data from NCBI Bioproject PRJNA376506. Mediation and multivariable MR analyses elucidated metabolite-mediated pathways linking microbial taxa to MPN. Results: Our MR analysis revealed that 7 intestinal taxa and 17 plasma metabolites are causally linked to MPN. External validation confirmed the three taxa’s differential abundance in MPN cohorts. Mediation analysis revealed two mediated relationships, of which succinylcarnitine mediated 14.5% of the effect, and lysine 27.9%, linking the *Eubacterium xylanophilum group* to MPN. Multivariate MR analysis showed that both succinylcarnitine (*p* = 0.004) and lysine (*p* = 0.040) had a significant causal effect on MPN. Conclusions: This study identifies novel gut microbiota–metabolite axes driving MPN pathogenesis through immunometabolic mechanisms. The validated biomarkers provide potential therapeutic targets for modulating inflammation in myeloproliferative disorders.

## 1. Introduction

Myeloproliferative neoplasms (MPN) are hematologic neoplasms driven by somatic mutations in hematopoietic stem cells (HSCs), leading to the excessive proliferation of mature myeloid cells. Epidemiologic evidence highlights the strong heritability of MPNs, positioning them among cancers with the highest genetic influence [1,2]. Despite this, only a limited number of genetic risk loci have been identified to date [3,4], underscoring critical gaps in understanding MPN pathogenesis.

The gastrointestinal tract, the body’s largest immune organ, plays a pivotal role in systemic immune regulation [5,6]. Within this system, the gut microbiome (GM) serves as a key immune modulator by maintaining intestinal barrier integrity and producing immunoregulatory metabolites [7,8]. Emerging studies suggest GM dysbiosis may influence MPN progression, with patients exhibiting reduced Phascolarctobacterium abundance and elevated Parabacteroides levels [9,10,11]. Furthermore, MPN-associated perturbations in plasma metabolites, including bile acids and short-chain fatty acids, correlate with systemic inflammation [12,13,14,15], prompting hypotheses of causal GM–metabolite–MPN interactions. Elucidating these relationships could reveal diagnostic biomarkers or therapeutic targets.

Mendelian randomization (MR), a method leveraging genetic variants as instrumental variables, enables robust causal inference by minimizing confounding and reverse causation [16,17]. Recent advances in microbial genomics now permit MR applications to investigate GM–disease relationships [18,19]. Here, we employ MR to systematically evaluate causal links between the gut microbiome, plasma metabolome, and MPN. Our analysis identifies 7 microbial taxa and 17 metabolites as putative causal factors, with evidence of mediation pathways. This study provides the first comprehensive MR assessment of gut microbiome–metabolome–MPN interactions.

## 2. Materials and Methods

### 2.1. Study Design

**Figure 1:**
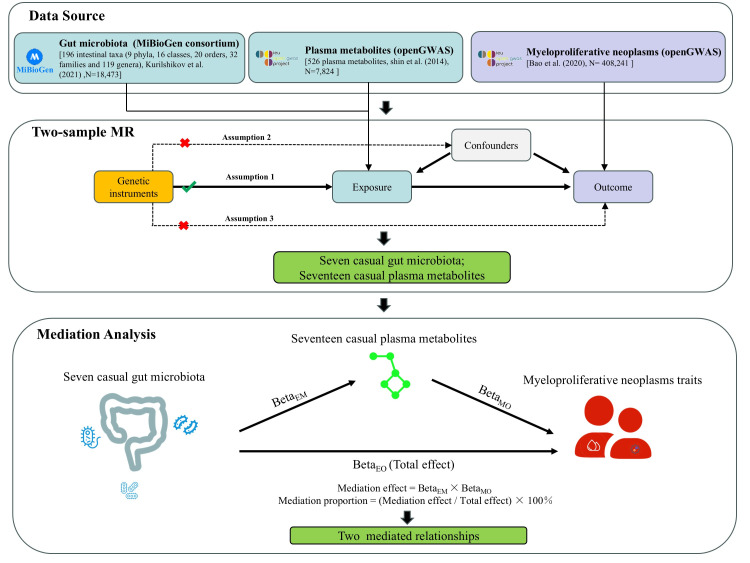
Research flow chart of the study. Mendelian randomization study rationale: assumption 1, genetic instruments are associated with exposure; assumption 2, genetic instruments are not associated with confounders; assumption 3, genetic instruments are not associated with outcome, and genetic instruments act on outcome only through exposure. MR, Mendelian randomization. N, the sample size of each dataset. Note: Gut microbiota data from Kurilshikov et al. (2021) [20]; Plasma metabolite data from Shin et al. (2014) [21]; Myeloproliferative neoplasms data from Bao et al. (2020) [2].

In this research, we initially gathered published GWAS summary data covering traits such as gut microbiota, plasma metabolites, and MPN. Next, two-sample Mendelian randomization (MR) analyses were employed to assess the causal relationships among gut microbiota, plasma metabolites, and MPN. Subsequently, two-step and multivariate MR analyses were conducted to explore the mediating effect of plasma metabolites on the relationship between gut microbiota and MPN. A summary of the study design is presented in Figure 1.

### 2.2. Data Sources

This study examined the abundance of intestinal bacteria and plasma metabolite concentrations as exposure factors, with MPN as the outcome. Summary data on gut microbiota were obtained from the MiBioGen consortium (https://mibiogen.gcc.rug.nl), which includes 18,340 participants from 24 cohorts, with 78% of the dataset comprising European subjects [20]. The MiBioGen consortium provided curated genome-wide genotypes and 16S fecal microbiome data. Only taxa present in over 10% of samples were included, resulting in 196 known taxa: 119 genera, 32 families, 20 orders, 16 classes, and 9 phyla. Plasma metabolomic data for up to 7824 European participants were sourced from the MetabolomIPS database (https://metabolomips.org/gwas/index.php, accessed on 9 July 2024) [21]. The database includes 526 plasma metabolites involved in various metabolic pathways relevant to different diseases. MPN genetic data were retrieved from the UK Biobank via the IEU OpenGWAS database (https://gwas.mrcieu.ac.uk/), serving as an independent validation cohort with 408,241 participants of European ancestry, including 1086 cases and 407,155 controls (Supplementary Table S1).

### 2.3. SNP Selection

SNPs were selected based on three core MR principles: (1) SNPs should not be associated with confounding variables; (2) SNPs must be strongly linked with the exposure or mediator; (3) SNPs should affect the outcome solely through the exposure [22]. For MR, SNPs associated with gut microbiota were chosen at a suggestive *p*-value threshold of less than 1 × 10^−5^, aligning with prior MR studies [18,23]. SNPs related to MPN and plasma metabolites were selected at conventional genome-wide association study (GWAS) thresholds (*p* < 5 × 10^−8^). Independent SNPs were clumped using a linkage disequilibrium (LD) threshold of r2 < 0.001 with the 1000 Genomes reference panel [24]. SNPs with an effect allele frequency > 0.01 and F-statistic ≥ 10 were included to ensure strong instrumental bias avoidance [25].

### 2.4. Statistical Analysis Strategy

Initially, we conducted univariate MR analysis to assess causal links between GM and MPN, as well as between plasma metabolites and MPN. Depending on the SNP count, different methods were employed: polygenic MR and inverse variance weighted (IVW) methods were used for SNP counts greater than 1, while cis-MR and Wald ratio models were applied when SNPs equaled 1 [26]. To evaluate heterogeneity and pleiotropy, we used Cochran’s Q test and MR-Egger’s intercept, with a non-zero MR-Egger intercept (*p* < 0.05) indicating potential horizontal pleiotropy [27,28].

For analyzing pathways from exposures to outcomes via mediators, we utilized mediation MR analysis. Two-step Mendelian randomization (TSMR) was applied to separate direct and indirect effects of GM and plasma metabolites on MPN. TSMR assumes no interaction between exposure and mediator. Alongside basic univariate MR estimates, we calculated: (1) the effect of the mediator (17 plasma metabolites) on MPN, and (2) the effect of the exposure (7 significant taxa) on the mediator. The mediation effect proportion was determined using the following formula: β_M = (β_EM × β_MO)/β_EO [29,30]. Here, β_M represents the mediation proportion, β_EM is the causal effect of exposure E on mediator M, β_MO is the effect of mediator M on outcome O, β_EM × β_MO denotes the ‘indirect’ effect, and β_EO is the ‘total’ effect of exposure E on outcome O. Multivariate MR was conducted to pinpoint key exposures affecting the mediator.

To validate the genetic causality of MR-identified GM and plasma metabolites in MPN pathogenesis and exclude confounding by linkage disequilibrium, Bayesian colocalization analysis was performed using summary statistics from GM, metabolites and MPN GWAS. SNPs within ±500 kb of genes were analyzed via the “coloc” R package (4.2.0), evaluating five hypotheses (H0–H4), with a focus on H4. A posterior probability for H4 (PPH4) > 75% was considered strong evidence of colocalization. Visualization using the “LocusCompareR” package highlighted shared genetic architecture at key loci.

All MR analyses were performed in R (version 4.3.1; R Foundation for Statistical Computing, Vienna, Austria) using the “TwoSampleMR”, “tidyverse”, “ggplot2”, “purr”, and “data.table” packages.

### 2.5. Ethical Approval and Consent to Participate

This study utilized publicly available data, with each GWAS included in the study having received approval from the relevant Institutional Review Board. Informed consent was obtained from participants, caregivers, legal guardians, or proxies as applicable.

## 3. Results

### 3.1. Genetic Instrumentation

We identified 3–18 SNPs (median = 10) as IVs for 196 GM taxa from MiBioGen, 1-26 SNPs (median = 5) for 526 metabolites from OpenGWAS, and 13 SNPs for MPN from Helmholtz Munich (Supplementary Tables S2 and S3). Median F-statistics confirmed the robust instrument strength (GM: 21.0, range 14.6–88.6; metabolites: 49.1, range 28.8–3938.3), exceeding the recommended threshold of 10 for MR validity.

### 3.2. Causal GM Taxa Associated with MPN

**Figure 2:**
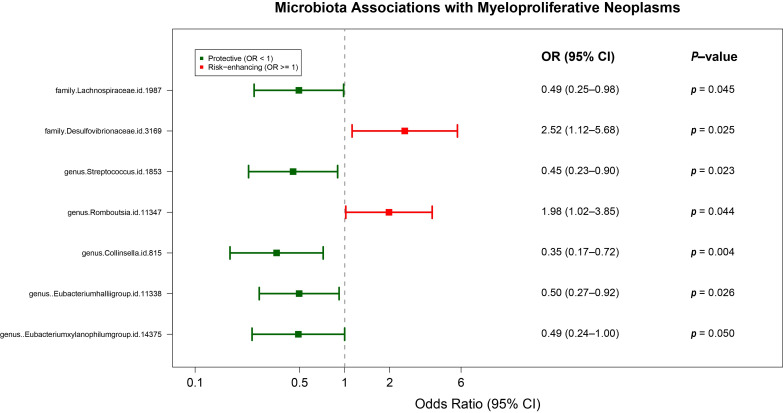
Mendelian randomization analyses show the causal effects of gut microbiota on MPN. CI indicates confidence intervals and *p*-values were calculated by IVW method.

MR analysis revealed seven GM taxa causally linked to MPN (Figure 2, Supplementary Figure S1, and Supplementary Table S4). Protective effects were observed for Lachnospiraceae (OR = 0.49, 95% CI: 0.25–0.98), Streptococcus (OR = 0.45, 95% CI: 0.23–0.90), Collinsella (OR = 0.35, 95% CI: 0.17–0.72), Eubacterium hallii group (OR = 0.50, 95% CI: 0.27–0.92), and *Eubacterium xylanophilum group* (OR = 0.49, 95% CI: 0.24–1.00). Conversely, Desulfovibrionaceae (OR = 2.52, 95% CI: 1.12–5.68) and Romboutsia (OR = 1.98, 95% CI: 1.02–3.85) exhibited risk-enhancing associations. Taxa were phylogenetically distributed across Pseudomonadota (Desulfovibrionaceae), Actinomycetota (Collinsella), and Bacillota (remaining taxa), with Desulfovibrionaceae showing the strongest risk association. Reverse-direction MR analysis revealed no significant effects of MPN on these seven GM taxa (Supplementary Table S5). MR-Egger and Cochrane Q analyses revealed negligible pleiotropy and heterogeneity (Supplementary Tables S6 and S7).

**Figure 3:**
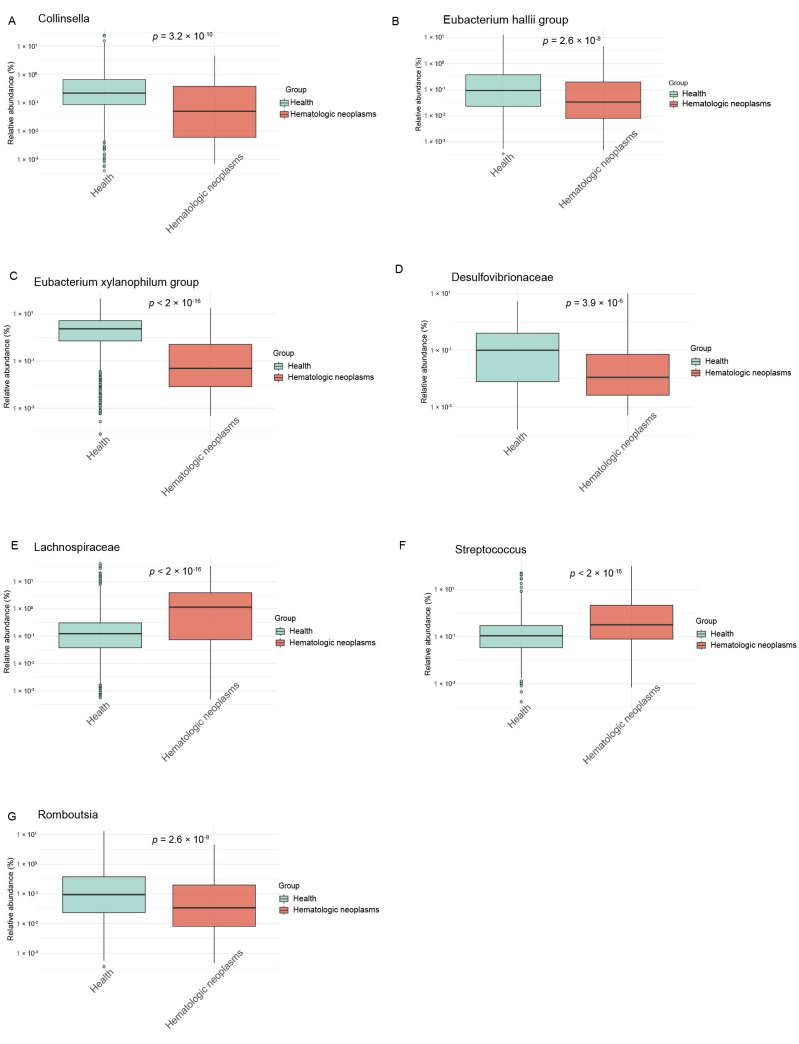
Differential abundance analysis of gut microbiota in hematologic neoplasms. Comparative visualization of bacterial taxa between the hematologic neoplasm cohort (*n* = 326) and healthy controls (*n* = 109) from the NCBI Bioproject PRJNA376506. (**A**) *Collinsella*, (**B**) *Eubacterium hallii group*, (**C**) *Eubacterium xylanophilum group*, (**D**) *Desulfovibrionaceae*, (**E**) *Lachnospiraceae*, (**F**) *Streptococcus*, (**G**) *Romboutsia*.

To validate the causal associations identified by Mendelian randomization, we quantified the relative abundance of seven bacterial taxa in hematologic neoplasm patients using a publicly available microbiome dataset. The analysis revealed significant intergroup differences in microbial composition, with notably reduced abundances of Collinsella, Eubacterium hallii group, and *Eubacterium xylanophilum group* in the hematologic neoplasms cohort compared to healthy controls (*p* < 0.001; Figure 3). This observation aligns with the MR-derived protective effects of these taxa (OR range: 0.35–0.50), strengthening the evidence for their causal protective role against MPN pathogenesis. Notably, the consistency between the directionality of effect estimates (MR analysis) and empirical abundance measurements (validation cohort) suggests minimal confounding in these associations.

### 3.3. Causal Plasma Metabolites Associated with MPN

**Figure 4:**
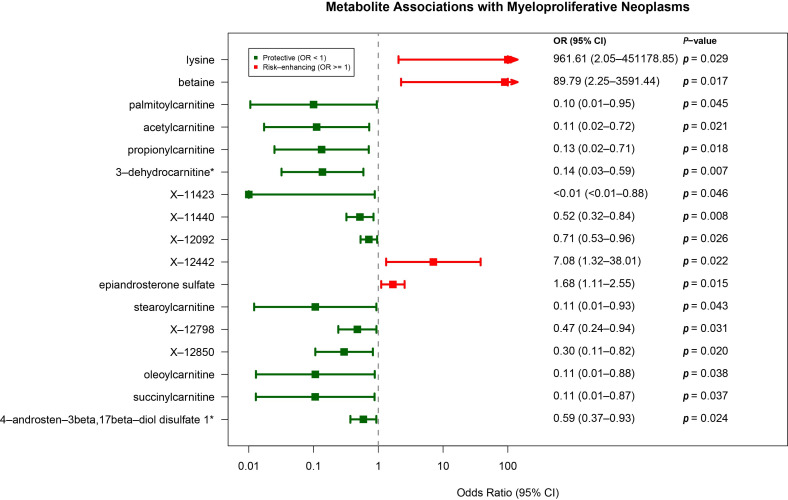
Mendelian randomization analyses show the causal effect of plasma metabolites on MPN. CI indicates confidence intervals and *p*-values were calculated by IVW method.* indicates putatively annotated metabolites whose exact chemical structures are not fully confirmed.

Seventeen plasma metabolites showed significant causal relationships with MPN (Figure 4, Supplementary Table S8). Elevated lysine (OR = 961.61, 95% CI: 2.05–451178.85), X-12442 (OR = 7.08, 95% CI: 1.32–38.01), betaine (OR = 89.79, 95% CI: 2.25–3591.44), and epiandrosterone sulfate (OR = 1.68, 95% CI: 1.11–2.55) increased MPN risk. Protective effects were observed for 13 metabolites, including 3-dehydrocarnitine (OR = 0.14, 95% CI: 0.03–0.59), propionyl carnitine (OR = 0.10, 95% CI: 0.01–0.95), and O-sulfo-L-tyrosine (X-11423). Metabolites spanned amino acid (betaine, lysine, X-11423), lipid (3-dehydrocarnitine, acetyl carnitine), and energy metabolism (succinylcarnitine) categories. No pleiotropy or heterogeneity was detected (Supplementary Tables S6 and S7).

### 3.4. Mediation Analysis

**Figure 5:**
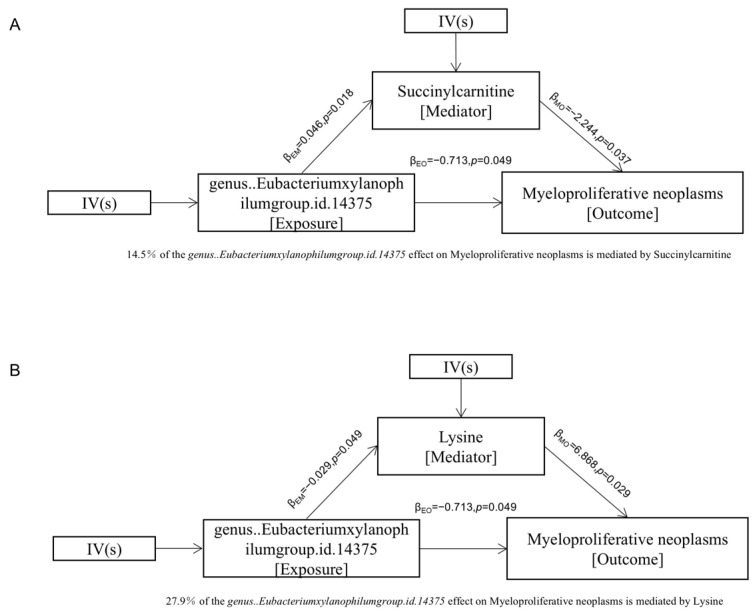
Metabolites mediate the effects of bacteria on MPN. is the proportion of mediation effect of the mediator M, is the MR casual effect of exposure E on mediator M, is the MR casual effect of mediator M on outcome O, and is the ‘total’ effect of exposure E on outcome O. The proportion of mediation effect is expressed as a percentage. The *p* values were calculated by IVW method. (**A**) succinylcarnitine, (**B**) lysine.

Mediation analysis employing a three-step causal model revealed that *Eubacterium xylanophilum group* causally influenced myeloproliferative neoplasm (MPN) risk through two key microbial metabolites: succinylcarnitine (an acylcarnitine derivative, OR = 1.05, 95% CI: 1.01–1.09) and lysine (an essential amino acid, OR = 0.97, 95% CI: 0.94–0.99) (Table 1; Supplementary Table S9). The proportion of total effect mediated reached 14.5% for succinylcarnitine and 27.9% for lysine (Figure 5A,B). Notably, the direction and magnitude of effect estimates in mediation models may differ from those in univariable MR analyses, owing to differences in model structure, instrumental variable selection, and effect scaling. For example, while univariable MR suggested that elevated lysine levels were associated with a markedly increased risk of MPN (OR = 961.61), the mediation analysis evaluating the pathway from *Eubacterium xylanophilum* to MPN through lysine showed a modest inverse association for lysine (OR = 0.97). This discrepancy may reflect distinct biological mechanisms captured in the mediation context, or potential residual confounding inherent in complex multivariable models.

Multivariable Mendelian randomization analysis adjusted for age, sex, and inflammatory markers confirmed the independent causal effects of both metabolites on MPN pathogenesis: succinylcarnitine (*p* = 0.004) and lysine (*p* = 0.040) (Table 2).

Furthermore, bidirectional mediation analysis revealed six gut microbial taxa (genus Collinsella, Streptococcus, Romboutsia, *Eubacterium xylanophilum group*, and families Desulfovibrionaceae and Lachnospiraceae) that significantly mediated the metabolic pathways linking 11 host–microbial co-metabolites (including acylcarnitines and steroid derivatives) to MPN risk (Supplementary Figure S2 and Supplementary Table S10).

## 4. Discussion

This MR study elucidates putative causal associations between GM taxa, plasma metabolites, and MPN. We identified seven GM taxa and seventeen metabolites with causal links to MPN risk, mediated in part by *Eubacterium xylanophilum group* interactions with succinylcarnitine and lysine. These findings advance our understanding of gut–microbiome–metabolome crosstalk in MPN pathogenesis.

Notably, the risk-enhancing association of Desulfovibrionaceae (Pseudomonadota phylum) aligns with its documented role in chronic myeloid leukemia progression, while protective taxa, Lachnospiraceae, Streptococcus, and Collinsella, mirror their established anti-tumor effects in immunotherapy contexts [31,32,33,34]. The contrasting MPN association of Romboutsia (risk-enhancing) further parallels its hepatocarcinogenic potential [35], underscoring conserved microbial influences across hematologic and solid malignancies.

Regarding plasma metabolites, our study identified specific metabolites like palmitoyl carnitine, which was negatively associated with MPN, in line with previous research [36]. Seven metabolites involved in carnitine metabolism were significantly associated with MPN, corroborating prior findings of reduced carnitine levels in MPN patients [37,38]. Given carnitine’s roles in mitochondrial function and thrombosis mitigation [39,40,41,42,43,44], these results suggest metabolic dysregulation may contribute to MPN-related complications. Importantly, Zhang et al. [42] reported that in acute inflammatory states, such as ACLF, mitochondrial breakpoints at isocitrate and succinate dehydrogenases disrupt TCA continuity, with compensatory shifts in acylcarnitine metabolism. Analogously, chronic inflammation and oxidative stress in MPN may impair mitochondrial function, and the observed succinylcarnitine perturbation could serve as a surrogate of disrupted immunometabolism. Conversely, the markedly elevated lysine levels (OR = 961.61) may reflect its dual role not only in redox balance but also in epigenetic immune regulation. Lysine-driven histone crotonylation has been shown to modulate tumor immunity via type I interferon signaling, a mechanism potentially relevant to MPN-associated inflammation [45,46,47].

Our mediation analysis enriched our understanding of the interactions between the gut microbiome and plasma metabolites. While direct links between *Eubacterium xylanophilum group* and specific metabolites like succinylcarnitine or lysine have not been previously reported, related studies have explored connections between gut microbiota and apolipoproteins. Succinylcarnitine, an energy metabolite, has been studied for its potential in treating neurological disorders and its association with gut microbiome changes [48,49,50]. Lysine accumulation in aging brains suggests that microbial dysbiosis might contribute to inflammation and oxidative stress, providing new insights into the regulation of amino acid and energy metabolism in MPN [51].

In this study, we designated 196 taxa and 526 metabolites as exposure factors, with MPN as the outcome variable. Subsequently, we stratified the MPN cohort based on the selected independent variables for each exposure factor. Analyzing the variations in MPN risk between exposed and control groups allowed us to infer the causal relationship between the exposure factors and MPN. Our study showed that MR analysis could sort out some GM and metabolites that have a potential relationship with MPN. In addition, by combining two-stage MR analysis and mediation analysis, we successfully linked GM and plasma metabolome, and constructed some pathways from intestinal bacteria to MPN through plasma metabolites or from plasma metabolites to MPN through intestinal bacteria.

Based on these findings, we propose that a composite “carnitine score” derived from the most robustly associated carnitine metabolites (e.g., succinylcarnitine, palmitoylcarnitine, etc.) could serve as a candidate biomarker panel for early MPN detection or progression monitoring. Prospective validation in well-characterized MPN cohorts is warranted to assess its diagnostic performance and clinical utility.

Despite the valuable insights, our study has limitations. Causal inference results regarding intestinal bacteria may be influenced by confounding factors like race, diet, disease status, and impact of medications. The absence of medication data in GWAS summary statistics precludes direct adjustment for treatment effects. Future studies integrating electronic health records with metagenomic data are needed to disentangle drug–microbiome interactions. Co-localization analysis did not reveal high posterior probabilities (PPH4 < 0.5), suggesting that the regulatory variants underlying microbial or metabolite traits and MPN risk may differ, or that the current resolution of GWAS and QTL datasets is insufficient to detect shared causal signals. These findings do not invalidate the MR results but rather highlight the complexity of genetic architecture and potential tissue-specific regulation (e.g., gut vs. hematopoietic) that may limit colocalization power (Supplementary Figure S3). Additionally, the absence of experimental validation necessitates cautious interpretation of our observed causal associations. Moreover, given that *Eubacterium xylanophilum* utilizes dietary xylan as a substrate, dietary fiber supplementation may represent a tractable intervention to modulate its abundance and downstream metabolic effects. Future preclinical and clinical studies are needed to evaluate whether targeting this microbial–metabolite axis could alter disease course or symptom burden in MPN patients. In addition, stratifying microbial and metabolite associations by MPN subtypes (polycythemia vera, essential thrombocythemia, and myelofibrosis) could uncover subtype-specific biomarkers or mechanisms. Publicly available datasets, such as PRJNA376506, offer opportunities to explore this hypothesis and should be incorporated in future investigations. Future mechanistic studies are crucial for validating these relationships and comprehensively understanding the intricate connections between the GM, plasma metabolites, and MPN. Integrating somatic mutation profiles with multi-omics data could unravel mutation-specific crosstalk between host genomics, gut microbiota, and metabolites. Prospective cohorts with annotated mutation status are needed to validate these genotype-dependent interactions.

## 5. Conclusions

This first GM–metabolome–MPN MR study identifies candidate microbial and metabolic biomarkers with diagnostic and therapeutic potential. Specifically, we propose a carnitine-based biomarker panel for validation in clinical cohorts, and highlight *Eubacterium xylanophilum* as a modifiable taxon via dietary fiber interventions. These findings lay the groundwork for precision oncology strategies in MPN management, including subtype stratification and microbiome-informed therapeutic modulation. These proposals provide a translational bridge from genomic findings to clinical hypothesis generation, warranting follow-up in experimental and observational settings.

## Figures and Tables

**Table 1:** Mediation analyses of the causal effects between gut microbiota, blood metabolites and MPN.

Mediated	Exposure	Method	Beta	SE	P	Q-Statistics	P_h_	Egger Intercept	P_pleio_
Succinyl carnitine	*Eubacterium* *Xylanophilum* *group*	Inverse variance weighted	0.046	0.019	0.0184	2.845	0.416	NA	NA
		MR Egger	0.126	0.059	0.169	0.832	0.659	−0.006	0.291
Lysine		Inverse variance weighted	−0.029	0.014	0.049	0.231	0.972	NA	NA
		MR Egger	−0.031	0.042	0.543	0.227	0.892	0.001	0.957

Note: Beta, effect value calculated by MR analysis; SE, standard error; P, MR analysis *p* value; Q-statistics, Cochrane Q-statistics value; P_h_, *p*-value for heterogeneity; Pleio, *p*-value for the intercept of the MR-Egger regression.

**Table 2:** Multivariate Mendel randomization analysis of *Eubacterium xylanophilum* *group*-related metabolites.

Exposure	Univariate				Multivariate			
	Beta	SE	*P*	OR (95% CI)	Beta	SE	*P*	OR (95% CI)
Lysine	6.868	3.138	0.028	961.61 (2.05 to 451,178.85)	5.714	2.780	0.039	303.18 (1.30 to 70,536.17)
Succinylcarnitine	−2.244	1.076	0.037	0.11 (0.01 to 0.87)	−2.342	0.823	0.004	0.10 (0.02 to 0.48)

Note: Beta, effect value calculated by MR analysis; SE, standard error; OR, odds ratio; CI, confidence interval.

## Data Availability

The datasets used and/or analyzed during the current study are available from the corresponding author upon reasonable request.

## Supplementary Materials

The following are available online at https://www.mdpi.com/article/10.3390/metabo15080501/s1, Figure S1: Scatter plots generated from MR analyses for the effect of (A) Lachnospiraceae, (B) Desulfovibrionaceae, (C) Streptococcus, (D) Romboutsia, (E) Collinsella, (F) Eubacterium hallii group, (G) Eubacterium xylanophilum group on MPN, FigureS2: Bacteria mediate the effects of metabolites on MPN, Figure S3: Regional association plot for colocalization analysis of bacteria/metabolites with myeloproliferative neoplasms risk, Table S1: Breakdown of MPN sub-types, Table S2: Used instrumental variables for 196 gut microbiota taxa from MiBioGen, Table S3: Used instrumental variables for 526 blood metabolites from MetabolomIPS database, Table S4: Five Mendelian randomization models estimate the causal effects of gut microbiota on Myeloproliferative neoplasms, Table S5: Five Mendelian randomization models estimate the causal effects of myeloproliferative neoplasms on gut microbiota, Table S6: Pleiotropy of all Mendelian randomization results, Table S7: Heterogeneity of all Mendelian randomization results, Table S8: Five Mendelian randomization models estimate the causal effects of plasma metabolites on Myeloproliferative neoplasms, Table S9: Five Mendelian randomization models estimate the causal effects of gut microbiota on blood metabolites, Table S10: Five Mendelian randomization models estimate the causal effects of blood metabolites on gut microbiota.
