# Supplementary material for: The Causal Role of the Gut Microbiota–Plasma Metabolome Axis in Myeloproliferative Neoplasm Pathogenesis: A Mendelian Randomization and Mediation Analysis
Source: Metabolites. 2025 Jul 28;15(8):501. doi: 10.3390/metabo15080501 (PMC12388087; doi:10.3390/metabo15080501)
Supplement: Supplementary file 1 [file metabolites-15-00501-s001.zip › metabolites-3721777-supplementary.pdf]

Supplemental Material

Supplemental Methods

Gut Microbiome Relative Abundance Analysis

The analysis of seven bacterial taxa (*Lachnospiraceae, Streptococcus, Collinsella, Eubacterium hallii group, Eubacterium xylanophilum group, Desulfovibrionaceae, Romboutsia*) between hematologic neoplasms patients (n=326) and healthy controls (n=109) was performed using 16S rRNA sequencing data from NCBI Bioproject PRJNA376506. Raw FASTQ files were preprocessed with Trimmomatic (Q20 threshold, sliding window: 4 bp) for quality filtering, followed by paired-end read merging via FLASH (minimum overlap: 15 bp). Chimeric sequences were removed using UCHIME in de novo mode, and denoised amplicon sequence variants (ASVs) were generated through DADA2 in QIIME2 (v2024.6). Taxonomic annotation was conducted against the SILVA 138.1 database (97% similarity). Relative abundances of the seven target taxa were calculated as (ASV counts per taxon / total reads per sample) × 100%, with rarefaction to 10,000 reads/sample to mitigate sequencing depth bias. Wilcoxon rank-sum tests (non-parametric) assessed intergroup differences, and P-values were adjusted for multiple comparisons using the Benjamini-Hochberg FDR method. Boxplots were generated in R/ggplot2 (v3.5.0) with a unified color scheme (#91D1C2B2 for controls, #E64B35B2 for cancer), outliers suppressed for clarity, and significance markers ($P < 0.05$) annotated via ggsignif.

Supplemental Figures for
Causal Role of Gut Microbiota-Plasma Metabolome Axis in Myeloproliferative Neoplasm Pathogenesis: A Mendelian Randomization and Mediation Analysis

Supplementary Figure 1. Scatter plots generated from MR analyses for the effect of (A) *Lachnospiraceae*, (B) *Desulfovibrionaceae*, (C) *Streptococcus*, (D) *Romboutsia*, (E) *Collinsella*, (F) *Eubacterium hallii group*, (G) *Eubacterium xylanophilum group* on MPN.

Supplementary Figure 2. Bacteria mediate the effects of metabolites on MPN. (A-T) The mediating effect of 11 serum metabolites on MPN was mediated by 6 bacteria.

Supplementary Figure 3. Regional association plot for colocalization analysis of bacteria/metabolites (A-X) with myeloproliferative neoplasms risk. The lead SNP is shown as a purple diamond. SNPs within ± 500kb of the protein quantitative trait locus were included; p12 = $1e^{-5}$, prior probability a SNP is associated with both protein and MPN. No loci showed moderate or strong colocalization (PPH4 > 0.5). This likely reflects resolution and LD differences across datasets, as discussed in the main text.

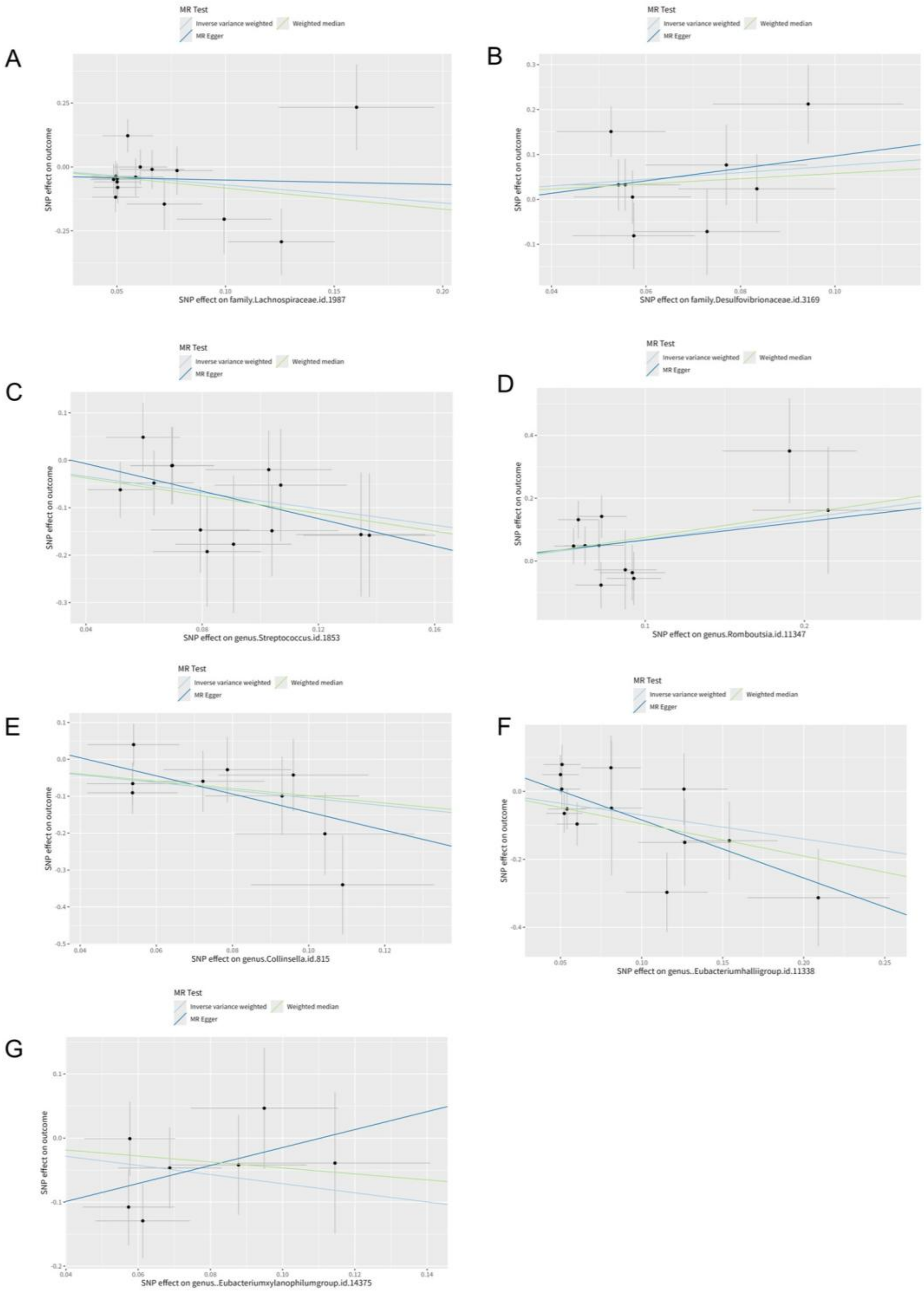
MR Test
Inverse variance weighted
Weighted median
MR Egger
A
SNP effect on outcome
SNP effect on family.Lachnospiraceae.id.1987
B
SNP effect on family.Desulfovibrionaceae.id.3169
C
SNP effect on genus.Streptococcus.id.1853
D
SNP effect on genus.Romboutsia.id.11347
E
SNP effect on genus.Collinsella.id.815
F
SNP effect on genus..Eubacteriumhalliigroup.id.11338
G
SNP effect on genus..Eubacteriumxylanophilumgroup.id.14375

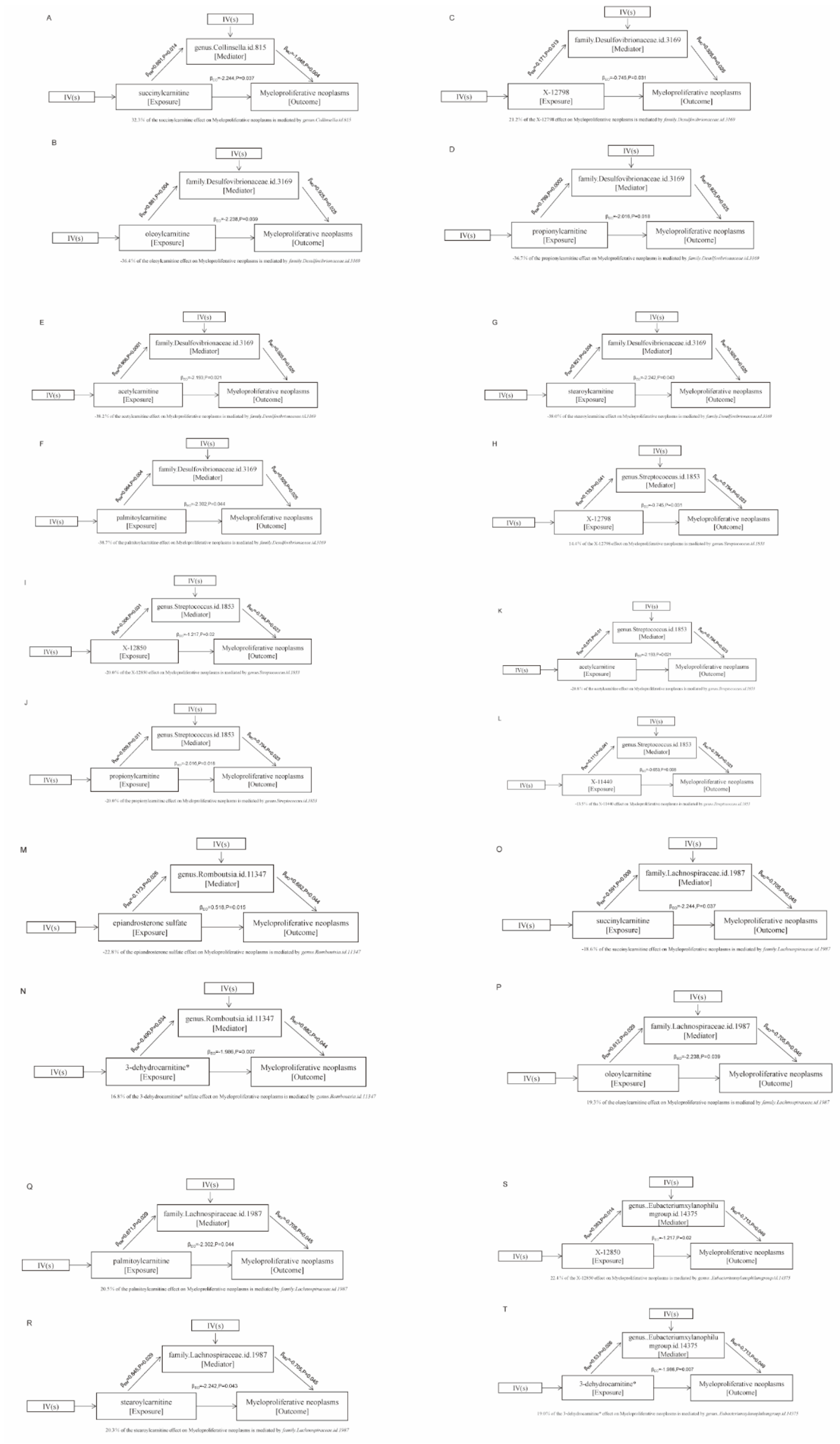

A
IV(s)
genus.Collinsella.id.815 [Mediator]
βEM=0.891,P=0.014
βMO=-1.048,P=0.004
βEO=-2.244,P=0.037
succinylcarnitine [Exposure]
Myeloproliferative neoplasms [Outcome]
32.3% of the succinylcarnitine effect on Myeloproliferative neoplasms is mediated by genus.Collinsella.id.815
B
family.Desulfovibrionaceae.id.3169 [Mediator]
βEM=0.861,P=0.004
βMO=0.925,P=0.025
βEO=-2.238,P=0.039
oleoylcarnitine [Exposure]
-36.4% of the oleoylcarnitine effect on Myeloproliferative neoplasms is mediated by family.Desulfovibrionaceae.id.3169
C
βEM=-0.171,P=0.013
βMO=0.925,P=0.025
βEO=-0.745,P=0.031
X-12798 [Exposure]
21.2% of the X-12798 effect on Myeloproliferative neoplasms is mediated by family.Desulfovibrionaceae.id.3169
D
βEM=0.799,P=0.0002
βMO=0.925,P=0.025
βEO=-2.016,P=0.018
propionylcarnitine [Exposure]
-36.7% of the propionylcarnitine effect on Myeloproliferative neoplasms is mediated by family.Desulfovibrionaceae.id.3169
E
acetylcarnitine [Exposure]
G
stearoylcarnitine [Exposure]
F
palmitoylcarnitine [Exposure]
H
genus.Streptococcus.id.1853 [Mediator]
X-12798 [Exposure]
I
X-12850 [Exposure]
K
acetylcarnitine [Exposure]
J
propionylcarnitine [Exposure]
L
X-11440 [Exposure]
M
genus.Romboutsia.id.11347 [Mediator]
epiandrosterone sulfate [Exposure]
O
family.Lachnospiraceae.id.1987 [Mediator]
succinylcarnitine [Exposure]
N
3-dehydrocarnitine* [Exposure]
P
oleoylcarnitine [Exposure]
Q
palmitoylcarnitine [Exposure]
S
genus..Eubacteriumxylanophilumgroup.id.14375 [Mediator]
X-12850 [Exposure]
R
stearoylcarnitine [Exposure]
T
3-dehydrocarnitine* [Exposure]

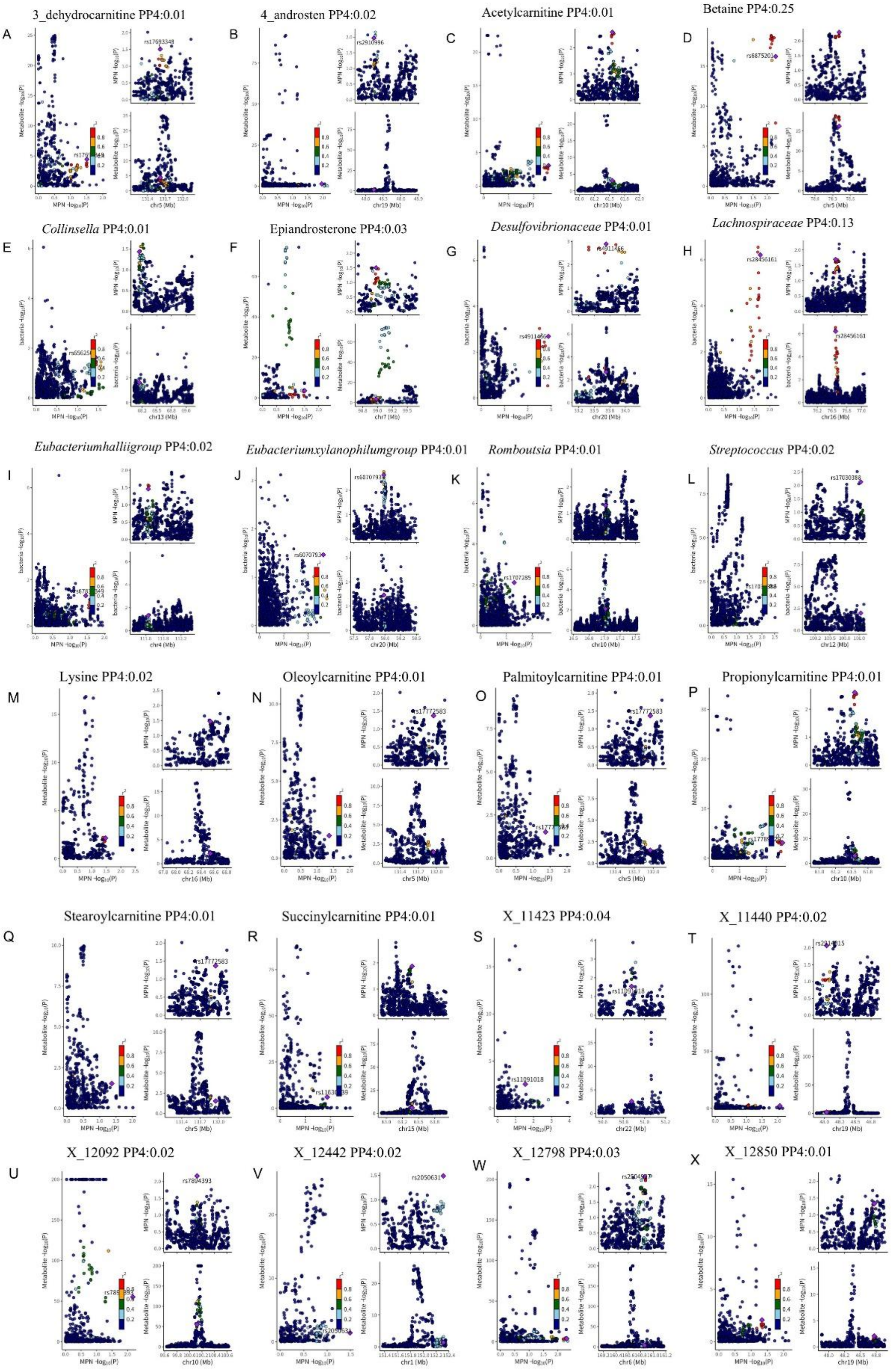
A 3_dehydrocarnitine PP4:0.01
B 4_androsten PP4:0.02
C Acetylcarnitine PP4:0.01
D Betaine PP4:0.25
E Collinsella PP4:0.01
F Epiandrosterone PP4:0.03
G Desulfovibrionaceae PP4:0.01
H Lachnospiraceae PP4:0.13
I Eubacteriumhalliigroup PP4:0.02
J Eubacteriumxylanophilumgroup PP4:0.01
K Romboutsia PP4:0.01
L Streptococcus PP4:0.02
M Lysine PP4:0.02
N Oleoylcarnitine PP4:0.01
O Palmitoylcarnitine PP4:0.01
P Propionylcarnitine PP4:0.01
Q Stearoylcarnitine PP4:0.01
R Succinylcarnitine PP4:0.01
S X_11423 PP4:0.04
T X_11440 PP4:0.02
U X_12092 PP4:0.02
V X_12442 PP4:0.02
W X_12798 PP4:0.03
X X_12850 PP4:0.01

Supplementary Table 1. Breakdown of MPN sub-types.

Notations:PV, polycythemia vera only; ET, essential thrombocythemia only; MF, primary myelofibrosis only; CML, chronic myelogenous leukemia only; Multiple, some combination of PV, ET, MF, and CML; JAK2 V617F, carriers of somatic JAK2 V617F mutation; Other, unspecified chronic myeloproliferative disease,malignant mastocytosis; Total, total MPN cases across all sub-types.Multiple: some combination of PV, ET, MF, and CML.Other: Chronic myeloproliferative disease, malignant mastocytosis

	PV	ET	MF	CML	Multiple	Other	Total
**UKBB**	385	246	45	266	96	48	1086

Supplementary Table 2. Used instrumental variables for 196 gut microbiota taxa from MiBioGen

Notations: SNP, Single nucleotide polymorphism; R^2, proportion of variance in exposure variable explained by SNPs

Taxonomy	SNP	Chromosome	Other_alle	Effect_allele	Beta	Standard error	P-value	Sample size	R^2	F-statistic
class.Actinobacteria.id.419	rs182549	2	T	C	0.111489458	0.012065989	3.79006E-20	16313	0.00520644	85.37716722
class.Actinobacteria.id.419	rs7570971	2	C	A	0.106845585	0.011924282	2.56268E-19	16182	0.004937044	80.28763641
class.Actinobacteria.id.419	rs1530559	2	A	G	0.065463239	0.011429659	7.54604E-09	16182	0.002023097	32.80412236
class.Actinobacteria.id.419	rs6660520	1	G	A	0.071148271	0.013449539	1.10652E-07	16308	0.001713044	27.98426588
class.Actinobacteria.id.419	rs12022129	1	A	G	0.069756094	0.013264752	1.21408E-07	16310	0.001692686	27.65451619
class.Actinobacteria.id.419	rs8047955	16	G	A	0.05758155	0.011676216	8.33727E-07	16306	0.00148925	24.3199362
class.Actinobacteria.id.419	rs7322849	13	C	T	0.094446681	0.019297598	6.20794E-07	16313	0.001466209	23.95338453
class.Actinobacteria.id.419	rs72767435	15	C	T	-0.126352156	0.027362598	2.56804E-06	12898	0.001650482	21.32311576
class.Actinobacteria.id.419	rs134366	22	A	G	0.111876589	0.023500975	1.50428E-06	14302	0.001582058	22.66244034
class.Actinobacteria.id.419	rs10841473	12	C	G	-0.058376469	0.012335331	2.5936E-06	16311	0.00137119	22.39618455
class.Actinobacteria.id.419	rs11745923	5	T	G	0.056375863	0.011552908	1.58131E-06	16312	0.001457683	23.81243166
class.Actinobacteria.id.419	rs1515761	10	C	T	0.076231731	0.016988402	4.96362E-06	15525	0.001295305	20.1356896
class.Actinobacteria.id.419	rs12899991	15	T	A	0.071992324	0.015466108	4.02818E-06	16182	0.001337202	21.66757561
class.Actinobacteria.id.419	rs12049045	1	G	A	0.05104521	0.01143249	8.62622E-06	16311	0.001220723	19.93555402
class.Actinobacteria.id.419	rs1376754	2	A	G	0.050924155	0.011250292	6.71385E-06	16182	0.001264557	20.48896701
class.Actinobacteria.id.419	rs11655079	17	C	T	-0.056067443	0.01242856	5.92512E-06	16309	0.001246267	20.35072568
class.Actinobacteria.id.419	rs7700479	5	A	T	-0.076965305	0.017393272	8.62148E-06	16313	0.001198871	19.5806637
class.Actinobacteria.id.419	rs80083040	8	G	T	0.155783797	0.034801661	8.62105E-06	8003	0.002497502	20.03755082
class.Actinobacteria.id.419	rs961091	7	A	G	0.050125533	0.01125498	8.67593E-06	16311	0.001214563	19.83483216
class.Actinobacteria.id.419	rs4945008	11	G	A	0.054074066	0.012139345	5.39237E-06	16313	0.001214859	19.84209555
class.Actinobacteria.id.419	rs857444	6	T	C	0.050955263	0.011539048	8.92233E-06	16312	0.001194021	19.50015784
class.Actinobacteria.id.419	rs4957061	5	C	T	0.049600191	0.011177342	9.66398E-06	16182	0.001215428	19.69199128
class.Alphaproteobacteria.id.2379	rs9813022	3	G	A	-0.075127704	0.015386652	1.05333E-06	9593	0.002479017	23.8403131
class.Alphaproteobacteria.id.2379	rs76784716	2	G	A	0.133271299	0.026773761	5.09141E-07	9593	0.002576201	24.77732936
class.Alphaproteobacteria.id.2379	rs140912403	9	T	C	-0.160677133	0.031770756	6.20363E-07	8520	0.002993034	25.57720309
class.Alphaproteobacteria.id.2379	rs34569731	2	G	A	0.070514454	0.015756373	7.37916E-06	9115	0.002192471	20.02828699
class.Alphaproteobacteria.id.2379	rs10803434	1	T	A	-0.069274224	0.01488663	3.55434E-06	9115	0.002370082	21.65461818
class.Alphaproteobacteria.id.2379	rs55876211	3	T	C	-0.080810954	0.01841593	7.53639E-06	9589	0.00200405	19.255428
class.Alphaproteobacteria.id.2379	rs7960664	12	A	G	0.096948894	0.021514853	8.84301E-06	9596	0.002111547	20.3052793
class.Alphaproteobacteria.id.2379	rs17061716	3	C	G	0.092734145	0.020756923	9.33258E-06	9465	0.00210435	19.95967249
class.Alphaproteobacteria.id.2379	rs12977163	19	C	G	0.069023356	0.015382895	7.14185E-06	9115	0.002203947	20.13334825
class.Alphaproteobacteria.id.2379	rs62285697	3	T	C	0.080525582	0.018091166	9.75933E-06	9115	0.002168877	19.81228778
class.Bacilli.id.1673	rs2952251	8	G	A	-0.059886787	0.012390272	1.07895E-06	17360	0.001343899	23.36147851
class.Bacilli.id.1673	rs77558518	5	G	A	-0.107254772	0.022287875	1.33772E-06	15836	0.001460212	23.15772628
class.Bacilli.id.1673	rs57872228	1	T	C	-0.071477336	0.014664695	9.21977E-07	17375	0.001365439	23.75694704
class.Bacilli.id.1673	rs35344081	16	A	G	0.061952894	0.012706646	1.01498E-06	17374	0.001366368	23.77175844
class.Bacilli.id.1673	rs78938557	7	C	T	0.108007225	0.023285767	1.06891E-06	15226	0.001410995	21.51416707
class.Bacilli.id.1673	rs11730038	4	A	G	-0.063074683	0.012871737	1.96025E-06	17377	0.001379944	24.01241503
class.Bacilli.id.1673	rs76717940	3	A	T	0.156331484	0.032594546	1.0725E-06	8017	0.002861191	23.00398315
class.Bacilli.id.1673	rs17708276	8	G	A	-0.078791463	0.016612349	1.84098E-06	17266	0.001301185	22.49553141
class.Bacilli.id.1673	rs1962325	3	G	C	0.055421868	0.011478925	1.38434E-06	17356	0.001341304	23.31094362
class.Bacilli.id.1673	rs74352383	6	A	T	-0.104495633	0.021708314	1.52752E-06	16519	0.001400721	23.17096398
class.Bacilli.id.1673	rs9581006	13	T	C	0.225309054	0.046769705	1.79458E-06	4253	0.005427118	23.20748253
class.Bacilli.id.1673	rs11110282	12	G	A	-0.101189316	0.021737883	4.85442E-06	16026	0.001350277	21.66879879
class.Bacilli.id.1673	rs4028634	17	C	T	0.052061417	0.010984163	2.2076E-06	17377	0.001291107	22.46456543
class.Bacilli.id.1673	rs11110281	12	C	T	-0.1011638	0.022182046	7.39393E-06	15915	0.001305188	20.79922059
class.Bacilli.id.1673	rs694949	15	G	A	-0.081145978	0.018004225	7.59585E-06	16600	0.00122221	20.31351739
class.Bacilli.id.1673	rs12797734	11	C	T	0.057215761	0.012679292	7.21166E-06	16902	0.001203318	20.3629798
class.Bacilli.id.1673	rs28564647	9	G	T	-0.061303431	0.013757893	7.81199E-06	17368	0.001141878	19.85481007
class.Bacilli.id.1673	rs7666190	4	A	C	-0.104044367	0.024793431	8.46829E-06	10436	0.001684603	17.61018359
class.Bacilli.id.1673	rs9345899	6	G	A	-0.080243842	0.017534638	6.67407E-06	16600	0.001260011	20.94256355
class.Bacilli.id.1673	rs4459992	4	C	T	0.053585401	0.011636181	4.29836E-06	17362	0.00121995	21.20664697
class.Bacilli.id.1673	rs34989881	19	G	A	0.111055893	0.024603898	6.54586E-06	12966	0.001568872	20.37395685
class.Bacilli.id.1673	rs111552159	4	G	C	0.128031637	0.028692104	9.12818E-06	11350	0.001751268	19.91176712
class.Bacilli.id.1673	rs13068444	3	G	A	0.060103454	0.013640742	9.53048E-06	17260	0.001123554	19.41435018
class.Bacilli.id.1673	rs1595463	2	A	C	0.04768644	0.010854727	7.9738E-06	17358	0.001110631	19.29976453
class.Bacilli.id.1673	rs74663707	3	T	C	0.097915445	0.022407373	8.45686E-06	15769	0.001209458	19.0950383
class.Bacteroidia.id.912	rs55773148	13	A	G	-0.121513597	0.023675903	3.89585E-07	11349	0.002315646	26.34126632
class.Bacteroidia.id.912	rs7631304	3	A	G	-0.064577053	0.013295739	8.36781E-07	18334	0.001285038	23.59019404
class.Bacteroidia.id.912	rs73512608	13	A	G	-0.121371693	0.023675946	4.07905E-07	11349	0.002310245	26.27968294
class.Bacteroidia.id.912	rs73846128	3	G	A	-0.064852276	0.013349001	8.75939E-07	18334	0.001285692	23.60222274
class.Bacteroidia.id.912	rs73975615	17	A	G	-0.207017892	0.044263343	1.21616E-06	3350	0.006487181	21.87395677
class.Bacteroidia.id.912	rs111845179	14	C	T	0.102647804	0.021384281	9.23988E-07	16393	0.001403594	23.04146084
class.Bacteroidia.id.912	rs2032750	2	C	T	-0.050839276	0.010680731	1.91509E-06	17853	0.001267461	22.65670539
class.Bacteroidia.id.912	rs7546249	1	A	T	-0.056711256	0.01183859	1.54814E-06	18333	0.001250149	22.9476706
class.Bacteroidia.id.912	rs929878	16	T	C	-0.054853206	0.012152954	4.73377E-06	18334	0.001109943	20.37231184
class.Bacteroidia.id.912	rs13291169	9	G	C	0.068967579	0.014876501	3.74579E-06	17513	0.001225731	21.49257077
class.Bacteroidia.id.912	rs17343978	22	C	A	-0.055205684	0.012027767	8.35928E-06	18334	0.001147735	21.06675116
class.Bacteroidia.id.912	rs72706335	1	C	T	-0.222409754	0.049345816	7.65519E-06	3819	0.005291189	20.31453977
class.Bacteroidia.id.912	rs11146701	10	G	A	0.047359582	0.010540996	7.08228E-06	18220	0.001106683	20.18610194
class.Bacteroidia.id.912	rs62531359	8	G	T	0.065572807	0.014989058	9.08665E-06	18320	0.001043566	19.13810288
class.Bacteroidia.id.912	rs4146051	2	G	A	-0.107377003	0.024566124	8.76426E-06	11372	0.001677195	19.10509891
class.Bacteroidia.id.912	rs79585701	8	C	A	0.064682288	0.01496282	9.98589E-06	18334	0.001018226	18.68718223
class.Bacteroidia.id.912	rs4916508	3	A	G	-0.046672941	0.010531247	8.47116E-06	18324	0.001070743	19.64132232
class.Bacteroidia.id.912	rs62575403	9	T	C	0.140054866	0.031112824	7.06129E-06	9668	0.002091566	20.26364366
class.Betaproteobacteria.id.2867	rs4033856	4	T	C	0.083242265	0.016723677	5.17166E-07	15945	0.001551406	24.77560208
class.Betaproteobacteria.id.2867	rs2367850	3	C	G	0.064005972	0.013037436	9.11762E-07	16717	0.001439702	24.10219791
class.Betaproteobacteria.id.2867	rs12467854	2	C	G	0.06413625	0.013176625	1.09863E-06	17038	0.001388598	23.69183773
class.Betaproteobacteria.id.2867	rs6087811	20	G	T	-0.097817738	0.019852201	7.44305E-07	16184	0.001497894	24.27827854
class.Betaproteobacteria.id.2867	rs62191117	2	G	A	0.064104357	0.013227087	1.16555E-06	17029	0.001377396	23.4880358
class.Betaproteobacteria.id.2867	rs62395635	5	C	T	0.109740893	0.02360002	2.94091E-06	15108	0.001429171	21.62281474
class.Betaproteobacteria.id.2867	rs2613606	7	T	C	-0.051310589	0.010909103	2.19817E-06	17146	0.001288585	22.12258738
class.Betaproteobacteria.id.2867	rs1928341	1	G	A	0.052558097	0.011040784	2.01529E-06	17150	0.001319599	22.66101828
class.Betaproteobacteria.id.2867	rs72747231	9	G	C	-0.137280671	0.029033768	2.05511E-06	11344	0.001966938	22.35692037
class.Betaproteobacteria.id.2867	rs11128180	3	G	A	0.059294412	0.012876923	3.67144E-06	17151	0.001234745	21.20329421
class.Betaproteobacteria.id.2867	rs2321387	13	A	G	-0.049459619	0.010942399	5.80036E-06	17149	0.001189928	20.43038108
class.Betaproteobacteria.id.2867	rs7638039	3	C	T	0.057694501	0.012653895	5.38696E-06	17132	0.001211953	20.78836748
class.Betaproteobacteria.id.2867	rs1511453	4	G	A	0.092349246	0.019915827	4.75765E-06	17085	0.001256923	21.50156164
class.Betaproteobacteria.id.2867	rs9964679	18	G	A	0.052911029	0.011486591	4.8458E-06	17151	0.001235616	21.21827583
class.Betaproteobacteria.id.2867	rs56386628	8	T	C	-0.062225169	0.013568762	5.86955E-06	17147	0.001224986	21.03060282
class.Betaproteobacteria.id.2867	rs320161	9	G	A	0.057228557	0.012619964	7.33242E-06	17035	0.001205711	20.56408218
class.Betaproteobacteria.id.2867	rs75242906	15	T	C	-0.121083253	0.028074987	9.27278E-06	10803	0.001718848	18.60068921
class.Clostridia.id.1859	rs6815608	4	C	T	0.103803179	0.021088299	4.01857E-07	16731	0.001446065	24.22915096
class.Clostridia.id.1859	rs112334273	21	A	G	0.064100812	0.012740366	3.81407E-07	18217	0.001387661	25.31414272
class.Clostridia.id.1859	rs6814436	4	C	T	0.073999481	0.015081514	9.65098E-07	18336	0.001311273	24.07506374
class.Clostridia.id.1859	rs72915163	18	C	T	-0.058102511	0.012055746	1.34295E-06	18317	0.001266476	23.22745467
class.Clostridia.id.1859	rs2273429	14	G	A	-0.072403727	0.015310495	4.52136E-06	18337	0.001218108	22.36368726
class.Clostridia.id.1859	rs10774377	12	G	A	0.052659008	0.011385834	3.24401E-06	18330	0.001165592	21.39024009
class.Clostridia.id.1859	rs13179700	5	C	T	0.051193667	0.010956865	3.37083E-06	18338	0.001189025	21.83030606
class.Clostridia.id.1859	rs6934062	6	C	G	0.052440576	0.01161154	6.53083E-06	17859	0.001140781	20.39648304
class.Clostridia.id.1859	rs1842454	5	A	G	-0.054720208	0.012733828	8.72023E-06	18340	0.001005868	18.46619808
class.Clostridia.id.1859	rs10209007	2	C	G	0.079417263	0.018242714	9.89343E-06	17812	0.001062861	18.95182033
class.Clostridia.id.1859	rs3792064	2	A	G	0.07956506	0.018252101	9.98416E-06	17812	0.00106572	19.00286373
class.Clostridia.id.1859	rs76767978	3	A	G	0.069888247	0.015631881	8.398E-06	18329	0.001089366	19.98875836
class.Clostridia.id.1859	rs13105690	4	C	T	-0.052909681	0.011817976	8.7779E-06	18340	0.001091718	20.04398451
class.Clostridia.id.1859	rs76860606	6	G	C	0.094703578	0.022511672	7.53167E-06	17579	0.001005741	17.69771836
class.Clostridia.id.1859	rs72738886	5	C	T	0.086616952	0.019039264	8.24241E-06	16934	0.001220718	20.69690802
class.Clostridia.id.1859	rs7269302	20	T	A	-0.096398079	0.021200675	9.04045E-06	17405	0.001186445	20.67461238
class.Clostridia.id.1859	rs6797343	3	T	G	0.059227896	0.013455633	9.35647E-06	18340	0.001055325	19.37511607
class.Clostridia.id.1859	rs13421739	2	G	C	0.088847155	0.017965467	9.54199E-06	18340	0.001331778	24.45738648
class.Coriobacteriia.id.809	rs719099	10	G	A	0.077840173	0.01558151	5.43317E-07	17687	0.001409035	24.95677559
class.Coriobacteriia.id.809	rs240104	1	C	T	-0.060342897	0.012684911	1.51603E-06	17577	0.001285801	22.62962954
class.Coriobacteriia.id.809	rs11250875	10	C	T	0.060749978	0.013093887	4.82844E-06	17690	0.001215344	21.5255891
class.Coriobacteriia.id.809	rs34739816	17	T	G	0.096501674	0.020766582	3.88358E-06	16593	0.00129972	21.59432574

class.Coriobacteriia.id.809	rs76779974	5	G	C	0.0774888	0.017187698	5.63091E-06	16419	0.001236398	20.32555641
class.Coriobacteriia.id.809	rs80046645	5	G	C	0.255839805	0.056199532	4.65689E-06	3204	0.006426556	20.72386883
class.Coriobacteriia.id.809	rs45480394	19	G	T	-0.050426168	0.011330684	9.71448E-06	17413	0.001136141	19.80613186
class.Coriobacteriia.id.809	rs1816223	12	G	A	-0.058626474	0.012900576	4.84114E-06	17688	0.001166229	20.65234419
class.Coriobacteriia.id.809	rs67561917	20	G	A	-0.071436723	0.015411343	5.39129E-06	17333	0.001238085	21.48632118
class.Coriobacteriia.id.809	rs3025411	9	G	A	0.092654285	0.020946781	8.26912E-06	15671	0.001246975	19.56574696
class.Coriobacteriia.id.809	rs11656361	17	C	A	0.077300587	0.01755297	8.02017E-06	16665	0.001162396	19.3938654
class.Coriobacteriia.id.809	rs1397793	5	A	G	-0.049885455	0.011244635	9.77381E-06	17527	0.001121662	19.68145117
class.Coriobacteriia.id.809	rs11073596	15	G	T	0.051021228	0.011433922	8.14449E-06	17527	0.001134777	19.91183886
class.Coriobacteriia.id.809	rs2442778	3	A	G	-0.116408329	0.025854513	9.02703E-06	13472	0.001502485	20.27194292
class.Coriobacteriia.id.809	rs7540303	1	T	C	0.048322746	0.010906179	9.62148E-06	17691	0.00110847	19.63170353
class.Coriobacteriia.id.809	rs62448869	7	A	T	-0.048605943	0.010898242	7.87276E-06	17527	0.001133615	19.89142221
class.Coriobacteriia.id.809	rs13307134	7	T	C	0.056568602	0.012626474	7.79931E-06	17527	0.001143884	20.0718175
class.Coriobacteriia.id.809	rs12974142	19	A	G	0.078979608	0.017720479	8.51232E-06	17413	0.001139489	19.86456328
class.Coriobacteriia.id.809	rs8010111	14	A	G	-0.10337688	0.022928631	6.89624E-06	15996	0.001269193	20.32780913
class.Deltaproteobacteria.id.3087	rs6058181	20	T	C	0.082571565	0.016595819	3.39554E-07	15100	0.001636723	24.75504021
class.Deltaproteobacteria.id.3087	rs4506934	17	T	C	-0.093651362	0.020120034	3.58887E-06	15061	0.001436457	21.66560229
class.Deltaproteobacteria.id.3087	rs17791387	9	G	A	-0.073588121	0.015424623	1.60223E-06	15071	0.001507957	22.76073606
class.Deltaproteobacteria.id.3087	rs2838334	21	A	G	0.056172159	0.012412391	5.44551E-06	15168	0.001348395	20.48006535
class.Deltaproteobacteria.id.3087	rs9928243	16	A	C	-0.053845737	0.011771796	5.02081E-06	15167	0.001377586	20.92267314
class.Deltaproteobacteria.id.3087	rs11599763	10	C	T	-0.054421883	0.011739659	3.94123E-06	15168	0.001414793	21.48998835
class.Deltaproteobacteria.id.3087	rs112381107	9	T	C	0.206594184	0.045690815	4.63054E-06	4457	0.004566129	20.44458854
class.Deltaproteobacteria.id.3087	rs17084793	18	A	G	-0.071076166	0.015953009	5.68634E-06	15164	0.001307317	19.85010218
class.Deltaproteobacteria.id.3087	rs2692012	1	G	A	0.1103349	0.025333574	3.14186E-06	13104	0.001445442	18.96849461
class.Deltaproteobacteria.id.3087	rs1035691	11	G	A	-0.055175977	0.012160175	9.65172E-06	15168	0.001355511	20.58829673
class.Deltaproteobacteria.id.3087	rs62020470	15	G	A	-0.058541133	0.012935848	4.85084E-06	15168	0.001348398	20.4801165
class.Deltaproteobacteria.id.3087	rs7164160	15	T	A	-0.058325007	0.012935769	5.27857E-06	15168	0.00133849	20.32942489
class.Deltaproteobacteria.id.3087	rs16851319	1	C	G	-0.07055156	0.015084942	5.67888E-06	15154	0.001441359	21.87388569
class.Deltaproteobacteria.id.3087	rs3935584	2	T	C	-0.052348451	0.011566041	7.50121E-06	15166	0.001348904	20.48510481
class.Deltaproteobacteria.id.3087	rs55744759	8	G	A	-0.077969339	0.017073844	7.3137E-06	15168	0.001372967	20.85379513
class.Deltaproteobacteria.id.3087	rs72647048	8	C	T	-0.07781647	0.017073835	7.31698E-06	15168	0.001367597	20.7721227
class.Erysipelotrichia.id.2147	rs62504403	8	T	C	0.068115892	0.012788339	1.12265E-07	16981	0.00166794	28.37060806
class.Erysipelotrichia.id.2147	rs7234058	18	C	T	-0.094575892	0.019409083	9.12341E-07	16874	0.001405148	23.74383587
class.Erysipelotrichia.id.2147	rs17530232	13	G	A	0.103049982	0.022464754	2.79123E-06	16622	0.001264328	21.04226554
class.Erysipelotrichia.id.2147	rs2300774	3	A	G	0.052415201	0.010678379	8.95466E-07	18216	0.001320921	24.09371743
class.Erysipelotrichia.id.2147	rs35161940	17	C	T	-0.080604056	0.01676409	1.8451E-06	18213	0.001267714	23.11819028
class.Erysipelotrichia.id.2147	rs10781552	10	T	C	-0.055221236	0.011607425	2.33114E-06	17789	0.001270682	22.63291274
class.Erysipelotrichia.id.2147	rs8003149	14	T	C	0.053879756	0.01168879	4.08475E-06	18213	0.001165264	21.24770908
class.Erysipelotrichia.id.2147	rs4078432	14	T	C	-0.060892934	0.013376363	4.23104E-06	17789	0.001163591	20.72324212
class.Erysipelotrichia.id.2147	rs1074800	5	G	A	0.049243827	0.010887044	6.14583E-06	17789	0.00114877	20.45896385
class.Erysipelotrichia.id.2147	rs56970041	14	G	T	0.072414447	0.016447064	5.40344E-06	18217	0.001063004	19.38535258
class.Erysipelotrichia.id.2147	rs7826267	8	G	T	-0.083907147	0.019913089	9.28449E-06	17586	0.001008592	17.75499888
class.Erysipelotrichia.id.2147	rs1884466	1	T	C	-0.047536345	0.01069386	9.52701E-06	18205	0.001084228	19.75979992
class.Erysipelotrichia.id.2147	rs290833	1	G	T	-0.049737371	0.011137647	8.029E-06	17789	0.0011198	19.94246102
class.Gammaproteobacteria.id.3303	rs6706173	2	C	A	0.074250965	0.014639234	1.99495E-07	16020	0.001603275	25.72571823
class.Gammaproteobacteria.id.3303	rs11181912	12	A	G	-0.057915255	0.011879636	9.94762E-07	16017	0.00148168	23.76728982
class.Gammaproteobacteria.id.3303	rs79795896	18	G	A	-0.159209919	0.03513731	7.92404E-06	7577	0.002702283	20.53067504
class.Gammaproteobacteria.id.3303	rs2234691	10	C	T	0.076290532	0.017196192	8.39264E-06	16011	0.001227792	19.68233837
class.Gammaproteobacteria.id.3303	rs6469506	8	A	T	0.054142416	0.011633307	3.28982E-06	16023	0.001350014	21.66052066
class.Gammaproteobacteria.id.3303	rs12404135	1	G	A	-0.078884268	0.017241506	8.89485E-06	15231	0.001372478	20.93294968
class.Gammaproteobacteria.id.3303	rs9973122	18	A	T	0.074061387	0.016259636	6.77007E-06	15650	0.001323952	20.74732312
class.Gammaproteobacteria.id.3303	rs9494710	6	T	C	-0.054967253	0.012092397	4.54607E-06	15650	0.001318548	20.66252056
class.Gammaproteobacteria.id.3303	rs75101789	3	T	C	0.072901201	0.016300252	8.78789E-06	16024	0.001246717	20.00233535
class.Lentisphaeria.id.2250	rs2825714	21	G	A	-0.137410136	0.02892458	1.72211E-06	4734	0.00474471	22.56853747
class.Lentisphaeria.id.2250	rs17114848	15	A	G	0.152376929	0.032433167	4.05865E-06	4734	0.004640996	22.07291674
class.Lentisphaeria.id.2250	rs77599476	20	G	A	0.230291775	0.048016811	1.86133E-06	3722	0.006142118	23.00224553
class.Lentisphaeria.id.2250	rs11770843	7	T	C	0.109430892	0.023487897	1.90729E-06	4705	0.004592329	21.70658996
class.Lentisphaeria.id.2250	rs72640280	1	G	A	0.220206945	0.048619648	5.18035E-06	3816	0.005346895	20.51343605
class.Lentisphaeria.id.2250	rs62570196	9	T	C	-0.216350467	0.043986577	1.07926E-06	3712	0.006475095	24.19219835
class.Lentisphaeria.id.2250	rs11764871	7	T	G	0.105796117	0.023346418	6.38503E-06	4725	0.004327268	20.5352007
class.Lentisphaeria.id.2250	rs2731834	5	G	C	0.109437835	0.023692997	4.24358E-06	4733	0.004487508	21.3351157
class.Lentisphaeria.id.2250	rs2031282	13	G	A	0.122368503	0.027032933	4.3826E-06	4734	0.004309719	20.4905185
class.Lentisphaeria.id.2250	rs2546105	5	T	A	0.106187252	0.023469029	6.52724E-06	4734	0.004305784	20.47172918
class.Lentisphaeria.id.2250	rs1002941	15	A	G	0.105025041	0.023348442	8.14836E-06	4734	0.00425588	20.2334482
class.Lentisphaeria.id.2250	rs73113483	3	A	T	-0.131216985	0.0288713	8.66341E-06	4718	0.004359054	20.65605772
class.Melainabacteria.id.1589	rs9864379	3	C	T	-0.159734555	0.029255297	5.3606E-08	5777	0.005133942	29.81183555
class.Melainabacteria.id.1589	rs11150282	16	C	T	0.09894875	0.019697164	6.02892E-07	5873	0.004278496	25.2355769
class.Melainabacteria.id.1589	rs16851659	1	C	G	-0.089610337	0.01864323	1.27487E-06	5884	0.003911104	23.10329317
class.Melainabacteria.id.1589	rs73074665	7	T	A	0.166215674	0.035572757	2.86608E-06	5758	0.003777406	21.83277313
class.Melainabacteria.id.1589	rs4129395	9	A	G	0.08961869	0.01851058	1.48056E-06	5887	0.003965859	23.43997431
class.Melainabacteria.id.1589	rs79790072	15	C	T	0.226721783	0.048784054	3.29009E-06	4107	0.005231522	21.59885485
class.Melainabacteria.id.1589	rs10738747	9	A	G	0.081462422	0.018440929	9.95804E-06	5884	0.00330551	19.51412366
class.Melainabacteria.id.1589	rs1221147	9	A	T	0.123968094	0.027895845	7.10737E-06	5768	0.003412174	19.74880427
class.Melainabacteria.id.1589	rs113884518	9	C	T	-0.20535201	0.045498234	8.0567E-06	4442	0.004565025	20.37083555
class.Melainabacteria.id.1589	rs789069	18	C	A	-0.103539228	0.023428675	6.84635E-06	5879	0.003311087	19.53054559
class.Melainabacteria.id.1589	rs367480	11	A	G	-0.08375796	0.018579584	8.19925E-06	5887	0.003440246	20.32264501
class.Melainabacteria.id.1589	rs28678345	17	C	T	0.214848361	0.047087794	6.69088E-06	4323	0.00479265	20.81840281
class.Melainabacteria.id.1589	rs10148250	14	A	G	0.086192825	0.019338072	8.67135E-06	5453	0.003629953	19.86624859
class.Methanobacteria.id.119	rs10202904	2	G	T	-0.121753579	0.023535682	3.0143E-07	3695	0.007190538	26.76146558
class.Methanobacteria.id.119	rs73457410	13	G	A	0.215339755	0.04366952	1.40855E-06	3498	0.006903404	24.3159698
class.Methanobacteria.id.119	rs76029318	13	C	T	0.214769455	0.0446468	1.58427E-06	3497	0.006573617	23.14005323
class.Methanobacteria.id.119	rs6776814	3	C	T	-0.199565931	0.04118249	1.63062E-06	3385	0.006889481	23.48267699
class.Methanobacteria.id.119	rs894996	4	A	C	0.216999069	0.044907554	1.87557E-06	3582	0.006476339	23.34946566
class.Methanobacteria.id.119	rs73031978	3	G	C	-0.203186321	0.042161972	1.90189E-06	3340	0.006905438	23.22453869
class.Methanobacteria.id.119	rs75208022	12	T	C	-0.227244713	0.048763001	5.92103E-06	3203	0.006734651	21.71734467
class.Methanobacteria.id.119	rs12825290	12	G	C	-0.216781075	0.049349641	6.07567E-06	3144	0.006100071	19.29633077
class.Methanobacteria.id.119	rs56131665	5	A	G	0.178661188	0.039297982	6.17992E-06	3572	0.005753111	20.66902243
class.Methanobacteria.id.119	rs11018665	11	T	A	0.111465287	0.025051554	6.51864E-06	3698	0.005325057	19.79748181
class.Methanobacteria.id.119	rs4257531	3	A	G	0.164498365	0.03649593	7.44344E-06	3162	0.006383973	20.31581768
class.Methanobacteria.id.119	rs6508769	19	C	T	0.15351913	0.034451866	8.22529E-06	3319	0.005947052	19.85635359
class.Methanobacteria.id.119	rs73068003	7	T	G	-0.158140381	0.035180317	8.44583E-06	3692	0.005443196	20.20626583
class.Methanobacteria.id.119	rs10424197	19	A	G	-0.111274175	0.024751678	9.27887E-06	3698	0.005435575	20.2106127
class.Mollicutes.id.3920	rs74603314	14	C	T	0.221639279	0.046291765	1.55689E-06	4499	0.005069472	22.92376615
class.Mollicutes.id.3920	rs10108398	8	A	G	0.076914215	0.015395275	1.09E-06	10932	0.002277971	24.95964108
class.Mollicutes.id.3920	rs11890098	2	G	A	0.074438031	0.015338858	9.56571E-07	11392	0.002063036	23.55069342
class.Mollicutes.id.3920	rs72901605	11	C	T	-0.084185237	0.01781192	3.2579E-06	10526	0.002117709	22.33831558
class.Mollicutes.id.3920	rs3768491	1	G	A	-0.068105197	0.01490611	4.23352E-06	11002	0.001893812	20.87525853
class.Mollicutes.id.3920	rs638542	1	A	G	-0.068036144	0.014955512	4.48578E-06	10999	0.00187805	20.69554296
class.Mollicutes.id.3920	rs17214486	14	A	C	0.060989958	0.013562263	6.60866E-06	11387	0.001772852	20.22331672
class.Mollicutes.id.3920	rs12566890	1	G	T	-0.101147132	0.023097845	3.65095E-06	11371	0.00168358	19.17627429
class.Mollicutes.id.3920	rs78169027	11	G	A	-0.108282943	0.023728915	5.8761E-06	10542	0.001971443	20.82400904
class.Mollicutes.id.3920	rs4885016	13	C	T	-0.081960567	0.018162779	7.26986E-06	11388	0.001784933	20.36316752
class.Mollicutes.id.3920	rs28537087	15	A	G	0.08208727	0.018831168	8.07399E-06	11382	0.001666687	19.00190425
class.Mollicutes.id.3920	rs2464826	7	C	A	0.094423946	0.021180616	8.39505E-06	10998	0.001803804	19.87408379
class.Mollicutes.id.3920	rs6043847	20	C	T	-0.11493717	0.024860602	4.55476E-06	11130	0.001916767	21.37458673
class.Negativicutes.id.2164	rs1643968	5	C	T	-0.056528439	0.011229806	4.14596E-07	18170	0.001392609	25.33899942
class.Negativicutes.id.2164	rs60274479	16	C	T	-0.065962286	0.013413689	1.1623E-06	17610	0.001371323	24.18215135
class.Negativicutes.id.2164	rs13086907	3	A	G	0.062531199	0.013173503	1.95488E-06	18163	0.001238982	22.53154506
class.Negativicutes.id.2164	rs73232831	4	A	G	-0.151828859	0.03149318	1.87452E-06	9382	0.002471187	23.2421138
class.Negativicutes.id.2164	rs71405394	15	A	G	-0.114177822	0.02404996	2.16631E-06	13861	0.001623434	22.53900806
class.Negativicutes.id.2164	rs61249479	9	C	A	0.077707533	0.01686282	2.95471E-06	18165	0.001167678	21.23566348
class.Negativicutes.id.2164	rs1447205	8	C	G	-0.050205329	0.010838203	2.93324E-06	18165	0.001179878	21.45779268
class.Negativicutes.id.2164	rs4722181	7	G	T	0.050130576	0.010579796	2.00112E-06	18163	0.001234601	22.45178116
class.Negativicutes.id.2164	rs4463806	10	C	T	-0.05439664	0.012936525	7.80662E-06	18160	0.000972681	17.68108324
class.Negativicutes.id.2164	rs9423647	10	A	G	0.047817029	0.010528201	6.06114E-06	18164	0.001134364	20.62798538
class.Negativicutes.id.2164	rs2834062	21	G	A	0.0489057	0.010884164	8.4392E-06	18171	0.001109859	20.18965087
class.Negativicutes.id.2164	rs1649999	10	G	A	0.074898861	0.01664577	7.5812E-06	18036	0.001121283	20.24615865
class.Negativicutes.id.2164	rs1135612	7	A	G	0.052978507	0.01191767	9.26307E-06	18164	0.001086758	19.76135468
class.Verrucomicrobiae.id.4029	rs11729256	4	C	T	0.074979802	0.015017677	6.7309E-07	11861	0.002097249	24.92774882
class.Verrucomicrobiae.id.4029	rs4936098	11	G	A	0.064884253	0.013592809	1.12221E-06	11858	0.001917852	22.78558435
class.Verrucomicrobiae.id.4029	rs9349825	6	G	A	-0.070403566	0.014712789	2.53687E-06	11858	0.001927303	22.89809198
class.Verrucomicrobiae.id.4029	rs11252894	10	C	A	0.077093323	0.016605195	2.93907E-06	11864	0.001813536	21.5548784
class.Verrucomicrobiae.id.4029	rs74542928	4	C	T	0.112163707	0.023642195	1.63222E-06	11216	0.002002721	22.50759662
class.Verrucomicrobiae.id.4029	rs3995795	10	T	C	0.064127531	0.014099468	5.18557E-06	11690	0.001766449	20.68633587
class.Verrucomicrobiae.id.4029	rs2602429	16	T	C	0.074684437	0.015619438	2.58297E-06	11690	0.00195194	22.86280045
class.Verrucomicrobiae.id.4029	rs4242783	10	A	G	0.068929219	0.014769297	2.63612E-06	11690	0.001859791	21.78146308
class.Verrucomicrobiae.id.4029	rs11184341	1	C	G	0.065534193	0.014223078	4.13405E-06	11864	0.001786246	21.22993935
class.Verrucomicrobiae.id.4029	rs61779207	1	A	G	-0.075830853	0.016776157	6.72285E-06	11591	0.001759629	20.43181414
class.Verrucomicrobiae.id.4029	rs117107102	18	G	A	0.20468288	0.043157251	2.91821E-06	4975	0.00450094	22.49342052

class.Verrucomicrobiae.id.4029	rs12908520	15	A	G	0.061892558	0.013094626	2.17163E-06	11863	0.001879659	22.34038678
class.Verrucomicrobiae.id.4029	rs111862613	12	C	T	0.090698957	0.019674596	3.73846E-06	11621	0.001825391	21.25166394
class.Verrucomicrobiae.id.4029	rs941682	20	A	G	-0.063143598	0.014376741	9.61003E-06	11809	0.001630857	19.29024644
family.Acidaminococcaceae.id.2166	rs6923842	6	C	T	-0.079610621	0.016919247	2.21303E-06	11904	0.001856436	22.14011988
family.Acidaminococcaceae.id.2166	rs6589457	11	G	A	0.165910976	0.034951601	2.31508E-06	7386	0.003041472	22.532848
family.Acidaminococcaceae.id.2166	rs2933324	9	G	A	-0.066255706	0.014025652	2.23676E-06	12525	0.001778483	22.31518286
family.Acidaminococcaceae.id.2166	rs262812	6	C	T	-0.065674997	0.014225656	3.24906E-06	12099	0.001758497	21.31353672
family.Acidaminococcaceae.id.2166	rs74540770	3	A	G	-0.108969098	0.024353266	7.08804E-06	10606	0.001884177	20.02130055
family.Acidaminococcaceae.id.2166	rs6427992	1	C	G	-0.059585662	0.012960033	4.24324E-06	12527	0.001684581	21.13836108
family.Acidaminococcaceae.id.2166	rs45497800	20	C	T	-0.117862982	0.025735121	5.86428E-06	11316	0.001850143	20.97502145
family.Acidaminococcaceae.id.2166	rs78702810	3	C	T	-0.143755204	0.032324372	9.16468E-06	7992	0.002468642	19.77820914
family.Actinomycetaceae.id.421	rs35011108	6	G	A	0.24182648	0.050379638	1.8267E-06	3630	0.006307294	23.04080338
family.Actinomycetaceae.id.421	rs2889192	9	T	G	0.088753744	0.019500862	3.64417E-06	7710	0.002679453	20.71408472
family.Actinomycetaceae.id.421	rs34583783	6	T	G	0.123755794	0.026426988	5.47971E-06	7710	0.002836272	21.92985464
family.Actinomycetaceae.id.421	rs58484246	15	C	T	0.0761407	0.016900672	6.12428E-06	7710	0.002625606	20.29671684
family.Actinomycetaceae.id.421	rs4073240	6	A	G	0.074754607	0.016468358	6.05333E-06	7710	0.002665397	20.60512857
family.Alcaligenaceae.id.2875	rs2321387	13	A	G	-0.056892183	0.011048955	2.46022E-07	16808	0.001574933	26.51323826
family.Alcaligenaceae.id.2875	rs62191117	2	G	A	0.068430199	0.013363598	2.75943E-07	16688	0.001568782	26.22097392
family.Alcaligenaceae.id.2875	rs9537886	13	C	A	-0.057088849	0.011068258	2.35108E-07	16807	0.0015804	26.60381954
family.Alcaligenaceae.id.2875	rs12467854	2	C	G	0.065731844	0.013311112	7.63053E-07	16697	0.001458313	24.38501118
family.Alcaligenaceae.id.2875	rs4033856	4	T	C	0.081833964	0.016893105	1.02902E-06	15623	0.001499795	23.46649336
family.Alcaligenaceae.id.2875	rs67737557	20	G	C	0.073840948	0.015167785	1.03742E-06	15876	0.0014906	23.7000999
family.Alcaligenaceae.id.2875	rs17000015	4	T	A	-0.060925748	0.012536315	1.12719E-06	16377	0.00144013	23.61902204
family.Alcaligenaceae.id.2875	rs2367850	3	C	G	0.062910409	0.013171198	1.84631E-06	16377	0.001391091	22.81363433
family.Alcaligenaceae.id.2875	rs7638039	3	C	T	0.060333529	0.012777892	2.70479E-06	16791	0.001326008	22.29456474
family.Alcaligenaceae.id.2875	rs6087811	20	G	T	-0.092704374	0.020045669	3.36838E-06	15868	0.001346022	21.38746554
family.Alcaligenaceae.id.2875	rs62395635	5	C	T	0.110582886	0.023851434	3.34696E-06	14802	0.001450094	21.49546464
family.Alcaligenaceae.id.2875	rs112159068	6	T	A	0.131483982	0.028108868	3.56484E-06	11656	0.001873678	21.88058755
family.Alcaligenaceae.id.2875	rs11128180	3	G	A	0.058649835	0.013003734	6.24095E-06	16810	0.001208661	20.34217587
family.Alcaligenaceae.id.2875	rs6969323	7	C	A	-0.059356404	0.012913653	3.88581E-06	16810	0.001255231	21.12695659
family.Alcaligenaceae.id.2875	rs7302582	12	C	G	-0.106816521	0.023298022	3.33554E-06	15113	0.001388943	21.02029214
family.Alcaligenaceae.id.2875	rs1153990	5	G	A	-0.058726971	0.012830567	5.96787E-06	16808	0.001244877	20.94997472
family.Alcaligenaceae.id.2875	rs9964679	18	G	A	0.053705118	0.011600815	4.47389E-06	16810	0.001273307	21.43158759
family.Alcaligenaceae.id.2875	rs112135816	9	G	T	-0.077505169	0.017155357	5.27662E-06	15545	0.001311297	20.41088365
family.Alcaligenaceae.id.2875	rs147968	16	T	C	0.048947517	0.011032087	9.12701E-06	16810	0.001169688	19.68547889
family.Alcaligenaceae.id.2875	rs2613606	7	T	C	-0.048629411	0.011021758	8.89205E-06	16805	0.001157057	19.46687481
family.Alcaligenaceae.id.2875	rs74776516	11	G	T	-0.09440518	0.021328653	6.85049E-06	14502	0.00134912	19.59136997
family.Alcaligenaceae.id.2875	rs28480294	15	C	T	-0.051696664	0.011550326	6.606E-06	16377	0.001221719	20.03256382
family.Bacteroidaceae.id.917	rs6795673	3	T	C	0.053856525	0.010525127	3.37909E-07	18301	0.001428651	26.18315417
family.Bacteroidaceae.id.917	rs28757219	6	A	T	0.081839827	0.017031866	1.2942E-06	14833	0.001554177	23.08898982
family.Bacteroidaceae.id.917	rs9507307	13	T	C	0.060445621	0.012912793	2.1271E-06	18184	0.001203585	21.91236741
family.Bacteroidaceae.id.917	rs66474973	20	T	G	0.081251539	0.016447899	6.80966E-07	17405	0.001400102	24.40294961
family.Bacteroidaceae.id.917	rs11585893	1	G	A	-0.074074561	0.014763325	1.79511E-06	17821	0.001410668	25.17501972
family.Bacteroidaceae.id.917	rs495004	9	G	C	-0.060747491	0.012986536	3.42092E-06	17707	0.001234209	21.88114365
family.Bacteroidaceae.id.917	rs17619981	19	G	T	0.088097761	0.018700207	2.68683E-06	15801	0.001402629	22.19406888
family.Bacteroidaceae.id.917	rs2023437	14	C	T	-0.07823245	0.016763399	5.01824E-06	17484	0.001244137	21.77958883
family.Bacteroidaceae.id.917	rs66710942	3	T	C	0.048803777	0.010741209	5.86259E-06	17821	0.001157086	20.64431778
family.Bacteroidaceae.id.917	rs13207588	6	G	A	-0.059204982	0.013119468	7.48504E-06	17707	0.001148788	20.36498267
family.Bacteroidaceae.id.917	rs2366421	3	A	T	-0.05281527	0.011712437	7.64945E-06	18299	0.001109979	20.33407866
family.Bacteroidaceae.id.917	rs1340391	1	C	T	-0.059200382	0.013224416	6.7301E-06	18302	0.00109376	20.03992112
family.BacteroidalesS24.7group.id.	rs738193	22	C	T	0.084732776	0.016585588	3.82163E-07	7778	0.0033444	26.10003585
family.BacteroidalesS24.7group.id.	rs941000	7	T	C	0.085026574	0.016338237	3.15557E-07	7778	0.003469936	27.08313674
family.BacteroidalesS24.7group.id.	rs689695	15	A	C	0.081457787	0.016705675	1.28039E-06	7954	0.002980268	23.77591135
family.BacteroidalesS24.7group.id.	rs17043785	2	C	T	-0.176186835	0.034713295	5.11837E-07	5208	0.004921997	25.76055611
family.BacteroidalesS24.7group.id.	rs10872669	6	G	A	-0.123070536	0.027566518	9.4901E-06	7368	0.002697873	19.93170087
family.BacteroidalesS24.7group.id.	rs12748533	1	T	G	-0.082110804	0.017270056	2.59019E-06	7948	0.002836102	22.60544699
family.BacteroidalesS24.7group.id.	rs6831034	4	A	T	-0.095654602	0.020522806	6.10396E-06	7576	0.002859267	21.72392301
family.BacteroidalesS24.7group.id.	rs61508842	3	C	T	0.122574311	0.027238851	7.82782E-06	7576	0.002665766	20.2498283
family.BacteroidalesS24.7group.id.	rs78609301	6	G	A	-0.086706514	0.019570857	7.09297E-06	7953	0.002461967	19.6283482
family.BacteroidalesS24.7group.id.	rs11135366	5	G	C	0.084214168	0.01837388	8.77822E-06	7953	0.002634462	21.00721834
family.BacteroidalesS24.7group.id.	rs7217209	17	T	C	0.084301877	0.018730791	8.42937E-06	7745	0.002608594	20.25639925
family.Bifidobacteriaceae.id.433	rs182549	2	T	C	0.117069879	0.01267034	5.942E-20	14915	0.005691304	85.37167272
family.Bifidobacteriaceae.id.433	rs7570971	2	C	A	0.112507848	0.012517823	2.5317E-19	14795	0.005430356	80.78078113
family.Bifidobacteriaceae.id.433	rs1530559	2	A	G	0.073829493	0.01197211	5.23222E-10	14795	0.002563826	38.02930229
family.Bifidobacteriaceae.id.433	rs7322849	13	C	T	0.110675689	0.020099551	1.74032E-08	14915	0.002028741	30.32017906
family.Bifidobacteriaceae.id.433	rs76671854	5	G	C	-0.089298109	0.018317684	1.03632E-06	13923	0.001704001	23.76530551
family.Bifidobacteriaceae.id.433	rs56108664	5	C	T	0.074567905	0.015721335	1.36916E-06	14041	0.001599675	22.49702148
family.Bifidobacteriaceae.id.433	rs4957061	5	C	T	0.056987252	0.011690731	1.1517E-06	14795	0.001603468	23.761404
family.Bifidobacteriaceae.id.433	rs10841473	12	C	G	-0.061303123	0.012884445	2.28067E-06	14914	0.001515587	22.63777448
family.Bifidobacteriaceae.id.433	rs4567981	2	A	T	0.057776313	0.011738841	8.88054E-07	14795	0.001634646	24.22418712
family.Bifidobacteriaceae.id.433	rs13020688	2	A	G	0.058404469	0.012208136	1.57375E-06	14795	0.001544569	22.887243
family.Bifidobacteriaceae.id.433	rs677208	18	C	G	0.072858928	0.016482072	6.0011E-06	14915	0.001308429	19.54078289
family.Bifidobacteriaceae.id.433	rs11745923	5	T	G	0.057975926	0.012102145	2.49154E-06	14914	0.001536416	22.94936694
family.Bifidobacteriaceae.id.433	rs12446429	16	C	T	0.081009916	0.019073029	8.52886E-06	14008	0.001286181	18.04002059
family.Bifidobacteriaceae.id.433	rs11655079	17	C	T	-0.058614646	0.012995163	5.92468E-06	14912	0.001362451	20.34458447
family.Bifidobacteriaceae.id.433	rs540489	17	G	T	-0.06325184	0.013816951	5.36811E-06	14688	0.001424754	20.95665056
family.Bifidobacteriaceae.id.433	rs857444	6	T	C	0.05539651	0.012066937	3.82492E-06	14914	0.001411118	21.0751537
family.Bifidobacteriaceae.id.433	rs10857328	4	A	T	0.056200033	0.012639517	7.24135E-06	14915	0.001323773	19.7702523
family.Bifidobacteriaceae.id.433	rs10831953	11	A	G	0.053753749	0.012374596	9.94742E-06	14915	0.001263523	18.86928686
family.Bifidobacteriaceae.id.433	rs7174549	15	T	C	0.055172631	0.01246547	6.8678E-06	14915	0.001311707	19.58980198
family.Bifidobacteriaceae.id.433	rs73797465	5	G	T	-0.094261173	0.020842122	4.8495E-06	14795	0.001380597	20.45416703
family.Bifidobacteriaceae.id.433	rs10496759	2	C	G	0.063713288	0.014430511	8.79796E-06	14914	0.001305375	19.49380532
family.Bifidobacteriaceae.id.433	rs55888705	4	G	A	0.053672952	0.012058615	8.66208E-06	14914	0.001326617	19.81144194
family.Bifidobacteriaceae.id.433	rs6899771	6	G	A	-0.09143118	0.020260353	7.27995E-06	14150	0.001437188	20.36547848
family.Bifidobacteriaceae.id.433	rs62181700	2	A	G	-0.060422854	0.013060631	4.12459E-06	14911	0.001433324	21.40297508
family.Christensenellaceae.id.1866	rs62573205	9	A	G	-0.065480354	0.013350288	1.48853E-06	16838	0.001426692	24.05696281
family.Christensenellaceae.id.1866	rs870002	8	T	C	0.048895121	0.010981691	6.54323E-06	16847	0.001175329	19.8240613
family.Christensenellaceae.id.1866	rs12657403	5	G	A	0.078107959	0.017188664	5.59276E-06	16245	0.001269506	20.64934722
family.Christensenellaceae.id.1866	rs117186816	11	A	G	-0.206475619	0.043636232	6.51964E-06	4310	0.005167918	22.38943187
family.Christensenellaceae.id.1866	rs9608766	22	C	T	-0.068213442	0.01495997	3.33084E-06	16185	0.001282946	20.79114892
family.Christensenellaceae.id.1866	rs4076564	6	A	G	-0.188623195	0.039209045	8.86105E-06	3730	0.00616627	23.14289382
family.Christensenellaceae.id.1866	rs4805124	19	G	C	0.048650752	0.010906688	8.36272E-06	16847	0.001179664	19.89726417
family.Christensenellaceae.id.1866	rs72706624	9	T	G	0.088262607	0.019977489	6.3775E-06	14869	0.001340373	19.95675883
family.Christensenellaceae.id.1866	rs77867022	7	T	G	-0.160443345	0.037958856	8.81983E-06	4033	0.004410312	17.86558252
family.Christensenellaceae.id.1866	rs7211194	17	T	C	0.049209379	0.011091636	9.93998E-06	16436	0.001196158	19.68360057
family.Christensenellaceae.id.1866	rs892686	9	G	A	0.050610664	0.010965057	4.12367E-06	16847	0.001262964	21.30405613
family.Christensenellaceae.id.1866	rs12380890	9	G	A	-0.050397219	0.011104738	5.78132E-06	16846	0.00122115	20.59664614
family.Clostridiaceae1.id.1869	rs12186080	3	A	G	0.074823577	0.016245981	5.33957E-06	14244	0.001486986	21.21216612
family.Clostridiaceae1.id.1869	rs2795528	10	A	G	-0.180968849	0.039091059	3.80626E-06	6411	0.00333179	21.43151039
family.Clostridiaceae1.id.1869	rs550843	6	C	T	-0.073478656	0.016838326	7.09119E-06	14234	0.001336032	19.04252731
family.Clostridiaceae1.id.1869	rs12341505	9	A	G	0.081450333	0.017933832	4.54486E-06	14250	0.001445428	20.62716496
family.Clostridiaceae1.id.1869	rs4723021	7	C	T	-0.106376242	0.024194378	7.42147E-06	13682	0.001410904	19.33126885
family.Clostridiaceae1.id.1869	rs881532	22	G	A	-0.053303513	0.011901062	7.89662E-06	14250	0.00140577	20.06042901
family.Clostridiaceae1.id.1869	rs2817172	1	T	C	0.056336654	0.012392023	5.27026E-06	13789	0.00149663	20.66796232
family.Clostridiaceae1.id.1869	rs10875374	1	T	C	-0.053675937	0.011943586	8.09821E-06	14253	0.001415039	20.19713693
family.Clostridiaceae1.id.1869	rs12490337	3	G	C	-0.06169436	0.013693009	6.90518E-06	14253	0.001422227	20.29986557
family.Clostridiaceae1.id.1869	rs62397761	6	G	A	0.061580452	0.013619788	9.07706E-06	14248	0.001432744	20.44301916
family.Clostridiaceae1.id.1869	rs56188186	16	G	A	0.096777351	0.02174714	8.24444E-06	13218	0.001495985	19.80355538
family.ClostridialesvadinBB60grou	rs55682560	15	T	C	-0.131519432	0.026131877	4.97034E-07	9183	0.002750792	25.33020184
family.ClostridialesvadinBB60grou	rs9979874	21	C	G	-0.073892457	0.015091139	1.05271E-06	10506	0.002276821	23.97486592
family.ClostridialesvadinBB60grou	rs28691777	17	T	C	0.137133741	0.026699624	6.95697E-07	10199	0.002579876	26.38021672
family.ClostridialesvadinBB60grou	rs6588624	1	A	G	-0.066231659	0.013814656	1.79287E-06	10512	0.002181811	22.98534839
family.ClostridialesvadinBB60grou	rs7538034	1	G	T	-0.078597952	0.016598223	2.36706E-06	10420	0.002147326	22.42328736
family.ClostridialesvadinBB60grou	rs66714985	8	C	A	0.116907778	0.025244679	4.85334E-06	10512	0.002035995	21.44603901
family.ClostridialesvadinBB60grou	rs118104867	8	T	C	0.214463834	0.04550976	3.43598E-06	4095	0.005393818	22.20746783
family.ClostridialesvadinBB60grou	rs2191834	2	T	G	0.074637506	0.015913614	2.50195E-06	10511	0.002088453	21.99766637
family.ClostridialesvadinBB60grou	rs7725895	5	G	A	-0.116223759	0.024036718	3.94356E-06	10512	0.002219164	23.37973047
family.ClostridialesvadinBB60grou	rs7226487	18	G	A	-0.064368248	0.013870145	3.58282E-06	10505	0.002045954	21.53680856
family.ClostridialesvadinBB60grou	rs10517600	4	G	T	0.062699295	0.01393642	6.82768E-06	10512	0.001921773	20.24057454
family.ClostridialesvadinBB60grou	rs17121075	14	A	G	0.07692543	0.017223352	7.91417E-06	10512	0.001894069	19.9482369
family.ClostridialesvadinBB60grou	rs10904722	10	T	C	-0.067231397	0.014712327	5.04837E-06	10512	0.001982596	20.88245509
family.ClostridialesvadinBB60grou	rs6755871	2	G	C	-0.061382475	0.013897576	9.33059E-06	10500	0.00185445	19.50790583
family.ClostridialesvadinBB60grou	rs34088226	5	G	A	-0.117806973	0.026923974	7.66216E-06	9716	0.001966624	19.14536623
family.ClostridialesvadinBB60grou	rs13409132	2	G	A	-0.165418684	0.035215443	4.37231E-06	5913	0.003717726	22.0649429
family.ClostridialesvadinBB60grou	rs989682	3	G	A	0.070193983	0.01553641	6.8471E-06	10129	0.00201121	20.41260502
family.Coriobacteriaceae.id.811	rs719099	10	G	A	0.077840173	0.01558151	5.43317E-07	17687	0.001409035	24.95677559
family.Coriobacteriaceae.id.811	rs240104	1	C	T	-0.060342897	0.012684911	1.51603E-06	17577	0.001285801	22.62962954
family.Coriobacteriaceae.id.811	rs11250875	10	C	T	0.060749978	0.013093887	4.82844E-06	17690	0.001215344	21.5255891
family.Coriobacteriaceae.id.811	rs34739816	17	T	G	0.096501674	0.020766582	3.88358E-06	16593	0.00129972	21.59432574

family.Coriobacteriaceae.id.811	rs76779974	5	G	C	0.0774888	0.017187698	5.63091E-06	16419	0.001236398	20.32555641
family.Coriobacteriaceae.id.811	rs80046645	5	G	C	0.255839805	0.056199532	4.65689E-06	3204	0.006426556	20.72386883
family.Coriobacteriaceae.id.811	rs45480394	19	G	T	-0.050426168	0.011330684	9.71448E-06	17413	0.001136141	19.80613186
family.Coriobacteriaceae.id.811	rs1816223	12	G	A	-0.058626474	0.012900576	4.84114E-06	17688	0.001166229	20.65234419
family.Coriobacteriaceae.id.811	rs67561917	20	G	A	-0.071436723	0.015411343	5.39129E-06	17333	0.001238085	21.48632118
family.Coriobacteriaceae.id.811	rs3025411	9	G	A	0.092654285	0.020946781	8.26912E-06	15671	0.001246975	19.56574696
family.Coriobacteriaceae.id.811	rs11656361	17	C	A	0.077300587	0.01755297	8.02017E-06	16665	0.001162396	19.3938654
family.Coriobacteriaceae.id.811	rs1397793	5	A	G	-0.049885455	0.011244635	9.77381E-06	17527	0.001121662	19.68145117
family.Coriobacteriaceae.id.811	rs11073596	15	G	T	0.051021228	0.011433922	8.14449E-06	17527	0.001134777	19.91183886
family.Coriobacteriaceae.id.811	rs2442778	3	A	G	-0.116408329	0.025854513	9.02703E-06	13472	0.001502485	20.27194292
family.Coriobacteriaceae.id.811	rs7540303	1	T	C	0.048322746	0.010906179	9.62148E-06	17691	0.00110847	19.63170353
family.Coriobacteriaceae.id.811	rs62448869	7	A	T	-0.048605943	0.010898242	7.87276E-06	17527	0.001133615	19.89142221
family.Coriobacteriaceae.id.811	rs13307134	7	T	C	0.056568602	0.012626474	7.79931E-06	17527	0.001143884	20.0718175
family.Coriobacteriaceae.id.811	rs12974142	19	A	G	0.078979608	0.017720479	8.51232E-06	17413	0.001139489	19.86456328
family.Coriobacteriaceae.id.811	rs8010111	14	A	G	-0.10337688	0.022928631	6.89624E-06	15996	0.001269193	20.32780913
family.Defluviitaleaceae.id.1924	rs72731813	4	T	C	-0.149751171	0.02934152	2.75777E-07	7786	0.003334344	26.04805198
family.Defluviitaleaceae.id.1924	rs4677103	3	G	A	0.097720319	0.019703264	9.42189E-07	8002	0.003064516	24.5976386
family.Defluviitaleaceae.id.1924	rs55658617	21	C	T	0.17731425	0.03618445	1.40608E-06	6676	0.003583997	24.0128284
family.Defluviitaleaceae.id.1924	rs9725395	1	G	A	-0.138356652	0.029518507	3.4137E-06	8031	0.002728069	21.96905172
family.Defluviitaleaceae.id.1924	rs17051335	4	T	C	-0.13431353	0.029220144	4.58117E-06	8031	0.002624002	21.12879906
family.Defluviitaleaceae.id.1924	rs112893842	9	C	T	0.11076127	0.023254479	2.75032E-06	8203	0.002757976	22.68624503
family.Defluviitaleaceae.id.1924	rs28696126	7	T	A	-0.107106351	0.023817267	5.56327E-06	7830	0.002576111	20.22304734
family.Defluviitaleaceae.id.1924	rs1582238	1	C	T	0.080192339	0.016706104	1.692E-06	8002	0.002871231	23.04174818
family.Defluviitaleaceae.id.1924	rs1908593	18	C	T	0.070222973	0.015660058	7.859E-06	8203	0.00244532	20.10813144
family.Defluviitaleaceae.id.1924	rs9608282	22	G	T	0.139013635	0.029942857	4.60831E-06	7736	0.002778456	21.55402262
family.Defluviitaleaceae.id.1924	rs4344384	10	T	G	0.070880349	0.015612105	5.86011E-06	8200	0.002507406	20.61241081
family.Defluviitaleaceae.id.1924	rs540220	9	C	T	-0.123953592	0.028981413	9.48137E-06	7887	0.002313988	18.29275521
family.Desulfovibrionaceae.id.3169	rs6058181	20	T	C	0.083474185	0.016611075	2.69912E-07	15074	0.001672451	25.25276185
family.Desulfovibrionaceae.id.3169	rs4506934	17	T	C	-0.094338072	0.020137994	3.16043E-06	15035	0.001457487	21.94530251
family.Desulfovibrionaceae.id.3169	rs2692012	1	G	A	0.11423063	0.025356811	1.5639E-06	13080	0.001549155	20.29438451
family.Desulfovibrionaceae.id.3169	rs11599763	10	C	T	-0.055593074	0.011749345	2.49704E-06	15142	0.001476349	22.38793505
family.Desulfovibrionaceae.id.3169	rs2838334	21	A	G	0.057141929	0.012422992	3.82375E-06	15142	0.0013953	21.15715957
family.Desulfovibrionaceae.id.3169	rs112381107	9	T	C	0.211435354	0.045696154	2.81875E-06	4455	0.004782623	21.40897654
family.Desulfovibrionaceae.id.3169	rs16851319	1	C	G	-0.073269696	0.015096375	2.39649E-06	15128	0.001554698	23.55610145
family.Desulfovibrionaceae.id.3169	rs17791387	9	G	A	-0.072925066	0.015438052	2.09678E-06	15045	0.001480924	22.31355022
family.Desulfovibrionaceae.id.3169	rs9928243	16	A	C	-0.054173165	0.011781876	4.48478E-06	15141	0.001394373	21.14168083
family.Desulfovibrionaceae.id.3169	rs7164160	15	T	A	-0.057445479	0.012946842	7.3435E-06	15142	0.001298484	19.68720196
family.Desulfovibrionaceae.id.3169	rs62020470	15	G	A	-0.057412736	0.012946941	7.37851E-06	15142	0.001296986	19.66446522
family.Desulfovibrionaceae.id.3169	rs3935584	2	T	C	-0.052583842	0.011575741	6.78137E-06	15140	0.001361099	20.63511975
family.Desulfovibrionaceae.id.3169	rs72647048	8	C	T	-0.077008881	0.017089038	9.60885E-06	15142	0.00133931	20.30702858
family.Enterobacteriaceae.id.3469	rs78143293	18	G	A	-0.084852127	0.017041521	1.19564E-06	13572	0.001823359	24.79183876
family.Enterobacteriaceae.id.3469	rs111229068	11	T	A	0.110560883	0.024171888	3.65356E-06	12350	0.001691141	20.92097106
family.Enterobacteriaceae.id.3469	rs62210023	20	G	A	0.060675357	0.013012497	3.13163E-06	13515	0.001606162	21.74219515
family.Enterobacteriaceae.id.3469	rs504442	18	G	T	0.084159032	0.01894789	5.17092E-06	13578	0.00145082	19.7278506
family.Enterobacteriaceae.id.3469	rs80319214	2	G	C	0.099040406	0.021587746	6.95307E-06	12641	0.001662288	21.04796975
family.Enterobacteriaceae.id.3469	rs11026530	11	C	T	0.082240818	0.018637912	9.42684E-06	13485	0.001441792	19.47064398
family.Enterobacteriaceae.id.3469	rs2374342	2	A	C	0.058292653	0.01261946	4.51618E-06	13577	0.001569135	21.33762573
family.Enterobacteriaceae.id.3469	rs79757635	13	A	C	0.075859672	0.017128562	9.31857E-06	13419	0.001459572	19.6146289
family.Enterobacteriaceae.id.3469	rs35673018	16	A	G	0.08996415	0.020293422	7.63109E-06	13326	0.001472613	19.65297836
family.Enterobacteriaceae.id.3469	rs61973590	14	G	C	-0.060539207	0.013369618	8.5354E-06	13570	0.00150869	20.50385086
family.Enterobacteriaceae.id.3469	rs4792380	17	T	A	0.115597849	0.025768301	9.48974E-06	12763	0.001574312	20.12463118
family.Erysipelotrichaceae.id.2149	rs62504403	8	T	C	0.068115892	0.012788339	1.12265E-07	16981	0.00166794	28.37060806
family.Erysipelotrichaceae.id.2149	rs7234058	18	C	T	-0.094575892	0.019409083	9.12341E-07	16874	0.001405148	23.74383587
family.Erysipelotrichaceae.id.2149	rs17530232	13	G	A	0.103049982	0.022464754	2.79123E-06	16622	0.001264328	21.04226554
family.Erysipelotrichaceae.id.2149	rs2300774	3	A	G	0.052415201	0.010678379	8.95466E-07	18216	0.001320921	24.09371743
family.Erysipelotrichaceae.id.2149	rs35161940	17	C	T	-0.080604056	0.01676409	1.8451E-06	18213	0.001267714	23.11819028
family.Erysipelotrichaceae.id.2149	rs10781552	10	T	C	-0.055221236	0.011607425	2.33114E-06	17789	0.001270682	22.63291274
family.Erysipelotrichaceae.id.2149	rs8003149	14	T	C	0.053879756	0.01168879	4.08475E-06	18213	0.001165264	21.24770908
family.Erysipelotrichaceae.id.2149	rs4078432	14	T	C	-0.060892934	0.013376363	4.23104E-06	17789	0.001163591	20.72324212
family.Erysipelotrichaceae.id.2149	rs1074800	5	G	A	0.049243827	0.010887044	6.14583E-06	17789	0.00114877	20.45896385
family.Erysipelotrichaceae.id.2149	rs56970041	14	G	T	0.072414447	0.016447064	5.40344E-06	18217	0.001063004	19.38535258
family.Erysipelotrichaceae.id.2149	rs7826267	8	G	T	-0.083907147	0.019913089	9.28449E-06	17586	0.001008592	17.75499888
family.Erysipelotrichaceae.id.2149	rs1884466	1	T	C	-0.047536345	0.01069386	9.52701E-06	18205	0.001084228	19.75979992
family.Erysipelotrichaceae.id.2149	rs290833	1	G	T	-0.049737371	0.011137647	8.029E-06	17789	0.0011198	19.94246102
family.FamilyXI.id.1936	rs2155352	11	G	A	-0.150537618	0.030231918	6.63432E-07	3150	0.007809856	24.79468931
family.FamilyXI.id.1936	rs697771	16	G	A	-0.117677217	0.025149135	3.19372E-06	3152	0.006898365	21.8946832
family.FamilyXI.id.1936	rs10759623	9	T	C	-0.162114556	0.032150976	5.78312E-07	3156	0.007991606	25.42469225
family.FamilyXI.id.1936	rs11547158	7	G	A	-0.177599232	0.037279461	2.70219E-06	3133	0.007191969	22.69566578
family.FamilyXI.id.1936	rs488164	1	G	T	0.117997561	0.025515955	4.79785E-06	3149	0.00674544	21.38564725
family.FamilyXI.id.1936	rs3733511	4	G	A	0.128253369	0.02747177	3.38554E-06	3156	0.006858645	21.79537191
family.FamilyXI.id.1936	rs6025153	20	C	G	0.118618541	0.026196893	7.07084E-06	3130	0.006507673	20.50244113
family.FamilyXI.id.1936	rs78222136	16	C	G	0.224834617	0.051167198	8.41266E-06	3136	0.006119294	19.30825747
family.FamilyXI.id.1936	rs17379710	11	C	T	-0.116383434	0.025212482	3.97107E-06	3156	0.006706435	21.30841359
family.FamilyXI.id.1936	rs2156611	18	C	T	-0.112486332	0.025109889	9.42661E-06	3155	0.006320579	20.06826943
family.FamilyXIII.id.1957	rs6501525	17	G	A	0.056155868	0.011558716	1.23726E-06	16269	0.001448707	23.60320998
family.FamilyXIII.id.1957	rs118170811	10	G	A	0.151619208	0.031711234	1.80357E-06	9645	0.002364568	22.86031179
family.FamilyXIII.id.1957	rs12643275	4	T	A	-0.055090712	0.011647397	2.41523E-06	16269	0.001373224	22.37170244
family.FamilyXIII.id.1957	rs1999289	21	T	A	-0.063736569	0.013926964	2.53805E-06	16269	0.001285715	20.94423271
family.FamilyXIII.id.1957	rs482905	1	T	G	0.059652844	0.012717031	3.72345E-06	16737	0.001312933	22.00345314
family.FamilyXIII.id.1957	rs3098182	15	T	G	0.05085114	0.011014044	4.04024E-06	16731	0.001272427	21.31609999
family.FamilyXIII.id.1957	rs6797051	3	T	C	-0.080598449	0.017136413	4.89281E-06	16737	0.001319965	22.12144771
family.FamilyXIII.id.1957	rs66753613	1	A	G	0.065113557	0.014425125	8.08288E-06	16269	0.001250833	20.37528399
family.FamilyXIII.id.1957	rs7514702	1	C	T	-0.066323634	0.014162562	3.91642E-06	16737	0.001308599	21.93072462
family.FamilyXIII.id.1957	rs7076829	10	A	T	-0.051639411	0.011231289	4.76089E-06	16269	0.001297713	21.13992207
family.FamilyXIII.id.1957	rs10404377	19	A	C	-0.050318303	0.011165005	6.99027E-06	16269	0.001246899	20.31113174
family.FamilyXIII.id.1957	rs4293737	3	G	A	-0.112090617	0.027130494	8.38485E-06	5704	0.002983636	17.06959087
family.FamilyXIII.id.1957	rs1887048	1	A	G	0.057997854	0.012960206	9.65391E-06	16717	0.001196525	20.02626944
family.FamilyXIII.id.1957	rs12904405	15	G	A	-0.085189628	0.019268843	7.67412E-06	16643	0.001173062	19.54619197
family.Lachnospiraceae.id.1987	rs9929145	16	A	G	-0.125721183	0.024523339	2.83594E-07	15866	0.001653756	26.28195764
family.Lachnospiraceae.id.1987	rs11755180	6	G	C	-0.04922703	0.010489407	2.68982E-06	18333	0.001199915	22.02446488
family.Lachnospiraceae.id.1987	rs35524804	9	C	T	-0.060748124	0.012521578	2.44999E-06	17859	0.00131619	23.53681133
family.Lachnospiraceae.id.1987	rs11979110	7	C	T	-0.050092357	0.010506859	1.81843E-06	18339	0.001237894	22.7298775
family.Lachnospiraceae.id.1987	rs79086868	9	C	T	0.077672344	0.016447234	3.01422E-06	17522	0.001271191	22.3021664
family.Lachnospiraceae.id.1987	rs112040820	17	G	A	0.054958365	0.011708564	2.41836E-06	17512	0.001256546	22.03232566
family.Lachnospiraceae.id.1987	rs2159863	4	G	A	-0.058583102	0.012874212	3.69557E-06	18340	0.001127753	20.70634399
family.Lachnospiraceae.id.1987	rs11139361	9	C	T	0.049446281	0.011050331	4.26262E-06	18333	0.001090961	20.02242424
family.Lachnospiraceae.id.1987	rs1205443	20	G	A	0.0501415	0.011215719	7.28844E-06	17779	0.00112291	19.98666692
family.Lachnospiraceae.id.1987	rs7359994	19	C	T	-0.050314217	0.01124817	5.35899E-06	18340	0.001089795	20.00864513
family.Lachnospiraceae.id.1987	rs146660815	6	C	T	-0.215113896	0.047677775	6.53238E-06	4112	0.004926145	20.35658692
family.Lachnospiraceae.id.1987	rs959845	4	T	C	-0.049366584	0.010780126	5.1695E-06	17859	0.001172875	20.97096648
family.Lachnospiraceae.id.1987	rs10402491	19	T	C	0.066170067	0.014850954	7.58351E-06	18334	0.001081651	19.8524643
family.Lachnospiraceae.id.1987	rs13005175	2	G	A	0.099348285	0.021839504	8.37071E-06	16893	0.00122348	20.69355989
family.Lachnospiraceae.id.1987	rs2910921	5	C	T	0.160302066	0.035832011	8.41974E-06	6549	0.003046742	20.0140908
family.Lachnospiraceae.id.1987	rs12760724	1	C	A	-0.048456589	0.010793397	7.26734E-06	18340	0.001097774	20.15530544
family.Lachnospiraceae.id.1987	rs3127230	10	T	C	-0.050353667	0.011220058	6.20325E-06	18340	0.001096973	20.14058349
family.Lachnospiraceae.id.1987	rs11841382	13	T	G	-0.071812797	0.017461974	9.57773E-06	18340	0.000921335	16.91285871
family.Lactobacillaceae.id.1836	rs16861661	1	A	G	-0.193287418	0.037929323	2.70131E-07	5385	0.004799344	25.96910477
family.Lactobacillaceae.id.1836	rs921925	19	C	A	0.0999419	0.020168726	5.77195E-07	7053	0.003469405	24.55490501
family.Lactobacillaceae.id.1836	rs768253	8	G	T	-0.079234443	0.017060426	3.60763E-06	7059	0.003046352	21.56990806
family.Lactobacillaceae.id.1836	rs6092149	20	T	A	-0.082564564	0.017028222	1.38158E-06	7006	0.003344443	23.50979198
family.Lactobacillaceae.id.1836	rs328312	16	A	T	0.082529013	0.016822496	8.69117E-07	7051	0.003401746	24.0675813
family.Lactobacillaceae.id.1836	rs11674854	2	T	C	-0.083022375	0.017526743	2.59545E-06	6750	0.003313163	22.43819247
family.Lactobacillaceae.id.1836	rs77478751	3	G	A	-0.219177718	0.047356625	5.95946E-06	3704	0.005749843	21.42058487
family.Lactobacillaceae.id.1836	rs74599091	1	G	A	0.191612904	0.042503931	7.69522E-06	4951	0.004088079	20.3231632
family.Lactobacillaceae.id.1836	rs9345899	6	G	A	-0.12404185	0.027894638	9.4459E-06	6602	0.002986212	19.77402137
family.Lactobacillaceae.id.1836	rs1590596	13	G	C	-0.103810248	0.022013537	3.89936E-06	6750	0.003283738	22.23825912
family.Lactobacillaceae.id.1836	rs7399658	13	A	G	-0.104569796	0.022031214	4.4209E-06	6707	0.003347734	22.52867511
family.Lactobacillaceae.id.1836	rs1530559	2	A	G	0.077245338	0.017692138	9.65254E-06	6750	0.002816147	19.06267825
family.Lactobacillaceae.id.1836	rs62314653	4	A	C	0.177081987	0.039226901	6.5919E-06	6016	0.003376014	20.37890139
family.Methanobacteriaceae.id.121	rs10202904	2	G	T	-0.121753579	0.023535682	3.0143E-07	3695	0.007190538	26.76146558
family.Methanobacteriaceae.id.121	rs73457410	13	G	A	0.215339755	0.04366952	1.40855E-06	3498	0.006903404	24.3159698
family.Methanobacteriaceae.id.121	rs76029318	13	C	T	0.214769455	0.0446468	1.58427E-06	3497	0.006573617	23.14005323
family.Methanobacteriaceae.id.121	rs6776814	3	C	T	-0.199565931	0.04118249	1.63062E-06	3385	0.006889481	23.48267699
family.Methanobacteriaceae.id.121	rs894996	4	A	C	0.216999069	0.044907554	1.87557E-06	3582	0.006476339	23.34946566
family.Methanobacteriaceae.id.121	rs73031978	3	G	C	-0.203186321	0.042161972	1.90189E-06	3340	0.006905438	23.22453869
family.Methanobacteriaceae.id.121	rs75208022	12	T	C	-0.227244713	0.048763001	5.92103E-06	3203	0.006734651	21.71734467
family.Methanobacteriaceae.id.121	rs12825290	12	G	C	-0.216781075	0.049349641	6.07567E-06	3144	0.006100071	19.29633077
family.Methanobacteriaceae.id.121	rs56131665	5	A	G	0.178661188	0.039297982	6.17992E-06	3572	0.005753111	20.66902243
family.Methanobacteriaceae.id.121	rs11018665	11	T	A	0.111465287	0.025051554	6.51864E-06	3698	0.005325057	19.79748181

family.Methanobacteriaceae.id.121	rs4257531	3	A	G	0.164498365	0.03649593	7.44344E-06	3162	0.006383973	20.31581768
family.Methanobacteriaceae.id.121	rs6508769	19	C	T	0.15351913	0.034451866	8.22529E-06	3319	0.005947052	19.85635359
family.Methanobacteriaceae.id.121	rs73068003	7	T	G	-0.158140381	0.035180317	8.44583E-06	3692	0.005443196	20.20626583
family.Methanobacteriaceae.id.121	rs10424197	19	A	G	-0.111274175	0.024751678	9.27887E-06	3698	0.005435575	20.2106127
family.Oxalobacteraceae.id.2966	rs4428215	3	A	G	0.125609687	0.023005262	4.87931E-08	5167	0.005736604	29.81205348
family.Oxalobacteraceae.id.2966	rs1569853	6	C	T	-0.140042787	0.028170393	7.45179E-07	4997	0.004921344	24.71358051
family.Oxalobacteraceae.id.2966	rs736744	9	T	C	0.105641925	0.020064781	1.49416E-07	5167	0.005336317	27.720674
family.Oxalobacteraceae.id.2966	rs6000536	22	T	C	-0.118382797	0.024135485	7.38718E-07	5166	0.004635461	24.05831192
family.Oxalobacteraceae.id.2966	rs36057338	4	T	G	0.181604096	0.03987425	6.25787E-06	4738	0.004358871	20.74274516
family.Oxalobacteraceae.id.2966	rs7993559	13	A	C	0.092105903	0.020111361	5.03729E-06	4989	0.004186552	20.97451968
family.Oxalobacteraceae.id.2966	rs12002250	9	C	A	0.196145295	0.044523026	5.53202E-06	4726	0.004089902	19.40825241
family.Oxalobacteraceae.id.2966	rs111966731	15	C	T	0.204031062	0.044574616	4.56318E-06	4387	0.004753139	20.9516076
family.Oxalobacteraceae.id.2966	rs17138946	16	T	G	-0.1894439	0.043024819	8.09008E-06	4776	0.00404296	19.38755956
family.Oxalobacteraceae.id.2966	rs11246212	11	C	T	0.136070377	0.029179663	4.50836E-06	5130	0.00422097	21.74536212
family.Oxalobacteraceae.id.2966	rs934049	2	A	G	0.109751235	0.023863562	4.21147E-06	4951	0.004254063	21.15184702
family.Oxalobacteraceae.id.2966	rs12509763	4	G	C	-0.163319668	0.036082292	8.04848E-06	5129	0.003978549	20.48748973
family.Oxalobacteraceae.id.2966	rs561239	12	G	A	0.10552502	0.023676874	7.1938E-06	4989	0.003965728	19.86379271
family.Oxalobacteraceae.id.2966	rs62435498	7	A	C	0.181521209	0.040060813	7.45831E-06	4780	0.004276869	20.5312427
family.Oxalobacteraceae.id.2966	rs80330081	4	C	A	-0.187559843	0.042368782	6.63998E-06	4672	0.004177025	19.59691742
family.Pasteurellaceae.id.3689	rs12191680	6	G	C	0.102213615	0.019718945	3.049E-07	9408	0.002847831	26.86891565
family.Pasteurellaceae.id.3689	rs9382510	6	T	C	-0.088186524	0.016996482	2.476E-07	9410	0.0028527	26.92070048
family.Pasteurellaceae.id.3689	rs10840326	11	G	C	-0.0717634	0.01492293	1.43842E-06	9154	0.002519945	23.12585676
family.Pasteurellaceae.id.3689	rs76022354	10	T	C	0.242889667	0.050040603	1.82858E-06	3692	0.006340869	23.55987662
family.Pasteurellaceae.id.3689	rs72756943	5	A	G	0.139879974	0.030302876	3.35218E-06	8020	0.002649822	21.30803366
family.Pasteurellaceae.id.3689	rs78909003	9	C	T	-0.241159469	0.049837229	2.04893E-06	3638	0.006395167	23.4153618
family.Pasteurellaceae.id.3689	rs6092684	20	T	A	0.068464719	0.014673987	3.44059E-06	9366	0.002318863	21.76894572
family.Pasteurellaceae.id.3689	rs4822728	22	C	T	0.068547871	0.014903232	4.71847E-06	9412	0.002242694	21.15568144
family.Pasteurellaceae.id.3689	rs35510	12	G	A	0.122729003	0.026495751	4.01576E-06	8671	0.002468309	21.45566408
family.Pasteurellaceae.id.3689	rs35509	12	A	G	0.122351799	0.026512106	4.39089E-06	8671	0.002450178	21.29767744
family.Pasteurellaceae.id.3689	rs10965428	9	A	C	-0.119902083	0.025821904	4.29151E-06	8873	0.00242411	21.56139886
family.Pasteurellaceae.id.3689	rs16970009	17	G	A	0.187381473	0.042957182	7.31531E-06	4703	0.004029519	19.02750021
family.Pasteurellaceae.id.3689	rs12050685	15	G	A	-0.067094787	0.015238983	9.18513E-06	9411	0.002055588	19.38498979
family.Pasteurellaceae.id.3689	rs73139353	3	C	A	-0.222572658	0.048463428	8.71314E-06	4047	0.005184712	21.09188361
family.Pasteurellaceae.id.3689	rs6972479	7	G	A	-0.078186189	0.01753656	7.74817E-06	8616	0.002301785	19.87793531
family.Pasteurellaceae.id.3689	rs9895850	17	C	T	-0.176406461	0.041016971	9.08251E-06	4737	0.003889609	18.4970219
family.Pasteurellaceae.id.3689	rs111582866	16	A	G	-0.113957275	0.025640298	7.06627E-06	8616	0.002287377	19.75322337
family.Pasteurellaceae.id.3689	rs62568866	9	A	T	-0.118018253	0.025920615	7.22663E-06	8020	0.002578174	20.73040357
family.Pasteurellaceae.id.3689	rs731534	20	G	C	-0.098650225	0.021631635	9.46292E-06	9115	0.002276517	20.79780299
family.Pasteurellaceae.id.3689	rs9938097	16	C	T	-0.071038642	0.015802434	8.22604E-06	9402	0.002144809	20.20883813
family.Peptococcaceae.id.2024	rs35703006	8	T	G	0.081265478	0.016435533	4.94775E-07	10715	0.002276474	24.44807259
family.Peptococcaceae.id.2024	rs4990837	8	G	A	0.091038704	0.018586948	1.74194E-06	10718	0.002233321	23.99031716
family.Peptococcaceae.id.2024	rs150600492	10	C	A	0.135773843	0.028975651	2.31305E-06	9761	0.002244376	21.95663724
family.Peptococcaceae.id.2024	rs12634826	3	G	T	-0.073988509	0.015093124	1.01109E-06	10381	0.002309547	24.03091229
family.Peptococcaceae.id.2024	rs12992764	2	G	T	0.068421261	0.014113692	1.46158E-06	10444	0.002245214	23.50178596
family.Peptococcaceae.id.2024	rs75898026	13	G	A	-0.082177853	0.017354142	2.0221E-06	10444	0.002142422	22.42349406
family.Peptococcaceae.id.2024	rs117452796	9	G	A	-0.257982233	0.054956896	3.15296E-06	3161	0.006922989	22.0361241
family.Peptococcaceae.id.2024	rs75430375	5	T	C	-0.147940821	0.031749824	3.40849E-06	8375	0.002585736	21.71167904
family.Peptococcaceae.id.2024	rs12144792	1	T	C	0.064399031	0.014124573	5.82242E-06	10718	0.001935765	20.78777484
family.Peptostreptococcaceae.id.20	rs61841503	10	A	G	0.091965839	0.016133103	9.8012E-09	15333	0.002114807	32.49505361
family.Peptostreptococcaceae.id.20	rs4692811	4	T	C	0.064207491	0.012691273	4.21123E-07	16061	0.001591098	25.59534633
family.Peptostreptococcaceae.id.20	rs12377846	9	A	C	-0.252000426	0.051162723	7.26254E-07	3736	0.006451747	24.26024892
family.Peptostreptococcaceae.id.20	rs2644627	8	G	C	-0.055850562	0.011224002	6.33336E-07	16059	0.001539473	24.76051274
family.Peptostreptococcaceae.id.20	rs59987323	22	T	C	-0.053707832	0.011443008	2.70082E-06	15584	0.00141157	22.02900203
family.Peptostreptococcaceae.id.20	rs9573937	13	G	A	-0.069363688	0.01429074	1.70589E-06	16061	0.00146469	23.55889643
family.Peptostreptococcaceae.id.20	rs75819860	18	A	T	0.126263781	0.026838281	2.48734E-06	11697	0.001888655	22.13340274
family.Peptostreptococcaceae.id.20	rs117020988	7	T	C	0.182411122	0.037212625	1.02794E-06	6831	0.0035052	24.02824266
family.Peptostreptococcaceae.id.20	rs1520207	3	T	C	0.052565835	0.011247608	3.16548E-06	16057	0.001358414	21.84171645
family.Peptostreptococcaceae.id.20	rs76982728	7	C	T	0.124202832	0.026692325	3.24058E-06	11185	0.001932032	21.6516105
family.Peptostreptococcaceae.id.20	rs12986312	19	G	T	0.057181848	0.01259472	5.76718E-06	15970	0.001289063	20.61291221
family.Peptostreptococcaceae.id.20	rs10805326	4	A	G	0.0566637	0.012275984	4.03396E-06	16061	0.001324796	21.30576886
family.Peptostreptococcaceae.id.20	rs1467258	17	A	G	0.072583976	0.016232118	7.89804E-06	15584	0.001281431	19.99544654
family.Peptostreptococcaceae.id.20	rs59865771	16	T	C	-0.057376395	0.012719207	7.69029E-06	16040	0.001267043	20.34915737
family.Peptostreptococcaceae.id.20	rs77540684	10	G	T	0.106725055	0.024563751	8.13712E-06	13495	0.001396894	18.87745251
family.Peptostreptococcaceae.id.20	rs6721459	2	G	A	0.050982344	0.011197512	5.07756E-06	16061	0.001289032	20.72986805
family.Porphyromonadaceae.id.943	rs10119172	9	G	C	-0.073732406	0.014258993	1.32426E-07	18022	0.001481468	26.73862798
family.Porphyromonadaceae.id.943	rs60884758	9	T	C	-0.070576241	0.013939023	2.65875E-07	18016	0.001420942	25.6361267
family.Porphyromonadaceae.id.943	rs17065783	3	G	A	-0.059132994	0.012223515	1.79351E-06	18028	0.001296452	23.40278325
family.Porphyromonadaceae.id.943	rs6953849	7	G	A	0.071814891	0.015072427	2.4448E-06	18028	0.001257675	22.70192263
family.Porphyromonadaceae.id.943	rs10858364	9	T	G	0.055315986	0.012084992	4.31063E-06	18028	0.001160798	20.95118431
family.Porphyromonadaceae.id.943	rs3111851	3	G	C	0.058189615	0.012114715	2.19497E-06	18024	0.001278374	23.07090274
family.Porphyromonadaceae.id.943	rs7330827	13	C	T	-0.103841997	0.023735535	8.04891E-06	13754	0.001389681	19.14026743
family.Porphyromonadaceae.id.943	rs35961441	1	C	A	0.091525669	0.020730567	8.37393E-06	16374	0.001189028	19.49231612
family.Porphyromonadaceae.id.943	rs115602804	3	A	G	0.095465391	0.021806057	6.65072E-06	17103	0.001119383	19.16627019
family.Porphyromonadaceae.id.943	rs10762312	10	A	G	-0.052368014	0.011881432	8.70354E-06	18028	0.001076414	19.42650458
family.Porphyromonadaceae.id.943	rs35233670	17	C	T	-0.047357561	0.010601956	7.90987E-06	18024	0.001105796	19.95292712
family.Porphyromonadaceae.id.943	rs864093	4	C	A	-0.052793912	0.011749938	9.598E-06	18027	0.001118631	20.18815118
family.Porphyromonadaceae.id.943	rs1980561	14	G	A	-0.048528896	0.010928377	8.94542E-06	18028	0.001092615	19.71920662
family.Prevotellaceae.id.960	rs9958960	18	A	G	-0.091480658	0.01736113	1.06324E-07	14061	0.001970741	27.76531325
family.Prevotellaceae.id.960	rs4493272	2	C	T	-0.060499212	0.011802729	3.02312E-07	14867	0.001764186	26.27450569
family.Prevotellaceae.id.960	rs3860225	1	G	A	0.083936026	0.016793605	5.49682E-07	14871	0.001677026	24.98094783
family.Prevotellaceae.id.960	rs912860	14	A	G	-0.229046147	0.048312418	9.2992E-07	3479	0.006419145	22.47648671
family.Prevotellaceae.id.960	rs12118202	1	C	T	-0.075198589	0.014726515	5.54234E-07	14869	0.001750559	26.07470519
family.Prevotellaceae.id.960	rs2206482	20	G	T	-0.056910511	0.011748649	1.30377E-06	14808	0.001582068	23.4643832
family.Prevotellaceae.id.960	rs9586501	13	A	G	0.059268113	0.012732933	2.58988E-06	14868	0.001455125	21.66632731
family.Prevotellaceae.id.960	rs12057990	1	T	C	0.058987846	0.013226834	8.9748E-06	14867	0.001336009	19.88901541
family.Prevotellaceae.id.960	rs148376875	3	G	T	0.08451502	0.017916322	2.08029E-06	14289	0.001554864	22.25205178
family.Prevotellaceae.id.960	rs4685827	3	C	T	-0.0679529	0.014540716	2.77192E-06	14867	0.001466843	21.83958639
family.Prevotellaceae.id.960	rs10131942	14	G	C	0.070121224	0.015832621	8.60821E-06	14871	0.001317288	19.61522638
family.Prevotellaceae.id.960	rs34660375	5	G	A	-0.080589392	0.01782913	7.40327E-06	14289	0.001427819	20.43127473
family.Prevotellaceae.id.960	rs7252711	19	G	A	-0.074087571	0.016192575	5.56586E-06	14540	0.001437704	20.93432042
family.Prevotellaceae.id.960	rs3758087	8	C	T	0.056419924	0.012465508	8.60581E-06	14871	0.001375647	20.48542674
family.Prevotellaceae.id.960	rs34071565	11	A	T	-0.156699931	0.034788229	5.60147E-06	8136	0.002487599	20.28957604
family.Prevotellaceae.id.960	rs13069367	3	C	A	-0.053976797	0.011968733	7.39489E-06	14860	0.0013668	20.33844977
family.Prevotellaceae.id.960	rs7975087	12	A	C	-0.060144779	0.013545406	7.59459E-06	14871	0.001324025	19.71568225
family.Prevotellaceae.id.960	rs2278540	3	A	G	0.055424915	0.012326469	8.43592E-06	14847	0.001359887	20.21774325
family.Rhodospirillaceae.id.2717	rs9813022	3	G	A	-0.08424439	0.016358324	2.53124E-07	8498	0.003111246	26.52188503
family.Rhodospirillaceae.id.2717	rs3754624	2	T	C	0.09713892	0.020006404	1.7148E-06	8496	0.002767136	23.57482592
family.Rhodospirillaceae.id.2717	rs55876211	3	T	C	-0.091185056	0.019639521	2.86679E-06	8494	0.002531468	21.556863
family.Rhodospirillaceae.id.2717	rs76784716	2	G	A	0.136049347	0.028576745	1.48988E-06	8498	0.002660075	22.66560883
family.Rhodospirillaceae.id.2717	rs4278423	10	C	T	0.107529262	0.023572494	3.12021E-06	8501	0.002441802	20.8085704
family.Rhodospirillaceae.id.2717	rs1549633	5	C	A	0.099931243	0.021863395	4.70392E-06	8010	0.002601379	20.89139239
family.Rhodospirillaceae.id.2717	rs11591293	10	T	G	0.074318385	0.015872409	2.67148E-06	8500	0.002572582	21.92334526
family.Rhodospirillaceae.id.2717	rs74354280	4	T	C	-0.091189567	0.02049069	6.67295E-06	8501	0.002324323	19.80510449
family.Rhodospirillaceae.id.2717	rs61933850	12	A	G	0.164808335	0.036064663	7.22582E-06	6830	0.003048232	20.88308221
family.Rhodospirillaceae.id.2717	rs7001029	8	T	C	0.117365754	0.026132422	5.3475E-06	8044	0.002501288	20.17081624
family.Rhodospirillaceae.id.2717	rs1035406	5	A	G	-0.113540052	0.025086266	5.84451E-06	8483	0.002408958	20.48453547
family.Rhodospirillaceae.id.2717	rs72714493	1	G	A	0.08168583	0.018079132	7.34924E-06	8497	0.002396793	20.41447944
family.Rhodospirillaceae.id.2717	rs77304857	4	A	C	-0.099718053	0.02244573	6.95498E-06	8500	0.002316615	19.73695412
family.Rhodospirillaceae.id.2717	rs12977163	19	C	G	0.07309515	0.016349062	7.62595E-06	8044	0.0024788	19.98901813
family.Rhodospirillaceae.id.2717	rs13336560	16	T	C	-0.070171864	0.01580613	9.17333E-06	8010	0.002454568	19.70946936
family.Rhodospirillaceae.id.2717	rs4822789	22	C	G	0.073432075	0.01605212	7.00522E-06	8492	0.00245826	20.92699105
family.Rhodospirillaceae.id.2717	rs6679026	1	C	T	0.111983836	0.025108815	9.94679E-06	8497	0.002335485	19.89107424
family.Rhodospirillaceae.id.2717	rs1923415	6	G	A	-0.099923761	0.022732585	9.63701E-06	8501	0.002267691	19.32145742
family.Rikenellaceae.id.967	rs9603208	13	T	G	0.082117307	0.015959564	1.91801E-07	17680	0.001495186	26.47447381
family.Rikenellaceae.id.967	rs1107244	13	A	G	0.087442832	0.017052197	1.91908E-07	17680	0.001485115	26.29588147
family.Rikenellaceae.id.967	rs6563550	13	C	T	0.085582813	0.017061276	2.807E-07	17680	0.001421184	25.1622856
family.Rikenellaceae.id.967	rs1939881	11	A	G	-0.105589494	0.020621272	5.64373E-07	17434	0.001501623	26.218662
family.Rikenellaceae.id.967	rs67705352	1	G	T	-0.054968793	0.01105338	6.57556E-07	17676	0.001397176	24.73103188
family.Rikenellaceae.id.967	rs4264350	15	C	T	-0.052639287	0.010855489	1.34832E-06	17668	0.001329096	23.51371424
family.Rikenellaceae.id.967	rs62532512	9	A	C	-0.050425666	0.010739924	2.76426E-06	17672	0.001245873	22.04453243
family.Rikenellaceae.id.967	rs67281112	7	C	G	0.063581352	0.013745123	3.63475E-06	17199	0.001242565	21.39746069
family.Rikenellaceae.id.967	rs6837275	4	G	A	0.057035488	0.011886994	1.447E-06	17199	0.001336787	23.02217082
family.Rikenellaceae.id.967	rs9578457	13	A	G	-0.141478496	0.031584793	3.99449E-06	9400	0.002129957	20.06433636
family.Rikenellaceae.id.967	rs2833282	21	A	G	0.071113715	0.015696614	4.31106E-06	17680	0.001159601	20.52555006
family.Rikenellaceae.id.967	rs77885767	14	T	C	-0.156209293	0.033648181	2.84694E-06	8478	0.00253568	21.55214784
family.Rikenellaceae.id.967	rs59663348	18	A	G	0.057091499	0.012504754	6.12244E-06	17678	0.001177735	20.84455287
family.Rikenellaceae.id.967	rs8130320	21	G	A	-0.048923962	0.010684335	4.72745E-06	17680	0.001184544	20.96757606
family.Rikenellaceae.id.967	rs74474130	14	G	T	0.137747171	0.029568753	3.61378E-06	11055	0.001959246	21.70197964
family.Rikenellaceae.id.967	rs7242694	18	T	C	-0.061867432	0.013488231	5.80709E-06	17199	0.001221744	21.03847125

family.Rikenellaceae.id.967	rs36021379	21	G	A	-0.065600501	0.014490275	7.1952E-06	17199	0.001190257	20.4956208
family.Rikenellaceae.id.967	rs10217435	9	T	C	-0.088375611	0.019750648	6.5076E-06	17679	0.001131235	20.02175484
family.Rikenellaceae.id.967	rs2447496	8	A	G	-0.054920309	0.012190433	6.0861E-06	17199	0.001178724	20.29680332
family.Rikenellaceae.id.967	rs35909684	8	C	A	-0.0850181	0.01915389	7.09826E-06	17565	0.001120402	19.70193181
family.Rikenellaceae.id.967	rs4783173	16	G	C	0.048151437	0.011000589	7.0641E-06	17086	0.001120107	19.15960777
family.Rikenellaceae.id.967	rs11841382	13	T	G	0.079592451	0.017891517	3.9802E-06	17680	0.001118102	19.79016498
family.Rikenellaceae.id.967	rs2450745	8	C	A	-0.079236666	0.018410759	8.6056E-06	17680	0.001046579	18.52290128
family.Rikenellaceae.id.967	rs10832801	11	C	A	-0.053476324	0.012266628	7.50417E-06	17199	0.001103799	19.00521266
family.Rikenellaceae.id.967	rs9389714	6	T	C	-0.063684112	0.014341562	8.79066E-06	17680	0.001114046	19.71829253
family.Rikenellaceae.id.967	rs6744030	2	T	C	0.069674656	0.015710296	9.31514E-06	17564	0.001118591	19.66893855
family.Ruminococcaceae.id.2050	rs55793120	12	C	T	0.138220105	0.02661521	1.44338E-07	12405	0.002169415	26.97010577
family.Ruminococcaceae.id.2050	rs2113833	2	C	T	0.169146233	0.035500935	1.14466E-06	4140	0.005453431	22.70100393
family.Ruminococcaceae.id.2050	rs17376049	1	C	T	0.084853361	0.017211379	7.29619E-07	18333	0.00132403	24.30562419
family.Ruminococcaceae.id.2050	rs56199908	9	C	T	-0.199326846	0.041044434	1.66414E-06	5618	0.00418044	23.58430275
family.Ruminococcaceae.id.2050	rs4347804	2	G	A	0.157597512	0.034617903	3.94115E-06	4518	0.004566288	20.72512666
family.Ruminococcaceae.id.2050	rs10093275	8	T	C	0.053459485	0.011676961	5.35228E-06	18335	0.00114186	20.95993267
family.Ruminococcaceae.id.2050	rs76724913	1	G	T	0.090105292	0.020310437	9.60494E-06	17516	0.001122379	19.68167494
family.Ruminococcaceae.id.2050	rs1612733	1	C	T	0.108708367	0.023800599	4.22436E-06	15016	0.001387372	20.86172339
family.Ruminococcaceae.id.2050	rs2426816	20	A	T	-0.048015186	0.010601121	6.04464E-06	17774	0.001152836	20.51415401
family.Ruminococcaceae.id.2050	rs3009418	1	A	C	0.092760682	0.021012315	8.69024E-06	16407	0.001186411	19.48857399
family.Ruminococcaceae.id.2050	rs10166469	2	C	T	-0.053336034	0.012020913	8.5216E-06	18314	0.001073783	19.68640947
family.Ruminococcaceae.id.2050	rs1158100	8	G	A	-0.049053898	0.010986476	8.60858E-06	18333	0.001086237	19.9356412
family.Ruminococcaceae.id.2050	rs499272	11	C	G	0.070643126	0.016045997	9.29359E-06	17854	0.001084425	19.38234784
family.Streptococcaceae.id.1850	rs11110281	12	C	T	-0.130554059	0.022594298	1.40137E-08	15316	0.002175164	33.38743621
family.Streptococcaceae.id.1850	rs11110282	12	G	A	-0.127921342	0.02215271	1.44205E-08	15426	0.002156955	33.34511934
family.Streptococcaceae.id.1850	rs2952251	8	G	A	-0.063929755	0.012652464	3.72237E-07	16612	0.0015345	25.53029574
family.Streptococcaceae.id.1850	rs4968759	17	G	A	-0.054403465	0.011127118	8.91881E-07	16624	0.001435913	23.90494207
family.Streptococcaceae.id.1850	rs17708276	8	G	A	-0.082760717	0.016946689	9.73863E-07	16517	0.001441853	23.84947333
family.Streptococcaceae.id.1850	rs395407	5	C	G	-0.082685467	0.017253602	1.3256E-06	16088	0.001425533	22.96671036
family.Streptococcaceae.id.1850	rs35344081	16	A	G	0.06093492	0.012970335	2.63845E-06	16624	0.001325923	22.07141172
family.Streptococcaceae.id.1850	rs2370083	14	T	G	-0.084275076	0.018450934	4.25666E-06	16516	0.001261562	20.86227041
family.Streptococcaceae.id.1850	rs77558518	5	G	A	-0.104238822	0.022806042	3.72194E-06	15161	0.001376047	20.89099539
family.Streptococcaceae.id.1850	rs76717940	3	A	T	0.150605742	0.033407945	3.08935E-06	7720	0.002625575	20.32279919
family.Streptococcaceae.id.1850	rs10028567	4	T	C	-0.093402667	0.019034346	3.72497E-06	16549	0.001452912	24.07923138
family.Streptococcaceae.id.1850	rs72739637	15	G	A	0.092798286	0.019202095	1.82163E-06	15111	0.001543188	23.35515126
family.Streptococcaceae.id.1850	rs16950051	12	G	A	0.107007503	0.023697285	5.33808E-06	14431	0.001410983	20.39065935
family.Streptococcaceae.id.1850	rs11720390	3	A	G	0.10304331	0.022664902	7.24084E-06	15421	0.001338559	20.66959273
family.Streptococcaceae.id.1850	rs77968078	1	A	G	-0.099301329	0.022478765	7.9334E-06	15519	0.001255902	19.5148501
family.Streptococcaceae.id.1850	rs7916711	10	G	A	0.095963939	0.021545035	6.32732E-06	15515	0.001277071	19.83908876
family.Streptococcaceae.id.1850	rs957755	7	G	T	-0.064244942	0.014270246	7.4151E-06	16627	0.001217508	20.2681902
family.Streptococcaceae.id.1850	rs6806351	3	C	T	-0.061920915	0.013574394	6.93793E-06	16198	0.001282965	20.80816855
family.Streptococcaceae.id.1850	rs11764382	7	G	A	-0.063367681	0.014265203	8.85153E-06	16627	0.001185361	19.73238989
family.Streptococcaceae.id.1850	rs9903102	17	A	C	-0.069301513	0.015409626	4.91806E-06	16623	0.001215245	20.22559017
family.Streptococcaceae.id.1850	rs57646748	4	A	G	-0.088020977	0.019875957	7.88359E-06	16160	0.001212127	19.61174678
family.Streptococcaceae.id.1850	rs6563952	16	C	G	0.080193069	0.017857592	8.70591E-06	16627	0.0012114	20.16637714
family.Veillonellaceae.id.2172	rs12741784	1	T	C	-0.062145234	0.011926612	1.28494E-07	15867	0.001708221	27.15072822
family.Veillonellaceae.id.2172	rs79535861	13	C	A	0.100666153	0.020701553	1.57687E-06	15061	0.001567567	23.64618667
family.Veillonellaceae.id.2172	rs111810795	14	T	C	-0.08671084	0.018057347	1.72927E-06	15605	0.001475482	23.05891657
family.Veillonellaceae.id.2172	rs12186441	5	A	G	0.208095725	0.045399862	4.52857E-06	5236	0.003996488	21.00957407
family.Veillonellaceae.id.2172	rs4797169	18	C	T	0.058779888	0.012829669	4.49346E-06	15974	0.001312329	20.99068335
family.Veillonellaceae.id.2172	rs12668619	7	G	A	0.055300975	0.011765373	2.57364E-06	15971	0.001381407	22.09297328
family.Veillonellaceae.id.2172	rs2175069	4	G	A	-0.052675165	0.01148863	4.63558E-06	15976	0.001314123	21.02206231
family.Veillonellaceae.id.2172	rs1442060	4	A	G	-0.051402432	0.011212452	4.50675E-06	15976	0.001313794	21.01677853
family.Veillonellaceae.id.2172	rs1447205	8	C	G	-0.052068875	0.011565567	7.34899E-06	15971	0.001267476	20.2685451
family.Veillonellaceae.id.2172	rs114889439	13	G	A	-0.253809201	0.053916917	6.19028E-06	3268	0.006735156	22.15973835
family.Veillonellaceae.id.2172	rs4461038	15	G	A	-0.055496494	0.011946736	3.73474E-06	15973	0.001349148	21.57906098
family.Veillonellaceae.id.2172	rs6909981	6	T	C	-0.063869651	0.014176514	5.47618E-06	15976	0.00126891	20.29785712
family.Veillonellaceae.id.2172	rs2561116	5	G	T	-0.083616969	0.018715906	7.88518E-06	15976	0.001247834	19.96030369
family.Veillonellaceae.id.2172	rs61264131	6	C	A	0.202366989	0.046438799	6.74544E-06	3075	0.006137599	18.98966806
family.Veillonellaceae.id.2172	rs9345168	6	A	C	0.050910225	0.011325295	8.48887E-06	15976	0.001263263	20.20742459
family.Veillonellaceae.id.2172	rs11700976	21	A	C	0.050333676	0.01137631	9.25376E-06	15971	0.001224194	19.57557354
family.Veillonellaceae.id.2172	rs6692542	1	G	A	0.053439722	0.011825371	8.67975E-06	15976	0.001276662	20.4220258
family.Veillonellaceae.id.2172	rs2585520	13	T	G	-0.090497192	0.020042803	5.26922E-06	15501	0.001313478	20.38699886
family.Veillonellaceae.id.2172	rs75768969	13	G	C	-0.070377296	0.015685477	7.19233E-06	15975	0.001258583	20.13120027
family.Veillonellaceae.id.2172	rs1693340	18	C	T	0.081893428	0.018245018	9.25271E-06	15058	0.001336169	20.14696008
family.Veillonellaceae.id.2172	rs4263802	8	A	G	0.050941954	0.011492112	7.45003E-06	15976	0.001228428	19.64949854
family.Verrucomicrobiaceae.id.403	rs11729256	4	C	T	0.074979802	0.015017677	6.7309E-07	11861	0.002097249	24.92774882
family.Verrucomicrobiaceae.id.403	rs4936098	11	G	A	0.0648691	0.013592808	1.12929E-06	11858	0.001916958	22.77494765
family.Verrucomicrobiaceae.id.403	rs9349825	6	G	A	-0.070435405	0.014712796	2.51287E-06	11858	0.001929042	22.91878632
family.Verrucomicrobiaceae.id.403	rs11252894	10	C	A	0.077087236	0.016605196	2.94793E-06	11864	0.00181325	21.55147129
family.Verrucomicrobiaceae.id.403	rs74542928	4	C	T	0.112123956	0.023642178	1.64613E-06	11216	0.002001308	22.49167857
family.Verrucomicrobiaceae.id.403	rs3995795	10	T	C	0.064217981	0.0140994	5.02531E-06	11690	0.001771444	20.74493291
family.Verrucomicrobiaceae.id.403	rs2602429	16	T	C	0.074551564	0.015619484	2.69939E-06	11690	0.001945002	22.78138613
family.Verrucomicrobiaceae.id.403	rs4242783	10	A	G	0.068799208	0.01476936	2.75444E-06	11690	0.001852779	21.69919
family.Verrucomicrobiaceae.id.403	rs11184341	1	C	G	0.065530075	0.014223048	4.13844E-06	11864	0.001786029	21.22736083
family.Verrucomicrobiaceae.id.403	rs61779207	1	A	G	-0.075881119	0.01677619	6.63029E-06	11591	0.001761952	20.45882901
family.Verrucomicrobiaceae.id.403	rs117107102	18	G	A	0.20468288	0.043157251	2.91821E-06	4975	0.00450094	22.49342052
family.Verrucomicrobiaceae.id.403	rs12908520	15	A	G	0.061910394	0.013094609	2.15138E-06	11863	0.001880745	22.35332159
family.Verrucomicrobiaceae.id.403	rs111862613	12	C	T	0.090706497	0.01967463	3.73127E-06	11621	0.001825688	21.25512351
family.Verrucomicrobiaceae.id.403	rs941682	20	A	G	-0.06315257	0.014376725	9.58114E-06	11809	0.001631323	19.29576917
family.Victivallaceae.id.2255	rs4396289	11	T	C	-0.152917575	0.028852532	1.53782E-07	3913	0.007127391	28.08968892
family.Victivallaceae.id.2255	rs56349194	11	G	A	-0.152330868	0.028857868	1.77942E-07	3915	0.007067007	27.86424926
family.Victivallaceae.id.2255	rs2944282	7	C	T	-0.124196623	0.025702577	1.56601E-06	3555	0.006525045	23.34888917
family.Victivallaceae.id.2255	rs11764871	7	T	G	0.12709902	0.025655983	7.49289E-07	3909	0.00623912	24.54183826
family.Victivallaceae.id.2255	rs6545794	2	G	A	-0.19778562	0.041049541	5.97005E-07	3916	0.005893362	23.21522074
family.Victivallaceae.id.2255	rs2546105	5	T	A	0.126643495	0.025711059	1.02501E-06	3917	0.006155886	24.26195774
family.Victivallaceae.id.2255	rs11770843	7	T	C	0.124345195	0.025836019	1.05598E-06	3891	0.005917903	23.16364148
family.Victivallaceae.id.2255	rs62570196	9	T	C	-0.246101492	0.048307716	2.69852E-07	3091	0.008326552	25.95347364
family.Victivallaceae.id.2255	rs7077363	10	A	G	0.14894216	0.031836622	2.83376E-06	3912	0.005563646	21.88675309
family.Victivallaceae.id.2255	rs2731834	5	G	C	0.123706365	0.025977319	2.3326E-06	3916	0.005757647	22.67751318
family.Victivallaceae.id.2255	rs67832247	2	T	C	-0.123489124	0.026669507	3.23801E-06	3555	0.005994825	21.44013148
family.Victivallaceae.id.2255	rs34962571	12	C	A	-0.186945459	0.04184118	6.24824E-06	3237	0.006129276	19.96282466
family.Victivallaceae.id.2255	rs7860510	9	C	G	0.164855226	0.037512763	9.60089E-06	3912	0.004912581	19.31289248
family.Victivallaceae.id.2255	rs7627405	3	T	C	-0.134158069	0.030142747	8.1942E-06	3555	0.005541343	19.80924528
family.Victivallaceae.id.2255	rs61702987	2	C	T	0.145501293	0.030000733	3.07921E-06	3906	0.005985911	23.52176868
family.Victivallaceae.id.2255	rs7314815	12	A	G	0.101087856	0.02255936	6.40086E-06	3912	0.005106486	20.0791076
family.Victivallaceae.id.2255	rs4764863	12	A	G	0.099823186	0.022519408	9.12318E-06	3916	0.004992666	19.64938377
family.Victivallaceae.id.2255	rs11671100	19	C	A	-0.160034432	0.034947236	4.08042E-06	3331	0.006256062	20.97013368
genus..Clostridiuminnocuumgroup.i	rs6890185	5	C	T	0.113423668	0.023313729	1.12244E-06	4166	0.005649424	23.66921581
genus..Clostridiuminnocuumgroup.i	rs61267978	18	C	T	0.14707953	0.032087502	5.58513E-06	4165	0.005019175	21.01031951
genus..Clostridiuminnocuumgroup.i	rs1948423	3	A	T	-0.108859115	0.023424976	3.4941E-06	4165	0.005158343	21.59589947
genus..Clostridiuminnocuumgroup.i	rs6577484	1	A	G	0.16042475	0.036085736	8.40602E-06	3858	0.005096714	19.76385291
genus..Clostridiuminnocuumgroup.i	rs10074000	5	C	T	-0.102648064	0.022750829	6.99943E-06	4048	0.005003663	20.35668516
genus..Clostridiuminnocuumgroup.i	rs1942371	18	A	G	-0.15793795	0.034186957	4.06337E-06	3971	0.005345942	21.34283461
genus..Clostridiuminnocuumgroup.i	rs40656	5	T	C	0.142664382	0.031102116	8.6152E-06	4048	0.005170818	21.04026644
genus..Clostridiuminnocuumgroup.i	rs71564433	6	A	T	-0.126745947	0.027465666	7.8001E-06	4164	0.005088172	21.29550259
genus..Clostridiuminnocuumgroup.i	rs77845139	15	G	A	-0.11499293	0.02571863	8.40622E-06	4048	0.004914356	19.99155767
genus..Clostridiuminnocuumgroup.i	rs10506058	12	G	A	0.099704805	0.022192573	8.92443E-06	4163	0.004825139	20.18444637
genus..Clostridiuminnocuumgroup.i	rs4869133	5	A	G	-0.180590537	0.040950516	7.24455E-06	3735	0.005179945	19.4478331
genus..Eubacteriumbrachygroup.id.	rs112617308	10	C	T	-0.170873361	0.036284397	2.38033E-06	4001	0.005512387	22.17731188
genus..Eubacteriumbrachygroup.id.	rs9613196	22	A	T	-0.238718472	0.052856104	4.98786E-06	3004	0.006744393	20.39772651
genus..Eubacteriumbrachygroup.id.	rs62348779	5	C	T	-0.201480569	0.043286015	3.78122E-06	3827	0.005629385	21.66562013
genus..Eubacteriumbrachygroup.id.	rs2913110	10	T	C	0.105143062	0.022941973	4.55806E-06	4001	0.005222245	21.00388938
genus..Eubacteriumbrachygroup.id.	rs4862235	4	A	G	0.104806298	0.022575135	3.73143E-06	4000	0.00535945	21.55331471
genus..Eubacteriumbrachygroup.id.	rs12151423	2	G	A	0.101328627	0.022732445	9.2705E-06	3854	0.005128934	19.8688176
genus..Eubacteriumbrachygroup.id.	rs73199919	4	C	T	-0.236716837	0.053133485	8.15524E-06	3008	0.006555225	19.84822557
genus..Eubacteriumbrachygroup.id.	rs6591893	11	A	G	0.108225435	0.024031859	7.34344E-06	3999	0.005045865	20.28074737
genus..Eubacteriumbrachygroup.id.	rs720439	22	G	A	-0.111927002	0.025125245	7.02827E-06	4001	0.004935507	19.8449095
genus..Eubacteriumbrachygroup.id.	rs13139592	4	C	T	-0.145996616	0.032719209	7.96753E-06	3842	0.005155583	19.91039992
genus..Eubacteriumbrachygroup.id.	rs1384962	14	G	A	0.120888267	0.026626427	6.99292E-06	4001	0.005125571	20.61306185
genus..Eubacteriumcoprostanoligen	rs17159861	7	T	C	0.096222758	0.016838828	1.03787E-08	17380	0.001875283	32.65366127
genus..Eubacteriumcoprostanoligen	rs9648214	7	C	T	-0.082871942	0.016430987	2.5203E-07	17379	0.001461597	25.43827964
genus..Eubacteriumcoprostanoligen	rs115325767	1	C	G	0.078332188	0.017471885	2.62197E-06	16555	0.001212676	20.10022729
genus..Eubacteriumcoprostanoligen	rs12906958	15	T	C	-0.053315925	0.011593477	4.34803E-06	17380	0.00121537	21.14883848
genus..Eubacteriumcoprostanoligen	rs4076415	15	G	T	0.051515202	0.011029435	1.98974E-06	16960	0.001284636	21.81545665
genus..Eubacteriumcoprostanoligen	rs6762473	3	A	C	0.052158365	0.011235251	4.26113E-06	17377	0.001238709	21.55174363
genus..Eubacteriumcoprostanoligen	rs10444197	10	G	A	-0.050582278	0.011345529	5.98464E-06	17380	0.001142355	19.87683812
genus..Eubacteriumcoprostanoligen	rs2644213	10	A	G	0.053884526	0.012124458	9.85638E-06	17380	0.001135169	19.75165547
genus..Eubacteriumcoprostanoligen	rs11720857	3	T	C	0.063077578	0.014448482	9.26255E-06	16960	0.001122514	19.05923975

genus..Eubacteriumcoprostanoligen	rs4717831	7	T	A	0.078678267	0.017403801	9.17719E-06	16854	0.001211134	20.43719814
genus..Eubacteriumcoprostanoligen	rs62024432	15	T	C	-0.076969763	0.017210135	7.50207E-06	17271	0.001156782	20.00192282
genus..Eubacteriumcoprostanoligen	rs76898927	3	A	G	0.12305502	0.026637491	4.78979E-06	12197	0.001746625	21.34086561
genus..Eubacteriumcoprostanoligen	rs1020520	7	G	T	-0.059070472	0.013292872	8.88975E-06	17371	0.001135494	19.74709672
genus..Eubacteriumcoprostanoligen	rs11052069	12	C	T	0.047783175	0.01078333	9.37949E-06	17380	0.001128507	19.63560325
genus..Eubacteriumcoprostanoligen	rs79895140	2	C	T	-0.06410167	0.014115288	8.61816E-06	16139	0.001276227	20.62335056
genus..Eubacteriumeligensgroup.id.	rs4583233	16	C	A	0.067020477	0.012812178	2.83852E-07	14327	0.001906273	27.36333995
genus..Eubacteriumeligensgroup.id.	rs265534	10	G	T	-0.056375608	0.01201272	2.26758E-06	14776	0.001488319	22.02418312
genus..Eubacteriumeligensgroup.id.	rs72839198	2	G	C	0.155658995	0.037561222	3.13914E-06	4926	0.003474267	17.17390351
genus..Eubacteriumeligensgroup.id.	rs74606150	14	G	C	-0.196453545	0.042564951	4.24506E-06	5381	0.003943089	21.30175875
genus..Eubacteriumeligensgroup.id.	rs6923695	6	G	T	0.103267603	0.02295245	4.87406E-06	12891	0.001567841	20.24277682
genus..Eubacteriumeligensgroup.id.	rs56080211	2	T	C	0.123092697	0.028274315	9.1429E-06	10699	0.001768351	18.95310632
genus..Eubacteriumeligensgroup.id.	rs158115	14	C	G	0.091527053	0.019061087	9.74185E-06	14678	0.001568394	23.05704315
genus..Eubacteriumeligensgroup.id.	rs2200429	13	G	A	-0.088882459	0.019847898	5.29721E-06	14761	0.001356743	20.05409667
genus..Eubacteriumeligensgroup.id.	rs182318	11	A	G	-0.082494113	0.019561497	8.39837E-06	14777	0.001202079	17.78450377
genus..Eubacteriumeligensgroup.id.	rs12719051	7	G	A	0.091662637	0.020818348	7.11766E-06	12799	0.001512373	19.38617706
genus..Eubacteriumfissicatenagroup	rs3771393	2	T	C	0.130842344	0.026667104	7.37909E-07	3792	0.00630853	24.07381461
genus..Eubacteriumfissicatenagroup	rs2733072	8	A	G	0.109644168	0.022830587	1.49089E-06	3833	0.00598126	23.06412226
genus..Eubacteriumfissicatenagroup	rs7104872	11	A	G	0.138611743	0.029190553	2.7315E-06	3829	0.005854369	22.54838372
genus..Eubacteriumfissicatenagroup	rs151257695	7	G	A	0.209509779	0.045484723	3.09681E-06	3587	0.005880102	21.21668341
genus..Eubacteriumfissicatenagroup	rs11876297	18	C	T	0.131469398	0.028171203	2.66556E-06	3396	0.006372276	21.77903112
genus..Eubacteriumfissicatenagroup	rs6934739	6	G	A	0.111463384	0.025278805	9.75326E-06	3831	0.005049411	19.44246715
genus..Eubacteriumfissicatenagroup	rs10147907	14	G	T	0.172262807	0.039600983	8.27246E-06	3833	0.004912399	18.92217766
genus..Eubacteriumfissicatenagroup	rs1768152	3	C	T	0.139488571	0.031618584	8.70054E-06	3590	0.005392	19.46222173
genus..Eubacteriumfissicatenagroup	rs11818408	10	A	G	0.10585035	0.023711404	8.20015E-06	3590	0.005520412	19.92829173
genus..Eubacteriumhalliigroup.id.11	rs13116360	4	C	T	0.154124445	0.029718533	2.94487E-07	10323	0.002598677	26.8960382
genus..Eubacteriumhalliigroup.id.11	rs949971	3	G	T	-0.054016364	0.011610071	3.29002E-06	16566	0.001304957	21.64616455
genus..Eubacteriumhalliigroup.id.11	rs10798999	1	T	C	0.060166074	0.012675245	2.60558E-06	16561	0.001358669	22.53152775
genus..Eubacteriumhalliigroup.id.11	rs60254196	7	G	A	-0.052283163	0.011186491	2.69672E-06	16238	0.001343444	21.84419042
genus..Eubacteriumhalliigroup.id.11	rs10808115	7	C	A	-0.050474741	0.010992248	4.42448E-06	16566	0.001271174	21.08507723
genus..Eubacteriumhalliigroup.id.11	rs74018587	15	T	C	0.208942799	0.043821974	3.70257E-06	3039	0.007425121	22.73374314
genus..Eubacteriumhalliigroup.id.11	rs6550770	3	C	T	-0.198087408	0.044354487	4.823E-06	4279	0.004639559	19.94520979
genus..Eubacteriumhalliigroup.id.11	rs10501370	11	T	C	-0.115588894	0.025248889	5.41918E-06	13824	0.001513757	20.95789618
genus..Eubacteriumhalliigroup.id.11	rs281379	19	G	A	-0.049952328	0.011215293	9.32796E-06	16130	0.00122835	19.83765067
genus..Eubacteriumhalliigroup.id.11	rs78056098	11	T	G	-0.050741114	0.011375822	8.28818E-06	16568	0.001199398	19.89548186
genus..Eubacteriumhalliigroup.id.11	rs138531890	3	G	A	0.153138471	0.034945641	5.4306E-06	7710	0.002484551	19.20359686
genus..Eubacteriumhalliigroup.id.11	rs28584818	3	G	A	0.126115364	0.026862747	4.43235E-06	11095	0.001982649	22.041195
genus..Eubacteriumhalliigroup.id.11	rs117748144	11	C	T	-0.126581958	0.028711955	7.86002E-06	10861	0.001786371	19.43650093
genus..Eubacteriumhalliigroup.id.11	rs17474256	1	A	G	0.081080509	0.018457309	9.45445E-06	16460	0.001171002	19.2972843
genus..Eubacteriumhalliigroup.id.11	rs630939	18	T	C	-0.050891423	0.011435211	9.15759E-06	16238	0.001218257	19.80618606
genus..Eubacteriumhalliigroup.id.11	rs17074066	4	C	T	-0.081398624	0.018927232	9.34905E-06	15804	0.00116892	18.49523868
genus..Eubacteriumnodatumgroup.i	rs34297067	14	G	A	-0.186906548	0.034147492	6.59784E-08	3271	0.009075932	29.95928327
genus..Eubacteriumnodatumgroup.i	rs113893692	9	T	C	-0.18510716	0.040357325	5.75702E-06	3195	0.006541548	21.03786592
genus..Eubacteriumnodatumgroup.i	rs77910827	9	T	C	0.201788441	0.041356826	9.05381E-07	3271	0.007225505	23.80664341
genus..Eubacteriumnodatumgroup.i	rs61841040	10	T	G	0.160621141	0.034160929	3.56141E-06	3295	0.00666479	22.10782801
genus..Eubacteriumnodatumgroup.i	rs9425984	1	C	T	-0.130233832	0.029234653	7.20681E-06	3294	0.005988521	19.84502895
genus..Eubacteriumnodatumgroup.i	rs10263623	7	T	C	0.193495904	0.043902338	8.90706E-06	3080	0.006267395	19.4253235
genus..Eubacteriumnodatumgroup.i	rs11006576	10	G	A	-0.110167094	0.024592963	7.98664E-06	3295	0.006053266	20.06698206
genus..Eubacteriumnodatumgroup.i	rs7880204	1	C	T	-0.125453808	0.027530312	6.83884E-06	3294	0.006264589	20.76564492
genus..Eubacteriumnodatumgroup.i	rs6818880	4	G	A	-0.110080596	0.024596445	7.8254E-06	3291	0.00604942	20.02981087
genus..Eubacteriumnodatumgroup.i	rs10458299	7	C	T	-0.187752781	0.041969011	8.36714E-06	3271	0.006081149	20.01314263
genus..Eubacteriumnodatumgroup.i	rs7827125	8	T	C	0.122299815	0.027133143	7.17355E-06	3294	0.006129956	20.31661668
genus..Eubacteriumoxidoreducensg	rs12423772	12	T	G	0.140979641	0.029502889	2.62925E-06	5321	0.004272979	22.83409009
genus..Eubacteriumoxidoreducensg	rs2973294	4	T	G	0.092355871	0.019543871	2.38836E-06	5378	0.004135114	22.33098268
genus..Eubacteriumoxidoreducensg	rs34561138	16	A	G	0.216145345	0.04599197	2.51163E-06	4186	0.005248595	22.08654364
genus..Eubacteriumoxidoreducensg	rs440215	5	T	C	0.093276592	0.019529391	1.65407E-06	5377	0.004224636	22.81224118
genus..Eubacteriumoxidoreducensg	rs1425962	4	C	G	0.090610394	0.020061232	7.32284E-06	5321	0.003819317	20.40050024
genus..Eubacteriumoxidoreducensg	rs12129908	1	A	C	0.08932725	0.019833287	5.79516E-06	5377	0.003758401	20.28516458
genus..Eubacteriumrectalegroup.id.	rs35398954	15	G	A	-0.090140156	0.017460171	5.39945E-07	16621	0.001600983	26.65260249
genus..Eubacteriumrectalegroup.id.	rs117151453	12	C	G	-0.113140609	0.02435414	1.84432E-06	13908	0.001549364	21.58198685
genus..Eubacteriumrectalegroup.id.	rs16960159	18	G	C	-0.156595964	0.03362338	4.10132E-06	8334	0.002595948	21.6909416
genus..Eubacteriumrectalegroup.id.	rs314726	2	C	T	0.052882354	0.010948624	1.37967E-06	16966	0.001373176	23.32934172
genus..Eubacteriumrectalegroup.id.	rs10797540	1	G	A	0.050317907	0.010838946	3.53384E-06	17350	0.001240602	21.55117894
genus..Eubacteriumrectalegroup.id.	rs10248854	7	A	C	-0.052782677	0.01134635	4.20676E-06	17348	0.00124589	21.64065755
genus..Eubacteriumrectalegroup.id.	rs3980709	1	T	A	-0.062270145	0.014057159	6.85524E-06	17340	0.00113038	19.62296565
genus..Eubacteriumrectalegroup.id.	rs2884897	11	G	A	-0.129363699	0.028902068	6.44426E-06	10813	0.001849341	20.03397006
genus..Eubacteriumrectalegroup.id.	rs10892089	11	G	C	-0.063552174	0.014007233	6.21821E-06	17351	0.001184995	20.58524787
genus..Eubacteriumrectalegroup.id.	rs143694765	1	C	T	0.087002523	0.01977896	9.75369E-06	15124	0.001277718	19.34892358
genus..Eubacteriumrectalegroup.id.	rs59427698	9	G	A	-0.057603509	0.013085337	5.36906E-06	16613	0.001165128	19.37885322
genus..Eubacteriumrectalegroup.id.	rs62547233	9	G	A	0.0536319	0.012020063	9.8982E-06	17347	0.001146332	19.90824209
genus..Eubacteriumruminantiumgro	rs2116427	5	G	A	0.091146051	0.018235307	4.67364E-07	7739	0.003217844	24.98328645
genus..Eubacteriumruminantiumgro	rs139749	22	T	C	-0.084543927	0.017179078	8.58845E-07	7314	0.00330046	24.21949921
genus..Eubacteriumruminantiumgro	rs72836424	10	T	C	-0.139824589	0.030068963	2.62125E-06	7314	0.002947767	21.6237092
genus..Eubacteriumruminantiumgro	rs2229917	9	G	A	0.153537929	0.032392215	2.15894E-06	7050	0.003176721	22.46725764
genus..Eubacteriumruminantiumgro	rs10131724	14	C	A	-0.199832279	0.041457713	2.38663E-06	5071	0.004560807	23.2338186
genus..Eubacteriumruminantiumgro	rs16891896	5	A	G	-0.174787011	0.039057268	2.3812E-06	4753	0.004195857	20.02693936
genus..Eubacteriumruminantiumgro	rs7000472	8	G	A	-0.076228208	0.016523022	4.06848E-06	7739	0.002742678	21.28396208
genus..Eubacteriumruminantiumgro	rs13025464	2	C	T	-0.073707715	0.016378577	6.96842E-06	7738	0.002610416	20.25226245
genus..Eubacteriumruminantiumgro	rs2418654	2	T	C	-0.074887891	0.016585161	6.17247E-06	7314	0.002779841	20.38843204
genus..Eubacteriumruminantiumgro	rs6676699	1	T	G	-0.088812364	0.019644717	6.37979E-06	7708	0.002644622	20.43879546
genus..Eubacteriumruminantiumgro	rs73139629	12	C	A	-0.115097848	0.024790941	5.36199E-06	7314	0.00293843	21.55501777
genus..Eubacteriumruminantiumgro	rs112375806	22	A	T	0.143141085	0.029375749	5.82166E-06	7123	0.003322328	23.74382611
genus..Eubacteriumruminantiumgro	rs606117	9	G	A	0.083324266	0.018055987	4.81639E-06	7263	0.002923566	21.29612221
genus..Eubacteriumruminantiumgro	rs57340348	6	C	T	-0.097942933	0.021216595	4.93278E-06	7314	0.002905202	21.31055919
genus..Eubacteriumruminantiumgro	rs2817174	1	T	C	-0.073430579	0.016368685	7.8696E-06	7710	0.002603393	20.12455534
genus..Eubacteriumruminantiumgro	rs10923018	1	A	G	0.072643786	0.016092406	6.79541E-06	7739	0.002626202	20.37769252
genus..Eubacteriumruminantiumgro	rs11637981	15	T	G	-0.073257996	0.016088796	5.43671E-06	7718	0.002679126	20.73303971
genus..Eubacteriumruminantiumgro	rs17519472	12	T	C	0.10780414	0.023398378	4.70356E-06	7710	0.002745686	21.22752649
genus..Eubacteriumruminantiumgro	rs209813	6	A	G	-0.103488116	0.023639057	9.23146E-06	7264	0.002631482	19.16552202
genus..Eubacteriumventriosumgrou	rs6048195	20	T	A	-0.060359432	0.011657994	2.50324E-07	14839	0.001803243	26.80665577
genus..Eubacteriumventriosumgrou	rs73615400	20	C	T	-0.095630317	0.01934276	9.53613E-07	15146	0.001611224	24.44298923
genus..Eubacteriumventriosumgrou	rs57199565	3	C	T	0.078339435	0.01602571	7.97037E-07	14913	0.001599801	23.89605995
genus..Eubacteriumventriosumgrou	rs11617697	13	G	A	-0.143348528	0.028624073	7.21757E-07	11497	0.002176671	25.07977383
genus..Eubacteriumventriosumgrou	rs16884680	8	T	G	-0.090642843	0.019190901	1.73969E-06	15289	0.001457015	22.30880381
genus..Eubacteriumventriosumgrou	rs12964517	18	A	G	0.058719806	0.012345332	2.07499E-06	15289	0.001477551	22.62370066
genus..Eubacteriumventriosumgrou	rs876734	12	T	C	-0.061869648	0.013253231	2.88908E-06	14913	0.001459192	21.79273451
genus..Eubacteriumventriosumgrou	rs3809430	14	C	T	-0.054817114	0.011842566	3.55317E-06	15287	0.00139962	21.42598227
genus..Eubacteriumventriosumgrou	rs72783037	16	A	C	0.065892572	0.014374684	6.54762E-06	15286	0.001372732	21.01243233
genus..Eubacteriumventriosumgrou	rs73849225	4	C	T	0.097571148	0.022438827	5.20909E-06	14573	0.001295778	18.90786608
genus..Eubacteriumventriosumgrou	rs9316536	13	G	T	-0.081711077	0.018312095	7.83519E-06	15289	0.001300593	19.91066648
genus..Eubacteriumventriosumgrou	rs35179274	14	T	C	-0.062726329	0.013785975	5.75792E-06	15287	0.00135243	20.70259406
genus..Eubacteriumventriosumgrou	rs78250280	1	A	G	0.074976242	0.016440448	3.35791E-06	14835	0.001399987	20.79792803
genus..Eubacteriumventriosumgrou	rs66746423	1	T	C	0.0751501	0.016497843	6.11142E-06	14578	0.001421311	20.74936413
genus..Eubacteriumventriosumgrou	rs6704822	2	G	A	0.073917922	0.016670495	6.6223E-06	15289	0.001284297	19.66086008
genus..Eubacteriumventriosumgrou	rs13082419	3	T	C	-0.071552205	0.016136969	9.55987E-06	14501	0.001353991	19.66084294
genus..Eubacteriumventriosumgrou	rs66830358	3	A	T	0.052875209	0.011797227	6.86917E-06	14913	0.001345224	20.08835399
genus..Eubacteriumxylanophilumgr	rs17830032	20	A	G	-0.160597431	0.031051453	2.38893E-07	9527	0.00279988	26.74935604
genus..Eubacteriumxylanophilumgr	rs13239072	7	A	G	0.068742734	0.014272832	1.81783E-06	13307	0.00174019	23.19708016
genus..Eubacteriumxylanophilumgr	rs79582700	17	G	C	-0.095167651	0.019996897	2.40981E-06	13256	0.001705688	22.64923291
genus..Eubacteriumxylanophilumgr	rs12980122	19	G	C	-0.108273525	0.023825715	5.01935E-06	12039	0.00171245	20.65155067
genus..Eubacteriumxylanophilumgr	rs2012708	16	G	A	0.057320248	0.012690502	6.5254E-06	13306	0.001530896	20.40133133
genus..Eubacteriumxylanophilumgr	rs2213117	11	G	T	0.087779736	0.018901228	4.21329E-06	12878	0.001671989	21.56793081
genus..Eubacteriumxylanophilumgr	rs10140184	14	C	A	0.057679453	0.01259899	4.95862E-06	12930	0.001618337	20.9590121
genus..Eubacteriumxylanophilumgr	rs10917203	1	C	A	0.061263327	0.013091688	3.15155E-06	12930	0.001690738	21.89827113
genus..Eubacteriumxylanophilumgr	rs112176119	16	T	C	-0.113455468	0.024576196	3.32766E-06	12199	0.001743971	21.31186985
genus..Eubacteriumxylanophilumgr	rs1999224	9	T	G	-0.094905143	0.020350281	3.75492E-06	13309	0.001631489	21.74896796
genus..Eubacteriumxylanophilumgr	rs75586835	4	G	A	-0.114497555	0.026345472	9.38708E-06	12550	0.001502739	18.88775978
genus..Eubacteriumxylanophilumgr	rs4654122	1	G	C	0.055350808	0.012358794	7.19609E-06	13309	0.001504861	20.05837412
genus..Ruminococcusgauvreauiigro	rs2047242	10	G	A	-0.06760301	0.013373773	2.46372E-07	13369	0.001907638	25.5519558
genus..Ruminococcusgauvreauiigro	rs71386687	16	G	T	0.121036908	0.02385996	2.90761E-07	11340	0.002264116	25.7333428
genus..Ruminococcusgauvreauiigro	rs9870933	3	G	A	0.062164279	0.012607163	8.4912E-07	13382	0.001813584	24.31347793
genus..Ruminococcusgauvreauiigro	rs1391597	12	T	C	0.059028381	0.012487351	1.85964E-06	13382	0.001667	22.34503816
genus..Ruminococcusgauvreauiigro	rs431418	5	G	A	-0.094737043	0.02101571	5.53667E-06	13289	0.001526847	20.32130131
genus..Ruminococcusgauvreauiigro	rs10931481	2	A	G	0.061009638	0.013048446	3.38211E-06	13382	0.001630983	21.8614683
genus..Ruminococcusgauvreauiigro	rs289410	15	A	G	-0.065492577	0.013910104	2.27464E-06	13380	0.001654049	22.16784122
genus..Ruminococcusgauvreauiigro	rs2105937	1	G	A	0.058021543	0.012780587	5.10352E-06	13364	0.001539825	20.60995143
genus..Ruminococcusgauvreauiigro	rs2166943	8	C	A	0.056697379	0.012349673	5.27717E-06	13377	0.001573159	21.07730397
genus..Ruminococcusgauvreauiigro	rs12539819	7	T	C	0.110653536	0.02406836	4.49375E-06	11860	0.001779015	21.13672031
genus..Ruminococcusgauvreauiigro	rs12079579	1	G	A	0.095532379	0.021343619	5.03725E-06	12986	0.001540353	20.03387913

genus..Ruminococcusgauvreauiigro	rs73802842	4	A	C	0.07368081	0.016966478	7.48026E-06	13382	0.00140732	18.85929142
genus..Ruminococcusgauvreauiigro	rs13188803	5	A	T	0.070941873	0.015685519	7.27559E-06	12834	0.001591306	20.45537686
genus..Ruminococcusgnavusgroup.i	rs13163520	5	A	G	-0.127377481	0.023388325	5.61015E-08	6326	0.004666877	29.66108634
genus..Ruminococcusgnavusgroup.i	rs934940	2	C	A	-0.105044905	0.022958834	2.73705E-06	6309	0.003307129	20.93390821
genus..Ruminococcusgnavusgroup.i	rs9872758	3	C	T	0.084923254	0.017664313	1.66029E-06	6398	0.00359956	23.11318199
genus..Ruminococcusgnavusgroup.i	rs2909242	8	A	C	-0.090996476	0.018351012	7.40957E-07	6401	0.003826628	24.58833387
genus..Ruminococcusgnavusgroup.i	rs3124783	9	G	A	-0.115989721	0.02490991	2.6652E-06	6401	0.003375812	21.68176764
genus..Ruminococcusgnavusgroup.i	rs62167033	2	C	T	0.185289033	0.039629529	3.49529E-06	5194	0.004191173	21.86057624
genus..Ruminococcusgnavusgroup.i	rs12989336	2	A	G	-0.084686061	0.01879361	7.11808E-06	6401	0.003162133	20.30502054
genus..Ruminococcusgnavusgroup.i	rs12136548	1	T	C	0.090194566	0.019647574	3.09631E-06	6401	0.003281463	21.07380058
genus..Ruminococcusgnavusgroup.i	rs78399089	3	C	T	0.144444798	0.032661769	6.62998E-06	5944	0.003279585	19.55799831
genus..Ruminococcusgnavusgroup.i	rs11597105	10	G	A	0.114672834	0.025065269	6.94868E-06	6328	0.003296672	20.93034268
genus..Ruminococcusgnavusgroup.i	rs11864644	16	C	T	-0.139803729	0.031825335	5.0088E-06	6241	0.003082454	19.29707821
genus..Ruminococcusgnavusgroup.i	rs4388134	4	T	C	-0.090499668	0.020354415	9.11637E-06	6399	0.003079818	19.76863789
genus..Ruminococcustorquesgroup.	rs35866622	19	C	T	-0.061202384	0.010942125	2.21337E-08	17240	0.001811376	31.28479748
genus..Ruminococcustorquesgroup.	rs281379	19	G	A	-0.060022655	0.010850935	3.34821E-08	17240	0.001771694	30.59821157
genus..Ruminococcustorquesgroup.	rs773123	12	A	T	0.082400465	0.017386159	1.58956E-06	17011	0.001318709	22.46218514
genus..Ruminococcustorquesgroup.	rs2548459	19	T	C	-0.05190054	0.010655845	1.12416E-06	17794	0.001331422	23.72290895
genus..Ruminococcustorquesgroup.	rs646327	19	A	G	-0.050827337	0.010652289	1.88788E-06	17792	0.001277994	22.76715737
genus..Ruminococcustorquesgroup.	rs10904297	10	G	A	-0.167804385	0.038981237	2.68713E-06	3143	0.005861354	18.53085145
genus..Ruminococcustorquesgroup.	rs8080469	17	A	G	0.049072662	0.010703625	3.49522E-06	17798	0.001179597	21.01926089
genus..Ruminococcustorquesgroup.	rs77034621	8	G	T	-0.151552842	0.033587775	6.06872E-06	8567	0.002370861	20.35943381
genus..Ruminococcustorquesgroup.	rs73130967	7	T	A	0.077037483	0.016826423	3.70741E-06	17011	0.00123071	20.96141311
genus..Ruminococcustorquesgroup.	rs1972694	15	A	T	-0.061409435	0.013732055	8.92829E-06	17353	0.00115113	19.99857811
genus..Ruminococcustorquesgroup.	rs12434631	14	G	A	0.074696994	0.015340053	2.77247E-06	17353	0.001364536	23.71114405
genus..Ruminococcustorquesgroup.	rs4073731	11	C	T	0.065194801	0.014222118	4.0525E-06	17793	0.001179602	21.0134496
genus..Ruminococcustorquesgroup.	rs13154778	5	A	T	0.056335821	0.012952644	7.15746E-06	17795	0.001061923	18.91700676
genus..Ruminococcustorquesgroup.	rs1475330	6	C	T	0.052349242	0.011820049	8.13435E-06	17353	0.00112906	19.6147244
genus..Ruminococcustorquesgroup.	rs10967781	9	A	C	0.050799379	0.011332229	8.36865E-06	17791	0.001128224	20.09491156
genus..Ruminococcustorquesgroup.	rs8141465	22	G	A	0.048077756	0.010744211	9.64837E-06	17791	0.001124218	20.02347062
genus.Actinomyces.id.423	rs34583783	6	T	G	0.126595837	0.026846063	4.48529E-06	7468	0.002968808	22.23707301
genus.Actinomyces.id.423	rs2715439	15	T	C	0.074668399	0.016482184	6.27E-06	7468	0.002740615	20.52315624
genus.Actinomyces.id.423	rs4146653	10	A	G	0.098522403	0.021418242	4.49647E-06	7468	0.002825331	21.15935112
genus.Actinomyces.id.423	rs35011108	6	G	A	0.232633598	0.051204357	6.33832E-06	3515	0.005837984	20.64101457
genus.Actinomyces.id.423	rs4073240	6	A	G	0.074968682	0.016736817	7.94274E-06	7468	0.002679443	20.06383799
genus.Actinomyces.id.423	rs7915461	10	C	T	0.187759792	0.040163611	5.91984E-06	5282	0.004120483	21.85444028
genus.Actinomyces.id.423	rs71315246	3	G	A	-0.09698092	0.021925041	9.82978E-06	7468	0.002613071	19.56553685
genus.Actinomyces.id.423	rs10787984	10	C	G	0.094315957	0.021351309	9.62305E-06	7468	0.002606056	19.51287854
genus.Adlercreutzia.id.812	rs7680684	4	T	C	-0.083381015	0.016890088	9.76904E-07	8243	0.002947835	24.37084213
genus.Adlercreutzia.id.812	rs2147798	1	G	C	0.092295902	0.019212124	1.39877E-06	8243	0.002791994	23.0788463
genus.Adlercreutzia.id.812	rs80078995	5	T	A	-0.113239745	0.023258622	1.57358E-06	8243	0.00286746	23.704445
genus.Adlercreutzia.id.812	rs2717140	18	T	C	-0.119225209	0.025108114	2.04594E-06	8243	0.00272795	22.54799861
genus.Adlercreutzia.id.812	rs9490822	6	T	C	-0.073451202	0.015578896	2.54421E-06	8243	0.002689488	22.22923132
genus.Adlercreutzia.id.812	rs12522517	5	T	A	-0.104884371	0.023469475	4.41254E-06	8187	0.002433503	19.97168904
genus.Adlercreutzia.id.812	rs13231526	7	A	C	0.143237051	0.031165443	4.81355E-06	8051	0.002616835	21.12341556
genus.Adlercreutzia.id.812	rs6664405	1	C	T	-0.095308388	0.02107517	5.23422E-06	8051	0.002533777	20.45126
genus.Adlercreutzia.id.812	rs1046175	10	G	C	0.112757932	0.025599407	6.35889E-06	8051	0.002404027	19.40145914
genus.Adlercreutzia.id.812	rs9915817	17	C	T	0.074921889	0.016833007	8.21673E-06	8243	0.002397542	19.81043671
genus.Adlercreutzia.id.812	rs55719207	3	A	G	-0.069924275	0.015803609	9.60505E-06	8243	0.002369343	19.57687586
genus.Adlercreutzia.id.812	rs11604400	11	T	C	-0.102524228	0.023483368	9.74463E-06	8107	0.00234559	19.06040971
genus.Akkermansia.id.4037	rs11729256	4	C	T	0.075047253	0.015018395	6.58393E-07	11860	0.002100992	24.9702293
genus.Akkermansia.id.4037	rs4936098	11	G	A	0.064922492	0.01359338	1.10359E-06	11857	0.001920109	22.81053301
genus.Akkermansia.id.4037	rs9349825	6	G	A	-0.070340692	0.014713291	2.59886E-06	11857	0.0019239	22.85565151
genus.Akkermansia.id.4037	rs11252894	10	C	A	0.076857793	0.016606095	3.15464E-06	11863	0.001802448	21.42105125
genus.Akkermansia.id.4037	rs74542928	4	C	T	0.112622737	0.023643438	1.4802E-06	11215	0.002019082	22.68981297
genus.Akkermansia.id.4037	rs3995795	10	T	C	0.064117532	0.014099927	5.21945E-06	11689	0.001765936	20.67853904
genus.Akkermansia.id.4037	rs2602429	16	T	C	0.074535241	0.015620127	2.71569E-06	11689	0.001944159	22.76953711
genus.Akkermansia.id.4037	rs61779207	1	A	G	-0.076053856	0.01677691	6.32116E-06	11590	0.001769969	20.5503164
genus.Akkermansia.id.4037	rs11184341	1	C	G	0.065585479	0.01422364	4.06353E-06	11863	0.001789047	21.26149809
genus.Akkermansia.id.4037	rs4242783	10	A	G	0.068545381	0.014770062	2.99799E-06	11689	0.001839141	21.53732431
genus.Akkermansia.id.4037	rs117107102	18	G	A	0.204406166	0.043162894	3.01139E-06	4974	0.004488563	22.42677745
genus.Akkermansia.id.4037	rs12908520	15	A	G	0.061772023	0.01309539	2.26482E-06	11862	0.001872298	22.25085925
genus.Akkermansia.id.4037	rs111862613	12	C	T	0.091119928	0.019674815	3.39236E-06	11620	0.001842461	21.44891851
genus.Akkermansia.id.4037	rs941682	20	A	G	-0.063295745	0.01437768	9.17126E-06	11808	0.001638637	19.38078793
genus.Alistipes.id.968	rs67281112	7	C	G	0.063457498	0.013784589	3.91578E-06	17090	0.001238506	21.19230614
genus.Alistipes.id.968	rs67705352	1	G	T	-0.053240398	0.011086401	1.65123E-06	17567	0.001311095	23.06223817
genus.Alistipes.id.968	rs11769002	7	A	G	-0.052879265	0.010938851	1.44962E-06	17571	0.00132817	23.36831428
genus.Alistipes.id.968	rs7129639	11	A	C	-0.052495538	0.010958377	1.78422E-06	17571	0.001304335	22.94839633
genus.Alistipes.id.968	rs1689282	9	C	A	-0.052006142	0.011394196	5.27578E-06	17090	0.001217505	20.8325244
genus.Alistipes.id.968	rs11958296	5	G	A	-0.098122204	0.021827201	9.29574E-06	16214	0.001244822	20.20870515
genus.Alistipes.id.968	rs8130320	21	G	A	-0.049001972	0.01071701	4.83612E-06	17571	0.001188412	20.90642736
genus.Alistipes.id.968	rs2875322	11	C	T	-0.058094464	0.013143238	8.77703E-06	17459	0.001117789	19.53730859
genus.Alistipes.id.968	rs2290844	10	T	C	0.081396908	0.019163185	9.10135E-06	17571	0.001025742	18.04182409
genus.Alistipes.id.968	rs35909684	8	C	A	-0.084528462	0.019209072	7.6466E-06	17457	0.001108006	19.36391464
genus.Alistipes.id.968	rs2450745	8	C	A	-0.080523959	0.018470178	7.11836E-06	17571	0.001080543	19.00675941
genus.Alistipes.id.968	rs62576416	9	C	T	0.049264678	0.010973282	7.50368E-06	17564	0.001146243	20.15571525
genus.Alistipes.id.968	rs1107244	13	A	G	0.075855776	0.017118221	3.59059E-06	17571	0.001116294	19.63631716
genus.Alistipes.id.968	rs34417064	17	G	A	-0.04820195	0.010686843	7.00749E-06	17570	0.001156528	20.34371862
genus.Alistipes.id.968	rs12990744	2	T	C	-0.077661791	0.017298561	8.20725E-06	16694	0.001205898	20.15555906
genus.Alistipes.id.968	rs6563550	13	C	T	0.075255827	0.017126002	3.96747E-06	17571	0.001097728	19.30937844
genus.Alistipes.id.968	rs4810359	20	G	A	-0.065242793	0.014615527	7.49669E-06	17380	0.001145219	19.92673493
genus.Allisonella.id.2174	rs602075	9	G	A	0.168974277	0.029697566	3.56575E-08	3212	0.009978575	32.37423322
genus.Allisonella.id.2174	rs35778461	9	T	C	0.146678707	0.029718557	1.2063E-06	3212	0.007526999	24.36008036
genus.Allisonella.id.2174	rs6742198	2	A	G	0.149151971	0.031648438	3.34514E-06	3182	0.006931583	22.21024871
genus.Allisonella.id.2174	rs1901739	5	G	T	0.115768513	0.024862618	3.58801E-06	3212	0.006704864	21.6813936
genus.Allisonella.id.2174	rs76904847	7	A	G	0.148523492	0.033485691	6.08751E-06	3182	0.006144617	19.67305363
genus.Allisonella.id.2174	rs35110698	12	C	T	-0.146321972	0.032085207	5.72322E-06	3212	0.006433257	20.79741683
genus.Allisonella.id.2174	rs7898615	10	G	T	0.167966358	0.037358631	8.87277E-06	3212	0.006254067	20.21448601
genus.Allisonella.id.2174	rs594561	11	T	C	0.112230607	0.025168069	9.41103E-06	3212	0.006152717	19.88487286
genus.Allisonella.id.2174	rs685403	18	C	G	-0.175203225	0.040447416	4.88394E-06	3212	0.005807611	18.76301559
genus.Alloprevotella.id.961	rs12675596	8	T	G	0.145668934	0.029067823	9.63726E-07	3008	0.008279806	25.11359343
genus.Alloprevotella.id.961	rs58212166	4	G	A	-0.161505876	0.035910233	7.93614E-06	3005	0.006686243	20.22740704
genus.Alloprevotella.id.961	rs34619204	21	A	G	-0.156036945	0.034439247	8.8419E-06	3003	0.006789434	20.52804484
genus.Alloprevotella.id.961	rs17380632	3	T	A	0.125563764	0.028040851	9.27121E-06	3009	0.00661972	20.0514728
genus.Alloprevotella.id.961	rs4680035	3	G	A	-0.119599445	0.025944593	4.99052E-06	3007	0.007017345	21.250277
genus.Alloprevotella.id.961	rs4364940	1	G	A	0.126417748	0.028220735	8.58353E-06	3008	0.006626954	20.06686119
genus.Alloprevotella.id.961	rs2154444	21	G	T	0.138234323	0.030896071	8.36755E-06	3009	0.006608812	20.01821139
genus.Anaerofilum.id.2053	rs816292	12	C	T	-0.113002639	0.022040046	2.64038E-07	5017	0.005212409	26.28767594
genus.Anaerofilum.id.2053	rs79598899	2	T	C	0.182585804	0.035728447	3.74728E-07	4709	0.005515376	26.11594473
genus.Anaerofilum.id.2053	rs4506496	1	A	G	0.103089882	0.021286122	1.4866E-06	4935	0.004730343	23.45519224
genus.Anaerofilum.id.2053	rs712981	3	C	A	0.100759617	0.020290248	6.83115E-07	5051	0.00485854	24.66029793
genus.Anaerofilum.id.2053	rs17105491	10	C	G	-0.19310744	0.04111866	1.56819E-06	4856	0.004521404	22.05566216
genus.Anaerofilum.id.2053	rs10794359	11	C	T	-0.095352431	0.020059979	2.23496E-06	4994	0.00450395	22.59449311
genus.Anaerofilum.id.2053	rs17096874	14	T	C	-0.126336895	0.02689189	2.8603E-06	5051	0.004350577	22.07078303
genus.Anaerofilum.id.2053	rs356049	17	A	G	0.132602907	0.028980209	6.55844E-06	4994	0.004174819	20.93645164
genus.Anaerofilum.id.2053	rs1563175	2	C	A	0.092383381	0.020215708	5.53848E-06	5051	0.004117565	20.88381284
genus.Anaerofilum.id.2053	rs17012738	2	G	T	0.090341716	0.020023943	7.24168E-06	5051	0.004013779	20.35529886
genus.Anaerofilum.id.2053	rs9299345	9	C	T	-0.136399548	0.030237163	8.04309E-06	5051	0.004012548	20.34903268
genus.Anaerofilum.id.2053	rs4244069	12	A	G	-0.146774518	0.032659424	9.81202E-06	5030	0.003999226	20.19687992
genus.Anaerostipes.id.1991	rs7193624	16	T	C	0.075064239	0.015072346	5.35482E-07	16926	0.001463235	24.80301381
genus.Anaerostipes.id.1991	rs2804244	10	G	A	-0.053088986	0.011098265	2.04051E-06	16926	0.001350074	22.88224882
genus.Anaerostipes.id.1991	rs62215703	21	A	G	0.064496549	0.013670195	1.9777E-06	15550	0.00142946	22.25991606
genus.Anaerostipes.id.1991	rs2396460	2	C	T	-0.051265275	0.010955542	2.91088E-06	16912	0.00129307	21.89670679
genus.Anaerostipes.id.1991	rs3900776	9	A	G	-0.1100132	0.023630171	2.74765E-06	14694	0.001472908	21.67483861
genus.Anaerostipes.id.1991	rs2014785	3	C	T	0.051568685	0.011219863	4.68061E-06	16445	0.001282938	21.12501493
genus.Anaerostipes.id.1991	rs62157625	2	C	T	0.088573041	0.018554786	1.44909E-06	15215	0.001495441	22.78721366
genus.Anaerostipes.id.1991	rs6726833	2	A	C	-0.087749727	0.018940832	3.32217E-06	16924	0.001266601	21.46314841
genus.Anaerostipes.id.1991	rs6474958	9	G	A	-0.050006507	0.011200088	6.74483E-06	16918	0.001176928	19.93472288
genus.Anaerostipes.id.1991	rs6854026	4	C	T	-0.050848158	0.010907505	3.19578E-06	16921	0.001282674	21.73199507
genus.Anaerostipes.id.1991	rs78735375	19	C	A	-0.13742857	0.030530436	5.33384E-06	10360	0.001952	20.26226681
genus.Anaerostipes.id.1991	rs7823228	8	C	G	-0.062108471	0.013661403	5.84203E-06	16445	0.001255254	20.66860134
genus.Anaerostipes.id.1991	rs13376554	1	T	A	0.19738899	0.04586424	7.72059E-06	3101	0.005937578	18.52240837
genus.Anaerostipes.id.1991	rs60983350	17	A	G	-0.053990065	0.011657526	4.42054E-06	16344	0.001310651	21.44938683
genus.Anaerostipes.id.1991	rs10502061	11	G	A	0.083575226	0.019201667	7.93539E-06	16825	0.001124692	18.94424203
genus.Anaerotruncus.id.2054	rs6563550	13	C	T	0.087713486	0.017674473	2.34517E-07	16566	0.001484489	24.62860322
genus.Anaerotruncus.id.2054	rs1107244	13	A	G	0.086158653	0.017666099	3.65691E-07	16566	0.001433757	23.78572967
genus.Anaerotruncus.id.2054	rs8005030	14	T	C	0.055444659	0.01178543	2.28298E-06	16548	0.00133568	22.13240242
genus.Anaerotruncus.id.2054	rs9603208	13	T	G	0.077551909	0.01652548	1.57451E-06	16566	0.001327646	22.0230169
genus.Anaerotruncus.id.2054	rs4669806	2	T	G	0.057638903	0.012299403	2.4206E-06	16566	0.001323946	21.96156744

genus.Anaerotruncus.id.2054	rs11841382	13	T	G	0.084158037	0.018542231	2.4501E-06	16566	0.001241966	20.59999991
genus.Anaerotruncus.id.2054	rs10150232	14	G	A	0.056708789	0.012487702	6.67667E-06	16117	0.001277898	20.62223461
genus.Anaerotruncus.id.2054	rs1272208	9	T	G	-0.061174271	0.012983073	4.27982E-06	16117	0.001375627	22.20151445
genus.Anaerotruncus.id.2054	rs12056802	8	G	C	0.077419353	0.017717789	6.12679E-06	16010	0.001191163	19.09325748
genus.Anaerotruncus.id.2054	rs11018566	11	G	A	-0.156465176	0.036603226	6.13709E-06	6278	0.002902105	18.27244509
genus.Anaerotruncus.id.2054	rs7675045	4	T	A	-0.049781529	0.01105734	6.91883E-06	16566	0.001222043	20.2691326
genus.Anaerotruncus.id.2054	rs1431492	3	T	C	-0.06549964	0.014618811	7.35565E-06	16560	0.001210786	20.07491655
genus.Anaerotruncus.id.2054	rs9347879	6	C	T	0.050618001	0.011049001	4.22333E-06	16566	0.001265309	20.98765746
genus.Anaerotruncus.id.2054	rs17734739	2	C	T	0.066005195	0.014908026	7.42772E-06	15927	0.001229271	19.602704
genus.Anaerotruncus.id.2054	rs6494922	15	G	A	0.090310638	0.020225652	6.62362E-06	16067	0.001239365	19.93759416
genus.Anaerotruncus.id.2054	rs34449434	12	C	A	-0.049700388	0.011340216	9.85065E-06	16117	0.001190352	19.20776823
genus.Anaerotruncus.id.2054	rs7155595	14	A	C	0.053933621	0.011890292	7.54982E-06	16553	0.001241418	20.57472984
genus.Anaerotruncus.id.2054	rs115414803	4	C	A	-0.144355888	0.031752395	6.83473E-06	9273	0.00222397	20.66883624
genus.Anaerotruncus.id.2054	rs2704155	11	A	T	-0.106234694	0.023481456	5.4183E-06	12419	0.001645436	20.46834349
genus.Bacteroides.id.918	rs6795673	3	T	C	0.053856525	0.010525127	3.37909E-07	18301	0.001428651	26.18315417
genus.Bacteroides.id.918	rs28757219	6	A	T	0.081839827	0.017031866	1.2942E-06	14833	0.001554177	23.08898982
genus.Bacteroides.id.918	rs9507307	13	T	C	0.060445621	0.012912793	2.1271E-06	18184	0.001203585	21.91236741
genus.Bacteroides.id.918	rs66474973	20	T	G	0.081251539	0.016447899	6.80966E-07	17405	0.001400102	24.40294961
genus.Bacteroides.id.918	rs11585893	1	G	A	-0.074074561	0.014763325	1.79511E-06	17821	0.001410668	25.17501972
genus.Bacteroides.id.918	rs495004	9	G	C	-0.060747491	0.012986536	3.42092E-06	17707	0.001234209	21.88114365
genus.Bacteroides.id.918	rs17619981	19	G	T	0.088097761	0.018700207	2.68683E-06	15801	0.001402629	22.19406888
genus.Bacteroides.id.918	rs2023437	14	C	T	-0.07823245	0.016763399	5.01824E-06	17484	0.001244137	21.77958883
genus.Bacteroides.id.918	rs66710942	3	T	C	0.048803777	0.010741209	5.86259E-06	17821	0.001157086	20.64431778
genus.Bacteroides.id.918	rs13207588	6	G	A	-0.059204982	0.013119468	7.48504E-06	17707	0.001148788	20.36498267
genus.Bacteroides.id.918	rs2366421	3	A	T	-0.05281527	0.011712437	7.64945E-06	18299	0.001109979	20.33407866
genus.Bacteroides.id.918	rs1340391	1	C	T	-0.059200382	0.013224416	6.7301E-06	18302	0.00109376	20.03992112
genus.Barnesiella.id.944	rs2276875	4	G	A	-0.069717565	0.013953704	4.65027E-07	13893	0.001793617	24.96349607
genus.Barnesiella.id.944	rs2428166	6	A	G	-0.165858717	0.033730907	8.50874E-07	8091	0.002979357	24.17801073
genus.Barnesiella.id.944	rs60316894	12	T	C	-0.121478156	0.025183972	1.19064E-06	12717	0.001826288	23.26740298
genus.Barnesiella.id.944	rs35177866	3	G	A	0.091712545	0.019013343	2.94763E-06	13394	0.001734109	23.26700646
genus.Barnesiella.id.944	rs13242616	7	C	T	-0.058374651	0.012317572	2.29017E-06	13945	0.001607981	22.45941214
genus.Barnesiella.id.944	rs79795328	4	G	A	-0.081864467	0.01764407	4.23302E-06	13657	0.001573816	21.52748773
genus.Barnesiella.id.944	rs2057922	7	C	G	0.091524071	0.019462277	3.82634E-06	14208	0.001554086	22.11481713
genus.Barnesiella.id.944	rs199035	6	A	G	0.055949483	0.011971558	3.00294E-06	13945	0.001563841	21.84191822
genus.Barnesiella.id.944	rs62251337	3	G	A	-0.069076268	0.014937412	4.24161E-06	13945	0.001531169	21.38488951
genus.Barnesiella.id.944	rs77455852	5	G	T	-0.089149084	0.019560057	3.16354E-06	13394	0.001548496	20.77272762
genus.Barnesiella.id.944	rs11155559	6	C	T	0.095602282	0.021296077	8.91914E-06	13383	0.001503593	20.15288827
genus.Barnesiella.id.944	rs12909713	15	T	C	-0.055071556	0.012003894	4.94527E-06	14205	0.001479538	21.04797763
genus.Barnesiella.id.944	rs113258194	2	G	A	0.098992031	0.021412093	7.31119E-06	13945	0.001530378	21.37382445
genus.Barnesiella.id.944	rs28479800	1	T	A	-0.091520023	0.021000228	7.93418E-06	12399	0.001529441	18.99259094
genus.Barnesiella.id.944	rs76181748	8	T	C	-0.077855934	0.017163996	6.7766E-06	14201	0.001446768	20.57532026
genus.Barnesiella.id.944	rs72684847	4	C	T	-0.114369278	0.025387327	6.76169E-06	12984	0.001560623	20.29480125
genus.Barnesiella.id.944	rs28418786	8	G	C	-0.079132421	0.017495701	6.47627E-06	13698	0.001491217	20.45720102
genus.Bifidobacterium.id.436	rs182549	2	T	C	0.119703495	0.012729374	1.27822E-20	14778	0.0059483	88.42998905
genus.Bifidobacterium.id.436	rs7570971	2	C	A	0.113335164	0.012574753	2.01343E-19	14666	0.005508329	81.23260879
genus.Bifidobacterium.id.436	rs1530559	2	A	G	0.07578753	0.012024529	2.10779E-10	14666	0.002701301	39.72458389
genus.Bifidobacterium.id.436	rs7322849	13	C	T	0.112428453	0.020181262	1.08368E-08	14778	0.0020957	31.03529117
genus.Bifidobacterium.id.436	rs10841473	12	C	G	-0.062420654	0.012943849	1.64522E-06	14777	0.001571305	23.25571624
genus.Bifidobacterium.id.436	rs56108664	5	C	T	0.072997098	0.015792589	2.4402E-06	13916	0.001532935	21.3650803
genus.Bifidobacterium.id.436	rs2686790	7	C	T	0.070741011	0.015792595	7.49893E-06	14778	0.001355909	20.06483284
genus.Bifidobacterium.id.436	rs4567981	2	A	T	0.056208395	0.011792309	1.92831E-06	14666	0.001546752	22.71980905
genus.Bifidobacterium.id.436	rs2491158	10	A	G	0.071262413	0.015983004	8.04716E-06	14021	0.001415826	19.87944289
genus.Bifidobacterium.id.436	rs76671854	5	G	C	-0.084605457	0.01840032	3.9567E-06	13806	0.001529022	21.14199753
genus.Bifidobacterium.id.436	rs75344046	21	T	C	0.232354091	0.050597867	4.86354E-06	3856	0.005439144	21.0880404
genus.Bifidobacterium.id.436	rs540489	17	G	T	-0.063764086	0.013874591	5.19458E-06	14561	0.001448409	21.12087287
genus.Bifidobacterium.id.436	rs857444	6	T	C	0.055823437	0.012121924	3.57097E-06	14777	0.001433114	21.20752575
genus.Bifidobacterium.id.436	rs13020688	2	A	G	0.056269631	0.012261748	4.07258E-06	14666	0.001433866	21.05927361
genus.Bifidobacterium.id.436	rs12022129	1	A	G	0.061935632	0.013893747	7.99643E-06	14776	0.00134308	19.87203725
genus.Bifidobacterium.id.436	rs62181700	2	A	G	-0.062464331	0.013120526	2.17246E-06	14774	0.001531785	22.66531171
genus.Bifidobacterium.id.436	rs55888705	4	G	A	0.054631934	0.012113865	6.67019E-06	14777	0.001374498	20.33891191
genus.Bifidobacterium.id.436	rs5746486	22	C	T	-0.053621605	0.012080057	8.99955E-06	14778	0.001331519	19.70342231
genus.Bifidobacterium.id.436	rs73797465	5	G	T	-0.095356633	0.02092365	4.38162E-06	14666	0.001414167	20.7695413
genus.Bifidobacterium.id.436	rs4957061	5	C	T	0.053423938	0.011743107	5.77933E-06	14666	0.001409229	20.69692062
genus.Bilophila.id.3170	rs116261629	6	C	G	0.128112276	0.026135828	8.62329E-07	11276	0.002126324	24.02752055
genus.Bilophila.id.3170	rs1571225	9	T	C	0.082682569	0.017062147	1.12289E-06	12854	0.0018236	23.48337867
genus.Bilophila.id.3170	rs3827020	20	T	C	0.076647909	0.016064223	1.78875E-06	12628	0.001799552	22.76570977
genus.Bilophila.id.3170	rs1241171	1	A	G	-0.069265389	0.015014825	4.24037E-06	12856	0.0016526	21.28099863
genus.Bilophila.id.3170	rs7802841	7	A	C	0.067009659	0.013770056	1.76952E-06	12856	0.001838647	23.68118389
genus.Bilophila.id.3170	rs8013541	14	T	A	-0.05719913	0.01256729	5.47538E-06	12856	0.001608757	20.71550674
genus.Bilophila.id.3170	rs6793291	3	A	C	0.112729046	0.024163294	3.1132E-06	11051	0.001965637	21.76503632
genus.Bilophila.id.3170	rs1917709	7	T	A	0.118509423	0.02674196	7.36957E-06	10886	0.001800811	19.63899671
genus.Bilophila.id.3170	rs542415	15	C	T	-0.061362319	0.013343594	4.70948E-06	12777	0.001652383	21.14744576
genus.Bilophila.id.3170	rs72676854	8	C	T	0.123208756	0.026882038	5.62268E-06	11616	0.001805167	21.00674487
genus.Bilophila.id.3170	rs4798126	18	A	G	0.073288019	0.016798984	7.14662E-06	12777	0.001487389	19.03267997
genus.Bilophila.id.3170	rs1969927	12	A	G	0.056458855	0.012693476	9.0722E-06	12777	0.001545974	19.78349494
genus.Bilophila.id.3170	rs60178956	8	A	G	-0.062481785	0.014148499	8.05619E-06	12777	0.001524035	19.5023133
genus.Bilophila.id.3170	rs2728491	7	T	G	-0.062741568	0.013945646	6.33255E-06	12856	0.00157197	20.24106895
genus.Bilophila.id.3170	rs2713349	7	T	A	0.061722789	0.014017424	8.63249E-06	12853	0.001506245	19.38896577
genus.Bilophila.id.3170	rs9899990	17	G	A	-0.102702281	0.023387821	9.06691E-06	12088	0.0015927	19.28326835
genus.Bilophila.id.3170	rs11069458	13	C	T	-0.068094659	0.015502996	7.71547E-06	12856	0.001498434	19.29278009
genus.Blautia.id.1992	rs11149971	16	T	C	0.117604747	0.023389975	1.03934E-06	16255	0.001552846	25.28076427
genus.Blautia.id.1992	rs12453000	17	T	C	0.062533456	0.012998711	1.26117E-06	17815	0.001297402	23.14324521
genus.Blautia.id.1992	rs115043014	1	A	G	-0.206605438	0.043991974	5.186E-06	4902	0.004479335	22.05650016
genus.Blautia.id.1992	rs67794373	7	T	C	0.060170889	0.012344281	1.00003E-06	18158	0.001306789	23.75971817
genus.Blautia.id.1992	rs117001700	7	C	T	0.196414252	0.044112197	8.83912E-06	4965	0.003977211	19.82570418
genus.Blautia.id.1992	rs72973581	19	G	A	0.125169075	0.026540793	1.73934E-06	12764	0.001739496	22.2416137
genus.Blautia.id.1992	rs4926264	19	C	T	0.082620681	0.017823907	5.10293E-06	18276	0.001174303	21.48679965
genus.Blautia.id.1992	rs7860714	9	G	A	-0.050217775	0.010985269	4.09147E-06	18162	0.001149292	20.89746167
genus.Blautia.id.1992	rs682885	11	G	A	-0.049337611	0.010735883	4.4908E-06	18274	0.001154371	21.1193554
genus.Blautia.id.1992	rs2788271	1	G	T	-0.057559633	0.013349169	7.16388E-06	18276	0.00101626	18.59206321
genus.Blautia.id.1992	rs113271346	14	T	C	0.078268222	0.017211647	6.85386E-06	17465	0.001182616	20.67883649
genus.Blautia.id.1992	rs3005511	6	G	A	0.050108496	0.01107676	6.18905E-06	18276	0.001118485	20.46431531
genus.Blautia.id.1992	rs16892041	6	C	T	-0.062260496	0.014180398	8.82028E-06	18276	0.001053681	19.27739374
genus.Butyricicoccus.id.2055	rs56221232	2	C	T	0.082802732	0.016740066	7.6194E-07	17133	0.001426008	24.46667892
genus.Butyricicoccus.id.2055	rs2017189	4	T	G	-0.050695599	0.011024022	3.87256E-06	17133	0.001232796	21.14756447
genus.Butyricicoccus.id.2055	rs7322368	13	C	T	0.081573314	0.018316651	5.5178E-06	17134	0.001156226	19.83371584
genus.Butyricicoccus.id.2055	rs75238760	18	A	T	0.061942289	0.013994184	6.797E-06	17022	0.001149659	19.59202475
genus.Butyricicoccus.id.2055	rs12585793	13	C	T	-0.262206199	0.056472877	5.79191E-06	3135	0.006829554	21.55788139
genus.Butyricicoccus.id.2055	rs62478070	7	G	T	0.224039318	0.049495932	5.9377E-06	4007	0.005087157	20.48846722
genus.Butyricicoccus.id.2055	rs4962426	10	T	G	0.061421574	0.013597912	7.3849E-06	16889	0.001206616	20.40315885
genus.Butyricicoccus.id.2055	rs10084203	2	G	A	0.054969862	0.012356262	8.58646E-06	17135	0.001153691	19.79133555
genus.Butyricicoccus.id.2055	rs12034718	1	G	A	0.0701199	0.01582135	9.57686E-06	16237	0.001208272	19.64244362
genus.Butyricimonas.id.945	rs113054641	21	A	G	-0.144880972	0.027457379	1.73694E-07	9949	0.002790689	27.84226396
genus.Butyricimonas.id.945	rs62390301	5	C	T	-0.087298337	0.017489288	7.41905E-07	10634	0.002337516	24.91538826
genus.Butyricimonas.id.945	rs7083431	10	C	A	0.070353941	0.014443868	8.8546E-07	10737	0.002204795	23.72519668
genus.Butyricimonas.id.945	rs2642760	5	C	G	0.071335713	0.014539571	8.5808E-07	10634	0.002258563	24.07192393
genus.Butyricimonas.id.945	rs782080	3	A	T	0.065348802	0.01379735	2.23398E-06	10733	0.00208572	22.43282154
genus.Butyricimonas.id.945	rs1862649	16	A	G	0.113123952	0.024817639	4.76256E-06	10123	0.002048276	20.77725761
genus.Butyricimonas.id.945	rs12304031	12	A	G	-0.086278048	0.019684629	6.70067E-06	10658	0.001799237	19.21083069
genus.Butyricimonas.id.945	rs71428626	2	T	G	-0.133188396	0.028990548	4.80413E-06	9776	0.002154379	21.10668039
genus.Butyricimonas.id.945	rs78453362	3	G	A	-0.14948019	0.032707281	4.0581E-06	8207	0.002538575	20.88711256
genus.Butyricimonas.id.945	rs326049	5	G	C	0.075969365	0.01676173	8.1865E-06	10557	0.001942024	20.54183981
genus.Butyricimonas.id.945	rs62130338	19	A	G	-0.073260812	0.015832388	3.8981E-06	10557	0.002024091	21.4116717
genus.Butyricimonas.id.945	rs9657374	8	T	C	0.068071943	0.014817248	4.50359E-06	10737	0.001961848	21.10577054
genus.Butyricimonas.id.945	rs12458763	18	C	A	0.122036628	0.026958664	6.36646E-06	10383	0.00196972	20.49196777
genus.Butyricimonas.id.945	rs2114713	15	T	G	0.062720962	0.013899768	6.87713E-06	10734	0.001893327	20.3615251
genus.Butyricimonas.id.945	rs270727	1	C	G	-0.069417267	0.015079545	5.38157E-06	10634	0.001988828	21.19134573
genus.Butyricimonas.id.945	rs72814525	10	G	A	0.066415351	0.01497662	8.24599E-06	10736	0.001828403	19.66569637
genus.Butyricimonas.id.945	rs11228830	11	G	A	0.135264872	0.029841132	6.55162E-06	8974	0.002284337	20.54657676
genus.Butyrivibrio.id.1993	rs72723662	14	T	C	0.224078206	0.044933642	7.86241E-07	3073	0.008027734	24.86886693
genus.Butyrivibrio.id.1993	rs7412979	1	G	C	0.186778577	0.038911205	1.70567E-06	3480	0.00657748	23.04118314
genus.Butyrivibrio.id.1993	rs7752361	6	G	A	-0.119230279	0.023997488	7.69113E-07	3481	0.007041556	24.68547887
genus.Butyrivibrio.id.1993	rs4537857	13	C	T	-0.124589674	0.026099163	1.80192E-06	3482	0.006502033	22.78824815
genus.Butyrivibrio.id.1993	rs11761679	7	C	T	0.154775363	0.032153939	2.20231E-06	3482	0.006610376	23.17049494
genus.Butyrivibrio.id.1993	rs16941336	17	T	C	0.127487508	0.026793591	1.53182E-06	3482	0.006459963	22.63984305
genus.Butyrivibrio.id.1993	rs77356209	18	C	T	0.216874487	0.04832994	6.6643E-06	3379	0.00592401	20.13651974
genus.Butyrivibrio.id.1993	rs74622183	14	G	A	-0.201027447	0.042820448	2.45748E-06	3478	0.006297024	22.03983323
genus.Butyrivibrio.id.1993	rs7763512	6	A	G	0.119845684	0.025336711	3.10655E-06	3259	0.006818496	22.3740355
genus.Butyrivibrio.id.1993	rs142855850	10	G	A	0.205013198	0.045713757	6.85543E-06	3234	0.006180694	20.11267374

genus.Butyrivibrio.id.1993	rs9349693	6	G	A	0.117972728	0.025986063	5.54958E-06	3482	0.005884241	20.61020333
genus.Butyrivibrio.id.1993	rs486484	20	G	A	-0.108348689	0.024000793	6.61084E-06	3456	0.005862312	20.37962332
genus.Butyrivibrio.id.1993	rs17163238	5	A	G	0.140986912	0.030911712	5.51196E-06	3482	0.005938761	20.80230573
genus.Butyrivibrio.id.1993	rs4928024	3	G	A	-0.174709994	0.038933257	8.18641E-06	3481	0.00575155	20.1369654
genus.Butyrivibrio.id.1993	rs1007475	6	T	G	0.118065426	0.026127086	7.92312E-06	3482	0.005830359	20.42036684
genus.Butyrivibrio.id.1993	rs16934069	9	C	T	-0.133784151	0.029944364	8.8591E-06	3482	0.005699908	19.96085478
genus.CandidatusSoleaferrea.id.113	rs4294381	1	C	T	0.112196526	0.023186007	1.3685E-06	6145	0.003796062	23.41568622
genus.CandidatusSoleaferrea.id.113	rs10090365	8	G	A	-0.083441348	0.018098882	4.17309E-06	6145	0.003446971	21.25490248
genus.CandidatusSoleaferrea.id.113	rs4678258	3	C	T	0.098613616	0.021564627	5.53183E-06	6101	0.003415882	20.9117257
genus.CandidatusSoleaferrea.id.113	rs10108780	8	G	A	-0.09283404	0.01998066	3.64079E-06	5999	0.003585552	21.58712741
genus.CandidatusSoleaferrea.id.113	rs386526	10	G	C	0.081845856	0.018008617	8.33076E-06	6144	0.00335061	20.65535482
genus.CandidatusSoleaferrea.id.113	rs36155147	7	T	C	0.104966126	0.024099527	5.41138E-06	5958	0.003173951	18.97061014
genus.CandidatusSoleaferrea.id.113	rs6881988	5	C	G	-0.081913114	0.018185036	9.22889E-06	6140	0.003293649	20.28983525
genus.CandidatusSoleaferrea.id.113	rs11153159	6	C	G	-0.128050003	0.028544611	4.41523E-06	6101	0.003287606	20.12384383
genus.CandidatusSoleaferrea.id.113	rs10809135	9	C	T	0.08348579	0.01824263	5.47174E-06	5999	0.003479027	20.9435459
genus.CandidatusSoleaferrea.id.113	rs9973954	2	G	A	0.089207514	0.019540149	5.94796E-06	6145	0.003380296	20.84237154
genus.CandidatusSoleaferrea.id.113	rs6489992	12	G	A	-0.084043749	0.018703073	7.88786E-06	6142	0.003276798	20.19225807
genus.CandidatusSoleaferrea.id.113	rs6494306	15	G	A	-0.09693036	0.021422086	5.80051E-06	6145	0.0033207	20.47368989
genus.CandidatusSoleaferrea.id.113	rs12500231	4	T	A	0.08138533	0.018269552	7.67641E-06	6145	0.003218951	19.84433092
genus.CandidatusSoleaferrea.id.113	rs7400877	14	C	T	-0.095108487	0.021279907	9.29448E-06	6145	0.00324017	19.97556635
genus.CandidatusSoleaferrea.id.113	rs2193878	3	A	T	0.228255107	0.050924135	9.45546E-06	3088	0.00646398	20.09063626
genus.CandidatusSoleaferrea.id.113	rs830149	19	G	C	0.184646616	0.039723748	9.58382E-06	5065	0.004247703	21.60639309
genus.Catenibacterium.id.2153	rs12404911	1	T	C	0.140717216	0.030414222	2.79891E-06	3239	0.006565523	21.40627179
genus.Catenibacterium.id.2153	rs77285108	4	A	G	-0.161826131	0.035344674	3.6268E-06	3210	0.006488098	20.96280205
genus.Catenibacterium.id.2153	rs73128290	7	G	A	0.129726249	0.02845626	4.29372E-06	3239	0.006375459	20.78260958
genus.Catenibacterium.id.2153	rs212393	6	A	G	-0.135253856	0.02862088	3.62489E-06	3239	0.006847598	22.33229385
genus.Catenibacterium.id.2153	rs7742829	6	T	C	0.114110133	0.025109895	5.60729E-06	3239	0.006335595	20.65183446
genus.ChristensenellaceaeR.7group.	rs999354	19	A	T	0.058024779	0.011718514	7.00695E-07	16324	0.001499697	24.51782484
genus.ChristensenellaceaeR.7group.	rs78521377	10	T	C	0.12499178	0.027479689	5.60674E-06	12605	0.001638642	20.68898937
genus.ChristensenellaceaeR.7group.	rs62467127	7	T	C	0.114108279	0.025198272	3.25362E-06	12719	0.001609682	20.50655951
genus.ChristensenellaceaeR.7group.	rs17081797	18	G	A	-0.090432475	0.020425314	3.34031E-06	16324	0.001199399	19.60249544
genus.ChristensenellaceaeR.7group.	rs62132810	19	G	A	-0.082885316	0.017962272	5.66711E-06	15066	0.001411306	21.29279434
genus.ChristensenellaceaeR.7group.	rs60954665	19	G	T	0.049814958	0.011078727	7.13177E-06	16324	0.001237017	20.21807512
genus.ChristensenellaceaeR.7group.	rs4805124	19	G	C	0.049460597	0.011078818	8.26872E-06	16325	0.001219407	19.93112529
genus.ChristensenellaceaeR.7group.	rs10461257	4	G	A	-0.05519694	0.01220692	6.50746E-06	15987	0.001277308	20.44644414
genus.ChristensenellaceaeR.7group.	rs79150079	5	A	C	0.121546581	0.027097732	9.41804E-06	11994	0.001674664	20.11961173
genus.ChristensenellaceaeR.7group.	rs62190261	2	C	A	0.095839425	0.021472282	8.74023E-06	15274	0.001302606	19.92195708
genus.ChristensenellaceaeR.7group.	rs892686	9	G	A	0.051409887	0.011135975	3.96861E-06	16325	0.001303818	21.31261686
genus.ChristensenellaceaeR.7group.	rs73952017	18	T	C	-0.086216444	0.019435393	8.45769E-06	15274	0.001286713	19.67857192
genus.Clostridiumsensustricto1.id.1	rs550843	6	C	T	-0.078324645	0.016920943	2.05071E-06	14102	0.001517077	21.42632678
genus.Clostridiumsensustricto1.id.1	rs2795528	10	A	G	-0.184314695	0.039220419	2.7204E-06	6366	0.003457202	22.08490143
genus.Clostridiumsensustricto1.id.1	rs2817172	1	T	C	0.058138957	0.012449219	2.76655E-06	13658	0.001594301	21.80972809
genus.Clostridiumsensustricto1.id.1	rs115807074	5	G	A	-0.227359786	0.049257523	4.31688E-06	4229	0.005012589	21.30503041
genus.Clostridiumsensustricto1.id.1	rs116847295	12	T	C	0.110021418	0.024603155	4.58118E-06	12910	0.001546587	19.9973692
genus.Clostridiumsensustricto1.id.1	rs12341505	9	A	G	0.081071775	0.018012111	4.82174E-06	14118	0.001432894	20.25863229
genus.Clostridiumsensustricto1.id.1	rs11586026	1	T	A	0.110966819	0.025049788	8.84988E-06	13095	0.001496313	19.62357654
genus.Clostridiumsensustricto1.id.1	rs11264403	1	A	G	-0.139061503	0.033445425	7.75544E-06	7285	0.002367454	17.28782706
genus.Clostridiumsensustricto1.id.1	rs12490337	3	G	C	-0.061687867	0.0137581	7.49465E-06	14121	0.001421671	20.10400411
genus.Collinsella.id.815	rs9541268	13	A	C	0.09596998	0.019731218	8.79419E-07	12914	0.001828552	23.65718275
genus.Collinsella.id.815	rs2671662	17	G	C	-0.056775091	0.011944997	2.2204E-06	14334	0.001573592	22.59142252
genus.Collinsella.id.815	rs73052258	2	A	G	0.093017814	0.020265842	1.7158E-06	14334	0.001467566	21.06701275
genus.Collinsella.id.815	rs2103510	21	A	G	0.078655101	0.016821672	2.42474E-06	14334	0.001522952	21.86328649
genus.Collinsella.id.815	rs75672793	4	G	A	-0.108908003	0.024052154	6.13549E-06	12601	0.001624428	20.50272724
genus.Collinsella.id.815	rs10890671	11	C	T	-0.053740144	0.011884842	6.52136E-06	14334	0.001424375	20.44611466
genus.Collinsella.id.815	rs12921100	16	T	A	0.056375862	0.012701892	8.2257E-06	14334	0.001372416	19.69924539
genus.Collinsella.id.815	rs59414781	15	G	C	0.066886563	0.014977291	9.14723E-06	14334	0.001389441	19.9439516
genus.Collinsella.id.815	rs62448871	7	A	C	-0.054030922	0.012032414	6.78253E-06	14334	0.001404757	20.16411941
genus.Collinsella.id.815	rs62448869	7	A	T	-0.054066284	0.01203245	6.81669E-06	14334	0.001406586	20.19039811
genus.Collinsella.id.815	rs1496626	19	C	T	-0.072230674	0.016160046	6.78175E-06	14334	0.001391829	19.97828316
genus.Collinsella.id.815	rs149807560	19	A	C	-0.104264209	0.023584097	7.0987E-06	13473	0.001448565	19.5448346
genus.Collinsella.id.815	rs11597285	10	T	G	-0.053794651	0.012054849	9.37615E-06	14334	0.001387345	19.91382379
genus.Coprobacter.id.949	rs305411	1	G	A	0.129218483	0.026451784	1.00906E-06	6777	0.003508934	23.86378554
genus.Coprobacter.id.949	rs3828477	3	T	G	-0.091217951	0.019569041	2.88974E-06	6798	0.003186064	21.7280901
genus.Coprobacter.id.949	rs55672356	4	A	T	-0.193354203	0.041381488	2.7369E-06	4903	0.00443306	21.83207551
genus.Coprobacter.id.949	rs143662916	11	T	C	0.25326389	0.05401412	3.07252E-06	3083	0.007080638	21.98527673
genus.Coprobacter.id.949	rs213863	6	T	C	-0.088741189	0.018837727	2.34883E-06	6798	0.003253845	22.19184949
genus.Coprobacter.id.949	rs72821405	6	C	T	-0.147365313	0.031997805	4.7576E-06	6177	0.00342203	21.21046362
genus.Coprobacter.id.949	rs11532348	12	T	C	-0.103930603	0.022682638	5.71184E-06	6364	0.003288056	20.99422117
genus.Coprobacter.id.949	rs12996055	2	C	A	0.092181727	0.020940499	8.07639E-06	6570	0.002940839	19.37829787
genus.Coprobacter.id.949	rs12684609	9	C	T	0.100826957	0.022036643	6.10177E-06	6364	0.003278733	20.93449343
genus.Coprobacter.id.949	rs5011652	15	C	G	0.089959749	0.02000097	5.5105E-06	6783	0.002973577	20.229929
genus.Coprobacter.id.949	rs74919520	2	A	G	0.125702917	0.02762957	5.76326E-06	6387	0.003230282	20.69867114
genus.Coprobacter.id.949	rs28402691	4	C	T	0.110695393	0.025119913	9.55668E-06	6387	0.003031151	19.41882021
genus.Coprobacter.id.949	rs189356	20	A	G	0.078112515	0.017191637	6.26291E-06	6763	0.003043292	20.64461371
genus.Coprobacter.id.949	rs76001613	4	G	C	0.215910371	0.049301859	9.12599E-06	3385	0.005633886	19.1787556
genus.Coprococcus1.id.11301	rs4277593	20	A	G	-0.058564915	0.010991363	1.13969E-07	16651	0.001702126	28.39042757
genus.Coprococcus1.id.11301	rs56405618	4	G	A	-0.089630001	0.018650815	1.57195E-06	16379	0.00140803	23.0946381
genus.Coprococcus1.id.11301	rs74101919	1	C	T	-0.071898851	0.014465115	1.03291E-06	16732	0.001474387	24.70586846
genus.Coprococcus1.id.11301	rs1010560	1	A	C	0.058023212	0.012272151	1.95915E-06	16723	0.001334959	22.35435396
genus.Coprococcus1.id.11301	rs73031725	11	C	T	0.167590996	0.035522381	1.97917E-06	7206	0.003079382	22.25856838
genus.Coprococcus1.id.11301	rs1519491	2	C	T	0.049919694	0.01135578	8.95283E-06	16733	0.001153546	19.32457296
genus.Coprococcus1.id.11301	rs1576241	6	G	A	-0.051034349	0.010953456	3.32862E-06	16729	0.001295954	21.7081515
genus.Coprococcus1.id.11301	rs73167075	3	C	T	0.057313703	0.01275731	8.56864E-06	16733	0.001204762	20.18360527
genus.Coprococcus1.id.11301	rs12794898	11	T	G	0.090329854	0.019721215	4.91531E-06	15186	0.001379597	20.97950671
genus.Coprococcus1.id.11301	rs946513	10	T	C	0.205898923	0.046001796	8.61855E-06	4310	0.004626657	20.03358045
genus.Coprococcus1.id.11301	rs1762123	6	T	C	-0.089153231	0.019855409	8.01468E-06	16724	0.001204074	20.16120443
genus.Coprococcus1.id.11301	rs7784490	7	G	C	-0.051854255	0.011313593	4.55233E-06	16733	0.00125386	21.00717711
genus.Coprococcus1.id.11301	rs12886051	14	C	G	-0.052178208	0.011805063	8.00779E-06	16633	0.001173171	19.53627301
genus.Coprococcus1.id.11301	rs2907920	19	G	A	0.056117333	0.012682384	7.65327E-06	16040	0.001219153	19.579078
genus.Coprococcus2.id.11302	rs59936925	20	T	A	0.117085317	0.023399473	9.37545E-07	10931	0.002285279	25.03760046
genus.Coprococcus2.id.11302	rs6677933	1	T	C	-0.080440499	0.016421626	1.18501E-06	11483	0.002085236	23.99480237
genus.Coprococcus2.id.11302	rs1958519	14	A	T	0.066628846	0.013855806	1.57692E-06	11009	0.002089777	23.05453792
genus.Coprococcus2.id.11302	rs72680320	4	C	T	-0.064938201	0.013918999	2.27115E-06	11009	0.001973235	21.76629339
genus.Coprococcus2.id.11302	rs2482516	9	T	C	0.075441464	0.016461865	4.72333E-06	11483	0.001825632	21.00207166
genus.Coprococcus2.id.11302	rs35890118	10	G	A	-0.066536003	0.014766106	8.25962E-06	11478	0.001765825	20.30399337
genus.Coprococcus2.id.11302	rs9426473	1	G	A	0.072736094	0.016166159	6.30687E-06	11486	0.001759352	20.24353151
genus.Coprococcus2.id.11302	rs12634070	3	C	T	0.073648589	0.016492134	9.95104E-06	11457	0.001737597	19.94229535
genus.Coprococcus2.id.11302	rs61823518	1	C	A	-0.095538071	0.021572431	6.68258E-06	10980	0.001783107	19.61348362
genus.Coprococcus2.id.11302	rs10070053	5	G	A	0.059431172	0.01353752	7.64883E-06	11473	0.001677044	19.27304594
genus.Coprococcus3.id.11303	rs8100692	19	C	T	0.057741093	0.011347595	4.16459E-07	15829	0.001633048	25.89180675
genus.Coprococcus3.id.11303	rs62481985	8	C	G	-0.058238523	0.011505221	4.1756E-07	15472	0.001653353	25.62304804
genus.Coprococcus3.id.11303	rs178271	22	C	T	0.145256923	0.029423868	7.81088E-07	10794	0.002252746	24.37104447
genus.Coprococcus3.id.11303	rs13394391	2	T	C	-0.070930158	0.015091785	2.20317E-06	15834	0.001393107	22.08923466
genus.Coprococcus3.id.11303	rs11080344	17	T	C	0.051692096	0.011305408	4.78692E-06	15835	0.001318514	20.90623558
genus.Coprococcus3.id.11303	rs7521171	1	A	G	-0.05964344	0.012927857	4.32242E-06	15472	0.001373817	21.28493198
genus.Coprococcus3.id.11303	rs11077359	17	C	T	-0.064544278	0.014883314	9.63598E-06	15378	0.001221478	18.80685684
genus.Coprococcus3.id.11303	rs13247359	7	A	G	0.051210989	0.011303088	7.32611E-06	15833	0.00129481	20.52731195
genus.Coprococcus3.id.11303	rs4575475	14	A	G	0.061951375	0.013778722	7.04284E-06	15154	0.001332226	20.21547745
genus.Coprococcus3.id.11303	rs10810043	9	G	A	0.051551783	0.011592302	9.27424E-06	15833	0.001247506	19.77643464
genus.DefluviitaleaceaeUCG011.id.	rs4677103	3	G	A	0.09779121	0.019726332	9.59557E-07	7983	0.003069064	24.57575924
genus.DefluviitaleaceaeUCG011.id.	rs72731813	4	T	C	-0.147382972	0.029379613	4.33116E-07	7767	0.003229567	25.16532123
genus.DefluviitaleaceaeUCG011.id.	rs112893842	9	C	T	0.113810202	0.023280562	1.44535E-06	8183	0.002912034	23.89876646
genus.DefluviitaleaceaeUCG011.id.	rs9725395	1	G	A	-0.138339669	0.029554002	3.51921E-06	8011	0.002727645	21.91093237
genus.DefluviitaleaceaeUCG011.id.	rs55658617	21	C	T	0.174368775	0.036222554	2.15292E-06	6663	0.003465786	23.17284232
genus.DefluviitaleaceaeUCG011.id.	rs9608282	22	G	T	0.14293919	0.029979298	2.52045E-06	7716	0.002937581	22.73315452
genus.DefluviitaleaceaeUCG011.id.	rs28696126	7	T	A	-0.106502748	0.023847256	6.62806E-06	7811	0.00254701	19.94549335
genus.DefluviitaleaceaeUCG011.id.	rs1582238	1	C	T	0.080503588	0.016725672	1.56775E-06	7983	0.002893601	23.16665435
genus.DefluviitaleaceaeUCG011.id.	rs4344384	10	T	G	0.071597782	0.015630912	4.83238E-06	8180	0.002558378	20.98121006
genus.DefluviitaleaceaeUCG011.id.	rs2892880	4	A	G	0.081775542	0.018168647	6.82881E-06	8182	0.002469837	20.25823862
genus.Desulfovibrio.id.3173	rs11160353	14	A	T	-0.069175564	0.014728505	2.70303E-06	9234	0.002383208	22.05911408
genus.Desulfovibrio.id.3173	rs2853179	8	T	C	0.081197078	0.017423443	2.42439E-06	8949	0.002420949	21.71765007
genus.Desulfovibrio.id.3173	rs16863365	2	G	A	0.109402491	0.022696375	1.7945E-06	8776	0.002640563	23.23493346
genus.Desulfovibrio.id.3173	rs12031543	1	C	T	-0.127184982	0.028188722	6.54764E-06	8855	0.002293692	20.35733317
genus.Desulfovibrio.id.3173	rs13066142	3	A	G	0.11914059	0.025089055	3.78665E-06	9241	0.002434296	22.55022584
genus.Desulfovibrio.id.3173	rs2590913	13	A	G	0.154495517	0.033939625	6.65421E-06	7225	0.002859803	20.72133324
genus.Desulfovibrio.id.3173	rs2032031	10	G	A	-0.065493057	0.014868084	9.14277E-06	9241	0.002095321	19.40351925
genus.Desulfovibrio.id.3173	rs72647089	8	G	T	-0.10659436	0.023933668	8.29763E-06	8985	0.002202795	19.83580945
genus.Desulfovibrio.id.3173	rs7729080	5	A	C	-0.070286961	0.015769108	9.95869E-06	9241	0.002145278	19.8671367
genus.Desulfovibrio.id.3173	rs4797774	18	A	G	0.21258194	0.04700444	5.64315E-06	3476	0.005849883	20.45384492

genus.Desulfovibrio.id.3173	rs6580353	5	C	T	0.0770979	0.016973529	4.93857E-06	9241	0.002227682	20.63197414
genus.Dialister.id.2183	rs11166701	8	A	G	-0.065530102	0.013186	5.50746E-07	11720	0.002102876	24.69764667
genus.Dialister.id.2183	rs517089	11	A	T	0.076227029	0.017041108	5.13634E-06	11988	0.001666293	20.00885923
genus.Dialister.id.2183	rs4263802	8	A	G	0.063465313	0.013243714	1.32682E-06	11989	0.001911786	22.96430304
genus.Dialister.id.2183	rs4747450	10	A	C	0.066850856	0.014768536	5.83729E-06	11987	0.001706423	20.4898628
genus.Dialister.id.2183	rs2314294	16	C	T	0.086593192	0.019371895	8.07899E-06	11339	0.001759073	19.98127948
genus.Dialister.id.2183	rs4753063	11	A	G	-0.059629704	0.013005185	4.85684E-06	11989	0.001750445	21.02288024
genus.Dialister.id.2183	rs2435610	7	C	A	0.064713149	0.014331424	5.93269E-06	11984	0.001698503	20.389491
genus.Dialister.id.2183	rs10938938	4	A	G	-0.077351446	0.017091491	7.37063E-06	11401	0.001793307	20.48221871
genus.Dialister.id.2183	rs10138457	14	C	T	-0.113053794	0.026193253	7.88122E-06	10968	0.001695614	18.62907952
genus.Dialister.id.2183	rs11071887	15	C	T	0.066240302	0.014631484	5.91407E-06	11989	0.001706644	20.49593968
genus.Dialister.id.2183	rs764177	3	A	C	-0.060145359	0.013536587	9.60772E-06	11988	0.001644086	19.74175936
genus.Dialister.id.2183	rs76680460	9	A	G	-0.16129611	0.036433211	8.19213E-06	7176	0.002723867	19.59985665
genus.Dialister.id.2183	rs75416973	1	G	A	0.072722304	0.016450983	9.46091E-06	11402	0.001710909	19.54121337
genus.Dorea.id.1997	rs13279148	8	A	G	0.071530899	0.015087753	2.25283E-06	17605	0.001275111	22.47699305
genus.Dorea.id.1997	rs62503162	8	G	A	-0.09740913	0.019437924	7.46605E-07	16210	0.001546836	25.11305511
genus.Dorea.id.1997	rs12216169	6	A	T	0.088172222	0.019368315	5.33123E-06	16086	0.001286686	20.72430187
genus.Dorea.id.1997	rs73729431	6	T	C	-0.137450296	0.029998324	3.1674E-06	9596	0.002183022	20.99410551
genus.Dorea.id.1997	rs4793307	17	T	C	0.057416514	0.012247163	4.0069E-06	17600	0.001247234	21.97873148
genus.Dorea.id.1997	rs11150408	16	G	T	0.04882539	0.010906337	7.05777E-06	17215	0.001162843	20.04165497
genus.Dorea.id.1997	rs345219	3	G	T	-0.049736844	0.011262081	8.8006E-06	17215	0.001131672	19.5037972
genus.Dorea.id.1997	rs62583469	9	A	T	-0.063413594	0.014222695	5.77508E-06	16819	0.001180559	19.8792923
genus.Dorea.id.1997	rs3005511	6	G	A	0.051612659	0.011285226	5.28702E-06	17610	0.001186362	20.91664161
genus.Dorea.id.1997	rs1899291	4	T	C	0.069721719	0.015028462	4.56686E-06	17609	0.001220793	21.52321414
genus.Dorea.id.1997	rs3752849	11	A	G	0.16384587	0.036607992	7.68057E-06	5322	0.00374984	20.03176347
genus.Dorea.id.1997	rs12537781	7	C	T	-0.055544008	0.012522298	9.15137E-06	17608	0.001116121	19.67461959
genus.Eggerthella.id.819	rs3851328	2	G	T	-0.107774251	0.023652669	4.17589E-06	5147	0.004017605	20.76202462
genus.Eggerthella.id.819	rs2240838	7	G	A	0.098058185	0.01976528	7.36205E-07	5147	0.004759219	24.6128403
genus.Eggerthella.id.819	rs2223081	21	A	G	0.102571659	0.022110762	3.88889E-06	5147	0.004163716	21.52025129
genus.Eggerthella.id.819	rs112205261	1	C	T	-0.188617927	0.040361319	3.34648E-06	5036	0.004317877	21.83912518
genus.Eggerthella.id.819	rs4985746	17	A	G	0.110522311	0.024765265	5.71461E-06	4906	0.004043216	19.91654244
genus.Eggerthella.id.819	rs1784446	11	A	G	0.090772774	0.019809579	5.23136E-06	5147	0.004062922	20.99716783
genus.Eggerthella.id.819	rs76663501	20	T	C	0.175349437	0.03785764	4.82574E-06	4814	0.004436745	21.45367407
genus.Eggerthella.id.819	rs6430926	2	T	C	0.08797364	0.019712748	8.37122E-06	5147	0.0038546	19.9163981
genus.Eggerthella.id.819	rs2877457	4	A	G	-0.093486043	0.021020752	9.02708E-06	5083	0.003876059	19.77866981
genus.Eggerthella.id.819	rs12701617	7	G	A	-0.089940242	0.019866367	6.74893E-06	5147	0.00396635	20.49609802
genus.Eggerthella.id.819	rs13070736	3	C	A	-0.12133107	0.027202382	7.61915E-06	5147	0.003850353	19.89436956
genus.Eggerthella.id.819	rs67490567	15	C	T	0.108494846	0.024545741	8.94216E-06	5083	0.00382895	19.5373626
genus.Eisenbergiella.id.11304	rs3812426	8	A	G	0.106447316	0.0224159	2.71687E-06	5583	0.004022896	22.550546
genus.Eisenbergiella.id.11304	rs1508033	15	C	A	0.091545961	0.019578652	3.22889E-06	5580	0.003902836	21.86315322
genus.Eisenbergiella.id.11304	rs12278566	11	A	T	-0.121064706	0.025165804	1.65001E-06	5582	0.004128828	23.14267106
genus.Eisenbergiella.id.11304	rs2683098	15	T	C	0.107318939	0.022515029	2.2375E-06	5582	0.00405372	22.71996758
genus.Eisenbergiella.id.11304	rs11079158	17	C	T	0.100626785	0.022546032	7.34849E-06	5582	0.003555904	19.91989083
genus.Eisenbergiella.id.11304	rs13258851	8	G	A	0.137001443	0.030212434	7.74648E-06	5583	0.003669565	20.562639
genus.Eisenbergiella.id.11304	rs12710729	2	A	C	0.089338392	0.019906928	9.8441E-06	5583	0.003594482	20.14038545
genus.Eisenbergiella.id.11304	rs12257723	10	C	A	-0.095274183	0.021156892	8.85121E-06	5582	0.003619778	20.27900909
genus.Eisenbergiella.id.11304	rs4462860	21	A	G	0.093907092	0.020110429	4.16221E-06	5460	0.003977686	21.80490037
genus.Eisenbergiella.id.11304	rs11027642	11	T	C	0.129005538	0.028484392	4.91871E-06	5583	0.003660518	20.51175329
genus.Eisenbergiella.id.11304	rs11938607	4	C	T	0.097812525	0.021663562	8.2182E-06	5583	0.003638134	20.38586771
genus.Eisenbergiella.id.11304	rs1553971	3	G	T	0.120964524	0.026305423	5.26837E-06	5583	0.003773254	21.14586556
genus.Enterorhabdus.id.820	rs11098863	4	A	T	-0.096600831	0.016267701	3.05646E-09	7524	0.004664767	35.26219681
genus.Enterorhabdus.id.820	rs7923280	10	T	A	0.086001907	0.016914834	5.23856E-07	7524	0.003424067	25.85119738
genus.Enterorhabdus.id.820	rs114731706	2	G	T	0.182308046	0.038229783	2.17342E-06	5956	0.00380363	22.74091887
genus.Enterorhabdus.id.820	rs3017103	11	G	A	0.098089701	0.020899456	2.94044E-06	7524	0.002919164	22.02809082
genus.Enterorhabdus.id.820	rs73331712	12	C	T	0.261989512	0.055123019	4.84923E-06	3109	0.00721335	22.58925032
genus.Enterorhabdus.id.820	rs77655283	2	A	G	0.132987641	0.029830551	5.88042E-06	7494	0.002645063	19.87467409
genus.Enterorhabdus.id.820	rs9470637	6	T	A	-0.075551295	0.016626296	5.69624E-06	7524	0.002736868	20.64870549
genus.Enterorhabdus.id.820	rs424715	19	C	T	0.081874254	0.017485462	4.4088E-06	7524	0.002905548	21.92504444
genus.Enterorhabdus.id.820	rs10098492	8	C	T	0.132323148	0.029368842	6.40572E-06	7361	0.002750205	20.30009151
genus.Enterorhabdus.id.820	rs2051957	7	T	C	0.084329062	0.018986405	8.89859E-06	7524	0.002615069	19.72736469
genus.Erysipelatoclostridium.id.113	rs7221249	17	G	A	0.083986052	0.01427414	4.30951E-09	9781	0.003526929	34.61899223
genus.Erysipelatoclostridium.id.113	rs710230	1	C	T	0.143381274	0.028151388	6.3312E-07	9016	0.002868954	25.94091274
genus.Erysipelatoclostridium.id.113	rs4697572	4	G	A	-0.081063643	0.016330822	7.5935E-07	9779	0.002513326	24.63974184
genus.Erysipelatoclostridium.id.113	rs58236560	11	T	G	-0.11115688	0.023442572	2.1612E-06	9368	0.002394276	22.4834096
genus.Erysipelatoclostridium.id.113	rs622418	9	G	A	-0.066805108	0.014322449	3.68156E-06	9783	0.002218952	21.75628589
genus.Erysipelatoclostridium.id.113	rs17804233	5	C	T	-0.066291401	0.014420319	4.58605E-06	9783	0.002155537	21.13317206
genus.Erysipelatoclostridium.id.113	rs1434153	2	A	G	-0.06845264	0.01524432	6.85295E-06	9662	0.002082533	20.16342339
genus.Erysipelatoclostridium.id.113	rs340991	5	G	A	-0.074048187	0.015889138	3.74942E-06	9781	0.00221555	21.71841667
genus.Erysipelatoclostridium.id.113	rs6474512	8	C	A	0.067014995	0.014320143	3.01546E-06	9783	0.002233604	21.90025963
genus.Erysipelatoclostridium.id.113	rs9590927	13	A	G	-0.064600302	0.014347949	6.39042E-06	9782	0.002068058	20.27166477
genus.Erysipelatoclostridium.id.113	rs61806970	1	T	C	0.142547998	0.032068815	9.0941E-06	8644	0.002280605	19.75861174
genus.Erysipelatoclostridium.id.113	rs45480394	19	G	T	-0.068713443	0.015183535	7.66155E-06	9604	0.002127945	20.48036061
genus.Erysipelatoclostridium.id.113	rs2901723	11	A	C	0.064133865	0.014429456	8.79469E-06	9662	0.002040426	19.75490366
genus.Erysipelatoclostridium.id.113	rs34528142	17	G	C	-0.087555073	0.019555627	6.12704E-06	9604	0.002082867	20.04560305
genus.Erysipelatoclostridium.id.113	rs16936671	10	T	C	-0.096802556	0.021788273	6.04309E-06	9488	0.002076112	19.73913136
genus.Erysipelatoclostridium.id.113	rs3804326	6	G	A	0.141469838	0.033635154	9.85273E-06	7028	0.00251083	17.69053128
genus.ErysipelotrichaceaeUCG003.	rs28568391	10	G	A	-0.058391081	0.011874052	6.41953E-07	14544	0.00165993	24.18216413
genus.ErysipelotrichaceaeUCG003.	rs11994308	8	T	C	0.115417413	0.024250252	1.32563E-06	13558	0.001667975	22.65218787
genus.ErysipelotrichaceaeUCG003.	rs76502207	15	C	T	0.14484376	0.028992913	6.41121E-07	10740	0.002318481	24.95834976
genus.ErysipelotrichaceaeUCG003.	rs10164067	18	G	T	-0.103260131	0.021240502	1.13086E-06	14427	0.001635494	23.63392191
genus.ErysipelotrichaceaeUCG003.	rs8053479	16	G	A	-0.083821185	0.018650912	5.83413E-06	13813	0.001460108	20.19795688
genus.ErysipelotrichaceaeUCG003.	rs17798136	7	A	G	0.15881755	0.034767431	3.23598E-06	7421	0.002803948	20.8666043
genus.ErysipelotrichaceaeUCG003.	rs59104037	18	G	A	-0.095126261	0.020474209	4.48256E-06	14848	0.001451736	21.586717
genus.ErysipelotrichaceaeUCG003.	rs11666127	19	G	A	-0.07191871	0.016137058	7.90414E-06	13813	0.001435896	19.86255135
genus.ErysipelotrichaceaeUCG003.	rs62403464	6	C	T	-0.073174326	0.015651572	3.43738E-06	14899	0.001464899	21.85755205
genus.ErysipelotrichaceaeUCG003.	rs59068084	4	G	T	0.056477243	0.012022387	3.11742E-06	14544	0.001515037	22.06813306
genus.ErysipelotrichaceaeUCG003.	rs73074432	7	T	C	0.072153999	0.016437785	9.9902E-06	14217	0.001353437	19.26789216
genus.ErysipelotrichaceaeUCG003.	rs4758231	11	T	G	-0.05517544	0.012204675	6.54912E-06	14890	0.00137072	20.43803604
genus.ErysipelotrichaceaeUCG003.	rs74988980	16	A	G	-0.133141406	0.034857439	8.64276E-06	5944	0.002448454	14.58932975
genus.ErysipelotrichaceaeUCG003.	rs75949021	2	C	T	-0.16966706	0.037423793	3.57507E-06	6721	0.003048874	20.55414697
genus.ErysipelotrichaceaeUCG003.	rs6875357	5	T	C	0.165673441	0.035383116	6.69637E-06	7034	0.003107132	21.92368858
genus.ErysipelotrichaceaeUCG003.	rs79396538	14	G	C	0.084814752	0.019114638	8.63479E-06	13750	0.001429837	19.68840996
genus.ErysipelotrichaceaeUCG003.	rs12251396	10	G	A	-0.070539949	0.015876598	9.51839E-06	14211	0.001387164	19.74037496
genus.Escherichia.Shigella.id.3504	rs2267739	7	C	G	0.115505392	0.02376277	1.42478E-06	11853	0.001989378	23.62709855
genus.Escherichia.Shigella.id.3504	rs1154904	11	G	A	-0.061337641	0.01306443	3.03751E-06	12002	0.001833254	22.04312858
genus.Escherichia.Shigella.id.3504	rs73208162	21	G	A	-0.119295648	0.024838994	2.19391E-06	11073	0.002078797	23.06647404
genus.Escherichia.Shigella.id.3504	rs113513883	7	G	A	0.172283306	0.038041024	5.27716E-06	6280	0.003255414	20.51077113
genus.Escherichia.Shigella.id.3504	rs7502686	17	C	G	-0.136383738	0.030257643	5.90386E-06	9990	0.002029585	20.31678535
genus.Escherichia.Shigella.id.3504	rs57024273	2	C	T	0.062617236	0.013991866	9.70464E-06	11853	0.001686845	20.02795192
genus.Escherichia.Shigella.id.3504	rs113127095	13	G	A	0.151009395	0.032343436	3.33349E-06	8433	0.002578293	21.79895181
genus.Escherichia.Shigella.id.3504	rs117092367	10	T	A	0.11736182	0.026453289	9.65021E-06	10304	0.0019066	19.68313747
genus.Escherichia.Shigella.id.3504	rs112767262	16	C	T	0.073296333	0.01635886	8.20636E-06	11771	0.00170257	20.07513314
genus.Escherichia.Shigella.id.3504	rs11706043	3	A	T	0.07565695	0.016415509	5.87272E-06	12001	0.001766866	21.24168415
genus.Escherichia.Shigella.id.3504	rs592299	9	C	T	-0.059200433	0.012948643	4.76927E-06	11995	0.001739582	20.90264529
genus.Escherichia.Shigella.id.3504	rs4731451	7	A	G	-0.060988547	0.013515708	7.4731E-06	12001	0.001693812	20.36193079
genus.Escherichia.Shigella.id.3504	rs35555519	16	G	C	0.101975959	0.022288306	4.92332E-06	12002	0.001741129	20.93348246
genus.Escherichia.Shigella.id.3504	rs118526	5	A	C	-0.059440114	0.013598005	7.99566E-06	11853	0.001609463	19.10771908
genus.Escherichia.Shigella.id.3504	rs2798105	1	G	A	-0.100850689	0.02220448	8.24501E-06	11296	0.001822886	20.62892213
genus.Faecalibacterium.id.2057	rs12320842	12	G	C	0.094832958	0.016396224	7.56951E-09	17638	0.001893035	33.45267919
genus.Faecalibacterium.id.2057	rs6910935	6	G	A	0.134863869	0.027703163	1.37848E-06	12640	0.001871423	23.69913625
genus.Faecalibacterium.id.2057	rs1271565	14	T	C	-0.057625117	0.011964928	1.30169E-06	17638	0.001313359	23.19548528
genus.Faecalibacterium.id.2057	rs75499067	16	T	C	0.227584711	0.046552213	1.76448E-06	4526	0.005252954	23.90041823
genus.Faecalibacterium.id.2057	rs9536330	13	C	T	-0.048317039	0.01079702	5.33258E-06	18082	0.001106282	20.02593689
genus.Faecalibacterium.id.2057	rs28376661	15	G	C	0.05048173	0.010936797	3.66454E-06	18087	0.00117655	21.30532472
genus.Faecalibacterium.id.2057	rs10927394	1	T	G	-0.232262107	0.051236432	7.01885E-06	3495	0.005845286	20.54939141
genus.Faecalibacterium.id.2057	rs114946999	2	T	C	-0.086156499	0.018960043	5.69574E-06	15673	0.00131575	20.648926
genus.Faecalibacterium.id.2057	rs79656633	1	C	T	0.145630704	0.032301873	8.13557E-06	9080	0.002233539	20.32593249
genus.Faecalibacterium.id.2057	rs61875484	10	G	C	0.081795263	0.018369023	9.18015E-06	16718	0.001184636	19.82824181
genus.Faecalibacterium.id.2057	rs12753492	1	C	A	0.064130474	0.014993333	8.80273E-06	17525	0.001042849	18.29500433
genus.Faecalibacterium.id.2057	rs2835874	21	C	T	-0.086635636	0.019649581	7.54405E-06	17567	0.001105372	19.43956603
genus.Faecalibacterium.id.2057	rs11776390	8	C	T	-0.078353971	0.017183046	6.39675E-06	16718	0.001242217	20.7932175
genus.FamilyXIIIAD3011group.id.	rs9852893	3	G	C	0.065783419	0.012905622	3.87539E-07	14184	0.001828443	25.98214644
genus.FamilyXIIIAD3011group.id.	rs62200412	20	T	C	-0.080085132	0.016383437	5.80219E-07	13661	0.001746033	23.89427054
genus.FamilyXIIIAD3011group.id.	rs16840310	1	G	A	-0.060805195	0.012214901	6.75113E-07	14201	0.001741909	24.78001027
genus.FamilyXIIIAD3011group.id.	rs72730932	1	A	C	-0.089955866	0.017710652	6.88766E-07	13102	0.00196516	25.79822789
genus.FamilyXIIIAD3011group.id.	rs16940167	15	T	C	0.073255157	0.015989797	3.90837E-06	14201	0.001475809	20.98894112
genus.FamilyXIIIAD3011group.id.	rs17156849	7	A	G	-0.112890921	0.024529236	4.19171E-06	13352	0.001583854	21.18117239
genus.FamilyXIIIAD3011group.id.	rs62029761	16	G	A	0.128752725	0.027600154	3.89083E-06	9655	0.002248846	21.76155124
genus.FamilyXIIIAD3011group.id.	rs11736617	4	A	G	-0.075949337	0.017210081	9.02048E-06	13778	0.001411505	19.47520918

genus.FamilyXIIIAD3011group.id.	rs12812672	12	C	T	-0.096065015	0.020828023	2.55807E-06	13565	0.001565792	21.27327872
genus.FamilyXIIIAD3011group.id.	rs9276029	6	G	A	-0.081138392	0.018566861	8.92766E-06	13566	0.001405766	19.09746845
genus.FamilyXIIIAD3011group.id.	rs12911842	15	T	A	-0.081197635	0.018333544	6.90824E-06	14125	0.001386765	19.61525317
genus.FamilyXIIIAD3011group.id.	rs149302	5	C	T	-0.064563538	0.014322495	7.4818E-06	14192	0.00142979	20.32063556
genus.FamilyXIIIAD3011group.id.	rs9837139	3	G	A	0.10752022	0.024048177	8.70808E-06	13527	0.001475615	19.99014556
genus.FamilyXIIIAD3011group.id.	rs739451	9	T	C	0.064958894	0.014753414	7.8781E-06	14197	0.00136365	19.38617718
genus.FamilyXIIIAD3011group.id.	rs11126423	2	T	C	0.090439883	0.019630134	5.91249E-06	13735	0.001543029	21.22625966
genus.FamilyXIIIUCG001.id.11294	rs1426266	3	C	T	-0.066552114	0.013715073	1.24935E-06	12751	0.00184324	23.54656044
genus.FamilyXIIIUCG001.id.11294	rs12049454	1	C	T	-0.064729775	0.013406537	1.17378E-06	12748	0.001825322	23.31175436
genus.FamilyXIIIUCG001.id.11294	rs3842897	1	A	G	-0.112639326	0.024275096	5.19705E-06	12407	0.001732361	21.53070203
genus.FamilyXIIIUCG001.id.11294	rs116979587	15	A	T	-0.121678937	0.02608207	3.04638E-06	11989	0.001812074	21.76439948
genus.FamilyXIIIUCG001.id.11294	rs62414802	6	T	C	-0.061190403	0.013456987	4.29181E-06	12750	0.001619038	20.67621106
genus.FamilyXIIIUCG001.id.11294	rs2276529	21	G	C	-0.075940605	0.016509926	5.35677E-06	12748	0.001656897	21.15718207
genus.FamilyXIIIUCG001.id.11294	rs112362903	17	G	A	-0.149047048	0.033327141	7.8777E-06	8579	0.002325962	20.00095089
genus.FamilyXIIIUCG001.id.11294	rs8076666	17	G	A	0.088655005	0.019809411	8.0183E-06	12265	0.001630374	20.02919122
genus.FamilyXIIIUCG001.id.11294	rs7119679	11	A	G	-0.080908465	0.017478204	3.52494E-06	12445	0.001718907	21.42862601
genus.FamilyXIIIUCG001.id.11294	rs76463770	3	G	A	0.193129687	0.04198659	3.77476E-06	5651	0.003730169	21.15810956
genus.Flavonifractor.id.2059	rs12030302	1	G	A	-0.069227582	0.013746695	5.6084E-07	10805	0.002341633	25.3607296
genus.Flavonifractor.id.2059	rs806808	10	C	T	0.0667272	0.013655748	1.17972E-06	10801	0.002205727	23.87672746
genus.Flavonifractor.id.2059	rs34066017	11	G	A	0.076429405	0.015978927	1.52086E-06	10705	0.002132612	22.8784038
genus.Flavonifractor.id.2059	rs11811696	1	C	T	-0.116062715	0.024101172	2.07346E-06	10221	0.002263766	23.19044694
genus.Flavonifractor.id.2059	rs11642826	16	C	G	0.146860461	0.032519094	6.64895E-06	8151	0.002495955	20.39543192
genus.Flavonifractor.id.2059	rs114873521	5	T	C	-0.130066614	0.029408684	7.12522E-06	9156	0.002131807	19.56052419
genus.Flavonifractor.id.2059	rs6761463	2	C	G	-0.083391953	0.018459615	8.10902E-06	10805	0.001885206	20.40811978
genus.Flavonifractor.id.2059	rs12038887	1	G	C	0.094271903	0.021164606	9.37203E-06	10583	0.001871208	19.84011399
genus.Fusicatenibacter.id.11305	rs4378146	1	C	A	-0.061669056	0.012525884	7.20451E-07	17384	0.001392397	24.23917392
genus.Fusicatenibacter.id.11305	rs62353480	5	G	A	-0.070139694	0.014558884	1.56768E-06	16119	0.001437833	23.20980992
genus.Fusicatenibacter.id.11305	rs704418	3	C	T	0.073912422	0.015107283	7.77428E-07	16626	0.001437638	23.93658056
genus.Fusicatenibacter.id.11305	rs2132128	8	A	G	-0.07719139	0.016034448	1.07686E-06	17370	0.00133245	23.17553165
genus.Fusicatenibacter.id.11305	rs206581	18	G	A	-0.056833856	0.012789397	8.95756E-06	17384	0.001134675	19.74759023
genus.Fusicatenibacter.id.11305	rs62187631	2	C	T	-0.07105711	0.015924014	4.54526E-06	16522	0.001203717	19.91177596
genus.Fusicatenibacter.id.11305	rs2025938	10	A	G	-0.096729791	0.020542557	2.98832E-06	15626	0.001416928	22.17233752
genus.Fusicatenibacter.id.11305	rs3303	10	C	T	-0.095365713	0.020406992	3.93891E-06	16877	0.001292319	21.83868841
genus.Fusicatenibacter.id.11305	rs2039204	9	A	T	-0.04974145	0.010794137	3.94178E-06	17383	0.00122013	21.23543034
genus.Fusicatenibacter.id.11305	rs8028026	15	G	A	-0.079214582	0.018050473	8.0633E-06	16522	0.001164299	19.25897211
genus.Fusicatenibacter.id.11305	rs1864685	17	C	A	-0.049479928	0.010810494	4.96161E-06	17374	0.001204326	20.94918087
genus.Fusicatenibacter.id.11305	rs792108	2	C	T	-0.050834518	0.011384326	8.49535E-06	16978	0.001173023	19.93897105
genus.Fusicatenibacter.id.11305	rs60254196	7	G	A	-0.049242701	0.010936491	5.46739E-06	16978	0.001192677	20.27345522
genus.Fusicatenibacter.id.11305	rs9905659	17	A	G	-0.06161703	0.013657585	7.30993E-06	17384	0.001169488	20.35418449
genus.Fusicatenibacter.id.11305	rs8063430	16	C	T	-0.104026455	0.022216952	4.9289E-06	15552	0.001407734	21.92394275
genus.Fusicatenibacter.id.11305	rs6515626	20	A	G	0.141575028	0.031355872	7.28654E-06	7796	0.002608132	20.38616521
genus.Fusicatenibacter.id.11305	rs10439674	21	G	A	-0.057212907	0.013000675	7.68485E-06	17381	0.001113007	19.36672583
genus.Fusicatenibacter.id.11305	rs167879	20	T	C	-0.065954324	0.014876261	5.86893E-06	16542	0.001186848	19.65617418
genus.Fusicatenibacter.id.11305	rs73103914	12	G	A	-0.059732424	0.013445773	8.29563E-06	17384	0.001133982	19.73552661
genus.Gordonibacter.id.821	rs76287110	3	T	A	-0.242964921	0.046617731	1.67486E-07	3483	0.007738514	27.16344775
genus.Gordonibacter.id.821	rs7294633	12	T	C	0.128670034	0.024994637	3.43763E-07	3723	0.007067856	26.5009337
genus.Gordonibacter.id.821	rs7220558	17	T	A	0.116884144	0.02348337	6.70634E-07	3723	0.006610236	24.77366996
genus.Gordonibacter.id.821	rs71545975	7	G	A	-0.153972004	0.033891756	7.03944E-06	3556	0.00577059	20.63931929
genus.Gordonibacter.id.821	rs35042269	4	A	C	-0.180292677	0.040329634	8.10852E-06	3595	0.005528421	19.98515942
genus.Gordonibacter.id.821	rs72714787	4	A	C	0.181404779	0.037710172	1.42889E-06	3723	0.006177266	23.14091001
genus.Gordonibacter.id.821	rs322296	7	A	G	0.178689528	0.037721957	4.02422E-06	3595	0.006203096	22.43932377
genus.Gordonibacter.id.821	rs72939513	1	G	A	-0.213989639	0.049059194	7.98199E-06	3435	0.005508319	19.02587659
genus.Gordonibacter.id.821	rs3765837	1	G	T	-0.190728517	0.043360843	7.16906E-06	3556	0.005411498	19.34798899
genus.Gordonibacter.id.821	rs13412653	2	C	A	0.107597447	0.023922776	8.61141E-06	3723	0.005404237	20.2292971
genus.Gordonibacter.id.821	rs117347059	7	C	G	-0.128261008	0.028512264	9.16713E-06	3684	0.00546295	20.23605655
genus.Gordonibacter.id.821	rs16955299	17	A	G	-0.196422874	0.043353771	6.37019E-06	3572	0.005713869	20.52723049
genus.Gordonibacter.id.821	rs4596722	9	G	A	0.102908274	0.023157103	9.0568E-06	3723	0.005276446	19.74840988
genus.Gordonibacter.id.821	rs768830	7	A	G	0.149852407	0.033331454	7.75583E-06	3723	0.00539976	20.21244816
genus.Gordonibacter.id.821	rs61934597	12	T	C	-0.172494533	0.038856681	8.36672E-06	3684	0.00532087	19.70694287
genus.Haemophilus.id.3698	rs12191680	6	G	C	0.106544332	0.020032321	1.46913E-07	9115	0.003093825	28.28773302
genus.Haemophilus.id.3698	rs9382510	6	T	C	-0.093521046	0.017264852	7.12051E-08	9117	0.003208083	29.34222101
genus.Haemophilus.id.3698	rs9574096	13	T	A	-0.073637529	0.015522726	2.1802E-06	9118	0.002462024	22.50413929
genus.Haemophilus.id.3698	rs76022354	10	T	C	0.244637568	0.050550437	1.83031E-06	3618	0.006431698	23.42051706
genus.Haemophilus.id.3698	rs111582866	16	A	G	-0.124265408	0.026016016	1.27347E-06	8370	0.002718387	22.81491904
genus.Haemophilus.id.3698	rs9895850	17	C	T	-0.192956876	0.041675519	2.1406E-06	4592	0.004646585	21.43672768
genus.Haemophilus.id.3698	rs35509	12	A	G	0.128248955	0.026877911	2.00952E-06	8430	0.002693504	22.76756727
genus.Haemophilus.id.3698	rs78909003	9	C	T	-0.246255715	0.050392389	1.66745E-06	3559	0.006665158	23.88046288
genus.Haemophilus.id.3698	rs35510	12	G	A	0.127598588	0.026861358	2.20249E-06	8430	0.002669607	22.56502369
genus.Haemophilus.id.3698	rs9328464	6	C	T	0.072309489	0.014879949	1.42269E-06	9119	0.002582957	23.61498538
genus.Haemophilus.id.3698	rs4822728	22	C	T	0.070586099	0.015138761	3.47849E-06	9119	0.002378353	21.73990701
genus.Haemophilus.id.3698	rs10840326	11	G	C	-0.067738403	0.015146258	7.36533E-06	8888	0.002245323	20.00134392
genus.Haemophilus.id.3698	rs12876183	13	A	T	0.074920105	0.016710973	9.6249E-06	9110	0.002201495	20.0998713
genus.Haemophilus.id.3698	rs10781340	9	A	G	0.094891501	0.020322072	4.32266E-06	9037	0.002406843	21.80312024
genus.Haemophilus.id.3698	rs56310940	7	C	G	-0.108393321	0.024705186	7.23232E-06	9119	0.002106521	19.24991332
genus.Holdemanella.id.11393	rs761624	6	G	C	0.095980128	0.017953732	1.37775E-07	7706	0.003695017	28.57940984
genus.Holdemanella.id.11393	rs607782	6	C	T	-0.08542142	0.017251511	7.19447E-07	7456	0.003277537	24.51767666
genus.Holdemanella.id.11393	rs75764681	10	C	T	-0.283102607	0.059899873	1.94273E-06	3003	0.007383497	22.33757033
genus.Holdemanella.id.11393	rs73011279	19	C	T	-0.096166927	0.019933645	1.36046E-06	7456	0.003111849	23.27437479
genus.Holdemanella.id.11393	rs4541991	9	C	T	-0.092733374	0.019443459	2.10094E-06	7657	0.002961953	22.74704911
genus.Holdemanella.id.11393	rs1830029	7	G	C	-0.095409594	0.021093039	5.34728E-06	7456	0.00273659	20.46000685
genus.Holdemanella.id.11393	rs12513188	4	A	G	0.090390629	0.019527621	4.6548E-06	7700	0.002774921	21.42634799
genus.Holdemanella.id.11393	rs12415649	10	C	G	0.083938435	0.019056694	7.87995E-06	7662	0.002525726	19.40111111
genus.Holdemanella.id.11393	rs1926302	1	A	G	-0.107971755	0.023142056	7.50141E-06	7702	0.0028183	21.76789488
genus.Holdemanella.id.11393	rs17586763	13	C	T	-0.227284509	0.051013457	7.71949E-06	3817	0.005173629	19.85044119
genus.Holdemanella.id.11393	rs8113760	19	A	G	0.078998984	0.017340046	4.62395E-06	7456	0.002776063	20.75594547
genus.Holdemanella.id.11393	rs34187114	8	A	C	-0.104505235	0.022600956	5.13495E-06	7704	0.002767595	21.38072675
genus.Holdemanella.id.11393	rs35228298	3	A	G	0.093483215	0.020288588	7.29849E-06	7456	0.002839376	21.2306672
genus.Holdemanella.id.11393	rs62113381	19	C	T	-0.105447171	0.023202912	5.53879E-06	7310	0.002817359	20.65308309
genus.Holdemania.id.2157	rs1867876	11	C	T	0.084291862	0.016219181	2.7388E-07	9109	0.002956357	27.00930652
genus.Holdemania.id.2157	rs9500080	6	T	C	0.092676436	0.01788862	4.08515E-07	9106	0.002938861	26.84015128
genus.Holdemania.id.2157	rs6133067	20	C	T	0.091082873	0.017856087	5.16929E-07	8863	0.002927164	26.01961505
genus.Holdemania.id.2157	rs150096134	12	A	T	0.162139279	0.033208945	2.37767E-06	6906	0.00343988	23.83780862
genus.Holdemania.id.2157	rs41438744	14	G	C	-0.124870498	0.02701295	2.44434E-06	8839	0.002411703	21.36857988
genus.Holdemania.id.2157	rs77293403	5	G	A	0.164556483	0.034177476	1.77466E-06	7585	0.003046977	23.18195313
genus.Holdemania.id.2157	rs80149660	10	T	C	-0.23298738	0.051918311	6.04217E-06	3283	0.006096729	20.13834084
genus.Holdemania.id.2157	rs116500994	13	T	G	-0.137550456	0.029338596	2.34479E-06	8432	0.002600065	21.98089759
genus.Holdemania.id.2157	rs55888180	17	G	C	0.128670991	0.028279464	5.89194E-06	8464	0.002439958	20.70231581
genus.Holdemania.id.2157	rs4146507	5	T	C	0.079488043	0.017700231	7.22973E-06	9108	0.002209338	20.16720688
genus.Holdemania.id.2157	rs111745969	15	G	A	0.120676888	0.026578103	3.70776E-06	8233	0.00249779	20.61580292
genus.Holdemania.id.2157	rs113593397	8	G	A	-0.128934656	0.028252607	9.35728E-06	8296	0.002504175	20.8267868
genus.Holdemania.id.2157	rs10885477	10	C	T	-0.13513664	0.030189444	8.59833E-06	8690	0.002300467	20.03715295
genus.Holdemania.id.2157	rs9529719	13	C	T	0.074037972	0.016049433	5.96669E-06	9107	0.002331313	21.28088388
genus.Holdemania.id.2157	rs967319	3	C	T	0.078863839	0.017674402	8.38155E-06	9108	0.002181198	19.90977659
genus.Holdemania.id.2157	rs12701617	7	G	A	-0.066061737	0.014942681	9.51844E-06	9106	0.002141825	19.54532554
genus.Holdemania.id.2157	rs73139538	7	A	G	-0.148593595	0.032746911	7.77178E-06	8095	0.002537111	20.59014969
genus.Holdemania.id.2157	rs11080063	17	A	G	-0.066523819	0.014983728	6.66887E-06	9103	0.002160681	19.71126999
genus.Howardella.id.2000	rs901099	11	G	T	-0.127180025	0.025092403	6.52858E-07	3647	0.0069947	25.68936078
genus.Howardella.id.2000	rs1484873	18	G	A	-0.227831171	0.046335026	2.55918E-06	3608	0.006656415	24.1772809
genus.Howardella.id.2000	rs17167098	7	A	G	-0.16936566	0.035206781	1.12323E-06	3586	0.006412009	23.14185048
genus.Howardella.id.2000	rs609430	4	G	T	-0.112045365	0.023932786	3.34484E-06	3798	0.005737824	21.91801779
genus.Howardella.id.2000	rs36081916	7	C	T	-0.181229725	0.040298718	4.7004E-06	3216	0.006249392	20.22443502
genus.Howardella.id.2000	rs12452946	17	G	A	-0.105833103	0.022894084	3.80393E-06	3800	0.005592133	21.36960657
genus.Howardella.id.2000	rs61771805	1	T	A	-0.136778682	0.029681112	4.0274E-06	3612	0.005844978	21.23618513
genus.Howardella.id.2000	rs672217	18	A	G	0.164145673	0.034999266	3.52468E-06	3611	0.006054469	21.99586248
genus.Howardella.id.2000	rs3791893	2	G	A	0.147035334	0.034023014	9.49716E-06	3795	0.004897269	18.6766012
genus.Howardella.id.2000	rs10048062	15	T	C	-0.14735223	0.033653121	8.58873E-06	3800	0.005019882	19.17179325
genus.Howardella.id.2000	rs2154047	14	A	C	-0.192557008	0.042009328	9.97042E-06	3623	0.005765641	21.01005476
genus.Hungatella.id.11306	rs13128780	4	C	T	-0.149725257	0.031277743	1.74998E-06	3999	0.005697528	22.914972
genus.Hungatella.id.11306	rs72759041	15	T	G	-0.126025023	0.028224238	3.86095E-06	3910	0.005073218	19.93742747
genus.Hungatella.id.11306	rs10044993	5	A	C	0.139546965	0.031674777	8.06668E-06	4209	0.004590253	19.40946862
genus.Hungatella.id.11306	rs13249325	8	G	T	-0.100022583	0.022588331	9.69162E-06	4209	0.004636929	19.60775285
genus.Hungatella.id.11306	rs17092615	14	A	G	0.152234676	0.033786729	7.37677E-06	4009	0.005038544	20.30181581
genus.Intestinibacter.id.11345	rs10805326	4	A	G	0.077515061	0.013966468	3.54544E-08	12303	0.00249748	30.80342704
genus.Intestinibacter.id.11345	rs4327025	15	A	G	-0.081034559	0.015439666	1.64326E-07	12303	0.002233996	27.546393
genus.Intestinibacter.id.11345	rs478972	11	C	T	-0.142680973	0.02971163	1.81991E-06	10333	0.002226817	23.06105541
genus.Intestinibacter.id.11345	rs6875660	5	T	C	0.089045542	0.01939052	3.05781E-06	12293	0.00171255	21.08848764
genus.Intestinibacter.id.11345	rs16938435	9	C	T	-0.112188506	0.023543887	1.80097E-06	12303	0.001842165	22.7059875
genus.Intestinibacter.id.11345	rs6062862	20	G	A	0.092450812	0.020465817	6.67984E-06	12189	0.001671355	20.40625295
genus.Intestinibacter.id.11345	rs118030283	16	A	G	-0.15182897	0.032447117	2.67439E-06	8830	0.002473551	21.89561104

genus.Intestinibacter.id.11345	rs11109097	12	T	C	0.062427238	0.013853923	5.49484E-06	11886	0.001705398	20.30498569
genus.Intestinibacter.id.11345	rs9348442	6	T	C	0.099081964	0.022162526	6.25936E-06	12303	0.00162194	19.98714161
genus.Intestinibacter.id.11345	rs447950	5	G	A	0.062834174	0.01366523	5.63911E-06	12303	0.001715541	21.14257614
genus.Intestinibacter.id.11345	rs62430350	6	C	T	0.151095721	0.035147611	6.84229E-06	7650	0.002409924	18.48045674
genus.Intestinibacter.id.11345	rs2702387	4	G	A	0.060855918	0.013214549	4.25994E-06	12291	0.001722523	21.20806133
genus.Intestinibacter.id.11345	rs68093214	3	T	C	0.066225756	0.014987729	9.25769E-06	11886	0.001639961	19.52460094
genus.Intestinibacter.id.11345	rs2098844	11	T	C	-0.057529528	0.012841562	6.78879E-06	12291	0.001630235	20.06993305
genus.Intestinibacter.id.11345	rs893394	2	A	G	0.058332172	0.013072938	7.85038E-06	11886	0.001672274	19.90993889
genus.Intestinimonas.id.2062	rs11258178	10	G	A	0.066074232	0.013413886	6.98195E-07	11766	0.002057934	24.26358862
genus.Intestinimonas.id.2062	rs12226153	11	G	A	-0.151142451	0.030692229	5.12171E-07	9023	0.002680399	24.25024132
genus.Intestinimonas.id.2062	rs4784055	16	C	T	-0.175323954	0.038599023	8.71812E-07	3193	0.006419982	20.63145678
genus.Intestinimonas.id.2062	rs716604	2	G	A	0.081806835	0.016599238	8.5663E-07	12090	0.002004956	24.2886213
genus.Intestinimonas.id.2062	rs2930225	16	T	G	0.072952384	0.015294848	1.34622E-06	12003	0.001891805	22.75038069
genus.Intestinimonas.id.2062	rs62240188	3	A	G	0.130073573	0.026717884	2.20308E-06	10252	0.002306548	23.70140149
genus.Intestinimonas.id.2062	rs2731794	5	T	C	0.120632118	0.025752925	1.91744E-06	11079	0.001976573	21.94182631
genus.Intestinimonas.id.2062	rs7170984	15	C	T	-0.06581157	0.014076733	2.98356E-06	12090	0.001804638	21.85751427
genus.Intestinimonas.id.2062	rs10262702	7	C	T	0.091801712	0.019488468	2.06073E-06	11766	0.001882344	22.18943078
genus.Intestinimonas.id.2062	rs12566247	1	A	T	0.063708441	0.013535548	2.19054E-06	12076	0.001831147	22.15349599
genus.Intestinimonas.id.2062	rs1859797	7	A	G	0.060367955	0.013179253	4.12235E-06	11766	0.001780036	20.98124721
genus.Intestinimonas.id.2062	rs17067892	8	T	C	0.107193792	0.025001344	6.37926E-06	11544	0.001589883	18.38283763
genus.Intestinimonas.id.2062	rs72982915	2	T	C	0.183157909	0.040274272	4.91157E-06	5591	0.003685555	20.68216308
genus.Intestinimonas.id.2062	rs994794	4	C	G	-0.141904818	0.031507362	7.30503E-06	9250	0.00218815	20.28477518
genus.Intestinimonas.id.2062	rs4113676	20	C	A	-0.218506906	0.049015185	7.41635E-06	3170	0.006230108	19.87325635
genus.Intestinimonas.id.2062	rs9823439	3	C	T	-0.058194711	0.013145465	9.85876E-06	11766	0.00166289	19.59815164
genus.Intestinimonas.id.2062	rs62427239	6	A	C	0.162658132	0.036764706	9.40505E-06	6385	0.003056322	19.57444049
genus.Intestinimonas.id.2062	rs2276760	3	G	A	-0.06852335	0.015254563	7.84062E-06	12081	0.001667439	20.17797798
genus.Intestinimonas.id.2062	rs6934519	6	T	C	0.069237276	0.015115352	8.56956E-06	12089	0.001732606	20.98183294
genus.Intestinimonas.id.2062	rs1000888	13	G	C	-0.058503766	0.01321901	9.71299E-06	12090	0.001617486	19.58708247
genus.Lachnoclostridium.id.11308	rs6112314	20	C	A	-0.056171476	0.010817419	2.43212E-07	17842	0.001508985	26.96400177
genus.Lachnoclostridium.id.11308	rs62285313	3	G	A	0.086420325	0.018156544	1.58331E-06	16991	0.001331582	22.65507018
genus.Lachnoclostridium.id.11308	rs78068103	17	G	A	0.088619929	0.019424795	3.66519E-06	17477	0.001189505	20.81373064
genus.Lachnoclostridium.id.11308	rs615997	3	C	T	0.05117524	0.010649056	2.02677E-06	17911	0.00128771	23.09391481
genus.Lachnoclostridium.id.11308	rs61915992	12	T	A	0.080387577	0.01722087	2.6727E-06	16773	0.001297457	21.7905262
genus.Lachnoclostridium.id.11308	rs789029	18	T	C	-0.06412885	0.013797406	3.75328E-06	17922	0.001203933	21.60289848
genus.Lachnoclostridium.id.11308	rs12566975	1	C	T	-0.04680969	0.010578675	9.57204E-06	17921	0.001091371	19.57983375
genus.Lachnoclostridium.id.11308	rs4738679	8	A	G	-0.052026675	0.011404049	4.41754E-06	17921	0.001160026	20.81296791
genus.Lachnoclostridium.id.11308	rs1031599	3	T	G	-0.078627049	0.017564435	6.31376E-06	17398	0.001150473	20.03897958
genus.Lachnoclostridium.id.11308	rs72829893	17	T	G	0.117472377	0.026810315	5.57766E-06	12404	0.001545376	19.19851709
genus.Lachnoclostridium.id.11308	rs3821998	4	A	C	-0.0864066	0.019251946	6.72052E-06	17920	0.001122842	20.14394734
genus.Lachnoclostridium.id.11308	rs2385421	18	G	A	0.074618572	0.018073408	7.13717E-06	17903	0.000951206	17.04565691
genus.Lachnoclostridium.id.11308	rs1528479	2	A	G	-0.049779849	0.011191926	9.63976E-06	17477	0.001130679	19.78324407
genus.Lachnoclostridium.id.11308	rs1997204	12	C	T	-0.108074801	0.024202203	5.97081E-06	14138	0.001408442	19.9406398
genus.Lachnoclostridium.id.11308	rs62028349	16	C	G	0.046998893	0.010597086	9.17043E-06	17922	0.001096325	19.66990657
genus.Lachnospira.id.2004	rs13157098	5	G	A	-0.076805837	0.015531203	5.99315E-07	15947	0.001531206	24.45559575
genus.Lachnospira.id.2004	rs4923324	11	A	G	-0.061732096	0.013338657	2.43593E-06	16490	0.001297219	21.41893399
genus.Lachnospira.id.2004	rs56791201	2	C	T	0.051822993	0.011069673	2.92823E-06	16486	0.001327649	21.91671153
genus.Lachnospira.id.2004	rs2326833	6	G	C	-0.078495035	0.017109015	4.59648E-06	15632	0.001344731	21.04913885
genus.Lachnospira.id.2004	rs4686798	3	C	T	0.053182605	0.011374743	2.73936E-06	16497	0.001323354	21.86029556
genus.Lachnospira.id.2004	rs159484	4	A	G	0.079466817	0.017699872	6.67622E-06	16487	0.001221122	20.15725482
genus.Lachnospira.id.2004	rs2520509	12	G	A	0.051906257	0.011577959	7.41854E-06	16500	0.001216642	20.09904316
genus.LachnospiraceaeFCS020grou	rs7249113	19	A	G	0.067948308	0.013349587	3.72097E-07	13534	0.001910579	25.90726792
genus.LachnospiraceaeFCS020grou	rs12078956	1	G	C	0.106101007	0.022290421	2.15431E-06	13101	0.001726425	22.6570036
genus.LachnospiraceaeFCS020grou	rs369444	16	G	C	0.125481334	0.025900786	3.14826E-06	12465	0.001879417	23.47104096
genus.LachnospiraceaeFCS020grou	rs72793667	2	G	A	-0.116880798	0.024658906	1.63421E-06	12396	0.001809134	22.46667032
genus.LachnospiraceaeFCS020grou	rs1363769	19	C	T	-0.200628437	0.044937217	1.58163E-06	3555	0.005575769	19.93299886
genus.LachnospiraceaeFCS020grou	rs10093861	8	A	G	-0.056886893	0.012115188	3.05624E-06	13772	0.001598352	22.04774179
genus.LachnospiraceaeFCS020grou	rs9788306	13	T	C	-0.062803973	0.013074422	1.38618E-06	13772	0.00167265	23.07433597
genus.LachnospiraceaeFCS020grou	rs113859143	5	C	G	-0.108906331	0.024205472	2.5545E-06	13238	0.001526839	20.24319863
genus.LachnospiraceaeFCS020grou	rs9919338	1	C	G	-0.055333855	0.012062109	4.90667E-06	13768	0.001526164	21.04434293
genus.LachnospiraceaeFCS020grou	rs35035870	11	C	T	-0.190615272	0.041440269	2.62028E-06	5642	0.003736041	21.1577873
genus.LachnospiraceaeFCS020grou	rs2862811	3	C	T	0.056492728	0.012173604	3.91911E-06	13772	0.001561245	21.53509352
genus.LachnospiraceaeFCS020grou	rs1254846	10	A	G	0.105962631	0.023250118	5.59664E-06	13172	0.001574415	20.77089137
genus.LachnospiraceaeFCS020grou	rs4452603	6	G	T	0.060420825	0.013596453	8.9819E-06	13487	0.00146208	19.74795071
genus.LachnospiraceaeFCS020grou	rs2322265	4	T	C	-0.0666305	0.014157654	5.20809E-06	13772	0.001605716	22.14948049
genus.LachnospiraceaeFCS020grou	rs3999074	10	T	G	-0.05505865	0.012184677	6.55048E-06	13772	0.001480412	20.41846505
genus.LachnospiraceaeFCS020grou	rs9308097	4	G	A	0.05538101	0.012365944	7.4729E-06	13542	0.00147891	20.057056
genus.LachnospiraceaeNC2004grou	rs6116753	20	A	G	0.099474834	0.020913933	2.9225E-06	6278	0.003590637	22.62325245
genus.LachnospiraceaeNC2004grou	rs3756315	5	G	A	-0.088346946	0.01883968	3.32583E-06	6328	0.003463083	21.99054748
genus.LachnospiraceaeNC2004grou	rs12127733	1	A	G	0.115181538	0.024600625	3.10594E-06	6226	0.003508633	21.92166172
genus.LachnospiraceaeNC2004grou	rs17067076	18	A	G	-0.154631362	0.035219374	5.61206E-06	4449	0.004314116	19.27666493
genus.LachnospiraceaeNC2004grou	rs1331592	9	G	C	0.094880626	0.02083688	5.34359E-06	6328	0.003265898	20.73431601
genus.LachnospiraceaeNC2004grou	rs1928659	9	C	T	0.102522036	0.022644542	6.17241E-06	5516	0.003702305	20.49780526
genus.LachnospiraceaeNC2004grou	rs1929743	13	C	T	0.083720631	0.019031847	9.0551E-06	6330	0.003047711	19.35098667
genus.LachnospiraceaeNC2004grou	rs12863463	13	A	G	-0.156364942	0.03453716	6.03984E-06	5516	0.00370229	20.49771871
genus.LachnospiraceaeNC2004grou	rs117467633	17	C	T	-0.169687489	0.038316857	9.13317E-06	5077	0.003848029	19.61191042
genus.LachnospiraceaeNC2004grou	rs12208226	6	A	C	-0.154739126	0.034037233	9.7484E-06	5620	0.003664049	20.66768218
genus.LachnospiraceaeND3007grou	rs9932954	16	G	A	-0.056186355	0.011598415	1.24882E-06	15222	0.001539302	23.46737141
genus.LachnospiraceaeND3007grou	rs13110238	4	G	C	-0.063697828	0.01420413	4.47808E-06	15019	0.001337204	20.110365
genus.LachnospiraceaeND3007grou	rs72776675	10	C	T	-0.064706741	0.014797022	8.71689E-06	15302	0.00124813	19.12275322
genus.LachnospiraceaeND3007grou	rs2861203	3	A	G	0.057230395	0.012727879	7.36931E-06	15305	0.001319273	20.21814852
genus.LachnospiraceaeNK4A136gr	rs12362320	11	C	G	0.057316768	0.011546623	8.04224E-07	16764	0.001467702	24.64072244
genus.LachnospiraceaeNK4A136gr	rs954878	1	G	A	-0.052065624	0.010908221	1.78203E-06	17227	0.00132072	22.78213013
genus.LachnospiraceaeNK4A136gr	rs7832116	8	G	A	-0.071475594	0.015170307	3.57084E-06	17219	0.001287536	22.1986615
genus.LachnospiraceaeNK4A136gr	rs7616165	3	T	G	-0.230541982	0.048346456	2.77391E-06	4094	0.005523539	22.73896804
genus.LachnospiraceaeNK4A136gr	rs76193507	3	G	A	-0.229730157	0.049977561	2.9309E-06	3578	0.005870681	21.12933852
genus.LachnospiraceaeNK4A136gr	rs11263806	17	G	A	-0.052461533	0.011675801	5.06517E-06	17236	0.00116994	20.18870998
genus.LachnospiraceaeNK4A136gr	rs73044693	19	G	A	-0.107579587	0.022988374	3.57256E-06	12773	0.001711616	21.89995614
genus.LachnospiraceaeNK4A136gr	rs160061	5	G	A	0.051383089	0.010809407	2.12198E-06	17234	0.001309428	22.59626496
genus.LachnospiraceaeNK4A136gr	rs7073658	10	G	T	-0.049962145	0.010969284	5.2691E-06	17236	0.001202172	20.74557963
genus.LachnospiraceaeNK4A136gr	rs68104925	14	C	T	-0.054910353	0.011538497	2.36779E-06	17227	0.001312894	22.64696507
genus.LachnospiraceaeNK4A136gr	rs12611395	19	G	A	-0.090253198	0.019965236	5.83039E-06	16486	0.001238007	20.43507785
genus.LachnospiraceaeNK4A136gr	rs2880566	17	C	T	0.059957809	0.013469261	5.61428E-06	17232	0.001148601	19.81545106
genus.LachnospiraceaeNK4A136gr	rs28540839	8	C	A	0.050828488	0.011059105	9.34319E-06	16764	0.00125849	21.12391745
genus.LachnospiraceaeNK4A136gr	rs59805249	5	C	T	0.09361625	0.020798243	9.45385E-06	15195	0.001331588	20.26045375
genus.LachnospiraceaeNK4A136gr	rs10952110	7	T	G	0.048770868	0.010961435	9.08113E-06	16764	0.001179494	19.7963951
genus.LachnospiraceaeNK4A136gr	rs4955932	3	C	T	-0.049228303	0.010938782	7.05426E-06	17236	0.001173668	20.25311545
genus.LachnospiraceaeUCG001.id.	rs437876	3	C	T	0.078463676	0.014476767	7.1655E-08	10150	0.002885845	29.37609989
genus.LachnospiraceaeUCG001.id.	rs985416	3	T	C	0.097025845	0.018180694	1.45591E-07	9917	0.002863704	28.48091522
genus.LachnospiraceaeUCG001.id.	rs2050911	1	A	G	0.075146621	0.015393431	1.10648E-06	10148	0.002342874	23.83131897
genus.LachnospiraceaeUCG001.id.	rs10815577	9	G	C	-0.06837061	0.014369051	1.71503E-06	10149	0.002225829	22.64032946
genus.LachnospiraceaeUCG001.id.	rs573933	9	C	T	-0.107898495	0.023234631	3.10508E-06	10150	0.002120174	21.56548489
genus.LachnospiraceaeUCG001.id.	rs78848836	1	G	A	-0.118867977	0.025975321	3.38171E-06	9506	0.002198135	20.94150471
genus.LachnospiraceaeUCG001.id.	rs9403580	6	T	C	0.107800527	0.022977722	3.46785E-06	10095	0.002175583	22.01039494
genus.LachnospiraceaeUCG001.id.	rs7213933	17	A	T	-0.081626876	0.018380006	9.01671E-06	9961	0.001976119	19.72309349
genus.LachnospiraceaeUCG001.id.	rs8104225	19	G	A	0.089209817	0.019766483	8.04216E-06	9908	0.002051581	20.36884801
genus.LachnospiraceaeUCG001.id.	rs74034332	16	A	G	0.168045475	0.038263689	3.33351E-06	6004	0.003202185	19.28767873
genus.LachnospiraceaeUCG001.id.	rs62496417	7	G	T	-0.074850403	0.016568056	5.87504E-06	10150	0.002006812	20.41010205
genus.LachnospiraceaeUCG001.id.	rs2371284	12	C	T	-0.076189302	0.017012675	7.55967E-06	9961	0.002009399	20.05592793
genus.LachnospiraceaeUCG001.id.	rs12131224	1	T	C	0.117095415	0.025909955	7.39863E-06	9676	0.002106371	20.42426686
genus.LachnospiraceaeUCG001.id.	rs4981345	14	C	T	-0.068197297	0.014983138	6.08596E-06	9961	0.002075504	20.7170902
genus.LachnospiraceaeUCG001.id.	rs79476906	1	A	T	-0.087088012	0.019700842	8.27425E-06	9917	0.001966581	19.54101735
genus.LachnospiraceaeUCG001.id.	rs7341608	8	C	T	-0.078475713	0.017764768	9.48124E-06	9917	0.001963891	19.51423064
genus.LachnospiraceaeUCG004.id.	rs12747809	1	A	G	-0.062197002	0.012568463	8.64875E-07	14464	0.001690252	24.48919858
genus.LachnospiraceaeUCG004.id.	rs2882478	2	A	G	-0.057709061	0.011833839	1.20811E-06	14466	0.00164125	23.78136
genus.LachnospiraceaeUCG004.id.	rs12673420	7	A	G	0.055435065	0.011837515	2.98079E-06	14463	0.00151402	21.93047442
genus.LachnospiraceaeUCG004.id.	rs2444793	6	T	C	-0.054243345	0.011818754	4.77076E-06	14463	0.001454317	21.06442247
genus.LachnospiraceaeUCG004.id.	rs2726805	4	G	A	0.054839706	0.01208444	6.30399E-06	14466	0.001421579	20.59383316
genus.LachnospiraceaeUCG004.id.	rs11128180	3	G	A	0.06482876	0.014012621	4.51737E-06	14466	0.001477427	21.40408712
genus.LachnospiraceaeUCG004.id.	rs12894272	14	G	A	0.057979201	0.012522898	4.33949E-06	14463	0.001479903	21.43555536
genus.LachnospiraceaeUCG004.id.	rs233486	6	G	A	-0.079908769	0.017765984	6.28457E-06	13710	0.00147344	20.2306737
genus.LachnospiraceaeUCG004.id.	rs35182105	12	G	A	-0.109724303	0.024221006	4.86508E-06	13170	0.00155582	20.52207696
genus.LachnospiraceaeUCG004.id.	rs6656451	1	T	C	-0.054359986	0.01194699	5.56781E-06	14458	0.001429921	20.70340123
genus.LachnospiraceaeUCG004.id.	rs4567981	2	A	T	-0.054635262	0.012063179	6.55347E-06	14026	0.00146034	20.51268465
genus.LachnospiraceaeUCG004.id.	rs12072562	1	C	T	0.133103727	0.030356565	7.0737E-06	9176	0.002090802	19.22539108
genus.LachnospiraceaeUCG004.id.	rs2706242	13	C	G	-0.09013993	0.019971219	9.83848E-06	14026	0.001450311	20.3716063
genus.LachnospiraceaeUCG004.id.	rs7629954	3	G	A	0.108440892	0.023824987	5.77222E-06	12568	0.001645657	20.71671156
genus.LachnospiraceaeUCG008.id.	rs10793103	11	T	C	0.097434405	0.018127922	9.34937E-08	7014	0.004101832	28.88874348
genus.LachnospiraceaeUCG008.id.	rs9873555	3	C	G	-0.121161831	0.023346742	2.41343E-07	6577	0.004078274	26.9326492
genus.LachnospiraceaeUCG008.id.	rs13024781	2	C	T	-0.079876878	0.016884555	2.29166E-06	7016	0.00317973	22.38014777

genus.LachnospiraceaeUCG008.id.	rs10741777	11	C	T	-0.097384219	0.019479136	7.68659E-07	7014	0.003550808	24.99411695
genus.LachnospiraceaeUCG008.id.	rs955844	16	C	A	0.112064162	0.022836934	1.80994E-06	6863	0.003496413	24.08007936
genus.LachnospiraceaeUCG008.id.	rs67078837	4	C	T	-0.08458483	0.017069269	7.68189E-07	7016	0.003487773	24.55585804
genus.LachnospiraceaeUCG008.id.	rs62277846	2	T	C	0.102292484	0.021231902	1.58892E-06	7016	0.003297506	23.21184514
genus.LachnospiraceaeUCG008.id.	rs10801803	1	A	G	-0.117029089	0.024308454	1.40029E-06	7017	0.003292224	23.17784022
genus.LachnospiraceaeUCG008.id.	rs61944774	12	G	A	0.179838121	0.039379746	6.34122E-06	6000	0.003463853	20.85536026
genus.LachnospiraceaeUCG008.id.	rs75356640	15	A	G	0.136523454	0.030312828	9.83206E-06	6423	0.003148142	20.28437513
genus.LachnospiraceaeUCG008.id.	rs57254474	8	A	G	0.08865115	0.019904502	6.92061E-06	7017	0.002818958	19.83654865
genus.LachnospiraceaeUCG008.id.	rs57091572	6	G	A	-0.110442734	0.023580145	2.85822E-06	6950	0.003146501	21.93720886
genus.LachnospiraceaeUCG010.id.	rs11192447	10	G	A	0.126592141	0.024345002	4.68957E-07	12695	0.002125383	27.03920202
genus.LachnospiraceaeUCG010.id.	rs9981767	21	C	A	0.065506222	0.01319942	9.96373E-07	12816	0.00191809	24.6294878
genus.LachnospiraceaeUCG010.id.	rs74315802	14	T	G	0.086726244	0.018347717	3.19006E-06	12715	0.001754116	22.3427705
genus.LachnospiraceaeUCG010.id.	rs12346653	9	T	C	0.065768558	0.013956927	2.70321E-06	12816	0.001729628	22.20531968
genus.LachnospiraceaeUCG010.id.	rs10414815	19	C	T	0.104529604	0.023025257	4.23977E-06	11564	0.00177905	20.60960403
genus.LachnospiraceaeUCG010.id.	rs72761829	1	T	A	0.111883594	0.023889286	2.57651E-06	11488	0.001905696	21.93443608
genus.LachnospiraceaeUCG010.id.	rs72894957	2	A	G	0.222262976	0.048642511	5.68428E-06	4316	0.00481421	20.87864332
genus.LachnospiraceaeUCG010.id.	rs336138	5	T	G	0.077954271	0.017186539	7.48417E-06	13236	0.001551928	20.57325242
genus.LachnospiraceaeUCG010.id.	rs4576377	7	C	A	-0.057200131	0.012704307	7.63056E-06	13225	0.001530493	20.27178963
genus.LachnospiraceaeUCG010.id.	rs2833528	21	T	C	-0.056214807	0.012776299	9.9199E-06	13233	0.001460825	19.35937518
genus.LachnospiraceaeUCG010.id.	rs2153460	9	T	A	-0.068403784	0.015656159	9.17312E-06	13236	0.001440148	19.08929097
genus.LachnospiraceaeUCG010.id.	rs17730011	6	A	G	-0.070235733	0.015706022	7.84597E-06	13226	0.001509729	19.99787354
genus.Lactobacillus.id.1837	rs921925	19	C	A	0.098507745	0.020322897	9.71665E-07	6952	0.003368174	23.49468043
genus.Lactobacillus.id.1837	rs16861661	1	A	G	-0.183146977	0.038148013	1.2781E-06	5325	0.004309831	23.04918945
genus.Lactobacillus.id.1837	rs768253	8	G	T	-0.079195015	0.017179101	4.2477E-06	6958	0.00304499	21.25175515
genus.Lactobacillus.id.1837	rs11674854	2	T	C	-0.085268613	0.017650364	1.592E-06	6655	0.003494642	23.33839927
genus.Lactobacillus.id.1837	rs328312	16	A	T	0.081509206	0.016944121	1.40618E-06	6950	0.003318538	23.14063419
genus.Lactobacillus.id.1837	rs6092149	20	T	A	-0.080134426	0.01714896	3.29042E-06	6906	0.003151847	21.83547711
genus.Lactobacillus.id.1837	rs75127669	6	A	C	0.13978419	0.031041446	6.83434E-06	6840	0.002955905	20.2783317
genus.Lactobacillus.id.1837	rs77478751	3	G	A	-0.219885755	0.047575911	7.32705E-06	3669	0.005788301	21.36092052
genus.Lactobacillus.id.1837	rs1530559	2	A	G	0.080400057	0.017820739	4.93109E-06	6655	0.003049207	20.35453946
genus.Lactobacillus.id.1837	rs62314653	4	A	C	0.187691889	0.039458456	2.24383E-06	5944	0.003792119	22.62615905
genus.Lactobacillus.id.1837	rs7399658	13	A	G	-0.107133701	0.022188263	3.12099E-06	6613	0.003513005	23.31340022
genus.Lactobacillus.id.1837	rs12693845	2	T	C	-0.080544718	0.017742876	8.9632E-06	6958	0.002952958	20.60753671
genus.Lactobacillus.id.1837	rs1590596	13	G	C	-0.104805089	0.022172763	4.00765E-06	6655	0.003345966	22.34215763
genus.Lactococcus.id.1851	rs34757988	18	C	G	0.122293892	0.022897605	8.95039E-08	4055	0.006985446	28.52524689
genus.Lactococcus.id.1851	rs757872	22	C	G	0.140799135	0.027581978	4.36882E-07	3792	0.006825062	26.05848489
genus.Lactococcus.id.1851	rs123059	17	C	T	-0.136709792	0.02746936	1.26562E-06	3760	0.006544294	24.76864047
genus.Lactococcus.id.1851	rs4766997	12	T	C	0.114599246	0.023839024	2.06145E-06	4053	0.005669448	23.10928861
genus.Lactococcus.id.1851	rs10417872	19	G	T	0.118305772	0.024521975	1.28678E-06	4056	0.005705818	23.2756054
genus.Lactococcus.id.1851	rs6674304	1	T	C	0.200757712	0.044212138	6.17759E-06	3869	0.005300959	20.61870777
genus.Lactococcus.id.1851	rs7992246	13	C	T	0.104232064	0.023078643	4.44724E-06	4057	0.005002638	20.39774463
genus.Lactococcus.id.1851	rs55910161	10	T	C	0.146425945	0.030736694	2.36214E-06	3836	0.005881409	22.69455931
genus.Lactococcus.id.1851	rs12621813	2	A	G	0.108422776	0.023997845	6.60829E-06	4057	0.005006243	20.41251743
genus.Lactococcus.id.1851	rs2293361	2	T	C	-0.199221089	0.043096892	1.39593E-06	3787	0.005610993	21.36872997
genus.Lactococcus.id.1851	rs17168302	7	A	G	0.191856546	0.042476093	6.28602E-06	4023	0.005045651	20.40159489
genus.Marvinbryantia.id.2005	rs61884471	11	A	G	0.124426313	0.024843116	1.01163E-06	11559	0.002165462	25.08489805
genus.Marvinbryantia.id.2005	rs2724813	10	G	A	-0.084076924	0.01675508	6.28451E-07	11610	0.002164151	25.18028239
genus.Marvinbryantia.id.2005	rs1187983	1	T	C	-0.093545581	0.019317443	2.02427E-06	11896	0.001967393	23.45023931
genus.Marvinbryantia.id.2005	rs2842896	6	T	C	-0.0649396	0.013114646	7.25363E-07	11709	0.00208967	24.51918557
genus.Marvinbryantia.id.2005	rs11645029	16	C	G	-0.060618043	0.013155125	4.14752E-06	11709	0.001810119	21.233121
genus.Marvinbryantia.id.2005	rs12963345	18	C	G	-0.05968101	0.013228853	6.60378E-06	11709	0.001735217	20.35297795
genus.Marvinbryantia.id.2005	rs2863363	3	G	A	0.063486062	0.013632279	3.11281E-06	11950	0.001811609	21.68801622
genus.Marvinbryantia.id.2005	rs72948274	11	C	A	-0.126353989	0.02722118	3.2591E-06	11561	0.001860201	21.5458692
genus.Marvinbryantia.id.2005	rs146541147	19	A	G	0.118845391	0.02684231	6.86288E-06	10778	0.001815505	19.60310754
genus.Marvinbryantia.id.2005	rs11620597	13	C	T	0.119479339	0.027168773	7.80178E-06	11031	0.001750129	19.33951844
genus.Marvinbryantia.id.2005	rs8006832	14	T	G	-0.095241547	0.021669867	6.58083E-06	11412	0.001689834	19.31703099
genus.Marvinbryantia.id.2005	rs3125832	1	C	A	0.067932264	0.015011987	5.02731E-06	11709	0.001745811	20.47744714
genus.Methanobrevibacter.id.123	rs76029318	13	C	T	0.22284888	0.045431939	1.07552E-06	3387	0.00705357	24.06015185
genus.Methanobrevibacter.id.123	rs73457410	13	G	A	0.218128046	0.044466023	1.47328E-06	3387	0.007054668	24.06392328
genus.Methanobrevibacter.id.123	rs10202904	2	G	T	-0.112811092	0.023910624	3.08549E-06	3583	0.006174264	22.25982612
genus.Methanobrevibacter.id.123	rs894996	4	A	C	0.214212565	0.045604463	3.81567E-06	3479	0.006301954	22.06354023
genus.Methanobrevibacter.id.123	rs11018665	11	T	A	0.113013025	0.025434251	7.03099E-06	3586	0.005475507	19.74327098
genus.Methanobrevibacter.id.123	rs1334944	10	C	T	0.115196507	0.025549236	7.6138E-06	3586	0.005637121	20.32931626
genus.Methanobrevibacter.id.123	rs6776814	3	C	T	-0.188956311	0.041990543	8.04905E-06	3283	0.006130253	20.24975739
genus.Methanobrevibacter.id.123	rs4779844	15	C	G	0.109638988	0.024797947	9.28281E-06	3585	0.005423102	19.54783175
genus.Methanobrevibacter.id.123	rs4802933	19	G	A	-0.135628431	0.030813872	9.73818E-06	3586	0.005373517	19.37353717
genus.Odoribacter.id.952	rs503751	15	G	C	0.061829433	0.011888659	2.07625E-07	14218	0.00189872	27.04735613
genus.Odoribacter.id.952	rs77779484	12	A	G	-0.133489405	0.026852607	6.56256E-07	11935	0.002066331	24.71272705
genus.Odoribacter.id.952	rs10423795	19	T	C	0.055069483	0.012116411	6.57936E-06	14135	0.001459298	20.65732131
genus.Odoribacter.id.952	rs6856150	4	A	G	0.088192184	0.019414623	6.0595E-06	14597	0.001411644	20.63489536
genus.Odoribacter.id.952	rs10093869	8	G	A	-0.057779486	0.012538929	3.67441E-06	14218	0.001491214	21.23373901
genus.Odoribacter.id.952	rs4793970	17	G	A	-0.057632938	0.012915412	6.02696E-06	14687	0.001353953	19.91246353
genus.Odoribacter.id.952	rs74553962	8	G	T	0.121448819	0.026410563	9.48684E-06	11803	0.001788387	21.14614835
genus.Odoribacter.id.952	rs28417404	14	G	A	-0.072689645	0.016137195	3.68032E-06	14698	0.001378579	20.29032375
genus.Odoribacter.id.952	rs16918425	11	T	A	0.099978081	0.022378451	8.85048E-06	13689	0.001455945	19.95949445
genus.Olsenella.id.822	rs1035588	2	G	A	-0.108148115	0.023684753	4.85501E-06	3739	0.00554536	20.84971866
genus.Olsenella.id.822	rs62112538	19	T	C	-0.199430349	0.040703259	1.1882E-06	3630	0.006569841	24.00623954
genus.Olsenella.id.822	rs35225860	1	G	A	-0.223603655	0.048239131	3.87041E-06	3630	0.005884224	21.48616242
genus.Olsenella.id.822	rs72691585	9	A	C	-0.249078856	0.052081416	2.95294E-06	3078	0.007376057	22.87221169
genus.Olsenella.id.822	rs9460691	6	A	C	0.119965803	0.026864494	7.28354E-06	3739	0.005305081	19.94148949
genus.Olsenella.id.822	rs17148768	10	A	G	0.140434164	0.029560175	2.1984E-06	3648	0.006148909	22.56999959
genus.Olsenella.id.822	rs61090148	4	G	A	-0.104782706	0.023134258	6.43728E-06	3739	0.005456778	20.51483771
genus.Olsenella.id.822	rs7540303	1	T	C	0.108043794	0.023637952	5.32027E-06	3739	0.005556542	20.8919971
genus.Olsenella.id.822	rs2759329	1	A	G	-0.111131408	0.02372184	3.43201E-06	3739	0.005835521	21.94708653
genus.Olsenella.id.822	rs6046522	20	T	C	0.12293822	0.026992758	4.48455E-06	3705	0.005567579	20.74337068
genus.Olsenella.id.822	rs8066522	17	A	G	-0.106528718	0.02403789	9.70095E-06	3739	0.005225285	19.63996473
genus.Oscillibacter.id.2063	rs234108	1	G	A	0.074955279	0.015263201	9.16336E-07	8694	0.002766244	24.11643885
genus.Oscillibacter.id.2063	rs36095275	14	T	C	-0.07523683	0.015686113	1.39627E-06	8694	0.002639143	23.00542565
genus.Oscillibacter.id.2063	rs11627628	14	C	T	0.143960728	0.029022314	1.0092E-06	8486	0.002891103	24.60503621
genus.Oscillibacter.id.2063	rs9393920	6	G	A	-0.074466242	0.015108116	9.92237E-07	8930	0.002713107	24.29395645
genus.Oscillibacter.id.2063	rs133832	22	C	A	-0.079552688	0.016241059	1.14719E-06	8926	0.002680763	23.99280554
genus.Oscillibacter.id.2063	rs12649930	4	G	T	0.121588701	0.025961173	4.09379E-06	8794	0.002488109	21.93500816
genus.Oscillibacter.id.2063	rs75453768	10	T	G	0.122120078	0.026862946	5.34738E-06	8256	0.002496959	20.66649721
genus.Oscillibacter.id.2063	rs16866406	2	G	A	0.098876509	0.02087954	3.07821E-06	8930	0.002504977	22.42561711
genus.Oscillibacter.id.2063	rs761240	20	G	T	-0.176639573	0.038881196	2.03841E-06	5185	0.003964813	20.63938601
genus.Oscillibacter.id.2063	rs4506202	8	G	A	-0.071132334	0.01522611	3.20825E-06	8694	0.002504077	21.82509762
genus.Oscillibacter.id.2063	rs61883564	11	G	A	-0.1013514	0.022101565	3.38712E-06	8930	0.002349311	21.02875037
genus.Oscillibacter.id.2063	rs16934185	9	G	A	-0.129567532	0.028156999	4.38031E-06	8492	0.002487299	21.1748152
genus.Oscillibacter.id.2063	rs12417956	11	G	C	0.078543098	0.017407454	6.02626E-06	8928	0.002275109	20.35848922
genus.Oscillibacter.id.2063	rs6901560	6	G	C	0.085503217	0.018693038	6.20951E-06	8525	0.002448196	20.92209296
genus.Oscillibacter.id.2063	rs62206502	20	A	C	-0.068142274	0.015134881	6.59895E-06	8883	0.002276804	20.27100281
genus.Oscillibacter.id.2063	rs137917150	12	A	T	-0.174992153	0.038839403	4.61985E-06	5733	0.003528378	20.29981383
genus.Oscillibacter.id.2063	rs11990279	8	C	T	-0.082493414	0.018045748	4.94298E-06	8528	0.002444436	20.89723226
genus.Oscillospira.id.2064	rs12206468	6	A	G	-0.133015789	0.026973335	1.04222E-06	9554	0.002538913	24.31851752
genus.Oscillospira.id.2064	rs73038677	3	A	T	-0.083210036	0.016866557	1.0943E-06	10195	0.002381638	24.33876433
genus.Oscillospira.id.2064	rs1954532	14	C	T	-0.082624583	0.017525166	2.26773E-06	9841	0.002253592	22.22768659
genus.Oscillospira.id.2064	rs28889936	4	C	A	0.114050943	0.025283847	3.36585E-06	9758	0.002080875	20.34751908
genus.Oscillospira.id.2064	rs751183	1	C	T	-0.07743224	0.017221553	6.85127E-06	9841	0.002050069	20.21617744
genus.Oscillospira.id.2064	rs8076323	17	G	A	0.07153922	0.015653872	5.61316E-06	10193	0.002044814	20.88549958
genus.Oscillospira.id.2064	rs72866977	6	C	A	-0.130569033	0.028167414	5.62701E-06	9098	0.002356221	21.48752689
genus.Oscillospira.id.2064	rs62422654	6	T	C	0.089845572	0.019808815	6.46754E-06	9769	0.002101419	20.57199247
genus.Oscillospira.id.2064	rs12925026	16	C	T	0.135590113	0.030677984	9.31021E-06	8200	0.002376595	19.53450499
genus.Oxalobacter.id.2978	rs4428215	3	A	G	0.130292636	0.0242237	7.51071E-08	4655	0.006176584	28.93068902
genus.Oxalobacter.id.2978	rs736744	9	T	C	0.117882083	0.021126239	2.57471E-08	4655	0.006644104	31.13516917
genus.Oxalobacter.id.2978	rs6000536	22	T	C	-0.130991749	0.025380356	2.06054E-07	4654	0.005690985	26.63743827
genus.Oxalobacter.id.2978	rs36057338	4	T	G	0.207847028	0.042143922	8.79803E-07	4244	0.005698499	24.32303343
genus.Oxalobacter.id.2978	rs6071435	20	A	T	-0.105512319	0.021488964	1.07431E-06	4635	0.005174549	24.10878852
genus.Oxalobacter.id.2978	rs12002250	9	C	A	0.217122385	0.046631729	1.41504E-06	4297	0.005019902	21.67934501
genus.Oxalobacter.id.2978	rs1569853	6	C	T	-0.138077533	0.029698126	3.64501E-06	4492	0.004789203	21.61662727
genus.Oxalobacter.id.2978	rs11108500	12	G	A	-0.199099074	0.04273266	3.74279E-06	4303	0.005019516	21.7079404
genus.Oxalobacter.id.2978	rs10464997	8	A	G	0.137690928	0.029480352	3.29754E-06	4650	0.004669386	21.81450348
genus.Oxalobacter.id.2978	rs111966731	15	C	T	0.213114454	0.047162014	7.29863E-06	3931	0.00516759	20.41931321
genus.Oxalobacter.id.2978	rs6993398	8	A	G	0.127217146	0.027885461	7.12773E-06	4656	0.004450262	20.81304564
genus.Oxalobacter.id.2978	rs3862635	11	T	C	-0.172142003	0.039402609	9.18685E-06	4469	0.004252678	19.08638791
genus.Parabacteroides.id.954	rs60884758	9	T	C	-0.07026666	0.014224884	5.70689E-07	17293	0.001409024	24.400635
genus.Parabacteroides.id.954	rs3860755	5	C	G	0.055878381	0.011662454	1.71325E-06	17294	0.001325672	22.95661007
genus.Parabacteroides.id.954	rs4236095	6	A	G	0.076196085	0.015704151	1.93145E-06	16383	0.001434892	23.54162284
genus.Parabacteroides.id.954	rs10119172	9	G	C	-0.067853591	0.01455377	2.61899E-06	17299	0.001254955	21.73675092
genus.Parabacteroides.id.954	rs115602804	3	A	G	0.10308013	0.02227358	1.93059E-06	16394	0.001304722	21.41755435

genus.Parabacteroides.id.954	rs114567323	3	C	T	0.186477784	0.040519936	5.65487E-06	5859	0.003601854	21.17954842
genus.Parabacteroides.id.954	rs7298818	12	T	C	0.088883151	0.02009019	8.53629E-06	16510	0.001184157	19.57360457
genus.Parabacteroides.id.954	rs6657302	1	C	T	-0.104519671	0.022552116	9.75699E-06	16552	0.001296008	21.47936698
genus.Parabacteroides.id.954	rs11965579	6	C	G	0.16282395	0.038186321	8.86592E-06	3890	0.00465207	18.18113222
genus.Parabacteroides.id.954	rs17141986	16	C	G	0.050030286	0.011145925	7.2741E-06	16825	0.001196076	20.14808304
genus.Parabacteroides.id.954	rs72893646	2	T	A	-0.071559422	0.015666777	8.83391E-06	16508	0.001262209	20.8628844
genus.Paraprevotella.id.962	rs2081023	5	G	A	-0.122560702	0.023650724	2.638E-07	7178	0.003727249	26.8542853
genus.Paraprevotella.id.962	rs9900242	17	G	A	-0.085296669	0.017521167	1.14068E-06	6900	0.003422945	23.69943998
genus.Paraprevotella.id.962	rs9602779	13	C	A	-0.10669184	0.02202635	6.92746E-07	7177	0.003258494	23.46266494
genus.Paraprevotella.id.962	rs140997932	3	C	T	-0.162375604	0.035417814	2.11119E-06	6187	0.003385673	21.01832279
genus.Paraprevotella.id.962	rs145020347	11	G	A	-0.124650032	0.026232336	4.03009E-06	6859	0.003281125	22.57931978
genus.Paraprevotella.id.962	rs4767113	12	T	C	0.088246646	0.018381962	2.14357E-06	6900	0.003329012	23.04690816
genus.Paraprevotella.id.962	rs3008582	1	C	T	0.105720143	0.022724571	4.35989E-06	7175	0.00300742	21.64333017
genus.Paraprevotella.id.962	rs4756632	11	T	G	-0.138905488	0.028989683	3.81747E-06	7179	0.003187875	22.95894252
genus.Paraprevotella.id.962	rs10842464	12	C	T	-0.075821491	0.017256778	6.59715E-06	7178	0.002682226	19.30479532
genus.Paraprevotella.id.962	rs3801748	7	A	G	0.077972078	0.01716912	5.20177E-06	7179	0.002864655	20.6244396
genus.Paraprevotella.id.962	rs7240324	18	G	T	-0.102290408	0.022694788	5.95902E-06	6900	0.002935565	20.31503453
genus.Paraprevotella.id.962	rs17109926	12	G	A	-0.098833506	0.021617178	6.75279E-06	7179	0.002903246	20.90308678
genus.Paraprevotella.id.962	rs17785622	6	G	A	0.248065378	0.052430396	1.92659E-06	3288	0.006762191	22.38546029
genus.Parasutterella.id.2892	rs2387977	20	C	T	-0.068177776	0.013475556	5.38476E-07	11345	0.002251174	25.59719025
genus.Parasutterella.id.2892	rs7572229	2	A	G	0.066272974	0.013273581	6.31698E-07	11383	0.002185194	24.92853273
genus.Parasutterella.id.2892	rs35414597	4	A	T	-0.068494619	0.01421433	1.51206E-06	11386	0.002035186	23.21988734
genus.Parasutterella.id.2892	rs6828768	4	T	C	0.063685185	0.013264516	1.78328E-06	11386	0.002020433	23.05122373
genus.Parasutterella.id.2892	rs10899911	10	G	A	-0.071710162	0.014815144	1.15272E-06	11386	0.002053458	23.42877745
genus.Parasutterella.id.2892	rs7303158	12	T	C	0.064685912	0.013425592	1.32776E-06	11386	0.002034683	23.21413477
genus.Parasutterella.id.2892	rs78383039	2	C	T	-0.146314647	0.029711928	1.57438E-06	9174	0.002636384	24.25012192
genus.Parasutterella.id.2892	rs35055552	8	C	T	0.109554176	0.023543268	3.34853E-06	9970	0.00216714	21.65331401
genus.Parasutterella.id.2892	rs2090816	6	C	A	0.084096728	0.017731494	2.89715E-06	11385	0.001971866	22.49404428
genus.Parasutterella.id.2892	rs1403396	12	T	A	-0.075672871	0.015903462	2.75519E-06	11378	0.001985948	22.64107662
genus.Parasutterella.id.2892	rs55877868	17	C	A	-0.10445781	0.022808932	2.87068E-06	10588	0.001976963	20.97354878
genus.Parasutterella.id.2892	rs8039785	15	G	T	0.061834541	0.013300041	3.62095E-06	11383	0.00189529	21.61505264
genus.Parasutterella.id.2892	rs823424	8	A	G	-0.071344819	0.015695821	4.95295E-06	11386	0.001811333	20.66125924
genus.Parasutterella.id.2892	rs7311004	12	C	T	-0.061752212	0.01364384	5.92054E-06	11384	0.001796206	20.48480291
genus.Parasutterella.id.2892	rs62273907	3	G	A	0.229468131	0.050226185	5.88384E-06	3691	0.0056233	20.87297631
genus.Parasutterella.id.2892	rs6809952	3	A	G	-0.068495728	0.015089156	8.13349E-06	11077	0.001856811	20.60616107
genus.Parasutterella.id.2892	rs11715853	3	A	G	-0.066295189	0.014611398	6.22913E-06	11386	0.001804782	20.58640271
genus.Peptococcus.id.2037	rs75754569	3	G	C	0.181434144	0.031942874	1.09884E-08	5481	0.005851692	32.26190937
genus.Peptococcus.id.2037	rs77681628	13	T	C	0.200307214	0.038732756	2.68836E-07	4842	0.005493124	26.74461664
genus.Peptococcus.id.2037	rs10031059	4	C	T	-0.121166531	0.022584403	1.23637E-07	5526	0.005181804	28.78380029
genus.Peptococcus.id.2037	rs6918730	6	A	G	0.135310568	0.028974201	1.14732E-06	5656	0.003841136	21.80923879
genus.Peptococcus.id.2037	rs413827	14	A	G	0.11022947	0.023752348	3.30085E-06	5482	0.003913276	21.53685774
genus.Peptococcus.id.2037	rs2054133	2	A	G	0.089543257	0.018833153	2.14166E-06	5657	0.003980169	22.60578976
genus.Peptococcus.id.2037	rs11001941	10	A	G	-0.195611127	0.039221681	1.32858E-06	5607	0.004416536	24.87337395
genus.Peptococcus.id.2037	rs7033353	9	G	T	0.090152001	0.018995009	2.21957E-06	5657	0.003966064	22.52535881
genus.Peptococcus.id.2037	rs5770862	22	C	T	0.162017845	0.035681339	3.21957E-06	5431	0.003781969	20.61785183
genus.Peptococcus.id.2037	rs34282744	15	C	G	0.19179739	0.03998324	1.83535E-06	5257	0.004358074	23.0106778
genus.Peptococcus.id.2037	rs72850165	11	C	T	-0.134304018	0.030042318	5.74152E-06	5627	0.003539113	19.98532053
genus.Peptococcus.id.2037	rs11030569	11	T	A	-0.174020126	0.037413336	3.12991E-06	5329	0.004043345	21.63446116
genus.Peptococcus.id.2037	rs7766680	6	C	G	0.09768317	0.021415492	3.50768E-06	5655	0.003665691	20.80574916
genus.Peptococcus.id.2037	rs74592222	1	A	G	0.137957011	0.030296488	8.55342E-06	5329	0.003875884	20.73494943
genus.Peptococcus.id.2037	rs36121075	20	G	A	-0.140670082	0.030627993	6.9872E-06	5301	0.003963545	21.09436232
genus.Peptococcus.id.2037	rs12069354	1	T	C	0.167629782	0.037950049	9.28339E-06	5352	0.003632296	19.51091661
genus.Phascolarctobacterium.id.216	rs75882962	12	C	T	0.096866054	0.019057501	3.18987E-07	10710	0.002406442	25.83516844
genus.Phascolarctobacterium.id.216	rs56157888	4	C	A	0.095486383	0.019397634	1.08506E-06	10031	0.002409868	24.23178393
genus.Phascolarctobacterium.id.216	rs56069061	3	A	G	-0.111305825	0.02306943	1.87174E-06	11007	0.002110453	23.27887981
genus.Phascolarctobacterium.id.216	rs74540770	3	A	G	-0.121014393	0.025862072	3.60275E-06	9403	0.002323116	21.89512423
genus.Phascolarctobacterium.id.216	rs76124218	17	G	C	-0.159360781	0.03445316	2.66969E-06	7314	0.00291663	21.39462932
genus.Phascolarctobacterium.id.216	rs6427992	1	C	G	-0.065245772	0.013750826	2.08614E-06	11113	0.002021794	22.51371543
genus.Phascolarctobacterium.id.216	rs12618201	2	G	A	0.06416571	0.013820192	3.37696E-06	11113	0.001935998	21.55647908
genus.Phascolarctobacterium.id.216	rs74847270	22	G	A	-0.104889022	0.023099793	5.73099E-06	11098	0.001854356	20.61787516
genus.Phascolarctobacterium.id.216	rs1264476	8	G	T	0.076733542	0.016604148	4.29567E-06	11104	0.001919656	21.35685483
genus.Phascolarctobacterium.id.216	rs28525131	14	A	G	-0.118657158	0.026904831	8.22677E-06	10457	0.001856578	19.45034632
genus.Phascolarctobacterium.id.216	rs7982713	13	A	G	0.072692152	0.016320086	9.72164E-06	10710	0.001849	19.83947511
genus.Phascolarctobacterium.id.216	rs11929846	4	C	T	-0.069719918	0.015796922	8.88416E-06	11114	0.001749596	19.47909532
genus.Prevotella7.id.11182	rs430270	3	C	A	0.13915522	0.029705527	2.86846E-06	3318	0.0065703	21.9444385
genus.Prevotella7.id.11182	rs57404562	2	A	C	0.15548647	0.031605691	6.21576E-07	3487	0.006892849	24.20218769
genus.Prevotella7.id.11182	rs9959718	18	A	G	0.133012021	0.027536133	1.90058E-06	3489	0.006643243	23.33328507
genus.Prevotella7.id.11182	rs9608249	22	G	A	-0.158213329	0.033631703	2.06687E-06	3482	0.006315506	22.13035717
genus.Prevotella7.id.11182	rs2240542	2	T	C	0.120849888	0.026177616	4.84146E-06	3489	0.006071368	21.31239833
genus.Prevotella7.id.11182	rs2918132	10	T	C	-0.11464837	0.025491556	6.42409E-06	3318	0.006059365	20.22753983
genus.Prevotella7.id.11182	rs16937247	11	C	G	0.146097858	0.035152923	9.63705E-06	3486	0.004930499	17.27288205
genus.Prevotella7.id.11182	rs118038478	16	G	A	0.205675553	0.046891721	7.85422E-06	3295	0.005804826	19.23857911
genus.Prevotella7.id.11182	rs12124567	1	G	A	-0.121281313	0.027501731	9.48982E-06	3283	0.005888867	19.44767635
genus.Prevotella7.id.11182	rs12195431	6	C	T	0.196527815	0.044232188	8.72818E-06	3489	0.005626261	19.74109369
genus.Prevotella7.id.11182	rs9426434	1	C	T	-0.123657205	0.027851446	9.71711E-06	3318	0.005906014	19.71257558
genus.Prevotella7.id.11182	rs79263163	11	C	A	-0.143981995	0.031500978	7.51048E-06	3489	0.00595216	20.89143679
genus.Prevotella9.id.11183	rs111509883	19	C	T	0.171130632	0.03476241	1.24389E-06	6960	0.0034699	24.23459413
genus.Prevotella9.id.11183	rs2683313	8	G	A	-0.072457582	0.015160279	1.68895E-06	10260	0.002221468	22.84300709
genus.Prevotella9.id.11183	rs117271932	22	G	A	0.20808857	0.044039277	2.81722E-06	4878	0.004556077	22.32626554
genus.Prevotella9.id.11183	rs10512344	9	G	C	0.247372806	0.054408651	3.19324E-06	3063	0.006703474	20.67130991
genus.Prevotella9.id.11183	rs9428102	1	G	A	-0.07788445	0.017606807	4.61734E-06	10264	0.001902815	19.56772444
genus.Prevotella9.id.11183	rs746764	20	C	T	-0.091577152	0.019324484	2.03999E-06	9385	0.002387186	22.4573476
genus.Prevotella9.id.11183	rs16966465	15	C	G	0.074334818	0.01652906	9.33202E-06	10261	0.001967177	20.22498608
genus.Prevotella9.id.11183	rs2104588	10	C	T	0.105558491	0.023773023	8.12987E-06	9268	0.002122798	19.71594119
genus.Prevotella9.id.11183	rs11199734	10	T	A	0.077020901	0.016946218	7.00288E-06	10263	0.002008741	20.65720745
genus.Prevotella9.id.11183	rs7232121	18	C	G	0.067129188	0.014422959	3.76013E-06	9790	0.00220786	21.66277332
genus.Prevotella9.id.11183	rs11685699	2	T	C	-0.141357386	0.029566474	2.02552E-06	8342	0.002732621	22.85798544
genus.Prevotella9.id.11183	rs9613013	22	A	G	0.091759081	0.020269664	6.10415E-06	9790	0.002088883	20.49297454
genus.Prevotella9.id.11183	rs2495052	1	G	A	0.083897846	0.018852315	8.97142E-06	9790	0.002018886	19.80488116
genus.Prevotella9.id.11183	rs72815774	2	C	T	-0.176185788	0.039306766	8.78291E-06	5728	0.003495292	20.09125724
genus.Prevotella9.id.11183	rs4968431	17	T	G	0.064011283	0.014416226	8.58441E-06	10271	0.001915862	19.71559507
genus.Prevotella9.id.11183	rs7976209	12	C	T	-0.087078586	0.019795052	7.28343E-06	10263	0.001881989	19.35126901
genus.Prevotella9.id.11183	rs12648235	4	C	T	0.078590196	0.017767553	7.39079E-06	10271	0.001901263	19.56507476
genus.Prevotella9.id.11183	rs1304512	5	A	G	0.076159345	0.016588486	5.29082E-06	9790	0.002148405	21.07817083
genus.Prevotella9.id.11183	rs7237249	18	T	C	-0.082376407	0.018212594	8.93303E-06	10271	0.001987857	20.45794798
genus.RikenellaceaeRC9gutgroup.i	rs2900503	9	T	G	-0.172329153	0.032667127	1.55282E-07	3320	0.008312514	27.82887364
genus.RikenellaceaeRC9gutgroup.i	rs17582787	4	G	A	-0.157739395	0.033992444	3.55377E-06	3320	0.006444211	21.53354647
genus.RikenellaceaeRC9gutgroup.i	rs7113155	11	C	G	0.114422634	0.024779825	5.26481E-06	3334	0.006354673	21.32197453
genus.RikenellaceaeRC9gutgroup.i	rs4270579	4	A	G	-0.118021261	0.027104126	5.45647E-06	3331	0.005659916	18.96049606
genus.RikenellaceaeRC9gutgroup.i	rs2998141	10	C	T	-0.136346427	0.029307262	4.42373E-06	3330	0.006457719	21.6439748
genus.RikenellaceaeRC9gutgroup.i	rs4717843	7	T	G	-0.119376102	0.026054834	4.7173E-06	3220	0.006477094	20.99221018
genus.RikenellaceaeRC9gutgroup.i	rs9887954	1	A	G	-0.114849386	0.024901293	4.80801E-06	3330	0.006347517	21.27225678
genus.RikenellaceaeRC9gutgroup.i	rs7193937	16	C	G	0.12415332	0.027741031	6.18767E-06	3220	0.006181908	20.02956363
genus.RikenellaceaeRC9gutgroup.i	rs80309088	6	A	G	0.174049957	0.038342318	4.55642E-06	3170	0.006458296	20.60587785
genus.RikenellaceaeRC9gutgroup.i	rs12501673	4	G	A	0.116395371	0.026225344	6.28983E-06	3334	0.005873609	19.69831328
genus.RikenellaceaeRC9gutgroup.i	rs17032291	4	C	T	-0.169578595	0.036825126	6.61004E-06	3206	0.006570931	21.20574776
genus.RikenellaceaeRC9gutgroup.i	rs2074881	19	C	T	-0.142235956	0.032390968	9.44772E-06	3315	0.005783204	19.28283687
genus.RikenellaceaeRC9gutgroup.i	rs7712231	5	G	A	0.156283835	0.035046199	7.97036E-06	3334	0.005929226	19.88594669
genus.Romboutsia.id.11347	rs61841503	10	A	G	0.092887978	0.017145406	3.99597E-08	13568	0.002158585	29.35103902
genus.Romboutsia.id.11347	rs10279978	7	G	A	-0.062220227	0.012767535	1.17009E-06	14143	0.001676405	23.74920613
genus.Romboutsia.id.11347	rs16843578	1	T	C	-0.087527594	0.019677862	5.08123E-06	14186	0.001392736	19.78491418
genus.Romboutsia.id.11347	rs77702691	13	G	A	-0.094405983	0.020854443	7.36973E-06	12487	0.001638444	20.49282493
genus.Romboutsia.id.11347	rs75987356	2	A	G	-0.129578364	0.028045444	6.70799E-06	10466	0.002035517	21.34717177
genus.Romboutsia.id.11347	rs75200530	8	G	T	-0.190644379	0.042106493	5.06555E-06	5246	0.003892493	20.49981158
genus.Romboutsia.id.11347	rs9567264	13	T	C	0.058001807	0.01274947	5.75689E-06	14180	0.001457435	20.69658963
genus.Romboutsia.id.11347	rs34302036	7	G	A	0.055026128	0.012083475	5.87667E-06	14185	0.00145979	20.73739485
genus.Romboutsia.id.11347	rs7109293	11	G	A	0.091911164	0.020573834	6.98354E-06	14141	0.001409332	19.95749666
genus.Romboutsia.id.11347	rs114398731	5	C	G	-0.13103857	0.029381546	7.95851E-06	10326	0.001922565	19.89065148
genus.Romboutsia.id.11347	rs28603357	7	C	T	-0.214910996	0.047473928	8.52235E-06	3693	0.005518541	20.49306559
genus.Romboutsia.id.11347	rs11221428	11	C	T	-0.072677936	0.015826331	6.48646E-06	14187	0.001484257	21.08845115
genus.Romboutsia.id.11347	rs9389266	6	G	T	0.072281496	0.016235341	9.38461E-06	14187	0.001395194	19.82126873
genus.Romboutsia.id.11347	rs62504452	7	G	A	-0.071004771	0.015665426	4.65831E-06	14178	0.001446928	20.5442677
genus.Roseburia.id.2012	rs2034589	16	C	G	0.062976455	0.012348898	5.01161E-07	17846	0.001455213	26.0075827
genus.Roseburia.id.2012	rs116270582	4	A	T	-0.153843918	0.032932399	1.19525E-06	9099	0.002392654	21.82297307
genus.Roseburia.id.2012	rs2160994	12	C	T	0.055068659	0.011248106	9.6985E-07	17444	0.00137217	23.96901457
genus.Roseburia.id.2012	rs16910295	11	C	T	-0.098043368	0.020956968	2.91111E-06	16648	0.001312946	21.88666302
genus.Roseburia.id.2012	rs12740451	1	C	T	0.069752736	0.015360737	7.34264E-06	17837	0.001154715	20.62046162
genus.Roseburia.id.2012	rs6445851	3	A	G	-0.04973359	0.010815826	3.52721E-06	17851	0.001183051	21.14366514
genus.Roseburia.id.2012	rs9300744	13	T	C	-0.058845175	0.012622789	4.75118E-06	17854	0.001215758	21.73256759

genus.Roseburia.id.2012	rs2943022	5	C	T	0.049378559	0.010676336	4.11408E-06	17854		0.001196677	21.39106283
genus.Roseburia.id.2012	rs6930661	6	T	C	-0.096158735	0.020497475	2.47942E-06	16945		0.001297094	22.00780606
genus.Roseburia.id.2012	rs4748237	10	C	G	0.048832628	0.010640433	4.67223E-06	17851		0.001178493	21.06209699
genus.Roseburia.id.2012	rs147990086	1	G	A	-0.057893629	0.013239999	8.92942E-06	17840		0.001070596	19.11990247
genus.Roseburia.id.2012	rs329182	5	C	T	0.069032724	0.015288408	5.90142E-06	17854		0.001140655	20.38850981
genus.Roseburia.id.2012	rs302266	1	C	T	-0.077731153	0.01729852	8.12587E-06	17056		0.001182446	20.19167353
genus.Roseburia.id.2012	rs75326254	6	T	C	-0.104627797	0.023089702	7.50204E-06	15893		0.001290301	20.53324171
genus.Roseburia.id.2012	rs55858165	15	C	A	0.17928439	0.040495413	9.98975E-06	5447		0.003585551	19.60077669
genus.Roseburia.id.2012	rs57466170	15	T	C	0.074140517	0.017160095	8.30102E-06	17333		0.001075798	18.66688486
genus.Roseburia.id.2012	rs78753150	5	C	A	0.096873855	0.021406676	9.98312E-06	16303		0.00125459	20.47928048
genus.Ruminiclostridium5.id.11355	rs243585	21	G	C	-0.058593933	0.012101692	1.32681E-06	16906		0.001384747	23.44299424
genus.Ruminiclostridium5.id.11355	rs2286384	12	C	G	-0.051881398	0.010748225	1.43857E-06	17394		0.001337732	23.29967484
genus.Ruminiclostridium5.id.11355	rs79968837	20	G	A	-0.095031084	0.019350746	1.14623E-06	16602		0.001450591	24.11770206
genus.Ruminiclostridium5.id.11355	rs2482038	10	A	C	0.051899121	0.010877683	1.70314E-06	17396		0.001306863	22.76392999
genus.Ruminiclostridium5.id.11355	rs113753996	5	C	T	0.082068892	0.017446254	3.98895E-06	16680		0.001324893	22.1285398
genus.Ruminiclostridium5.id.11355	rs1492620	6	C	T	-0.083050484	0.018007297	3.52577E-06	17397		0.001221187	21.27096963
genus.Ruminiclostridium5.id.11355	rs2791343	8	C	T	0.051717025	0.011336879	5.53959E-06	17392		0.001195119	20.81038018
genus.Ruminiclostridium5.id.11355	rs10827477	10	G	A	-0.054738653	0.011516344	2.19177E-06	17391		0.00129739	22.59222351
genus.Ruminiclostridium5.id.11355	rs6121460	20	A	G	0.09329552	0.019920597	2.64107E-06	17318		0.001264939	21.93395175
genus.Ruminiclostridium5.id.11355	rs2801960	1	G	C	0.052097504	0.011511124	6.20872E-06	17387		0.001176691	20.48322635
genus.Ruminiclostridium5.id.11355	rs8053158	16	G	A	-0.074054329	0.015915101	5.89806E-06	17397		0.00124299	21.65120579
genus.Ruminiclostridium5.id.11355	rs1223978	13	C	T	0.048416888	0.010825421	8.16186E-06	17381		0.001149555	20.00341445
genus.Ruminiclostridium5.id.11355	rs73002572	2	C	G	0.181547526	0.041407845	8.8222E-06	3761		0.005085083	19.22274556
genus.Ruminiclostridium5.id.11355	rs2833828	21	A	G	0.048961687	0.010870209	6.81651E-06	17397		0.001164814	20.28789408
genus.Ruminiclostridium5.id.11355	rs4955951	3	G	A	-0.07137494	0.01658264	9.95519E-06	17397		0.00106377	18.52610594
genus.Ruminiclostridium6.id.11356	rs1756364	14	T	G	0.099918929	0.019716857	2.53654E-07	12933		0.001981798	25.68148781
genus.Ruminiclostridium6.id.11356	rs71414120	14	G	T	0.200894087	0.040643398	1.07697E-06	5567		0.004369495	24.43173427
genus.Ruminiclostridium6.id.11356	rs61060922	16	G	T	0.159129692	0.032228344	1.08647E-06	8955		0.002715064	24.37959391
genus.Ruminiclostridium6.id.11356	rs79968172	1	A	G	0.116139597	0.02430834	1.66054E-06	12802		0.001779912	22.82706248
genus.Ruminiclostridium6.id.11356	rs77193512	11	G	A	0.073653265	0.015316349	1.29587E-06	13380		0.001725311	23.12456268
genus.Ruminiclostridium6.id.11356	rs11992182	8	C	A	0.06252993	0.013787562	4.64839E-06	13381		0.001534777	20.56842033
genus.Ruminiclostridium6.id.11356	rs72991535	18	G	T	0.135603894	0.029517443	4.95367E-06	9642		0.002184088	21.10507348
genus.Ruminiclostridium6.id.11356	rs663262	11	C	T	-0.134980385	0.03107145	3.39417E-06	7876		0.002390414	18.87201549
genus.Ruminiclostridium6.id.11356	rs9555756	13	C	A	-0.080413824	0.017669194	7.10127E-06	13369		0.00154688	20.71228253
genus.Ruminiclostridium6.id.11356	rs792058	2	A	G	0.055423396	0.012542318	8.57953E-06	12933		0.001507565	19.52678061
genus.Ruminiclostridium6.id.11356	rs10829821	10	C	T	-0.097605954	0.021607241	3.47141E-06	12802		0.001591419	20.40581769
genus.Ruminiclostridium6.id.11356	rs67479537	19	C	T	0.118994191	0.026487011	9.302E-06	11613		0.00173495	20.18299463
genus.Ruminiclostridium6.id.11356	rs77968078	1	A	G	0.119199655	0.02497032	2.62666E-06	12602		0.001805002	22.7877669
genus.Ruminiclostridium6.id.11356	rs1871858	15	G	C	-0.105421541	0.023727442	9.11606E-06	12818		0.001537688	19.74044168
genus.Ruminiclostridium6.id.11356	rs116969552	10	G	A	-0.166822503	0.037668056	9.15949E-06	6824		0.002866008	19.61385032
genus.Ruminiclostridium6.id.11356	rs2548459	19	T	C	0.055486587	0.012275456	6.40206E-06	13377		0.001525033	20.43152475
genus.Ruminiclostridium6.id.11356	rs73176030	7	C	T	0.058665044	0.013207973	7.28883E-06	13378		0.0014725	19.72815489
genus.Ruminiclostridium6.id.11356	rs35362464	4	A	C	0.072007723	0.016538673	8.99138E-06	13308		0.001422414	18.95644304
genus.Ruminiclostridium9.id.11357	rs57665991	3	G	C	-0.064150707	0.012333861	2.07274E-07	16725		0.001614867	27.05233558
genus.Ruminiclostridium9.id.11357	rs12040548	1	T	G	0.057042151	0.012235891	3.14872E-06	16721		0.001298058	21.7330443
genus.Ruminiclostridium9.id.11357	rs115044523	7	A	G	-0.098002558	0.020261641	2.37202E-06	16107		0.001450376	23.395137
genus.Ruminiclostridium9.id.11357	rs12419854	11	A	T	-0.072797471	0.015559424	3.17992E-06	16027		0.001363957	21.88998828
genus.Ruminiclostridium9.id.11357	rs6082461	20	C	A	0.058642458	0.013100394	4.86594E-06	16650		0.00120204	20.03805736
genus.Ruminiclostridium9.id.11357	rs113048721	9	G	C	0.060073481	0.013273731	4.10352E-06	16719		0.001223595	20.48234128
genus.Ruminiclostridium9.id.11357	rs918449	19	G	A	-0.095090817	0.019717682	2.55994E-06	16725		0.00138866	23.25762883
genus.Ruminiclostridium9.id.11357	rs73592673	6	T	A	-0.08162902	0.016951466	2.13971E-06	15493		0.001494478	23.18860689
genus.Ruminiclostridium9.id.11357	rs79082720	7	G	C	0.092857633	0.020487143	6.46935E-06	16094		0.001274836	20.54340381
genus.Ruminiclostridium9.id.11357	rs74303178	8	C	T	0.053274162	0.011927361	7.91566E-06	16706		0.001192762	19.95007457
genus.Ruminiclostridium9.id.11357	rs13033315	2	A	T	0.051119464	0.011141773	5.68052E-06	16724		0.001257122	21.05057451
genus.Ruminiclostridium9.id.11357	rs9809789	3	T	C	-0.071745357	0.015967315	8.71968E-06	16025		0.001258285	20.18941936
genus.Ruminiclostridium9.id.11357	rs9522712	13	C	T	0.069972768	0.015493905	4.65962E-06	16725		0.001217982	20.3955927
genus.Ruminiclostridium9.id.11357	rs78191726	6	C	T	0.094499761	0.02102058	7.58287E-06	16039		0.001258484	20.21026543
genus.Ruminiclostridium9.id.11357	rs7137760	12	T	C	0.050797017	0.011218236	7.06911E-06	16722		0.001224636	20.50346421
genus.RuminococcaceaeNK4A214g	rs5994253	22	G	A	-0.081130217	0.015762089	2.35183E-07	15288		0.001729956	26.49340197
genus.RuminococcaceaeNK4A214g	rs136761	22	A	G	-0.058752097	0.01191547	8.15195E-07	14822		0.001637592	24.31220794
genus.RuminococcaceaeNK4A214g	rs11586410	1	A	G	-0.086342178	0.016993683	3.66276E-07	14822		0.001738635	25.81493156
genus.RuminococcaceaeNK4A214g	rs34576931	12	C	G	-0.087331196	0.019488596	4.71641E-06	14678		0.001366209	20.08064662
genus.RuminococcaceaeNK4A214g	rs114244418	2	G	C	-0.17526622	0.037277927	3.59151E-06	7123		0.003093744	22.10512316
genus.RuminococcaceaeNK4A214g	rs4814689	20	T	C	-0.108300291	0.023070832	4.5519E-06	14009		0.001570518	22.03599783
genus.RuminococcaceaeNK4A214g	rs73158814	7	G	C	-0.10925732	0.022745845	2.19798E-06	13077		0.001761259	23.07262225
genus.RuminococcaceaeNK4A214g	rs12731	2	G	A	-0.052768427	0.01150534	4.87237E-06	15288		0.001374047	21.03533129
genus.RuminococcaceaeNK4A214g	rs7573569	2	C	T	0.107738536	0.023363816	3.23139E-06	14822		0.001432601	21.26447021
genus.RuminococcaceaeNK4A214g	rs147475196	3	G	A	-0.133768553	0.029519585	4.72496E-06	9917		0.002066374	20.53466212
genus.RuminococcaceaeNK4A214g	rs62027366	16	C	T	0.061538935	0.013761184	6.58456E-06	14822		0.001347398	19.99808094
genus.RuminococcaceaeNK4A214g	rs12642039	4	C	T	-0.055300297	0.011939746	3.43005E-06	15276		0.001402315	21.4518515
genus.RuminococcaceaeNK4A214g	rs35559912	5	C	T	-0.092507085	0.020367383	4.89023E-06	13976		0.00147386	20.62906502
genus.RuminococcaceaeNK4A214g	rs13087692	3	G	T	0.057462316	0.012593957	8.69224E-06	15288		0.001359879	20.81813567
genus.RuminococcaceaeNK4A214g	rs6681678	1	T	C	-0.100174165	0.024000023	9.04931E-06	13031		0.00133515	17.42160394
genus.RuminococcaceaeNK4A214g	rs11241747	5	T	C	0.053385843	0.012003462	6.58971E-06	14822		0.001332764	19.78058553
genus.RuminococcaceaeUCG002.ic	rs77564310	15	C	A	-0.071309265	0.014085525	3.28614E-07	17000		0.001505368	25.62983629
genus.RuminococcaceaeUCG002.ic	rs55793120	12	C	T	0.137396059	0.027413962	4.80674E-07	11669		0.002148015	25.11914909
genus.RuminococcaceaeUCG002.ic	rs10927423	1	A	C	-0.071361729	0.014771224	8.49518E-07	17096		0.001363361	23.33983368
genus.RuminococcaceaeUCG002.ic	rs67746927	4	G	C	-0.054237005	0.011035718	9.17088E-07	16651		0.001448506	24.1540647
genus.RuminococcaceaeUCG002.ic	rs116974815	11	A	C	-0.189730541	0.039656625	2.02518E-06	5819		0.003918227	22.88985227
genus.RuminococcaceaeUCG002.ic	rs7155595	14	A	C	0.056992918	0.011698636	1.14931E-06	17084		0.001387327	23.73402182
genus.RuminococcaceaeUCG002.ic	rs7120052	11	C	A	0.062479604	0.013552314	1.96895E-06	17097		0.001241624	21.254438
genus.RuminococcaceaeUCG002.ic	rs10916131	1	T	C	-0.069333027	0.014675325	2.87497E-06	17097		0.001303822	22.32054772
genus.RuminococcaceaeUCG002.ic	rs79016051	1	T	C	-0.088774672	0.018942419	2.33742E-06	16937		0.001295114	21.96379087
genus.RuminococcaceaeUCG002.ic	rs12463378	19	G	A	-0.052206066	0.011208649	2.95523E-06	16994		0.001274928	21.69378804
genus.RuminococcaceaeUCG002.ic	rs11750293	5	T	G	-0.057830385	0.012051229	1.76136E-06	17094		0.001345306	23.02764243
genus.RuminococcaceaeUCG002.ic	rs2265670	1	G	C	-0.051240397	0.010928696	2.99166E-06	17096		0.001284209	21.98306512
genus.RuminococcaceaeUCG002.ic	rs15256	10	T	C	0.073237587	0.016833997	9.45659E-06	17095		0.001105971	18.92751439
genus.RuminococcaceaeUCG002.ic	rs882348	4	G	A	-0.079994585	0.017861803	5.44852E-06	17097		0.001171768	20.0572143
genus.RuminococcaceaeUCG002.ic	rs72874194	2	C	G	-0.077028853	0.016724954	3.46664E-06	16651		0.001272283	21.21177501
genus.RuminococcaceaeUCG002.ic	rs6793778	3	T	C	-0.055870572	0.012525794	9.81289E-06	17094		0.001162537	19.89553965
genus.RuminococcaceaeUCG002.ic	rs7342369	12	A	C	-0.052780292	0.011602332	5.66121E-06	16651		0.00124129	20.69440923
genus.RuminococcaceaeUCG002.ic	rs10964441	9	A	G	-0.149060086	0.034485997	7.45116E-06	5346		0.003482516	18.68259344
genus.RuminococcaceaeUCG002.ic	rs11607472	11	G	A	-0.0780229	0.017632484	7.18671E-06	16282		0.001201123	19.58020322
genus.RuminococcaceaeUCG002.ic	rs7249614	19	G	A	-0.049277826	0.011080679	9.07376E-06	16651		0.001186355	19.77745338
genus.RuminococcaceaeUCG002.ic	rs76847269	5	G	A	0.163508017	0.035615077	5.1719E-06	7654		0.002746171	21.07707389
genus.RuminococcaceaeUCG002.ic	rs6542556	2	G	A	0.050974014	0.011406021	7.86123E-06	17094		0.001167021	19.97235666
genus.RuminococcaceaeUCG002.ic	rs57079348	13	G	T	-0.07657295	0.01728195	7.21822E-06	16372		0.001197686	19.63203353
genus.RuminococcaceaeUCG002.ic	rs56030423	15	A	G	-0.098287388	0.021634426	6.30385E-06	14937		0.001379881	20.6397692
genus.RuminococcaceaeUCG002.ic	rs362417	14	C	G	-0.054862993	0.01209839	7.80005E-06	17097		0.001201329	20.56382052
genus.RuminococcaceaeUCG002.ic	rs113147300	9	G	A	-0.075841986	0.016456212	7.6914E-06	16370		0.001295829	21.24025155
genus.RuminococcaceaeUCG003.ic	rs73341549	7	C	T	-0.169857156	0.031876785	1.50953E-07	6744		0.004192533	28.39348247
genus.RuminococcaceaeUCG003.ic	rs6759615	2	G	A	0.10252284	0.020020394	7.86224E-07	14410		0.001816529	26.22382381
genus.RuminococcaceaeUCG003.ic	rs646327	19	A	G	0.058669624	0.01183695	7.8318E-07	14406		0.001702408	24.56671098
genus.RuminococcaceaeUCG003.ic	rs11613919	12	T	G	0.072760434	0.015571648	1.62509E-06	14309		0.001523526	21.83340087
genus.RuminococcaceaeUCG003.ic	rs11243416	9	C	T	-0.092565265	0.019120311	1.66882E-06	14004		0.001670813	23.43722596
genus.RuminococcaceaeUCG003.ic	rs16959793	15	C	A	-0.062527033	0.013125865	2.21709E-06	14412		0.001572071	22.69236608
genus.RuminococcaceaeUCG003.ic	rs4452755	8	C	A	-0.06344632	0.01347408	3.29335E-06	14406		0.00153675	22.17249879
genus.RuminococcaceaeUCG003.ic	rs2548459	19	T	C	0.055446102	0.011842425	3.17535E-06	14409		0.001519031	21.92101815
genus.RuminococcaceaeUCG003.ic	rs10490280	2	T	C	-0.067209365	0.014330937	4.15551E-06	14004		0.001568111	21.99431183
genus.RuminococcaceaeUCG003.ic	rs4532474	6	A	G	0.076923106	0.017044961	4.82127E-06	14412		0.001411185	20.36674293
genus.RuminococcaceaeUCG003.ic	rs35866622	19	C	T	0.05664758	0.012186173	4.02264E-06	13915		0.001550497	21.60866881
genus.RuminococcaceaeUCG003.ic	rs4629039	18	A	T	0.055374213	0.012265944	6.54355E-06	14404		0.001412915	20.38042142
genus.RuminococcaceaeUCG003.ic	rs2523124	7	C	T	-0.054662483	0.012082341	5.7837E-06	14409		0.00141849	20.46805382
genus.RuminococcaceaeUCG003.ic	rs3013089	1	A	G	-0.05514734	0.012034599	4.38133E-06	14408		0.001455291	20.99838563
genus.RuminococcaceaeUCG003.ic	rs78720113	3	G	A	-0.115348392	0.02496006	7.59261E-06	13078		0.001630354	21.35658614
genus.RuminococcaceaeUCG003.ic	rs281379	19	G	A	0.05470387	0.012081047	7.01648E-06	13915		0.00147131	20.50345109
genus.RuminococcaceaeUCG003.ic	rs139730	22	C	G	-0.057765528	0.013064482	9.72112E-06	14004		0.001394104	19.55028463
genus.RuminococcaceaeUCG004.ic	rs6769553	3	G	A	0.084974255	0.015745058	7.91068E-08	9501		0.003056241	29.12635955
genus.RuminococcaceaeUCG004.ic	rs12125734	1	T	G	0.133971622	0.02574165	2.09428E-07	8938		0.003021331	27.08649808
genus.RuminococcaceaeUCG004.ic	rs511258	3	A	G	-0.075747368	0.016247466	4.52052E-06	9500		0.002282694	21.73520864
genus.RuminococcaceaeUCG004.ic	rs2248146	7	C	T	0.068945165	0.015364919	8.20001E-06	9502		0.002114525	20.13478856
genus.RuminococcaceaeUCG004.ic	rs10976229	9	G	T	0.095975994	0.021455386	7.04477E-06	9116		0.002190262	20.01025264
genus.RuminococcaceaeUCG004.ic	rs7569771	2	G	A	-0.075914029	0.01703081	8.11964E-06	9502		0.002086658	19.86888331
genus.RuminococcaceaeUCG004.ic	rs872501	8	A	G	0.116124682	0.025946001	5.80805E-06	8473		0.002358552	20.03125822
genus.RuminococcaceaeUCG004.ic	rs550351	1	C	A	0.078587023	0.018003041	9.43128E-06	9498		0.0020022	19.0550439
genus.RuminococcaceaeUCG004.ic	rs3800154	6	C	A	-0.079795901	0.017763932	6.11581E-06	9406		0.002140658	20.17822457
genus.RuminococcaceaeUCG004.ic	rs11961899	6	A	G	-0.070782539	0.01613814	9.1802E-06	9501		0.00202068	19.23735346
genus.RuminococcaceaeUCG004.ic	rs9818949	3	T	G	0.085992665	0.018867146	5.38832E-06	9116		0.002273618	20.77353472
genus.RuminococcaceaeUCG004.ic	rs7123615	11	G	C	-0.07861572	0.017959154	7.09181E-06	9502		0.002012598	19.16227346

genus.RuminococcaceaeUCG005.ic	rs10950694	7	C	T	0.057762846	0.011412014	4.298E-07	15821	0.001616724	25.61961263
genus.RuminococcaceaeUCG005.ic	rs34781347	20	A	G	0.188684782	0.03864836	6.04728E-07	6061	0.003917083	23.8348051
genus.RuminococcaceaeUCG005.ic	rs12288512	11	G	A	0.066635035	0.014435565	3.10278E-06	15473	0.001375199	21.30775583
genus.RuminococcaceaeUCG005.ic	rs60081663	5	G	C	0.158081538	0.031958601	9.28113E-07	9342	0.002612227	24.46734129
genus.RuminococcaceaeUCG005.ic	rs114279581	2	G	A	-0.146604207	0.031603425	3.21853E-06	8465	0.002535683	21.51912296
genus.RuminococcaceaeUCG005.ic	rs12458218	18	C	T	0.067732182	0.014452491	2.41027E-06	16099	0.001362429	21.96366152
genus.RuminococcaceaeUCG005.ic	rs35166120	8	G	C	-0.068615325	0.014625203	3.75403E-06	16092	0.00136595	22.01093228
genus.RuminococcaceaeUCG005.ic	rs7555878	1	G	A	0.058667737	0.012527736	2.81022E-06	16100	0.001360305	21.93074876
genus.RuminococcaceaeUCG005.ic	rs394449	17	T	A	0.069290871	0.014871206	2.59897E-06	15728	0.001378438	21.70999309
genus.RuminococcaceaeUCG005.ic	rs10873449	14	C	T	0.06548432	0.01439735	4.11169E-06	16098	0.001283453	20.68757306
genus.RuminococcaceaeUCG005.ic	rs2893871	10	A	G	-0.073643982	0.015547986	3.53845E-06	16103	0.001391281	22.43500952
genus.RuminococcaceaeUCG005.ic	rs72776570	10	A	C	0.087067007	0.019717899	5.35716E-06	15376	0.001266462	19.49781512
genus.RuminococcaceaeUCG005.ic	rs898577	15	C	T	-0.12301218	0.028667054	7.46313E-06	9991	0.001839592	18.41323522
genus.RuminococcaceaeUCG005.ic	rs7449320	5	A	C	0.059916334	0.013083635	4.80776E-06	16095	0.001301299	20.97169878
genus.RuminococcaceaeUCG005.ic	rs10937802	4	A	G	0.075581108	0.016822622	8.17353E-06	15821	0.001274241	20.18548283
genus.RuminococcaceaeUCG005.ic	rs7586445	2	A	G	0.07823492	0.017645763	8.8145E-06	15099	0.001300191	19.65714262
genus.RuminococcaceaeUCG005.ic	rs55793120	12	C	T	0.121547709	0.0279546	7.37046E-06	11224	0.001681545	18.90544846
genus.RuminococcaceaeUCG009.ic	rs8009993	14	C	G	-0.135936179	0.024485659	4.42176E-08	7389	0.004153872	30.8209848
genus.RuminococcaceaeUCG009.ic	rs1550196	17	A	G	0.130841508	0.026247787	1.13288E-06	7389	0.003351676	24.84881625
genus.RuminococcaceaeUCG009.ic	rs4708333	6	G	T	-0.084032963	0.017475702	1.56246E-06	7511	0.003069004	23.12225079
genus.RuminococcaceaeUCG009.ic	rs61779334	1	C	G	-0.138137216	0.029207242	1.94241E-06	6865	0.003247783	22.36867696
genus.RuminococcaceaeUCG009.ic	rs4079028	1	T	C	0.091562303	0.019936034	3.28244E-06	7480	0.002812103	21.09385215
genus.RuminococcaceaeUCG009.ic	rs2058609	12	G	A	0.081646419	0.017471971	3.12485E-06	7510	0.002899276	21.83687355
genus.RuminococcaceaeUCG009.ic	rs12508214	4	T	C	-0.077464871	0.016893699	4.75487E-06	7511	0.002791569	21.02616718
genus.RuminococcaceaeUCG009.ic	rs9558661	13	C	T	-0.089782261	0.020073862	7.00847E-06	7480	0.002667213	20.0041094
genus.RuminococcaceaeUCG009.ic	rs758191	16	G	T	0.177019514	0.037511304	9.00992E-06	5829	0.003805992	22.26988443
genus.RuminococcaceaeUCG009.ic	rs2192926	2	G	A	-0.089048031	0.019298901	4.87746E-06	7511	0.002826548	21.29038315
genus.RuminococcaceaeUCG009.ic	rs113006825	6	C	T	-0.092889294	0.020803474	7.97701E-06	7165	0.002774832	19.93699044
genus.RuminococcaceaeUCG009.ic	rs138460696	2	G	A	0.13927429	0.031579435	9.80841E-06	6865	0.002825296	19.45060884
genus.RuminococcaceaeUCG009.ic	rs6952765	7	A	G	0.073209862	0.016677682	8.13062E-06	7511	0.002558923	19.26938301
genus.RuminococcaceaeUCG009.ic	rs78410648	19	G	A	0.120988897	0.027731677	9.67203E-06	7510	0.00252813	19.034381
genus.RuminococcaceaeUCG010.ic	rs35506912	15	C	G	-0.069374287	0.014792554	3.20918E-06	12793	0.001716297	21.99433092
genus.RuminococcaceaeUCG010.ic	rs682403	9	G	A	-0.058816059	0.01246713	2.36537E-06	12864	0.001727158	22.25660155
genus.RuminococcaceaeUCG010.ic	rs6958419	7	T	C	-0.058571831	0.012499376	2.84162E-06	12870	0.001703264	21.95841277
genus.RuminococcaceaeUCG010.ic	rs2820282	6	C	A	-0.059230421	0.012591704	2.85385E-06	12865	0.001716977	22.12690261
genus.RuminococcaceaeUCG010.ic	rs12597105	16	A	G	0.067085456	0.014441482	4.873E-06	12415	0.001735131	21.57909352
genus.RuminococcaceaeUCG010.ic	rs7935775	11	T	A	-0.063135075	0.013795347	4.99423E-06	12864	0.001625525	20.94479738
genus.RuminococcaceaeUCG010.ic	rs73218807	4	A	G	-0.166210686	0.036793834	6.43033E-06	6714	0.00303018	20.40646564
genus.RuminococcaceaeUCG010.ic	rs7441445	4	T	C	-0.056949974	0.012650232	6.80187E-06	12852	0.001574472	20.26702688
genus.RuminococcaceaeUCG011.ic	rs1416041	6	C	A	-0.182339224	0.033989816	7.04419E-08	3632	0.007861206	28.77812974
genus.RuminococcaceaeUCG011.ic	rs12724320	1	T	C	-0.120880657	0.024922917	1.51986E-06	3632	0.006435262	23.5242555
genus.RuminococcaceaeUCG011.ic	rs79113084	12	T	C	-0.152165548	0.031751932	2.06123E-06	3610	0.006321658	22.96637026
genus.RuminococcaceaeUCG011.ic	rs9729514	1	G	A	0.18493353	0.039461636	2.36769E-06	3632	0.00601059	21.96246879
genus.RuminococcaceaeUCG011.ic	rs2729556	7	T	C	-0.109096996	0.023369889	3.18679E-06	3632	0.005964422	21.79276229
genus.RuminococcaceaeUCG011.ic	rs10274562	7	T	C	0.11091724	0.024455551	6.49607E-06	3632	0.005631766	20.57042201
genus.RuminococcaceaeUCG011.ic	rs4490371	3	C	T	-0.111816319	0.024896269	7.74624E-06	3610	0.005556671	20.17166945
genus.RuminococcaceaeUCG011.ic	rs12636310	3	A	G	0.132725381	0.028203654	2.81235E-06	3632	0.00606054	22.14609742
genus.RuminococcaceaeUCG013.ic	rs12781711	10	T	C	-0.065612917	0.011747657	2.55301E-08	16772	0.001856454	31.19436572
genus.RuminococcaceaeUCG013.ic	rs12189346	5	A	G	0.068496125	0.014558037	1.67955E-06	16470	0.001342299	22.13738744
genus.RuminococcaceaeUCG013.ic	rs75088940	12	C	T	-0.094296811	0.02007125	2.55399E-06	15638	0.001409456	22.07217685
genus.RuminococcaceaeUCG013.ic	rs16918863	10	C	A	0.111491182	0.024015687	4.15503E-06	12643	0.001701771	21.55216959
genus.RuminococcaceaeUCG013.ic	rs76973485	3	T	G	0.194975714	0.041821342	3.3498E-06	5299	0.004085013	21.73527336
genus.RuminococcaceaeUCG013.ic	rs12485353	3	A	G	-0.060790269	0.013084699	4.19379E-06	16771	0.001285355	21.5844349
genus.RuminococcaceaeUCG013.ic	rs9565219	13	A	T	-0.052459177	0.011770144	8.72524E-06	16772	0.001182988	19.86457875
genus.RuminococcaceaeUCG013.ic	rs1729063	16	C	G	-0.053341105	0.01207565	9.63581E-06	16470	0.001183301	19.51205337
genus.RuminococcaceaeUCG013.ic	rs7784330	7	A	G	-0.049832949	0.011206613	8.1626E-06	16770	0.001177713	19.77354025
genus.RuminococcaceaeUCG013.ic	rs11581881	1	T	C	0.066121072	0.014473649	4.72582E-06	15730	0.00132501	20.87006077
genus.RuminococcaceaeUCG013.ic	rs2428106	9	G	C	-0.0490863	0.010990264	8.37605E-06	16768	0.001188247	19.9482273
genus.RuminococcaceaeUCG013.ic	rs2730183	8	A	G	-0.048869155	0.010990742	8.43787E-06	16772	0.001177387	19.77040787
genus.RuminococcaceaeUCG013.ic	rs9313055	5	C	T	0.105087207	0.023446005	9.54713E-06	15764	0.001272748	20.08916971
genus.RuminococcaceaeUCG013.ic	rs12336782	9	C	T	-0.085604658	0.018931084	8.60305E-06	16772	0.001217671	20.44766954
genus.RuminococcaceaeUCG013.ic	rs4385846	10	T	G	0.05984045	0.013180685	6.46209E-06	16772	0.001227426	20.61169623
genus.RuminococcaceaeUCG014.ic	rs115777838	5	C	T	-0.188349525	0.038664295	4.62331E-07	5674	0.004164923	23.73060874
genus.RuminococcaceaeUCG014.ic	rs72809222	2	C	T	0.067177506	0.013983796	2.41191E-06	13922	0.001654919	23.07796991
genus.RuminococcaceaeUCG014.ic	rs12638134	3	G	T	0.058254795	0.011965732	1.21144E-06	14403	0.001642925	23.70199149
genus.RuminococcaceaeUCG014.ic	rs56105232	9	A	G	0.139275689	0.029913177	2.9128E-06	9190	0.002353351	21.6783167
genus.RuminococcaceaeUCG014.ic	rs995642	2	T	C	0.060047967	0.012641687	1.90222E-06	14402	0.00156417	22.56246397
genus.RuminococcaceaeUCG014.ic	rs10941294	5	T	C	-0.122057405	0.026001669	2.397E-06	12615	0.001743735	22.03564671
genus.RuminococcaceaeUCG014.ic	rs79640386	18	A	T	-0.110863476	0.024809787	8.73755E-06	13692	0.001456234	19.96783221
genus.RuminococcaceaeUCG014.ic	rs439810	2	C	G	-0.057712693	0.012667538	7.04188E-06	14403	0.001439063	20.75669563
genus.RuminococcaceaeUCG014.ic	rs10495392	1	T	C	-0.082485954	0.018719441	9.96329E-06	13902	0.001394732	19.41664827
genus.RuminococcaceaeUCG014.ic	rs62478832	7	A	T	-0.058128986	0.012903031	6.04427E-06	14331	0.0014142	20.29560629
genus.RuminococcaceaeUCG014.ic	rs61898819	11	T	A	0.060786464	0.01387607	9.91789E-06	14325	0.001337842	19.19025793
genus.RuminococcaceaeUCG014.ic	rs853612	10	G	A	-0.05282572	0.011936668	9.75451E-06	14403	0.001357943	19.58504689
genus.RuminococcaceaeUCG014.ic	rs73186226	3	A	G	-0.099385194	0.02168186	6.71749E-06	13760	0.001524647	21.01117318
genus.RuminococcaceaeUCG014.ic	rs34402072	8	T	C	-0.068802318	0.015608285	9.80426E-06	13863	0.001399685	19.43102798
genus.RuminococcaceaeUCG014.ic	rs74060145	1	G	C	-0.115766761	0.025419855	8.7051E-06	13011	0.001591546	20.74061509
genus.RuminococcaceaeUCG014.ic	rs77627087	16	G	C	0.067561994	0.015010339	7.42665E-06	14390	0.001405892	20.25927505
genus.RuminococcaceaeUCG014.ic	rs17296933	19	G	C	-0.08295439	0.018505763	7.33724E-06	13177	0.001522602	20.09392453
genus.RuminococcaceaeUCG014.ic	rs10791168	11	G	A	-0.066462357	0.015006876	9.76182E-06	14403	0.001359962	19.61421434
genus.Ruminococcus1.id.11373	rs10769159	11	C	G	-0.064027674	0.011005922	5.29128E-09	16603	0.002034284	33.84406814
genus.Ruminococcus1.id.11373	rs7117576	11	G	A	0.082950453	0.017089133	6.47979E-07	16606	0.001416826	23.56120209
genus.Ruminococcus1.id.11373	rs17781867	17	T	C	0.099927121	0.021172816	1.96335E-06	14349	0.001549938	22.27457963
genus.Ruminococcus1.id.11373	rs7583465	2	T	C	0.052765423	0.011261892	2.55613E-06	16588	0.001321624	21.95210355
genus.Ruminococcus1.id.11373	rs78613526	2	A	G	0.167496641	0.036763917	5.11425E-06	7266	0.002848619	20.75719381
genus.Ruminococcus1.id.11373	rs78572139	5	A	G	0.1250377	0.027941129	5.23105E-06	11667	0.001713523	20.02599314
genus.Ruminococcus1.id.11373	rs3000856	1	A	T	-0.070999702	0.016276599	9.27669E-06	16126	0.001178547	19.02767551
genus.Ruminococcus1.id.11373	rs6493760	15	T	C	0.053526195	0.011588457	3.38363E-06	16126	0.001321237	21.33445179
genus.Ruminococcus1.id.11373	rs11783695	8	T	G	-0.07341143	0.016139812	4.72679E-06	16606	0.001244299	20.68856866
genus.Ruminococcus1.id.11373	rs10995816	10	G	C	-0.07620021	0.017468886	8.38258E-06	16597	0.00114513	19.0275083
genus.Ruminococcus1.id.11373	rs10167839	2	G	A	0.051950897	0.011630648	8.09254E-06	16605	0.001200101	19.95162396
genus.Ruminococcus1.id.11373	rs3819978	1	T	C	-0.115039796	0.026013681	8.74133E-06	12769	0.001529224	19.55656595
genus.Ruminococcus1.id.11373	rs4849717	2	A	T	0.132763838	0.029981613	8.82616E-06	10484	0.001866857	19.60873696
genus.Ruminococcus1.id.11373	rs6105066	20	C	T	-0.060705188	0.01342499	5.05914E-06	16049	0.001272397	20.44672227
genus.Ruminococcus2.id.11374	rs78120384	3	G	A	-0.192815497	0.039204507	3.31185E-07	6082	0.003961336	24.18866404
genus.Ruminococcus2.id.11374	rs12986628	2	T	C	0.066593727	0.014016808	2.14202E-06	14714	0.001531694	22.57191492
genus.Ruminococcus2.id.11374	rs7635831	3	A	G	0.061834357	0.012903108	1.98054E-06	14924	0.001536448	22.965241
genus.Ruminococcus2.id.11374	rs2997412	13	G	A	-0.056843902	0.012232461	4.22116E-06	15336	0.001406102	21.59434681
genus.Ruminococcus2.id.11374	rs2368224	2	G	T	0.199647633	0.043845344	3.6293E-06	3026	0.006805293	20.73391776
genus.Ruminococcus2.id.11374	rs4400279	7	G	A	0.05461138	0.012007747	5.79929E-06	15333	0.001347196	20.68441475
genus.Ruminococcus2.id.11374	rs58681734	9	G	A	0.07238752	0.016078702	4.18175E-06	15338	0.001319724	20.26867818
genus.Ruminococcus2.id.11374	rs1819812	7	T	G	0.084235642	0.018489341	5.28435E-06	14868	0.001394089	20.75625026
genus.Ruminococcus2.id.11374	rs12406309	1	C	A	-0.063192979	0.014227548	9.78953E-06	15339	0.001284465	19.72774853
genus.Ruminococcus2.id.11374	rs2846589	18	T	G	0.052209321	0.011638161	7.58896E-06	14924	0.001346657	20.12460552
genus.Ruminococcus2.id.11374	rs55707116	9	A	C	0.08655215	0.01891409	8.00593E-06	15336	0.001363578	20.94039172
genus.Ruminococcus2.id.11374	rs75140805	11	G	T	0.083682495	0.017644509	3.95297E-06	14249	0.001576089	22.49314121
genus.Ruminococcus2.id.11374	rs4799823	18	T	C	0.083700126	0.018226258	5.40138E-06	14650	0.001437457	21.08905861
genus.Ruminococcus2.id.11374	rs61791565	4	C	T	-0.052350302	0.011711882	6.78933E-06	15319	0.001302532	19.97951793
genus.Ruminococcus2.id.11374	rs7693984	4	A	G	-0.102821803	0.023508663	9.42134E-06	14154	0.001349736	19.12998293
genus.Sellimonas.id.14369	rs13417181	2	C	T	0.166509314	0.033752138	7.61748E-07	3047	0.007924046	24.33741769
genus.Sellimonas.id.14369	rs2371572	2	C	A	0.127348939	0.025086238	4.46357E-07	3154	0.008104456	25.77030696
genus.Sellimonas.id.14369	rs113379006	3	C	T	-0.162758788	0.035702495	7.20777E-06	3154	0.006546028	20.7822117
genus.Sellimonas.id.14369	rs2187447	11	C	A	0.243457117	0.052769543	3.97806E-06	3028	0.006980399	21.2852266
genus.Sellimonas.id.14369	rs2016057	15	C	A	-0.125877526	0.02561532	1.02698E-06	3154	0.007598408	24.14887082
genus.Sellimonas.id.14369	rs72553859	1	C	G	-0.150458067	0.033262641	5.37687E-06	3154	0.006445366	20.46055882
genus.Sellimonas.id.14369	rs56203279	7	C	T	-0.124045002	0.026911497	3.71999E-06	3154	0.006691222	21.24627693
genus.Sellimonas.id.14369	rs553697	6	C	T	-0.153767275	0.033909892	6.12773E-06	3047	0.006703191	20.56245791
genus.Sellimonas.id.14369	rs7968030	12	T	A	-0.127110283	0.028061737	5.56417E-06	3095	0.006585701	20.51786999
genus.Sellimonas.id.14369	rs4600608	2	G	A	-0.137151754	0.03017017	4.94843E-06	3095	0.006632792	20.66556202
genus.Sellimonas.id.14369	rs41816	7	G	A	0.132201227	0.029108726	8.39372E-06	3154	0.006497286	20.62645478
genus.Senegalimassilia.id.11160	rs11787826	9	A	C	0.081327043	0.017115415	2.63466E-06	6923	0.003250773	22.57849919
genus.Senegalimassilia.id.11160	rs72887800	17	A	T	-0.082246156	0.017572066	2.41534E-06	6923	0.003154417	21.90713323
genus.Senegalimassilia.id.11160	rs7225245	17	A	G	0.079173009	0.017042101	4.18017E-06	6923	0.003107863	21.58281286
genus.Senegalimassilia.id.11160	rs57512504	13	A	T	0.081949442	0.017224097	2.03318E-06	6923	0.00325917	22.63701035
genus.Senegalimassilia.id.11160	rs10036909	5	T	C	0.185519381	0.040088092	8.05438E-06	4996	0.004268425	21.41646612
genus.Senegalimassilia.id.11160	rs13383270	2	C	G	0.077467918	0.017084039	6.03944E-06	6923	0.002961286	20.56187285
genus.Senegalimassilia.id.11160	rs1990708	2	C	A	-0.109619487	0.024778524	8.90578E-06	6923	0.00281906	19.57152473
genus.Senegalimassilia.id.11160	rs2017373	14	T	C	0.078225822	0.017684094	9.50288E-06	6923	0.002818477	19.56746806
genus.Slackia.id.825	rs8901	17	T	C	0.093459378	0.018681296	6.06998E-07	6063	0.004111072	25.02832326

genus.Slackia.id.825	rs4492265	7	G	A	-0.0905757	0.019165844	2.40925E-06	6063	0.003670144	22.33405036
genus.Slackia.id.825	rs16894137	8	T	C	-0.12279207	0.026304664	2.71183E-06	6063	0.003581207	21.79089771
genus.Slackia.id.825	rs112764253	7	A	T	0.194713531	0.041159784	3.39569E-06	5634	0.003956467	22.37927856
genus.Slackia.id.825	rs12440440	15	G	A	0.090193357	0.019058248	2.62872E-06	6063	0.003680394	22.39665556
genus.Slackia.id.825	rs35156985	7	C	T	-0.155708877	0.034808468	8.05947E-06	5965	0.003343428	20.01045372
genus.Slackia.id.825	rs10409783	19	G	A	0.095082845	0.021123946	7.69592E-06	5941	0.003398726	20.26069141
genus.Slackia.id.825	rs13339230	16	G	C	0.147054846	0.033063509	7.42025E-06	6063	0.00325206	19.78157234
genus.Slackia.id.825	rs58767323	4	C	G	-0.102833438	0.022707713	4.59992E-06	6063	0.003371071	20.50793491
genus.Streptococcus.id.1853	rs11110281	12	C	T	-0.137518884	0.022739814	2.58314E-09	15120	0.002412959	36.57218116
genus.Streptococcus.id.1853	rs11110282	12	G	A	-0.134637454	0.022297012	2.96907E-09	15229	0.002388518	36.46182923
genus.Streptococcus.id.1853	rs4968759	17	G	A	-0.051510944	0.011206836	3.78116E-06	16384	0.001287815	21.12676571
genus.Streptococcus.id.1853	rs11764382	7	G	A	-0.06953447	0.014367132	1.28631E-06	16387	0.001427382	23.42395136
genus.Streptococcus.id.1853	rs957755	7	G	T	-0.069737945	0.014372501	1.36669E-06	16387	0.001434665	23.54364043
genus.Streptococcus.id.1853	rs72739637	15	G	A	0.095994182	0.019321261	1.03307E-06	14920	0.001651704	24.68419074
genus.Streptococcus.id.1853	rs1918540	11	A	G	0.059639038	0.012814811	2.44067E-06	16373	0.001321096	21.65892341
genus.Streptococcus.id.1853	rs17708276	8	G	A	-0.079395519	0.017062753	3.04096E-06	16278	0.001328359	21.65178943
genus.Streptococcus.id.1853	rs77558518	5	G	A	-0.103999229	0.022971364	4.7086E-06	14949	0.00136924	20.49682875
genus.Streptococcus.id.1853	rs2952251	8	G	A	-0.057600624	0.012742871	5.88322E-06	16372	0.001246454	20.43242041
genus.Streptococcus.id.1853	rs11720390	3	A	G	0.107023904	0.022812145	3.59482E-06	15220	0.001444066	22.01047022
genus.Streptococcus.id.1853	rs395407	5	C	G	-0.079278061	0.01736973	4.36503E-06	15864	0.001311406	20.83147058
genus.Streptococcus.id.1853	rs10448310	9	G	A	-0.051793473	0.011132356	3.30706E-06	16384	0.001319419	21.64591326
genus.Streptococcus.id.1853	rs7916711	10	G	A	0.102890941	0.021736227	2.71702E-06	15294	0.001462949	22.40711509
genus.Streptococcus.id.1853	rs76717940	3	A	T	0.14952808	0.033686911	4.33947E-06	7607	0.002583368	19.70258129
genus.Streptococcus.id.1853	rs71481756	10	G	T	0.09310478	0.020794918	6.51476E-06	14526	0.001378112	20.04608293
genus.Streptococcus.id.1853	rs2370083	14	T	G	-0.081683584	0.018585095	9.75245E-06	16277	0.001185361	19.31701394
genus.Streptococcus.id.1853	rs10028567	4	T	C	-0.092116652	0.019188114	7.30343E-06	16311	0.00141097	23.04685614
genus.Streptococcus.id.1853	rs6806351	3	C	T	-0.063382863	0.013664741	4.93869E-06	15973	0.001345147	21.51497679
genus.Streptococcus.id.1853	rs57646748	4	A	G	-0.090769573	0.020034438	5.4754E-06	15939	0.001286193	20.527036
genus.Streptococcus.id.1853	rs9903102	17	A	C	-0.070948345	0.015527498	4.17998E-06	16384	0.001272649	20.87764903
genus.Streptococcus.id.1853	rs6563952	16	C	G	0.082734414	0.018003533	5.82134E-06	16387	0.001287058	21.11820158
genus.Subdoligranulum.id.2070	rs12638227	3	C	G	-0.055728761	0.010855202	2.47707E-07	17165	0.001533108	26.35621031
genus.Subdoligranulum.id.2070	rs10065321	5	C	T	-0.051282785	0.010810523	2.10155E-06	17589	0.001277772	22.5034861
genus.Subdoligranulum.id.2070	rs4347804	2	G	A	0.166063165	0.035748326	2.18405E-06	4312	0.004979531	21.5791935
genus.Subdoligranulum.id.2070	rs6555306	5	C	T	-0.074077668	0.015546287	2.80664E-06	17591	0.001289052	22.70498733
genus.Subdoligranulum.id.2070	rs3761728	4	G	T	-0.054345782	0.011886608	3.86747E-06	17570	0.001188304	20.90334643
genus.Subdoligranulum.id.2070	rs2114677	10	T	C	-0.104176347	0.023082878	2.71726E-06	15566	0.001306813	20.36846728
genus.Subdoligranulum.id.2070	rs75158211	19	C	T	-0.07234304	0.015933044	7.52162E-06	17165	0.001199585	20.61560203
genus.Subdoligranulum.id.2070	rs2171249	6	T	C	0.10674366	0.023321252	4.50556E-06	14679	0.001425163	20.94982841
genus.Subdoligranulum.id.2070	rs35940633	14	A	G	-0.051100832	0.011001029	4.21698E-06	17582	0.001225712	21.57691524
genus.Subdoligranulum.id.2070	rs76528319	5	T	G	-0.143278588	0.031066225	7.41394E-06	8468	0.00250562	21.27088744
genus.Subdoligranulum.id.2070	rs1667315	2	A	G	0.0485017	0.010745207	6.72143E-06	17586	0.001157216	20.37437639
genus.Subdoligranulum.id.2070	rs76664262	3	A	T	0.083407827	0.018535669	4.87344E-06	16712	0.00121016	20.2486964
genus.Subdoligranulum.id.2070	rs10497836	2	T	C	-0.052406498	0.011869733	8.37954E-06	17579	0.001107676	19.4934344
genus.Subdoligranulum.id.2070	rs16962433	16	T	A	0.085599862	0.018921315	7.64534E-06	15857	0.001289028	20.46649501
genus.Sutterella.id.2896	rs13173038	5	G	A	-0.071807564	0.015162698	2.72984E-06	13245	0.001690444	22.42783795
genus.Sutterella.id.2896	rs2321387	13	A	G	-0.059287622	0.012450922	1.86519E-06	13252	0.001708052	22.67383721
genus.Sutterella.id.2896	rs7499539	16	G	A	0.061747829	0.013099845	2.35769E-06	13254	0.001673542	22.21831185
genus.Sutterella.id.2896	rs62501473	7	A	G	0.069423259	0.014941649	5.51851E-06	12762	0.00168873	21.58802564
genus.Sutterella.id.2896	rs143438747	1	C	T	-0.145792693	0.030686706	3.28021E-06	9368	0.002403693	22.57204814
genus.Sutterella.id.2896	rs9350083	6	G	T	-0.059306886	0.013392944	8.22778E-06	13254	0.001477301	19.60912159
genus.Sutterella.id.2896	rs7638039	3	C	T	0.064562374	0.014388651	8.66397E-06	13236	0.001518805	20.13347943
genus.Sutterella.id.2896	rs11591622	10	G	T	-0.0688382	0.015137492	6.49857E-06	12604	0.001638064	20.68002929
genus.Sutterella.id.2896	rs2613606	7	T	C	-0.055679377	0.012411747	7.2023E-06	13249	0.001516633	20.12439844
genus.Sutterella.id.2896	rs1145877	6	G	A	-0.073548024	0.016241333	7.19949E-06	13251	0.001545178	20.50683729
genus.Sutterella.id.2896	rs607327	11	T	C	0.057833353	0.012905264	6.6306E-06	13251	0.001513271	20.08274112
genus.Sutterella.id.2896	rs2050185	6	A	G	0.057513842	0.012876636	7.96955E-06	13243	0.00150418	19.9498626
genus.Terrisporobacter.id.11348	rs1883097	11	T	C	0.226398815	0.045464179	4.15789E-07	4417	0.005582784	24.79759667
genus.Terrisporobacter.id.11348	rs2569953	3	C	A	-0.077559072	0.01746408	8.95009E-06	6637	0.002962875	19.72303584
genus.Terrisporobacter.id.11348	rs7184125	16	C	T	0.091256006	0.020549332	8.48103E-06	6232	0.003154481	19.72093499
genus.Terrisporobacter.id.11348	rs2872237	19	A	C	-0.08145049	0.017594312	3.97135E-06	6632	0.003221048	21.43102017
genus.Terrisporobacter.id.11348	rs58405430	11	T	G	0.134617158	0.030117736	7.9371E-06	6176	0.003224381	19.97819262
genus.Terrisporobacter.id.11348	rs7034891	9	C	G	-0.079920945	0.01737936	4.53776E-06	6629	0.003179966	21.14723924
genus.Turicibacter.id.2162	rs149744580	2	G	A	0.169882696	0.031547837	7.00965E-08	8459	0.003416283	28.99740396
genus.Turicibacter.id.2162	rs4869133	5	A	G	0.131186095	0.027196968	2.55369E-06	8256	0.002810236	23.26669101
genus.Turicibacter.id.2162	rs12603364	17	C	T	0.110860918	0.022559841	8.66598E-07	8923	0.002698984	24.14820594
genus.Turicibacter.id.2162	rs11054680	12	C	T	-0.104751216	0.02269975	2.30977E-06	8928	0.002379508	21.29491921
genus.Turicibacter.id.2162	rs55756211	7	C	T	-0.115115214	0.024070764	2.80533E-06	8941	0.002551468	22.87102802
genus.Turicibacter.id.2162	rs61265175	11	C	G	-0.085859068	0.018577761	4.13678E-06	8941	0.002383215	21.35923054
genus.Turicibacter.id.2162	rs7199484	16	A	G	-0.07314282	0.016017166	5.76664E-06	8948	0.002325065	20.85316774
genus.Turicibacter.id.2162	rs11649454	16	C	G	0.095089088	0.020343331	3.26627E-06	8943	0.002437105	21.84827875
genus.Turicibacter.id.2162	rs2834977	21	C	T	-0.095999549	0.020826107	3.95588E-06	8946	0.002369534	21.24820214
genus.Turicibacter.id.2162	rs2952020	8	A	G	-0.075901896	0.016576375	5.63308E-06	8949	0.002337412	20.96650957
genus.Turicibacter.id.2162	rs11666533	19	T	C	-0.111689111	0.024843618	7.37109E-06	8256	0.002442083	20.2111951
genus.Turicibacter.id.2162	rs4247078	15	G	C	0.071037672	0.015522123	5.46077E-06	8948	0.002335253	20.94475086
genus.Turicibacter.id.2162	rs2221441	5	C	G	0.071036413	0.015343033	3.45667E-06	8720	0.002452205	21.43579481
genus.Turicibacter.id.2162	rs3734633	6	A	G	-0.120956846	0.026830036	5.31914E-06	8626	0.002350644	20.32443027
genus.Tyzzerella3.id.11335	rs67476743	19	G	T	0.132163631	0.02220797	3.74049E-09	4948	0.007106881	35.41654677
genus.Tyzzerella3.id.11335	rs17706273	5	C	T	-0.140368365	0.027470793	5.87816E-07	5395	0.004816238	26.10935234
genus.Tyzzerella3.id.11335	rs7561370	2	C	T	0.131341092	0.028629245	1.51605E-06	5396	0.003885249	21.04657621
genus.Tyzzerella3.id.11335	rs55799124	17	G	A	-0.114350138	0.023860417	1.3355E-06	5362	0.004265149	22.96769041
genus.Tyzzerella3.id.11335	rs17809157	7	T	A	-0.163819473	0.033638641	1.54133E-06	4621	0.005106166	23.71669488
genus.Tyzzerella3.id.11335	rs7019909	9	C	T	0.144155503	0.030162645	1.7612E-06	5396	0.00421519	22.8414471
genus.Tyzzerella3.id.11335	rs75091807	13	T	G	-0.184966239	0.038302914	1.70674E-06	4621	0.005021104	23.31961062
genus.Tyzzerella3.id.11335	rs4904512	14	C	T	-0.117150935	0.025030561	3.09269E-06	5396	0.004043141	21.9053576
genus.Tyzzerella3.id.11335	rs7333521	13	C	T	-0.20719145	0.045312058	4.87602E-06	4335	0.004799957	20.90817274
genus.Tyzzerella3.id.11335	rs112102233	10	G	A	-0.216348445	0.04775752	6.17669E-06	3857	0.005292608	20.52220481
genus.Tyzzerella3.id.11335	rs1232220	6	T	G	-0.143869315	0.031828041	7.90792E-06	5000	0.004069822	20.43226632
genus.Tyzzerella3.id.11335	rs191093	12	A	G	0.15900811	0.035330566	6.76393E-06	4621	0.004364173	20.25523849
genus.Tyzzerella3.id.11335	rs10898797	11	T	C	0.122380253	0.027468252	8.85446E-06	5387	0.00367127	19.85000632
genus.Tyzzerella3.id.11335	rs6920448	6	T	C	-0.141080632	0.030544578	4.15376E-06	5010	0.004240172	21.33371787
genus.Veillonella.id.2198	rs2013594	11	C	T	-0.072069819	0.01551532	3.42129E-06	9283	0.002318936	21.5767176
genus.Veillonella.id.2198	rs12679709	8	G	C	-0.079349857	0.016481776	1.77579E-06	9290	0.002488779	23.1784443
genus.Veillonella.id.2198	rs742016	22	G	A	-0.068857084	0.01497691	4.65692E-06	9279	0.002272812	21.13746118
genus.Veillonella.id.2198	rs1882878	21	G	A	-0.07689658	0.016390829	2.98089E-06	9282	0.002365604	22.00960381
genus.Veillonella.id.2198	rs7645873	3	T	A	0.076138533	0.016422757	3.11802E-06	9286	0.00230932	21.4939828
genus.Veillonella.id.2198	rs55807413	11	G	A	0.107329355	0.023763343	5.51435E-06	8610	0.002363693	20.39961501
genus.Veillonella.id.2198	rs62376424	5	T	C	-0.076221631	0.016349923	3.65453E-06	8999	0.002409257	21.73326875
genus.Veillonella.id.2198	rs7359080	14	A	C	-0.135390734	0.030351973	7.39902E-06	6189	0.003204716	19.89775294
genus.Veillonella.id.2198	rs11141494	9	A	G	-0.078342138	0.017439254	9.74642E-06	9291	0.002167356	20.18064419
genus.Veillonella.id.2198	rs6656807	1	G	A	0.070306243	0.015395397	5.49694E-06	8999	0.002312099	20.85479953
genus.Veillonella.id.2198	rs11614532	12	C	G	0.074453782	0.016521422	7.12638E-06	8411	0.002408705	20.30853854
genus.Victivallis.id.2256	rs56349194	11	G	A	-0.158501176	0.031523075	6.25748E-07	3266	0.007681442	25.28179003
genus.Victivallis.id.2256	rs4396289	11	T	C	-0.158103202	0.031524149	6.54287E-07	3263	0.007649667	25.15327751
genus.Victivallis.id.2256	rs592514	9	A	T	-0.181448867	0.039167751	2.60375E-06	3241	0.006578183	21.46106435
genus.Victivallis.id.2256	rs911666	14	C	T	-0.118575703	0.026314576	7.65252E-06	3267	0.006176731	20.30479719
genus.Victivallis.id.2256	rs4764863	12	A	G	0.121560633	0.024605824	8.2222E-07	3266	0.00741755	24.4067574
genus.Victivallis.id.2256	rs7314815	12	A	G	0.120827207	0.024661642	8.53669E-07	3263	0.00730273	24.00410346
genus.Victivallis.id.2256	rs12512543	4	C	A	-0.1780361	0.037434441	2.5413E-06	3239	0.0069349	22.61900261
genus.Victivallis.id.2256	rs342302	7	G	A	-0.152754045	0.035157217	8.15946E-06	3265	0.005748697	18.87802046
genus.Victivallis.id.2256	rs4895919	6	C	T	-0.116917513	0.024751549	2.75199E-06	3254	0.006810343	22.31281445
genus.Victivallis.id.2256	rs11899949	2	A	G	0.130567821	0.027623889	2.7671E-06	3258	0.006810573	22.3410034
genus.Victivallis.id.2256	rs6445926	3	C	G	0.117030674	0.024976353	2.95824E-06	3267	0.006675493	21.95540036
genus.Victivallis.id.2256	rs173120	13	C	T	0.133809734	0.029012714	7.65253E-06	3263	0.006476788	21.2715291
genus.Victivallis.id.2256	rs2546432	5	C	T	-0.110803155	0.024965483	9.93269E-06	3266	0.005995103	19.6980982
genus.Victivallis.id.2256	rs1882775	21	G	A	-0.138270295	0.031272974	8.72833E-06	3258	0.005964448	19.54876878
order.Actinomycetales.id.420	rs35011108	6	G	A	0.24154595	0.050380314	1.87658E-06	3630	0.006292594	22.98676119
order.Actinomycetales.id.420	rs2889192	9	T	G	0.088405157	0.019499679	3.96723E-06	7711	0.00265848	20.55418548
order.Actinomycetales.id.420	rs34583783	6	T	G	0.123687922	0.026425408	5.53884E-06	7711	0.002833142	21.90842694
order.Actinomycetales.id.420	rs4073240	6	A	G	0.074972982	0.016467174	5.67895E-06	7711	0.002680988	20.72866842
order.Actinomycetales.id.420	rs58484246	15	C	T	0.075828017	0.016899684	6.67477E-06	7711	0.002604108	20.13270822
order.Bacillales.id.1674	rs74887130	21	G	C	-0.192165775	0.035881712	1.16118E-07	3218	0.008834176	28.68175798
order.Bacillales.id.1674	rs4617108	7	G	A	0.248860918	0.052592896	1.97506E-06	3018	0.007364273	22.39026468
order.Bacillales.id.1674	rs62640857	12	G	A	0.148237754	0.032645545	4.49259E-06	3235	0.00633339	20.61910536
order.Bacillales.id.1674	rs1287018	20	A	G	0.141125041	0.031930898	9.87091E-06	3207	0.006054101	19.53376226
order.Bacillales.id.1674	rs10410917	19	C	T	0.114710488	0.02500254	5.57348E-06	3239	0.006456748	21.04931677
order.Bacillales.id.1674	rs74420793	4	G	A	-0.164413018	0.035376325	3.07323E-06	3077	0.006970782	21.59966253
order.Bacillales.id.1674	rs11034576	11	G	A	0.205764503	0.045331088	8.85974E-06	3076	0.006653696	20.60386177
order.Bacillales.id.1674	rs10233278	7	C	T	-0.116318665	0.024852733	3.50803E-06	3239	0.006717572	21.90536609
order.Bacillales.id.1674	rs11207728	1	A	G	-0.144550179	0.031691157	5.73457E-06	3239	0.006382187	20.80468251

order.Bacillales.id.1674	rs11844714	14	G	A	-0.143245189	0.032027153	5.06212E-06	3239	0.006138164	20.00430284
order.Bacillales.id.1674	rs12522021	5	A	T	0.174338861	0.040051506	7.60217E-06	3235	0.005822911	18.94744685
order.Bacteroidales.id.913	rs55773148	13	A	G	-0.121513597	0.023675903	3.89585E-07	11349	0.002315646	26.34126632
order.Bacteroidales.id.913	rs7631304	3	A	G	-0.064577053	0.013295739	8.36781E-07	18334	0.001285038	23.59019404
order.Bacteroidales.id.913	rs73512608	13	A	G	-0.121371693	0.023675946	4.07905E-07	11349	0.002310245	26.27968294
order.Bacteroidales.id.913	rs73846128	3	G	A	-0.064852276	0.013349001	8.75939E-07	18334	0.001285692	23.60222274
order.Bacteroidales.id.913	rs73975615	17	A	G	-0.207017892	0.044263343	1.21616E-06	3350	0.006487181	21.87395677
order.Bacteroidales.id.913	rs111845179	14	C	T	0.102647804	0.021384281	9.23988E-07	16393	0.001403594	23.04146084
order.Bacteroidales.id.913	rs2032750	2	C	T	-0.050839276	0.010680731	1.91509E-06	17853	0.001267461	22.65670539
order.Bacteroidales.id.913	rs7546249	1	A	T	-0.056711256	0.01183859	1.54814E-06	18333	0.001250149	22.9476706
order.Bacteroidales.id.913	rs929878	16	T	C	-0.054853206	0.012152954	4.73377E-06	18334	0.001109943	20.37231184
order.Bacteroidales.id.913	rs13291169	9	G	C	0.068967579	0.014876501	3.74579E-06	17513	0.001225731	21.49257077
order.Bacteroidales.id.913	rs17343978	22	C	A	-0.055205684	0.012027767	8.35928E-06	18334	0.001147735	21.06675116
order.Bacteroidales.id.913	rs72706335	1	C	T	-0.222409754	0.049345816	7.65519E-06	3819	0.005291189	20.31453977
order.Bacteroidales.id.913	rs11146701	10	G	A	0.047359582	0.010540996	7.08228E-06	18220	0.001106683	20.18610194
order.Bacteroidales.id.913	rs62531359	8	G	T	0.065572807	0.014989058	9.08665E-06	18320	0.001043566	19.13810288
order.Bacteroidales.id.913	rs4146051	2	G	A	-0.107377003	0.024566124	8.76426E-06	11372	0.001677195	19.10509891
order.Bacteroidales.id.913	rs79585701	8	C	A	0.064682288	0.01496282	9.98589E-06	18334	0.001018226	18.68718223
order.Bacteroidales.id.913	rs4916508	3	A	G	-0.046672941	0.010531247	8.47116E-06	18324	0.001070743	19.64132232
order.Bacteroidales.id.913	rs62575403	9	T	C	0.140054866	0.031112824	7.06129E-06	9668	0.002091566	20.26364366
order.Bifidobacteriales.id.432	rs182549	2	T	C	0.117069879	0.01267034	5.942E-20	14915	0.005691304	85.37167272
order.Bifidobacteriales.id.432	rs7570971	2	C	A	0.112507848	0.012517823	2.5317E-19	14795	0.005430356	80.78078113
order.Bifidobacteriales.id.432	rs1530559	2	A	G	0.073829493	0.01197211	5.23222E-10	14795	0.002563826	38.02930229
order.Bifidobacteriales.id.432	rs7322849	13	C	T	0.110675689	0.020099551	1.74032E-08	14915	0.002028741	30.32017906
order.Bifidobacteriales.id.432	rs76671854	5	G	C	-0.089298109	0.018317684	1.03632E-06	13923	0.001704001	23.76530551
order.Bifidobacteriales.id.432	rs56108664	5	C	T	0.074567905	0.015721335	1.36916E-06	14041	0.001599675	22.49702148
order.Bifidobacteriales.id.432	rs4957061	5	C	T	0.056987252	0.011690731	1.1517E-06	14795	0.001603468	23.761404
order.Bifidobacteriales.id.432	rs10841473	12	C	G	-0.061303123	0.012884445	2.28067E-06	14914	0.001515587	22.63777448
order.Bifidobacteriales.id.432	rs4567981	2	A	T	0.057776313	0.011738841	8.88054E-07	14795	0.001634646	24.22418712
order.Bifidobacteriales.id.432	rs13020688	2	A	G	0.058404469	0.012208136	1.57375E-06	14795	0.001544569	22.887243
order.Bifidobacteriales.id.432	rs677208	18	C	G	0.072858928	0.016482072	6.0011E-06	14915	0.001308429	19.54078289
order.Bifidobacteriales.id.432	rs11745923	5	T	G	0.057975926	0.012102145	2.49154E-06	14914	0.001536416	22.94936694
order.Bifidobacteriales.id.432	rs12446429	16	C	T	0.081009916	0.019073029	8.52886E-06	14008	0.001286181	18.04002059
order.Bifidobacteriales.id.432	rs11655079	17	C	T	-0.058614646	0.012995163	5.92468E-06	14912	0.001362451	20.34458447
order.Bifidobacteriales.id.432	rs540489	17	G	T	-0.06325184	0.013816951	5.36811E-06	14688	0.001424754	20.95665056
order.Bifidobacteriales.id.432	rs857444	6	T	C	0.05539651	0.012066937	3.82492E-06	14914	0.001411118	21.0751537
order.Bifidobacteriales.id.432	rs10857328	4	A	T	0.056200033	0.012639517	7.24135E-06	14915	0.001323773	19.7702523
order.Bifidobacteriales.id.432	rs10831953	11	A	G	0.053753749	0.012374596	9.94742E-06	14915	0.001263523	18.86928686
order.Bifidobacteriales.id.432	rs7174549	15	T	C	0.055172631	0.01246547	6.8678E-06	14915	0.001311707	19.58980198
order.Bifidobacteriales.id.432	rs73797465	5	G	T	-0.094261173	0.020842122	4.8495E-06	14795	0.001380597	20.45416703
order.Bifidobacteriales.id.432	rs10496759	2	C	G	0.063713288	0.014430511	8.79796E-06	14914	0.001305375	19.49380532
order.Bifidobacteriales.id.432	rs55888705	4	G	A	0.053672952	0.012058615	8.66208E-06	14914	0.001326617	19.81144194
order.Bifidobacteriales.id.432	rs6899771	6	G	A	-0.09143118	0.020260353	7.27995E-06	14150	0.001437188	20.36547848
order.Bifidobacteriales.id.432	rs62181700	2	A	G	-0.060422854	0.013060631	4.12459E-06	14911	0.001433324	21.40297508
order.Burkholderiales.id.2874	rs62191117	2	G	A	0.067999488	0.013247059	2.79099E-07	16975	0.001549847	26.34949881
order.Burkholderiales.id.2874	rs6087811	20	G	T	-0.101586745	0.019881673	2.88374E-07	16135	0.001615463	26.1076774
order.Burkholderiales.id.2874	rs12467854	2	C	G	0.065933414	0.013196596	5.91277E-07	16984	0.001467606	24.96245613
order.Burkholderiales.id.2874	rs4033856	4	T	C	0.083344552	0.016748909	5.66823E-07	15897	0.001555214	24.76175256
order.Burkholderiales.id.2874	rs2367850	3	C	G	0.063330043	0.013059522	1.23614E-06	16664	0.001409202	23.51608533
order.Burkholderiales.id.2874	rs62395635	5	C	T	0.109911047	0.023637504	2.90228E-06	15062	0.001433421	21.62118349
order.Burkholderiales.id.2874	rs2321387	13	A	G	-0.050851372	0.010958763	3.25787E-06	17095	0.001257959	21.53189711
order.Burkholderiales.id.2874	rs7638039	3	C	T	0.058098748	0.012673046	4.83593E-06	17078	0.001229137	21.01704011
order.Burkholderiales.id.2874	rs2613606	7	T	C	-0.049956147	0.010926388	4.13357E-06	17092	0.001221521	20.90377042
order.Burkholderiales.id.2874	rs9537886	13	C	A	-0.049651209	0.010977691	5.83285E-06	17094	0.001195293	20.45679717
order.Burkholderiales.id.2874	rs11128180	3	G	A	0.058279373	0.012896608	5.53168E-06	17097	0.001193	20.42108085
order.Burkholderiales.id.2874	rs72747231	9	G	C	-0.133728739	0.029073834	3.76217E-06	11315	0.00186629	21.15655123
order.Burkholderiales.id.2874	rs1928341	1	G	A	0.050778221	0.011058697	4.51508E-06	17096	0.001231735	21.08371265
order.Burkholderiales.id.2874	rs9964679	18	G	A	0.052509048	0.011503961	6.05981E-06	17097	0.001217095	20.83403808
order.Burkholderiales.id.2874	rs1511453	4	G	A	0.09109992	0.01994793	8.00097E-06	17032	0.001223047	20.85644593
order.Burkholderiales.id.2874	rs75242906	15	T	C	-0.120974344	0.028127277	9.74519E-06	10766	0.001715265	18.49827278
order.Clostridiales.id.1863	rs6815608	4	C	T	0.104089026	0.021088307	3.71989E-07	16731	0.001454028	24.36275917
order.Clostridiales.id.1863	rs112334273	21	A	G	0.063938344	0.012740449	4.07257E-07	18217	0.001380627	25.18565581
order.Clostridiales.id.1863	rs6814436	4	C	T	0.074185762	0.015081384	9.06288E-07	18336	0.001317897	24.19684558
order.Clostridiales.id.1863	rs72915163	18	C	T	-0.058024623	0.012055772	1.39104E-06	18317	0.001263081	23.16512194
order.Clostridiales.id.1863	rs2273429	14	G	A	-0.072619093	0.015310519	4.16871E-06	18337	0.001225353	22.49685641
order.Clostridiales.id.1863	rs10774377	12	G	A	0.052300318	0.011385929	3.80899E-06	18330	0.001149766	21.09947891
order.Clostridiales.id.1863	rs13179700	5	C	T	0.051095292	0.010956887	3.52111E-06	18338	0.001184461	21.74640301
order.Clostridiales.id.1863	rs6934062	6	C	G	0.05223974	0.011611578	7.09483E-06	17859	0.001132063	20.24042046
order.Clostridiales.id.1863	rs10209007	2	C	G	0.079542419	0.018242704	9.59556E-06	17812	0.001066211	19.01162155
order.Clostridiales.id.1863	rs3792064	2	A	G	0.079670519	0.018252096	9.74925E-06	17812	0.001068545	19.05328319
order.Clostridiales.id.1863	rs1842454	5	A	G	-0.054367588	0.012734002	9.91744E-06	18340	0.000992932	18.2284739
order.Clostridiales.id.1863	rs76767978	3	A	G	0.069980605	0.015631841	8.17561E-06	18329	0.001092249	20.04172671
order.Clostridiales.id.1863	rs13105690	4	C	T	-0.052744064	0.011818018	9.36806E-06	18340	0.001084894	19.91855803
order.Clostridiales.id.1863	rs7269302	20	T	A	-0.096534637	0.02120063	8.84345E-06	17405	0.00118981	20.73331664
order.Clostridiales.id.1863	rs72738886	5	C	T	0.086533337	0.019039215	8.41577E-06	16934	0.001218372	20.65707513
order.Clostridiales.id.1863	rs76860606	6	G	C	0.094122019	0.022511723	8.58167E-06	17579	0.000993434	17.48094869
order.Clostridiales.id.1863	rs290772	2	A	G	0.084254009	0.01962973	9.99759E-06	17129	0.001074369	18.42266694
order.Clostridiales.id.1863	rs6442336	3	T	C	-0.054772905	0.012416948	9.62568E-06	18333	0.001060248	19.45816399
order.Coriobacteriales.id.810	rs719099	10	G	A	0.077840173	0.01558151	5.43317E-07	17687	0.001409035	24.95677559
order.Coriobacteriales.id.810	rs240104	1	C	T	-0.060342897	0.012684911	1.51603E-06	17577	0.001285801	22.62962954
order.Coriobacteriales.id.810	rs11250875	10	C	T	0.060749978	0.013093887	4.82844E-06	17690	0.001215344	21.5255891
order.Coriobacteriales.id.810	rs34739816	17	T	G	0.096501674	0.020766582	3.88358E-06	16593	0.00129972	21.59432574
order.Coriobacteriales.id.810	rs76779974	5	G	C	0.0774888	0.017187698	5.63091E-06	16419	0.001236398	20.32555641
order.Coriobacteriales.id.810	rs80046645	5	G	C	0.255839805	0.056199532	4.65689E-06	3204	0.006426556	20.72386883
order.Coriobacteriales.id.810	rs45480394	19	G	T	-0.050426168	0.011330684	9.71448E-06	17413	0.001136141	19.80613186
order.Coriobacteriales.id.810	rs1816223	12	G	A	-0.058626474	0.012900576	4.84114E-06	17688	0.001166229	20.65234419
order.Coriobacteriales.id.810	rs67561917	20	G	A	-0.071436723	0.015411343	5.39129E-06	17333	0.001238085	21.48632118
order.Coriobacteriales.id.810	rs3025411	9	G	A	0.092654285	0.020946781	8.26912E-06	15671	0.001246975	19.56574696
order.Coriobacteriales.id.810	rs11656361	17	C	A	0.077300587	0.01755297	8.02017E-06	16665	0.001162396	19.3938654
order.Coriobacteriales.id.810	rs1397793	5	A	G	-0.049885455	0.011244635	9.77381E-06	17527	0.001121662	19.68145117
order.Coriobacteriales.id.810	rs11073596	15	G	T	0.051021228	0.011433922	8.14449E-06	17527	0.001134777	19.91183886
order.Coriobacteriales.id.810	rs2442778	3	A	G	-0.116408329	0.025854513	9.02703E-06	13472	0.001502485	20.27194292
order.Coriobacteriales.id.810	rs7540303	1	T	C	0.048322746	0.010906179	9.62148E-06	17691	0.00110847	19.63170353
order.Coriobacteriales.id.810	rs62448869	7	A	T	-0.048605943	0.010898242	7.87276E-06	17527	0.001133615	19.89142221
order.Coriobacteriales.id.810	rs13307134	7	T	C	0.056568602	0.012626474	7.79931E-06	17527	0.001143884	20.0718175
order.Coriobacteriales.id.810	rs12974142	19	A	G	0.078979608	0.017720479	8.51232E-06	17413	0.001139489	19.86456328
order.Coriobacteriales.id.810	rs8010111	14	A	G	-0.10337688	0.022928631	6.89624E-06	15996	0.001269193	20.32780913
order.Desulfovibrionales.id.3156	rs6058181	20	T	C	0.083676458	0.016609784	2.5277E-07	15076	0.001680591	25.37923927
order.Desulfovibrionales.id.3156	rs4506934	17	T	C	-0.095338347	0.020136702	2.42998E-06	15037	0.001488505	22.41602284
order.Desulfovibrionales.id.3156	rs11599763	10	C	T	-0.055486167	0.011748632	2.60698E-06	15144	0.001470669	22.30462156
order.Desulfovibrionales.id.3156	rs2838334	21	A	G	0.056902402	0.01242233	4.16791E-06	15144	0.001383608	20.98239636
order.Desulfovibrionales.id.3156	rs9928243	16	A	C	-0.054471273	0.011781078	3.96636E-06	15143	0.001409744	21.37789577
order.Desulfovibrionales.id.3156	rs112381107	9	T	C	0.210090212	0.045697378	3.21653E-06	4455	0.004721998	21.13630503
order.Desulfovibrionales.id.3156	rs16851319	1	C	G	-0.072913728	0.015095443	2.70287E-06	15130	0.001539639	23.33065192
order.Desulfovibrionales.id.3156	rs17791387	9	G	A	-0.072750113	0.015436947	2.25294E-06	15047	0.001473853	22.20979281
order.Desulfovibrionales.id.3156	rs186073	3	C	T	0.052921896	0.011854172	8.74432E-06	14841	0.001341165	19.93096378
order.Desulfovibrionales.id.3156	rs2692012	1	G	A	0.112244115	0.025355338	2.27021E-06	13082	0.001495768	19.59694367
order.Desulfovibrionales.id.3156	rs62020470	15	G	A	-0.057363273	0.012946185	7.50981E-06	15144	0.001294735	19.63288979
order.Desulfovibrionales.id.3156	rs7164160	15	T	A	-0.057293808	0.01294613	7.76227E-06	15144	0.001291616	19.58553472
order.Desulfovibrionales.id.3156	rs3935584	2	T	C	-0.052446625	0.011574877	7.1974E-06	15142	0.001354037	20.53063142
order.Desulfovibrionales.id.3156	rs72647048	8	C	T	-0.077245856	0.017087645	9.00294E-06	15144	0.001347596	20.43553317
order.Desulfovibrionales.id.3156	rs55744759	8	G	A	-0.077394236	0.017087666	9.01945E-06	15144	0.001352768	20.51406492
order.Enterobacteriales.id.3468	rs78143293	18	G	A	-0.084852127	0.017041521	1.19564E-06	13572	0.001823359	24.79183876
order.Enterobacteriales.id.3468	rs111229068	11	T	A	0.110560883	0.024171888	3.65356E-06	12350	0.001691141	20.92097106
order.Enterobacteriales.id.3468	rs62210023	20	G	A	0.060675357	0.013012497	3.13163E-06	13515	0.001606162	21.74219515
order.Enterobacteriales.id.3468	rs504442	18	G	T	0.084159032	0.01894789	5.17092E-06	13578	0.00145082	19.7278506
order.Enterobacteriales.id.3468	rs80319214	2	G	C	0.099040406	0.021587746	6.95307E-06	12641	0.001662288	21.04796975
order.Enterobacteriales.id.3468	rs11026530	11	C	T	0.082240818	0.018637912	9.42684E-06	13485	0.001441792	19.47064398
order.Enterobacteriales.id.3468	rs2374342	2	A	C	0.058292653	0.01261946	4.51618E-06	13577	0.001569135	21.33762573
order.Enterobacteriales.id.3468	rs79757635	13	A	C	0.075859672	0.017128562	9.31857E-06	13419	0.001459572	19.6146289
order.Enterobacteriales.id.3468	rs35673018	16	A	G	0.08996415	0.020293422	7.63109E-06	13326	0.001472613	19.65297836
order.Enterobacteriales.id.3468	rs61973590	14	G	C	-0.060539207	0.013369618	8.5354E-06	13570	0.00150869	20.50385086
order.Enterobacteriales.id.3468	rs4792380	17	T	A	0.115597849	0.025768301	9.48974E-06	12763	0.001574312	20.12463118
order.Erysipelotrichales.id.2148	rs62504403	8	T	C	0.068115892	0.012788339	1.12265E-07	16981	0.00166794	28.37060806
order.Erysipelotrichales.id.2148	rs7234058	18	C	T	-0.094575892	0.019409083	9.12341E-07	16874	0.001405148	23.74383587
order.Erysipelotrichales.id.2148	rs17530232	13	G	A	0.103049982	0.022464754	2.79123E-06	16622	0.001264328	21.04226554
order.Erysipelotrichales.id.2148	rs2300774	3	A	G	0.052415201	0.010678379	8.95466E-07	18216	0.001320921	24.09371743
order.Erysipelotrichales.id.2148	rs35161940	17	C	T	-0.080604056	0.01676409	1.8451E-06	18213	0.001267714	23.11819028
order.Erysipelotrichales.id.2148	rs10781552	10	T	C	-0.055221236	0.011607425	2.33114E-06	17789	0.001270682	22.63291274

order.Erysipelotrichales.id.2148	rs8003149	14	T	C	0.053879756	0.01168879	4.08475E-06	18213	0.001165264	21.24770908
order.Erysipelotrichales.id.2148	rs4078432	14	T	C	-0.060892934	0.013376363	4.23104E-06	17789	0.001163591	20.72324212
order.Erysipelotrichales.id.2148	rs1074800	5	G	A	0.049243827	0.010887044	6.14583E-06	17789	0.00114877	20.45896385
order.Erysipelotrichales.id.2148	rs56970041	14	G	T	0.072414447	0.016447064	5.40344E-06	18217	0.001063004	19.38535258
order.Erysipelotrichales.id.2148	rs7826267	8	G	T	-0.083907147	0.019913089	9.28449E-06	17586	0.001008592	17.75499888
order.Erysipelotrichales.id.2148	rs1884466	1	T	C	-0.047536345	0.01069386	9.52701E-06	18205	0.001084228	19.75979992
order.Erysipelotrichales.id.2148	rs290833	1	G	T	-0.049737371	0.011137647	8.029E-06	17789	0.0011198	19.94246102
order.Gastranaerophilales.id.1591	rs9864379	3	C	T	-0.160521381	0.029275217	4.65873E-08	5768	0.005185402	30.0653001
order.Gastranaerophilales.id.1591	rs11150282	16	C	T	0.098234998	0.019712655	7.36053E-07	5864	0.004217091	24.83374669
order.Gastranaerophilales.id.1591	rs16851659	1	C	G	-0.089516816	0.01865552	1.27024E-06	5875	0.003903803	23.02472896
order.Gastranaerophilales.id.1591	rs4129395	9	A	G	0.090426365	0.01852532	1.21742E-06	5878	0.004037126	23.82641619
order.Gastranaerophilales.id.1591	rs1221147	9	A	T	0.126305018	0.027916132	4.8596E-06	5759	0.003541952	20.47060782
order.Gastranaerophilales.id.1591	rs79790072	15	C	T	0.226102517	0.048801659	3.5381E-06	4104	0.005203178	21.46553052
order.Gastranaerophilales.id.1591	rs73074665	7	T	A	0.164631221	0.035603665	3.6238E-06	5749	0.003705361	21.3813435
order.Gastranaerophilales.id.1591	rs113884518	9	C	T	-0.205909694	0.045537561	7.73987E-06	4434	0.004590081	20.44626796
order.Gastranaerophilales.id.1591	rs789069	18	C	A	-0.104129854	0.023445913	6.50003E-06	5870	0.003349047	19.72496377
order.Gastranaerophilales.id.1591	rs367480	11	A	G	-0.084164921	0.018594792	7.52418E-06	5878	0.003473273	20.48705839
order.Gastranaerophilales.id.1591	rs8028558	15	G	A	0.083459982	0.018850404	9.78236E-06	5875	0.003325529	19.60267022
order.Gastranaerophilales.id.1591	rs28678345	17	C	T	0.213082993	0.047137686	8.05953E-06	4315	0.004713336	20.43436078
order.Lactobacillales.id.1800	rs2952251	8	G	A	-0.06293169	0.012417747	3.36461E-07	17278	0.001484276	25.68343838
order.Lactobacillales.id.1800	rs17708276	8	G	A	-0.083581438	0.016647604	4.38008E-07	17184	0.001464722	25.20671154
order.Lactobacillales.id.1800	rs35344081	16	A	G	0.064244209	0.012735835	4.16066E-07	17292	0.001469364	25.44562685
order.Lactobacillales.id.1800	rs76717940	3	A	T	0.160942577	0.032699971	6.55312E-07	7976	0.003027924	24.22407296
order.Lactobacillales.id.1800	rs77558518	5	G	A	-0.106465937	0.022348355	1.67435E-06	15758	0.00143815	22.69500233
order.Lactobacillales.id.1800	rs11110282	12	G	A	-0.102389846	0.021784941	3.95665E-06	15956	0.001382535	22.09026931
order.Lactobacillales.id.1800	rs9581006	13	T	C	0.225829511	0.046922805	1.76596E-06	4224	0.005453741	23.16292791
order.Lactobacillales.id.1800	rs4028634	17	C	T	0.053291462	0.011009978	1.34806E-06	17295	0.001352801	23.42838434
order.Lactobacillales.id.1800	rs74352383	6	A	T	-0.103396459	0.021757973	1.88028E-06	16442	0.001371588	22.5826266
order.Lactobacillales.id.1800	rs78938557	7	C	T	0.105530209	0.023358105	2.31087E-06	15152	0.001345314	20.411663
order.Lactobacillales.id.1800	rs57872228	1	T	C	-0.068841481	0.014699394	2.58252E-06	17293	0.00126672	21.93317609
order.Lactobacillales.id.1800	rs1962325	3	G	C	0.054687802	0.01150694	1.96232E-06	17274	0.001305873	22.58713989
order.Lactobacillales.id.1800	rs11730038	4	A	G	-0.060538395	0.01290523	5.09746E-06	17295	0.001270742	22.00545221
order.Lactobacillales.id.1800	rs11110281	12	C	T	-0.101766774	0.022227995	7.02902E-06	15845	0.001321128	20.96097307
order.Lactobacillales.id.1800	rs2370083	14	T	G	-0.080539958	0.018099185	8.33495E-06	17183	0.00115108	19.80180409
order.Lactobacillales.id.1800	rs9345899	6	G	A	-0.082515531	0.017576055	3.78222E-06	16523	0.001332173	22.04086231
order.Lactobacillales.id.1800	rs12797734	11	C	T	0.057127947	0.012711299	7.76523E-06	16820	0.001199417	20.19841952
order.Lactobacillales.id.1800	rs34989881	19	G	A	0.113478844	0.024645168	4.09354E-06	12921	0.001638167	21.20148261
order.Lactobacillales.id.1800	rs11627423	14	A	C	0.049926314	0.010974412	5.08753E-06	17275	0.001196626	20.69648105
order.Lactobacillales.id.1800	rs11720390	3	A	G	0.100082923	0.02230291	9.73244E-06	15940	0.001261712	20.13709837
order.Lactobacillales.id.1800	rs111552159	4	G	C	0.128854393	0.028739711	8.30804E-06	11312	0.001773876	20.10174095
order.Lactobacillales.id.1800	rs1595463	2	A	C	0.047974934	0.010880146	7.44354E-06	17276	0.001124159	19.44282122
order.Lactobacillales.id.1800	rs74663707	3	T	C	0.09825937	0.022461255	8.39558E-06	15695	0.001217838	19.13726715
order.Methanobacteriales.id.120	rs10202904	2	G	T	-0.121753579	0.023535682	3.0143E-07	3695	0.007190538	26.76146558
order.Methanobacteriales.id.120	rs73457410	13	G	A	0.215339755	0.04366952	1.40855E-06	3498	0.006903404	24.3159698
order.Methanobacteriales.id.120	rs76029318	13	C	T	0.214769455	0.0446468	1.58427E-06	3497	0.006573617	23.14005323
order.Methanobacteriales.id.120	rs6776814	3	C	T	-0.199565931	0.04118249	1.63062E-06	3385	0.006889481	23.48267699
order.Methanobacteriales.id.120	rs894996	4	A	C	0.216999069	0.044907554	1.87557E-06	3582	0.006476339	23.34946566
order.Methanobacteriales.id.120	rs73031978	3	G	C	-0.203186321	0.042161972	1.90189E-06	3340	0.006905438	23.22453869
order.Methanobacteriales.id.120	rs75208022	12	T	C	-0.227244713	0.048763001	5.92103E-06	3203	0.006734651	21.71734467
order.Methanobacteriales.id.120	rs12825290	12	G	C	-0.216781075	0.049349641	6.07567E-06	3144	0.006100071	19.29633077
order.Methanobacteriales.id.120	rs56131665	5	A	G	0.178661188	0.039297982	6.17992E-06	3572	0.005753111	20.66902243
order.Methanobacteriales.id.120	rs11018665	11	T	A	0.111465287	0.025051554	6.51864E-06	3698	0.005325057	19.79748181
order.Methanobacteriales.id.120	rs4257531	3	A	G	0.164498365	0.03649593	7.44344E-06	3162	0.006383973	20.31581768
order.Methanobacteriales.id.120	rs6508769	19	C	T	0.15351913	0.034451866	8.22529E-06	3319	0.005947052	19.85635359
order.Methanobacteriales.id.120	rs73068003	7	T	G	-0.158140381	0.035180317	8.44583E-06	3692	0.005443196	20.20626583
order.Methanobacteriales.id.120	rs10424197	19	A	G	-0.111274175	0.024751678	9.27887E-06	3698	0.005435575	20.2106127
order.MollicutesRF9.id.11579	rs74603314	14	C	T	0.230767094	0.048970099	2.27641E-06	4033	0.005476118	22.20679063
order.MollicutesRF9.id.11579	rs76373661	3	A	G	0.090821805	0.020298596	5.15941E-06	10359	0.001928821	20.01927169
order.MollicutesRF9.id.11579	rs17235252	7	C	T	-0.12200211	0.02554629	2.15879E-06	9531	0.002387275	22.80756955
order.MollicutesRF9.id.11579	rs638542	1	A	G	-0.070591966	0.015704899	5.17285E-06	9970	0.002022395	20.2041345
order.MollicutesRF9.id.11579	rs62188991	2	C	G	-0.110864082	0.02413552	5.26703E-06	9531	0.002208867	21.09931802
order.MollicutesRF9.id.11579	rs949341	11	A	G	0.065660222	0.014721644	7.73282E-06	10355	0.001917381	19.89262279
order.MollicutesRF9.id.11579	rs13100746	3	T	C	0.063897883	0.014268538	7.28846E-06	10359	0.001932218	20.0545986
order.MollicutesRF9.id.11579	rs515984	10	C	T	-0.087545066	0.019068514	6.61366E-06	10359	0.002030622	21.07801201
order.MollicutesRF9.id.11579	rs7706512	5	A	G	0.065735114	0.013897929	2.27207E-06	10359	0.002154964	22.37147845
order.MollicutesRF9.id.11579	rs11779863	8	A	G	-0.077276806	0.017217056	6.68845E-06	10353	0.001942093	20.14561597
order.MollicutesRF9.id.11579	rs7801843	7	G	A	-0.086949468	0.01945094	9.46882E-06	10359	0.001925298	19.98263043
order.MollicutesRF9.id.11579	rs10071529	5	C	G	0.125104505	0.027867407	8.64406E-06	10147	0.001982227	20.15360715
order.MollicutesRF9.id.11579	rs739151	22	G	C	0.065265852	0.013977274	3.09287E-06	10337	0.002104832	21.80354443
order.MollicutesRF9.id.11579	rs12566890	1	G	T	-0.10313363	0.024177477	8.11201E-06	10339	0.001756857	18.19611471
order.MollicutesRF9.id.11579	rs3932485	3	T	C	0.062620895	0.014162324	9.92857E-06	10359	0.001883791	19.55102208
order.MollicutesRF9.id.11579	rs7853673	9	A	G	-0.062438944	0.013983251	6.7325E-06	10359	0.001921064	19.93860704
order.NB1n.id.3953	rs60583455	7	C	T	0.108933641	0.021176966	2.59578E-07	4773	0.005513205	26.46040897
order.NB1n.id.3953	rs2172426	8	T	C	-0.102119829	0.019894658	3.17425E-07	5099	0.005140719	26.3479734
order.NB1n.id.3953	rs11251024	10	A	G	0.104201088	0.020673705	6.62801E-07	5107	0.004949793	25.40433888
order.NB1n.id.3953	rs13219468	6	C	G	0.115343714	0.023695802	1.40637E-06	5104	0.004620861	23.69436346
order.NB1n.id.3953	rs267959	5	G	A	0.098861186	0.020967556	2.61993E-06	5107	0.004334148	22.23084666
order.NB1n.id.3953	rs11606187	11	G	A	-0.154539114	0.032628781	3.30586E-06	5102	0.004377532	22.43236871
order.NB1n.id.3953	rs7911787	10	T	G	-0.223070508	0.047023233	3.38824E-06	3879	0.005768027	22.50398062
order.NB1n.id.3953	rs72671304	14	C	T	0.172321792	0.037002808	3.79578E-06	4887	0.004418203	21.68757775
order.NB1n.id.3953	rs13385922	2	C	T	0.092949422	0.020134823	3.96829E-06	5106	0.004156312	21.31070158
order.NB1n.id.3953	rs4383094	15	C	T	0.149188097	0.032066228	4.28134E-06	5091	0.004233766	21.64574826
order.NB1n.id.3953	rs55921101	10	T	A	-0.109245446	0.023620072	4.32139E-06	5105	0.004172847	21.39164819
order.NB1n.id.3953	rs9542068	13	C	T	0.099070907	0.021814934	6.52339E-06	5107	0.004022243	20.62455005
order.NB1n.id.3953	rs2930903	19	G	C	0.090833483	0.020943373	6.8435E-06	4789	0.003912473	18.81042917
order.NB1n.id.3953	rs60775321	15	C	T	-0.096242537	0.021384172	7.1046E-06	5101	0.00395524	20.25579323
order.NB1n.id.3953	rs8126061	20	C	T	-0.159135032	0.035190375	7.36334E-06	5089	0.0040023	20.44955208
order.NB1n.id.3953	rs166849	2	A	G	0.09110531	0.020223942	7.73898E-06	5107	0.003957925	20.29344428
order.NB1n.id.3953	rs80270230	2	C	G	0.176113096	0.039321411	8.40763E-06	4261	0.004685693	20.05973159
order.Pasteurellales.id.3688	rs12191680	6	G	C	0.102213615	0.019718945	3.049E-07	9408	0.002847831	26.86891565
order.Pasteurellales.id.3688	rs9382510	6	T	C	-0.088186524	0.016996482	2.476E-07	9410	0.0028527	26.92070048
order.Pasteurellales.id.3688	rs10840326	11	G	C	-0.0717634	0.01492293	1.43842E-06	9154	0.002519945	23.12585676
order.Pasteurellales.id.3688	rs76022354	10	T	C	0.242889667	0.050040603	1.82858E-06	3692	0.006340869	23.55987662
order.Pasteurellales.id.3688	rs72756943	5	A	G	0.139879974	0.030302876	3.35218E-06	8020	0.002649822	21.30803366
order.Pasteurellales.id.3688	rs78909003	9	C	T	-0.241159469	0.049837229	2.04893E-06	3638	0.006395167	23.4153618
order.Pasteurellales.id.3688	rs6092684	20	T	A	0.068464719	0.014673987	3.44059E-06	9366	0.002318863	21.76894572
order.Pasteurellales.id.3688	rs4822728	22	C	T	0.068547871	0.014903232	4.71847E-06	9412	0.002242694	21.15568144
order.Pasteurellales.id.3688	rs35510	12	G	A	0.122729003	0.026495751	4.01576E-06	8671	0.002468309	21.45566408
order.Pasteurellales.id.3688	rs35509	12	A	G	0.122351799	0.026512106	4.39089E-06	8671	0.002450178	21.29767744
order.Pasteurellales.id.3688	rs10965428	9	A	C	-0.119902083	0.025821904	4.29151E-06	8873	0.00242411	21.56139886
order.Pasteurellales.id.3688	rs16970009	17	G	A	0.187381473	0.042957182	7.31531E-06	4703	0.004029519	19.02750021
order.Pasteurellales.id.3688	rs12050685	15	G	A	-0.067094787	0.015238983	9.18513E-06	9411	0.002055588	19.38498979
order.Pasteurellales.id.3688	rs73139353	3	C	A	-0.222572658	0.048463428	8.71314E-06	4047	0.005184712	21.09188361
order.Pasteurellales.id.3688	rs6972479	7	G	A	-0.078186189	0.01753656	7.74817E-06	8616	0.002301785	19.87793531
order.Pasteurellales.id.3688	rs9895850	17	C	T	-0.176406461	0.041016971	9.08251E-06	4737	0.003889609	18.4970219
order.Pasteurellales.id.3688	rs111582866	16	A	G	-0.113957275	0.025640298	7.06627E-06	8616	0.002287377	19.75322337
order.Pasteurellales.id.3688	rs62568866	9	A	T	-0.118018253	0.025920615	7.22663E-06	8020	0.002578174	20.73040357
order.Pasteurellales.id.3688	rs731534	20	G	C	-0.098650225	0.021631635	9.46292E-06	9115	0.002276517	20.79780299
order.Pasteurellales.id.3688	rs9938097	16	C	T	-0.071038642	0.015802434	8.22604E-06	9402	0.002144809	20.20883813
order.Rhodospirillales.id.2667	rs9813022	3	G	A	-0.083079452	0.016264151	3.07213E-07	8600	0.003024895	26.09302486
order.Rhodospirillales.id.2667	rs11630875	15	C	T	0.094519847	0.02021388	3.70379E-06	8595	0.002537449	21.86485648
order.Rhodospirillales.id.2667	rs76784716	2	G	A	0.136039348	0.02843993	1.31382E-06	8600	0.002653503	22.88084357
order.Rhodospirillales.id.2667	rs3754624	2	T	C	0.094128044	0.01983893	2.68367E-06	8598	0.002611371	22.5113514
order.Rhodospirillales.id.2667	rs1549633	5	C	A	0.099673509	0.02166668	3.88024E-06	8112	0.002602048	21.16287867
order.Rhodospirillales.id.2667	rs7001029	8	T	C	0.120725521	0.026098899	2.83071E-06	8063	0.002646709	21.39704564
order.Rhodospirillales.id.2667	rs1035406	5	A	G	-0.114724899	0.024799361	4.07163E-06	8585	0.002486639	21.40101572
order.Rhodospirillales.id.2667	rs4278423	10	C	T	0.105215078	0.023377775	3.9804E-06	8603	0.002348975	20.25581008
order.Rhodospirillales.id.2667	rs61933850	12	A	G	0.164662572	0.036028532	6.99876E-06	6846	0.003041842	20.88798997
order.Rhodospirillales.id.2667	rs77304857	4	A	C	-0.099743255	0.02221655	6.02031E-06	8602	0.002337749	20.15644114
order.Rhodospirillales.id.2667	rs55876211	3	T	C	-0.086500238	0.019519668	7.87313E-06	8596	0.002279304	19.63766031
order.Rhodospirillales.id.2667	rs74354280	4	T	C	-0.089064217	0.020309082	9.0088E-06	8603	0.00223052	19.23206414
order.Rhodospirillales.id.2667	rs11591293	10	T	G	0.072275477	0.01581858	4.68529E-06	8602	0.002421	20.87598257
order.Rhodospirillales.id.2667	rs4822789	22	C	G	0.072775517	0.01595216	7.32717E-06	8594	0.002415938	20.81285275
order.Rhodospirillales.id.2667	rs13336560	16	T	C	-0.069531323	0.0157065	9.74686E-06	8112	0.002410051	19.5975653
order.Rhodospirillales.id.2667	rs3730086	5	G	A	0.08003537	0.017873721	7.98252E-06	8602	0.002325538	20.05090453
order.Selenomonadales.id.2165	rs1643968	5	C	T	-0.056528439	0.011229806	4.14596E-07	18170	0.001392609	25.33899942
order.Selenomonadales.id.2165	rs60274479	16	C	T	-0.065962286	0.013413689	1.1623E-06	17610	0.001371323	24.18215135
order.Selenomonadales.id.2165	rs13086907	3	A	G	0.062531199	0.013173503	1.95488E-06	18163	0.001238982	22.53154506
order.Selenomonadales.id.2165	rs73232831	4	A	G	-0.151828859	0.03149318	1.87452E-06	9382	0.002471187	23.2421138

order.Selenomonadales.id.2165	rs71405394	15	A	G	-0.114177822	0.02404996	2.16631E-06	13861	0.001623434	22.53900806
order.Selenomonadales.id.2165	rs61249479	9	C	A	0.077707533	0.01686282	2.95471E-06	18165	0.001167678	21.23566348
order.Selenomonadales.id.2165	rs1447205	8	C	G	-0.050205329	0.010838203	2.93324E-06	18165	0.001179878	21.45779268
order.Selenomonadales.id.2165	rs4722181	7	G	T	0.050130576	0.010579796	2.00112E-06	18163	0.001234601	22.45178116
order.Selenomonadales.id.2165	rs4463806	10	C	T	-0.05439664	0.012936525	7.80662E-06	18160	0.000972681	17.68108324
order.Selenomonadales.id.2165	rs9423647	10	A	G	0.047817029	0.010528201	6.06114E-06	18164	0.001134364	20.62798538
order.Selenomonadales.id.2165	rs2834062	21	G	A	0.0489057	0.010884164	8.4392E-06	18171	0.001109859	20.18965087
order.Selenomonadales.id.2165	rs1649999	10	G	A	0.074898861	0.01664577	7.5812E-06	18036	0.001121283	20.24615865
order.Selenomonadales.id.2165	rs1135612	7	A	G	0.052978507	0.01191767	9.26307E-06	18164	0.001086758	19.76135468
order.Verrucomicrobiales.id.4030	rs11729256	4	C	T	0.074979802	0.015017677	6.7309E-07	11861	0.002097249	24.92774882
order.Verrucomicrobiales.id.4030	rs4936098	11	G	A	0.064884253	0.013592809	1.12221E-06	11858	0.001917852	22.78558435
order.Verrucomicrobiales.id.4030	rs9349825	6	G	A	-0.070403566	0.014712789	2.53687E-06	11858	0.001927303	22.89809198
order.Verrucomicrobiales.id.4030	rs11252894	10	C	A	0.077093323	0.016605195	2.93907E-06	11864	0.001813536	21.5548784
order.Verrucomicrobiales.id.4030	rs74542928	4	C	T	0.112163707	0.023642195	1.63222E-06	11216	0.002002721	22.50759662
order.Verrucomicrobiales.id.4030	rs3995795	10	T	C	0.064127531	0.014099468	5.18557E-06	11690	0.001766449	20.68633587
order.Verrucomicrobiales.id.4030	rs2602429	16	T	C	0.074684437	0.015619438	2.58297E-06	11690	0.00195194	22.86280045
order.Verrucomicrobiales.id.4030	rs4242783	10	A	G	0.068929219	0.014769297	2.63612E-06	11690	0.001859791	21.78146308
order.Verrucomicrobiales.id.4030	rs11184341	1	C	G	0.065534193	0.014223078	4.13405E-06	11864	0.001786246	21.22993935
order.Verrucomicrobiales.id.4030	rs61779207	1	A	G	-0.075830853	0.016776157	6.72285E-06	11591	0.001759629	20.43181414
order.Verrucomicrobiales.id.4030	rs117107102	18	G	A	0.20468288	0.043157251	2.91821E-06	4975	0.00450094	22.49342052
order.Verrucomicrobiales.id.4030	rs12908520	15	A	G	0.061892558	0.013094626	2.17163E-06	11863	0.001879659	22.34038678
order.Verrucomicrobiales.id.4030	rs111862613	12	C	T	0.090698957	0.019674596	3.73846E-06	11621	0.001825391	21.25166394
order.Verrucomicrobiales.id.4030	rs941682	20	A	G	-0.063143598	0.014376741	9.61003E-06	11809	0.001630857	19.29024644
order.Victivallales.id.2254	rs2825714	21	G	A	-0.137410136	0.02892458	1.72211E-06	4734	0.00474471	22.56853747
order.Victivallales.id.2254	rs17114848	15	A	G	0.152376929	0.032433167	4.05865E-06	4734	0.004640996	22.07291674
order.Victivallales.id.2254	rs77599476	20	G	A	0.230291775	0.048016811	1.86133E-06	3722	0.006142118	23.00224553
order.Victivallales.id.2254	rs11770843	7	T	C	0.109430892	0.023487897	1.90729E-06	4705	0.004592329	21.70658996
order.Victivallales.id.2254	rs72640280	1	G	A	0.220206945	0.048619648	5.18035E-06	3816	0.005346895	20.51343605
order.Victivallales.id.2254	rs62570196	9	T	C	-0.216350467	0.043986577	1.07926E-06	3712	0.006475095	24.19219835
order.Victivallales.id.2254	rs11764871	7	T	G	0.105796117	0.023346418	6.38503E-06	4725	0.004327268	20.5352007
order.Victivallales.id.2254	rs2731834	5	G	C	0.109437835	0.023692997	4.24358E-06	4733	0.004487508	21.3351157
order.Victivallales.id.2254	rs2031282	13	G	A	0.122368503	0.027032933	4.3826E-06	4734	0.004309719	20.4905185
order.Victivallales.id.2254	rs2546105	5	T	A	0.106187252	0.023469029	6.52724E-06	4734	0.004305784	20.47172918
order.Victivallales.id.2254	rs1002941	15	A	G	0.105025041	0.023348442	8.14836E-06	4734	0.00425588	20.2334482
order.Victivallales.id.2254	rs73113483	3	A	T	-0.131216985	0.0288713	8.66341E-06	4718	0.004359054	20.65605772
phylum.Actinobacteria.id.400	rs7570971	2	C	A	0.086653039	0.01136229	1.41347E-14	17782	0.003260146	58.16153359
phylum.Actinobacteria.id.400	rs182549	2	T	C	0.084637462	0.011486423	1.12717E-13	17990	0.003008956	54.2944807
phylum.Actinobacteria.id.400	rs4429415	2	T	C	0.058190752	0.011134335	2.05146E-07	17990	0.001515966	27.31362751
phylum.Actinobacteria.id.400	rs10841473	12	C	G	-0.059657241	0.011753696	4.46577E-07	17987	0.001430201	25.76187299
phylum.Actinobacteria.id.400	rs1530559	2	A	G	0.054705285	0.010896323	3.7753E-07	17782	0.001415477	25.20569292
phylum.Actinobacteria.id.400	rs74037001	14	A	G	-0.081939134	0.016533368	6.7124E-07	17179	0.001427716	24.56179279
phylum.Actinobacteria.id.400	rs55888705	4	G	A	0.053422107	0.010997529	1.31242E-06	17988	0.001310085	23.59672767
phylum.Actinobacteria.id.400	rs62448869	7	A	T	-0.052453124	0.010822585	1.13897E-06	17782	0.001319249	23.48987346
phylum.Actinobacteria.id.400	rs62448871	7	A	C	-0.051784373	0.010762435	1.3184E-06	17980	0.001285961	23.15135353
phylum.Actinobacteria.id.400	rs8047955	16	G	A	0.052111493	0.011119219	2.66203E-06	17980	0.001220109	21.96435616
phylum.Actinobacteria.id.400	rs34284163	16	T	A	-0.060331227	0.013685143	8.82113E-06	17987	0.001079339	19.43504918
phylum.Actinobacteria.id.400	rs9833771	3	C	T	0.049007706	0.010656419	4.0744E-06	17990	0.001174261	21.14979664
phylum.Actinobacteria.id.400	rs1397793	5	A	G	-0.052138694	0.011158738	3.73769E-06	17782	0.001226243	21.83183196
phylum.Actinobacteria.id.400	rs6496870	15	C	T	0.05107769	0.011380876	4.61743E-06	17990	0.001118392	20.14239559
phylum.Actinobacteria.id.400	rs857444	6	T	C	0.050731493	0.010988454	3.80349E-06	17988	0.001183545	21.31483916
phylum.Actinobacteria.id.400	rs12528285	6	T	C	0.08094693	0.018078413	5.68792E-06	17990	0.00111318	20.0484197
phylum.Actinobacteria.id.400	rs80124826	2	C	T	-0.124191142	0.027856576	8.75422E-06	11999	0.00165372	19.87585284
phylum.Actinobacteria.id.400	rs13192624	6	C	T	-0.052286185	0.011774241	9.32576E-06	17985	0.001095271	19.72005196
phylum.Actinobacteria.id.400	rs75211493	19	A	G	0.084078593	0.018473851	9.27186E-06	16605	0.001245879	20.71362
phylum.Actinobacteria.id.400	rs6743026	2	C	T	0.058944616	0.013462245	9.8833E-06	17875	0.001071376	19.17137829
phylum.Actinobacteria.id.400	rs11766971	7	C	T	0.047637595	0.010646046	9.39927E-06	17987	0.001111939	20.02271523
phylum.Bacteroidetes.id.905	rs73846128	3	G	A	-0.066426181	0.013348134	4.78296E-07	18335	0.001348871	24.76494849
phylum.Bacteroidetes.id.905	rs7631304	3	A	G	-0.065955837	0.013294921	4.89587E-07	18335	0.001340514	24.61132258
phylum.Bacteroidetes.id.905	rs73512608	13	A	G	-0.123117207	0.02367433	2.53891E-07	11350	0.002377129	27.04469789
phylum.Bacteroidetes.id.905	rs55773148	13	A	G	-0.122676962	0.023674576	2.84362E-07	11350	0.002360149	26.85106926
phylum.Bacteroidetes.id.905	rs111845179	14	C	T	0.104176948	0.021383712	6.47186E-07	16394	0.001445652	23.73433594
phylum.Bacteroidetes.id.905	rs73975615	17	A	G	-0.207162752	0.044262873	1.20411E-06	3350	0.006496341	21.90504518
phylum.Bacteroidetes.id.905	rs2032750	2	C	T	-0.051085659	0.010680423	1.70835E-06	17854	0.001279763	22.87816122
phylum.Bacteroidetes.id.905	rs7546249	1	A	T	-0.056917331	0.01183824	1.41931E-06	18334	0.001259245	23.11611033
phylum.Bacteroidetes.id.905	rs13291169	9	G	C	0.070092873	0.014875617	2.60991E-06	17514	0.001266083	22.20228724
phylum.Bacteroidetes.id.905	rs17343978	22	C	A	-0.055604102	0.012027223	7.2247E-06	18335	0.001164384	21.37385938
phylum.Bacteroidetes.id.905	rs929878	16	T	C	-0.054000099	0.012152588	6.5112E-06	18335	0.00107573	19.74474547
phylum.Bacteroidetes.id.905	rs72706335	1	C	T	-0.223193178	0.049338227	7.13111E-06	3820	0.005328574	20.46419918
phylum.Bacteroidetes.id.905	rs7999780	13	A	G	0.054363486	0.01240667	9.46652E-06	18325	0.001046659	19.20012799
phylum.Bacteroidetes.id.905	rs6586324	21	C	T	0.047779239	0.010543692	7.36878E-06	18332	0.001118915	20.53491979
phylum.Bacteroidetes.id.905	rs62531359	8	G	T	0.06577884	0.014988611	8.42316E-06	18321	0.001050133	19.25970519
phylum.Bacteroidetes.id.905	rs62575403	9	T	C	0.145423872	0.031107867	2.95953E-06	9669	0.002255116	21.85400003
phylum.Cyanobacteria.id.1500	rs789068	18	A	G	-0.111384961	0.021292193	1.57171E-07	7110	0.003834196	27.3660627
phylum.Cyanobacteria.id.1500	rs789069	18	C	A	-0.110945307	0.021326204	1.86418E-07	7110	0.003792025	27.06392352
phylum.Cyanobacteria.id.1500	rs9864379	3	C	T	-0.138933303	0.026804476	2.03334E-07	6922	0.003866199	26.86569431
phylum.Cyanobacteria.id.1500	rs76531781	7	C	T	-0.231867725	0.049339267	2.87336E-06	3658	0.00600119	22.08488871
phylum.Cyanobacteria.id.1500	rs7148504	14	T	G	0.080040381	0.017662085	6.62391E-06	7118	0.002876903	20.53687541
phylum.Cyanobacteria.id.1500	rs584122	6	T	C	-0.151773535	0.032682655	4.22775E-06	6922	0.00310581	21.56539612
phylum.Cyanobacteria.id.1500	rs2314810	17	G	C	-0.2182567	0.046457143	4.24763E-06	3934	0.005579124	22.07141459
phylum.Cyanobacteria.id.1500	rs2553290	4	T	A	0.096213138	0.02026167	3.61331E-06	7113	0.003160028	22.54853168
phylum.Cyanobacteria.id.1500	rs12555298	9	A	G	0.097432733	0.021935139	8.08628E-06	7096	0.002772742	19.73008711
phylum.Cyanobacteria.id.1500	rs2585223	15	C	T	0.111364409	0.024774324	8.85734E-06	6911	0.002915281	20.20641255
phylum.Cyanobacteria.id.1500	rs61972390	13	C	T	0.107345656	0.024246867	9.11371E-06	6486	0.0030128	19.60007354
phylum.Euryarchaeota.id.55	rs10202904	2	G	T	-0.115985759	0.023027088	6.1888E-07	3861	0.006528104	25.37063205
phylum.Euryarchaeota.id.55	rs76029318	13	C	T	0.214866479	0.043844209	1.04622E-06	3660	0.006519155	24.0166741
phylum.Euryarchaeota.id.55	rs73457410	13	G	A	0.208000696	0.042847395	2.3711E-06	3661	0.006395793	23.56572037
phylum.Euryarchaeota.id.55	rs73031978	3	G	C	-0.19752268	0.04144971	2.74069E-06	3501	0.006444526	22.70863045
phylum.Euryarchaeota.id.55	rs34928225	6	C	T	0.199769775	0.042542135	4.33093E-06	3483	0.006291102	22.05063029
phylum.Euryarchaeota.id.55	rs7635189	3	A	G	0.119983324	0.0259356	4.635E-06	3483	0.006107104	21.40174454
phylum.Euryarchaeota.id.55	rs77658038	6	C	A	-0.160156679	0.034085617	4.7542E-06	3810	0.005761208	22.0773934
phylum.Euryarchaeota.id.55	rs894996	4	A	C	0.203576769	0.044003319	5.11931E-06	3746	0.005681244	21.403538
phylum.Euryarchaeota.id.55	rs56131665	5	A	G	0.176758917	0.038578653	5.30389E-06	3736	0.005587637	20.99271236
phylum.Euryarchaeota.id.55	rs45498998	21	A	G	-0.131882333	0.029228979	5.32453E-06	3850	0.005260109	20.35850863
phylum.Euryarchaeota.id.55	rs6776814	3	C	T	-0.184814711	0.040382772	7.12742E-06	3546	0.005871978	20.94502179
phylum.Euryarchaeota.id.55	rs7015093	8	A	G	-0.118111053	0.02643893	7.1987E-06	3855	0.005150228	19.95691205
phylum.Euryarchaeota.id.55	rs11022995	11	A	G	-0.103729602	0.022888851	7.73001E-06	3864	0.005287106	20.53796583
phylum.Euryarchaeota.id.55	rs6508769	19	C	T	0.150528693	0.033709102	8.12354E-06	3483	0.00569261	19.94087477
phylum.Euryarchaeota.id.55	rs6064552	20	C	T	-0.123611632	0.02757488	9.3378E-06	3775	0.005295034	20.09515804
phylum.Firmicutes.id.1672	rs3792064	2	A	G	0.089734864	0.018253212	6.75498E-07	17812	0.001355009	24.16817432
phylum.Firmicutes.id.1672	rs10209007	2	C	G	0.089297405	0.018243532	7.39682E-07	17812	0.00134327	23.95851274
phylum.Firmicutes.id.1672	rs2009919	17	C	T	0.053645718	0.010629448	4.94716E-07	17859	0.001424205	25.4711573
phylum.Firmicutes.id.1672	rs112334273	21	A	G	0.062745897	0.012741527	9.26452E-07	18217	0.001329453	24.25089241
phylum.Firmicutes.id.1672	rs13421739	2	G	C	0.094394435	0.017964144	1.94644E-06	18340	0.001503236	27.61084657
phylum.Firmicutes.id.1672	rs7975768	12	G	C	0.051273172	0.010922627	2.82482E-06	17859	0.001232348	22.03566678
phylum.Firmicutes.id.1672	rs2332027	4	A	G	-0.048265611	0.010482301	4.04546E-06	18340	0.001154679	21.20129554
phylum.Firmicutes.id.1672	rs2273429	14	G	A	-0.070211154	0.01530628	9.26239E-06	18337	0.001146164	21.04131702
phylum.Firmicutes.id.1672	rs7247191	19	C	T	-0.071303301	0.015742403	4.73101E-06	18340	0.001117358	20.51526975
phylum.Firmicutes.id.1672	rs72771021	10	T	C	-0.141356654	0.030830333	5.12438E-06	9068	0.002312911	21.02209985
phylum.Firmicutes.id.1672	rs3852931	20	T	C	-0.048208647	0.010499991	4.52594E-06	18260	0.001153109	21.08007139
phylum.Firmicutes.id.1672	rs2547978	5	G	A	0.046580183	0.010507469	8.56845E-06	18338	0.001070506	19.65197243
phylum.Firmicutes.id.1672	rs36111799	10	T	C	-0.04807078	0.010542949	5.17441E-06	18226	0.001139336	20.78921703
phylum.Firmicutes.id.1672	rs12554342	9	C	G	-0.052570302	0.01169079	6.8085E-06	18332	0.001101805	20.22056156
phylum.Firmicutes.id.1672	rs72738886	5	C	T	0.086460893	0.019039766	7.67884E-06	16934	0.001216265	20.62130817
phylum.Firmicutes.id.1672	rs4750583	10	G	A	0.061600686	0.013793602	5.78565E-06	16934	0.001176373	19.94415803
phylum.Firmicutes.id.1672	rs6815608	4	C	T	0.093576464	0.021086864	7.23859E-06	16731	0.001175646	19.69288088
phylum.Firmicutes.id.1672	rs8085381	18	A	G	0.064729115	0.014621949	8.67253E-06	18339	0.001067454	19.59696382
phylum.Firmicutes.id.1672	rs56199908	9	C	T	-0.186193968	0.041050975	8.66656E-06	5618	0.003648506	20.57236662
phylum.Firmicutes.id.1672	rs7573799	2	G	C	-0.069844604	0.015393468	8.05164E-06	17859	0.001151424	20.58698664
phylum.Firmicutes.id.1672	rs6814436	4	C	T	0.068020505	0.015084366	6.80463E-06	18336	0.001107745	20.33412834
phylum.Lentisphaerae.id.2238	rs2825714	21	G	A	-0.138226357	0.028909621	1.49772E-06	4739	0.004800873	22.86108926
phylum.Lentisphaerae.id.2238	rs11770843	7	T	C	0.111895531	0.023477604	1.13813E-06	4709	0.004800642	22.7152721
phylum.Lentisphaerae.id.2238	rs77599476	20	G	A	0.229981464	0.047991818	1.90263E-06	3726	0.006125478	22.96419722
phylum.Lentisphaerae.id.2238	rs62570196	9	T	C	-0.217203229	0.043962139	9.64474E-07	3716	0.006526129	24.4104012
phylum.Lentisphaerae.id.2238	rs72640280	1	G	A	0.220185072	0.048594661	5.19196E-06	3820	0.005345735	20.53045793
phylum.Lentisphaerae.id.2238	rs11764871	7	T	G	0.10842739	0.023334631	3.70396E-06	4730	0.004543987	21.59116795
phylum.Lentisphaerae.id.2238	rs17114848	15	A	G	0.148972108	0.032416219	6.76994E-06	4739	0.004436774	21.11957639
phylum.Lentisphaerae.id.2238	rs1002941	15	A	G	0.107660547	0.023338177	4.3088E-06	4739	0.004470404	21.28037622
phylum.Lentisphaerae.id.2238	rs2731834	5	G	C	0.109934636	0.023684845	3.79601E-06	4738	0.004526501	21.5440816
phylum.Lentisphaerae.id.2238	rs60995569	10	G	T	-0.160620002	0.033680552	9.19393E-06	4737	0.004778124	22.74263931

phylum.Lentisphaerae.id.2238	rs2546105	5	T	A	0.106444588	0.023460355	7.01883E-06	4739	0.004325226	20.58628579
phylum.Lentisphaerae.id.2238	rs2031282	13	G	A	0.120411947	0.027026292	5.86463E-06	4739	0.004171231	19.85026181
phylum.Lentisphaerae.id.2238	rs73113483	3	A	T	-0.131681316	0.028850337	8.79384E-06	4723	0.004391543	20.83274633
phylum.Proteobacteria.id.2375	rs922773	3	T	C	-0.080377814	0.015785107	3.67958E-07	18132	0.001427944	25.92849706
phylum.Proteobacteria.id.2375	rs2532663	10	A	G	-0.125578004	0.025803359	7.46812E-07	13013	0.001816802	23.68506946
phylum.Proteobacteria.id.2375	rs12150865	19	T	C	0.051161535	0.010640719	1.54085E-06	17769	0.001299324	23.11773114
phylum.Proteobacteria.id.2375	rs2347697	7	T	G	0.050228084	0.010896783	4.26914E-06	18248	0.00116299	21.24695259
phylum.Proteobacteria.id.2375	rs312757	17	G	C	-0.051726884	0.011285376	4.839E-06	18250	0.001149842	21.00876786
phylum.Proteobacteria.id.2375	rs12467198	2	T	C	0.049826716	0.011137964	6.30669E-06	18238	0.001096123	20.01303167
phylum.Proteobacteria.id.2375	rs10750258	11	C	A	-0.049124751	0.010824018	8.71899E-06	18250	0.001127382	20.59793521
phylum.Proteobacteria.id.2375	rs6707783	2	T	C	0.084959348	0.018778235	8.08593E-06	17324	0.00118019	20.46976167
phylum.Proteobacteria.id.2375	rs72771021	10	T	C	0.141725865	0.03092421	7.18097E-06	9009	0.002326018	21.00395151
phylum.Proteobacteria.id.2375	rs3890996	22	G	T	-0.047461372	0.010564451	6.94663E-06	18120	0.001112615	20.18304798
phylum.Proteobacteria.id.2375	rs74757828	8	T	A	0.095128506	0.021692115	9.25067E-06	16634	0.001154833	19.23169395
phylum.Proteobacteria.id.2375	rs4340090	12	T	C	-0.066816186	0.015338746	9.99003E-06	18250	0.001038651	18.97508191
phylum.Proteobacteria.id.2375	rs11715072	3	A	G	-0.051928888	0.01152464	6.90409E-06	18249	0.001111326	20.30314452
phylum.Proteobacteria.id.2375	rs11126162	2	C	T	-0.077068857	0.018707864	9.25979E-06	18250	0.000929058	16.97107831
phylum.Tenericutes.id.3919	rs74603314	14	C	T	0.221639279	0.046291765	1.55689E-06	4499	0.005069472	22.92376615
phylum.Tenericutes.id.3919	rs10108398	8	A	G	0.076914215	0.015395275	1.09E-06	10932	0.002277971	24.95964108
phylum.Tenericutes.id.3919	rs11890098	2	G	A	0.074438031	0.015338858	9.56571E-07	11392	0.002063036	23.55069342
phylum.Tenericutes.id.3919	rs72901605	11	C	T	-0.084185237	0.01781192	3.2579E-06	10526	0.002117709	22.33831558
phylum.Tenericutes.id.3919	rs3768491	1	G	A	-0.068105197	0.01490611	4.23352E-06	11002	0.001893812	20.87525853
phylum.Tenericutes.id.3919	rs638542	1	A	G	-0.068036144	0.014955512	4.48578E-06	10999	0.00187805	20.69554296
phylum.Tenericutes.id.3919	rs17214486	14	A	C	0.060989958	0.013562263	6.60866E-06	11387	0.001772852	20.22331672
phylum.Tenericutes.id.3919	rs12566890	1	G	T	-0.101147132	0.023097845	3.65095E-06	11371	0.00168358	19.17627429
phylum.Tenericutes.id.3919	rs78169027	11	G	A	-0.108282943	0.023728915	5.8761E-06	10542	0.001971443	20.82400904
phylum.Tenericutes.id.3919	rs4885016	13	C	T	-0.081960567	0.018162779	7.26986E-06	11388	0.001784933	20.36316752
phylum.Tenericutes.id.3919	rs28537087	15	A	G	0.08208727	0.018831168	8.07399E-06	11382	0.001666687	19.00190425
phylum.Tenericutes.id.3919	rs2464826	7	C	A	0.094423946	0.021180616	8.39505E-06	10998	0.001803804	19.87408379
phylum.Tenericutes.id.3919	rs6043847	20	C	T	-0.11493717	0.024860602	4.55476E-06	11130	0.001916767	21.37458673
phylum.Verrucomicrobia.id.3982	rs74542928	4	C	T	0.115994333	0.023132372	4.08375E-07	11703	0.002143897	25.14393279
phylum.Verrucomicrobia.id.3982	rs11252894	10	C	A	0.078405494	0.016276917	1.10556E-06	12381	0.001870595	23.20324332
phylum.Verrucomicrobia.id.3982	rs2602429	16	T	C	0.076414823	0.015348672	8.7104E-07	12104	0.002043602	24.78640893
phylum.Verrucomicrobia.id.3982	rs11729256	4	C	T	0.069698414	0.01469534	2.23402E-06	12377	0.001814189	22.49502722
phylum.Verrucomicrobia.id.3982	rs4242783	10	A	G	0.068984922	0.014517714	1.75496E-06	12104	0.001861974	22.57937401
phylum.Verrucomicrobia.id.3982	rs45598138	1	A	C	-0.143901454	0.030540001	2.19127E-06	9932	0.002230416	22.20200813
phylum.Verrucomicrobia.id.3982	rs61779207	1	A	G	-0.075525262	0.016438461	5.27971E-06	12087	0.001743355	21.10873374
phylum.Verrucomicrobia.id.3982	rs76430504	5	C	T	-0.117530089	0.025458042	3.4956E-06	11624	0.001830193	21.31317342
phylum.Verrucomicrobia.id.3982	rs9349825	6	G	A	-0.066024311	0.014391139	6.27486E-06	12373	0.00169826	21.04831907
phylum.Verrucomicrobia.id.3982	rs117107102	18	G	A	0.204282775	0.042756505	2.67935E-06	5071	0.004481412	22.82753905
phylum.Verrucomicrobia.id.3982	rs3995795	10	T	C	0.061091431	0.013859187	9.72209E-06	12104	0.001602727	19.4305487
phylum.Verrucomicrobia.id.3982	rs12908520	15	A	G	0.059491573	0.012823012	3.40448E-06	12380	0.001735624	21.52438779
phylum.Verrucomicrobia.id.3982	rs12512971	4	C	A	0.171056895	0.03980534	9.81381E-06	3919	0.004690094	18.46709082

Supplementary Table 3. Used instrumental variables for 526 blood metabolites from MetabolomIPS database.

Notations: SNP, Single nucleotide polymorphism; R^2, proportion of variance in exposure variable explained by SNPs

Exposure	SNP	effect_allele.exposure	other_allel	eaf.exposure	beta.exposure	se.exposure	pval.exposure	mr_keep.exposu	Sample size	R^2	F
x-12435	rs17115338	T	C	0.9866	-0.6712	0.1168	9.042E-09	TRUE	231	0.125076649	33.02312817
x-12712	rs726389	T	C	0.9733	-0.31	0.0521	2.708E-09	TRUE	260	0.11984836	35.40364204
x-12712	rs707922	T	G	0.04	0.2854	0.0406	2.12E-12	TRUE	260	0.159703699	49.4146667
x-12189	rs10425480	A	G	0.0157	0.5394	0.0954	1.589E-08	TRUE	481	0.062320978	31.96871168
x-12189	rs8082167	T	C	0.0288	0.4457	0.0774	8.492E-09	TRUE	481	0.06449199	33.15914675
x-12728	rs12769714	T	C	0.011	-1.008	0.18	2.144E-08	TRUE	537	0.055176297	31.36
x-12728	rs1912320	A	G	0.0355	0.3974	0.0662	1.98E-09	TRUE	537	0.062886531	36.0362629
x-12728	rs16896783	A	G	0.989	1.024	0.179	1.061E-08	TRUE	537	0.057441763	32.72606972
x-12728	rs12326998	A	C	0.011	-1.038	0.18	8.085E-09	TRUE	537	0.058315099	33.25444444
x-12728	rs2830801	A	G	0.023	-1.072	0.179	2.114E-09	TRUE	537	0.062608086	35.86604663
x-12728	rs9566930	T	C	0.015	-0.989	0.176	1.917E-08	TRUE	537	0.055536456	31.57673683
x-12728	rs8089543	T	C	0.011	-1.029	0.179	8.998E-09	TRUE	537	0.057971488	33.0464405
x-12728	rs13094553	A	G	0.985	0.975	0.177	3.62E-08	TRUE	537	0.053483125	30.34329216
x-12040	rs11736351	C	G	0.0263	0.516	0.092	2.022E-08	TRUE	559	0.053276432	31.45746692
x-12040	rs12508311	A	T	0.9577	-0.4363	0.0793	3.706E-08	TRUE	559	0.051369948	30.27081064
n-(2-furoyl)glycine	rs2277503	A	G	0.0502	0.1667	0.0302	3.429E-08	TRUE	604	0.048022741	30.46893777
x-12188	rs1899124	T	C	0.051	0.304	0.055	3.252E-08	TRUE	832	0.035419068	30.5507438
x-12188	rs12335217	T	C	0.013	0.323	0.057	1.456E-08	TRUE	832	0.037160859	32.11111111
estrone 3-sulfate	rs2912546	T	C	0.0501	0.2679	0.0469	1.154E-08	TRUE	957	0.032970646	32.62869781
x-13658	rs1028074	T	C	0.8585	-0.0838	0.0148	1.522E-08	TRUE	1412	0.022201348	32.06008035
x-12729	rs9619080	C	G	0.0106	0.3568	0.065	3.981E-08	TRUE	1753	0.016898166	30.13165444
x-12704	rs1470777	T	C	0.5972	-0.0515	0.0087	3.726E-09	TRUE	1813	0.018961136	35.04095653
x-12013	rs1681529	A	G	0.0182	0.4453	0.0799	2.489E-08	TRUE	1948	0.015694689	31.06074239
cysteine-glutathione disulfide	rs5760492	A	G	0.333	-0.0492	0.007	2.339E-12	TRUE	1997	0.024140342	49.40081633
x-11792	rs4253311	A	G	0.479	-0.0845	0.0089	1.719E-21	TRUE	2442	0.035599601	90.14328999
x-11792	rs4253252	T	G	0.4803	-0.0862	0.0089	2.827E-22	TRUE	2442	0.036992897	93.80684257
x-11792	rs698078	A	G	0.5789	-0.0636	0.0111	1.015E-08	TRUE	2442	0.013265479	32.82980278
x-11792	rs2731672	T	C	0.257	-0.12	0.02	1.973E-09	TRUE	2442	0.014527845	36
inosine	rs494562	A	G	0.8988	-0.1182	0.0167	1.339E-12	TRUE	2675	0.018383162	50.0958801
x-14086	rs4351	A	G	0.4739	-0.0343	0.0053	1.188E-10	TRUE	2686	0.01535362	41.88287647
x-14086	rs4362	T	C	0.5277	0.0345	0.0055	4.594E-10	TRUE	2686	0.014437466	39.34710744
1-linoleoylglycerol (1-monolinolein	rs964184	C	G	0.8592	-0.0508	0.009	1.64E-08	TRUE	2797	0.011262401	31.85975309
1-linoleoylglycerol (1-monolinolein	rs11825181	A	G	0.0766	0.0761	0.0114	2.934E-11	TRUE	2797	0.01568204	44.56148046
x-12093	rs10469966	A	G	0.2526	0.1024	0.0068	1.117E-51	TRUE	2854	0.07360767	226.7681661
x-12093	rs7096654	T	C	0.6444	0.078	0.0062	1.042E-36	TRUE	2854	0.052542599	158.2726327
x-12093	rs4488133	A	T	0.7014	0.0985	0.0067	1.26E-48	TRUE	2854	0.070398846	216.1338828
x-12093	rs7573275	A	G	0.2971	0.0744	0.0066	2.605E-29	TRUE	2854	0.042627041	127.0743802
x-12093	rs10206899	T	C	0.7555	-0.101	0.0067	8.089E-51	TRUE	2854	0.073750845	227.2443751
x-12093	rs13538	A	G	0.7571	-0.1005	0.0068	4.504E-50	TRUE	2854	0.07109387	218.4310121
x-11858	rs885479	A	G	0.0535	0.1621	0.028	7.004E-09	TRUE	3427	0.009685212	33.51582908
1,6-anhydroglucose	rs10120252	A	G	0.8189	-0.0484	0.0087	3.031E-08	TRUE	3663	0.008378404	30.94939886
x-12740	rs4001461	T	C	0.4402	0.0513	0.009	1.387E-08	TRUE	3731	0.008632944	32.49
adpsgegdfxaegggvr*	rs651007	T	C	0.2099	0.0653	0.0071	6.344E-20	TRUE	3939	0.02102307	84.58817695
adpsgegdfxaegggvr*	rs601338	A	G	0.4316	0.0413	0.0062	2.524E-11	TRUE	3939	0.011139502	44.37278876
adpsgegdfxaegggvr*	rs649129	T	C	0.2181	0.0623	0.0069	2.089E-19	TRUE	3939	0.020276613	81.52257929
aspartylphenylalanine	rs4351	A	G	0.4754	-0.0455	0.005	1.24E-19	TRUE	3948	0.020544258	82.81
aspartylphenylalanine	rs4362	T	C	0.5251	0.0442	0.0051	4.304E-18	TRUE	3948	0.018669907	75.11111111
homocitrulline	rs8101881	T	C	0.613	0.0325	0.0039	1.434E-16	TRUE	4135	0.016516913	69.44444444
x-14662	rs2199680	A	T	0.8296	-0.0501	0.0088	1.026E-08	TRUE	4212	0.007636468	32.41231921
x-14662	rs4149081	A	G	0.1715	0.0463	0.0081	9.475E-09	TRUE	4212	0.007697464	32.67322055
x-14662	rs1871395	A	G	0.8372	-0.0497	0.0088	1.728E-08	TRUE	4212	0.007515928	31.89682335
x-14662	rs4149056	T	C	0.8367	-0.049	0.0087	2.185E-08	TRUE	4212	0.007474924	31.72149557
x-14662	rs12317268	A	G	0.8372	-0.0497	0.0088	1.767E-08	TRUE	4212	0.007515928	31.89682335
x-14662	rs7969341	A	G	0.8289	-0.0479	0.0081	2.898E-09	TRUE	4212	0.008234206	34.97043134
bradykinin, des-arg(9)	rs4253311	A	G	0.4869	-0.1412	0.0097	3.559E-48	TRUE	4570	0.044312439	211.8975449
bradykinin, des-arg(9)	rs4253252	T	G	0.4889	-0.141	0.0097	5.302E-48	TRUE	4570	0.044192541	211.2976937
bradykinin, des-arg(9)	rs5030062	A	C	0.6147	-0.0718	0.01	5.935E-13	TRUE	4570	0.01115478	51.5524
bradykinin, des-arg(9)	rs698078	A	G	0.5732	-0.0663	0.0113	4.377E-09	TRUE	4570	0.007476439	34.42470045
bradykinin, des-arg(9)	rs2731672	T	C	0.257	-0.169	0.017	2.756E-23	TRUE	4570	0.021167413	98.82698962
bradykinin, des-arg(9)	rs4253236	T	C	0.362	-0.146	0.015	2.174E-22	TRUE	4570	0.020309347	94.73777778
x-11483	rs17057690	T	C	0.0555	0.0831	0.0142	5.001E-09	TRUE	4608	0.007377294	34.24722277
x-11905	rs2199680	A	T	0.8319	-0.0768	0.0057	1.76E-41	TRUE	4761	0.036730135	181.5401662
x-11905	rs4149006	T	G	0.1473	-0.0501	0.0068	1.493E-13	TRUE	4761	0.011272906	54.28222318
x-11905	rs4149002	A	G	0.1449	-0.048	0.0068	1.306E-12	TRUE	4761	0.010357261	49.82698962
x-11905	rs4149081	A	G	0.1723	0.0782	0.0057	5.369E-43	TRUE	4761	0.038030069	188.2191444
x-11905	rs12829704	A	G	0.191	-0.0566	0.0058	2.804E-22	TRUE	4761	0.019609999	95.23067776
x-11905	rs1871395	A	G	0.8379	-0.0818	0.0057	1.833E-46	TRUE	4761	0.041463629	205.9476762
x-11905	rs11045975	A	G	0.1856	-0.0407	0.0067	1.318E-09	TRUE	4761	0.007691091	36.90109156
x-11905	rs4149056	T	C	0.8367	-0.0827	0.0057	1.078E-47	TRUE	4761	0.042342205	210.5044629
x-11905	rs12826576	A	G	0.1631	-0.0319	0.0059	4.851E-08	TRUE	4761	0.006102681	29.2332663
x-11905	rs12317268	A	G	0.838	-0.0818	0.0057	1.84E-46	TRUE	4761	0.041463629	205.9476762
x-11905	rs7969341	A	G	0.8286	-0.0792	0.0057	5.35E-44	TRUE	4761	0.038970777	193.0637119
x-12456	rs2199680	A	T	0.8252	-0.0746	0.0058	1.357E-37	TRUE	4774	0.03349227	165.4328181
x-12456	rs2547231	A	C	0.833	0.0409	0.0061	2.824E-11	TRUE	4774	0.009328976	44.95592583
x-12456	rs4149006	T	G	0.1436	-0.0447	0.0069	9.336E-11	TRUE	4774	0.008714316	41.96786389
x-12456	rs296396	T	C	0.1592	-0.0431	0.0067	1.673E-10	TRUE	4774	0.008593582	41.3813767
x-12456	rs4149002	A	G	0.1417	-0.0429	0.0068	3.559E-10	TRUE	4774	0.008268155	39.80125433
x-12456	rs4149081	A	G	0.177	0.0757	0.0058	8.896E-39	TRUE	4774	0.034452979	170.347503
x-12456	rs12829704	A	G	0.1892	-0.0564	0.0061	1.469E-20	TRUE	4774	0.017591713	85.48669712
x-12456	rs1871395	A	G	0.833	-0.0793	0.0059	8.693E-42	TRUE	4774	0.036461053	180.6518242
x-12456	rs4149056	T	C	0.832	-0.0805	0.0058	9.491E-44	TRUE	4774	0.038785867	192.6352556
x-12456	rs12317268	A	G	0.833	-0.0793	0.0059	9.95E-42	TRUE	4774	0.036461053	180.6518242

x-12456	rs296381	T	C	0.8332	0.0412	0.0068	1.021E-09	TRUE	4774	0.007630755	36.70934256
x-12456	rs7969341	A	G	0.8232	-0.0751	0.0058	3.238E-38	TRUE	4774	0.03392745	167.6578478
x-11799	rs3099557	A	G	0.8504	-0.1484	0.0113	1.825E-39	TRUE	5038	0.033100466	172.4689482
x-12855	rs1171614	T	C	0.2232	-0.0263	0.0047	2.549E-08	TRUE	5120	0.006078521	31.31235853
x-12855	rs11161521	T	C	0.6957	-0.0247	0.0043	7.635E-09	TRUE	5120	0.006403202	32.99567334
x-12855	rs1171617	T	G	0.776	0.0263	0.0047	2.616E-08	TRUE	5120	0.006078521	31.31235853
x-12855	rs7552404	A	G	0.6963	-0.0246	0.0043	9.02E-09	TRUE	5120	0.006351788	32.72904273
x-12855	rs12136754	A	C	0.3343	0.026	0.0043	1.235E-09	TRUE	5120	0.007090056	36.56030287
x-12855	rs1171615	T	C	0.7768	0.0263	0.0047	2.571E-08	TRUE	5120	0.006078521	31.31235853
laurylcarnitine	rs2070630	C	G	0.2936	-0.0327	0.0055	2.093E-09	TRUE	5170	0.00679079	35.34842975
laurylcarnitine	rs4690909	C	G	0.6902	0.0301	0.0049	1.063E-09	TRUE	5170	0.007245894	37.73469388
laurylcarnitine	rs8396	T	C	0.6977	0.0299	0.0049	1.445E-09	TRUE	5170	0.007150609	37.23490212
deoxycholate	rs8192870	T	G	0.3498	-0.0365	0.0067	3.985E-08	TRUE	5194	0.005681457	29.67810203
n2,n2-dimethylguanosine	rs7749940	A	C	0.3736	0.0109	0.0019	8.299E-09	TRUE	5228	0.006255828	32.91135734
n2,n2-dimethylguanosine	rs7203065	T	G	0.3934	0.0114	0.0019	1.998E-09	TRUE	5228	0.006838906	36
bilirubin (e,z or z,e)*	rs887829	T	C	0.3243	0.081	0.0051	1.792E-56	TRUE	5295	0.045472833	252.2491349
bilirubin (e,z or z,e)*	rs4148324	T	G	0.675	-0.0795	0.0051	5.674E-55	TRUE	5295	0.043877462	242.9930796
x-13741	rs12189736	T	C	0.8963	-0.0642	0.0114	1.559E-08	TRUE	5325	0.005920547	31.71468144
x-11334	rs2080403	C	G	0.4643	-0.0149	0.0027	3.816E-08	TRUE	5462	0.005544707	30.45404664
x-11334	rs9424148	A	C	0.7912	-0.0201	0.0036	1.609E-08	TRUE	5462	0.005674973	31.17361111
adsgegdfxaegggvr*	rs2731672	T	C	0.257	-0.081	0.011	1.79E-13	TRUE	5588	0.009610244	54.2231405
adsgegdfxaegggvr*	rs4253236	T	C	0.362	-0.071	0.01	1.248E-12	TRUE	5588	0.008940464	50.41
hydroxyisovaleroyl carnitine	rs2270968	T	G	0.2634	0.0307	0.0037	1.307E-16	TRUE	5588	0.012170236	68.84514244
hydroxyisovaleroyl carnitine	rs274570	T	C	0.2962	0.0224	0.0037	1.779E-09	TRUE	5588	0.006516238	36.65157049
1-oleoylglycerol (1-monoolein)	rs964184	C	G	0.8615	-0.0443	0.0073	1.202E-09	TRUE	5717	0.006400368	36.82660912
x-13477	rs10469966	A	G	0.2301	-0.0246	0.0033	7.164E-14	TRUE	5952	0.009250037	55.57024793
x-13477	rs7573275	A	G	0.2787	-0.0207	0.0031	2.831E-11	TRUE	5952	0.00743555	44.58792924
x-13477	rs10206899	T	C	0.7805	0.025	0.0033	1.725E-14	TRUE	5952	0.009550401	57.39210285
x-13477	rs13538	A	G	0.7805	0.0243	0.0033	8.492E-14	TRUE	5952	0.009027827	54.2231405
x-13548	rs12246027	A	G	0.2666	0.0115	0.0018	1.045E-10	TRUE	6022	0.006732497	40.81790123
x-13548	rs622034	T	C	0.128	0.0141	0.0026	4.198E-08	TRUE	6022	0.004859985	29.40976331
tetradecanedioate	rs2199680	A	T	0.8275	-0.096	0.0061	2.929E-55	TRUE	6046	0.039353056	247.6753561
tetradecanedioate	rs4149006	T	G	0.1453	-0.0536	0.0073	1.754E-13	TRUE	6046	0.008838128	53.91180334
tetradecanedioate	rs4149002	A	G	0.1431	-0.0535	0.0072	1.343E-13	TRUE	6046	0.009049537	55.21315586
tetradecanedioate	rs4149081	A	G	0.1753	0.0972	0.0061	1.297E-56	TRUE	6046	0.040303132	253.9059393
tetradecanedioate	rs12829704	A	G	0.1882	-0.0698	0.0064	7.519E-28	TRUE	6046	0.01929397	118.9462891
tetradecanedioate	rs1871395	A	G	0.834	-0.1	0.0062	3.222E-59	TRUE	6046	0.041252723	260.1456816
tetradecanedioate	rs11045975	A	G	0.1867	-0.0506	0.0063	1.391E-15	TRUE	6046	0.010557049	64.50894432
tetradecanedioate	rs4149056	T	C	0.8325	-0.0994	0.0061	3.398E-59	TRUE	6046	0.042070577	265.5296963
tetradecanedioate	rs12826576	A	G	0.162	-0.0411	0.0065	1.848E-10	TRUE	6046	0.006569409	39.98130178
tetradecanedioate	rs9333029	A	G	0.8706	0.0553	0.0074	5.263E-14	TRUE	6046	0.009152203	55.84532505
tetradecanedioate	rs12317268	A	G	0.8342	-0.1008	0.0062	4.161E-60	TRUE	6046	0.041887649	264.3246618
tetradecanedioate	rs10770772	A	G	0.7925	0.0398	0.0063	3.209E-10	TRUE	6046	0.00655782	39.91030486
tetradecanedioate	rs7306033	A	G	0.8511	0.0415	0.0064	1.069E-10	TRUE	6046	0.006906504	42.04711914
tetradecanedioate	rs1572603	A	G	0.13	-0.054	0.008	1.478E-11	TRUE	6046	0.007479608	45.5625
tetradecanedioate	rs6663731	A	T	0.8857	0.0624	0.0081	1.385E-14	TRUE	6046	0.009720504	59.34705075
tetradecanedioate	rs7969341	A	G	0.8249	-0.0984	0.0061	5.41E-58	TRUE	6046	0.041263098	260.213921
tetradecanedioate	rs4149022	A	G	0.2454	-0.0407	0.0055	1.051E-13	TRUE	6046	0.008975931	54.76
phenyllactate (pla)	rs4784054	A	G	0.1063	0.0349	0.0037	2.31E-21	TRUE	6064	0.014459809	88.97078159
alpha-ketoglutarate	rs7036192	A	T	0.9301	0.0835	0.0152	3.497E-08	TRUE	6130	0.004898832	30.17767486
3-phenylpropionate (hydrocinnamat	rs11647589	A	G	0.7151	0.0303	0.0045	2.854E-11	TRUE	6182	0.007280443	45.33777778
3-phenylpropionate (hydrocinnamat	rs6497490	T	G	0.882	-0.0477	0.0075	1.911E-10	TRUE	6182	0.006500591	40.4496
3-phenylpropionate (hydrocinnamat	rs1394678	T	C	0.2765	-0.0295	0.0053	1.942E-08	TRUE	6182	0.004986459	30.98077608
x-12850	rs182420	T	C	0.7559	0.0392	0.0063	6.517E-10	TRUE	6251	0.006155453	38.71604938
x-12850	rs2547231	A	C	0.8319	0.059	0.0072	3.523E-16	TRUE	6251	0.010627942	67.14891975
x-12850	rs296396	T	C	0.1597	-0.0567	0.0079	9.847E-13	TRUE	6251	0.008173315	51.51241788
x-12850	rs279941	A	C	0.1171	-0.0478	0.0081	4.31E-09	TRUE	6251	0.005540175	34.82456943
x-12850	rs11019976	C	G	0.0717	0.1331	0.0234	1.361E-08	TRUE	6251	0.005149118	32.35373292
x-12850	rs296381	T	C	0.8308	0.0572	0.0072	2.568E-15	TRUE	6251	0.009995733	63.11419753
x-13215	rs1339995	A	C	0.4714	0.011	0.0019	1.631E-08	TRUE	6305	0.005287988	33.51800554
x-13215	rs7499892	T	C	0.175	-0.017	0.003	1.456E-08	TRUE	6305	0.005067153	32.11111111
x-13215	rs11076175	A	G	0.845	0.017	0.003	1.456E-08	TRUE	6305	0.005067153	32.11111111
x-13429	rs2199680	A	T	0.8266	-0.1271	0.0079	3.306E-58	TRUE	6344	0.039201793	258.8432943
x-13429	rs2547231	A	C	0.8321	0.051	0.0083	6.856E-10	TRUE	6344	0.005916215	37.75584265
x-13429	rs4149006	T	G	0.1453	-0.0822	0.0091	1.995E-19	TRUE	6344	0.012698357	81.59449342
x-13429	rs296396	T	C	0.1609	-0.0498	0.0089	2.383E-08	TRUE	6344	0.004911084	31.30968312
x-13429	rs4149002	A	G	0.1434	-0.0829	0.0091	6.568E-20	TRUE	6344	0.012912747	82.99009781
x-13429	rs4149081	A	G	0.1766	0.1295	0.0079	1.125E-60	TRUE	6344	0.040635519	268.7109438
x-13429	rs12829704	A	G	0.1879	-0.1001	0.0083	7.346E-34	TRUE	6344	0.022413213	145.4494121
x-13429	rs1871395	A	G	0.8337	-0.137	0.0079	7.869E-67	TRUE	6344	0.045259437	300.7370614
x-13429	rs11045975	A	G	0.1868	-0.072	0.0082	1.229E-18	TRUE	6344	0.012006822	77.09696609
x-13429	rs4149056	T	C	0.8328	-0.1365	0.0079	2.784E-67	TRUE	6344	0.0449445	298.5459061
x-13429	rs12317268	A	G	0.8337	-0.1377	0.0079	2.091E-67	TRUE	6344	0.045701933	303.8181381
x-13429	rs10770772	A	G	0.7907	0.0554	0.0088	3.605E-10	TRUE	6344	0.006208494	39.63274793
x-13429	rs7306033	A	G	0.8528	0.0493	0.009	4.749E-08	TRUE	6344	0.004707565	30.00604938
x-13429	rs296381	T	C	0.8302	0.053	0.0082	1.071E-10	TRUE	6344	0.006541997	41.77572873
x-13429	rs7969341	A	G	0.8241	-0.1295	0.0079	1.541E-60	TRUE	6344	0.040635519	268.7109438
x-13429	rs4149022	A	G	0.2444	-0.0547	0.0073	6.105E-14	TRUE	6344	0.008772815	56.14730719
x-01911	rs4680	A	G	0.5065	-0.0436	0.0059	1.309E-13	TRUE	6360	0.008513315	54.60959494
x-12450	rs2898615	C	G	0.4547	-0.0159	0.0029	4.446E-08	TRUE	6432	0.004651866	30.06064209
dodecanedioate	rs1572603	A	G	0.13	-0.036	0.0064	1.464E-08	TRUE	6478	0.004860579	31.640625
scyllo-inositol	rs4787294	A	T	0.9278	0.075	0.008	9.644E-21	TRUE	6500	0.013341239	87.890625
scyllo-inositol	rs4788439	T	C	0.075	-0.0697	0.0079	1.316E-18	TRUE	6500	0.011833902	77.84153181
x-11452	rs9348689	T	G	0.5165	0.0339	0.0062	4.413E-08	TRUE	6519	0.004565075	29.89620187
2-hydroxyisobutyrate	rs493519	T	C	0.1252	-0.0358	0.0041	1.324E-18	TRUE	6539	0.011525308	76.24271267
2-hydroxyisobutyrate	rs11635023	A	G	0.508	-0.0152	0.0028	4.248E-08	TRUE	6539	0.004486492	29.46938776
2-hydroxyisobutyrate	rs11160112	T	C	0.7128	-0.0146	0.0026	2.337E-08	TRUE	6539	0.004799085	31.53254438
x-11491	rs2199680	A	T	0.8256	-0.0877	0.0075	6.828E-32	TRUE	6584	0.020345106	136.7340444
x-11491	rs4149081	A	G	0.1765	0.085	0.0075	4.149E-30	TRUE	6584	0.019135271	128.4444444
x-11491	rs12829704	A	G	0.1906	-0.0613	0.0078	3.073E-15	TRUE	6584	0.009293662	61.76347798
x-11491	rs1871395	A	G	0.8337	-0.0866	0.0075	1.342E-30	TRUE	6584	0.019848005	133.3255111
x-11491	rs4149056	T	C	0.8325	-0.0881	0.0075	3.423E-32	TRUE	6584	0.020527299	137.9841778
x-11491	rs12317268	A	G	0.8337	-0.0874	0.0075	4.471E-31	TRUE	6584	0.020208961	135.8001778
x-11491	rs7306033	A	G	0.8517	0.0438	0.0079	2.922E-08	TRUE	6584	0.004647092	30.7393046
x-11491	rs12645107	A	G	0.1473	0.0463	0.0079	4.611E-09	TRUE	6584	0.00518989	34.34850184
x-11491	rs7969341	A	G	0.8239	-0.0865	0.0075	4.356E-31	TRUE	6584	0.0198031	133.0177778
x-11478	rs11647589	A	G	0.7186	0.0404	0.0047	1.302E-17	TRUE	6593	0.011082658	73.88682662
x-11478	rs6497490	T	G	0.8816	-0.0824	0.0077	4.954E-27	TRUE	6593	0.017073051	114.5177939
x-11478	rs1394678	T	C	0.2749	-0.0433	0.0055	6.208E-15	TRUE	6593	0.009313302	61.97983471
x-11529	rs2199680	A	T	0.8133	-0.2765	0.0079	1E-200	TRUE	6664	0.155279503	1225
x-11529	rs4149006	T	G	0.1425	-0.1261	0.0096	3.171E-39	TRUE	6664	0.025237795	172.539171
x-11529	rs4149002	A	G	0.1405	-0.1244	0.0095	6.92E-39	TRUE	6664	0.025085631	171.472133
x-11529	rs4149081	A	G	0.1895	0.2793	0.0079	1E-200	TRUE	6664	0.157941104	1249.935747
x-11529	rs12829704	A	G	0.1833	-0.1582	0.0088	2.769E-72	TRUE	6664	0.0462536	323.1823347
x-11529	rs1871395	A	G	0.8199	-0.2952	0.0079	1E-200	TRUE	6664	0.173231695	1396.299311
x-11529	rs11045975	A	G	0.1839	-0.1194	0.0092	1.793E-38	TRUE	6664	0.024652302	168.4352552
x-11529	rs4149056	T	C	0.8182	-0.2952	0.0078	1E-200	TRUE	6664	0.176911159	1432.331361
x-11529	rs12826576	A	G	0.1589	-0.0687	0.0089	8.845E-15	TRUE	6664	0.008862017	59.58452216
x-11529	rs12317268	A	G	0.82	-0.2951	0.0079	1E-200	TRUE	6664	0.173134666	1395.353469
x-11529	rs10770772	A	G	0.7952	0.075	0.0093	5.858E-16	TRUE	6664	0.009665042	65.0364204
x-11529	rs7306033	A	G	0.8543	0.0618	0.0095	6.578E-11	TRUE	6664	0.006310236	42.31844875
x-11529	rs7969341	A	G	0.8106	-0.2801	0.0078	1E-200	TRUE	6664	0.16213478	1289.546515
x-11529	rs4149022	A	G	0.2396	-0.0732	0.0078	5.998E-21	TRUE	6664	0.013043554	88.07100592
biliverdin	rs887829	T	C	0.3411	0.1131	0.0041	2.5E-168	TRUE	6686	0.102183063	760.9524093
biliverdin	rs4148324	T	G	0.6595	-0.1101	0.0041	7.94E-160	TRUE	6686	0.097354853	721.1189768
x-13069	rs8022857	T	C	0.9764	0.0772	0.0136	1.45E-08	TRUE	6770	0.004737028	32.22231834
x-06307	rs3783200	A	C	0.6859	-0.0414	0.0073	1.514E-08	TRUE	6774	0.004725553	32.16288234
x-10429	rs4073054	A	C	0.6266	0.0162	0.0027	2.148E-09	TRUE	6807	0.005260851	36
bilirubin (z,z)	rs887829	T	C	0.3265	0.1677	0.0064	2.15E-152	TRUE	6812	0.091564214	686.6037598
bilirubin (z,z)	rs4148324	T	G	0.6731	-0.1617	0.0064	4.9E-142	TRUE	6812	0.08568076	638.3518066
x-14473	rs4986894	T	C	0.838	-0.0421	0.0061	5.549E-12	TRUE	6884	0.006871776	47.63262564
hexadecanedioate	rs2199680	A	T	0.8249	-0.0838	0.0058	4.519E-47	TRUE	6887	0.029419384	208.7526754
hexadecanedioate	rs4149006	T	G	0.1461	-0.0569	0.0068	9.232E-17	TRUE	6887	0.010064301	70.0175173
hexadecanedioate	rs4149002	A	G	0.1447	-0.0554	0.0062	2.079E-19	TRUE	6887	0.011460409	79.84287201
hexadecanedioate	rs4149081	A	G	0.1777	0.0843	0.0058	9.672E-48	TRUE	6887	0.029761019	211.2511891
hexadecanedioate	rs12829704	A	G	0.1891	-0.0682	0.0061	1.976E-29	TRUE	6887	0.017826545	124.9997313
hexadecanedioate	rs1871395	A	G	0.8326	-0.0893	0.0059	1.649E-52	TRUE	6887	0.032192722	229.0861821
hexadecanedioate	rs11045975	A	G	0.1891	-0.0536	0.006	4.082E-19	TRUE	6887	0.011454957	79.80444444
hexadecanedioate	rs4149056	T	C	0.8318	-0.0877	0.0058	1.652E-51	TRUE	6887	0.032131391	228.6352556

hexadecanedioate	rs12826576	A	G	0.1616	-0.0408	0.0061	2.818E-11	TRUE	6887	0.006453846	44.73636119
hexadecanedioate	rs9333029	A	G	0.8692	0.041	0.0069	2.43E-09	TRUE	6887	0.005100569	35.30770846
hexadecanedioate	rs12317268	A	G	0.8326	-0.0893	0.0059	1.73E-52	TRUE	6887	0.032192722	229.0861821
hexadecanedioate	rs10770772	A	G	0.7892	0.0415	0.006	4.427E-12	TRUE	6887	0.006898541	47.84027778
hexadecanedioate	rs7306033	A	G	0.8518	0.0396	0.0061	9.281E-11	TRUE	6887	0.006082066	42.14350981
hexadecanedioate	rs6663731	A	T	0.8823	0.0452	0.0069	5.492E-11	TRUE	6887	0.006192285	42.91199328
hexadecanedioate	rs7969341	A	G	0.8228	-0.0858	0.0058	2.668E-49	TRUE	6887	0.030796646	218.8359096
hexadecanedioate	rs4149022	A	G	0.2452	-0.0421	0.0052	5.799E-16	TRUE	6887	0.009427869	65.5477071
1-eicosadienoylglycerophosphochol	rs174550	T	C	0.6636	-0.0195	0.0035	2.343E-08	TRUE	6892	0.004483697	31.04081633
1-eicosadienoylglycerophosphochol	rs174535	T	C	0.6635	-0.0194	0.0035	2.916E-08	TRUE	6892	0.004438032	30.72326531
1-eicosadienoylglycerophosphochol	rs1535	A	G	0.662	-0.0203	0.0035	6.146E-09	TRUE	6892	0.004857313	33.64
2-linoleoylglycerophosphocholine*	rs174450	T	G	0.5386	-0.0164	0.0027	9.759E-10	TRUE	6894	0.005323177	36.89437586
x-14626	rs2199680	A	T	0.8371	-0.05	0.0038	6.167E-40	TRUE	6904	0.024463333	173.1301939
x-14626	rs4149006	T	G	0.1481	-0.0257	0.0047	4.982E-08	TRUE	6904	0.004312141	29.89995473
x-14626	rs4149002	A	G	0.1465	-0.0243	0.0038	2.442E-10	TRUE	6904	0.005888163	40.89265928
x-14626	rs4149081	A	G	0.1671	0.0497	0.0038	1.78E-39	TRUE	6904	0.02417773	171.0588643
x-14626	rs12829704	A	G	0.1923	-0.0257	0.0038	1.867E-11	TRUE	6904	0.006581585	45.74030471
x-14626	rs1871395	A	G	0.8428	-0.0527	0.0038	5.887E-44	TRUE	6904	0.027103167	192.3331025
x-14626	rs11045975	A	G	0.1879	-0.0236	0.0038	5.929E-10	TRUE	6904	0.005555671	38.57063712
x-14626	rs4149056	T	C	0.8424	-0.0525	0.0038	6.903E-44	TRUE	6904	0.026903365	190.8760388
x-14626	rs12317268	A	G	0.8428	-0.0536	0.0038	2.202E-45	TRUE	6904	0.028010645	198.9584488
x-14626	rs7969341	A	G	0.8337	-0.0504	0.0038	1.166E-40	TRUE	6904	0.024846548	175.9113573
4-acetamidobutanoate	rs7228099	T	C	0.4364	-0.0097	0.0018	3.609E-08	TRUE	6930	0.004173007	29.04012346
4-acetamidobutanoate	rs721399	T	C	0.7176	-0.0289	0.0018	3.948E-58	TRUE	6930	0.035863762	257.7808642
x-11412	rs3764261	A	C	0.3354	0.0113	0.0019	3.393E-09	TRUE	6935	0.005074506	35.37119114
succinylcarnitine	rs10988217	A	G	0.3883	-0.0164	0.0019	1.363E-18	TRUE	6948	0.010609343	74.50415512
succinylcarnitine	rs924135	A	T	0.3828	-0.0167	0.0018	1.522E-19	TRUE	6948	0.012237165	86.07716049
succinylcarnitine	rs2062541	A	G	0.6153	0.0159	0.0018	6.874E-18	TRUE	6948	0.011105532	78.02777778
succinylcarnitine	rs17806888	T	C	0.8806	-0.0224	0.0034	7.154E-11	TRUE	6948	0.006208315	43.40484429
succinylcarnitine	rs1472631	A	G	0.4919	0.0366	0.0018	1.847E-88	TRUE	6948	0.056163494	413.4444444
succinylcarnitine	rs8060756	A	G	0.3703	0.0102	0.0018	4.066E-08	TRUE	6948	0.004600372	32.11111111
succinylcarnitine	rs875740	A	C	0.6652	0.0147	0.0019	2.621E-15	TRUE	6948	0.008541657	59.85872576
succinylcarnitine	rs6703518	T	G	0.4273	-0.0108	0.0019	5.848E-09	TRUE	6948	0.00462877	32.31024931
succinylcarnitine	rs2686513	T	C	0.552	-0.011	0.002	3.798E-08	TRUE	6948	0.004334898	30.25
succinylcarnitine	rs246234	C	G	0.2951	-0.0171	0.0026	5.198E-11	TRUE	6948	0.006187145	43.25591716
1-stearoylglycerol (1-monostearin)	rs6662425	C	G	0.6385	0.0148	0.0027	3.512E-08	TRUE	6965	0.004295417	30.04663923
x-13435	rs924135	A	T	0.3834	-0.0187	0.0027	6.923E-12	TRUE	6970	0.006835091	47.96844993
x-13435	rs2062541	A	G	0.615	0.0196	0.0027	5.758E-13	TRUE	6970	0.00750379	52.69684499
x-13435	rs875740	A	C	0.6652	0.018	0.0027	6.324E-11	TRUE	6970	0.006336132	44.44444444
x-13435	rs9392556	T	C	0.6581	-0.0358	0.0028	4.028E-38	TRUE	6970	0.022916531	163.4744898
levulinate (4-oxovalerate)	rs711995	T	C	0.4481	0.0108	0.0019	2.704E-08	TRUE	6982	0.004606333	32.31024931
levulinate (4-oxovalerate)	rs13278849	A	G	0.73	-0.013	0.002	2.786E-11	TRUE	6982	0.006014877	42.25
2-tetradecenoyl carnitine	rs273914	A	T	0.3688	0.0269	0.0048	1.989E-08	TRUE	6998	0.0044679	31.40668403
2-tetradecenoyl carnitine	rs273913	T	C	0.3808	0.0238	0.0043	2.52E-08	TRUE	6998	0.00435859	30.6349378
2-tetradecenoyl carnitine	rs272869	A	G	0.3702	0.0248	0.0042	5.033E-09	TRUE	6998	0.004957611	34.86621315
2-tetradecenoyl carnitine	rs274567	T	C	0.3704	0.0257	0.0042	1.268E-09	TRUE	6998	0.005322017	37.44274376
2-tetradecenoyl carnitine	rs272881	A	G	0.6208	-0.0251	0.0042	3.479E-09	TRUE	6998	0.005077666	35.71485261
x-13859	rs12098046	C	G	0.4684	0.0118	0.0019	7.745E-10	TRUE	7002	0.00547834	38.57063712
x-13859	rs2708257	A	C	0.6508	-0.0108	0.0019	2.169E-08	TRUE	7002	0.004593236	32.31024931
x-11441	rs887829	T	C	0.3285	0.1089	0.0048	3.53E-114	TRUE	7072	0.067845192	514.7226563
x-11441	rs4148324	T	G	0.6706	-0.1091	0.0048	1.52E-116	TRUE	7072	0.068077642	516.6150174
glycochenodeoxycholate	rs4870490	A	G	0.066	0.0857	0.0145	3.143E-09	TRUE	7087	0.004904873	34.93217598
n-acetylglycine	rs10164524	A	G	0.6793	0.0388	0.0051	2.091E-14	TRUE	7135	0.008046747	57.8792772
n-acetylglycine	rs7948073	T	G	0.5702	-0.0256	0.0044	4.604E-09	TRUE	7135	0.004721989	33.85123967
n-acetylglycine	rs715	T	C	0.7096	-0.091	0.0057	6.658E-58	TRUE	7135	0.034490205	254.8784241
x-11442	rs887829	T	C	0.3296	0.1093	0.0048	3.65E-116	TRUE	7142	0.067686198	518.5108507
x-11442	rs4148324	T	G	0.6696	-0.109	0.0047	5.37E-117	TRUE	7142	0.070033336	537.8451788
x-12749	rs6151429	T	C	0.9293	-0.0249	0.0035	5.375E-13	TRUE	7178	0.007001767	50.61306122
stearoylcarnitine	rs273914	A	T	0.3714	0.0213	0.0033	1.213E-10	TRUE	7183	0.005766521	41.66115702
stearoylcarnitine	rs419291	T	C	0.3716	0.0205	0.0034	9.695E-10	TRUE	7183	0.005035604	36.35380623
stearoylcarnitine	rs273913	T	C	0.3854	0.0213	0.0033	1.901E-10	TRUE	7183	0.005766521	41.66115702
stearoylcarnitine	rs272869	A	G	0.3736	0.0213	0.0033	1.327E-10	TRUE	7183	0.005766521	41.66115702
stearoylcarnitine	rs274567	T	C	0.3744	0.0211	0.0033	1.562E-10	TRUE	7183	0.005659347	40.88246097
stearoylcarnitine	rs274570	T	C	0.2963	0.0199	0.0034	7.836E-09	TRUE	7183	0.004746529	34.25692042
stearoylcarnitine	rs272881	A	G	0.617	-0.0214	0.0033	1.112E-10	TRUE	7183	0.005820477	42.05325987
x-11521	rs16833668	A	C	0.3149	0.0211	0.0038	2.914E-08	TRUE	7193	0.004268056	30.83171745
x-12063	rs2572023	A	G	0.4685	-0.0324	0.005	1.013E-10	TRUE	7197	0.005800588	41.9904
x-12063	rs2199680	A	T	0.831	-0.1117	0.0065	2.174E-66	TRUE	7197	0.039415209	295.3110059
x-12063	rs2547231	A	C	0.8302	0.0392	0.0067	6.152E-09	TRUE	7197	0.004733804	34.2312319
x-12063	rs4149006	T	G	0.1476	-0.0548	0.0074	1.623E-13	TRUE	7197	0.007562223	54.84002922
x-12063	rs296396	T	C	0.1628	-0.0409	0.0067	1.055E-09	TRUE	7197	0.005151131	37.26464691
x-12063	rs4917639	A	C	0.8015	-0.0362	0.006	1.394E-09	TRUE	7197	0.005032365	36.40111111
x-12063	rs4149002	A	G	0.1466	-0.0536	0.0074	3.216E-13	TRUE	7197	0.007237027	52.46457268
x-12063	rs4149081	A	G	0.1711	0.1111	0.0065	6.948E-66	TRUE	7197	0.03900938	292.1469822
x-12063	rs1934963	T	C	0.8015	-0.0362	0.006	1.395E-09	TRUE	7197	0.005032365	36.40111111
x-12063	rs12829704	A	G	0.1937	-0.0624	0.0066	5.43E-21	TRUE	7197	0.012267865	89.38842975
x-12063	rs1871395	A	G	0.8385	-0.1155	0.0065	9.947E-70	TRUE	7197	0.042027986	315.7455621
x-12063	rs11045975	A	G	0.1921	-0.0455	0.0066	5.437E-12	TRUE	7197	0.006560318	47.52640037
x-12063	rs4149056	T	C	0.8379	-0.1176	0.0065	1.258E-73	TRUE	7197	0.04350308	327.3315976
x-12063	rs10242455	A	G	0.9335	0.2212	0.0099	1.67E-109	TRUE	7197	0.06486671	499.2290583
x-12063	rs12317268	A	G	0.8385	-0.1154	0.0065	1.315E-69	TRUE	7197	0.041958294	315.1990533
x-12063	rs10770772	A	G	0.7853	0.0431	0.0066	6.092E-11	TRUE	7197	0.005890463	42.64485767
x-12063	rs6544713	T	C	0.3211	-0.0314	0.0051	8.97E-10	TRUE	7197	0.005239454	37.90695886
x-12063	rs296381	T	C	0.8295	0.0398	0.0067	2.868E-09	TRUE	7197	0.004879113	35.28714636
x-12063	rs7969341	A	G	0.8291	-0.1112	0.0065	7.003E-66	TRUE	7197	0.03907689	292.6731361
x-12063	rs4149022	A	G	0.2477	-0.0355	0.0058	1.11E-09	TRUE	7197	0.005178386	37.46284185
x-11470	rs2734331	A	G	0.9651	0.0726	0.0101	6.559E-13	TRUE	7271	0.007056041	51.66905205
x-11470	rs559555	A	T	0.56	0.0308	0.0035	6.535E-19	TRUE	7271	0.010538291	77.44
beta-hydroxyisovalerate	rs10945649	A	T	0.1287	0.0212	0.0036	4.291E-09	TRUE	7283	0.004739073	34.67901235
hypoxanthine	rs6459467	A	G	0.3888	0.0202	0.0025	1.931E-16	TRUE	7287	0.008879741	65.2864
octadecanedioate	rs4149006	T	G	0.1472	-0.0357	0.0053	1.138E-11	TRUE	7300	0.006176905	45.37166251
octadecanedioate	rs4149002	A	G	0.1465	-0.0347	0.0052	2.461E-11	TRUE	7300	0.00606301	44.52995562
octadecanedioate	rs4149081	A	G	0.1725	0.0286	0.005	9.381E-09	TRUE	7300	0.004461974	32.7184
octadecanedioate	rs12829704	A	G	0.1927	-0.042	0.0045	9.493E-21	TRUE	7300	0.011792311	87.11111111
octadecanedioate	rs2108622	T	C	0.288	-0.0248	0.0044	0.000000012	TRUE	7300	0.004333006	31.76859504
octadecanedioate	rs1871395	A	G	0.8367	-0.0309	0.005	8.678E-10	TRUE	7300	0.005204606	38.1924
octadecanedioate	rs4149056	T	C	0.8363	-0.0286	0.005	1.044E-08	TRUE	7300	0.004461974	32.7184
octadecanedioate	rs12317268	A	G	0.8368	-0.0302	0.005	1.996E-09	TRUE	7300	0.004972629	36.4816
octadecanedioate	rs7969341	A	G	0.8276	-0.0291	0.005	5.303E-09	TRUE	7300	0.004618624	33.8724
octadecanedioate	rs4149022	A	G	0.2472	-0.0254	0.0043	4.517E-09	TRUE	7300	0.00475704	34.89237426
x-13671	rs2041073	T	C	0.5366	0.0178	0.0025	3.412E-13	TRUE	7311	0.006886241	50.6944
5alpha-androstan-3beta,17beta-diol	rs182420	T	C	0.7557	0.062	0.0075	1.301E-16	TRUE	7345	0.00921822	68.33777778
5alpha-androstan-3beta,17beta-diol	rs2547231	A	C	0.8298	0.081	0.0084	3.349E-22	TRUE	7345	0.012501329	92.98469388
5alpha-androstan-3beta,17beta-diol	rs296396	T	C	0.164	-0.0856	0.0088	3.841E-22	TRUE	7345	0.01271837	94.61983471
5alpha-androstan-3beta,17beta-diol	rs4149081	A	G	0.1694	0.0459	0.0081	1.64E-08	TRUE	7345	0.004352803	32.11111111
5alpha-androstan-3beta,17beta-diol	rs10278040	A	G	0.0419	-0.1901	0.0168	1.171E-29	TRUE	7345	0.01713359	128.0400014
5alpha-androstan-3beta,17beta-diol	rs4149056	T	C	0.8386	-0.0509	0.0086	3.995E-09	TRUE	7345	0.004746577	35.02988102
5alpha-androstan-3beta,17beta-diol	rs11761528	T	C	0.0739	-0.127	0.0127	1.23E-23	TRUE	7345	0.013431833	100
5alpha-androstan-3beta,17beta-diol	rs11981478	T	C	0.086	-0.1201	0.0115	1.265E-25	TRUE	7345	0.014631777	109.0662382
5alpha-androstan-3beta,17beta-diol	rs9632722	A	G	0.909	0.1126	0.0114	4.351E-23	TRUE	7345	0.013108252	97.55894121
5alpha-androstan-3beta,17beta-diol	rs296381	T	C	0.829	0.0833	0.0088	4.326E-21	TRUE	7345	0.012052214	89.60343492
5alpha-androstan-3beta,17beta-diol	rs7969341	A	G	0.8308	-0.0477	0.0081	4.423E-09	TRUE	7345	0.004699258	34.67901235
dimethylarginine (sdma + adma)	rs37379	A	G	0.0867	0.0147	0.0027	3.434E-08	TRUE	7347	0.004018356	29.64197531
x-06246	rs6053126	A	G	0.253	-0.022	0.004	3.798E-08	TRUE	7358	0.004094339	30.25
1-stearoylglycerophosphoethanolam	rs1077835	A	G	0.7949	-0.029	0.0043	1.185E-11	TRUE	7369	0.006134486	45.48404543
1-stearoylglycerophosphoethanolam	rs588136	T	C	0.7961	-0.0293	0.0043	8.301E-12	TRUE	7369	0.006261264	46.42996214
1-stearoylglycerophosphoethanolam	rs2070895	A	G	0.2113	0.0301	0.0048	2.726E-10	TRUE	7369	0.005307996	39.32335069
indolelactate	rs15676	A	G	0.7123	0.0191	0.0027	1.326E-12	TRUE	7378	0.006736973	50.04252401
malate	rs7503429	A	C	0.4255	-0.016	0.0026	1.635E-09	TRUE	7384	0.005102464	37.86982249
malate	rs4625783	T	C	0.4266	-0.0153	0.0026	6.097E-09	TRUE	7384	0.004667803	34.62869822
x-11247	rs4917639	A	C	0.8018	-0.0522	0.0087	2.059E-09	TRUE	7403	0.00483936	36
x-11247	rs1853207	T	C	0.0598	0.1085	0.0152	9.575E-13	TRUE	7403	0.006835742	50.95329813
x-11247	rs1934963	T	C	0.8018	-0.0523	0.0087	2.038E-09	TRUE	7403	0.004857829	36.13806315
x-11530	rs887829	T	C	0.3321	0.1116	0.0044	1.96E-140	TRUE	7409	0.079891823	643.3140496
x-11530	rs4148324	T	G	0.6668	-0.1106	0.0044	3.2E-140	TRUE	7409	0.078578486	631.8367769
x-12696	rs3800993	T	C	0.1701	0.0224	0.0036	4.062E-10	TRUE	7409	0.005198379	38.71604938
x-12696	rs7570971	A	C	0.3257	-0.0292	0.0026	3.03E-28	TRUE	7409	0.016738951	126.1301775
x-12696	rs12465802	A	G	0.2879	-0.0302	0.0026	2.351E-30	TRUE	7409	0.017884231	134.9171598
x-12627	rs7773173	C	G	0.5344	-0.027	0.0043	2.749E-10	TRUE	7419	0.005286198	39.42671714

x-12627	rs4713169	C	G	0.4244	-0.0322	0.0042	2.865E-14	TRUE	7419	0.007860327	58.77777778
2-methylbutyroylcarnitine	rs662138	C	G	0.8312	0.0187	0.0034	3.648E-08	TRUE	7420	0.004060266	30.25
tryptophan betaine	rs2405522	A	G	0.1672	-0.1264	0.0113	4.492E-29	TRUE	7439	0.016541644	125.1230323
x-12094	rs6430553	T	C	0.4007	-0.0204	0.0035	4.809E-09	TRUE	7467	0.004529046	33.9722449
gamma-glutamyltyrosine	rs12192718	A	T	0.1412	0.0142	0.0025	2.096E-08	TRUE	7468	0.004301503	32.2624
gamma-glutamyltyrosine	rs1512342	T	C	0.3393	0.0095	0.0017	4.659E-08	TRUE	7468	0.004164212	31.2283737
gamma-glutamyltyrosine	rs875740	A	C	0.6656	0.0102	0.0018	6.345E-09	TRUE	7468	0.004281418	32.11111111
gamma-glutamyltyrosine	rs9940990	A	T	0.3545	-0.0104	0.0018	4.745E-09	TRUE	7468	0.004450208	33.38271605
gamma-glutamyltyrosine	rs956966	A	G	0.4858	-0.0101	0.0018	1.475E-08	TRUE	7468	0.004198231	31.4845679
x-12556	rs4675874	A	G	0.7094	-0.0243	0.0026	3.134E-20	TRUE	7483	0.011538513	87.35059172
x-12556	rs2235649	T	C	0.2159	-0.0184	0.0027	1.951E-11	TRUE	7483	0.006168014	46.44170096
x-12092	rs8101881	T	C	0.6089	0.0281	0.005	2.569E-08	TRUE	7500	0.004193593	31.5844
x-12092	rs4919234	T	G	0.3296	0.0288	0.0051	1.516E-08	TRUE	7500	0.004233901	31.88927336
x-12092	rs12411523	T	C	0.3183	-0.032	0.0051	5.231E-10	TRUE	7500	0.005221852	39.36947328
x-12092	rs1889459	C	G	0.1628	-0.0412	0.0067	9.936E-10	TRUE	7500	0.005016484	37.81332145
x-12092	rs7096654	T	C	0.6211	0.2537	0.0047	1E-200	TRUE	7500	0.279795064	2913.70258
x-12092	rs4488133	A	T	0.664	0.2824	0.0045	1E-200	TRUE	7500	0.344305851	3938.259753
pentadecanoate (15:0)	rs6887589	A	G	0.7787	-0.0142	0.0026	4.33E-08	TRUE	7502	0.003960314	29.82840237
1-arachidonoylglycerophosphocholi	rs174550	T	C	0.6606	0.0559	0.0027	5.01E-94	TRUE	7507	0.054014946	428.6433471
1-arachidonoylglycerophosphocholi	rs174535	T	C	0.6602	0.0561	0.0027	1.86E-94	TRUE	7507	0.054381092	431.7160494
1-arachidonoylglycerophosphocholi	rs174538	A	G	0.3099	-0.0518	0.0027	9.833E-80	TRUE	7507	0.046738793	368.0713306
1-arachidonoylglycerophosphocholi	rs968567	T	C	0.1832	-0.0344	0.0036	3.289E-21	TRUE	7507	0.012016969	91.30864198
1-arachidonoylglycerophosphocholi	rs2727271	A	T	0.8937	0.0546	0.0044	7.015E-35	TRUE	7507	0.020099964	153.9855372
1-arachidonoylglycerophosphocholi	rs174556	T	C	0.3047	-0.0528	0.0027	9.506E-83	TRUE	7507	0.048472482	382.4197531
1-arachidonoylglycerophosphocholi	rs174578	A	T	0.3445	-0.0541	0.0027	3.379E-88	TRUE	7507	0.050766103	401.4828532
1-arachidonoylglycerophosphocholi	rs1535	A	G	0.6589	0.0551	0.0027	2.25E-91	TRUE	7507	0.052560644	416.4622771
1-arachidonoylglycerophosphocholi	rs174548	C	G	0.6941	0.0526	0.0027	3.083E-82	TRUE	7507	0.048123599	379.5281207
1-arachidonoylglycerophosphocholi	rs174601	T	C	0.3412	-0.0548	0.0034	1.525E-57	TRUE	7507	0.0334474	259.7785467
1-arachidonoylglycerophosphocholi	rs3738544	T	C	0.8071	0.0203	0.0035	8.051E-09	TRUE	7507	0.00446116	33.64
1-arachidonoylglycerophosphocholi	rs174450	T	G	0.5383	0.0218	0.0027	1.128E-15	TRUE	7507	0.008609222	65.19067215
1-arachidonoylglycerophosphocholi	rs748196	T	G	0.4596	0.0152	0.0027	0.000000017	TRUE	7507	0.004204009	31.69272977
x-14625	rs12550729	T	C	0.9238	0.0284	0.0038	5.227E-14	TRUE	7511	0.00738166	55.85595568
x-14625	rs7822232	A	C	0.9203	0.0299	0.0038	5.116E-15	TRUE	7511	0.008175461	61.91204986
x-12798	rs3799344	T	C	0.4519	0.0197	0.0034	8.939E-09	TRUE	7552	0.004425744	33.57179931
x-12798	rs12356193	A	G	0.8347	-0.0396	0.0044	3.273E-19	TRUE	7552	0.010611817	81
x-12798	rs316020	A	G	0.0937	-0.1799	0.0053	1E-200	TRUE	7552	0.132368307	1152.154147
x-12798	rs1165196	A	G	0.5469	-0.0189	0.0034	3.028E-08	TRUE	7552	0.004075026	30.90051903
x-12798	rs316019	A	C	0.0935	-0.1813	0.0053	1E-200	TRUE	7552	0.134159059	1170.156283
x-12798	rs315988	T	C	0.2324	-0.0887	0.0036	2.12E-132	TRUE	7552	0.074404863	607.0748457
x-12798	rs11754288	A	G	0.4387	0.0194	0.0034	1.342E-08	TRUE	7552	0.00429255	32.55709343
x-12798	rs1171614	T	C	0.221	0.0476	0.0039	3.19E-34	TRUE	7552	0.019343699	148.9651545
x-12798	rs1171617	T	G	0.7783	-0.0467	0.0039	4.468E-33	TRUE	7552	0.018632631	143.3852728
x-12798	rs1171615	T	C	0.7791	-0.0476	0.0039	3.186E-34	TRUE	7552	0.019343699	148.9651545
n-acetylornithine	rs6546815	A	T	0.8703	-0.0366	0.0063	4.595E-09	TRUE	7574	0.00443634	33.75056689
n-acetylornithine	rs7573275	A	G	0.2739	-0.1511	0.0042	1E-200	TRUE	7574	0.145945478	1294.286281
n-acetylornithine	rs10469966	A	G	0.2285	-0.2148	0.0048	1E-200	TRUE	7574	0.209110785	2002.5625
n-acetylornithine	rs10206899	T	C	0.7815	0.2211	0.0047	1E-200	TRUE	7574	0.22611642	2213.001811
n-acetylornithine	rs13538	A	G	0.7814	0.2189	0.0047	1E-200	TRUE	7574	0.222635817	2169.181077
cortisone	rs10938815	T	G	0.5932	0.0099	0.0017	8.381E-09	TRUE	7575	0.004457075	33.91349481
pantothenate	rs1395	A	G	0.7034	0.0172	0.0027	8.138E-11	TRUE	7604	0.005308547	40.58161866
indoleacetate	rs7809234	A	T	0.8755	0.0285	0.0045	1.604E-10	TRUE	7618	0.005237729	40.11111111
indoleacetate	rs6497490	T	G	0.8823	-0.0305	0.0055	3.487E-08	TRUE	7618	0.004020534	30.75206612
3-methyl-2-oxobutyrate	rs1440581	T	C	0.4567	-0.0122	0.0021	6.546E-09	TRUE	7648	0.004393604	33.75056689
x-13496	rs10069896	A	C	0.1781	0.0104	0.0018	1.417E-08	TRUE	7656	0.004341404	33.38271605
x-13496	rs795213	A	G	0.3339	0.01	0.0018	2.954E-08	TRUE	7656	0.004015187	30.86419753
cis-4-decenoyl carnitine	rs2070630	C	G	0.2946	-0.027	0.0043	3.366E-10	TRUE	7660	0.005120734	39.42671714
cis-4-decenoyl carnitine	rs11161521	T	C	0.6983	0.0608	0.0036	1.217E-63	TRUE	7660	0.035900081	285.2345679
cis-4-decenoyl carnitine	rs4690909	C	G	0.6883	0.0251	0.0037	8.443E-12	TRUE	7660	0.005971919	46.01972243
cis-4-decenoyl carnitine	rs7552404	A	G	0.6985	0.0602	0.0036	2.579E-62	TRUE	7660	0.035219855	279.632716
cis-4-decenoyl carnitine	rs12136754	A	C	0.3316	-0.0526	0.0036	7.767E-48	TRUE	7660	0.027114369	213.4845679
cis-4-decenoyl carnitine	rs8396	T	C	0.6961	0.0262	0.0037	8.96E-13	TRUE	7660	0.006503345	50.14170928
gamma-glutamylglutamine	rs2657879	A	G	0.8262	0.0238	0.0027	5.854E-19	TRUE	7662	0.010039272	77.70096022
gamma-glutamylglutamine	rs1751956	T	G	0.8647	0.016	0.0029	2.353E-08	TRUE	7662	0.003957126	30.43995244
gamma-glutamylglutamine	rs1260326	T	C	0.4122	-0.0121	0.0021	5.608E-09	TRUE	7662	0.004314319	33.19954649
x-18601	rs2199680	A	T	0.8328	-0.0415	0.0065	2.174E-10	TRUE	7663	0.005291351	40.76331361
x-18601	rs296396	T	C	0.1648	-0.0399	0.0072	3.105E-08	TRUE	7663	0.003991581	30.71006944
x-18601	rs4149081	A	G	0.1694	0.0386	0.0065	3.525E-09	TRUE	7663	0.004580944	35.26532544
x-18601	rs1871395	A	G	0.84	-0.0384	0.0066	6.204E-09	TRUE	7663	0.004398063	33.85123967
x-18601	rs10278040	A	G	0.042	-0.0763	0.0131	5.702E-09	TRUE	7663	0.004407469	33.92395548
x-18601	rs4149056	T	C	0.8395	-0.0393	0.0065	1.933E-09	TRUE	7663	0.004747803	36.55597633
x-18601	rs11761528	T	C	0.0738	-0.0601	0.0098	8.118E-10	TRUE	7663	0.004883955	37.60943357
x-18601	rs12317268	A	G	0.84	-0.039	0.0066	3.672E-09	TRUE	7663	0.004535948	34.91735537
x-18601	rs11981478	T	C	0.0862	-0.0562	0.0091	7.154E-10	TRUE	7663	0.004952617	38.14080425
x-18601	rs9632722	A	G	0.9087	0.0571	0.0085	1.517E-11	TRUE	7663	0.005854442	45.12678201
x-18601	rs296381	T	C	0.8282	0.039	0.0067	4.825E-09	TRUE	7663	0.004402149	33.88282468
x-18601	rs7969341	A	G	0.8308	-0.0382	0.0065	5.183E-09	TRUE	7663	0.004486918	34.53822485
alpha-hydroxyisovalerate	rs12141041	T	C	0.4692	-0.0253	0.0036	1.774E-12	TRUE	7668	0.006399788	49.38966049
alpha-hydroxyisovalerate	rs2403254	T	C	0.5505	-0.0409	0.0036	1.258E-30	TRUE	7668	0.016554265	129.0748457
alpha-hydroxyisovalerate	rs893971	A	G	0.5882	-0.0197	0.0036	4.05E-08	TRUE	7668	0.003890027	29.94521605
x-11327	rs4541909	T	C	0.674	0.01	0.0018	4.649E-08	TRUE	7671	0.004007367	30.86419753
x-11327	rs12191817	T	G	0.684	0.0104	0.0018	1.664E-08	TRUE	7671	0.004332951	33.38271605
x-11440	rs182420	T	C	0.7554	0.1193	0.0057	3.45E-96	TRUE	7686	0.053921031	438.0575562
x-11440	rs2547231	A	C	0.8292	0.1576	0.0065	1.19E-129	TRUE	7686	0.071052065	587.8759763
x-11440	rs296396	T	C	0.1652	-0.1643	0.0069	1.02E-124	TRUE	7686	0.068701389	566.9920185
x-11440	rs3732218	A	G	0.0805	-0.0532	0.0089	2.769E-09	TRUE	7686	0.00462731	35.73084207
x-11440	rs296381	T	C	0.8285	0.1636	0.0064	5.38E-143	TRUE	7686	0.078355537	653.4414063
x-11440	rs4715354	A	G	0.478	0.0278	0.0049	1.656E-08	TRUE	7686	0.004170442	32.18825489
hwesasxx*	rs7535263	A	G	0.435	-0.0443	0.0079	2.202E-08	TRUE	7700	0.004067172	31.44512097
hwesasxx*	rs2007084	A	G	0.091	-0.0683	0.0097	1.771E-12	TRUE	7700	0.00639764	49.57902009
glutaroyl carnitine	rs924135	A	T	0.3838	-0.0153	0.0026	2.311E-09	TRUE	7701	0.00447652	34.62869822
glutaroyl carnitine	rs2062541	A	G	0.6147	0.0154	0.0026	1.893E-09	TRUE	7701	0.004534962	35.08284024
glutaroyl carnitine	rs8056893	A	C	0.7336	-0.0302	0.0026	2.227E-30	TRUE	7701	0.017217788	134.9171598
glutaroyl carnitine	rs1981524	T	C	0.197	0.016	0.0027	3.149E-09	TRUE	7701	0.004539306	35.11659808
glutaroyl carnitine	rs2291449	A	G	0.914	-0.0276	0.0043	1.436E-10	TRUE	7701	0.00532129	41.19848567
glutaroyl carnitine	rs13375749	T	C	0.7801	0.0297	0.0027	2.797E-28	TRUE	7701	0.015469189	121
glutaroyl carnitine	rs8012	A	G	0.4414	0.0374	0.0027	6.36E-45	TRUE	7701	0.024309751	191.8737997
glutaroyl carnitine	rs2863979	A	G	0.7244	-0.0296	0.0026	1.584E-29	TRUE	7701	0.016551645	129.6094675
glutaroyl carnitine	rs2469211	A	T	0.8924	-0.0216	0.0036	1.192E-09	TRUE	7701	0.004652966	36
glutaroyl carnitine	rs246234	C	G	0.2958	-0.0199	0.0026	2.561E-14	TRUE	7701	0.007549552	58.58136095
glutaroyl carnitine	rs17641971	T	C	0.6608	-0.0252	0.0026	3.352E-22	TRUE	7701	0.012051513	93.9408284
glutaroyl carnitine	rs715	T	C	0.7174	0.0215	0.0026	3.496E-16	TRUE	7701	0.00880124	68.38017751
palmitoylcarnitine	rs273914	A	T	0.3691	0.0209	0.0033	4.017E-10	TRUE	7701	0.00518157	40.11111111
palmitoylcarnitine	rs419291	T	C	0.37	0.0202	0.0034	2.381E-09	TRUE	7701	0.004562593	35.29757785
palmitoylcarnitine	rs273913	T	C	0.3836	0.0202	0.0034	2.239E-09	TRUE	7701	0.004562593	35.29757785
palmitoylcarnitine	rs272869	A	G	0.3719	0.02	0.0033	2.071E-09	TRUE	7701	0.004746992	36.73094582
palmitoylcarnitine	rs274567	T	C	0.3725	0.0207	0.0033	5.878E-10	TRUE	7701	0.005083378	39.34710744
palmitoylcarnitine	rs274570	T	C	0.295	0.02	0.0035	7.983E-09	TRUE	7701	0.004222204	32.65306122
palmitoylcarnitine	rs272881	A	G	0.6186	-0.0202	0.0033	1.65E-09	TRUE	7701	0.004841944	37.46923783
x-02269	rs3799344	T	C	0.4494	0.0363	0.0066	3.431E-08	TRUE	7701	0.003912692	30.25
x-02269	rs2199680	A	T	0.8322	0.0526	0.0088	2.18E-09	TRUE	7701	0.004617946	35.72778926
x-02269	rs1165196	A	G	0.549	-0.0409	0.0066	4.795E-10	TRUE	7701	0.004961938	38.40243343
x-02269	rs11754288	A	G	0.436	0.0409	0.0066	4.885E-10	TRUE	7701	0.004961938	38.40243343
x-02269	rs1934955	A	G	0.7115	0.0517	0.0073	1.415E-12	TRUE	7701	0.006470961	50.15744042
x-02269	rs2071426	T	C	0.7107	0.0547	0.0073	6.866E-14	TRUE	7701	0.007238139	56.14730719
x-02269	rs4149081	A	G	0.1699	-0.0513	0.0088	5.066E-09	TRUE	7701	0.004393494	33.98360021
x-02269	rs1871395	A	G	0.8394	0.0512	0.0089	7.685E-09	TRUE	7701	0.00427908	33.09481126
x-02269	rs4149056	T	C	0.8391	0.0493	0.0088	1.996E-08	TRUE	7701	0.004058962	31.38545971
x-02269	rs2762353	A	G	0.4544	0.0409	0.0066	4.965E-10	TRUE	7701	0.004961938	38.40243343
x-02269	rs12317268	A	G	0.8394	0.0512	0.0089	7.977E-09	TRUE	7701	0.00427908	33.09481126
x-02269	rs1165209	A	G	0.5495	-0.0403	0.0066	8.813E-10	TRUE	7701	0.004818119	37.28397612
x-02269	rs7969341	A	G	0.8303	0.0502	0.0088	1.119E-08	TRUE	7701	0.004207883	32.54183884
oleoylcarnitine	rs273914	A	T	0.3678	0.0232	0.0035	3.04E-11	TRUE	7708	0.005667997	43.93795918
oleoylcarnitine	rs419291	T	C	0.3692	0.0215	0.0035	1.001E-09	TRUE	7708	0.004871674	37.73469388
oleoylcarnitine	rs273913	T	C	0.3824	0.0217	0.0035	5.879E-10	TRUE	7708	0.004962279	38.44
oleoylcarnitine	rs272869	A	G	0.3712	0.0221	0.0035	2.287E-10	TRUE	7708	0.005145957	39.87020408
oleoylcarnitine	rs274567	T	C	0.3716	0.0228	0.0035	5.647E-11	TRUE	7708	0.005475294	42.43591837
oleoylcarnitine	rs11950562	A	C	0.5237	0.0189	0.0035	4.977E-08	TRUE	7708	0.003768825	29.16
oleoylcarnitine	rs272881	A	G	0.6196	-0.0224	0.0035	1.351E-10	TRUE	7708	0.005285871	40.96
x-11820	rs445925	A	G	0.079	0.065	0.0082	2.455E-15	TRUE	7710	0.008083875	62.83462225

x-11820	rs439401	T	C	0.351	-0.034	0.0041	1.219E-16	TRUE	7710	0.00884055	68.76859012
n-acetylalanine	rs11144136	T	G	0.1375	0.0097	0.0018	4.862E-08	TRUE	7720	0.003747577	29.04012346
x-08402	rs4902243	A	G	0.8582	-0.0677	0.004	2.934E-63	TRUE	7726	0.03575129	286.455625
x-08402	rs4814176	T	C	0.3938	0.0216	0.0026	2.968E-17	TRUE	7726	0.008854085	69.01775148
x-08402	rs7157785	T	G	0.1649	0.0681	0.0034	7.226E-87	TRUE	7726	0.049362444	401.1773356
2-stearoylglycerophosphocholine*	rs2121073	T	C	0.1209	-0.0293	0.0052	2.148E-08	TRUE	7730	0.00409043	31.74889053
1,5-anhydroglucitol (1,5-ag)	rs6430553	T	C	0.3928	-0.0167	0.0026	2.605E-10	TRUE	7746	0.005297876	41.25591716
1,5-anhydroglucitol (1,5-ag)	rs3800993	T	C	0.1697	0.0287	0.0036	1.528E-15	TRUE	7746	0.008138276	63.55632716
1,5-anhydroglucitol (1,5-ag)	rs7570971	A	C	0.3255	-0.0373	0.0027	7.861E-45	TRUE	7746	0.024045954	190.8491084
1,5-anhydroglucitol (1,5-ag)	rs9756306	A	G	0.3332	-0.0158	0.0027	3.64E-09	TRUE	7746	0.004401426	34.2441701
1,5-anhydroglucitol (1,5-ag)	rs12465802	A	G	0.31	-0.0364	0.0032	3.466E-29	TRUE	7746	0.016429741	129.390625
bilirubin (e,e)*	rs887829	T	C	0.3295	0.128	0.0062	3.76E-95	TRUE	7748	0.052142289	426.2226847
bilirubin (e,e)*	rs4148324	T	G	0.6694	-0.1249	0.0061	7.69E-92	TRUE	7748	0.051332186	419.242408
1-oleoylglycerophosphoethanolamir	rs1077835	A	G	0.7947	-0.026	0.0044	2.612E-09	TRUE	7749	0.004485833	34.91735537
1-oleoylglycerophosphoethanolamir	rs2070895	A	G	0.2092	0.0266	0.0044	1.103E-09	TRUE	7749	0.004694278	36.54752066
erythronate*	rs4687717	T	C	0.4248	0.0133	0.0018	1.069E-13	TRUE	7752	0.006993532	54.59567901
erythronate*	rs10263766	T	C	0.2442	-0.0106	0.0018	7.47E-09	TRUE	7752	0.004453633	34.67901235
erythronate*	rs2391217	A	C	0.1069	0.0149	0.0027	4.882E-08	TRUE	7752	0.003913168	30.45404664
gamma-glutamylphenylalanine	rs2817817	A	G	0.2809	-0.01	0.0018	2.137E-08	TRUE	7753	0.003965151	30.86419753
gamma-glutamylphenylalanine	rs2263065	T	G	0.4417	-0.0105	0.0018	3.563E-09	TRUE	7753	0.004369803	34.02777778
gamma-glutamylvaline	rs1083801	A	G	0.7066	-0.0139	0.0024	8.781E-09	TRUE	7753	0.004307868	33.54340278
x-11381	rs12356193	A	G	0.8359	0.0236	0.0026	8.363E-20	TRUE	7753	0.010515179	82.39053254
x-11381	rs1171614	T	C	0.2181	-0.0238	0.0027	2E-18	TRUE	7753	0.009922606	77.70096022
x-11381	rs1171617	T	G	0.7812	0.0246	0.0027	1.386E-19	TRUE	7753	0.010593698	83.01234568
x-11381	rs1171615	T	C	0.782	0.0238	0.0027	2.036E-18	TRUE	7753	0.009922606	77.70096022
x-11444	rs559555	A	T	0.5581	0.0327	0.0034	4.397E-22	TRUE	7758	0.01178258	92.49913495
x-11444	rs207420	T	C	0.9512	0.0656	0.0081	8.28E-16	TRUE	7758	0.008383619	65.59000152
x-12038	rs3764261	A	C	0.3353	0.0128	0.0019	1.795E-11	TRUE	7758	0.005816071	45.38504155
x-12038	rs7499892	T	C	0.175	-0.017	0.003	1.456E-08	TRUE	7758	0.004122035	32.11111111
x-12038	rs11076175	A	G	0.845	0.017	0.003	1.456E-08	TRUE	7758	0.004122035	32.11111111
x-12038	rs247616	T	C	0.3217	0.0128	0.0019	1.793E-11	TRUE	7758	0.005816071	45.38504155
x-12038	rs1800775	A	C	0.4977	0.0128	0.0019	1.024E-11	TRUE	7758	0.005816071	45.38504155
asparagine	rs3103098	C	G	0.4482	0.0102	0.0018	8.397E-09	TRUE	7761	0.004120448	32.11111111
asparagine	rs2011069	A	G	0.37	0.027	0.002	1.564E-41	TRUE	7761	0.022944009	182.25
asparagine	rs3017098	A	G	0.5517	0.0107	0.0018	1.476E-09	TRUE	7761	0.004532439	35.33641975
1-palmitoylglycerophosphoethanola	rs1532085	A	G	0.3829	0.0179	0.0026	1.157E-11	TRUE	7763	0.006068568	47.39792899
1-palmitoylglycerophosphoethanola	rs10468017	T	C	0.2879	0.0187	0.0027	3.329E-12	TRUE	7763	0.006141165	47.96844993
1-palmitoylglycerophosphoethanola	rs1077835	A	G	0.7961	-0.029	0.0034	9.448E-18	TRUE	7763	0.009284479	72.75086505
1-palmitoylglycerophosphoethanola	rs588136	T	C	0.7967	-0.0286	0.0034	3.29E-17	TRUE	7763	0.009032419	70.75778547
1-palmitoylglycerophosphoethanola	rs2070895	A	G	0.208	0.0297	0.0034	1.36E-18	TRUE	7763	0.009733689	76.30536332
1-palmitoylglycerophosphoethanola	rs633695	A	G	0.7195	-0.0188	0.0033	1.567E-08	TRUE	7763	0.004163383	32.45546373
decanoylcarnitine	rs2062541	A	G	0.6144	0.0225	0.004	2.89E-08	TRUE	7766	0.004057718	31.640625
decanoylcarnitine	rs2070630	C	G	0.2948	-0.053	0.0041	2.07E-37	TRUE	7766	0.021064004	167.1029149
decanoylcarnitine	rs11161521	T	C	0.6983	0.0561	0.0041	4.84E-43	TRUE	7766	0.023540456	187.2224866
decanoylcarnitine	rs4690909	C	G	0.6889	0.0521	0.0041	3.205E-36	TRUE	7766	0.020369145	161.4759072
decanoylcarnitine	rs7552404	A	G	0.6984	0.0563	0.0041	3.323E-43	TRUE	7766	0.023704616	188.5597858
decanoylcarnitine	rs12136754	A	C	0.331	-0.0489	0.0041	4.959E-33	TRUE	7766	0.017987452	142.2492564
decanoylcarnitine	rs8396	T	C	0.6969	0.0533	0.0041	8.692E-38	TRUE	7766	0.021298047	169
oleate (18:1n9)	rs1348656	T	C	0.7976	-0.0172	0.003	1.058E-08	TRUE	7768	0.004213774	32.87111111
x-12844	rs2035647	A	G	0.2052	-0.0292	0.0034	2.182E-17	TRUE	7768	0.009405772	73.75778547
x-12844	rs11666603	T	C	0.2406	0.0234	0.004	6.962E-09	TRUE	7768	0.00438625	34.2225
x-12844	rs17614939	A	G	0.782	0.0204	0.0035	5.672E-09	TRUE	7768	0.004354315	33.9722449
epiandrosterone sulfate	rs2572023	A	G	0.4686	-0.0457	0.0058	2.774E-15	TRUE	7769	0.007927834	62.08353151
epiandrosterone sulfate	rs2525554	A	G	0.486	0.0451	0.0058	6.865E-15	TRUE	7769	0.007722627	60.46403092
epiandrosterone sulfate	rs10278040	A	G	0.0422	-0.2677	0.0146	3.645E-75	TRUE	7769	0.041478933	336.1948302
epiandrosterone sulfate	rs11761528	T	C	0.0744	-0.1943	0.0112	1.857E-67	TRUE	7769	0.037293929	300.9605389
epiandrosterone sulfate	rs11981478	T	C	0.0861	-0.1844	0.01	2.801E-75	TRUE	7769	0.041932691	340.0336
epiandrosterone sulfate	rs9632722	A	G	0.9089	0.1733	0.0099	5.966E-68	TRUE	7769	0.037945585	306.4267932
5-dodecenoate (12:1n7)	rs603424	A	G	0.1825	-0.0308	0.0049	4.274E-10	TRUE	7770	0.005059242	39.51020408
x-11261	rs12356193	A	G	0.8361	0.0385	0.0062	4.741E-10	TRUE	7771	0.00493755	38.56009365
x-11261	rs1171614	T	C	0.2233	-0.0423	0.0056	6.681E-14	TRUE	7771	0.007288711	57.05644133
x-11261	rs1171617	T	G	0.7759	0.0413	0.0056	2.271E-13	TRUE	7771	0.006950532	54.390625
x-11261	rs1171615	T	C	0.7767	0.0423	0.0056	6.777E-14	TRUE	7771	0.007288711	57.05644133
x-11261	rs662138	C	G	0.8307	0.0633	0.0062	9.376E-25	TRUE	7771	0.01323611	104.237513
citrulline	rs10164524	A	G	0.6775	-0.01	0.0017	1.059E-08	TRUE	7773	0.004431844	34.60207612
citrulline	rs682103	T	C	0.7055	-0.0097	0.0018	4.257E-08	TRUE	7773	0.003722119	29.04012346
citrulline	rs1509820	A	G	0.5299	0.0096	0.0017	1.749E-08	TRUE	7773	0.004085807	31.88927336
citrulline	rs1935815	A	T	0.3513	0.0111	0.0017	1.548E-10	TRUE	7773	0.005454864	42.63321799
glucose	rs13431652	T	C	0.7174	0.0099	0.0017	4.337E-09	TRUE	7773	0.004344034	33.91349481
stearidonate (18:4n3)	rs174550	T	C	0.6673	0.0329	0.0041	1.459E-15	TRUE	7775	0.008213755	64.39083879
stearidonate (18:4n3)	rs174535	T	C	0.6675	0.0331	0.0041	1.073E-15	TRUE	7775	0.00831309	65.17608566
stearidonate (18:4n3)	rs174538	A	G	0.3028	-0.0305	0.0042	3.302E-13	TRUE	7775	0.006736976	52.73526077
stearidonate (18:4n3)	rs2727271	A	T	0.8929	0.0398	0.0059	1.108E-11	TRUE	7775	0.005818718	45.50531456
stearidonate (18:4n3)	rs174556	T	C	0.2984	-0.0312	0.0042	9.128E-14	TRUE	7775	0.007047558	55.18367347
stearidonate (18:4n3)	rs174578	A	T	0.338	-0.032	0.0041	9.594E-15	TRUE	7775	0.007773963	60.91612136
stearidonate (18:4n3)	rs1535	A	G	0.6659	0.0329	0.0041	1.504E-15	TRUE	7775	0.008213755	64.39083879
stearidonate (18:4n3)	rs174548	C	G	0.7003	0.0307	0.0042	1.971E-13	TRUE	7775	0.006825014	53.42913832
stearidonate (18:4n3)	rs174601	T	C	0.3398	-0.0336	0.0042	7.93E-16	TRUE	7775	0.008164307	64
4-androsten-3beta,17beta-diol disulf	rs3799344	T	C	0.4485	0.0266	0.0041	7.445E-11	TRUE	7776	0.005383873	42.09161214
4-androsten-3beta,17beta-diol disulf	rs2199680	A	T	0.8316	-0.0463	0.0055	4.159E-17	TRUE	7776	0.009031094	70.86578512
4-androsten-3beta,17beta-diol disulf	rs1165196	A	G	0.5499	-0.0296	0.0041	4.395E-13	TRUE	7776	0.00665822	52.12135634
4-androsten-3beta,17beta-diol disulf	rs11754288	A	G	0.4352	0.0293	0.0041	6.888E-13	TRUE	7776	0.006524816	51.07019631
4-androsten-3beta,17beta-diol disulf	rs4149081	A	G	0.1702	0.0454	0.0055	1.606E-16	TRUE	7776	0.008686426	68.13752066
4-androsten-3beta,17beta-diol disulf	rs1871395	A	G	0.8391	-0.0475	0.0056	1.499E-17	TRUE	7776	0.009167589	71.94674745
4-androsten-3beta,17beta-diol disulf	rs4149056	T	C	0.8386	-0.0485	0.0055	1.332E-18	TRUE	7776	0.009901032	77.76033058
4-androsten-3beta,17beta-diol disulf	rs2762353	A	G	0.4536	0.0299	0.0041	2.505E-13	TRUE	7776	0.006792947	53.18322427
4-androsten-3beta,17beta-diol disulf	rs12317268	A	G	0.8391	-0.0474	0.0056	1.665E-17	TRUE	7776	0.009129381	71.64413265
4-androsten-3beta,17beta-diol disulf	rs1165209	A	G	0.5504	-0.0298	0.0041	2.775E-13	TRUE	7776	0.006747891	52.82807852
4-androsten-3beta,17beta-diol disulf	rs7969341	A	G	0.8299	-0.045	0.0055	3.18E-16	TRUE	7776	0.008535336	66.94214876
4-methyl-2-oxopentanoate	rs1440581	T	C	0.4571	-0.0141	0.0022	2.426E-10	TRUE	7776	0.005254707	41.07644628
4-methyl-2-oxopentanoate	rs893971	A	G	0.5853	-0.0137	0.0023	1.356E-09	TRUE	7776	0.004542052	35.48015123
4-methyl-2-oxopentanoate	rs4941615	T	C	0.2119	0.0133	0.0024	4.537E-08	TRUE	7776	0.003933804	30.71006944
palmitoleate (16:1n7)	rs603424	A	G	0.1849	-0.0367	0.0048	1.313E-14	TRUE	7776	0.007461749	58.45876736
x-08988	rs10938307	T	C	0.4663	-0.0106	0.0019	1.484E-08	TRUE	7776	0.003986699	31.12465374
x-08988	rs10508477	C	G	0.5383	0.0102	0.0019	0.000000048	TRUE	7776	0.003692583	28.8199446
x-08988	rs715	T	C	0.7175	-0.0327	0.0027	7.146E-35	TRUE	7776	0.018513815	146.6790123
x-11550	rs3764261	A	C	0.3318	0.0091	0.0014	1.232E-10	TRUE	7776	0.005404023	42.25
x-11550	rs247616	T	C	0.3242	0.0096	0.0014	1.111E-11	TRUE	7776	0.006010518	47.02040816
adrenate (22:4n6)	rs174550	T	C	0.6676	0.0347	0.0034	6.79E-24	TRUE	7778	0.013214656	104.1600346
adrenate (22:4n6)	rs174535	T	C	0.6678	0.0341	0.0035	5.172E-23	TRUE	7778	0.012056928	94.92326531
adrenate (22:4n6)	rs174538	A	G	0.3025	-0.0328	0.0035	5.604E-21	TRUE	7778	0.011165223	87.82367347
adrenate (22:4n6)	rs2727271	A	T	0.8926	0.0299	0.0051	5.53E-09	TRUE	7778	0.00439966	34.37178008
adrenate (22:4n6)	rs174556	T	C	0.298	-0.0321	0.0035	4.082E-20	TRUE	7778	0.010698788	84.11510204
adrenate (22:4n6)	rs174578	A	T	0.3376	-0.0331	0.0034	8.12E-22	TRUE	7778	0.012038441	94.77595156
adrenate (22:4n6)	rs1535	A	G	0.6662	0.0341	0.0034	5.183E-23	TRUE	7778	0.0127674	100.5891003
adrenate (22:4n6)	rs174548	C	G	0.7007	0.0318	0.0035	6.814E-20	TRUE	7778	0.010501835	82.55020408
adrenate (22:4n6)	rs174601	T	C	0.3395	-0.0339	0.0035	2.53E-22	TRUE	7778	0.011917593	93.81306122
3-methyl-2-oxovalerate	rs1440581	T	C	0.4586	-0.014	0.0017	1.212E-16	TRUE	7779	0.008643	67.8200692
3-methyl-2-oxovalerate	rs893971	A	G	0.5883	-0.0118	0.0017	5.693E-12	TRUE	7779	0.006155465	48.1799308
3-methyl-2-oxovalerate	rs11084396	T	C	0.544	-0.011	0.002	3.798E-08	TRUE	7779	0.003873611	30.25
3-methyl-2-oxovalerate	rs1260326	T	C	0.406	0.01	0.0017	4.572E-09	TRUE	7779	0.004428441	34.60207612
x-11469	rs3799344	T	C	0.451	0.0391	0.0062	2.41E-10	TRUE	7779	0.005086647	39.77133195
x-11469	rs2199680	A	T	0.8293	0.0453	0.0082	3.339E-08	TRUE	7779	0.003907909	30.51888757
x-11469	rs1165196	A	G	0.5465	-0.0433	0.0062	2.017E-12	TRUE	7779	0.006230948	48.77445369
x-11469	rs11754288	A	G	0.4387	0.0434	0.0062	1.878E-12	TRUE	7779	0.006259581	49
x-11469	rs1934955	A	G	0.7084	0.0502	0.0068	2.077E-13	TRUE	7779	0.006957189	54.49913495
x-11469	rs2071426	T	C	0.7065	0.0517	0.0068	3.965E-14	TRUE	7779	0.007376057	57.80471453
x-11469	rs4149081	A	G	0.171	-0.0437	0.0077	1.658E-08	TRUE	7779	0.004123473	32.20931017
x-11469	rs2762353	A	G	0.4581	0.0435	0.0062	1.991E-12	TRUE	7779	0.006288279	49.2260666
x-11469	rs1165209	A	G	0.5468	-0.043	0.0062	3.276E-12	TRUE	7779	0.006145435	48.10093652
x-11469	rs7969341	A	G	0.8291	0.0433	0.0078	2.287E-08	TRUE	7779	0.003945897	30.81673241
nonadecanoate (19:0)	rs603424	A	G	0.1833	0.0182	0.0032	2.229E-08	TRUE	7783	0.004138991	32.34765625
x-10395	rs4073054	A	C	0.6255	0.0163	0.0018	4.417E-19	TRUE	7784	0.010425001	82.00308642
x-10395	rs1800775	A	C	0.4986	0.0108	0.0019	7.958E-09	TRUE	7784	0.004133696	32.31024931
androsterone sulfate	rs2572023	A	G	0.4687	-0.0576	0.0064	4.178E-19	TRUE	7785	0.010297483	81
androsterone sulfate	rs182420	T	C	0.7553	0.051	0.0074	6.237E-12	TRUE	7785	0.006064243	47.49817385
androsterone sulfate	rs2547231	A	C	0.8292	0.0552	0.0083	2.773E-11	TRUE	7785	0.005649407	44.23051241
androsterone sulfate	rs2525554	A	G	0.4863	0.056	0.0064	4.108E-18	TRUE	7785	0.00973884	76.5625
androsterone sulfate	rs296396	T	C	0.1647	-0.0569	0.0088	8.606E-11	TRUE	7785	0.005341639	41.80798037

androsterone sulfate	rs10278040	A	G	0.0419	-0.3609	0.016	8.82E-113	TRUE	7785	0.061345266	508.7844141
androsterone sulfate	rs11761528	T	C	0.0737	-0.2691	0.012	5.47E-111	TRUE	7785	0.060676625	502.880625
androsterone sulfate	rs11981478	T	C	0.0858	-0.2456	0.0114	1.23E-103	TRUE	7785	0.056265018	464.1378886
androsterone sulfate	rs9632722	A	G	0.9091	0.2373	0.0107	9.45E-109	TRUE	7785	0.059424169	491.8446153
androsterone sulfate	rs296381	T	C	0.8285	0.0572	0.0088	6.616E-11	TRUE	7785	0.005397809	42.25
x-11315	rs1809148	A	G	0.136	-0.0262	0.0043	9.514E-10	TRUE	7785	0.004746144	37.1249324
x-11315	rs17279437	A	G	0.0954	-0.0347	0.0052	2.079E-11	TRUE	7785	0.005687437	44.52995562
x-11315	rs3761097	T	C	0.053	0.057	0.008	1.041E-12	TRUE	7785	0.006478706	50.765625
x-11315	rs4327428	A	C	0.1117	-0.031	0.0044	2.814E-12	TRUE	7785	0.006335765	49.63842975
c-glycosyltryptophan*	rs6867478	A	G	0.0139	0.0695	0.0125	2.969E-08	TRUE	7786	0.003954707	30.9136
hexanoylcarnitine	rs7727544	T	C	0.5545	-0.02	0.0033	1.579E-09	TRUE	7786	0.004695412	36.73094582
hexanoylcarnitine	rs273914	A	T	0.3671	0.0211	0.0033	1.877E-10	TRUE	7786	0.005223339	40.88246097
hexanoylcarnitine	rs419291	T	C	0.3687	0.0225	0.0033	1.673E-11	TRUE	7786	0.005935228	46.48760331
hexanoylcarnitine	rs2070630	C	G	0.2948	-0.021	0.0034	6.757E-10	TRUE	7786	0.004875775	38.14878893
hexanoylcarnitine	rs1171614	T	C	0.2222	-0.0246	0.0037	4.048E-11	TRUE	7786	0.005645386	44.20452885
hexanoylcarnitine	rs11161521	T	C	0.6983	0.0703	0.0033	6.42E-100	TRUE	7786	0.055076343	453.8191001
hexanoylcarnitine	rs4690909	C	G	0.6886	0.0198	0.0034	5.68E-09	TRUE	7786	0.004336812	33.91349481
hexanoylcarnitine	rs1171617	T	G	0.777	0.0246	0.0037	4.075E-11	TRUE	7786	0.005645386	44.20452885
hexanoylcarnitine	rs7552404	A	G	0.6985	0.07	0.0033	1.11E-98	TRUE	7786	0.054632904	449.9540863
hexanoylcarnitine	rs273913	T	C	0.3818	0.0209	0.0033	3.752E-10	TRUE	7786	0.005125293	40.11111111
hexanoylcarnitine	rs272869	A	G	0.3706	0.0224	0.0033	1.395E-11	TRUE	7786	0.005882898	46.07529844
hexanoylcarnitine	rs12136754	A	C	0.3312	-0.063	0.0033	2.585E-80	TRUE	7786	0.044716824	364.4628099
hexanoylcarnitine	rs8396	T	C	0.6964	0.0205	0.0034	1.506E-09	TRUE	7786	0.004647425	36.35380623
hexanoylcarnitine	rs274567	T	C	0.371	0.0217	0.0033	5.856E-11	TRUE	7786	0.005522961	43.2405877
hexanoylcarnitine	rs274570	T	C	0.2946	0.0206	0.0034	1.742E-09	TRUE	7786	0.004692663	36.70934256
hexanoylcarnitine	rs11950562	A	C	0.5233	0.0196	0.0033	2.863E-09	TRUE	7786	0.004510312	35.27640037
hexanoylcarnitine	rs1171615	T	C	0.7778	0.0246	0.0037	4.046E-11	TRUE	7786	0.005645386	44.20452885
hexanoylcarnitine	rs272881	A	G	0.6201	-0.0211	0.0033	2.295E-10	TRUE	7786	0.005223339	40.88246097
pro-hydroxy-pro	rs9594738	T	C	0.4782	0.0137	0.0025	4.525E-08	TRUE	7787	0.003841663	30.0304
alanine	rs11183620	A	G	0.4677	0.0098	0.0017	1.548E-08	TRUE	7788	0.004248926	33.23183391
alanine	rs1260326	T	C	0.4051	0.0131	0.0017	5.562E-14	TRUE	7788	0.007566935	59.38062284
10-nonadecenoate (19:1n9)	rs1348656	T	C	0.798	-0.0232	0.0042	3.459E-08	TRUE	7789	0.003902094	30.51247166
acetylphosphate	rs4970834	T	C	0.1821	-0.0098	0.0017	1.06E-08	TRUE	7789	0.004248383	33.23183391
acetylphosphate	rs2799635	T	C	0.3644	0.0089	0.0016	1.96E-08	TRUE	7789	0.003956731	30.94140625
isovalerylcarnitine	rs7727544	T	C	0.5544	-0.0328	0.0033	2.486E-23	TRUE	7789	0.012524615	98.79155188
isovalerylcarnitine	rs273914	A	T	0.3693	0.0389	0.0033	3.594E-32	TRUE	7789	0.017527105	138.9540863
isovalerylcarnitine	rs419291	T	C	0.3699	0.0389	0.0033	1.549E-31	TRUE	7789	0.017527105	138.9540863
isovalerylcarnitine	rs1981524	T	C	0.1991	0.0267	0.0035	4.718E-14	TRUE	7789	0.007416039	58.19510204
isovalerylcarnitine	rs2291449	A	G	0.9135	-0.0288	0.0052	2.354E-08	TRUE	7789	0.003922741	30.67455621
isovalerylcarnitine	rs9635324	A	G	0.6075	0.0337	0.0027	1.886E-35	TRUE	7789	0.019608754	155.78738
isovalerylcarnitine	rs273913	T	C	0.3838	0.0381	0.0033	1.596E-30	TRUE	7789	0.016825614	133.2975207
isovalerylcarnitine	rs272869	A	G	0.3719	0.0386	0.0033	7.645E-32	TRUE	7789	0.017262456	136.8191001
isovalerylcarnitine	rs274567	T	C	0.3726	0.0381	0.0033	4.64E-31	TRUE	7789	0.016825614	133.2975207
isovalerylcarnitine	rs274570	T	C	0.2948	0.0377	0.0034	4.122E-28	TRUE	7789	0.015539656	122.9489619
isovalerylcarnitine	rs11950562	A	C	0.5231	0.0362	0.0027	2.309E-41	TRUE	7789	0.022557914	179.7585734
isovalerylcarnitine	rs272881	A	G	0.6187	-0.0379	0.0033	1.112E-30	TRUE	7789	0.016652365	131.9017447
octanoylcarnitine	rs924135	A	T	0.384	-0.0209	0.0034	1.085E-09	TRUE	7790	0.004827205	37.78633218
octanoylcarnitine	rs2062541	A	G	0.6145	0.0208	0.0034	1.171E-09	TRUE	7790	0.004781343	37.42560554
octanoylcarnitine	rs2070630	C	G	0.2948	-0.0473	0.0041	6.369E-31	TRUE	7790	0.016798087	133.0928019
octanoylcarnitine	rs11161521	T	C	0.6982	0.0743	0.004	4.181E-77	TRUE	7790	0.042412947	345.030625
octanoylcarnitine	rs4690909	C	G	0.6892	0.0468	0.0041	2.083E-30	TRUE	7790	0.016450636	130.2938727
octanoylcarnitine	rs7552404	A	G	0.6984	0.0746	0.004	1.269E-77	TRUE	7790	0.04274147	347.8225
octanoylcarnitine	rs12136754	A	C	0.3308	-0.0641	0.004	1.179E-57	TRUE	7790	0.031913382	256.800625
octanoylcarnitine	rs8396	T	C	0.6972	0.0478	0.0041	1.377E-31	TRUE	7790	0.017148981	135.9214753
pipecolate	rs12725284	T	C	0.1606	0.0315	0.0056	1.72E-08	TRUE	7792	0.004044233	31.640625
x-10510	rs3762660	T	G	0.8935	0.0199	0.0036	2.68E-08	TRUE	7792	0.003906182	30.55632716
x-10510	rs4902243	A	G	0.8624	-0.0442	0.0035	3.408E-37	TRUE	7792	0.020056744	159.4808163
x-10510	rs4814176	T	C	0.394	0.0157	0.0026	1.043E-09	TRUE	7792	0.004657749	36.46301775
x-10510	rs7157785	T	G	0.1646	0.0442	0.0035	3.543E-37	TRUE	7792	0.020056744	159.4808163
dehydroisoandrosterone sulfate (dhe	rs182420	T	C	0.7552	0.037	0.0057	1.224E-10	TRUE	7793	0.005377832	42.13604186
dehydroisoandrosterone sulfate (dhe	rs2547231	A	C	0.829	0.0406	0.0065	5.271E-10	TRUE	7793	0.004981405	39.01443787
dehydroisoandrosterone sulfate (dhe	rs296396	T	C	0.1646	-0.0462	0.0065	1.166E-12	TRUE	7793	0.006440896	50.51928994
dehydroisoandrosterone sulfate (dhe	rs10278040	A	G	0.042	-0.0823	0.0122	1.599E-11	TRUE	7793	0.005805594	45.50718893
dehydroisoandrosterone sulfate (dhe	rs11761528	T	C	0.0736	-0.0675	0.009	6.701E-14	TRUE	7793	0.00716629	56.25
dehydroisoandrosterone sulfate (dhe	rs11981478	T	C	0.086	-0.0609	0.0083	2.835E-13	TRUE	7793	0.006860943	53.83669618
dehydroisoandrosterone sulfate (dhe	rs9632722	A	G	0.909	0.0583	0.0083	1.773E-12	TRUE	7793	0.006291227	49.33793003
dehydroisoandrosterone sulfate (dhe	rs296381	T	C	0.8283	0.0437	0.0065	1.674E-11	TRUE	7793	0.0057666	45.19976331
laurate (12:0)	rs6441206	A	G	0.4638	0.0099	0.0018	2.088E-08	TRUE	7793	0.003866679	30.25
laurate (12:0)	rs9563502	A	G	0.7115	-0.01	0.0018	2.56E-08	TRUE	7793	0.003944879	30.86419753
mannose	rs1395	A	G	0.7024	-0.017	0.0025	5.974E-12	TRUE	7793	0.005898531	46.24
mannose	rs1260326	T	C	0.4088	-0.0439	0.0024	1.296E-77	TRUE	7793	0.041166603	334.5850694
3-(4-hydroxyphenyl)lactate	rs7503429	A	C	0.4256	-0.0171	0.0026	5.079E-11	TRUE	7795	0.005518564	43.25591716
3-(4-hydroxyphenyl)lactate	rs4625783	T	C	0.4268	-0.0187	0.0026	3.902E-13	TRUE	7795	0.006592465	51.72928994
methionine	rs320485	T	C	0.1324	0.0094	0.0017	4.97E-08	TRUE	7795	0.003906984	30.57439446
x-12644	rs1532085	A	G	0.3838	0.0157	0.0027	5.102E-09	TRUE	7795	0.004318927	33.81207133
x-12644	rs1077835	A	G	0.7976	-0.0208	0.0035	1.824E-09	TRUE	7795	0.00451036	35.31755102
x-12644	rs4149081	A	G	0.1675	0.0207	0.0035	3.098E-09	TRUE	7795	0.004467289	34.97877551
x-12644	rs588136	T	C	0.796	-0.0228	0.0041	1.979E-08	TRUE	7795	0.00395154	30.92444973
x-12644	rs4149056	T	C	0.8397	-0.0246	0.0041	1.921E-09	TRUE	7795	0.004597114	36
x-12644	rs2070895	A	G	0.2097	0.0235	0.004	5.983E-09	TRUE	7795	0.004408399	34.515625
x-12644	rs7969341	A	G	0.833	-0.0213	0.0035	1.011E-09	TRUE	7795	0.004728773	37.03591837
butyrylcarnitine	rs12356193	A	G	0.8358	0.0358	0.0052	5.064E-12	TRUE	7796	0.006043035	47.39792899
butyrylcarnitine	rs4556628	T	C	0.2953	0.0679	0.0042	6.981E-58	TRUE	7796	0.032437557	261.3611111
butyrylcarnitine	rs273914	A	T	0.3673	0.0236	0.0041	1.168E-08	TRUE	7796	0.004231971	33.13265913
butyrylcarnitine	rs1171614	T	C	0.2221	-0.0349	0.0047	6.785E-14	TRUE	7796	0.007022997	55.13852422
butyrylcarnitine	rs2291449	A	G	0.914	-0.0373	0.0068	3.49E-08	TRUE	7796	0.003844635	30.08845156
butyrylcarnitine	rs1171617	T	G	0.7771	0.0358	0.0047	1.611E-14	TRUE	7796	0.007387175	58.01901313
butyrylcarnitine	rs273913	T	C	0.382	0.0229	0.0042	3.873E-08	TRUE	7796	0.00379881	29.72845805
butyrylcarnitine	rs272869	A	G	0.3709	0.023	0.0041	2.836E-08	TRUE	7796	0.004020375	31.46936347
butyrylcarnitine	rs2469211	A	T	0.8921	-0.0342	0.006	1.058E-08	TRUE	7796	0.004150226	32.49
butyrylcarnitine	rs274567	T	C	0.3712	0.024	0.0041	6.227E-09	TRUE	7796	0.00437601	34.26531826
butyrylcarnitine	rs1171615	T	C	0.7779	0.0349	0.0047	6.764E-14	TRUE	7796	0.007022997	55.13852422
butyrylcarnitine	rs272881	A	G	0.6199	-0.0228	0.0041	3.829E-08	TRUE	7796	0.003951035	30.92444973
butyrylcarnitine	rs11065202	T	C	0.5822	-0.1297	0.004	1E-200	TRUE	7796	0.118835243	1051.380625
margarate (17:0)	rs603424	A	G	0.1865	0.0292	0.0038	1.56E-14	TRUE	7796	0.007517089	59.04709141
serine	rs4947534	T	C	0.2479	-0.0183	0.0024	1.96E-14	TRUE	7796	0.007402544	58.140625
serine	rs1163251	T	C	0.6	0.0188	0.0017	7.05E-27	TRUE	7796	0.015444933	122.2975779
serine	rs715	T	C	0.7111	-0.0223	0.0024	2.687E-21	TRUE	7796	0.010952981	86.33506944
1-arachidonoylglycerophosphoinosi	rs174550	T	C	0.6643	0.0258	0.0026	5.937E-23	TRUE	7797	0.01247139	98.46745562
1-arachidonoylglycerophosphoinosi	rs2199680	A	T	0.8342	-0.0382	0.0034	1.32E-29	TRUE	7797	0.015931862	126.2318339
1-arachidonoylglycerophosphoinosi	rs174535	T	C	0.6641	0.0257	0.0026	1.041E-22	TRUE	7797	0.012376094	97.7056213
1-arachidonoylglycerophosphoinosi	rs174538	A	G	0.3062	-0.0249	0.0026	4.586E-21	TRUE	7797	0.011626409	91.71745562
1-arachidonoylglycerophosphoinosi	rs2727271	A	T	0.8933	0.0294	0.0042	3.153E-12	TRUE	7797	0.00624522	49
1-arachidonoylglycerophosphoinosi	rs9326061	C	G	0.8497	-0.0188	0.0034	4.393E-08	TRUE	7797	0.003905986	30.57439446
1-arachidonoylglycerophosphoinosi	rs174556	T	C	0.3012	-0.0263	0.0026	2.628E-23	TRUE	7797	0.012953139	102.3210059
1-arachidonoylglycerophosphoinosi	rs4149081	A	G	0.1685	0.0377	0.0034	6.253E-29	TRUE	7797	0.015523959	122.9489619
1-arachidonoylglycerophosphoinosi	rs174578	A	T	0.3409	-0.0266	0.0026	3.019E-24	TRUE	7797	0.013246397	104.6686391
1-arachidonoylglycerophosphoinosi	rs1535	A	G	0.6627	0.0259	0.0026	5.68E-23	TRUE	7797	0.012567038	99.23224852
1-arachidonoylglycerophosphoinosi	rs1871395	A	G	0.841	-0.0395	0.0034	3.66E-31	TRUE	7797	0.017015915	134.9697232
1-arachidonoylglycerophosphoinosi	rs174548	C	G	0.6976	0.0262	0.0026	3.193E-23	TRUE	7797	0.012856088	101.5443787
1-arachidonoylglycerophosphoinosi	rs4149056	T	C	0.8405	-0.0392	0.0034	4.247E-31	TRUE	7797	0.016762743	132.9273356
1-arachidonoylglycerophosphoinosi	rs174601	T	C	0.3386	-0.0252	0.0032	7.892E-15	TRUE	7797	0.007891017	62.015625
1-arachidonoylglycerophosphoinosi	rs12317268	A	G	0.841	-0.0395	0.0034	3.975E-31	TRUE	7797	0.017015915	134.9697232
1-arachidonoylglycerophosphoinosi	rs7969341	A	G	0.8319	-0.0369	0.0034	9.116E-28	TRUE	7797	0.014881808	117.7863322
1-arachidonoylglycerophosphoinosi	rs8736	T	C	0.4379	-0.0227	0.0026	8.449E-18	TRUE	7797	0.009681715	76.22633136
2-hydroxypalmitate	rs9404466	T	C	0.2671	0.0105	0.0018	1.264E-08	TRUE	7797	0.004345251	34.02777778
carnitine	rs11620955	A	G	0.4767	-0.0051	0.0009	1.632E-08	TRUE	7797	0.004101502	32.11111111
carnitine	rs12356193	A	G	0.8362	0.0274	0.0016	3.686E-63	TRUE	7797	0.036249196	293.265625
carnitine	rs7727544	T	C	0.555	-0.0068	0.0009	4.546E-14	TRUE	7797	0.007268372	57.08641975
carnitine	rs4860022	T	C	0.3362	-0.0055	0.0009	9.602E-10	TRUE	7797	0.004766917	37.34567901
carnitine	rs6862024	A	G	0.3691	-0.0055	0.0009	8.988E-10	TRUE	7797	0.004766917	37.34567901
carnitine	rs419291	T	C	0.3667	0.0079	0.0009	3.096E-18	TRUE	7797	0.00978523	77.04938272
carnitine	rs735315	T	C	0.4943	0.0054	0.0009	1.868E-09	TRUE	7797	0.00459594	36
carnitine	rs11183620	A	G	0.4676	0.005	0.0009	3.003E-08	TRUE	7797	0.003942863	30.86419753
carnitine	rs2492788	T	C	0.5457	0.0049	0.0009	4.58E-08	TRUE	7797	0.003787317	29.64197531
carnitine	rs1171614	T	C	0.2128	-0.0291	0.0017	1.799E-62	TRUE	7797	0.036219201	293.0138408
carnitine	rs1171617	T	G	0.7866	0.0291	0.0017	1.663E-62	TRUE	7797	0.036219201	293.0138408
carnitine	rs273913	T	C	0.3781	0.0071	0.0009	3.792E-15	TRUE	7797	0.007918655	62.2345679
carnitine	rs272869	A	G	0.3687	0.007	0.0009	5.505E-15	TRUE	7797	0.00769887	60.49382716

carnitine	rs2405522	A	G	0.1697	-0.0113	0.0016	2.318E-12	TRUE	7797	0.006356528	49.87890625
carnitine	rs1466788	A	G	0.4068	-0.0074	0.0009	3.053E-16	TRUE	7797	0.008596101	67.60493827
carnitine	rs3736438	T	G	0.3889	-0.0057	0.0009	3.186E-10	TRUE	7797	0.005118099	40.11111111
carnitine	rs2279014	T	C	0.367	-0.005	0.0009	4.863E-08	TRUE	7797	0.003942863	30.86419753
carnitine	rs2114713	T	G	0.58	-0.005	0.0009	2.665E-08	TRUE	7797	0.003942863	30.86419753
carnitine	rs2396004	A	G	0.4389	0.0049	0.0009	4.315E-08	TRUE	7797	0.003787317	29.64197531
carnitine	rs274567	T	C	0.3685	0.0069	0.0009	1.809E-14	TRUE	7797	0.007482108	58.77777778
carnitine	rs1171615	T	C	0.7872	0.0291	0.0017	1.734E-62	TRUE	7797	0.036219201	293.0138408
carnitine	rs13182512	A	T	0.3806	-0.005	0.0009	3.363E-08	TRUE	7797	0.003942863	30.86419753
carnitine	rs12709393	T	G	0.6286	0.0053	0.0009	6.408E-09	TRUE	7797	0.004428043	34.67901235
carnitine	rs272881	A	G	0.6226	-0.007	0.0009	6.573E-15	TRUE	7797	0.00769887	60.49382716
carnitine	rs10821585	A	G	0.4124	-0.0086	0.0009	1.277E-21	TRUE	7797	0.011575186	91.30864198
carnitine	rs11620973	A	G	0.6326	-0.0051	0.0009	2.346E-08	TRUE	7797	0.004101502	32.11111111
carnitine	rs9842133	T	C	0.6649	0.0064	0.0009	4.2E-12	TRUE	7797	0.006443767	50.56790123
1-arachidonoylglycerophosphoethar	rs174550	T	C	0.6642	0.0349	0.0026	1.135E-40	TRUE	7798	0.022583975	180.1789941
1-arachidonoylglycerophosphoethar	rs2199680	A	T	0.834	-0.0286	0.0034	2.175E-17	TRUE	7798	0.008992243	70.75778547
1-arachidonoylglycerophosphoethar	rs174535	T	C	0.6641	0.0341	0.0026	9.865E-39	TRUE	7798	0.021582563	172.0133136
1-arachidonoylglycerophosphoethar	rs10468017	T	C	0.2875	0.0171	0.0027	1.287E-10	TRUE	7798	0.005117446	40.11111111
1-arachidonoylglycerophosphoethar	rs174538	A	G	0.3062	-0.0325	0.0026	7.71E-35	TRUE	7798	0.019643587	156.25
1-arachidonoylglycerophosphoethar	rs968567	T	C	0.1801	-0.0252	0.0035	5.515E-13	TRUE	7798	0.006603956	51.84
1-arachidonoylglycerophosphoethar	rs2727271	A	T	0.893	0.0332	0.0042	2.686E-15	TRUE	7798	0.007949288	62.48526077
1-arachidonoylglycerophosphoethar	rs174556	T	C	0.3012	-0.0337	0.0026	1.916E-37	TRUE	7798	0.021089813	168.0014793
1-arachidonoylglycerophosphoethar	rs4149081	A	G	0.1686	0.0272	0.0034	7.284E-16	TRUE	7798	0.008140422	64
1-arachidonoylglycerophosphoethar	rs174578	A	T	0.341	-0.0354	0.0026	6.122E-42	TRUE	7798	0.023220582	185.3786982
1-arachidonoylglycerophosphoethar	rs1535	A	G	0.6627	0.0342	0.0026	4.819E-39	TRUE	7798	0.021706581	173.0236686
1-arachidonoylglycerophosphoethar	rs1871395	A	G	0.8409	-0.0279	0.0034	2.563E-16	TRUE	7798	0.008561173	67.33650519
1-arachidonoylglycerophosphoethar	rs174548	C	G	0.6975	0.0337	0.0026	1.296E-37	TRUE	7798	0.021089813	168.0014793
1-arachidonoylglycerophosphoethar	rs4149056	T	C	0.8405	-0.0294	0.0034	2.664E-18	TRUE	7798	0.009497497	74.7716263
1-arachidonoylglycerophosphoethar	rs174601	T	C	0.3388	-0.0364	0.0032	2.132E-29	TRUE	7798	0.016321969	129.390625
1-arachidonoylglycerophosphoethar	rs12317268	A	G	0.8409	-0.0285	0.0034	4.938E-17	TRUE	7798	0.008930031	70.26384083
1-arachidonoylglycerophosphoethar	rs174450	T	G	0.5391	0.0181	0.0026	3.487E-12	TRUE	7798	0.006176416	48.46301775
1-arachidonoylglycerophosphoethar	rs7969341	A	G	0.8317	-0.0277	0.0034	2E-16	TRUE	7798	0.008439904	66.37456747
leucine	rs7647029	A	G	0.6479	0.0053	0.0009	1.021E-08	TRUE	7799	0.004426913	34.67901235
leucine	rs1871053	A	G	0.4513	-0.005	0.0009	2.401E-08	TRUE	7799	0.003941856	30.86419753
leucine	rs10923016	A	G	0.6191	0.0053	0.0009	2.88E-09	TRUE	7799	0.004426913	34.67901235
leucine	rs7740614	A	G	0.4838	-0.0049	0.0009	4.746E-08	TRUE	7799	0.00378635	29.64197531
leucine	rs7584842	T	C	0.5379	-0.0051	0.0009	1.892E-08	TRUE	7799	0.004100454	32.11111111
leucine	rs6737109	T	C	0.5761	0.0051	0.0009	1.454E-08	TRUE	7799	0.004100454	32.11111111
leucine	rs1440581	T	C	0.4592	-0.0081	0.0009	1.441E-19	TRUE	7799	0.010279188	81
leucine	rs6735596	A	C	0.6267	-0.0052	0.0009	1.153E-08	TRUE	7799	0.004262141	33.38271605
leucine	rs2713737	A	G	0.44	-0.006	0.001	1.973E-09	TRUE	7799	0.004594767	36
leucine	rs6820313	T	G	0.5169	0.0049	0.0009	4.651E-08	TRUE	7799	0.00378635	29.64197531
leucine	rs6820011	T	G	0.5152	-0.0051	0.0009	1.182E-08	TRUE	7799	0.004100454	32.11111111
leucine	rs9541214	T	C	0.6529	-0.005	0.0009	0.000000034	TRUE	7799	0.003941856	30.86419753
leucine	rs893971	A	G	0.5895	-0.0067	0.0009	8.296E-14	TRUE	7799	0.007055869	55.41975309
x-11204	rs2935461	A	G	0.81	-0.011	0.002	3.798E-08	TRUE	7799	0.003863716	30.25
x-11204	rs10468017	T	C	0.289	0.0108	0.0018	3.454E-09	TRUE	7799	0.004594767	36
x-11204	rs588136	T	C	0.7969	-0.0142	0.0026	2.409E-08	TRUE	7799	0.003810072	29.82840237
palmitate (16:0)	rs603424	A	G	0.1836	0.0151	0.0024	2.455E-10	TRUE	7800	0.005049383	39.58506944
pyroglutamine*	rs2080403	C	G	0.4672	-0.0375	0.0036	4.08E-25	TRUE	7800	0.013720282	108.5069444
pyroglutamine*	rs1600760	A	T	0.663	-0.0216	0.0037	5.121E-09	TRUE	7800	0.004350268	34.08035062
pyroglutamine*	rs17279437	A	G	0.0953	0.059	0.0063	1.251E-20	TRUE	7800	0.011119168	87.70471151
pyroglutamine*	rs11613331	A	G	0.5532	0.0374	0.0036	2.228E-25	TRUE	7800	0.013648202	107.9290123
pyroglutamine*	rs715	T	C	0.7139	0.0361	0.0044	2.455E-16	TRUE	7800	0.008556232	67.31456612
pyroglutamine*	rs4327428	A	C	0.1116	0.0501	0.0056	2.276E-19	TRUE	7800	0.010157131	80.03858418
uridine	rs131794	A	C	0.2074	0.0146	0.0018	4.367E-16	TRUE	7800	0.008364083	65.79012346
uridine	rs532545	T	C	0.3065	-0.0095	0.0017	3.41E-08	TRUE	7800	0.003987672	31.2283737
uridine	rs2686796	T	C	0.4524	0.0094	0.0017	1.889E-08	TRUE	7800	0.003904489	30.57439446
5-oxoproline	rs12550729	T	C	0.9233	0.0637	0.0029	5.97E-110	TRUE	7802	0.058239469	482.4839477
5-oxoproline	rs7822232	A	C	0.9201	0.0634	0.0029	4.56E-106	TRUE	7802	0.057723785	477.9500595
caprylate (8:0)	rs8059670	T	C	0.4437	-0.0097	0.0018	3.717E-08	TRUE	7802	0.003708335	29.04012346
caprylate (8:0)	rs10514491	T	C	0.6751	0.0103	0.0018	1.585E-08	TRUE	7802	0.004179311	32.74382716
glycine	rs10164524	A	G	0.6787	0.0299	0.0025	1.066E-33	TRUE	7802	0.018003883	143.0416
glycine	rs715	T	C	0.708	-0.076	0.0029	1.58E-147	TRUE	7802	0.080906761	686.8014269
heptanoate (7:0)	rs10153405	A	G	0.5342	-0.0103	0.0018	9.267E-09	TRUE	7802	0.004179311	32.74382716
heptanoate (7:0)	rs864428	T	C	0.4058	0.0101	0.0018	2.986E-08	TRUE	7802	0.004019229	31.4845679
indolepropionate	rs11647589	A	G	0.7122	0.0342	0.0038	4.334E-19	TRUE	7803	0.010273973	81
indolepropionate	rs6497490	T	G	0.8821	-0.0589	0.0068	6.263E-18	TRUE	7803	0.009523473	75.02616782
indolepropionate	rs1394678	T	C	0.2785	-0.0354	0.0038	1.696E-20	TRUE	7803	0.010999532	86.78393352
myo-inositol	rs4787294	A	T	0.9253	0.0271	0.0038	1.416E-12	TRUE	7803	0.006475723	50.85941828
myo-inositol	rs4808136	A	G	0.3392	0.0132	0.0018	5.417E-14	TRUE	7803	0.006844762	53.77777778
myo-inositol	rs4788439	T	C	0.0772	-0.0271	0.0038	6.593E-13	TRUE	7803	0.006475723	50.85941828
pelargonate (9:0)	rs1938479	T	C	0.6445	-0.01	0.0018	1.784E-08	TRUE	7803	0.003939843	30.86419753
phenylalanine	rs1498694	A	G	0.4035	0.0075	0.0011	7.336E-11	TRUE	7803	0.005922374	46.48760331
stearate (18:0)	rs1348656	T	C	0.798	-0.0137	0.0024	1.169E-08	TRUE	7803	0.004158601	32.58506944
stearate (18:0)	rs603424	A	G	0.1835	0.0225	0.0024	6.064E-21	TRUE	7803	0.011138239	87.890625
4-androsten-3beta,17beta-diol disulf	rs182420	T	C	0.7554	0.1328	0.0073	1.833E-73	TRUE	7804	0.040681413	330.9408895
4-androsten-3beta,17beta-diol disulf	rs2547231	A	C	0.829	0.166	0.0087	2.839E-81	TRUE	7804	0.044571633	364.063945
4-androsten-3beta,17beta-diol disulf	rs296396	T	C	0.1651	-0.1754	0.0086	1.48E-92	TRUE	7804	0.050604837	415.9702542
4-androsten-3beta,17beta-diol disulf	rs11761528	T	C	0.074	-0.0696	0.012	6.269E-09	TRUE	7804	0.004292108	33.64
4-androsten-3beta,17beta-diol disulf	rs11981478	T	C	0.0861	-0.0618	0.0113	4.81E-08	TRUE	7804	0.003818049	29.91025139
4-androsten-3beta,17beta-diol disulf	rs296381	T	C	0.8284	0.1747	0.0086	6.05E-92	TRUE	7804	0.050221972	412.6567063
histidine	rs16868246	C	G	0.2113	0.0097	0.0013	1.778E-14	TRUE	7804	0.00708357	55.67455621
histidine	rs938554	C	G	0.2256	0.0096	0.0013	2.983E-14	TRUE	7804	0.006939278	54.53254438
myristoleate (14:1n5)	rs603424	A	G	0.1851	-0.0368	0.0046	7.16E-16	TRUE	7804	0.008134215	64
tryptophan	rs6480970	A	G	0.6159	-0.0049	0.0009	4.287E-08	TRUE	7804	0.003783933	29.64197531
tryptophan	rs284191	A	G	0.615	-0.006	0.001	1.973E-09	TRUE	7804	0.004591837	36
tryptophan	rs38271	A	G	0.5925	-0.0051	0.0009	1.185E-08	TRUE	7804	0.004097838	32.11111111
tryptophan	rs6901004	C	G	0.5742	-0.0061	0.0009	1.078E-11	TRUE	7804	0.005852055	45.9382716
tryptophan	rs972459	T	C	0.5787	0.005	0.0009	1.969E-08	TRUE	7804	0.00393934	30.86419753
tryptophan	rs7584842	T	C	0.5377	-0.005	0.0009	4.15E-08	TRUE	7804	0.00393934	30.86419753
tryptophan	rs4306882	T	G	0.6153	-0.0057	0.0009	2.52E-10	TRUE	7804	0.005113532	40.11111111
tryptophan	rs9511152	A	G	0.559	-0.005	0.0009	2.985E-08	TRUE	7804	0.00393934	30.86419753
tryptophan	rs1373962	T	C	0.5973	0.005	0.0009	2.707E-08	TRUE	7804	0.00393934	30.86419753
tryptophan	rs710580	A	C	0.3566	-0.005	0.0009	3.574E-08	TRUE	7804	0.00393934	30.86419753
tryptophan	rs2111118	T	C	0.6207	0.0051	0.0009	1.212E-08	TRUE	7804	0.004097838	32.11111111
tryptophan	rs13122250	T	C	0.5542	0.0062	0.0009	8.949E-12	TRUE	7804	0.006044329	47.45679012
tryptophan	rs4615256	A	G	0.5327	0.0049	0.0009	4.988E-08	TRUE	7804	0.003783933	29.64197531
tryptophan	rs6935961	A	G	0.5578	-0.0056	0.0009	3.753E-10	TRUE	7804	0.004936561	38.71604938
tryptophan	rs7463805	T	C	0.5213	-0.0053	0.0009	4.596E-09	TRUE	7804	0.004424089	34.67901235
tryptophan	rs1016522	A	G	0.5803	0.0058	0.0009	1.589E-10	TRUE	7804	0.00529357	41.5308642
tryptophan	rs4695138	A	T	0.5352	0.0052	0.0009	7.996E-09	TRUE	7804	0.004259421	33.38271605
tryptophan	rs1559063	C	G	0.6209	0.0052	0.0009	7.818E-09	TRUE	7804	0.004259421	33.38271605
tryptophan	rs4958379	A	G	0.433	-0.005	0.0009	2.176E-08	TRUE	7804	0.00393934	30.86419753
tryptophan	rs603446	T	C	0.4448	0.0051	0.0009	1.383E-08	TRUE	7804	0.004097838	32.11111111
x-03094	rs964184	C	G	0.8617	-0.0252	0.0032	7.949E-15	TRUE	7804	0.007883995	62.015625
x-03094	rs11825181	A	G	0.0724	0.0232	0.0041	1.325E-08	TRUE	7804	0.004086136	32.01903629
x-03094	rs6679002	T	G	0.5914	0.0141	0.0018	2.16E-15	TRUE	7804	0.007801436	61.36111111
x-03094	rs9855331	A	T	0.7473	0.0147	0.0027	3.809E-08	TRUE	7804	0.003783933	29.64197531
x-03094	rs2187375	A	G	0.1651	-0.0221	0.0026	2.649E-17	TRUE	7804	0.009173147	72.25
x-03094	rs1260326	T	C	0.4088	0.0133	0.0024	1.827E-08	TRUE	7804	0.003919745	30.71006944
x-11538	rs2199680	A	T	0.8309	-0.1032	0.0054	1.47E-80	TRUE	7804	0.044708542	365.2345679
x-11538	rs4149006	T	G	0.1479	-0.0725	0.0058	5.43E-36	TRUE	7804	0.019628781	156.25
x-11538	rs4149002	A	G	0.1471	-0.071	0.0057	2.642E-35	TRUE	7804	0.019493957	155.1554324
x-11538	rs4149081	A	G	0.1708	0.1049	0.0054	2.16E-83	TRUE	7804	0.04612513	377.3665981
x-11538	rs12829704	A	G	0.1943	-0.0909	0.005	1.884E-73	TRUE	7804	0.04063088	330.5124
x-11538	rs2108622	T	C	0.2918	-0.0248	0.0043	8.492E-09	TRUE	7804	0.00424426	33.26338561
x-11538	rs1871395	A	G	0.8385	-0.1101	0.0055	1.06E-89	TRUE	7804	0.048841061	400.7276033
x-11538	rs11045975	A	G	0.1937	-0.0646	0.0055	1.696E-31	TRUE	7804	0.017370495	137.9557025
x-11538	rs4149056	T	C	0.838	-0.1085	0.0054	4.31E-89	TRUE	7804	0.049186943	403.7122771
x-11538	rs12826576	A	G	0.1637	-0.0512	0.0051	2.81E-23	TRUE	7804	0.012749979	100.7858516
x-11538	rs12317268	A	G	0.8385	-0.11	0.0055	1.5E-89	TRUE	7804	0.048756704	400
x-11538	rs10770772	A	G	0.7837	0.0523	0.0055	4.131E-21	TRUE	7804	0.011454012	90.42280992
x-11538	rs7306033	A	G	0.849	0.0488	0.0057	1.085E-17	TRUE	7804	0.009304921	73.29763004
x-11538	rs7969341	A	G	0.8293	-0.1051	0.0054	1.053E-83	TRUE	7804	0.04629303	378.8069273
x-11538	rs4149022	A	G	0.248	-0.0522	0.0048	3.432E-27	TRUE	7804	0.014928258	118.265625
acetylcarnitine	rs12356193	A	G	0.8359	0.027	0.0027	4.012E-23	TRUE	7805	0.012650221	100
acetylcarnitine	rs7727544	T	C	0.5545	-0.0144	0.0025	9.595E-09	TRUE	7805	0.004232821	33.1776
acetylcarnitine	rs273914	A	T	0.3668	0.0168	0.0025	2.388E-11	TRUE	7805	0.005752546	45.1584

acetylcarnitine	rs419291	T	C	0.3687	0.0173	0.0025	9.831E-12	TRUE	7805	0.006097936	47.8864
acetylcarnitine	rs1171614	T	C	0.2231	-0.0279	0.0028	3.375E-23	TRUE	7805	0.012561157	99.2869898
acetylcarnitine	rs1171617	T	G	0.7762	0.0279	0.0028	3.458E-23	TRUE	7805	0.012561157	99.2869898
acetylcarnitine	rs273913	T	C	0.3816	0.0169	0.0025	3.009E-11	TRUE	7805	0.005820833	45.6976
acetylcarnitine	rs272869	A	G	0.3706	0.0168	0.0025	2.66E-11	TRUE	7805	0.005752546	45.1584
acetylcarnitine	rs2405522	A	G	0.1691	-0.0164	0.0027	1.134E-09	TRUE	7805	0.004704778	36.89437586
acetylcarnitine	rs274567	T	C	0.3709	0.0176	0.0025	2.849E-12	TRUE	7805	0.006309913	49.5616
acetylcarnitine	rs274570	T	C	0.2949	0.0159	0.0026	9.808E-10	TRUE	7805	0.004768685	37.39792899
acetylcarnitine	rs1171615	T	C	0.777	0.0279	0.0028	3.401E-23	TRUE	7805	0.012561157	99.2869898
acetylcarnitine	rs272881	A	G	0.6202	-0.0167	0.0025	3.587E-11	TRUE	7805	0.005684656	44.6224
dihomo-linolenate (20:3n3 or n6)	rs174550	T	C	0.6646	-0.0191	0.0026	1.996E-13	TRUE	7805	0.006866804	53.96597633
dihomo-linolenate (20:3n3 or n6)	rs174535	T	C	0.6645	-0.0191	0.0026	2.323E-13	TRUE	7805	0.006866804	53.96597633
dihomo-linolenate (20:3n3 or n6)	rs174538	A	G	0.3058	0.0193	0.0026	1.741E-13	TRUE	7805	0.007010351	55.10207101
dihomo-linolenate (20:3n3 or n6)	rs968567	T	C	0.1796	0.0329	0.0035	2.144E-21	TRUE	7805	0.011194219	88.36
dihomo-linolenate (20:3n3 or n6)	rs174556	T	C	0.301	0.0216	0.0026	1.466E-16	TRUE	7805	0.008765252	69.01775148
dihomo-linolenate (20:3n3 or n6)	rs174578	A	T	0.3406	0.0193	0.0026	1.019E-13	TRUE	7805	0.007010351	55.10207101
dihomo-linolenate (20:3n3 or n6)	rs1535	A	G	0.6631	-0.0191	0.0026	1.939E-13	TRUE	7805	0.006866804	53.96597633
dihomo-linolenate (20:3n3 or n6)	rs174548	C	G	0.6978	-0.0222	0.0026	2.189E-17	TRUE	7805	0.009254405	72.90532544
dihomo-linolenate (20:3n3 or n6)	rs174601	T	C	0.3407	0.0186	0.0026	1.332E-12	TRUE	7805	0.006514302	51.17751479
dihomo-linolenate (20:3n3 or n6)	rs6498540	A	G	0.7042	0.0172	0.0026	2.551E-11	TRUE	7805	0.005575823	43.76331361
x-09789	rs7642243	C	G	0.3882	0.0481	0.006	7.499E-16	TRUE	7805	0.008166827	64.26694444
x-09789	rs556339	T	C	0.2533	0.0382	0.0061	4.827E-10	TRUE	7805	0.004999395	39.21633969
10-undecenoate (11:1n1)	rs6077678	A	G	0.848	-0.0228	0.0041	3.382E-08	TRUE	7806	0.003945993	30.92444973
10-undecenoate (11:1n1)	rs9333029	A	G	0.8669	-0.0695	0.0042	1.524E-61	TRUE	7806	0.033889811	273.8236961
10-undecenoate (11:1n1)	rs1572603	A	G	0.13	0.073	0.005	2.808E-48	TRUE	7806	0.026581338	213.16
10-undecenoate (11:1n1)	rs6663731	A	T	0.8799	-0.068	0.0042	2.138E-58	TRUE	7806	0.032489743	262.1315193
betaine	rs2851391	T	C	0.4582	-0.0121	0.0018	1.146E-11	TRUE	7806	0.005755596	45.1882716
betaine	rs495360	A	C	0.497	-0.019	0.003	2.399E-10	TRUE	7806	0.005112228	40.11111111
betaine	rs16876394	T	C	0.8993	0.0304	0.0034	1.488E-19	TRUE	7806	0.010137611	79.94463668
betaine	rs13182512	A	T	0.3813	0.0134	0.0024	2.059E-08	TRUE	7806	0.00397766	31.17361111
betaine	rs715	T	C	0.713	0.0221	0.0025	2.077E-19	TRUE	7806	0.00991174	78.1456
tyrosine	rs6901004	C	G	0.5743	-0.0097	0.0016	8.96E-10	TRUE	7807	0.004685755	36.75390625
tyrosine	rs12192718	A	T	0.1447	0.0142	0.0023	5.224E-10	TRUE	7807	0.004858717	38.11720227
tyrosine	rs9940990	A	T	0.3502	-0.0093	0.0016	1.48E-08	TRUE	7807	0.0043089	33.78515625
tyrosine	rs9400467	T	C	0.7059	-0.0121	0.0016	6.541E-14	TRUE	7807	0.007272382	57.19140625
tyrosine	rs172650	A	C	0.6543	0.0102	0.0016	2.818E-10	TRUE	7807	0.005178706	40.640625
tyrosine	rs12728678	T	C	0.2003	-0.0096	0.0017	2.189E-08	TRUE	7807	0.004068086	31.88927336
valine	rs1440581	T	C	0.4577	-0.0081	0.0012	2.558E-12	TRUE	7808	0.005801507	45.5625
valine	rs893971	A	G	0.5863	-0.0066	0.0012	1.899E-08	TRUE	7808	0.00385928	30.25
1-eicosatrienoylglycerophosphochol	rs174550	T	C	0.6653	-0.0207	0.0035	3.432E-09	TRUE	7809	0.004459315	34.97877551
1-eicosatrienoylglycerophosphochol	rs174535	T	C	0.6652	-0.0204	0.0035	5.8E-09	TRUE	7809	0.004331552	33.9722449
1-eicosatrienoylglycerophosphochol	rs174538	A	G	0.305	0.0217	0.0035	9.128E-10	TRUE	7809	0.004898413	38.44
1-eicosatrienoylglycerophosphochol	rs968567	T	C	0.1798	0.04	0.0045	2.841E-19	TRUE	7809	0.010016762	79.01234568
1-eicosatrienoylglycerophosphochol	rs3198697	T	C	0.4174	0.0193	0.0034	1.65E-08	TRUE	7809	0.004109349	32.22231834
1-eicosatrienoylglycerophosphochol	rs174556	T	C	0.3002	0.0271	0.0035	1.742E-14	TRUE	7809	0.007618783	59.95183673
1-eicosatrienoylglycerophosphochol	rs174578	A	T	0.34	0.0211	0.0035	1.714E-09	TRUE	7809	0.004632515	36.34367347
1-eicosatrienoylglycerophosphochol	rs1535	A	G	0.6638	-0.0207	0.0035	3.267E-09	TRUE	7809	0.004459315	34.97877551
1-eicosatrienoylglycerophosphochol	rs174548	C	G	0.6985	-0.0272	0.0035	1.301E-14	TRUE	7809	0.007674682	60.39510204
1-eicosatrienoylglycerophosphochol	rs174601	T	C	0.3406	0.0214	0.0035	1.369E-09	TRUE	7809	0.00476455	37.3844898
1-eicosatrienoylglycerophosphochol	rs6498540	A	G	0.7038	0.019	0.0035	4.86E-08	TRUE	7809	0.003759584	29.46938776
3-dehydrocarnitine*	rs316020	A	G	0.0988	-0.0224	0.0041	3.804E-08	TRUE	7809	0.003807817	29.84889946
3-dehydrocarnitine*	rs316019	A	C	0.0986	-0.0229	0.0041	2.059E-08	TRUE	7809	0.003979022	31.19631172
3-dehydrocarnitine*	rs7727544	T	C	0.5545	-0.0212	0.0025	7.686E-18	TRUE	7809	0.009124631	71.9104
3-dehydrocarnitine*	rs273914	A	T	0.3675	0.025	0.0025	5.613E-24	TRUE	7809	0.012643823	100
3-dehydrocarnitine*	rs419291	T	C	0.369	0.026	0.0025	2.205E-25	TRUE	7809	0.013661464	108.16
3-dehydrocarnitine*	rs1981524	T	C	0.1981	0.0187	0.0026	1.052E-12	TRUE	7809	0.006580724	51.72928994
3-dehydrocarnitine*	rs273913	T	C	0.3822	0.0261	0.0025	1.084E-25	TRUE	7809	0.013765305	108.9936
3-dehydrocarnitine*	rs272869	A	G	0.3709	0.0258	0.0025	1.494E-25	TRUE	7809	0.013454913	106.5024
3-dehydrocarnitine*	rs2291428	C	G	0.2351	-0.0168	0.0026	8.707E-11	TRUE	7809	0.005318151	41.75147929
3-dehydrocarnitine*	rs2405522	A	G	0.17	-0.0321	0.0033	1.226E-22	TRUE	7809	0.011971709	94.61983471
3-dehydrocarnitine*	rs274567	T	C	0.3713	0.0256	0.0025	3.57E-25	TRUE	7809	0.013249872	104.8576
3-dehydrocarnitine*	rs274570	T	C	0.2947	0.0249	0.0026	2.336E-22	TRUE	7809	0.01160875	91.71745562
3-dehydrocarnitine*	rs11950562	A	C	0.5233	0.0161	0.0025	5.832E-11	TRUE	7809	0.005282942	41.4736
3-dehydrocarnitine*	rs272881	A	G	0.6198	-0.0257	0.0025	2.253E-25	TRUE	7809	0.013352204	105.6784
x-12524	rs2780242	T	C	0.1179	-0.0117	0.0019	7.08E-10	TRUE	7809	0.004832427	37.91966759
x-12524	rs11076175	A	G	0.845	0.011	0.002	3.798E-08	TRUE	7809	0.003858788	30.25
creatinine	rs2042499	A	C	0.4837	0.0052	0.0009	4.883E-08	TRUE	7810	0.004256163	33.38271605
x-11317	rs6593265	A	C	0.311	0.011	0.002	3.798E-08	TRUE	7811	0.003857803	30.25
x-11317	rs7499892	T	C	0.175	-0.02	0.003	2.617E-11	TRUE	7811	0.005657789	44.44444444
x-11317	rs11076175	A	G	0.845	0.019	0.003	2.399E-10	TRUE	7811	0.005108973	40.11111111
x-11787	rs10469966	A	G	0.2283	-0.0385	0.0017	1.16E-112	TRUE	7811	0.061616542	512.8892734
x-11787	rs4917639	A	C	0.8021	0.0293	0.0017	8.918E-65	TRUE	7811	0.036637066	297.0553633
x-11787	rs1853207	T	C	0.0593	-0.0402	0.0026	7.573E-53	TRUE	7811	0.029696573	239.0591716
x-11787	rs1934963	T	C	0.8021	0.0293	0.0017	8.691E-65	TRUE	7811	0.036637066	297.0553633
x-11787	rs7573275	A	G	0.275	-0.0262	0.0017	2.081E-56	TRUE	7811	0.029511316	237.5224913
x-11787	rs10206899	T	C	0.78	0.0399	0.0017	7.74E-123	TRUE	7811	0.065878638	550.8685121
x-11787	rs13538	A	G	0.7803	0.0396	0.0017	9.61E-121	TRUE	7811	0.064955813	542.615917
1-palmitoleoylglycerophosphocholi	rs603424	A	G	0.181	-0.0274	0.0043	2.267E-10	TRUE	7812	0.005170714	40.6035695
3-carboxy-4-methyl-5-propyl-2-fura	rs3799344	T	C	0.4463	0.0432	0.0077	1.988E-08	TRUE	7812	0.004013077	31.47647158
3-carboxy-4-methyl-5-propyl-2-fura	rs1165196	A	G	0.5529	-0.0476	0.0077	5.619E-10	TRUE	7812	0.004868004	38.21487603
3-carboxy-4-methyl-5-propyl-2-fura	rs11754288	A	G	0.4322	0.0488	0.0077	2.246E-10	TRUE	7812	0.005115272	40.16596391
3-carboxy-4-methyl-5-propyl-2-fura	rs2762353	A	G	0.4498	0.0485	0.0077	3.041E-10	TRUE	7812	0.005052889	39.67363805
3-carboxy-4-methyl-5-propyl-2-fura	rs1165209	A	G	0.5535	-0.048	0.0077	4.253E-10	TRUE	7812	0.004949756	38.85984146
isobutyrylcarnitine	rs10945649	A	T	0.1293	-0.0361	0.0054	1.822E-11	TRUE	7812	0.005688361	44.69170096
isobutyrylcarnitine	rs2404602	A	G	0.4398	0.0259	0.0036	3.558E-13	TRUE	7812	0.006582097	51.76003086
isobutyrylcarnitine	rs7746102	T	C	0.7629	-0.0296	0.0043	6.5E-12	TRUE	7812	0.006029175	47.38561385
isobutyrylcarnitine	rs2291449	A	G	0.9137	-0.0358	0.0062	8.452E-09	TRUE	7812	0.004249823	33.34131113
isobutyrylcarnitine	rs662138	C	G	0.832	0.0767	0.0052	4.976E-49	TRUE	7812	0.027095187	217.5625
lysine	rs8056893	A	C	0.7333	0.0143	0.0017	2.083E-17	TRUE	7812	0.008976273	70.75778547
lysine	rs2863979	A	G	0.7241	0.0143	0.0017	1.443E-17	TRUE	7812	0.008976273	70.75778547
phenylacetylglutamine	rs4797105	C	G	0.7968	0.0406	0.0074	3.586E-08	TRUE	7812	0.003838452	30.10153397
cholesterol	rs602633	T	G	0.2163	-0.0102	0.0017	5.024E-09	TRUE	7813	0.004586572	36
cholesterol	rs445925	A	G	0.079	-0.025	0.004	4.105E-10	TRUE	7813	0.004974808	39.0625
cholesterol	rs4970834	T	C	0.1813	-0.0099	0.0018	2.284E-08	TRUE	7813	0.00385682	30.25
citrate	rs17804635	T	C	0.0934	-0.0187	0.0034	3.623E-08	TRUE	7813	0.00385682	30.25
citrate	rs6507399	A	G	0.6713	-0.0101	0.0018	1.703E-08	TRUE	7813	0.004013593	31.4845679
citrate	rs170149	A	G	0.074	0.0256	0.0035	1.421E-13	TRUE	7813	0.006800837	53.49877551
citrate	rs807670	A	C	0.421	0.011	0.0018	6.841E-10	TRUE	7813	0.004757202	37.34567901
citrate	rs835154	A	G	0.5753	-0.0114	0.0018	1.147E-10	TRUE	7813	0.005107671	40.11111111
citrate	rs868395	T	G	0.6653	0.0101	0.0018	2.031E-08	TRUE	7813	0.004013593	31.4845679
n1-methyladenosine	rs315988	T	C	0.2367	0.0072	0.0013	1.08E-08	TRUE	7813	0.003910738	30.67455621
propionylcarnitine	rs12356193	A	G	0.836	0.0328	0.0027	1.961E-33	TRUE	7813	0.018538542	147.5775034
propionylcarnitine	rs7727544	T	C	0.5553	-0.0148	0.0018	6.607E-16	TRUE	7813	0.008578648	67.60493827
propionylcarnitine	rs273914	A	T	0.3665	0.0176	0.0025	3.131E-12	TRUE	7813	0.006303493	49.5616
propionylcarnitine	rs419291	T	C	0.3685	0.0175	0.0025	6.017E-12	TRUE	7813	0.006232511	49
propionylcarnitine	rs1171614	T	C	0.2229	-0.0316	0.0028	2.494E-29	TRUE	7813	0.016040485	127.3673469
propionylcarnitine	rs1171617	T	G	0.7763	0.0316	0.0028	2.572E-29	TRUE	7813	0.016040485	127.3673469
propionylcarnitine	rs273913	T	C	0.3813	0.0168	0.0025	3.135E-11	TRUE	7813	0.00574669	45.1584
propionylcarnitine	rs272869	A	G	0.3704	0.0169	0.0025	1.883E-11	TRUE	7813	0.005814908	45.6976
propionylcarnitine	rs274567	T	C	0.3707	0.0166	0.0025	3.468E-11	TRUE	7813	0.005611442	44.0896
propionylcarnitine	rs274570	T	C	0.2947	0.0161	0.0026	5.315E-10	TRUE	7813	0.004883835	38.34467456
propionylcarnitine	rs11950562	A	C	0.5226	0.0143	0.0018	7.006E-15	TRUE	7813	0.008013367	63.11419753
propionylcarnitine	rs1171615	T	C	0.7771	0.0316	0.0028	2.52E-29	TRUE	7813	0.016040485	127.3673469
propionylcarnitine	rs662138	C	G	0.8299	0.0206	0.0027	3.146E-14	TRUE	7813	0.007395463	58.21124829
propionylcarnitine	rs2715311	A	G	0.3884	-0.0107	0.0018	6.574E-09	TRUE	7813	0.004502409	35.33641975
propionylcarnitine	rs272881	A	G	0.6205	-0.0168	0.0025	2.441E-11	TRUE	7813	0.00574669	45.1584
2-aminobutyrate	rs10211524	A	G	0.4188	0.0189	0.0023	5.588E-16	TRUE	7814	0.00856757	67.52551985
2-aminobutyrate	rs10022462	T	C	0.452	-0.0117	0.0018	4.548E-11	TRUE	7814	0.005377884	42.25
palmitoyl sphingomyelin	rs445925	A	G	0.079	-0.024	0.004	1.973E-09	TRUE	7814	0.004585987	36
palmitoyl sphingomyelin	rs633695	A	G	0.7183	-0.01	0.0018	2.472E-08	TRUE	7814	0.003934319	30.86419753
arachidonate (20:4n6)	rs174550	T	C	0.6674	0.0475	0.0025	7.431E-82	TRUE	7816	0.044148221	361
arachidonate (20:4n6)	rs174535	T	C	0.6675	0.0475	0.0025	2.045E-81	TRUE	7816	0.044148221	361
arachidonate (20:4n6)	rs174538	A	G	0.3028	-0.0464	0.0025	6.241E-76	TRUE	7816	0.042212452	344.4736
arachidonate (20:4n6)	rs968567	T	C	0.1817	-0.0323	0.0027	2.012E-33	TRUE	7816	0.01798096	143.1124829
arachidonate (20:4n6)	rs2727271	A	T	0.8934	0.0506	0.0034	6.182E-50	TRUE	7816	0.027556436	221.4844291
arachidonate (20:4n6)	rs174556	T	C	0.2981	-0.0488	0.0025	2.976E-84	TRUE	7816	0.046483956	381.0304
arachidonate (20:4n6)	rs174578	A	T	0.3379	-0.0461	0.0025	5.684E-77	TRUE	7816	0.041691049	340.0336
arachidonate (20:4n6)	rs1535	A	G	0.666	0.0469	0.0025	1.588E-79	TRUE	7816	0.043087694	351.9376
arachidonate (20:4n6)	rs174548	C	G	0.7005	0.0488	0.0025	1.429E-84	TRUE	7816	0.046483956	381.0304

arachidonate (20:4n6)	rs174601	T	C	0.3396	-0.0463	0.0025	4.986E-76	TRUE	7816	0.042038338	342.9904
arachidonate (20:4n6)	rs174450	T	G	0.539	0.0215	0.0018	2.551E-32	TRUE	7816	0.017926332	142.6697531
eicosapentaenoate (epa; 20:5n3)	rs174550	T	C	0.6666	0.0348	0.0036	5.037E-22	TRUE	7816	0.011814287	93.44444444
eicosapentaenoate (epa; 20:5n3)	rs174535	T	C	0.6666	0.0343	0.0036	2.545E-21	TRUE	7816	0.011481104	90.77854938
eicosapentaenoate (epa; 20:5n3)	rs174538	A	G	0.3037	-0.0344	0.0037	5.196E-21	TRUE	7816	0.010938361	86.43973703
eicosapentaenoate (epa; 20:5n3)	rs2727271	A	T	0.8928	0.0389	0.0054	4.443E-13	TRUE	7816	0.006595583	51.89334705
eicosapentaenoate (epa; 20:5n3)	rs174556	T	C	0.299	-0.0356	0.0037	1.965E-22	TRUE	7816	0.011705724	92.57560263
eicosapentaenoate (epa; 20:5n3)	rs174578	A	T	0.3387	-0.0329	0.0036	7.913E-20	TRUE	7816	0.010572705	83.51929012
eicosapentaenoate (epa; 20:5n3)	rs1535	A	G	0.6651	0.0341	0.0036	3.553E-21	TRUE	7816	0.011349119	89.72299383
eicosapentaenoate (epa; 20:5n3)	rs174548	C	G	0.6996	0.0353	0.0036	3.709E-22	TRUE	7816	0.012152061	96.14891975
eicosapentaenoate (epa; 20:5n3)	rs174601	T	C	0.3383	-0.0343	0.0042	5.681E-16	TRUE	7816	0.008460869	66.69444444
kynurenine	rs16924894	A	T	0.024	0.0814	0.0146	2.329E-08	TRUE	7816	0.003961273	31.08444361
kynurenine	rs2160860	A	T	0.6139	0.0095	0.0017	4.895E-08	TRUE	7816	0.003979542	31.2283737
kynurenine	rs4843718	A	G	0.2413	0.0253	0.0024	1.06E-25	TRUE	7816	0.014018539	111.1267361
kynurenine	rs8051149	A	G	0.2127	0.0258	0.0025	9.073E-26	TRUE	7816	0.013443025	106.5024
kynurenine	rs10085935	T	C	0.3782	-0.0103	0.0017	3.326E-09	TRUE	7816	0.004674736	36.70934256
kynurenine	rs3184504	T	C	0.4851	0.0149	0.0017	6.046E-18	TRUE	7816	0.009732905	76.8200692
proline	rs2518802	A	C	0.903	-0.0534	0.0033	4.152E-59	TRUE	7816	0.032415952	261.8512397
proline	rs11802885	A	T	0.3634	0.0098	0.0018	2.327E-08	TRUE	7816	0.003778145	29.64197531
proline	rs5747934	T	C	0.0468	0.0715	0.0051	1.997E-44	TRUE	7816	0.024530196	196.5494041
1-linoleoylglycerophosphoethanolar	rs174550	T	C	0.6675	-0.0439	0.0035	3.304E-36	TRUE	7817	0.019728729	157.3232653
1-linoleoylglycerophosphoethanolar	rs174535	T	C	0.6677	-0.044	0.0035	2.816E-36	TRUE	7817	0.019816929	158.0408163
1-linoleoylglycerophosphoethanolar	rs174538	A	G	0.3026	0.0424	0.0035	5.748E-33	TRUE	7817	0.018427978	146.7559184
1-linoleoylglycerophosphoethanolar	rs968567	T	C	0.1781	0.0295	0.0045	4.769E-11	TRUE	7817	0.005467614	42.97530864
1-linoleoylglycerophosphoethanolar	rs2727271	A	T	0.8925	-0.044	0.0052	2.6E-17	TRUE	7817	0.009076091	71.59763314
1-linoleoylglycerophosphoethanolar	rs1077835	A	G	0.7937	-0.0252	0.0042	2.859E-09	TRUE	7817	0.004584235	36
1-linoleoylglycerophosphoethanolar	rs174556	T	C	0.2981	0.0423	0.0035	6.368E-33	TRUE	7817	0.018342749	146.0644898
1-linoleoylglycerophosphoethanolar	rs174578	A	T	0.3378	0.0407	0.0035	2.435E-31	TRUE	7817	0.017004511	135.2236735
1-linoleoylglycerophosphoethanolar	rs1535	A	G	0.6661	-0.0432	0.0035	4.205E-35	TRUE	7817	0.019116515	152.3461224
1-linoleoylglycerophosphoethanolar	rs174548	C	G	0.7006	-0.0431	0.0035	3.164E-34	TRUE	7817	0.019029797	151.6416327
1-linoleoylglycerophosphoethanolar	rs2070895	A	G	0.21	0.0257	0.0042	1.215E-09	TRUE	7817	0.004767078	37.44274376
1-linoleoylglycerophosphoethanolar	rs174601	T	C	0.3395	0.0395	0.0035	5.355E-29	TRUE	7817	0.016032409	127.3673469
docosahexaenoate (dha; 22:6n3)	rs7773173	C	G	0.5385	-0.0184	0.0034	3.868E-08	TRUE	7818	0.003732143	29.28719723
x-02249	rs1934955	A	G	0.713	-0.0301	0.0037	3.329E-16	TRUE	7818	0.008394078	66.18042367
x-02249	rs1853207	T	C	0.0598	0.0402	0.0072	2.848E-08	TRUE	7818	0.003971579	31.17361111
x-02249	rs2071426	T	C	0.7125	-0.0286	0.0037	9.268E-15	TRUE	7818	0.007584492	59.74872169
urate	rs16868246	C	G	0.2113	-0.0333	0.0017	1.071E-84	TRUE	7819	0.04677716	383.6989619
urate	rs938554	C	G	0.2256	-0.0348	0.0017	5.85E-93	TRUE	7819	0.050867042	419.0449827
docosapentaenoate (n3 dpa; 22:5n3)	rs174550	T	C	0.6676	0.0252	0.0035	4.927E-13	TRUE	7821	0.006584663	51.84
docosapentaenoate (n3 dpa; 22:5n3)	rs174535	T	C	0.6677	0.0252	0.0035	5.063E-13	TRUE	7821	0.006584663	51.84
docosapentaenoate (n3 dpa; 22:5n3)	rs174538	A	G	0.3025	-0.0264	0.0035	8.902E-14	TRUE	7821	0.007222068	56.89469388
docosapentaenoate (n3 dpa; 22:5n3)	rs174556	T	C	0.2937	-0.0258	0.0041	2.656E-10	TRUE	7821	0.005037512	39.59785842
docosapentaenoate (n3 dpa; 22:5n3)	rs174578	A	T	0.3377	-0.0241	0.0035	5.342E-12	TRUE	7821	0.006025746	47.41306122
docosapentaenoate (n3 dpa; 22:5n3)	rs1535	A	G	0.6662	0.0252	0.0035	5.202E-13	TRUE	7821	0.006584663	51.84
docosapentaenoate (n3 dpa; 22:5n3)	rs174548	C	G	0.7048	0.0256	0.0041	2.944E-10	TRUE	7821	0.0049601	38.98631767
docosapentaenoate (n3 dpa; 22:5n3)	rs174601	T	C	0.3378	-0.0247	0.0041	1.236E-09	TRUE	7821	0.004619056	36.29327781
glutamine	rs2657879	A	G	0.8179	0.015	0.0017	6.147E-18	TRUE	7821	0.009856451	77.85467128
creatine	rs10164524	A	G	0.6781	0.0217	0.0038	8.018E-09	TRUE	7822	0.004151716	32.6101108
creatine	rs715	T	C	0.7113	-0.0451	0.0044	9.628E-25	TRUE	7822	0.013253648	105.0625
caprate (10:0)	rs8043217	A	G	0.524	0.009	0.0016	2.793E-08	TRUE	7802	0.00403907	31.640625
caprate (10:0)	rs10514491	T	C	0.6781	0.0094	0.0017	4.457E-08	TRUE	7802	0.003903492	30.57439446
x-03056	rs1005390	T	G	0.7998	0.0261	0.0034	2.5E-14	TRUE	7812	0.007486817	58.92820069
x-03056	rs11101730	A	T	0.9414	-0.0553	0.0076	3.275E-13	TRUE	7812	0.006731741	52.94477147
x-03056	rs662138	C	G	0.8307	0.0342	0.0035	1.035E-22	TRUE	7812	0.012074745	95.48081633
x-03056	rs12602901	C	G	0.1717	-0.0368	0.0034	1.145E-26	TRUE	7812	0.014774447	117.1487889
x-04499	rs1030420	T	C	0.1884	-0.0221	0.0032	1.016E-11	TRUE	6948	0.006817947	47.69628906
x-11423	rs651007	T	C	0.2007	-0.0152	0.0024	2.78E-10	TRUE	7765	0.005139083	40.11111111
x-11423	rs6151429	T	C	0.9291	-0.0289	0.0034	6.507E-18	TRUE	7765	0.009218795	72.25
x-11423	rs649129	T	C	0.2138	-0.0142	0.0024	3.015E-09	TRUE	7765	0.004488066	35.00694444
x-11445	rs2199680	A	T	0.8189	-0.0654	0.011	2.529E-09	TRUE	2570	0.01356764	35.34842975
x-11445	rs10491431	A	C	0.1504	0.0807	0.012	1.591E-11	TRUE	2570	0.017293202	45.225625
x-11445	rs296396	T	C	0.1687	-0.0664	0.0118	1.803E-08	TRUE	2570	0.012170849	31.66446423
x-11445	rs4149081	A	G	0.1794	0.068	0.0109	5.218E-10	TRUE	2570	0.01491778	38.91928289
x-11445	rs1871395	A	G	0.8288	-0.0654	0.0113	7.719E-09	TRUE	2570	0.012865943	33.49643668
x-11445	rs4149056	T	C	0.8281	-0.0659	0.0111	2.483E-09	TRUE	2570	0.013529319	35.24722019
x-11445	rs12317268	A	G	0.8289	-0.0651	0.0113	8.871E-09	TRUE	2570	0.012749679	33.18983476
x-11445	rs296381	T	C	0.8303	0.0663	0.0117	1.35E-08	TRUE	2570	0.012340407	32.11111111
x-11445	rs7969341	A	G	0.8198	-0.0667	0.011	1.124E-09	TRUE	2570	0.014104704	36.76768595
x-11593	rs10412803	T	C	0.3119	0.0097	0.0018	4.416E-08	TRUE	7788	0.003714977	29.04012346
x-11593	rs4680	A	G	0.5069	-0.0486	0.0017	4.66E-178	TRUE	7788	0.094975005	817.2871972
x-11593	rs9318225	T	C	0.6829	-0.0106	0.0018	1.946E-09	TRUE	7788	0.004433138	34.67901235
x-11593	rs174675	T	C	0.2812	0.0155	0.0018	2.281E-18	TRUE	7788	0.009431418	74.15123457
x-11593	rs7707010	A	G	0.9582	0.0252	0.0044	9.611E-09	TRUE	7788	0.004194155	32.80165289
x-11593	rs2686184	A	G	0.408	-0.0097	0.0017	1.889E-08	TRUE	7788	0.004163015	32.55709343
x-11786	rs7061710	C	G	0.2282	0.0453	0.0061	7.517E-14	TRUE	2811	0.019241458	55.14888471
x-11793	rs3923630	A	G	0.333	-0.028	0.005	2.144E-08	TRUE	7611	0.004103444	31.36
x-11793	rs887829	T	C	0.3275	0.0932	0.0041	3E-114	TRUE	7611	0.063576236	516.7305175
x-11793	rs4148324	T	G	0.6718	-0.0916	0.0041	1.59E-111	TRUE	7611	0.061545291	499.1409875
x-12095	rs6430553	T	C	0.393	-0.0181	0.0026	6.293E-12	TRUE	7711	0.006245666	48.46301775
x-12100	rs2160860	A	T	0.6141	0.012	0.0018	7.347E-11	TRUE	7499	0.005891797	44.44444444
x-12100	rs4843718	A	G	0.2413	0.0158	0.0026	1.714E-09	TRUE	7499	0.00490039	36.92899408
x-12100	rs8051149	A	G	0.2099	0.0153	0.0027	7.998E-09	TRUE	7499	0.004263795	32.11111111
x-12100	rs10085935	T	C	0.3777	-0.0105	0.0018	1.292E-08	TRUE	7499	0.004517145	34.02777778
x-12244	rs8002180	T	C	0.7203	-0.0239	0.0024	8.226E-23	TRUE	6608	0.014785435	99.16840278
x-12244	rs7775554	A	C	0.5279	0.019	0.0024	1.35E-15	TRUE	6608	0.009395395	62.67361111
x-12244	rs6804368	A	G	0.5714	0.0221	0.0024	8.172E-21	TRUE	6608	0.012669359	84.79340278
x-12441	rs2271316	C	G	0.3822	0.1133	0.0113	1.791E-23	TRUE	2761	0.035132121	100.5316783
x-12442	rs12566232	A	C	0.713	0.04	0.0042	1.2E-21	TRUE	7780	0.011524123	90.70294785
x-12442	rs6685187	A	G	0.4088	-0.0432	0.0041	2.879E-26	TRUE	7780	0.014069111	111.0196312
x-12442	rs16833668	A	C	0.3144	-0.0422	0.0041	2.503E-24	TRUE	7780	0.013433951	105.9393218
x-12510	rs3799344	T	C	0.4467	0.0208	0.0036	1.046E-08	TRUE	7566	0.004392819	33.38271605
x-12510	rs10469966	A	G	0.2261	-0.1132	0.0045	6.82E-141	TRUE	7566	0.077182249	632.8019753
x-12510	rs1466245	T	C	0.0511	0.0481	0.0081	2.775E-09	TRUE	7566	0.004639107	35.26306965
x-12510	rs1165196	A	G	0.5525	-0.0224	0.0036	6.157E-10	TRUE	7566	0.005091058	38.71604938
x-12510	rs11754288	A	G	0.4326	0.0219	0.0036	1.486E-09	TRUE	7566	0.004867409	37.00694444
x-12510	rs2762353	A	G	0.4499	0.0221	0.0036	1.181E-09	TRUE	7566	0.004956275	37.68595679
x-12510	rs7573275	A	G	0.273	-0.084	0.0043	1.246E-83	TRUE	7566	0.048015894	381.611682
x-12510	rs10206899	T	C	0.7826	0.1181	0.0044	1.39E-155	TRUE	7566	0.086941423	720.4344008
x-12510	rs1165209	A	G	0.5531	-0.0226	0.0036	5.13E-10	TRUE	7566	0.005181902	39.41049383
x-12510	rs13538	A	G	0.7828	0.1186	0.0044	7.89E-157	TRUE	7566	0.087614514	726.5475207
x-12990	rs2727271	A	T	0.8882	0.0551	0.0083	2.735E-11	TRUE	2581	0.016788274	44.07040209
x-13431	rs12566232	A	C	0.7095	-0.0446	0.005	5.694E-19	TRUE	6591	0.011927983	79.5664
x-13431	rs3738934	T	C	0.6231	-0.1056	0.0043	1.21E-134	TRUE	6591	0.083832867	603.1022174
x-13431	rs6685187	A	G	0.413	0.0331	0.0049	1.537E-11	TRUE	6591	0.006875687	45.63140358
x-13431	rs16833668	A	C	0.3168	0.0439	0.005	1.061E-18	TRUE	6591	0.011560795	77.0884
x-14189	rs651007	T	C	0.2089	-0.0461	0.0075	9.578E-10	TRUE	2745	0.013576887	37.78151111
x-14189	rs4351	A	G	0.475	-0.0581	0.006	3.642E-22	TRUE	2745	0.033030871	93.76694444
x-14189	rs649129	T	C	0.2178	-0.0437	0.0074	4.32E-09	TRUE	2745	0.012545106	34.873813
x-14189	rs4362	T	C	0.5265	0.0586	0.0062	3.464E-21	TRUE	2745	0.031518169	89.33298647
x-14205	rs4351	A	G	0.4732	-0.0437	0.0057	1.167E-14	TRUE	1789	0.031809982	58.77777778
x-14205	rs13105073	A	T	0.9767	-0.1302	0.0227	9.177E-09	TRUE	1789	0.018057024	32.8980574
x-14205	rs4362	T	C	0.5288	0.0441	0.0059	7.524E-14	TRUE	1789	0.030283604	55.86929043
x-14208	rs4351	A	G	0.4734	-0.0587	0.0062	2.59E-21	TRUE	2455	0.035226281	89.63813736
x-14208	rs12266302	A	C	0.955	-0.1028	0.0188	4.705E-08	TRUE	2455	0.012032659	29.89995473
x-14208	rs4362	T	C	0.5281	0.0612	0.0064	1.366E-21	TRUE	2455	0.035909488	91.44140625
x-14304	rs651007	T	C	0.2052	-0.0447	0.0079	1.82E-08	TRUE	2434	0.012982701	32.01554238
x-14304	rs4351	A	G	0.4635	-0.0529	0.0063	3.111E-17	TRUE	2434	0.028151922	70.50667674
x-14304	rs649129	T	C	0.2141	-0.0437	0.0077	1.262E-08	TRUE	2434	0.01306025	32.20931017
x-14304	rs4362	T	C	0.5371	0.0529	0.0065	3.112E-16	TRUE	2434	0.026491337	66.23455621

Supplementary Table 4. Five Mendelian randomization models estimate the causal effects of gut microbiota on Myeloproliferative neoplasms

Notations: Green back means P-value < 0.05 in inverse variance weighted method

Outcome	Exposure	Method	Number of	Beta	Standard error	P-value	lo_ci	up_ci	OR	OR_lci95	OR_uci95
Myeloproliferative neoplasms	genus.Collinsella.id.815	Inverse variance weighted	9	-1.047915969	0.3660353	0.004198065	-1.765345158	-0.33048678	0.350667791	0.17112771	0.718573861
Myeloproliferative neoplasms	genus.Streptococcus.id.1853	Inverse variance weighted	10	-0.793853368	0.349762284	0.023226154	-1.479387446	-0.108319291	0.452099329	0.227777171	0.897341038
Myeloproliferative neoplasms	family.Desulfovibrionaceae.id.3169	Inverse variance weighted	8	0.925213351	0.413723886	0.025331711	0.114314534	1.736112169	2.522406362	1.121104695	5.675236116
Myeloproliferative neoplasms	genus..Eubacteriumhalliigroup.id.11338	Inverse variance weighted	13	-0.700385929	0.313609519	0.025528792	-1.315060587	-0.085711271	0.496393694	0.268458058	0.917859206
Myeloproliferative neoplasms	genus.Romboutsia.id.11347	Inverse variance weighted	11	0.682450348	0.339417004	0.044361956	0.017193021	1.347707675	1.97872035	1.017341671	3.848593184
Myeloproliferative neoplasms	family.Lachnospiraceae.id.1987	Inverse variance weighted	15	-0.705088263	0.351363049	0.044779181	-1.393759839	-0.016416686	0.494064965	0.24814058	0.983717333
Myeloproliferative neoplasms	genus..Eubacteriumxylanophilumgroup.id.14375	Inverse variance weighted	7	-0.712982558	0.363641871	0.049917258	-1.425720625	-0.000244491	0.490180025	0.240335209	0.999755539

Myeloproliferative neoplasms	genus.Oxalobacter.id.2978	Inverse variance weighted	10	0.323585313	0.174208789	0.063246182	-0.017863914	0.66503454	1.38207406	0.982294699	1.944557685
Myeloproliferative neoplasms	order.Bifidobacteriales.id.432	Inverse variance weighted	9	0.524909676	0.299189469	0.079355312	-0.061501684	1.111321036	1.690306166	0.940351362	3.038369538
Myeloproliferative neoplasms	genus..Ruminococcustorquesgroup.id.14377	Inverse variance weighted	10	0.632020616	0.362392664	0.081155332	-0.078269006	1.342310238	1.881408344	0.924715639	3.827876603
Myeloproliferative neoplasms	genus.Ruminiclostridium9.id.11357	Inverse variance weighted	7	0.739616792	0.427589177	0.083677287	-0.098457494	1.577691578	2.095132489	0.906233759	4.84376145
Myeloproliferative neoplasms	family.Bifidobacteriaceae.id.433	Inverse variance weighted	10	0.464889484	0.279227477	0.095930095	-0.082396371	1.012175338	1.591838255	0.920906866	2.751580126
Myeloproliferative neoplasms	genus.Haemophilus.id.3698	Inverse variance weighted	8	-0.472496313	0.28837901	0.10132661	-1.037719174	0.092726547	0.623444013	0.354261771	1.097161672
Myeloproliferative neoplasms	genus.Dialister.id.2183	Inverse variance weighted	11	0.459525788	0.282921406	0.10432948	-0.095000167	1.014051743	1.583322976	0.909372783	2.756748052
Myeloproliferative neoplasms	family.Pasteurellaceae.id.3689	Inverse variance weighted	10	-0.439383019	0.277195762	0.112943602	-0.982686712	0.103920674	0.644433902	0.374304099	1.109512438
Myeloproliferative neoplasms	order.Pasteurellales.id.3688	Inverse variance weighted	10	-0.439383019	0.277195762	0.112943602	-0.982686712	0.103920674	0.644433902	0.374304099	1.109512438
Myeloproliferative neoplasms	genus.Alistipes.id.968	Inverse variance weighted	12	0.560899278	0.357434989	0.116593334	-0.139673301	1.261471856	1.752247549	0.8696423	3.530614222
Myeloproliferative neoplasms	genus.RuminococcaceaeUCG010.id.11367	Inverse variance weighted	6	0.567280469	0.361578315	0.116671081	-0.14141303	1.275973967	1.763464727	0.868130674	3.582188645
Myeloproliferative neoplasms	genus.Butyricimonas.id.945	Inverse variance weighted	13	-0.403906369	0.260663935	0.121254567	-0.914807682	0.106994944	0.667706636	0.40059366	1.112928628
Myeloproliferative neoplasms	genus.Sutterella.id.2896	Inverse variance weighted	12	-0.413820866	0.280427689	0.140030456	-0.963459136	0.135817405	0.66111937	0.381570696	1.145472716
Myeloproliferative neoplasms	genus.Coprococcus3.id.11303	Inverse variance weighted	5	-0.931844513	0.643778137	0.147767519	-2.193649661	0.329960636	0.393826622	0.111509035	1.390913376
Myeloproliferative neoplasms	order.Lactobacillales.id.1800	Inverse variance weighted	11	-0.453476716	0.318992746	0.155145474	-1.078702498	0.171749065	0.635415149	0.340036438	1.187379841
Myeloproliferative neoplasms	genus.LachnospiraceaeUCG008.id.11328	Inverse variance weighted	10	0.359614784	0.264747908	0.174359285	-0.159291116	0.878520684	1.43277738	0.852748074	2.40733586
Myeloproliferative neoplasms	family.Oxalobacteraceae.id.2966	Inverse variance weighted	14	0.213651459	0.157428529	0.174738939	-0.094908457	0.522211375	1.238191019	0.909456185	1.685751359
Myeloproliferative neoplasms	genus.Bilophila.id.3170	Inverse variance weighted	13	-0.378019722	0.28146945	0.179264783	-0.929699843	0.1736604	0.685216987	0.394672156	1.189651491
Myeloproliferative neoplasms	genus.DefluviitaleaceaeUCG011.id.11287	Inverse variance weighted	8	-0.381923247	0.294522301	0.194715521	-0.959186957	0.195340462	0.682547439	0.383204321	1.215724824
Myeloproliferative neoplasms	genus.Fusicatenibacter.id.11305	Inverse variance weighted	18	0.426921266	0.330508358	0.196457263	-0.220875116	1.074717648	1.532531995	0.801816808	2.929165729
Myeloproliferative neoplasms	genus..Eubacteriumfissicatenagroup.id.14373	Inverse variance weighted	8	0.253518302	0.201259379	0.207792198	-0.140950081	0.647986685	1.288550962	0.868532667	1.911688121
Myeloproliferative neoplasms	genus.Victivallis.id.2256	Inverse variance weighted	8	-0.227233208	0.180986449	0.209287844	-0.581966647	0.127500232	0.796734956	0.558798326	1.135985132
Myeloproliferative neoplasms	order.Bacteroidales.id.913	Inverse variance weighted	12	0.617357233	0.492560231	0.210073288	-0.34806082	1.582775286	1.854021813	0.706055932	4.868448414
Myeloproliferative neoplasms	class.Bacteroidia.id.912	Inverse variance weighted	12	0.617357233	0.492560231	0.210073288	-0.34806082	1.582775286	1.854021813	0.706055932	4.868448414
Myeloproliferative neoplasms	genus..Eubacteriumeligensgroup.id.14372	Inverse variance weighted	6	-0.516555108	0.414864909	0.213088961	-1.329690329	0.296580113	0.596572139	0.264559175	1.345250328
Myeloproliferative neoplasms	order.Actinomycetales.id.420	Inverse variance weighted	3	-0.667080167	0.544507309	0.220534596	-1.734314492	0.400154159	0.513204865	0.176521165	1.492054694
Myeloproliferative neoplasms	family.Actinomycetaceae.id.421	Inverse variance weighted	3	-0.666856613	0.544493786	0.22067784	-1.734064433	0.400351208	0.513319607	0.176565312	1.49234873
Myeloproliferative neoplasms	genus.Phascolarctobacterium.id.2168	Inverse variance weighted	7	0.488149248	0.400277314	0.222643992	-0.296394287	1.272692783	1.629298001	0.74349422	3.570454087
Myeloproliferative neoplasms	genus.Lachnospira.id.2004	Inverse variance weighted	5	-0.618718208	0.51086585	0.225850648	-1.620015274	0.382578858	0.538634413	0.197895676	1.466060481
Myeloproliferative neoplasms	class.Actinobacteria.id.419	Inverse variance weighted	12	0.332815181	0.278847739	0.232659009	-0.213726388	0.87935675	1.394889472	0.807569315	2.409349394
Myeloproliferative neoplasms	genus.Hungatella.id.11306	Inverse variance weighted	4	-0.521728778	0.441360353	0.237168987	-1.386795071	0.343337514	0.593493642	0.249874854	1.409644457
Myeloproliferative neoplasms	order.Desulfovibrionales.id.3156	Inverse variance weighted	10	0.527525614	0.446385719	0.237296652	-0.347390395	1.402441622	1.69473369	0.706529449	4.065113329
Myeloproliferative neoplasms	family.Acidaminococcaceae.id.2166	Inverse variance weighted	6	-0.400093688	0.343530491	0.244159633	-1.07341345	0.273226074	0.670257248	0.341839671	1.314197317
Myeloproliferative neoplasms	genus.Sellimonas.id.14369	Inverse variance weighted	7	-0.206312482	0.181123687	0.254674132	-0.561314908	0.148689943	0.813578807	0.57045847	1.16031317
Myeloproliferative neoplasms	genus.RuminococcaceaeUCG002.id.11360	Inverse variance weighted	17	-0.337081636	0.296021423	0.254825425	-0.917283626	0.243120353	0.713850561	0.399603039	1.275222092
Myeloproliferative neoplasms	genus..Eubacteriumbrachygroup.id.11296	Inverse variance weighted	6	0.250988619	0.223056551	0.26049406	-0.186202221	0.688179459	1.285295457	0.830105713	1.990089195
Myeloproliferative neoplasms	genus..Eubacteriumoxidoreducensgroup.id.11339	Inverse variance weighted	3	-0.420954513	0.37420763	0.260621956	-1.154401468	0.312492443	0.656419959	0.315246165	1.366827611
Myeloproliferative neoplasms	genus.Escherichia.Shigella.id.3504	Inverse variance weighted	8	0.377374871	0.350253817	0.281287059	-0.309122611	1.063872353	1.458450938	0.734090757	2.897569705
Myeloproliferative neoplasms	genus.Slackia.id.825	Inverse variance weighted	6	-0.306309304	0.28437498	0.281421436	-0.863684265	0.251065657	0.736158887	0.421605909	1.285394477
Myeloproliferative neoplasms	genus.Ruminiclostridium6.id.11356	Inverse variance weighted	12	-0.300364189	0.287240303	0.29570438	-0.863355184	0.262626806	0.740548472	0.421744675	1.300341348
Myeloproliferative neoplasms	genus.Eggerthella.id.819	Inverse variance weighted	10	0.209318255	0.201254327	0.298308149	-0.185140226	0.603776735	1.232837292	0.83098775	1.829013472
Myeloproliferative neoplasms	genus.Catenibacterium.id.2153	Inverse variance weighted	4	0.259681593	0.250416855	0.299737132	-0.231135442	0.750498629	1.296517201	0.793631968	2.118055877
Myeloproliferative neoplasms	genus.Prevotella7.id.11182	Inverse variance weighted	9	-0.177787542	0.17450685	0.308298027	-0.519820967	0.164245884	0.837120258	0.594626996	1.178504055
Myeloproliferative neoplasms	family.FamilyXI.id.1936	Inverse variance weighted	8	-0.193209159	0.192244129	0.314887309	-0.570007653	0.183589335	0.824309544	0.565521111	1.201522298
Myeloproliferative neoplasms	genus.Oscillospira.id.2064	Inverse variance weighted	7	-0.355683912	0.366242512	0.331463398	-1.073519235	0.362151412	0.700694066	0.341803511	1.436416416
Myeloproliferative neoplasms	genus.RuminococcaceaeUCG013.id.11370	Inverse variance weighted	11	-0.385242733	0.401373895	0.337150779	-1.171935567	0.4014501	0.680285488	0.309766786	1.493989562
Myeloproliferative neoplasms	genus.RuminococcaceaeUCG004.id.11362	Inverse variance weighted	9	-0.264749015	0.278148811	0.3411857	-0.809920685	0.280422655	0.767398531	0.444893351	1.323689158
Myeloproliferative neoplasms	class.Gammaproteobacteria.id.3303	Inverse variance weighted	6	-0.545978479	0.577380647	0.344346159	-1.677644547	0.58568759	0.579274698	0.186813489	1.796225627
Myeloproliferative neoplasms	phylum.Euryarchaeota.id.55	Inverse variance weighted	11	-0.148778868	0.158475383	0.347826379	-0.459390619	0.161832882	0.861759656	0.631668455	1.175663751
Myeloproliferative neoplasms	family.Methanobacteriaceae.id.121	Inverse variance weighted	8	-0.170169933	0.183064899	0.352598593	-0.528977135	0.18863727	0.843521463	0.589207341	1.207602839
Myeloproliferative neoplasms	order.Methanobacteriales.id.120	Inverse variance weighted	8	-0.170169933	0.183064899	0.352598593	-0.528977135	0.18863727	0.843521463	0.589207341	1.207602839
Myeloproliferative neoplasms	class.Methanobacteria.id.119	Inverse variance weighted	8	-0.170169933	0.183064899	0.352598593	-0.528977135	0.18863727	0.843521463	0.589207341	1.207602839
Myeloproliferative neoplasms	genus.Roseburia.id.2012	Inverse variance weighted	11	0.378979207	0.413912084	0.35987508	-0.432288478	1.190246892	1.460792661	0.649022121	3.287892862
Myeloproliferative neoplasms	genus.Ruminococcus2.id.11374	Inverse variance weighted	13	0.232507592	0.256640364	0.364953543	-0.270507521	0.735522705	1.261760026	0.762992161	2.08657237
Myeloproliferative neoplasms	genus.Alloprevotella.id.961	Inverse variance weighted	5	-0.190908914	0.211027624	0.365643871	-0.604523057	0.222705228	0.826207841	0.546334935	1.249452216
Myeloproliferative neoplasms	order.Clostridiales.id.1863	Inverse variance weighted	12	-0.363419171	0.41056079	0.37606091	-1.16811832	0.441279978	0.695294925	0.310951502	1.554695923
Myeloproliferative neoplasms	family.Christensenellaceae.id.1866	Inverse variance weighted	10	0.268346973	0.308657939	0.384628807	-0.336622587	0.873316533	1.307800833	0.714178329	2.394840265
Myeloproliferative neoplasms	genus.RuminococcaceaeUCG005.id.11363	Inverse variance weighted	13	0.266145089	0.308702257	0.388609546	-0.338911335	0.871201513	1.304924375	0.712545624	2.389780482
Myeloproliferative neoplasms	order.Victivallales.id.2254	Inverse variance weighted	8	-0.178117505	0.206982582	0.389489542	-0.583803365	0.227568355	0.836844084	0.557772913	1.255543259
Myeloproliferative neoplasms	class.Lentisphaeria.id.2250	Inverse variance weighted	8	-0.178117505	0.206982582	0.389489542	-0.583803365	0.227568355	0.836844084	0.557772913	1.255543259
Myeloproliferative neoplasms	genus.Bifidobacterium.id.436	Inverse variance weighted	12	0.205296842	0.242533029	0.397290812	-0.270067895	0.680661578	1.2278895	0.763327667	1.975184038
Myeloproliferative neoplasms	genus.Lachnoclostridium.id.11308	Inverse variance weighted	13	-0.248559388	0.306802189	0.417847237	-0.849891678	0.352772902	0.779923541	0.427461233	1.423007944
Myeloproliferative neoplasms	genus.Holdemanella.id.11393	Inverse variance weighted	9	-0.18810031	0.232188226	0.417871249	-0.643189232	0.266988613	0.828531593	0.525613445	1.306025574
Myeloproliferative neoplasms	genus..Eubacteriumcoprostanoligenesgroup.id.11375	Inverse variance weighted	12	0.313222172	0.386741096	0.417996821	-0.444790375	1.071234719	1.36782539	0.640958623	2.918981399
Myeloproliferative neoplasms	family.Erysipelotrichaceae.id.2149	Inverse variance weighted	11	-0.386915908	0.485454469	0.425440628	-1.338406667	0.564574852	0.679148204	0.262263208	1.758699916
Myeloproliferative neoplasms	order.Erysipelotrichales.id.2148	Inverse variance weighted	11	-0.386915908	0.485454469	0.425440628	-1.338406667	0.564574852	0.679148204	0.262263208	1.758699916
Myeloproliferative neoplasms	class.Erysipelotrichia.id.2147	Inverse variance weighted	11	-0.386915908	0.485454469	0.425440628	-1.338406667	0.564574852	0.679148204	0.262263208	1.758699916
Myeloproliferative neoplasms	family.Streptococcaceae.id.1850	Inverse variance weighted	9	-0.377627802	0.476134212	0.427712518	-1.310850858	0.555595254	0.68548559	0.269590575	1.742978191
Myeloproliferative neoplasms	order.Selenomonadales.id.2165	Inverse variance weighted	12	0.255617341	0.325143842	0.431769688	-0.381664589	0.892899271	1.291258522	0.682724008	2.442199997
Myeloproliferative neoplasms	class.Negativicutes.id.2164	Inverse variance weighted	12	0.255617341	0.325143842	0.431769688	-0.381664589	0.892899271	1.291258522	0.682724008	2.442199997
Myeloproliferative neoplasms	order.Burkholderiales.id.2874	Inverse variance weighted	10	0.263989303	0.339085785	0.436255157	-0.400618836	0.928597442	1.302114267	0.669905356	2.530956872
Myeloproliferative neoplasms	phylum.Cyanobacteria.id.1500	Inverse variance weighted	7	-0.411320258	0.528963139	0.436806509	-1.44808801	0.625447493	0.662774639	0.235019213	1.869082172
Myeloproliferative neoplasms	genus.LachnospiraceaeUCG010.id.11330	Inverse variance weighted	10	0.306832554	0.398939702	0.441822001	-0.475089262	1.08875437	1.359113371	0.621829549	2.970571533
Myeloproliferative neoplasms	family.FamilyXIII.id.1957	Inverse variance weighted	5	0.491328591	0.6513179	0.450632697	-0.785254494	1.767911675	1.634486341	0.456003637	5.858605905
Myeloproliferative neoplasms	genus.Parasutterella.id.2892	Inverse variance weighted	13	0.171792858	0.231943496	0.458895465	-0.282816394	0.626402109	1.18743184	0.753658152	1.87086728
Myeloproliferative neoplasms	genus.LachnospiraceaeND3007group.id.11317	Inverse variance weighted	3	0.467518594	0.636788937	0.462838187	-0.780587722	1.715624911	1.59602888	0.458136675	5.560149024
Myeloproliferative neoplasms	genus.LachnospiraceaeUCG004.id.11324	Inverse variance weighted	12	-0.274037846	0.376968988	0.467256195	-1.012897063	0.46482137	0.7603033	0.363165341	1.591729833
Myeloproliferative neoplasms	phylum.Bacteroidetes.id.905	Inverse variance weighted	10	0.452574817	0.624475869	0.468619239	-0.771397886	1.676547519	1.572355505	0.462366281	5.347063434
Myeloproliferative neoplasms	class.Deltaproteobacteria.id.3087	Inverse variance weighted	11	0.343103639	0.502667984	0.494881673	-0.642125609	1.328332887	1.409314814	0.526172797	3.774745214
Myeloproliferative neoplasms	family.Lactobacillaceae.id.1836	Inverse variance weighted	7	-0.184162785	0.272428065	0.49903735	-0.718121792	0.349796221	0.831800388	0.487667337	1.418778402
Myeloproliferative neoplasms	class.Clostridia.id.1859	Inverse variance weighted	11	-0.296786731	0.440325326	0.50030054	-1.159824369	0.566250907	0.743202497	0.313541243	1.761650066
Myeloproliferative neoplasms	family.Porphyromonadaceae.id.943	Inverse variance weighted	9	-0.313618228	0.46811156	0.502880283	-1.231116885	0.603880429	0.730797973	0.291966303	1.829203139
Myeloproliferative neoplasms	genus.LachnospiraceaeUCG001.id.11321	Inverse variance weighted	11	-0.16250698	0.247228353	0.510978196	-0.647074551	0.322060591	0.850010157	0.523575231	1.379968386
Myeloproliferative neoplasms	order.NB1n.id.3953	Inverse variance weighted	12	-0.112111221	0.173629219	0.518478008	-0.45242449	0.228202047	0.893944826	0.636084101	1.25633914
Myeloproliferative neoplasms	genus.Anaerofilum.id.2053	Inverse variance weighted	9	-0.164165393	0.258845803	0.525936733	-0.671503167	0.343172381	0.848601658	0.510939972	1.409411697
Myeloproliferative neoplasms	phylum.Lentisphaerae.id.2238	Inverse variance weighted	9	-0.122109424	0.193839866	0.528727297	-0.502035562	0.257816714	0.885051517	0.605297284	1.294101606
Myeloproliferative neoplasms	genus.Lactococcus.id.1851	Inverse variance weighted	9	0.180014375	0.288264202	0.532314345	-0.384983461	0.745012211	1.197234573	0.68046189	2.106467157
Myeloproliferative neoplasms	genus.Turicibacter.id.2162	Inverse variance weighted	8	-0.179494461	0.289055971	0.534620787	-0.746044164	0.387055243	0.83569258	0.474238858	1.472637842
Myeloproliferative neoplasms	family.Peptococcaceae.id.2024	Inverse variance weighted	8	0.215120395	0.347481235	0.535860413	-0.465942826	0.896183616	1.240011179	0.627543162	2.45023421
Myeloproliferative neoplasms	genus.Lactobacillus.id.1837	Inverse variance weighted	7	-0.158346881	0.259114384	0.541128063	-0.666211074	0.349517312	0.853553649	0.513651081	1.418382747
Myeloproliferative neoplasms	genus.Eisenbergiella.id.11304	Inverse variance weighted	11	-0.117945951	0.193311166	0.541772236	-0.496835837	0.260943934	0.888744087	0.608452861	1.298154881
Myeloproliferative neoplasms	genus.RuminococcaceaeUCG011.id.11368	Inverse variance weighted	8	-0.105868225	0.175017317	0.545244494	-0.448902166	0.237165716	0.899543178	0.638328546	1.267651171
Myeloproliferative neoplasms	genus.Blautia.id.1992	Inverse variance weighted	11	-0.233773851	0.386915093	0.545711107	-0.992127433	0.524579731	0.791540802	0.370787027	1.68974855
Myeloproliferative neoplasms	genus.Ruminococcus1.id.11373	Inverse variance weighted	8	-0.215425581	0.357156382	0.54639641	-0.91545209	0.484600927	0.806198264	0.400335597	1.623526974
Myeloproliferative neoplasms	order.Rhodospirillales.id.2667	Inverse variance weighted	12	0.174481014	0.290249687	0.547746016	-0.394408372	0.7433704	1.190628136	0.674078725	2.103011572
Myeloproliferative neoplasms	genus.RuminococcaceaeUCG009.id.11366	Inverse variance weighted	10	0.141679781	0.236304587	0.548796682	-0.321477209	0.604836771	1.152207631	0.725077155	1.83095332
Myeloproliferative neoplasms	phylum.Firmicutes.id.1672	Inverse variance weighted	13	0.168404194	0.289707386	0.561044581	-0.399422281	0.73623067	1.183414844	0.670707414	2.088050113
Myeloproliferative neoplasms	family.Clostridiaceae1.id.1869	Inverse variance weighted	7	-0.2211184	0.383677989	0.564404013	-0.973127259	0.530890459	0.801621763	0.377899399	1.700445812
Myeloproliferative neoplasms	genus.Butyricicoccus.id.2055	Inverse variance weighted	7	0.233182086	0.410401313	0.569912307	-0.571204488	1.03756866	1.262611362	0.56484468	2.822346582
Myeloproliferative neoplasms	genus.Tyzzerella3.id.11335	Inverse variance weighted	12	-0.143826773	0.253882776	0.571048096	-0.641437014	0.353783468	0.866037756	0.526535242	1.424446715
Myeloproliferative neoplasms	genus.Odoribacter.id.952	Inverse variance weighted	5	0.385318971	0.714312912	0.589592931	-1.014734337	1.78537228	1.470083161	0.36249872	5.96179899
Myeloproliferative neoplasms	genus.Faecalibacterium.id.2057	Inverse variance weighted	10	-0.181538884	0.336888317	0.589976811	-0.841839986	0.478762217	0.833985816	0.430916913	1.61407529
Myeloproliferative neoplasms	genus.Olsenella.id.822	Inverse variance weighted	9	0.088529509	0.164803841	0.591142523	-0.234486019	0.411545038	1.092566493	0.790977292	1.509147675
Myeloproliferative neoplasms	genus.Anaerostipes.id.1991	Inverse variance weighted	12	0.174630701	0.328256269	0.594729442	-0.468751585	0.818012988	1.190806372	0.625783018	2.265992806
Myeloproliferative neoplasms	genus.Ruminiclostridium5.id.11355	Inverse variance weighted	10	0.176033363	0.353883404	0.618863658	-0.517568109	0.869654835	1.192489767	0.595968117	2.386087118
Myeloproliferative neoplasms	family.Rikenellaceae.id.967	Inverse variance weighted	16	0.147325852	0.297961505	0.620991286	-0.436678697	0.731330402	1.158731477	0.646179017	2.077843135
Myeloproliferative neoplasms	genus.FamilyXIIIUCG001.id.11294	Inverse variance weighted	7	0.178899951	0.36196794	0.621134906	-0.530557212	0.888357114	1.19590109	0.588277084	2.431132296
Myeloproliferative neoplasms	family.Ruminococcaceae.id.2050	Inverse variance weighted	7	-0.178060665	0.363251594	0.624002639	-0.89003379	0.53391246	0.836891652	0.410641877	1.705592334
Myeloproliferative neoplasms	genus.Veillonella.id.2198	Inverse variance weighted	6	-0.177140298	0.362875158	0.625438153	-0.888375609	0.534095013	0.837662254	0.411323361	1.705903722
Myeloproliferative neoplasms	family.Peptostreptococcaceae.id.2042	Inverse variance weighted	12	-0.12637309	0.262295002	0.629949769	-0.640471294	0.387725114	0.881285987	0.527043973	1.47362465
Myeloproliferative neoplasms	genus.Intestinimonas.id.2062	Inverse variance weighted	14	-0.147774474	0.317237435	0.641346757	-0.769559846	0.474010897	0.862625637	0.46321691	1.606424493
Myeloproliferative neoplasms	genus.LachnospiraceaeFCS020group.id.11314	Inverse variance weighted	11	-0.127900596	0.280732639	0.648680701	-0.678136568	0.422335375	0.879940844	0.507561919	1.52552006
Myeloproliferative neoplasms	genus.Paraprevotella.id.962	Inverse variance weighted	13	-0.085658657	0.197947485	0.665207841	-0.473635728	0.302318413	0.917907499	0.622734056	1.352991969
Myeloproliferative neoplasms	family.Victivallaceae.id.2255	Inverse variance weighted	9	0.075049745	0.173934234	0.666116877	-0.265861354	0.415960843	1.077937771	0.766545399	1.515826513
Myeloproliferative neoplasms	genus.Enterorhabdus.id.820	Inverse variance weighted	6	0.131278652	0.307131115	0.669062367	-0.470698332	0.733255637	1.14028548	0.624565961	2.081847326
Myeloproliferative neoplasms	genus..Ruminococcusgnavusgroup.id.14376	Inverse variance weighted	11	0.085498158	0.203406535	0.674243358	-0.31317865	0.484174966	1.089259555	0.731119287	1.622835562
Myeloproliferative neoplasms	genus..Eubacteriumruminantiumgroup.id.11340	Inverse variance weighted	16	0.077636439	0.184936641	0.67463142	-0.284839377	0.440112254	1.080729676	0.752135055	1.552881526
Myeloproliferative neoplasms	genus.LachnospiraceaeNK4A136group.id.11319	Inverse variance weighted	13	-0.129225454	0.313090276	0.679795773	-0.742882396	0.484431488	0.87877582	0.475740664	1.623251908
Myeloproliferative neoplasms	genus..Eubacteriumventriosumgroup.id.11341	Inverse variance weighted	13	-0.120902001	0.297674439	0.684628621	-0.704343902	0.462539901	0.886120795	0.494432864	1.588102489
Myeloproliferative neoplasms	phylum.Actinobacteria.id.400	Inverse variance weighted	14	0.118644739	0.294294088	0.686837685	-0.458171672	0.695461151	1.125969834	0.632438895	2.004633299
Myeloproliferative neoplasms	genus.RuminococcaceaeUCG014.id.11371	Inverse variance weighted	10	-0.179342528	0.445885553	0.687524698	-1.05327821	0.694593155	0.835819559	0.348792458	2.002894041
Myeloproliferative neoplasms	genus.Gordonibacter.id.821	Inverse variance weighted	10	0.066069895	0.166741929	0.691927661	-0.260744285	0.392884076	1.068301384	0.770477917	1.481246667
Myeloproliferative neoplasms	genus.Prevotella9.id.11183	Inverse variance weighted	13	0.129841739	0.329548272	0.693581667	-0.516072874	0.775756352	1.138648165	0.596859896	2.172234479
Myeloproliferative neoplasms	genus.Holdemania.id.2157	Inverse variance weighted	12	0.089073771	0.232771872	0.701967778	-0.367159098	0.54530664	1.093161297	0.692699429	1.725137297
Myeloproliferative neoplasms	genus.Akkermansia.id.4037	Inverse variance weighted	10	0.113287238	0.299899637	0.705615948	-0.47451605	0.701090526	1.11995358	0.622186091	2.015949955
Myeloproliferative neoplasms	family.Verrucomicrobiaceae.id.4036	Inverse variance weighted	10	0.112606133	0.299887371	0.707292552	-0.475173115	0.700385381	1.119191034	0.621777408	2.014528918
Myeloproliferative neoplasms	order.Verrucomicrobiales.id.4030	Inverse variance weighted	10	0.112456476	0.299808298	0.707590108	-0.475167788	0.700080739	1.119023551	0.62178072	2.013915303
Myeloproliferative neoplasms	class.Verrucomicrobiae.id.4029	Inverse variance weighted	10	0.112456476	0.299808298	0.707590108	-0.475167788	0.700080739	1.119023551	0.62178072	2.013915303
Myeloproliferative neoplasms	family.Alcaligenaceae.id.2875	Inverse variance weighted	11	-0.127225787	0.340105317	0.708346571	-0.793832208	0.539380635	0.880534837	0.452108896	1.714944356

Myeloproliferative neoplasms	order.MollicutesRF9.id.11579	Inverse variance weighted	11	0.093939893	0.267679599	0.725632201	-0.430712122	0.618591907	1.098493717	0.650046018	1.856312341
Myeloproliferative neoplasms	genus.Dorea.id.1997	Inverse variance weighted	10	-0.126087339	0.369627262	0.733013067	-0.850556772	0.598382094	0.881537851	0.427177026	1.819173167
Myeloproliferative neoplasms	family.Coriobacteriaceae.id.811	Inverse variance weighted	12	0.111177601	0.327482215	0.734239102	-0.530687541	0.753042743	1.117593375	0.588200419	2.123451314
Myeloproliferative neoplasms	order.Coriobacteriales.id.810	Inverse variance weighted	12	0.111177601	0.327482215	0.734239102	-0.530687541	0.753042743	1.117593375	0.588200419	2.123451314
Myeloproliferative neoplasms	class.Coriobacteriia.id.809	Inverse variance weighted	12	0.111177601	0.327482215	0.734239102	-0.530687541	0.753042743	1.117593375	0.588200419	2.123451314
Myeloproliferative neoplasms	family.ClostridialesvadinBB60group.id.11286	Inverse variance weighted	14	-0.079921934	0.251825751	0.75096288	-0.573500407	0.413656538	0.923188413	0.56354933	1.512337606
Myeloproliferative neoplasms	family.Enterobacteriaceae.id.3469	Inverse variance weighted	5	0.156154161	0.49606396	0.75292388	-0.816131201	1.128439522	1.169006404	0.442138897	3.090829564
Myeloproliferative neoplasms	order.Enterobacteriales.id.3468	Inverse variance weighted	5	0.156154161	0.49606396	0.75292388	-0.816131201	1.128439522	1.169006404	0.442138897	3.090829564
Myeloproliferative neoplasms	class.Alphaproteobacteria.id.2379	Inverse variance weighted	6	0.098697538	0.322663243	0.759692933	-0.533722419	0.731117496	1.103732412	0.586418009	2.077400797
Myeloproliferative neoplasms	genus.LachnospiraceaeNC2004group.id.11316	Inverse variance weighted	8	-0.06948203	0.232684684	0.765237451	-0.525544011	0.386579951	0.932876897	0.591233639	1.471938076
Myeloproliferative neoplasms	genus.FamilyXIIIAD3011group.id.11293	Inverse variance weighted	12	0.087585757	0.29597004	0.767285236	-0.492515522	0.667687035	1.091535867	0.611087256	1.949722462
Myeloproliferative neoplasms	genus.Coprobacter.id.949	Inverse variance weighted	10	0.075115951	0.25899426	0.771793743	-0.432512799	0.5827447	1.07800914	0.648876548	1.790947304
Myeloproliferative neoplasms	genus.RuminococcaceaeNK4A214group.id.11358	Inverse variance weighted	11	-0.086644871	0.324320148	0.789347158	-0.722312362	0.549022619	0.917002692	0.485628009	1.731559798
Myeloproliferative neoplasms	class.Betaproteobacteria.id.2867	Inverse variance weighted	10	0.100508754	0.39492304	0.79910766	-0.673540404	0.874557913	1.105733322	0.509900126	2.397815016
Myeloproliferative neoplasms	genus.RikenellaceaeRC9gutgroup.id.11191	Inverse variance weighted	10	0.039326525	0.160574389	0.806524905	-0.275399278	0.354052328	1.040110051	0.759268904	1.424829743
Myeloproliferative neoplasms	genus.Desulfovibrio.id.3173	Inverse variance weighted	8	0.07770531	0.319267624	0.807706291	-0.548059233	0.703469853	1.08080411	0.578070623	2.020752271
Myeloproliferative neoplasms	genus..Ruminococcusgauvreauiigroup.id.11342	Inverse variance weighted	9	-0.076769561	0.32804345	0.814967482	-0.719734723	0.566195601	0.92610324	0.486881397	1.761552639
Myeloproliferative neoplasms	genus.Flavonifractor.id.2059	Inverse variance weighted	4	0.099775228	0.433374812	0.817914226	-0.749639404	0.94918986	1.104922534	0.472536917	2.583615721
Myeloproliferative neoplasms	genus.Barnesiella.id.944	Inverse variance weighted	10	0.069950058	0.322368185	0.828217624	-0.561891585	0.701791701	1.072454619	0.570129595	2.017363984
Myeloproliferative neoplasms	genus.Terrisporobacter.id.11348	Inverse variance weighted	5	-0.069716716	0.324087692	0.829676235	-0.704928593	0.565495161	0.93265799	0.494143858	1.760319209
Myeloproliferative neoplasms	genus.Allisonella.id.2174	Inverse variance weighted	8	0.036105034	0.16836361	0.830198667	-0.293887643	0.36609771	1.036764736	0.745360233	1.442096143
Myeloproliferative neoplasms	class.Bacilli.id.1673	Inverse variance weighted	14	-0.058487013	0.279283387	0.834121816	-0.605882451	0.488908424	0.943190489	0.545592755	1.630535395
Myeloproliferative neoplasms	genus.Methanobrevibacter.id.123	Inverse variance weighted	6	-0.046956571	0.225702347	0.835192701	-0.489333171	0.395420029	0.954128833	0.613035047	1.485007806
Myeloproliferative neoplasms	phylum.Proteobacteria.id.2375	Inverse variance weighted	10	0.067760074	0.336589305	0.840453208	-0.591954963	0.727475112	1.070108531	0.553244654	2.06984787
Myeloproliferative neoplasms	order.Bacillales.id.1674	Inverse variance weighted	8	0.03367945	0.170105314	0.843051512	-0.299726966	0.367085867	1.034253024	0.741020517	1.443521864
Myeloproliferative neoplasms	genus.Marvinbryantia.id.2005	Inverse variance weighted	9	-0.074461602	0.392304627	0.849461494	-0.843378672	0.694455468	0.928243116	0.430254377	2.002618287
Myeloproliferative neoplasms	genus.Actinomyces.id.423	Inverse variance weighted	5	-0.105395244	0.571953818	0.853799846	-1.226424727	1.01563424	0.899968745	0.293339474	2.76111405
Myeloproliferative neoplasms	genus..Eubacteriumrectalegroup.id.14374	Inverse variance weighted	8	0.06908184	0.384291631	0.857337766	-0.684129757	0.822293438	1.071523899	0.504529101	2.275713063
Myeloproliferative neoplasms	genus.Senegalimassilia.id.11160	Inverse variance weighted	5	-0.087943551	0.496845636	0.859505427	-1.061760997	0.885873895	0.915812572	0.34584624	2.42510275
Myeloproliferative neoplasms	genus.Intestinibacter.id.11345	Inverse variance weighted	14	-0.041464824	0.25006237	0.868300237	-0.53158707	0.448657422	0.959383082	0.587671553	1.566208017
Myeloproliferative neoplasms	genus.Coprococcus2.id.11302	Inverse variance weighted	6	0.066972987	0.408271171	0.8696993	-0.733238509	0.867184483	1.069266594	0.480350848	2.380199917
Myeloproliferative neoplasms	genus.Peptococcus.id.2037	Inverse variance weighted	11	0.030017933	0.184816781	0.87097516	-0.332222958	0.392258823	1.030473013	0.717327371	1.480320804
Myeloproliferative neoplasms	family.Veillonellaceae.id.2172	Inverse variance weighted	18	0.046447171	0.289593886	0.872576022	-0.521156846	0.614051187	1.047542737	0.593833177	1.847902454
Myeloproliferative neoplasms	genus.Subdoligranulum.id.2070	Inverse variance weighted	10	0.055679589	0.356490765	0.875884813	-0.643042309	0.754401488	1.057258873	0.525690675	2.126338503
Myeloproliferative neoplasms	family.Rhodospirillaceae.id.2717	Inverse variance weighted	13	0.047768908	0.317748344	0.880499797	-0.575017846	0.670555662	1.048928229	0.562694827	1.955323518
Myeloproliferative neoplasms	phylum.Verrucomicrobia.id.3982	Inverse variance weighted	12	0.038348063	0.257175334	0.881464845	-0.465715591	0.542411717	1.039092839	0.627685778	1.720150379
Myeloproliferative neoplasms	genus.Parabacteroides.id.954	Inverse variance weighted	5	0.070218347	0.471201227	0.881537952	-0.853336059	0.993772752	1.072742385	0.425991426	2.701407009
Myeloproliferative neoplasms	genus.Oscillibacter.id.2063	Inverse variance weighted	13	0.039960485	0.268679553	0.88176735	-0.486651439	0.566572408	1.040769647	0.614681249	1.762216529
Myeloproliferative neoplasms	genus.Clostridiumsensustricto1.id.1873	Inverse variance weighted	5	-0.060277523	0.422395228	0.886523894	-0.888172169	0.767617124	0.941503209	0.411407049	2.154625925
Myeloproliferative neoplasms	genus.ErysipelotrichaceaeUCG003.id.11384	Inverse variance weighted	12	0.037449307	0.27616701	0.892134469	-0.503838033	0.578736647	1.038159369	0.604207237	1.783783458
Myeloproliferative neoplasms	genus.Howardella.id.2000	Inverse variance weighted	9	0.02652761	0.208963028	0.898980907	-0.383039925	0.436095145	1.026882599	0.681785678	1.546655944
Myeloproliferative neoplasms	order.Gastranaerophilales.id.1591	Inverse variance weighted	9	-0.044621906	0.408041942	0.912919994	-0.844384112	0.755140301	0.956359008	0.429821999	2.127910051
Myeloproliferative neoplasms	genus.Anaerotruncus.id.2054	Inverse variance weighted	13	-0.033398192	0.335207056	0.920634492	-0.690404021	0.623607637	0.96715337	0.501373463	1.865646491
Myeloproliferative neoplasms	genus.Coprococcus1.id.11301	Inverse variance weighted	9	-0.039786205	0.439504794	0.927869967	-0.901215602	0.821643191	0.960994873	0.406075733	2.27423377
Myeloproliferative neoplasms	family.Defluviitaleaceae.id.1924	Inverse variance weighted	10	0.023285444	0.269516494	0.93115075	-0.504966884	0.551537773	1.023558667	0.603525561	1.735920418
Myeloproliferative neoplasms	family.Prevotellaceae.id.960	Inverse variance weighted	13	-0.0291277	0.370027574	0.937257208	-0.754381744	0.696126344	0.971292422	0.470301291	2.005967212
Myeloproliferative neoplasms	genus..Clostridiuminnocuumgroup.id.14397	Inverse variance weighted	6	0.014515247	0.210522286	0.94503042	-0.398108433	0.427138927	1.014621104	0.671589201	1.532865603
Myeloproliferative neoplasms	genus..Eubacteriumnodatumgroup.id.11297	Inverse variance weighted	11	0.010014576	0.151737898	0.947378481	-0.287391703	0.307420856	1.01006489	0.750217808	1.359913175
Myeloproliferative neoplasms	genus.Adlercreutzia.id.812	Inverse variance weighted	7	0.018356842	0.299035107	0.951051075	-0.567751968	0.604465652	1.018526364	0.566798188	1.830273944
Myeloproliferative neoplasms	class.Melainabacteria.id.1589	Inverse variance weighted	9	0.025011621	0.414400864	0.95187202	-0.787214072	0.837237314	1.025327036	0.455110937	2.309976413
Myeloproliferative neoplasms	genus.Bacteroides.id.918	Inverse variance weighted	7	-0.022790865	0.407212138	0.955367267	-0.820926656	0.775344925	0.977466885	0.440023715	2.171340949
Myeloproliferative neoplasms	family.Bacteroidaceae.id.917	Inverse variance weighted	7	-0.022790865	0.407212138	0.955367267	-0.820926656	0.775344925	0.977466885	0.440023715	2.171340949
Myeloproliferative neoplasms	family.BacteroidalesS24.7group.id.11173	Inverse variance weighted	7	-0.016328396	0.307692421	0.957678312	-0.619405541	0.58674875	0.98380419	0.538264318	1.798132722
Myeloproliferative neoplasms	genus.CandidatusSoleaferrea.id.11350	Inverse variance weighted	8	-0.010054251	0.277352063	0.971082331	-0.553664296	0.533555793	0.989996124	0.574839564	1.704984113
Myeloproliferative neoplasms	genus.Erysipelatoclostridium.id.11381	Inverse variance weighted	14	-0.008207998	0.255772332	0.974399457	-0.509521769	0.493105774	0.991825596	0.600782823	1.637393706
Myeloproliferative neoplasms	genus.ChristensenellaceaeR.7group.id.11283	Inverse variance weighted	6	0.014595093	0.488519691	0.976165817	-0.9429035	0.972093687	1.014702122	0.389495292	2.643473275
Myeloproliferative neoplasms	class.Mollicutes.id.3920	Inverse variance weighted	10	-0.00657102	0.289518361	0.98189246	-0.574027007	0.560884967	0.993450522	0.563252643	1.752222473
Myeloproliferative neoplasms	phylum.Tenericutes.id.3919	Inverse variance weighted	10	-0.00657102	0.289518361	0.98189246	-0.574027007	0.560884967	0.993450522	0.563252643	1.752222473
Myeloproliferative neoplasms	genus.Butyrivibrio.id.1993	Inverse variance weighted	13	-0.002205532	0.140972549	0.98751751	-0.278511728	0.274100663	0.997796898	0.756909391	1.315347202
Myeloproliferative neoplasms	genus.RuminococcaceaeUCG003.id.11361	Inverse variance weighted	14	0.004525043	0.304751652	0.988153208	-0.592788196	0.601838282	1.004535297	0.552783864	1.825471449
Myeloproliferative neoplasms	genus..Eubacteriumhalliigroup.id.11338	MR Egger	13	-1.709136319	0.640666064	0.021881794	-2.964841805	-0.453430834	0.18102207	0.051568627	0.635444303
Myeloproliferative neoplasms	family.Christensenellaceae.id.1866	MR Egger	10	1.64347741	0.69164719	0.044812146	0.287848918	2.999105903	5.173127357	1.333555812	20.06758653
Myeloproliferative neoplasms	genus.Akkermansia.id.4037	MR Egger	10	2.161269722	0.925464566	0.047762254	0.347359172	3.975180272	8.682154582	1.415324978	53.25971726
Myeloproliferative neoplasms	family.Verrucomicrobiaceae.id.4036	MR Egger	10	2.15278999	0.925078186	0.048376907	0.339636745	3.965943235	8.608843509	1.404437329	52.77002046
Myeloproliferative neoplasms	order.Verrucomicrobiales.id.4030	MR Egger	10	2.152169512	0.925073549	0.048426706	0.339025356	3.965313668	8.603503567	1.403578934	52.73680864
Myeloproliferative neoplasms	class.Verrucomicrobiae.id.4029	MR Egger	10	2.152169512	0.925073549	0.048426706	0.339025356	3.965313668	8.603503567	1.403578934	52.73680864
Myeloproliferative neoplasms	genus.Eisenbergiella.id.11304	MR Egger	11	-2.668661256	1.430270926	0.094925525	-5.47199227	0.134669759	0.069344998	0.004202851	1.144158873
Myeloproliferative neoplasms	class.Gammaproteobacteria.id.3303	MR Egger	6	-3.064342826	1.411585135	0.095718781	-5.83104969	-0.297635962	0.046684512	0.002934995	0.742571615
Myeloproliferative neoplasms	genus.Collinsella.id.815	MR Egger	9	-2.457401074	1.366086138	0.115075873	-5.134929904	0.220127756	0.085657279	0.005887464	1.246235935
Myeloproliferative neoplasms	genus..Ruminococcusgauvreauiigroup.id.11342	MR Egger	9	3.66797299	2.045345965	0.116011832	-0.340905101	7.67685108	39.17242242	0.71112639	2157.814279
Myeloproliferative neoplasms	genus.Ruminiclostridium5.id.11355	MR Egger	10	2.484478721	1.425620449	0.119546338	-0.309737359	5.278694801	11.99486595	0.733639615	196.1137407
Myeloproliferative neoplasms	genus..Eubacteriumnodatumgroup.id.11297	MR Egger	11	-1.153684672	0.676031583	0.1220896	-2.478706575	0.171337232	0.315472214	0.083851611	1.186890939
Myeloproliferative neoplasms	genus.RuminococcaceaeUCG002.id.11360	MR Egger	17	1.364874283	0.873248448	0.138904104	-0.346692675	3.076441241	3.91523081	0.707022581	21.6811071
Myeloproliferative neoplasms	family.Defluviitaleaceae.id.1924	MR Egger	10	1.475994117	0.90984534	0.143407511	-0.30730275	3.259290984	4.375383254	0.735427916	26.03107415
Myeloproliferative neoplasms	genus.Dorea.id.1997	MR Egger	10	-1.570643065	1.001145811	0.155322258	-3.532888854	0.391602725	0.207911439	0.02922038	1.479349886
Myeloproliferative neoplasms	order.MollicutesRF9.id.11579	MR Egger	11	-1.919141072	1.268298663	0.164532829	-4.405006453	0.566724308	0.146732941	0.012216028	1.762484229
Myeloproliferative neoplasms	genus.LachnospiraceaeUCG001.id.11321	MR Egger	11	-1.517138221	1.027510427	0.173916205	-3.531058658	0.496782216	0.219338688	0.029273908	1.643424568
Myeloproliferative neoplasms	order.Rhodospirillales.id.2667	MR Egger	12	2.201834858	1.586874651	0.195420957	-0.908439458	5.312109175	9.041588321	0.40315287	202.7774708
Myeloproliferative neoplasms	genus.Odoribacter.id.952	MR Egger	5	-3.092509169	1.90790655	0.203487074	-6.832006008	0.64698767	0.045387925	0.001078692	1.90977927
Myeloproliferative neoplasms	genus.DefluviitaleaceaeUCG011.id.11287	MR Egger	8	1.593511528	1.121299966	0.205105585	-0.604236404	3.791259461	4.920998851	0.546491566	44.3121746
Myeloproliferative neoplasms	order.Selenomonadales.id.2165	MR Egger	12	-1.512382295	1.116903986	0.205516657	-3.701514107	0.676749517	0.220384332	0.024686121	1.967472093
Myeloproliferative neoplasms	class.Negativicutes.id.2164	MR Egger	12	-1.512382295	1.116903986	0.205516657	-3.701514107	0.676749517	0.220384332	0.024686121	1.967472093
Myeloproliferative neoplasms	genus.Coprococcus2.id.11302	MR Egger	6	-5.693571431	3.831293993	0.211452161	-13.20290766	1.815764795	0.003367545	1.84523E-06	6.145774639
Myeloproliferative neoplasms	genus.Fusicatenibacter.id.11305	MR Egger	18	1.606224957	1.237527363	0.212710996	-0.819328675	4.03177859	4.983961005	0.440727427	56.36106537
Myeloproliferative neoplasms	genus.Clostridiumsensustricto1.id.1873	MR Egger	5	2.086854225	1.334211317	0.215746332	-0.528199957	4.701908407	8.059521804	0.589665438	110.1571968
Myeloproliferative neoplasms	genus.Oscillibacter.id.2063	MR Egger	13	-1.294531988	0.986019925	0.215951149	-3.227131041	0.638067065	0.274026082	0.039671151	1.892818646
Myeloproliferative neoplasms	genus.Anaerostipes.id.1991	MR Egger	12	-1.511340134	1.153186934	0.219301111	-3.771586525	0.748906257	0.220614127	0.02301552	2.114685828
Myeloproliferative neoplasms	family.Enterobacteriaceae.id.3469	MR Egger	5	4.188051516	2.873634249	0.241060479	-1.444271612	9.820374643	65.89427183	0.235917854	18404.94474
Myeloproliferative neoplasms	order.Enterobacteriales.id.3468	MR Egger	5	4.188051516	2.873634249	0.241060479	-1.444271612	9.820374643	65.89427183	0.235917854	18404.94474
Myeloproliferative neoplasms	class.Melainabacteria.id.1589	MR Egger	9	-1.481319523	1.157022562	0.241234663	-3.749083743	0.786444698	0.227337513	0.023539304	2.195576596
Myeloproliferative neoplasms	phylum.Verrucomicrobia.id.3982	MR Egger	12	0.835318383	0.673524057	0.243205322	-0.484788768	2.155425535	2.30554798	0.615827265	8.631562428
Myeloproliferative neoplasms	genus.RikenellaceaeRC9gutgroup.id.11191	MR Egger	10	1.248743532	0.999657439	0.246913129	-0.710585048	3.208072112	3.485960208	0.491356646	24.73136095
Myeloproliferative neoplasms	genus..Eubacteriumcoprostanoligenesgroup.id.11375	MR Egger	12	-1.710043364	1.407232708	0.252208174	-4.468219473	1.048132744	0.18085795	0.011467716	2.852320129
Myeloproliferative neoplasms	order.Bifidobacteriales.id.432	MR Egger	9	1.130937571	0.917376097	0.257444551	-0.667119579	2.928994721	3.098560259	0.513184639	18.70881346
Myeloproliferative neoplasms	genus.Faecalibacterium.id.2057	MR Egger	10	-0.791950313	0.649612357	0.257525072	-2.065190532	0.481289906	0.452960519	0.12679413	1.618160332
Myeloproliferative neoplasms	family.Bifidobacteriaceae.id.433	MR Egger	10	1.101745296	0.915634686	0.263272696	-0.692898689	2.896389281	3.00941376	0.500124261	18.10864196
Myeloproliferative neoplasms	genus.Intestinimonas.id.2062	MR Egger	14	0.999399243	0.86486016	0.270350889	-0.695726671	2.694525157	2.716649292	0.498711917	14.79849012
Myeloproliferative neoplasms	genus.Enterorhabdus.id.820	MR Egger	6	1.025975901	0.809444358	0.273738526	-0.560535041	2.612486843	2.789816717	0.570903525	13.63291164
Myeloproliferative neoplasms	genus.Sutterella.id.2896	MR Egger	12	-1.392984984	1.214430178	0.278072992	-3.773268133	0.987298165	0.248332927	0.022976849	2.683973016
Myeloproliferative neoplasms	genus.Turicibacter.id.2162	MR Egger	8	1.260610296	1.079161422	0.28706226	-0.854546091	3.375766683	3.527573694	0.425476275	29.24669813
Myeloproliferative neoplasms	genus..Eubacteriumventriosumgroup.id.11341	MR Egger	13	-1.894141206	1.694926687	0.287579028	-5.216197513	1.427915101	0.150447483	0.00542793	4.169996102
Myeloproliferative neoplasms	genus.LachnospiraceaeUCG008.id.11328	MR Egger	10	1.532128822	1.368518355	0.295388545	-1.150167153	4.214424797	4.628018569	0.316583847	67.65523917
Myeloproliferative neoplasms	order.Gastranaerophilales.id.1591	MR Egger	9	-1.344977667	1.193970036	0.297095429	-3.685158936	0.995203603	0.260545527	0.025093186	2.705275087
Myeloproliferative neoplasms	genus.Prevotella9.id.11183	MR Egger	13	1.245978938	1.141920852	0.298540958	-0.992185932	3.484143808	3.476336252	0.370765337	32.594508
Myeloproliferative neoplasms	genus.Phascolarctobacterium.id.2168	MR Egger	7	2.252793087	1.954795572	0.301242197	-1.578606234	6.084192408	9.514272953	0.206262379	438.8652457
Myeloproliferative neoplasms	genus.Butyricicoccus.id.2055	MR Egger	7	1.013489226	0.89048141	0.306642511	-0.731854337	2.758832789	2.75519777	0.481016196	15.78141195
Myeloproliferative neoplasms	genus.Alistipes.id.968	MR Egger	12	1.755159907	1.657983749	0.314677732	-1.494488242	5.004808056	5.784372629	0.224363395	149.128456
Myeloproliferative neoplasms	genus.Holdemania.id.2157	MR Egger	12	-0.707799143	0.673704394	0.318164815	-2.028259754	0.612661469	0.492727428	0.131564276	1.845336174
Myeloproliferative neoplasms	genus.LachnospiraceaeNC2004group.id.11316	MR Egger	8	-1.058059384	0.983779582	0.323483085	-2.986267365	0.870148597	0.347128801	0.050475492	2.387265568
Myeloproliferative neoplasms	genus.Blautia.id.1992	MR Egger	11	-1.06913072	1.036435831	0.329207805	-3.100544948	0.962283508	0.343306818	0.04502466	2.617667119
Myeloproliferative neoplasms	genus.RuminococcaceaeUCG004.id.11362	MR Egger	9	-1.635675664	1.562332305	0.329923372	-4.697846982	1.426495655	0.194820694	0.00911488	4.164081215
Myeloproliferative neoplasms	genus.Butyrivibrio.id.1993	MR Egger	13	0.770220096	0.770200048	0.338788651	-0.739371998	2.279812191	2.160241662	0.477413638	9.77484443
Myeloproliferative neoplasms	genus.Marvinbryantia.id.2005	MR Egger	9	1.706056404	1.671927124	0.341497068	-1.570920759	4.983033567	5.507200423	0.207853711	145.916358
Myeloproliferative neoplasms	phylum.Bacteroidetes.id.905	MR Egger	10	1.413999541	1.41340209	0.346401127	-1.356268557	4.184267638	4.112370147	0.257620281	65.64540709
Myeloproliferative neoplasms	family.Porphyromonadaceae.id.943	MR Egger	9	-2.116218584	2.099711308	0.34708223	-6.231652748	1.999215581	0.120486377	0.0019662	7.383262254
Myeloproliferative neoplasms	class.Actinobacteria.id.419	MR Egger	12	0.872583803	0.896944128	0.353576228	-0.885426689	2.630594295	2.393086135	0.41253811	13.88201746
Myeloproliferative neoplasms	genus.Intestinibacter.id.11345	MR Egger	14	-0.826018104	0.861830687	0.356751037	-2.515206251	0.863170042	0.437789051	0.080846236	2.3706639
Myeloproliferative neoplasms	order.Desulfovibrionales.id.3156	MR Egger	10	2.206491255	2.25688595	0.356871809	-2.217005208	6.629987717	9.083787713	0.108934858	757.4728666
Myeloproliferative neoplasms	phylum.Firmicutes.id.1672	MR Egger	13	-0.708713894	0.739096782	0.358215505	-2.157343586	0.739915797	0.492276911	0.11563188	2.095759039
Myeloproliferative neoplasms	genus..Ruminococcusgnavusgroup.id.14376	MR Egger	11	0.9431322	0.974365992	0.358362106	-0.966625144	2.852889545	2.568012363	0.38036455	17.3378079
Myeloproliferative neoplasms	family.Veillonellaceae.id.2172	MR Egger	18	-0.606518526	0.641667491	0.358601981	-1.864186809	0.651149757	0.545245828	0.155022221	1.917744501
Myeloproliferative neoplasms	genus.LachnospiraceaeNK4A136group.id.11319	MR Egger	13	-0.582421034	0.612680151	0.362221026	-1.78327413	0.618432062	0.558544473	0.168086907	1.856015642
Myeloproliferative neoplasms	order.Actinomycetales.id.420	MR Egger	3	-4.145868058	2.703195471	0.367835759	-9.44413118	1.152395065	0.015829689	7.91527E-05	3.165766053
Myeloproliferative neoplasms	family.Actinomycetaceae.id.421	MR Egger	3	-4.085155092	2.682923147	0.36994315	-9.34368446	1.173374277	0.01682053	8.75164E-05	3.232882897
Myeloproliferative neoplasms	genus.Bacteroides.id.918	MR Egger	7	-1.989370866	2.021468838	0.370249339	-5.951449789	1.972708056	0.136781452	0.002602065	7.190121401
Myeloproliferative neoplasms	family.Bacteroidaceae.id.917	MR Egger	7	-1.989370866	2.021468838	0.370249339	-5.951449789	1.972708056	0.136781452	0.002602065	7.190121401
Myeloproliferative neoplasms	order.Bacteroidales.id.913	MR Egger	12	1.110938729	1.190694742	0.372790686	-1.222822966	3.444700424	3.037208172	0.294397917	31.33389515
Myeloproliferative neoplasms	class.Bacteroidia.id.912	MR Egger	12	1.110938729	1.190694742	0.372790686	-1.222822966	3.444700424	3.037208172	0.294397917	31.33389515
Myeloproliferative neoplasms	genus.Lactococcus.id.1851	MR Egger	9	-1.195551768	1.27473947	0.379529672	-3.694041129	1.302937592	0.302536978	0.024871291	3.680091412

Myeloproliferative neoplasms	genus.CandidatusSoleaferrea.id.11350	MR Egger	8	2.818169384	2.990988192	0.382452604	-3.044167472	8.680506239	16.74616679	0.047635954	5887.026081
Myeloproliferative neoplasms	genus.RuminococcaceaeUCG005.id.11363	MR Egger	13	-0.74113408	0.814863522	0.382572337	-2.338266584	0.855998424	0.476573137	0.096494759	2.353723221
Myeloproliferative neoplasms	family.Victivallaceae.id.2255	MR Egger	9	0.678998414	0.733348093	0.385313056	-0.758363848	2.116360676	1.971901713	0.468432226	8.300872885
Myeloproliferative neoplasms	genus.RuminococcaceaeUCG014.id.11371	MR Egger	10	1.391433819	1.526708463	0.38872439	-1.600914769	4.383782407	4.020610751	0.201711914	80.14058518
Myeloproliferative neoplasms	order.Lactobacillales.id.1800	MR Egger	11	-0.694240228	0.767303651	0.389160112	-2.198155384	0.809674927	0.499453775	0.111007736	2.247177371
Myeloproliferative neoplasms	genus.RuminococcaceaeNK4A214group.id.11358	MR Egger	11	1.184505873	1.314602871	0.391042493	-1.392115753	3.7611275	3.269071087	0.24854888	42.99687762
Myeloproliferative neoplasms	genus.Streptococcus.id.1853	MR Egger	10	-1.108751787	1.22429422	0.39159272	-3.508368458	1.290864885	0.329970578	0.029945732	3.635929856
Myeloproliferative neoplasms	class.Deltaproteobacteria.id.3087	MR Egger	11	2.362386706	2.661752723	0.397890811	-2.854648632	7.579422044	10.61625913	0.057576048	1957.497292
Myeloproliferative neoplasms	family.Rhodospirillaceae.id.2717	MR Egger	13	1.703124966	1.951277455	0.401409714	-2.121378846	5.527628777	5.491080044	0.119866237	251.5467299
Myeloproliferative neoplasms	genus.Ruminiclostridium9.id.11357	MR Egger	7	-1.71855924	1.943808899	0.417112369	-5.528424682	2.091306202	0.179324325	0.003972242	8.095482595
Myeloproliferative neoplasms	genus..Eubacteriumxylanophilumgroup.id.14375	MR Egger	7	1.401853664	1.58836726	0.417871152	-1.711346166	4.515053493	4.062723915	0.180622481	91.38245421
Myeloproliferative neoplasms	genus.Ruminiclostridium6.id.11356	MR Egger	12	-0.620137876	0.736566905	0.419506181	-2.063809009	0.823533257	0.537870273	0.12696942	2.278536285
Myeloproliferative neoplasms	genus.Methanobrevibacter.id.123	MR Egger	6	-0.741897631	0.84425472	0.429151493	-2.396636882	0.912841619	0.476209388	0.091023562	2.491392071
Myeloproliferative neoplasms	family.Pasteurellaceae.id.3689	MR Egger	10	0.512022601	0.627125739	0.437871769	-0.717143847	1.74118905	1.668662824	0.488144482	5.704121877
Myeloproliferative neoplasms	order.Pasteurellales.id.3688	MR Egger	10	0.512022601	0.627125739	0.437871769	-0.717143847	1.74118905	1.668662824	0.488144482	5.704121877
Myeloproliferative neoplasms	order.Victivallales.id.2254	MR Egger	8	-0.603874657	0.73545686	0.4429898	-2.045370102	0.837620787	0.546689293	0.129332315	2.310862398
Myeloproliferative neoplasms	class.Lentisphaeria.id.2250	MR Egger	8	-0.603874657	0.73545686	0.4429898	-2.045370102	0.837620787	0.546689293	0.129332315	2.310862398
Myeloproliferative neoplasms	genus.ChristensenellaceaeR.7group.id.11283	MR Egger	6	1.350171947	1.600692603	0.446441576	-1.787185556	4.487529449	3.858088859	0.167430732	88.90153857
Myeloproliferative neoplasms	genus.Allisonella.id.2174	MR Egger	8	0.936102858	1.174632471	0.455859303	-1.366176784	3.238382501	2.550024223	0.255080325	25.49245435
Myeloproliferative neoplasms	genus.FamilyXIIIAD3011group.id.11293	MR Egger	12	-1.202792277	1.585860252	0.465682547	-4.311078371	1.905493817	0.300354367	0.013419071	6.722726603
Myeloproliferative neoplasms	genus.Coprococcus3.id.11303	MR Egger	5	-2.492549831	3.050584486	0.473737871	-8.471695423	3.486595761	0.082698829	0.00020931	32.67452628
Myeloproliferative neoplasms	class.Mollicutes.id.3920	MR Egger	10	1.325305027	1.771474167	0.475793325	-2.14678434	4.797394395	3.763333099	0.116859334	121.1942215
Myeloproliferative neoplasms	phylum.Tenericutes.id.3919	MR Egger	10	1.325305027	1.771474167	0.475793325	-2.14678434	4.797394395	3.763333099	0.116859334	121.1942215
Myeloproliferative neoplasms	genus.LachnospiraceaeFCS020group.id.11314	MR Egger	11	-0.53494745	0.721413793	0.477277009	-1.948918485	0.879023585	0.585700068	0.142428026	2.408546818
Myeloproliferative neoplasms	phylum.Euryarchaeota.id.55	MR Egger	11	-0.519148773	0.701502447	0.478127793	-1.89409357	0.855796024	0.595026835	0.15045465	2.353246875
Myeloproliferative neoplasms	family.Peptococcaceae.id.2024	MR Egger	8	-0.680838967	0.90790504	0.481673153	-2.460332845	1.098654912	0.506192136	0.085406519	3.000127873
Myeloproliferative neoplasms	family.Clostridiaceae1.id.1869	MR Egger	7	1.464862641	1.938483372	0.483902482	-2.334564768	5.264290049	4.326948854	0.096852627	193.3090202
Myeloproliferative neoplasms	genus.Bifidobacterium.id.436	MR Egger	12	0.448230299	0.621121233	0.487041921	-0.769167318	1.665627917	1.565539197	0.463398772	5.288993254
Myeloproliferative neoplasms	genus.Bilophila.id.3170	MR Egger	13	0.995424302	1.384193925	0.487053539	-1.717595791	3.708444396	2.705872205	0.179497178	40.79030357
Myeloproliferative neoplasms	genus.Veillonella.id.2198	MR Egger	6	2.271289738	2.97621512	0.487901506	-3.562091898	8.104671374	9.691892757	0.028379396	3309.893769
Myeloproliferative neoplasms	genus.LachnospiraceaeUCG004.id.11324	MR Egger	12	-1.16440759	1.61691253	0.487927974	-4.333556148	2.004740968	0.312107503	0.013120805	7.424170547
Myeloproliferative neoplasms	genus.Oscillospira.id.2064	MR Egger	7	1.054326391	1.422626691	0.491942398	-1.734021922	3.842674705	2.870041218	0.176572818	46.65008293
Myeloproliferative neoplasms	genus.RuminococcaceaeUCG013.id.11370	MR Egger	11	-0.848010748	1.208205536	0.500502773	-3.216093598	1.520072102	0.428266014	0.040111444	4.572554873
Myeloproliferative neoplasms	family.Peptostreptococcaceae.id.2042	MR Egger	12	0.407808595	0.595812495	0.509237467	-0.759983894	1.575601085	1.503519353	0.467673959	4.833646176
Myeloproliferative neoplasms	genus.Slackia.id.825	MR Egger	6	-1.326990716	1.841538399	0.511026409	-4.936405979	2.282424547	0.265274347	0.007180358	9.800413187
Myeloproliferative neoplasms	family.Erysipelotrichaceae.id.2149	MR Egger	11	1.655783406	2.470610686	0.519559666	-3.186613539	6.498180352	5.237181155	0.041311534	663.9324096
Myeloproliferative neoplasms	order.Erysipelotrichales.id.2148	MR Egger	11	1.655783406	2.470610686	0.519559666	-3.186613539	6.498180352	5.237181155	0.041311534	663.9324096
Myeloproliferative neoplasms	class.Erysipelotrichia.id.2147	MR Egger	11	1.655783406	2.470610686	0.519559666	-3.186613539	6.498180352	5.237181155	0.041311534	663.9324096
Myeloproliferative neoplasms	phylum.Lentisphaerae.id.2238	MR Egger	9	-0.4979654	0.737447022	0.521186565	-1.943361563	0.947430762	0.607765963	0.143221691	2.579074883
Myeloproliferative neoplasms	genus.Roseburia.id.2012	MR Egger	11	1.132879872	1.739594719	0.531170995	-2.276725776	4.542485521	3.104584445	0.102619657	93.92396023
Myeloproliferative neoplasms	genus.Desulfovibrio.id.3173	MR Egger	8	-0.63744944	0.96235741	0.53232874	-2.523669964	1.248771085	0.528639032	0.080164864	3.486056256
Myeloproliferative neoplasms	genus.Lactobacillus.id.1837	MR Egger	7	-0.56141482	0.84351201	0.535155406	-2.21469836	1.091868719	0.570401477	0.109186444	2.979837351
Myeloproliferative neoplasms	genus.Anaerotruncus.id.2054	MR Egger	13	0.642795872	1.011768905	0.53821207	-1.340271183	2.625862926	1.901790616	0.26177467	13.81649166
Myeloproliferative neoplasms	family.Alcaligenaceae.id.2875	MR Egger	11	0.996673457	1.561340944	0.539151066	-2.063554792	4.056901707	2.709254371	0.127001702	57.79496765
Myeloproliferative neoplasms	genus.Peptococcus.id.2037	MR Egger	11	-0.430028281	0.703006288	0.555871643	-1.807920607	0.947864044	0.650490698	0.163994792	2.580192592
Myeloproliferative neoplasms	genus..Clostridiuminnocuumgroup.id.14397	MR Egger	6	-0.645896779	1.012990165	0.558395063	-2.631357501	1.339563944	0.524192247	0.071980682	3.817378551
Myeloproliferative neoplasms	family.Methanobacteriaceae.id.121	MR Egger	8	-0.442290722	0.717858079	0.560443789	-1.849292557	0.964711113	0.642562801	0.157348442	2.624029499
Myeloproliferative neoplasms	order.Methanobacteriales.id.120	MR Egger	8	-0.442290722	0.717858079	0.560443789	-1.849292557	0.964711113	0.642562801	0.157348442	2.624029499
Myeloproliferative neoplasms	class.Methanobacteria.id.119	MR Egger	8	-0.442290722	0.717858079	0.560443789	-1.849292557	0.964711113	0.642562801	0.157348442	2.624029499
Myeloproliferative neoplasms	class.Bacilli.id.1673	MR Egger	14	-0.4347571	0.731161939	0.563147093	-1.8678345	0.998320301	0.647421907	0.154457778	2.713719767
Myeloproliferative neoplasms	genus..Eubacteriumfissicatenagroup.id.14373	MR Egger	8	0.565444496	0.966404995	0.579802485	-1.328709294	2.459598287	1.760230025	0.264818844	11.70011051
Myeloproliferative neoplasms	genus.RuminococcaceaeUCG010.id.11367	MR Egger	6	0.657499041	1.09817017	0.581614003	-1.494914492	2.809912574	1.929959544	0.224267781	16.60846615
Myeloproliferative neoplasms	class.Betaproteobacteria.id.2867	MR Egger	10	0.744843437	1.307538014	0.584549668	-1.81793107	3.307617945	2.106111671	0.162361318	27.31997023
Myeloproliferative neoplasms	genus.Romboutsia.id.11347	MR Egger	11	0.586284193	1.040609691	0.586920047	-1.453310801	2.625879188	1.797297582	0.233794957	13.81671634
Myeloproliferative neoplasms	phylum.Cyanobacteria.id.1500	MR Egger	7	-1.10567172	1.931690453	0.591835235	-4.891785008	2.680441567	0.330988476	0.007508009	14.59153502
Myeloproliferative neoplasms	genus.Haemophilus.id.3698	MR Egger	8	0.374657509	0.680270033	0.60169439	-0.958671756	1.707986774	1.454493179	0.383401799	5.517841626
Myeloproliferative neoplasms	family.Coriobacteriaceae.id.811	MR Egger	12	-0.704663	1.323718678	0.606118236	-3.299151608	1.889825608	0.494275117	0.036914472	6.618214418
Myeloproliferative neoplasms	order.Coriobacteriales.id.810	MR Egger	12	-0.704663	1.323718678	0.606118236	-3.299151608	1.889825608	0.494275117	0.036914472	6.618214418
Myeloproliferative neoplasms	class.Coriobacteriia.id.809	MR Egger	12	-0.704663	1.323718678	0.606118236	-3.299151608	1.889825608	0.494275117	0.036914472	6.618214418
Myeloproliferative neoplasms	family.FamilyXI.id.1936	MR Egger	8	-0.69241345	1.30567808	0.614940933	-3.251542488	1.866715588	0.500367	0.038714445	6.467021117
Myeloproliferative neoplasms	family.Streptococcaceae.id.1850	MR Egger	9	1.005792192	1.919302182	0.616428265	-2.756040084	4.767624468	2.734072325	0.063542895	117.6394535
Myeloproliferative neoplasms	genus.Paraprevotella.id.962	MR Egger	13	-0.390538989	0.777195889	0.625225081	-1.913842932	1.132764953	0.676692046	0.147512416	3.104227688
Myeloproliferative neoplasms	class.Alphaproteobacteria.id.2379	MR Egger	6	0.638592679	1.212030954	0.626144689	-1.73698799	3.014173349	1.8938138	0.176049867	20.37224325
Myeloproliferative neoplasms	genus.Actinomyces.id.423	MR Egger	5	-1.917671095	3.558196355	0.627350718	-8.891735951	5.05639376	0.146948794	0.000137521	157.0232307
Myeloproliferative neoplasms	genus.LachnospiraceaeUCG010.id.11330	MR Egger	10	0.60597848	1.279639371	0.648471324	-1.902114687	3.114071648	1.83304493	0.149252663	22.51252111
Myeloproliferative neoplasms	genus.RuminococcaceaeUCG003.id.11361	MR Egger	14	-0.523371819	1.119868703	0.648621158	-2.718314477	1.671570839	0.592519308	0.065985881	5.320518914
Myeloproliferative neoplasms	family.Desulfovibrionaceae.id.3169	MR Egger	8	0.981766362	2.139486678	0.662474143	-3.211627526	5.17516025	2.669166794	0.040290985	176.8249481
Myeloproliferative neoplasms	genus..Eubacteriumruminantiumgroup.id.11340	MR Egger	16	-0.333721553	0.749488659	0.662935182	-1.802719325	1.13527622	0.716253193	0.164849997	3.11203303
Myeloproliferative neoplasms	genus..Ruminococcustorquesgroup.id.14377	MR Egger	10	-0.587353795	1.307189362	0.665120146	-3.149444944	1.974737354	0.555796091	0.042875919	7.204727114
Myeloproliferative neoplasms	genus.Anaerofilum.id.2053	MR Egger	9	0.784533497	1.754041856	0.668190985	-2.653388541	4.222455536	2.191384415	0.070412214	68.20074822
Myeloproliferative neoplasms	genus.FamilyXIIIUCG001.id.11294	MR Egger	7	0.571053072	1.291258827	0.676786053	-1.959814228	3.101920372	1.770130145	0.140884591	22.24062057
Myeloproliferative neoplasms	family.Lactobacillaceae.id.1836	MR Egger	7	-0.34840459	0.791086213	0.678026494	-1.898933567	1.202124388	0.705813254	0.149728209	3.327177635
Myeloproliferative neoplasms	family.Oxalobacteraceae.id.2966	MR Egger	14	0.231934823	0.608056986	0.709547662	-0.959856869	1.423726515	1.261037536	0.382947694	4.152566242
Myeloproliferative neoplasms	family.FamilyXIII.id.1957	MR Egger	5	-2.521724188	6.287706301	0.715235495	-14.84562854	9.802180162	0.080320999	3.56965E-07	18073.10431
Myeloproliferative neoplasms	genus..Eubacteriumoxidoreducensgroup.id.11339	MR Egger	3	-0.577304994	1.219832413	0.718593399	-2.968176524	1.813566535	0.561409331	0.051396946	6.132279467
Myeloproliferative neoplasms	genus.Lachnospira.id.2004	MR Egger	5	1.097969373	2.810952888	0.722175205	-4.411498287	6.607437032	2.998071871	0.01213698	740.5824953
Myeloproliferative neoplasms	genus.Alloprevotella.id.961	MR Egger	5	0.693352143	1.832094544	0.730291462	-2.897553163	4.284257449	2.000409967	0.055158018	72.54865564
Myeloproliferative neoplasms	order.Burkholderiales.id.2874	MR Egger	10	0.381802652	1.072205136	0.730980427	-1.719719416	2.483324719	1.464922957	0.179116398	11.98103184
Myeloproliferative neoplasms	genus.Gordonibacter.id.821	MR Egger	10	-0.234469724	0.671308132	0.735899893	-1.550233663	1.081294215	0.790990182	0.212198385	2.948493068
Myeloproliferative neoplasms	genus.Butyricimonas.id.945	MR Egger	13	-0.31985939	0.92717907	0.736614548	-2.137130367	1.497411587	0.726251148	0.117992954	4.470103607
Myeloproliferative neoplasms	genus..Eubacteriumbrachygroup.id.11296	MR Egger	6	0.400895197	1.131747384	0.741053499	-1.817329676	2.619120069	1.493160772	0.16245899	13.72364241
Myeloproliferative neoplasms	phylum.Actinobacteria.id.400	MR Egger	14	0.410536464	1.218555449	0.74200817	-1.977832217	2.798905145	1.507626356	0.138368866	16.42665211
Myeloproliferative neoplasms	family.Prevotellaceae.id.960	MR Egger	13	0.421682515	1.308039007	0.753210782	-2.142073939	2.98543897	1.524524435	0.117411087	19.79518982
Myeloproliferative neoplasms	family.Acidaminococcaceae.id.2166	MR Egger	6	-0.38221309	1.136092017	0.753457196	-2.608953443	1.844527262	0.682349636	0.073611542	6.325108966
Myeloproliferative neoplasms	family.Rikenellaceae.id.967	MR Egger	16	0.318001007	1.009286917	0.757351957	-1.66020135	2.296203364	1.374377645	0.190100699	9.93638591
Myeloproliferative neoplasms	genus.Howardella.id.2000	MR Egger	9	-0.281934542	0.880418848	0.758142588	-2.007555485	1.4436864	0.754323059	0.134316613	4.236283706
Myeloproliferative neoplasms	family.Ruminococcaceae.id.2050	MR Egger	7	0.248240664	0.782359754	0.763839735	-1.285184453	1.781665782	1.281768371	0.276599559	5.939742496
Myeloproliferative neoplasms	genus.Lachnoclostridium.id.11308	MR Egger	13	0.308376465	1.043920701	0.773187973	-1.737708109	2.35446104	1.361213342	0.175923136	10.53245076
Myeloproliferative neoplasms	genus.Hungatella.id.11306	MR Egger	4	-0.931605104	2.914308894	0.779524326	-6.643650536	4.780440329	0.393920919	0.001302265	119.1568067
Myeloproliferative neoplasms	genus.Terrisporobacter.id.11348	MR Egger	5	-0.289691497	0.975686669	0.785905394	-2.202037368	1.622654373	0.748494445	0.110577641	5.066520923
Myeloproliferative neoplasms	genus.Senegalimassilia.id.11160	MR Egger	5	-0.649405588	2.199632249	0.787068932	-4.960684796	3.66187362	0.522356179	0.007008127	38.93422249
Myeloproliferative neoplasms	genus.Adlercreutzia.id.812	MR Egger	7	-0.359394779	1.279918856	0.790115982	-2.868035737	2.149246179	0.698098702	0.056810408	8.57838939
Myeloproliferative neoplasms	genus.ErysipelotrichaceaeUCG003.id.11384	MR Egger	12	0.197727472	0.738491668	0.794336992	-1.249716196	1.645171141	1.218630238	0.28658612	5.181896665
Myeloproliferative neoplasms	genus.Olsenella.id.822	MR Egger	9	-0.143852488	0.554159789	0.802651651	-1.230005673	0.942300698	0.866015487	0.292290919	2.565877943
Myeloproliferative neoplasms	phylum.Proteobacteria.id.2375	MR Egger	10	-0.224350286	0.911700488	0.811817222	-2.011283243	1.56258267	0.799035194	0.133816845	4.771127597
Myeloproliferative neoplasms	genus.Holdemanella.id.11393	MR Egger	9	0.219038977	0.94195194	0.822773271	-1.627186826	2.06526478	1.244879797	0.196481534	7.887386043
Myeloproliferative neoplasms	genus.Subdoligranulum.id.2070	MR Egger	10	0.233084062	1.06025127	0.831502801	-1.845008427	2.311176551	1.262487602	0.158023989	10.08628471
Myeloproliferative neoplasms	genus.Erysipelatoclostridium.id.11381	MR Egger	14	-0.234681229	1.16531073	0.843767477	-2.518690259	2.049327802	0.790822901	0.080565057	7.762681293
Myeloproliferative neoplasms	order.NB1n.id.3953	MR Egger	12	-0.14295549	0.738999311	0.850483406	-1.591394139	1.305483159	0.866792649	0.203641509	3.689471265
Myeloproliferative neoplasms	genus.Coprococcus1.id.11301	MR Egger	9	-0.295102864	1.555931532	0.85495559	-3.344728665	2.754522938	0.744455006	0.035269784	15.71354278
Myeloproliferative neoplasms	genus.Escherichia.Shigella.id.3504	MR Egger	8	0.231034622	1.254256305	0.859924062	-2.227307736	2.68937698	1.259902859	0.107818315	14.72250064
Myeloproliferative neoplasms	genus.LachnospiraceaeND3007group.id.11317	MR Egger	3	-2.389737457	10.7122243	0.860267656	-23.38569708	18.60622217	0.091653744	6.97784E-11	120387006.8
Myeloproliferative neoplasms	genus.Parabacteroides.id.954	MR Egger	5	-0.554394313	2.934326265	0.862203603	-6.305673793	5.196885167	0.574420075	0.001825915	180.7084876
Myeloproliferative neoplasms	genus..Eubacteriumrectalegroup.id.14374	MR Egger	8	0.250481385	1.403470482	0.864225287	-2.500320759	3.00128353	1.284643676	0.082058673	20.11133386
Myeloproliferative neoplasms	family.ClostridialesvadinBB60group.id.11286	MR Egger	14	0.11558879	0.696282087	0.870914367	-1.249124101	1.48030168	1.12253418	0.286755856	4.394271147
Myeloproliferative neoplasms	genus.Victivallis.id.2256	MR Egger	8	-0.327636491	1.964852473	0.873045459	-4.178747338	3.523474357	0.720624926	0.015317683	33.90201176
Myeloproliferative neoplasms	genus.Flavonifractor.id.2059	MR Egger	4	0.413861125	2.50056849	0.883762312	-4.487253115	5.314975365	1.512647043	0.011251508	203.3595033
Myeloproliferative neoplasms	family.Lachnospiraceae.id.1987	MR Egger	15	-0.17454902	1.222846332	0.888684202	-2.571327831	2.222229792	0.839835685	0.076433987	9.227884203
Myeloproliferative neoplasms	class.Clostridia.id.1859	MR Egger	11	-0.293550808	2.056851099	0.889656525	-4.324978962	3.737877346	0.745611339	0.013233829	42.00872547
Myeloproliferative neoplasms	genus.Dialister.id.2183	MR Egger	11	0.164510138	1.173015217	0.891554562	-2.134599688	2.463619964	1.17881552	0.118291935	11.74725932
Myeloproliferative neoplasms	genus..Eubacteriumeligensgroup.id.14372	MR Egger	6	0.218350476	1.547610055	0.894619936	-2.814965231	3.251666183	1.244022992	0.059906802	25.83334717
Myeloproliferative neoplasms	genus.Barnesiella.id.944	MR Egger	10	-0.171950879	1.284516786	0.896816223	-2.68960378	2.345702022	0.842020533	0.06790784	10.44059968
Myeloproliferative neoplasms	family.BacteroidalesS24.7group.id.11173	MR Egger	7	-0.163850552	1.302641286	0.904804652	-2.717027474	2.389326369	0.848868874	0.06607086	10.90614475
Myeloproliferative neoplasms	genus.Tyzzerella3.id.11335	MR Egger	12	-0.177862243	1.472119789	0.906226381	-3.06321703	2.707492544	0.837057726	0.046737098	14.99163747
Myeloproliferative neoplasms	order.Clostridiales.id.1863	MR Egger	12	-0.221228968	1.854621527	0.907411718	-3.856287161	3.413829226	0.801533134	0.021146367	30.38135888
Myeloproliferative neoplasms	genus.Parasutterella.id.2892	MR Egger	13	-0.072726168	0.638875683	0.911420007	-1.324922506	1.17947017	0.929855419	0.265823558	3.252650395
Myeloproliferative neoplasms	genus.Eggerthella.id.819	MR Egger	10	-0.093923193	0.898508841	0.91932019	-1.855000522	1.667154136	0.910352681	0.156452861	5.297071582
Myeloproliferative neoplasms	genus.Catenibacterium.id.2153	MR Egger	4	-0.341928056	3.80075707	0.936514773	-7.791411913	7.107555801	0.710399312	0.000413269	1221.159139
Myeloproliferative neoplasms	genus.Oxalobacter.id.2978	MR Egger	10	0.054783007	0.800818696	0.947139184	-1.514821636	1.624387651	1.056311378	0.219847394	5.075310223
Myeloproliferative neoplasms	genus.Sellimonas.id.14369	MR Egger	7	-0.051672239	1.071696573	0.963411262	-2.152197521	2.048853043	0.949640071	0.116228462	7.758996767
Myeloproliferative neoplasms	genus.RuminococcaceaeUCG011.id.11368	MR Egger	8	-0.039035104	0.875520504	0.965884885	-1.755055292	1.676985085	0.961716949	0.172897682	5.349403635
Myeloproliferative neoplasms	order.Bacillales.id.1674	MR Egger	8	0.027761689	0.725976315	0.970736545	-1.395151888	1.450675265	1.028150635	0.247795396	4.26599422
Myeloproliferative neoplasms	genus.RuminococcaceaeUCG009.id.11366	MR Egger	10	-0.035772188	1.49530799	0.981500018	-2.966575847	2.895031472	0.964860076	0.051479282	18.08407058
Myeloproliferative neoplasms	genus.Ruminococcus1.id.11373	MR Egger	8	-0.026306267	1.109338528	0.98185014	-2.200609781	2.147997248	0.974036729	0.110735613	8.56768226
Myeloproliferative neoplasms	genus.Coprobacter.id.949	MR Egger	10	0.019199576	1.02656447	0.98553628	-1.992866785	2.031265938	1.019385073	0.13630411	7.62373142
Myeloproliferative neoplasms	genus.Prevotella7.id.11182	MR Egger	9	0.019491806	1.264669104	0.988133119	-2.459259638	2.498243249	1.019683011	0.085498227	12.16111115
Myeloproliferative neoplasms	genus.Ruminococcus2.id.11374	MR Egger	13	0.007933979	0.58189978	0.989365639	-1.13258959	1.148457549	1.007965537	0.322197815	3.153325306
Myeloproliferative neoplasms	genus.Phascolarctobacterium.id.2168	Simple mode	7	1.204439929	0.692196242	0.132505493	-0.152264705	2.561144562	3.334890778	0.858760933	12.95063164
Myeloproliferative neoplasms	genus..Eubacteriumhalliigroup.id.11338	Simple mode	13	-1.127004024	0.709236318	0.138036855	-2.517107208	0.263099159	0.324002507	0.080692697	1.300955714

Myeloproliferative neoplasms	genus.RuminococcaceaeUCG005.id.11363	Simple mode	13	1.310608331	0.827222699	0.139099885	-0.310748159	2.931964821	3.708428978	0.732898427	18.7644631
Myeloproliferative neoplasms	genus.Hungatella.id.11306	Simple mode	4	-1.199436186	0.706243911	0.188009165	-2.583674251	0.184801879	0.301364077	0.075496102	1.202980081
Myeloproliferative neoplasms	genus.Ruminiclostridium9.id.11357	Simple mode	7	1.180179096	0.79950739	0.190365785	-0.386855388	2.74721358	3.2549571	0.679189307	15.59910561
Myeloproliferative neoplasms	class.Erysipelotrichia.id.2147	Simple mode	11	-1.087331942	0.79606117	0.201904019	-2.647611836	0.472947952	0.337114737	0.070820141	1.604717858
Myeloproliferative neoplasms	family.Prevotellaceae.id.960	Simple mode	13	-1.044403614	0.804297191	0.218505624	-2.620826108	0.53201888	0.351901626	0.072742745	1.702365713
Myeloproliferative neoplasms	family.Erysipelotrichaceae.id.2149	Simple mode	11	-1.087331942	0.839744853	0.224473749	-2.733231853	0.558567969	0.337114737	0.065008851	1.748167277
Myeloproliferative neoplasms	order.Erysipelotrichales.id.2148	Simple mode	11	-1.087331942	0.851683893	0.230563038	-2.756632372	0.581968488	0.337114737	0.063505271	1.789557689
Myeloproliferative neoplasms	family.Streptococcaceae.id.1850	Simple mode	9	-1.014801312	0.785613714	0.232516872	-2.554604191	0.525001567	0.362474442	0.077722989	1.690461497
Myeloproliferative neoplasms	genus..Ruminococcustorquesgroup.id.14377	Simple mode	10	0.868677476	0.684821655	0.236455556	-0.473572968	2.210927921	2.383756194	0.62277314	9.12417898
Myeloproliferative neoplasms	genus.DefluviitaleaceaeUCG011.id.11287	Simple mode	8	-0.81420212	0.633182635	0.239381424	-2.055240085	0.426835845	0.442992641	0.128062086	1.532401089
Myeloproliferative neoplasms	genus.Tyzzerella3.id.11335	Simple mode	12	-0.573288009	0.466642298	0.244881006	-1.487906914	0.341330895	0.563669039	0.225844874	1.406818674
Myeloproliferative neoplasms	genus.LachnospiraceaeUCG008.id.11328	Simple mode	10	0.603304925	0.49471404	0.253647623	-0.366334594	1.572944444	1.828150729	0.693270798	4.820821957
Myeloproliferative neoplasms	genus.Collinsella.id.815	Simple mode	9	-0.977253052	0.810763583	0.262508825	-2.566349675	0.61184357	0.376343476	0.076815436	1.843827493
Myeloproliferative neoplasms	order.NB1n.id.3953	Simple mode	12	-0.437337674	0.377361136	0.271022496	-1.176965502	0.302290153	0.64575334	0.308212592	1.352953733
Myeloproliferative neoplasms	genus.Eggerthella.id.819	Simple mode	10	0.529568746	0.464564334	0.283744988	-0.380977348	1.440114841	1.698199796	0.683193365	4.221180553
Myeloproliferative neoplasms	family.Lachnospiraceae.id.1987	Simple mode	15	-0.853816932	0.767984174	0.284967677	-2.359065914	0.65143205	0.425786628	0.094508461	1.918285945
Myeloproliferative neoplasms	genus.RuminococcaceaeUCG013.id.11370	Simple mode	11	-0.84047965	0.758186034	0.293577182	-2.326524276	0.645564976	0.431503503	0.097634509	1.907064171
Myeloproliferative neoplasms	family.BacteroidalesS24.7group.id.11173	Simple mode	7	0.557280032	0.509767053	0.316240426	-0.441863391	1.556423456	1.745917197	0.642837447	4.74183151
Myeloproliferative neoplasms	genus.RuminococcaceaeUCG010.id.11367	Simple mode	6	0.730578946	0.660245887	0.31887385	-0.563502992	2.024660885	2.076282316	0.569211623	7.573542208
Myeloproliferative neoplasms	genus.Coprobacter.id.949	Simple mode	10	0.504879442	0.480671161	0.320935749	-0.437236034	1.446994918	1.656785769	0.645818978	4.250322734
Myeloproliferative neoplasms	genus.RuminococcaceaeNK4A214group.id.11358	Simple mode	11	-0.752458957	0.721795024	0.321734817	-2.167177204	0.662259291	0.471206451	0.114500372	1.939168535
Myeloproliferative neoplasms	genus.Lactococcus.id.1851	Simple mode	9	0.536059748	0.508857594	0.3229065	-0.461301136	1.533420633	1.709258667	0.630462793	4.634000959
Myeloproliferative neoplasms	genus.Senegalimassilia.id.11160	Simple mode	5	-0.720521389	0.644253428	0.326032035	-1.983258107	0.542215329	0.486498535	0.137620125	1.719812595
Myeloproliferative neoplasms	genus.Streptococcus.id.1853	Simple mode	10	-0.739214972	0.720366451	0.331602477	-2.151133217	0.672703272	0.47748861	0.116352231	1.959527304
Myeloproliferative neoplasms	class.Methanobacteria.id.119	Simple mode	8	-0.332700773	0.327462278	0.343453981	-0.974526838	0.309125292	0.716984703	0.377370869	1.362233036
Myeloproliferative neoplasms	genus.Slackia.id.825	Simple mode	6	-0.55855218	0.535227028	0.344485294	-1.607597156	0.490492795	0.57203667	0.20036849	1.633120815
Myeloproliferative neoplasms	class.Melainabacteria.id.1589	Simple mode	9	0.444582162	0.446278296	0.348326895	-0.430123297	1.319287621	1.5598383	0.650428894	3.740755592
Myeloproliferative neoplasms	genus.RuminococcaceaeUCG009.id.11366	Simple mode	10	0.486626718	0.496084983	0.352256916	-0.485699849	1.458953285	1.626819234	0.615266452	4.301454776
Myeloproliferative neoplasms	genus.Coprococcus3.id.11303	Simple mode	5	-1.04985496	1.001271938	0.353571507	-3.012347958	0.912638039	0.349988508	0.04917608	2.490884925
Myeloproliferative neoplasms	genus.Victivallis.id.2256	Simple mode	8	-0.356589156	0.359969551	0.354875107	-1.062129477	0.348951165	0.700060054	0.345718826	1.417579961
Myeloproliferative neoplasms	genus.CandidatusSoleaferrea.id.11350	Simple mode	8	-0.408429795	0.415789395	0.358676499	-1.22337701	0.40651742	0.664693136	0.294234853	1.501579298
Myeloproliferative neoplasms	order.MollicutesRF9.id.11579	Simple mode	11	0.663384521	0.689721698	0.358822571	-0.688470007	2.01523905	1.941351774	0.502344064	7.502520641
Myeloproliferative neoplasms	family.Methanobacteriaceae.id.121	Simple mode	8	-0.332700773	0.341101234	0.361869411	-1.001259193	0.335857646	0.716984703	0.367416502	1.399139838
Myeloproliferative neoplasms	order.Methanobacteriales.id.120	Simple mode	8	-0.332700773	0.342020008	0.363082786	-1.003059989	0.337658443	0.716984703	0.366755455	1.401661674
Myeloproliferative neoplasms	genus.Parasutterella.id.2892	Simple mode	13	0.533221437	0.568642435	0.366883901	-0.581317735	1.647760609	1.704414136	0.559161055	5.195332407
Myeloproliferative neoplasms	genus.Oscillibacter.id.2063	Simple mode	13	-0.660902299	0.707090621	0.368381917	-2.046799915	0.724995318	0.51638519	0.129147526	2.064721432
Myeloproliferative neoplasms	genus.Subdoligranulum.id.2070	Simple mode	10	-0.780248587	0.833716318	0.373771313	-2.414332571	0.853835396	0.458292072	0.089427005	2.348637555
Myeloproliferative neoplasms	order.Clostridiales.id.1863	Simple mode	12	-0.753149071	0.825694091	0.381250643	-2.371509489	0.865211346	0.470881376	0.093339725	2.375508088
Myeloproliferative neoplasms	genus.RuminococcaceaeUCG004.id.11362	Simple mode	9	-0.461920963	0.50777227	0.389564777	-1.457154612	0.533312685	0.630072137	0.232898018	1.704569668
Myeloproliferative neoplasms	genus.Romboutsia.id.11347	Simple mode	11	0.750129526	0.835346138	0.390301186	-0.887148905	2.387407957	2.117274241	0.411828242	10.88524233
Myeloproliferative neoplasms	genus..Eubacteriumoxidoreducensgroup.id.11339	Simple mode	3	-0.535010645	0.494055035	0.392040088	-1.503358513	0.433337223	0.585663056	0.222382032	1.542396264
Myeloproliferative neoplasms	genus.Bilophila.id.3170	Simple mode	13	-0.643233519	0.741464657	0.402673553	-2.096504247	0.810037209	0.525590168	0.122885255	2.24799163
Myeloproliferative neoplasms	genus.Dorea.id.1997	Simple mode	10	-0.707963079	0.815688953	0.407977685	-2.306713426	0.890787269	0.492646659	0.099588018	2.437047508
Myeloproliferative neoplasms	family.Christensenellaceae.id.1866	Simple mode	10	0.578942363	0.691213791	0.423953556	-0.775836667	1.933721393	1.784150449	0.460318487	6.915196582
Myeloproliferative neoplasms	genus..Eubacteriumfissicatenagroup.id.14373	Simple mode	8	0.305114832	0.35997559	0.424690804	-0.400437325	1.010666988	1.356780795	0.670026963	2.747432908
Myeloproliferative neoplasms	genus.Alloprevotella.id.961	Simple mode	5	-0.307167898	0.355044065	0.435756268	-1.003054267	0.38871847	0.735527097	0.366757553	1.475089211
Myeloproliferative neoplasms	genus.Anaerotruncus.id.2054	Simple mode	13	-0.578135054	0.736021229	0.447400128	-2.020736663	0.864466555	0.560943521	0.132557779	2.373739488
Myeloproliferative neoplasms	genus.Sutterella.id.2896	Simple mode	12	-0.52025791	0.668890408	0.453095772	-1.831283111	0.79076729	0.594367235	0.160207871	2.205087719
Myeloproliferative neoplasms	class.Clostridia.id.1859	Simple mode	11	-0.630301246	0.814042771	0.456678067	-2.225825077	0.965222585	0.532431384	0.107978292	2.625371961
Myeloproliferative neoplasms	genus.RuminococcaceaeUCG003.id.11361	Simple mode	14	-0.592714368	0.777925644	0.45971036	-2.117448631	0.932019894	0.552824677	0.120338264	2.539633792
Myeloproliferative neoplasms	family.Verrucomicrobiaceae.id.4036	Simple mode	10	-0.55167197	0.727296499	0.467535304	-1.977173108	0.873829169	0.575985974	0.138460096	2.396068259
Myeloproliferative neoplasms	phylum.Euryarchaeota.id.55	Simple mode	11	-0.240947596	0.322972385	0.472821145	-0.87397347	0.392078278	0.785882808	0.417290161	1.480053562
Myeloproliferative neoplasms	phylum.Firmicutes.id.1672	Simple mode	13	-0.518334667	0.700767995	0.473724687	-1.891839938	0.855170603	0.595511447	0.150794102	2.351775567
Myeloproliferative neoplasms	genus.Akkermansia.id.4037	Simple mode	10	-0.55275673	0.739961822	0.474121791	-2.003081902	0.897568442	0.575361506	0.134918835	2.453629708
Myeloproliferative neoplasms	genus.ErysipelotrichaceaeUCG003.id.11384	Simple mode	12	0.463279773	0.626982653	0.475437077	-0.765606227	1.692165773	1.589277917	0.465051918	5.431230788
Myeloproliferative neoplasms	genus.Oxalobacter.id.2978	Simple mode	10	0.284563388	0.389823346	0.48396821	-0.479490369	1.048617146	1.329181565	0.619098823	2.853702135
Myeloproliferative neoplasms	genus.LachnospiraceaeNC2004group.id.11316	Simple mode	8	-0.353307253	0.480821336	0.486343164	-1.295717072	0.589102566	0.702361358	0.27370153	1.802370181
Myeloproliferative neoplasms	class.Verrucomicrobiae.id.4029	Simple mode	10	-0.55190596	0.761723265	0.487136256	-2.044883559	0.941071639	0.575851214	0.129395256	2.562726265
Myeloproliferative neoplasms	genus.Lachnoclostridium.id.11308	Simple mode	13	0.533199882	0.745718696	0.488278046	-0.928408762	1.994808526	1.704377398	0.395182039	7.350795405
Myeloproliferative neoplasms	order.Verrucomicrobiales.id.4030	Simple mode	10	-0.55190596	0.765842325	0.489417461	-2.052956916	0.949144997	0.575851214	0.128354807	2.583499814
Myeloproliferative neoplasms	genus.Erysipelatoclostridium.id.11381	Simple mode	14	0.435676955	0.612707675	0.48960439	-0.765230089	1.636583999	1.546009283	0.465226875	5.137589489
Myeloproliferative neoplasms	genus.LachnospiraceaeUCG004.id.11324	Simple mode	12	0.631890152	0.889947169	0.492462845	-1.1124063	2.376186603	1.881162904	0.328766897	10.76377794
Myeloproliferative neoplasms	genus.Ruminiclostridium6.id.11356	Simple mode	12	0.441602488	0.632001802	0.499223786	-0.797121044	1.680326019	1.555197408	0.450624426	5.367305532
Myeloproliferative neoplasms	genus.Blautia.id.1992	Simple mode	11	-0.567935097	0.818516552	0.503569278	-2.17222754	1.036357345	0.5666944	0.113923565	2.818929901
Myeloproliferative neoplasms	phylum.Proteobacteria.id.2375	Simple mode	10	-0.531870339	0.770089864	0.5071988	-2.041246472	0.977505795	0.587505108	0.129866735	2.657818822
Myeloproliferative neoplasms	phylum.Bacteroidetes.id.905	Simple mode	10	-0.439422908	0.64753968	0.51445766	-1.708600681	0.829754866	0.644408197	0.181119059	2.292756638
Myeloproliferative neoplasms	genus.Anaerostipes.id.1991	Simple mode	12	0.512792174	0.770567634	0.519457916	-0.997520387	2.023104736	1.669947476	0.368792772	7.561765814
Myeloproliferative neoplasms	genus.Butyricimonas.id.945	Simple mode	13	-0.385925963	0.581835608	0.519683596	-1.526323755	0.754471829	0.679820856	0.21733317	2.126488078
Myeloproliferative neoplasms	phylum.Verrucomicrobia.id.3982	Simple mode	12	-0.40899564	0.618924121	0.522328484	-1.622086918	0.804095637	0.664317128	0.197486131	2.234674628
Myeloproliferative neoplasms	class.Deltaproteobacteria.id.3087	Simple mode	11	0.454919072	0.709699436	0.535934116	-0.936091823	1.845929968	1.576045832	0.392157465	6.333987456
Myeloproliferative neoplasms	genus.Ruminiclostridium5.id.11355	Simple mode	10	0.497979132	0.77916616	0.538670134	-1.029186542	2.025144805	1.645392787	0.357297489	7.577208085
Myeloproliferative neoplasms	family.Defluviitaleaceae.id.1924	Simple mode	10	0.389635223	0.614046054	0.541521879	-0.813895042	1.593165489	1.476442124	0.443128696	4.919296285
Myeloproliferative neoplasms	genus.Peptococcus.id.2037	Simple mode	11	0.254612138	0.408346441	0.54690079	-0.545746885	1.054971162	1.289961197	0.57940887	2.871892333
Myeloproliferative neoplasms	genus.Butyricicoccus.id.2055	Simple mode	7	-0.624585804	0.985208738	0.549494966	-2.555594929	1.306423322	0.535483178	0.077646024	3.692941601
Myeloproliferative neoplasms	genus.LachnospiraceaeNK4A136group.id.11319	Simple mode	13	0.453971747	0.739309707	0.550647578	-0.995075278	1.903018772	1.574553511	0.369695614	6.70610813
Myeloproliferative neoplasms	genus.RikenellaceaeRC9gutgroup.id.11191	Simple mode	10	-0.2117092	0.342604465	0.551928419	-0.883213951	0.459795552	0.809199979	0.41345196	1.583750157
Myeloproliferative neoplasms	genus.Odoribacter.id.952	Simple mode	5	0.757067431	1.169345062	0.552636871	-1.53484889	3.048983751	2.132014763	0.215488251	21.09389688
Myeloproliferative neoplasms	genus.Alistipes.id.968	Simple mode	12	0.525711425	0.863056356	0.554811624	-1.165879034	2.217301883	1.69166191	0.311648592	9.182521894
Myeloproliferative neoplasms	genus.Holdemania.id.2157	Simple mode	12	-0.340552721	0.567591351	0.560665436	-1.453031769	0.771926327	0.711377021	0.233860202	2.163930679
Myeloproliferative neoplasms	genus.FamilyXIIIAD3011group.id.11293	Simple mode	12	-0.417390345	0.695665498	0.560671344	-1.780894721	0.94611403	0.658763724	0.168487331	2.575681168
Myeloproliferative neoplasms	genus.Veillonella.id.2198	Simple mode	6	0.470006708	0.757347967	0.562055918	-1.014395306	1.954408723	1.600004927	0.362621639	7.059743526
Myeloproliferative neoplasms	family.Peptostreptococcaceae.id.2042	Simple mode	12	-0.318621038	0.540439729	0.567398208	-1.377882907	0.74064083	0.727151059	0.252111732	2.097279084
Myeloproliferative neoplasms	genus.Terrisporobacter.id.11348	Simple mode	5	-0.361171553	0.585582215	0.570758063	-1.508912695	0.786569588	0.69685944	0.221150305	2.195850818
Myeloproliferative neoplasms	family.Coriobacteriaceae.id.811	Simple mode	12	0.392059987	0.678814262	0.575188616	-0.938415967	1.72253594	1.480026491	0.391247093	5.598708466
Myeloproliferative neoplasms	family.Desulfovibrionaceae.id.3169	Simple mode	8	0.484484226	0.825467662	0.575688746	-1.133432391	2.102400844	1.623337518	0.321926381	8.185799169
Myeloproliferative neoplasms	class.Coriobacteriia.id.809	Simple mode	12	0.392059987	0.681586087	0.576721093	-0.943848743	1.727968716	1.480026491	0.389127299	5.62920777
Myeloproliferative neoplasms	family.Pasteurellaceae.id.3689	Simple mode	10	-0.345627791	0.601591421	0.579689166	-1.524746977	0.833491394	0.707775878	0.217676127	2.301339613
Myeloproliferative neoplasms	order.Coriobacteriales.id.810	Simple mode	12	0.392059987	0.688483675	0.580490338	-0.957368017	1.74148799	1.480026491	0.383901981	5.705827325
Myeloproliferative neoplasms	genus.RuminococcaceaeUCG014.id.11371	Simple mode	10	-0.416991452	0.737263268	0.585492379	-1.862027457	1.028044553	0.659026553	0.155357331	2.795593852
Myeloproliferative neoplasms	order.Pasteurellales.id.3688	Simple mode	10	-0.345627791	0.612532068	0.58636144	-1.546190645	0.854935062	0.707775878	0.213058044	2.351221693
Myeloproliferative neoplasms	genus..Eubacteriumventriosumgroup.id.11341	Simple mode	13	-0.337900823	0.609242538	0.589339544	-1.532016197	0.85621455	0.713266024	0.216099529	2.354231978
Myeloproliferative neoplasms	genus.Catenibacterium.id.2153	Simple mode	4	0.275984949	0.462929121	0.593048109	-0.631356127	1.183326026	1.31782803	0.531870028	3.265216356
Myeloproliferative neoplasms	genus.Dialister.id.2183	Simple mode	11	0.33811	0.614495101	0.594241827	-0.866300399	1.542520399	1.402294747	0.420504374	4.676361727
Myeloproliferative neoplasms	genus..Ruminococcusgauvreauiigroup.id.11342	Simple mode	9	-0.311565721	0.562402041	0.594726875	-1.413873722	0.79074228	0.732299481	0.243199369	2.205032571
Myeloproliferative neoplasms	genus.Howardella.id.2000	Simple mode	9	0.215709708	0.405257975	0.608994812	-0.578595922	1.010015338	1.240742149	0.56068506	2.745643129
Myeloproliferative neoplasms	genus.Gordonibacter.id.821	Simple mode	10	0.195657756	0.375762871	0.615145936	-0.540837472	0.932152984	1.216110627	0.582260421	2.539971813
Myeloproliferative neoplasms	phylum.Tenericutes.id.3919	Simple mode	10	0.3104694	0.599010554	0.616746703	-0.863591286	1.484530086	1.364065256	0.421645112	4.412891248
Myeloproliferative neoplasms	class.Mollicutes.id.3920	Simple mode	10	0.3104694	0.599321396	0.616926809	-0.864200536	1.485139336	1.364065256	0.421388303	4.415580621
Myeloproliferative neoplasms	genus.LachnospiraceaeFCS020group.id.11314	Simple mode	11	-0.319821582	0.620503587	0.617459287	-1.536008612	0.896365448	0.726278607	0.21523849	2.450679782
Myeloproliferative neoplasms	order.Bifidobacteriales.id.432	Simple mode	9	0.292814176	0.570758566	0.621795642	-0.825872614	1.411500965	1.340193727	0.437852749	4.102107906
Myeloproliferative neoplasms	genus.Coprococcus1.id.11301	Simple mode	9	-0.453113582	0.904055334	0.629727494	-2.225062036	1.318834873	0.635645932	0.108060715	3.739062354
Myeloproliferative neoplasms	family.Oxalobacteraceae.id.2966	Simple mode	14	0.17645747	0.360007245	0.632196193	-0.529156731	0.88207167	1.192983688	0.589101531	2.415899473
Myeloproliferative neoplasms	family.Acidaminococcaceae.id.2166	Simple mode	6	-0.289195001	0.569530269	0.633209075	-1.405474328	0.827084326	0.748866162	0.2452507	2.28664191
Myeloproliferative neoplasms	family.Clostridiaceae1.id.1869	Simple mode	7	-0.347800604	0.696715805	0.63541089	-1.713363582	1.017762374	0.706239684	0.180258458	2.766996329
Myeloproliferative neoplasms	genus.Flavonifractor.id.2059	Simple mode	4	0.389664893	0.745337175	0.637265734	-1.07119597	1.850525756	1.47648593	0.342598535	6.363164112
Myeloproliferative neoplasms	class.Gammaproteobacteria.id.3303	Simple mode	6	0.439122541	0.884552404	0.640639481	-1.294600171	2.172845252	1.551345379	0.274007399	8.783239048
Myeloproliferative neoplasms	genus..Eubacteriumxylanophilumgroup.id.14375	Simple mode	7	-0.355997023	0.725581463	0.641109141	-1.77813669	1.066142643	0.700474705	0.168952665	2.904155502
Myeloproliferative neoplasms	genus.Holdemanella.id.11393	Simple mode	9	-0.195999156	0.410848116	0.646079314	-1.001261464	0.609263152	0.822012929	0.367415667	1.839075781
Myeloproliferative neoplasms	genus.Ruminococcus2.id.11374	Simple mode	13	0.230053297	0.493911653	0.649714692	-0.738013542	1.198120136	1.258667092	0.478062624	3.313881418
Myeloproliferative neoplasms	genus..Ruminococcusgnavusgroup.id.14376	Simple mode	11	-0.211262734	0.45550457	0.652722805	-1.104051691	0.681526223	0.80956134	0.331525122	1.97689261
Myeloproliferative neoplasms	order.Desulfovibrionales.id.3156	Simple mode	10	0.39072028	0.87451089	0.665586161	-1.323321065	2.104761626	1.478045017	0.2662496	8.205146879
Myeloproliferative neoplasms	genus.Prevotella9.id.11183	Simple mode	13	-0.24027526	0.555819667	0.673197006	-1.329681808	0.849131288	0.786411364	0.264561429	2.337615256
Myeloproliferative neoplasms	genus.Actinomyces.id.423	Simple mode	5	-0.306058477	0.674347571	0.673465156	-1.627779715	1.015662762	0.736343559	0.196365077	2.761192804
Myeloproliferative neoplasms	genus..Eubacteriumbrachygroup.id.11296	Simple mode	6	0.188229416	0.421258944	0.673690118	-0.637438115	1.013896947	1.207110414	0.528645018	2.756321352
Myeloproliferative neoplasms	genus.Butyrivibrio.id.1993	Simple mode	13	0.144400078	0.340310013	0.678840821	-0.522607548	0.811407705	1.155346245	0.592972326	2.251074605
Myeloproliferative neoplasms	family.Rikenellaceae.id.967	Simple mode	16	0.317150996	0.75445908	0.680176178	-1.1615888	1.795890791	1.373209905	0.312988509	6.024839208
Myeloproliferative neoplasms	family.Bifidobacteriaceae.id.433	Simple mode	10	0.231369484	0.548524145	0.683064846	-0.843737839	1.306476808	1.260324824	0.430099871	3.693139124
Myeloproliferative neoplasms	family.ClostridialesvadinBB60group.id.11286	Simple mode	14	0.213220312	0.514303518	0.685208531	-0.794814584	1.221255208	1.237657292	0.451664973	3.391442028
Myeloproliferative neoplasms	family.Alcaligenaceae.id.2875	Simple mode	11	-0.249994085	0.600769875	0.686109839	-1.427503041	0.927514871	0.77880539	0.239907214	2.528218415
Myeloproliferative neoplasms	order.Enterobacteriales.id.3468	Simple mode	5	-0.3930118	0.925535307	0.692949411	-2.207061002	1.421037401	0.675020782	0.110023533	4.14141452
Myeloproliferative neoplasms	family.Enterobacteriaceae.id.3469	Simple mode	5	-0.3930118	0.931204151	0.694686957	-2.218171936	1.432148335	0.675020782	0.108807835	4.187686087
Myeloproliferative neoplasms	class.Betaproteobacteria.id.2867	Simple mode	10	0.279545811	0.69814146	0.698189723	-1.08881145	1.647903072	1.322528998	0.336616341	5.196072604
Myeloproliferative neoplasms	class.Actinobacteria.id.419	Simple mode	12	0.22300665	0.560882363	0.698532271	-0.876322781	1.322336081	1.249828885	0.416310967	3.752176534
Myeloproliferative neoplasms	genus.Oscillospira.id.2064	Simple mode	7	0.305976675	0.753230897	0.698672884	-1.170355883	1.782309233	1.357950632	0.310256507	5.94356566
Myeloproliferative neoplasms	genus..Eubacteriumruminantiumgroup.id.11340	Simple mode	16	0.166791346	0.427162771	0.701683262	-0.670447685	1.004030376	1.181507713	0.511479545	2.729259635
Myeloproliferative neoplasms	class.Alphaproteobacteria.id.2379	Simple mode	6	0.207063863	0.525067299	0.709576284	-0.822068043	1.236195768	1.230061124	0.439521764	3.442492484
Myeloproliferative neoplasms	genus.Adlercreutzia.id.812	Simple mode	7	0.240073919	0.615924927	0.710160837	-0.967138939	1.447286777	1.271343123	0.380169171	4.251563411
Myeloproliferative neoplasms	genus.Anaerofilum.id.2053	Simple mode	9	-0.178108531	0.472691965	0.716123024	-1.104584781	0.74836772	0.836851595	0.331348436	2.113547298
Myeloproliferative neoplasms	genus.Parabacteroides.id.954	Simple mode	5	0.292244232	0.765346886	0.721994739	-1.207835666	1.792324129	1.339430109	0.298843377	6.003388915

Myeloproliferative neoplasms	genus.Olsenella.id.822	Simple mode	9	-0.12965647	0.354384174	0.723951991	-0.824249452	0.564936512	0.878397135	0.438564032	1.759336083
Myeloproliferative neoplasms	genus.Allisonella.id.2174	Simple mode	8	-0.141071265	0.392603091	0.729948317	-0.910573324	0.628430793	0.868427421	0.402293513	1.87466653
Myeloproliferative neoplasms	genus.Bifidobacterium.id.436	Simple mode	12	0.158483016	0.459519063	0.736682087	-0.742174349	1.05914038	1.171732023	0.476077631	2.883890872
Myeloproliferative neoplasms	genus.Roseburia.id.2012	Simple mode	11	-0.24425813	0.708406695	0.7373871	-1.632735252	1.144218992	0.783285419	0.19539439	3.139988044
Myeloproliferative neoplasms	genus.RuminococcaceaeUCG002.id.11360	Simple mode	17	0.271469401	0.796388442	0.7376322	-1.289451945	1.832390747	1.311890729	0.275421688	6.248808135
Myeloproliferative neoplasms	genus.LachnospiraceaeUCG001.id.11321	Simple mode	11	0.168675059	0.49449478	0.740082373	-0.80053471	1.137884827	1.183735432	0.449088768	3.120161698
Myeloproliferative neoplasms	genus.Coprococcus2.id.11302	Simple mode	6	-0.308903728	0.882092198	0.740462366	-2.037804437	1.419996981	0.734251454	0.130314511	4.13710795
Myeloproliferative neoplasms	order.Gastranaerophilales.id.1591	Simple mode	9	0.154203243	0.460025254	0.746090275	-0.747446256	1.055852742	1.166727992	0.473574398	2.874425252
Myeloproliferative neoplasms	family.FamilyXIII.id.1957	Simple mode	5	0.349886337	1.054228881	0.756635377	-1.716402269	2.416174943	1.418906262	0.17971154	11.20292542
Myeloproliferative neoplasms	genus.Intestinimonas.id.2062	Simple mode	14	0.216749328	0.704746981	0.763291846	-1.164554754	1.598053411	1.24203272	0.312061575	4.943400282
Myeloproliferative neoplasms	genus.ChristensenellaceaeR.7group.id.11283	Simple mode	6	-0.275330894	0.869852271	0.764391397	-1.980241345	1.429579557	0.759320827	0.138035919	4.176942655
Myeloproliferative neoplasms	genus..Eubacteriumcoprostanoligenesgroup.id.11375	Simple mode	12	-0.240499619	0.787314339	0.76571229	-1.783635724	1.302636486	0.786234945	0.168026139	3.678983482
Myeloproliferative neoplasms	family.Bacteroidaceae.id.917	Simple mode	7	-0.224443843	0.726262717	0.767741483	-1.649718769	1.199031083	0.798960443	0.192450025	3.316901564
Myeloproliferative neoplasms	genus.Eisenbergiella.id.11304	Simple mode	11	-0.141481831	0.469109299	0.769138063	-1.060936058	0.777972395	0.868070947	0.34613166	2.177053583
Myeloproliferative neoplasms	order.Lactobacillales.id.1800	Simple mode	11	0.195950861	0.689052613	0.781923032	-1.15459226	1.546493982	1.216467128	0.315186025	4.694980604
Myeloproliferative neoplasms	genus.Sellimonas.id.14369	Simple mode	7	0.097789552	0.338289497	0.782255304	-0.565257862	0.760836965	1.102730693	0.568213607	2.140066633
Myeloproliferative neoplasms	genus.Bacteroides.id.918	Simple mode	7	-0.224443843	0.789607352	0.785775643	-1.772074253	1.323186568	0.798960443	0.169980041	3.755369068
Myeloproliferative neoplasms	genus.Marvinbryantia.id.2005	Simple mode	9	0.189472363	0.68111596	0.787929196	-1.14551492	1.524459645	1.208611721	0.3180601	4.592661232
Myeloproliferative neoplasms	phylum.Actinobacteria.id.400	Simple mode	14	0.164555523	0.647362582	0.803322226	-1.104275138	1.433386184	1.178869022	0.331451051	4.192873021
Myeloproliferative neoplasms	class.Bacilli.id.1673	Simple mode	14	0.149839925	0.589474502	0.803323265	-1.005530099	1.305209949	1.161648277	0.365850646	3.688463401
Myeloproliferative neoplasms	family.Lactobacillaceae.id.1836	Simple mode	7	-0.132454549	0.514049334	0.805272095	-1.135991244	0.875082146	0.875942746	0.319821822	2.399072361
Myeloproliferative neoplasms	genus.Lachnospira.id.2004	Simple mode	5	-0.244126377	0.932344032	0.806374307	-2.07152068	1.583267926	0.783388626	0.125994039	4.8708474
Myeloproliferative neoplasms	family.Victivallaceae.id.2255	Simple mode	9	-0.086837128	0.371133581	0.820878552	-0.814258947	0.640584691	0.916826409	0.442967468	1.89759006
Myeloproliferative neoplasms	genus.Enterorhabdus.id.820	Simple mode	6	0.1327133	0.568944399	0.824806472	-0.982417723	1.247844323	1.141922562	0.374404797	3.482827009
Myeloproliferative neoplasms	family.Actinomycetaceae.id.421	Simple mode	3	-0.182852662	0.756811119	0.831596341	-1.666202454	1.300497131	0.832890863	0.188963302	3.671121243
Myeloproliferative neoplasms	order.Actinomycetales.id.420	Simple mode	3	-0.185000048	0.776925786	0.833962261	-1.707774588	1.337774492	0.831104244	0.181268742	3.810553645
Myeloproliferative neoplasms	genus.Turicibacter.id.2162	Simple mode	8	-0.130293567	0.615849336	0.838472502	-1.337358264	1.076771131	0.877837689	0.26253831	2.9351869
Myeloproliferative neoplasms	genus.Fusicatenibacter.id.11305	Simple mode	18	0.140145028	0.699604821	0.843606979	-1.231080421	1.511370476	1.150440632	0.291976949	4.532938825
Myeloproliferative neoplasms	genus.Intestinibacter.id.11345	Simple mode	14	-0.097082954	0.484478416	0.844279144	-1.046660649	0.85249474	0.907480723	0.351108267	2.34549095
Myeloproliferative neoplasms	family.Veillonellaceae.id.2172	Simple mode	18	-0.124559943	0.628284782	0.845197843	-1.355998115	1.106878229	0.882885337	0.257689962	3.024900592
Myeloproliferative neoplasms	genus.FamilyXIIIUCG001.id.11294	Simple mode	6	-0.150626472	0.747468721	0.846953856	-1.615691165	1.314412221	0.860168936	0.198758419	3.722562299
Myeloproliferative neoplasms	family.Ruminococcaceae.id.2050	Simple mode	7	-0.135923226	0.679021812	0.847956078	-1.466805978	1.194959525	0.872909646	0.230661046	3.303424061
Myeloproliferative neoplasms	genus.Barnesiella.id.944	Simple mode	10	0.125766907	0.640042424	0.848586038	-1.128716244	1.380250058	1.134017806	0.323448219	3.975895709
Myeloproliferative neoplasms	genus.LachnospiraceaeND3007group.id.11317	Simple mode	3	0.174324711	0.880735552	0.861392731	-1.551916971	1.900566392	1.190442053	0.21184149	6.689682355
Myeloproliferative neoplasms	order.Burkholderiales.id.2874	Simple mode	10	0.115844268	0.67429044	0.867394348	-1.205764993	1.43745353	1.122821	0.299462825	4.209961616
Myeloproliferative neoplasms	genus.Ruminococcus1.id.11373	Simple mode	8	-0.110900908	0.697936116	0.878236218	-1.478855696	1.25705388	0.895027435	0.227898324	3.515050456
Myeloproliferative neoplasms	order.Rhodospirillales.id.2667	Simple mode	12	-0.095127752	0.609678835	0.878836552	-1.290098269	1.099842766	0.909256768	0.275243734	3.003693703
Myeloproliferative neoplasms	class.Bacteroidia.id.912	Simple mode	12	-0.098184932	0.631360989	0.879233844	-1.335652471	1.139282607	0.906481251	0.262986528	3.124526049
Myeloproliferative neoplasms	order.Bacteroidales.id.913	Simple mode	12	-0.098184932	0.634726049	0.879868538	-1.342247989	1.145878125	0.906481251	0.261257704	3.145202026
Myeloproliferative neoplasms	genus..Eubacteriumnodatumgroup.id.11297	Simple mode	11	0.052988839	0.386898699	0.893782244	-0.705332611	0.811310289	1.054417876	0.493944255	2.250855326
Myeloproliferative neoplasms	family.Porphyromonadaceae.id.943	Simple mode	9	0.11091421	0.824618345	0.896326661	-1.505337747	1.727166167	1.11729905	0.221942321	5.624691864
Myeloproliferative neoplasms	genus.LachnospiraceaeUCG010.id.11330	Simple mode	10	-0.084402008	0.647058001	0.899087616	-1.352635691	1.183831675	0.919061712	0.258557883	3.266867828
Myeloproliferative neoplasms	family.Peptococcaceae.id.2024	Simple mode	8	-0.063090871	0.484668572	0.90009118	-1.013041272	0.886859529	0.938858154	0.363112973	2.427494193
Myeloproliferative neoplasms	genus..Eubacteriumrectalegroup.id.14374	Simple mode	8	0.092199758	0.721865078	0.901958952	-1.322655794	1.50705531	1.096583851	0.266426787	4.513420581
Myeloproliferative neoplasms	phylum.Lentisphaerae.id.2238	Simple mode	9	0.048735002	0.398313524	0.90563694	-0.731959505	0.829429509	1.049942081	0.480965612	2.292010797
Myeloproliferative neoplasms	genus.RuminococcaceaeUCG011.id.11368	Simple mode	8	-0.037367971	0.358571738	0.919923092	-0.740168576	0.665432635	0.963321596	0.477033492	1.945331958
Myeloproliferative neoplasms	order.Bacillales.id.1674	Simple mode	8	-0.028658212	0.380127123	0.942012893	-0.773707373	0.71639095	0.97174854	0.461299684	2.047032023
Myeloproliferative neoplasms	genus.Methanobrevibacter.id.123	Simple mode	6	0.032989951	0.445954744	0.943897816	-0.841081249	0.907061249	1.033540153	0.431243947	2.477032446
Myeloproliferative neoplasms	family.Rhodospirillaceae.id.2717	Simple mode	13	-0.03200044	0.554439096	0.954924182	-1.118701069	1.054700189	0.968506156	0.326703885	2.871114234
Myeloproliferative neoplasms	genus.Prevotella7.id.11182	Simple mode	9	0.016337647	0.36320843	0.965224616	-0.695550875	0.728226169	1.016471836	0.498799596	2.071403028
Myeloproliferative neoplasms	genus.Escherichia.Shigella.id.3504	Simple mode	8	0.028553721	0.648146514	0.966091382	-1.241813446	1.298920888	1.028965286	0.288859911	3.665339219
Myeloproliferative neoplasms	genus.Faecalibacterium.id.2057	Simple mode	10	0.022747994	0.636577876	0.972273843	-1.224944643	1.270440631	1.023008703	0.293773962	3.562421931
Myeloproliferative neoplasms	genus.Haemophilus.id.3698	Simple mode	8	0.01931127	0.556404802	0.973282156	-1.071242143	1.109864682	1.019498938	0.342582716	3.03394782
Myeloproliferative neoplasms	genus..Clostridiuminnocuumgroup.id.14397	Simple mode	6	-0.011560201	0.345337797	0.974590987	-0.688422283	0.665301882	0.988506362	0.502368039	1.945077616
Myeloproliferative neoplasms	class.Negativicutes.id.2164	Simple mode	12	0.021709479	0.70897369	0.976120323	-1.367878953	1.411297911	1.021946844	0.254646504	4.101275042
Myeloproliferative neoplasms	order.Selenomonadales.id.2165	Simple mode	12	0.021709479	0.725035151	0.976649147	-1.399359417	1.442778375	1.021946844	0.24675498	4.232438798
Myeloproliferative neoplasms	genus.Desulfovibrio.id.3173	Simple mode	8	-0.016391958	0.603266091	0.979080951	-1.198793496	1.166009579	0.983741659	0.301557823	3.209161151
Myeloproliferative neoplasms	genus..Eubacteriumeligensgroup.id.14372	Simple mode	6	0.020637854	0.831368167	0.981155636	-1.608843752	1.650119461	1.020852287	0.200118867	5.207601896
Myeloproliferative neoplasms	phylum.Cyanobacteria.id.1500	Simple mode	7	-0.012276217	0.57916601	0.983776326	-1.147441597	1.122889162	0.987798828	0.317447891	3.073721868
Myeloproliferative neoplasms	family.FamilyXI.id.1936	Simple mode	8	0.006700421	0.318179479	0.983786592	-0.616931359	0.630332201	1.006722919	0.539597731	1.878234426
Myeloproliferative neoplasms	order.Victivallales.id.2254	Simple mode	8	-0.00841437	0.39974955	0.983793917	-0.791923489	0.775094748	0.991620931	0.452972669	2.170797796
Myeloproliferative neoplasms	class.Lentisphaeria.id.2250	Simple mode	8	-0.00841437	0.408269606	0.984132061	-0.808622798	0.791794057	0.991620931	0.445471148	2.207352994
Myeloproliferative neoplasms	genus.Paraprevotella.id.962	Simple mode	13	0.007838033	0.422631373	0.985508244	-0.820519459	0.836195525	1.007868831	0.440202928	2.307571158
Myeloproliferative neoplasms	genus.Lactobacillus.id.1837	Simple mode	7	0.007128066	0.470277873	0.988398252	-0.914616565	0.928872698	1.007153531	0.400670227	2.53165363
Myeloproliferative neoplasms	genus.Clostridiumsensustricto1.id.1873	Simple mode	5	0.00100522	0.742737689	0.998984952	-1.45476065	1.45677109	1.001005725	0.233456235	4.292078394
Myeloproliferative neoplasms	genus..Eubacteriumhalliigroup.id.11338	Weighted median	13	-0.952234255	0.388560467	0.014258982	-1.713812771	-0.19065574	0.38587791	0.180177506	0.826417042
Myeloproliferative neoplasms	genus.LachnospiraceaeUCG008.id.11328	Weighted median	10	0.616279383	0.309306664	0.046321239	0.01003832	1.222520445	1.852024532	1.010088873	3.395735722
Myeloproliferative neoplasms	genus.Collinsella.id.815	Weighted median	9	-0.98738847	0.500446572	0.048493881	-1.968263752	-0.006513189	0.372548343	0.139699198	0.993507976
Myeloproliferative neoplasms	family.Lachnospiraceae.id.1987	Weighted median	15	-0.826830029	0.428699972	0.053769161	-1.667081974	0.013421915	0.437433743	0.188797178	1.013512394
Myeloproliferative neoplasms	genus.RuminococcaceaeUCG013.id.11370	Weighted median	11	-0.774217603	0.44240338	0.080113755	-1.641328227	0.092893022	0.461064375	0.193722564	1.097344337
Myeloproliferative neoplasms	genus.Lactococcus.id.1851	Weighted median	9	0.469609889	0.271967561	0.084219242	-0.06344653	1.002666309	1.59937014	0.938524301	2.725539278
Myeloproliferative neoplasms	genus.Phascolarctobacterium.id.2168	Weighted median	7	0.739623003	0.436241533	0.089991263	-0.115410401	1.594656408	2.095145502	0.891000401	4.926636026
Myeloproliferative neoplasms	family.Christensenellaceae.id.1866	Weighted median	10	0.701530626	0.416519234	0.092129604	-0.114847072	1.517908324	2.01683737	0.891502469	4.562671576
Myeloproliferative neoplasms	genus.Romboutsia.id.11347	Weighted median	11	0.756904397	0.451238129	0.093465346	-0.127522335	1.641331129	2.131667201	0.880273755	5.162036276
Myeloproliferative neoplasms	genus..Ruminococcustorquesgroup.id.14377	Weighted median	10	0.79070289	0.482078397	0.100964493	-0.154170767	1.735576548	2.204945717	0.85712564	5.672197154
Myeloproliferative neoplasms	genus.Ruminiclostridium9.id.11357	Weighted median	7	0.902028312	0.561944352	0.108451661	-0.199382617	2.003439241	2.464597017	0.81923638	7.414512596
Myeloproliferative neoplasms	genus.Coprococcus3.id.11303	Weighted median	5	-1.072045874	0.695937108	0.123454358	-2.436082606	0.291990859	0.342307483	0.087502964	1.339090777
Myeloproliferative neoplasms	order.Bifidobacteriales.id.432	Weighted median	9	0.583909131	0.391542223	0.135881265	-0.183513626	1.351331887	1.793033953	0.832340535	3.862566609
Myeloproliferative neoplasms	genus.Streptococcus.id.1853	Weighted median	10	-0.6801302	0.475856566	0.152925023	-1.612809069	0.252548669	0.506551035	0.199326904	1.287302146
Myeloproliferative neoplasms	order.NB1n.id.3953	Weighted median	12	-0.334570821	0.234953683	0.154450443	-0.795080039	0.125938397	0.71564516	0.451545092	1.134212296
Myeloproliferative neoplasms	order.Clostridiales.id.1863	Weighted median	12	-0.674380114	0.476868807	0.157307961	-1.609042975	0.260282748	0.509472138	0.200079003	1.297296843
Myeloproliferative neoplasms	class.Melainabacteria.id.1589	Weighted median	9	0.430132118	0.315267856	0.17246101	-0.187792879	1.048057115	1.537460636	0.828786348	2.852104421
Myeloproliferative neoplasms	genus.Slackia.id.825	Weighted median	6	-0.481401867	0.353133651	0.1728105	-1.173543823	0.210740089	0.617916548	0.309269002	1.23459143
Myeloproliferative neoplasms	genus.RuminococcaceaeUCG010.id.11367	Weighted median	6	0.625313365	0.469494686	0.182897676	-0.294896219	1.54552295	1.868831493	0.74460886	4.690423841
Myeloproliferative neoplasms	genus.Sutterella.id.2896	Weighted median	12	-0.488842804	0.367599464	0.183576074	-1.209337754	0.231652145	0.613335733	0.298394825	1.260681119
Myeloproliferative neoplasms	genus.RuminococcaceaeUCG009.id.11366	Weighted median	10	0.417799236	0.321715736	0.194060767	-0.212763607	1.048362079	1.51861576	0.808347202	2.852974342
Myeloproliferative neoplasms	family.Bifidobacteriaceae.id.433	Weighted median	10	0.485253564	0.37374073	0.194160395	-0.247278266	1.217785394	1.624586893	0.780923359	3.379694748
Myeloproliferative neoplasms	class.Clostridia.id.1859	Weighted median	11	-0.602221787	0.475604512	0.205432943	-1.53440663	0.329963056	0.547593647	0.215583574	1.390916742
Myeloproliferative neoplasms	order.MollicutesRF9.id.11579	Weighted median	11	0.46619226	0.377069602	0.216326268	-0.272864159	1.205248679	1.593913415	0.761196182	3.337588963
Myeloproliferative neoplasms	genus.Dorea.id.1997	Weighted median	10	-0.635071993	0.515130269	0.217636339	-1.64472732	0.374583334	0.529897338	0.193065201	1.454385295
Myeloproliferative neoplasms	genus.Faecalibacterium.id.2057	Weighted median	10	-0.480891272	0.391945425	0.219847299	-1.249104305	0.287321761	0.618232133	0.286761533	1.332853005
Myeloproliferative neoplasms	genus.Senegalimassilia.id.11160	Weighted median	5	-0.579708139	0.483990736	0.231007732	-1.528329981	0.368913703	0.560061803	0.216897588	1.446162799
Myeloproliferative neoplasms	genus.RuminococcaceaeUCG004.id.11362	Weighted median	9	-0.429492091	0.365613838	0.240108723	-1.146095214	0.287111033	0.650839578	0.317875585	1.332572165
Myeloproliferative neoplasms	genus..Eubacteriumfissicatenagroup.id.14373	Weighted median	8	0.289325173	0.250456366	0.248011837	-0.201569304	0.78021965	1.335525935	0.817446924	2.181951479
Myeloproliferative neoplasms	class.Methanobacteria.id.119	Weighted median	8	-0.258530465	0.224146416	0.248746263	-0.697857441	0.180796511	0.772185506	0.497650408	1.19817134
Myeloproliferative neoplasms	family.Desulfovibrionaceae.id.3169	Weighted median	8	0.587822038	0.510957957	0.249966266	-0.413655557	1.589299633	1.800063672	0.661228667	4.900315708
Myeloproliferative neoplasms	genus.Alloprevotella.id.961	Weighted median	5	-0.30030648	0.261054055	0.249995097	-0.811972429	0.211359469	0.74059121	0.44398148	1.235356347
Myeloproliferative neoplasms	genus.Victivallis.id.2256	Weighted median	8	-0.266551712	0.231901119	0.250382966	-0.721077906	0.187974482	0.766016391	0.486227866	1.20680272
Myeloproliferative neoplasms	order.Methanobacteriales.id.120	Weighted median	8	-0.258530465	0.23206253	0.265255512	-0.713373023	0.196312094	0.772185506	0.489988664	1.216906635
Myeloproliferative neoplasms	class.Actinobacteria.id.419	Weighted median	12	0.39497682	0.357475426	0.26920023	-0.305675016	1.095628655	1.484349783	0.736625973	2.991062438
Myeloproliferative neoplasms	order.Erysipelotrichales.id.2148	Weighted median	11	-0.520522783	0.476270704	0.274431688	-1.454013362	0.412967797	0.594209824	0.233630759	1.511296357
Myeloproliferative neoplasms	genus.Butyricicoccus.id.2055	Weighted median	7	0.561081097	0.515467223	0.276378696	-0.449234661	1.571396854	1.75256617	0.63811634	4.813367069
Myeloproliferative neoplasms	genus..Eubacteriumoxidoreducensgroup.id.11339	Weighted median	3	-0.493375969	0.456819119	0.280131145	-1.388741442	0.401989504	0.610561674	0.249388978	1.494795644
Myeloproliferative neoplasms	family.Methanobacteriaceae.id.121	Weighted median	8	-0.258530465	0.239995433	0.281377184	-0.728921514	0.211860585	0.772185506	0.482429002	1.235975559
Myeloproliferative neoplasms	family.Streptococcaceae.id.1850	Weighted median	9	-0.574386	0.543398837	0.290500309	-1.639447721	0.490675722	0.563050476	0.194087203	1.633419584
Myeloproliferative neoplasms	genus.Eisenbergiella.id.11304	Weighted median	11	-0.27543238	0.263744131	0.296338926	-0.792370877	0.241506118	0.759243771	0.45277006	1.273165244
Myeloproliferative neoplasms	family.BacteroidalesS24.7group.id.11173	Weighted median	7	0.374984201	0.363077101	0.301699861	-0.336646916	1.086615318	1.454968427	0.714160954	2.96422412
Myeloproliferative neoplasms	genus.Alistipes.id.968	Weighted median	12	0.501429676	0.487114769	0.303297792	-0.453315271	1.456174622	1.651080093	0.635517742	4.289519072
Myeloproliferative neoplasms	genus.Oxalobacter.id.2978	Weighted median	10	0.248078529	0.24132075	0.303948273	-0.224910141	0.7210672	1.281560568	0.798587976	2.056626871
Myeloproliferative neoplasms	family.Erysipelotrichaceae.id.2149	Weighted median	11	-0.520522783	0.519403652	0.316268909	-1.538553941	0.497508376	0.594209824	0.214691333	1.644618391
Myeloproliferative neoplasms	genus.Butyricimonas.id.945	Weighted median	13	-0.34412376	0.351875564	0.33089241	-1.033799866	0.345552346	0.708841196	0.355652956	1.412770041
Myeloproliferative neoplasms	class.Erysipelotrichia.id.2147	Weighted median	11	-0.520522783	0.536013405	0.33149833	-1.571109056	0.530063491	0.594209824	0.207814577	1.699040178
Myeloproliferative neoplasms	genus..Eubacteriumxylanophilumgroup.id.14375	Weighted median	7	-0.467138425	0.487398633	0.337844988	-1.422439746	0.488162896	0.62679332	0.241125015	1.629320238
Myeloproliferative neoplasms	genus.CandidatusSoleaferrea.id.11350	Weighted median	8	-0.313512891	0.327524213	0.338455972	-0.95546035	0.328434567	0.730874957	0.384635037	1.388792364
Myeloproliferative neoplasms	family.Acidaminococcaceae.id.2166	Weighted median	6	-0.405220303	0.429706803	0.345672823	-1.247445638	0.437005031	0.66682989	0.287237569	1.548063866
Myeloproliferative neoplasms	order.Desulfovibrionales.id.3156	Weighted median	10	0.468857043	0.498031974	0.346489892	-0.507285626	1.444999711	1.598166513	0.602127763	4.241850917
Myeloproliferative neoplasms	genus.Defluviitaleaceae UCG011.id.11287	Weighted median	8	-0.356116664	0.382958873	0.352418525	-1.106716055	0.394482727	0.700390905	0.330642994	1.483616557
Myeloproliferative neoplasms	phylum.Verrucomicrobia.id.3982	Weighted median	12	-0.324399849	0.366516067	0.376107882	-1.042771341	0.393971643	0.722961109	0.352476494	1.482858499
Myeloproliferative neoplasms	genus.Coprobacter.id.949	Weighted median	10	0.250971408	0.286157157	0.380464655	-0.30989662	0.811839436	1.285273336	0.733522784	2.252046676
Myeloproliferative neoplasms	phylum.Euryarchaeota.id.55	Weighted median	11	-0.170968959	0.201795751	0.396862667	-0.56648863	0.224550712	0.842847736	0.567514698	1.251760189
Myeloproliferative neoplasms	family.Oxalobacteraceae.id.2966	Weighted median	14	0.174358129	0.209721928	0.405760307	-0.23669685	0.585413107	1.190481835	0.789230507	1.795732662
Myeloproliferative neoplasms	class.Deltaproteobacteria.id.3087	Weighted median	11	0.392902338	0.474504751	0.407655696	-0.537126975	1.32293165	1.481273718	0.58442491	3.754411882
Myeloproliferative neoplasms	family.Verrucomicrobiaceae.id.4036	Weighted median	10	-0.330764696	0.401679782	0.410250046	-1.118057069	0.456527677	0.718374185	0.32691435	1.578583107
Myeloproliferative neoplasms	genus.Hungatella.id.11306	Weighted median	4	-0.358033687	0.43496059	0.410428087	-1.210556443	0.494489068	0.699049525	0.298031396	1.639660271
Myeloproliferative neoplasms	genus.Odoribacter.id.952	Weighted median	5	0.605365156	0.736042932	0.410815695	-0.837278991	2.048009304	1.831921025	0.432886811	7.752452958
Myeloproliferative neoplasms	order.Verrucomicrobiales.id.4030	Weighted median	10	-0.330342213	0.408113534	0.418263801	-1.13024474	0.469560314	0.718677751	0.322954207	1.599290853
Myeloproliferative neoplasms	genus.Ruminococcus2.id.11374	Weighted median	13	0.278046389	0.346183519	0.421872857	-0.400473308	0.956566086	1.320547455	0.670002853	2.602743513
Myeloproliferative neoplasms	class.Verrucomicrobiae.id.4029	Weighted median	10	-0.330342213	0.414036087	0.424952607	-1.141852944	0.481168518	0.718677751	0.319226964	1.617963918
Myeloproliferative neoplasms	genus.Catenibacterium.id.2153	Weighted median	4	0.252791044	0.317517745	0.425946152	-0.369543736	0.875125825	1.287614194	0.691049559	2.399177151

Myeloproliferative neoplasms	genus.Oscillibacter.id.2063	Weighted median	13	-0.265060857	0.333575479	0.426843111	-0.918868795	0.388747081	0.767159262	0.398970103	1.475131416
Myeloproliferative neoplasms	order.Coriobacteriales.id.810	Weighted median	12	0.309677043	0.393461329	0.431247561	-0.461507161	1.080861247	1.362984858	0.630332916	2.94721674
Myeloproliferative neoplasms	genus.Akkermansia.id.4037	Weighted median	10	-0.329889396	0.423009808	0.43547194	-1.158988621	0.499209828	0.719003254	0.313803395	1.647419013
Myeloproliferative neoplasms	genus.Parasutterella.id.2892	Weighted median	13	0.24108572	0.314742418	0.443689483	-0.375809419	0.85798086	1.272630121	0.686733199	2.358393955
Myeloproliferative neoplasms	order.Pasteurellales.id.3688	Weighted median	10	-0.272563269	0.357857005	0.446266453	-0.973962999	0.42883646	0.761425253	0.377583705	1.535469903
Myeloproliferative neoplasms	genus.Peptococcus.id.2037	Weighted median	11	0.186429259	0.245494664	0.447611825	-0.294740283	0.667598801	1.20493938	0.74472498	1.949550436
Myeloproliferative neoplasms	genus.Dialister.id.2183	Weighted median	11	0.296950898	0.392380212	0.449173428	-0.472114316	1.066016113	1.345749219	0.623682212	2.903788062
Myeloproliferative neoplasms	family.Pasteurellaceae.id.3689	Weighted median	10	-0.272563269	0.363258822	0.453057176	-0.984550561	0.439424022	0.761425253	0.373607103	1.55181315
Myeloproliferative neoplasms	family.Prevotellaceae.id.960	Weighted median	13	-0.3142777	0.419828033	0.454106324	-1.137140645	0.508585244	0.730316191	0.320734807	1.662936881
Myeloproliferative neoplasms	genus.Anaerofilum.id.2053	Weighted median	9	-0.202908996	0.276118595	0.462423691	-0.744101443	0.338283451	0.816352529	0.475161068	1.402537997
Myeloproliferative neoplasms	genus.Anaerostipes.id.1991	Weighted median	12	0.319556631	0.438429104	0.466082848	-0.539764413	1.178877675	1.376517324	0.582885557	3.250723785
Myeloproliferative neoplasms	family.Rikenellaceae.id.967	Weighted median	16	0.275931429	0.381541843	0.469556426	-0.471890584	1.023753441	1.317757501	0.623821766	2.783623347
Myeloproliferative neoplasms	genus.Terrisporobacter.id.11348	Weighted median	5	-0.309156716	0.429348159	0.471487652	-1.150679107	0.532365675	0.734065721	0.316421812	1.702956188
Myeloproliferative neoplasms	class.Coriobacteriia.id.809	Weighted median	12	0.309677043	0.430446944	0.471875162	-0.533998967	1.153353053	1.362984858	0.586255858	3.168800272
Myeloproliferative neoplasms	family.Coriobacteriaceae.id.811	Weighted median	12	0.309677043	0.430598634	0.472031283	-0.53429628	1.153650366	1.362984858	0.586081583	3.169742537
Myeloproliferative neoplasms	genus.Bifidobacterium.id.436	Weighted median	12	0.224906659	0.313899312	0.47368699	-0.390335993	0.84014931	1.252205828	0.676829427	2.316712859
Myeloproliferative neoplasms	genus.Enterorhabdus.id.820	Weighted median	6	0.26486047	0.379341029	0.485044604	-0.478647946	1.008368886	1.303249121	0.619620586	2.741126278
Myeloproliferative neoplasms	genus..Eubacteriumbrachygroup.id.11296	Weighted median	6	0.193300709	0.277926441	0.486735543	-0.351435116	0.738036534	1.213247573	0.703677506	2.091824254
Myeloproliferative neoplasms	genus.Bilophila.id.3170	Weighted median	13	-0.279488988	0.40333229	0.48834112	-1.070020275	0.5110423	0.756170055	0.343001563	1.667027832
Myeloproliferative neoplasms	genus.Eggerthella.id.819	Weighted median	10	0.18609312	0.271179596	0.492564489	-0.345418888	0.717605129	1.204534422	0.70792375	2.049518994
Myeloproliferative neoplasms	genus.Holdemanella.id.11393	Weighted median	9	-0.201733392	0.295047135	0.494143932	-0.780025777	0.376558992	0.817312801	0.458394195	1.457261504
Myeloproliferative neoplasms	genus.RuminococcaceaeNK4A214group.id.11358	Weighted median	11	-0.289690936	0.424076358	0.494537136	-1.120880597	0.541498725	0.748494865	0.3259926	1.718580613
Myeloproliferative neoplasms	genus.Anaerotruncus.id.2054	Weighted median	13	-0.302494365	0.445662882	0.497295308	-1.175993614	0.571004885	0.738972652	0.308512285	1.770044849
Myeloproliferative neoplasms	family.Peptostreptococcaceae.id.2042	Weighted median	12	-0.234729411	0.355362159	0.508910083	-0.931239243	0.461780421	0.790784798	0.394065065	1.586896816
Myeloproliferative neoplasms	genus.LachnospiraceaeNC2004group.id.11316	Weighted median	8	-0.204918179	0.311118588	0.510120711	-0.814710612	0.404874254	0.814713974	0.44276744	1.499113981
Myeloproliferative neoplasms	genus.Blautia.id.1992	Weighted median	11	-0.320953353	0.492480346	0.514589598	-1.286214832	0.644308126	0.725457091	0.276314704	1.904668782
Myeloproliferative neoplasms	genus.Prevotella9.id.11183	Weighted median	13	0.210367912	0.335265227	0.530353071	-0.446751932	0.867487757	1.234132029	0.639702578	2.380921879
Myeloproliferative neoplasms	genus.RuminococcaceaeUCG014.id.11371	Weighted median	10	-0.295786762	0.471873568	0.530767546	-1.220658955	0.629085431	0.743946049	0.295035688	1.87589416
Myeloproliferative neoplasms	class.Mollicutes.id.3920	Weighted median	10	0.234545556	0.374606649	0.531241767	-0.499683477	0.968774588	1.264334069	0.606722671	2.634713871
Myeloproliferative neoplasms	phylum.Tenericutes.id.3919	Weighted median	10	0.234545556	0.37543013	0.532142876	-0.501297498	0.97038861	1.264334069	0.605744197	2.63896979
Myeloproliferative neoplasms	genus.Intestinimonas.id.2062	Weighted median	14	0.238496042	0.381987555	0.532394124	-0.510199566	0.987191649	1.269338682	0.600375752	2.683687145
Myeloproliferative neoplasms	genus..Ruminococcusgnavusgroup.id.14376	Weighted median	11	-0.172112196	0.276355384	0.533420566	-0.713768749	0.369544357	0.841884712	0.489794801	1.447075115
Myeloproliferative neoplasms	genus.Ruminiclostridium5.id.11355	Weighted median	10	0.296155245	0.478088256	0.535615165	-0.640897737	1.233208226	1.344678895	0.526819267	3.43222324
Myeloproliferative neoplasms	genus..Ruminococcusgauvreauiigroup.id.11342	Weighted median	9	-0.253758445	0.416409405	0.54226164	-1.06992088	0.562403989	0.775879197	0.343035657	1.754886161
Myeloproliferative neoplasms	family.Alcaligenaceae.id.2875	Weighted median	11	-0.268115147	0.440770358	0.542996738	-1.13202505	0.595794755	0.764819709	0.32237976	1.814472433
Myeloproliferative neoplasms	family.Clostridiaceae1.id.1869	Weighted median	7	-0.286935365	0.48035053	0.550276542	-1.228422404	0.654551674	0.75056024	0.292754061	1.92427962
Myeloproliferative neoplasms	genus.Lachnospira.id.2004	Weighted median	5	-0.388882678	0.658068642	0.554556964	-1.678697215	0.90093186	0.677813788	0.186616939	2.461896186
Myeloproliferative neoplasms	genus.FamilyXIIIAD3011group.id.11293	Weighted median	12	-0.237664138	0.403255221	0.555617074	-1.028044372	0.552716095	0.788467463	0.357705817	1.737967097
Myeloproliferative neoplasms	family.Veillonellaceae.id.2172	Weighted median	18	-0.208363946	0.353587859	0.555670248	-0.901396149	0.484668257	0.811911491	0.406002424	1.623636289
Myeloproliferative neoplasms	genus..Eubacteriumruminantiumgroup.id.11340	Weighted median	16	0.146474418	0.249920987	0.557819803	-0.343730716	0.636319553	1.157745313	0.709375186	1.88951381
Myeloproliferative neoplasms	genus.Oscillospira.id.2064	Weighted median	7	-0.262358744	0.459147015	0.56772537	-1.162286894	0.637569405	0.769235015	0.31277009	1.891876899
Myeloproliferative neoplasms	genus.Escherichia.Shigella.id.3504	Weighted median	8	0.25564855	0.450548945	0.570431778	-0.627427383	1.138724483	1.291298822	0.53396372	3.122782662
Myeloproliferative neoplasms	genus.Tyzzerella3.id.11335	Weighted median	12	-0.149853934	0.269168382	0.577712373	-0.677423962	0.377716095	0.860833706	0.507923739	1.458948681
Myeloproliferative neoplasms	genus.RuminococcaceaeUCG003.id.11361	Weighted median	14	-0.223160913	0.40090463	0.5777711	-1.008933989	0.562612162	0.79998611	0.364607448	1.755251519
Myeloproliferative neoplasms	family.Ruminococcaceae.id.2050	Weighted median	7	-0.253275335	0.466252267	0.586981618	-1.167129779	0.660579109	0.776254123	0.311259043	1.935913115
Myeloproliferative neoplasms	genus.Gordonibacter.id.821	Weighted median	10	0.105015553	0.211069449	0.618808103	-0.308680566	0.518711673	1.110727886	0.73441533	1.679862043
Myeloproliferative neoplasms	genus..Eubacteriumventriosumgroup.id.11341	Weighted median	13	-0.196976111	0.398301588	0.620924787	-0.977647224	0.583695002	0.821210251	0.376195161	1.792650053
Myeloproliferative neoplasms	genus.Actinomyces.id.423	Weighted median	5	-0.254831134	0.519523901	0.623773783	-1.27309798	0.763435711	0.775047366	0.279962957	2.145635355
Myeloproliferative neoplasms	genus.Intestinibacter.id.11345	Weighted median	14	-0.153487832	0.316032218	0.627200114	-0.77291098	0.465935316	0.857711201	0.461667206	1.593503922
Myeloproliferative neoplasms	class.Betaproteobacteria.id.2867	Weighted median	10	0.214845481	0.449484153	0.632662785	-0.66614346	1.095834422	1.239670329	0.513685813	2.991677963
Myeloproliferative neoplasms	genus.Parabacteroides.id.954	Weighted median	5	0.279018334	0.58930473	0.635877629	-0.876018937	1.434055604	1.321831578	0.41643748	4.195680753
Myeloproliferative neoplasms	genus.LachnospiraceaeUCG001.id.11321	Weighted median	11	-0.145323104	0.309225756	0.638385056	-0.751405586	0.460759377	0.864742846	0.471703067	1.585277352
Myeloproliferative neoplasms	genus.LachnospiraceaeFCS020group.id.11314	Weighted median	11	-0.181053089	0.38553461	0.638629637	-0.936700925	0.574594747	0.83439106	0.391918674	1.776410486
Myeloproliferative neoplasms	phylum.Bacteroidetes.id.905	Weighted median	10	-0.241368728	0.515657574	0.639727754	-1.252057572	0.769320116	0.785551918	0.285915899	2.158298363
Myeloproliferative neoplasms	family.ClostridialesvadinBB60group.id.11286	Weighted median	14	0.144849485	0.314495069	0.645100519	-0.47156044	0.76126041	1.155866056	0.62402775	2.14097264
Myeloproliferative neoplasms	genus.Marvinbryantia.id.2005	Weighted median	9	0.214187519	0.466578181	0.646190866	-0.700305715	1.128680753	1.238854942	0.496433513	3.091575257
Myeloproliferative neoplasms	genus.Ruminococcus1.id.11373	Weighted median	8	-0.219260911	0.484291552	0.650731878	-1.168472353	0.729950531	0.80311215	0.310841435	2.074977959
Myeloproliferative neoplasms	order.Gastranaerophilales.id.1591	Weighted median	9	0.148390191	0.329404557	0.652364315	-0.497242741	0.794023124	1.159965416	0.608205329	2.212278819
Myeloproliferative neoplasms	genus.Fusicatenibacter.id.11305	Weighted median	18	0.168610907	0.391402338	0.666623498	-0.598537676	0.93575949	1.183659496	0.549614763	2.549148777
Myeloproliferative neoplasms	family.Actinomycetaceae.id.421	Weighted median	3	-0.278456691	0.646581024	0.666715487	-1.545755499	0.988842117	0.75695105	0.213150775	2.68812014
Myeloproliferative neoplasms	phylum.Actinobacteria.id.400	Weighted median	14	0.163026152	0.385981084	0.672756254	-0.593496772	0.919549077	1.177067472	0.552392313	2.508159148
Myeloproliferative neoplasms	order.Burkholderiales.id.2874	Weighted median	10	0.188130628	0.447036598	0.673872335	-0.688061104	1.064322361	1.206991172	0.502549516	2.898873928
Myeloproliferative neoplasms	order.Enterobacteriales.id.3468	Weighted median	5	-0.255046617	0.612010835	0.676871913	-1.454587853	0.944494618	0.774880374	0.233496579	2.571513454
Myeloproliferative neoplasms	order.Actinomycetales.id.420	Weighted median	3	-0.278423521	0.67628597	0.680563024	-1.603944022	1.04709698	0.756976158	0.201101802	2.849367329
Myeloproliferative neoplasms	family.Enterobacteriaceae.id.3469	Weighted median	5	-0.255046617	0.625933652	0.683665836	-1.481876576	0.971783341	0.774880374	0.22721091	2.642653012
Myeloproliferative neoplasms	genus.RuminococcaceaeUCG005.id.11363	Weighted median	13	-0.161849274	0.405503807	0.689796438	-0.956636737	0.632938188	0.850569398	0.384182824	1.883135465
Myeloproliferative neoplasms	genus.Haemophilus.id.3698	Weighted median	8	-0.143604389	0.36070542	0.690541022	-0.850587013	0.563378234	0.86623037	0.427164107	1.756596683
Myeloproliferative neoplasms	family.Defluviitaleaceae.id.1924	Weighted median	10	0.140343387	0.360798759	0.697291177	-0.566822181	0.847508955	1.150668855	0.567325434	2.333825938
Myeloproliferative neoplasms	genus.Flavonifractor.id.2059	Weighted median	4	0.202333531	0.535085855	0.705331918	-0.846434745	1.251101806	1.224256267	0.428941495	3.494190758
Myeloproliferative neoplasms	class.Gammaproteobacteria.id.3303	Weighted median	6	0.226611181	0.624802533	0.716834617	-0.998001785	1.451224146	1.254342061	0.368615278	4.268336387
Myeloproliferative neoplasms	family.Lactobacillaceae.id.1836	Weighted median	7	-0.125139284	0.350137271	0.720791899	-0.811408336	0.561129768	0.882373993	0.444231998	1.752651471
Myeloproliferative neoplasms	class.Alphaproteobacteria.id.2379	Weighted median	6	0.14367824	0.420490578	0.732582741	-0.680483294	0.967839773	1.154512573	0.506372207	2.632252052
Myeloproliferative neoplasms	genus.Lachnoclostridium.id.11308	Weighted median	13	-0.135342513	0.399281386	0.734635912	-0.91793403	0.647249003	0.873416704	0.399343221	1.910278424
Myeloproliferative neoplasms	genus.ChristensenellaceaeR.7group.id.11283	Weighted median	6	-0.204513841	0.609885873	0.737375163	-1.399890152	0.990862471	0.815043461	0.246624054	2.693556585
Myeloproliferative neoplasms	genus.LachnospiraceaeND3007group.id.11317	Weighted median	3	0.245597718	0.771707523	0.750293541	-1.266949027	1.758144462	1.278385198	0.28168974	5.801662195
Myeloproliferative neoplasms	genus.Erysipelatoclostridium.id.11381	Weighted median	14	0.100514057	0.329820329	0.760553537	-0.545933789	0.746961902	1.105739184	0.579300586	2.110578123
Myeloproliferative neoplasms	genus.Barnesiella.id.944	Weighted median	10	0.132166355	0.436287232	0.761939953	-0.722956619	0.987289329	1.141298164	0.48531524	2.683949299
Myeloproliferative neoplasms	phylum.Proteobacteria.id.2375	Weighted median	10	-0.138188201	0.458977282	0.763354619	-1.037783673	0.761407271	0.870934765	0.354238922	2.141287473
Myeloproliferative neoplasms	genus.Holdemania.id.2157	Weighted median	12	-0.092023837	0.320355328	0.773916676	-0.71992028	0.535872606	0.912083408	0.486791061	1.708938822
Myeloproliferative neoplasms	genus.Sellimonas.id.14369	Weighted median	7	0.069892559	0.245131448	0.775550081	-0.410565078	0.550350197	1.072392956	0.663275342	1.733860103
Myeloproliferative neoplasms	family.Bacteroidaceae.id.917	Weighted median	7	-0.145643609	0.537315395	0.786346463	-1.198781783	0.907494565	0.864465736	0.301561355	2.478106016
Myeloproliferative neoplasms	genus.Bacteroides.id.918	Weighted median	7	-0.145643609	0.543949409	0.788890126	-1.211784451	0.920497233	0.864465736	0.297665636	2.510538402
Myeloproliferative neoplasms	phylum.Cyanobacteria.id.1500	Weighted median	7	-0.108267452	0.411069132	0.792257584	-0.913962952	0.697428047	0.897387556	0.400932197	2.008580085
Myeloproliferative neoplasms	genus..Eubacteriumrectalegroup.id.14374	Weighted median	8	0.116869151	0.460527093	0.799671254	-0.785763951	1.019502252	1.123972349	0.455771382	2.771814755
Myeloproliferative neoplasms	genus.RikenellaceaeRC9gutgroup.id.11191	Weighted median	10	0.050243217	0.19920132	0.800868458	-0.34019137	0.440677804	1.051526815	0.711634124	1.553760007
Myeloproliferative neoplasms	genus.Subdoligranulum.id.2070	Weighted median	10	-0.119412388	0.474051363	0.801120235	-1.04855306	0.809728283	0.887441755	0.350444455	2.247297275
Myeloproliferative neoplasms	family.FamilyXIII.id.1957	Weighted median	5	0.174960488	0.722369327	0.808622367	-1.240883394	1.590804369	1.191199149	0.289128691	4.90769494
Myeloproliferative neoplasms	phylum.Firmicutes.id.1672	Weighted median	13	-0.094608621	0.397971487	0.812092737	-0.874632735	0.685415493	0.909728914	0.417015147	1.98459625
Myeloproliferative neoplasms	genus.RuminococcaceaeUCG011.id.11368	Weighted median	8	-0.049489245	0.227725583	0.827959239	-0.495831387	0.396852896	0.951715393	0.609064328	1.487137151
Myeloproliferative neoplasms	genus..Eubacteriumcoprostanoligenesgroup.id.11375	Weighted median	12	-0.100688078	0.48223619	0.834608966	-1.04587101	0.844494854	0.904215033	0.351385626	2.326802142
Myeloproliferative neoplasms	class.Lentisphaeria.id.2250	Weighted median	8	-0.054398189	0.265241959	0.837502646	-0.574272428	0.46547605	0.947054924	0.563114426	1.592772247
Myeloproliferative neoplasms	order.Victivallales.id.2254	Weighted median	8	-0.054398189	0.277271346	0.844460541	-0.597850028	0.489053649	0.947054924	0.549992835	1.630772207
Myeloproliferative neoplasms	genus.Coprococcus1.id.11301	Weighted median	9	-0.101237208	0.535244986	0.849981669	-1.150317381	0.947842964	0.903718638	0.316536291	2.580138203
Myeloproliferative neoplasms	family.Porphyromonadaceae.id.943	Weighted median	9	-0.100836936	0.534147631	0.850264446	-1.147766294	0.946092421	0.904080444	0.317344833	2.575625511
Myeloproliferative neoplasms	genus.Olsenella.id.822	Weighted median	9	-0.042038101	0.225517515	0.852125402	-0.484052431	0.399976228	0.958833247	0.616280889	1.491789235
Myeloproliferative neoplasms	order.Rhodospirillales.id.2667	Weighted median	12	0.061252442	0.333775737	0.854394919	-0.592948003	0.715452888	1.063167269	0.552695532	2.045112679
Myeloproliferative neoplasms	family.Rhodospirillaceae.id.2717	Weighted median	13	-0.057937636	0.328294928	0.859916507	-0.701395696	0.585520423	0.943708799	0.495892705	1.795925383
Myeloproliferative neoplasms	genus.LachnospiraceaeUCG010.id.11330	Weighted median	10	0.073711691	0.420642212	0.860894138	-0.750747044	0.898170426	1.076496397	0.472013806	2.455107199
Myeloproliferative neoplasms	genus.Clostridiumsensustricto1.id.1873	Weighted median	5	0.092294237	0.527849691	0.861197919	-0.942291117	1.126879631	1.09668746	0.38973387	3.086011963
Myeloproliferative neoplasms	genus..Eubacteriumnodatumgroup.id.11297	Weighted median	11	-0.035325102	0.203783296	0.86237915	-0.434740362	0.364090158	0.965291547	0.647432743	1.439203964
Myeloproliferative neoplasms	genus.Lactobacillus.id.1837	Weighted median	7	0.0579176	0.349141354	0.868246806	-0.626399455	0.742234655	1.059627679	0.534512878	2.100624444
Myeloproliferative neoplasms	genus.Ruminiclostridium6.id.11356	Weighted median	12	0.067118531	0.408689549	0.869551151	-0.733912985	0.868150048	1.069422231	0.480026972	2.382499265
Myeloproliferative neoplasms	genus.Butyrivibrio.id.1993	Weighted median	13	0.030833917	0.188570023	0.870113425	-0.338763329	0.400431163	1.031314206	0.712651093	1.492468056
Myeloproliferative neoplasms	order.Bacteroidales.id.913	Weighted median	12	-0.069819084	0.457725298	0.878765064	-0.966960667	0.827322499	0.93256252	0.38023695	2.28718659
Myeloproliferative neoplasms	class.Bacteroidia.id.912	Weighted median	12	-0.069819084	0.46131641	0.879701603	-0.973999248	0.83436108	0.93256252	0.377570019	2.303341926
Myeloproliferative neoplasms	order.Selenomonadales.id.2165	Weighted median	12	0.061475124	0.406971025	0.879932106	-0.736188086	0.859138334	1.063404043	0.478936104	2.361125315
Myeloproliferative neoplasms	genus.Veillonella.id.2198	Weighted median	6	-0.064739305	0.441234273	0.883350504	-0.929558481	0.80007987	0.937311784	0.394727952	2.225718689
Myeloproliferative neoplasms	class.Negativicutes.id.2164	Weighted median	12	0.061475124	0.43100656	0.886581197	-0.783297734	0.906247982	1.063404043	0.4568968	2.475018775
Myeloproliferative neoplasms	genus.LachnospiraceaeUCG004.id.11324	Weighted median	12	-0.062149875	0.447111849	0.889447803	-0.938489099	0.81418935	0.939742033	0.391218481	2.257345014
Myeloproliferative neoplasms	genus.RuminococcaceaeUCG002.id.11360	Weighted median	17	-0.048664403	0.385293873	0.899490785	-0.803840395	0.706511589	0.952500732	0.447606673	2.026908222
Myeloproliferative neoplasms	class.Bacilli.id.1673	Weighted median	14	0.047871463	0.381761823	0.900209936	-0.700371710	0.796124637	1.049035807	0.496395788	2.216932839
Myeloproliferative neoplasms	family.Victivallaceae.id.2255	Weighted median	9	-0.02799262	0.225733044	0.901309234	-0.470429386	0.414444147	0.972395543	0.624733959	1.513529206
Myeloproliferative neoplasms	genus.Howardella.id.2000	Weighted median	9	0.028484538	0.248876128	0.908878961	-0.459312673	0.51628175	1.028894102	0.631717693	1.675785063
Myeloproliferative neoplasms	genus.Methanobrevibacter.id.123	Weighted median	6	0.031956347	0.294268326	0.913522978	-0.544809572	0.608722266	1.032472433	0.579952212	1.838081318
Myeloproliferative neoplasms	phylum.Lentisphaerae.id.2238	Weighted median	9	-0.025174657	0.245128097	0.91820107	-0.505625727	0.455276414	0.975139583	0.603128064	1.576609119
Myeloproliferative neoplasms	order.Lactobacillales.id.1800	Weighted median	11	-0.040634297	0.432779902	0.925195451	-0.888882905	0.807614311	0.960180207	0.411114751	2.242551568
Myeloproliferative neoplasms	genus.Desulfovibrio.id.3173	Weighted median	8	0.036431435	0.39424699	0.926374167	-0.736292666	0.809155536	1.037103193	0.478886019	2.24601051
Myeloproliferative neoplasms	order.Bacillales.id.1674	Weighted median	8	0.020209342	0.220767469	0.92706249	-0.412494898	0.452913582	1.020414933	0.661996574	1.572888254
Myeloproliferative neoplasms	genus.Adlercreutzia.id.812	Weighted median	7	-0.03288112	0.400930161	0.934637099	-0.818704235	0.752941994	0.967653587	0.44100272	2.123237389
Myeloproliferative neoplasms	genus.Prevotella7.id.11182	Weighted median	9	-0.018986584	0.240047769	0.936957041	-0.489480211	0.451507043	0.981192526	0.612944913	1.570677481
Myeloproliferative neoplasms	genus.Turicibacter.id.2162	Weighted median	8	-0.026719575	0.383885578	0.944509675	-0.779135309	0.725696158	0.973634234	0.458802562	2.06616898
Myeloproliferative neoplasms	genus..Eubacteriumeligensgroup.id.14372	Weighted median	6	-0.033271189	0.541490585	0.951005845	-1.094592736	1.028050359	0.96727621	0.334675881	2.795610081
Myeloproliferative neoplasms	genus.FamilyXIIIUCG001.id.11294	Weighted median	7	0.028178379	0.481488244	0.953331641	-0.915538578	0.971895337	1.028579145	0.400300975	2.642948995
Myeloproliferative neoplasms	genus.Paraprevotella.id.962	Weighted median	13	-0.007208494	0.246132275	0.976635636	-0.489627754	0.475210765	0.992817425	0.612854485	1.608353147
Myeloproliferative neoplasms	genus..Clostridiuminnocuumgroup.id.14397	Weighted median	6	0.007192983	0.248904746	0.976945512	-0.480660318	0.495046285	1.007218915	0.618374933	1.640574171
Myeloproliferative neoplasms	family.Peptococcaceae.id.2024	Weighted median	8	0.009223715	0.369420369	0.98008043	-0.714840209	0.733287639	1.009266384	0.489270287	2.081913949
Myeloproliferative neoplasms	genus.ErysipelotrichaceaeUCG003.id.11384	Weighted median	12	0.003560432	0.353689697	0.991968198	-0.689671374	0.696792238	1.003566778	0.501740927	2.007303417
Myeloproliferative neoplasms	genus.Coprococcus2.id.11302	Weighted median	6	0.004006911	0.516251829	0.993807247	-1.007846674	1.015860495	1.004014949	0.365004107	2.761738838
Myeloproliferative neoplasms	genus.LachnospiraceaeNK4A136group.id.11319	Weighted median	13	0.00267285	0.377937382	0.994357244	-0.738084419	0.74343012	1.002676426	0.478028742	2.103137168

Myeloproliferative neoplasms	family.FamilyXI.id.1936	Weighted median	8	-0.001408187	0.226405429	0.995037382	-0.445162828	0.442346453	0.998592804	0.640719941	1.556354851
Myeloproliferative neoplasms	genus.Roseburia.id.2012	Weighted median	11	0.002277095	0.461262715	0.996061135	-0.901797827	0.906352017	1.00227969	0.405839375	2.475276277
Myeloproliferative neoplasms	genus.Allisonella.id.2174	Weighted median	8	4.98477E-05	0.230950289	0.999827787	-0.452612718	0.452712413	1.000049849	0.635964383	1.572571871
Myeloproliferative neoplasms	genus..Eubacteriumhalliigroup.id.11338	Weighted mode	13	-1.198039447	0.619686393	0.077137859	-2.412624776	0.016545883	0.301785298	0.089579859	1.016683524
Myeloproliferative neoplasms	genus.Phascolarctobacterium.id.2168	Weighted mode	7	1.075409702	0.580570151	0.113422639	-0.062507794	2.213327197	2.93119357	0.939405741	9.146096693
Myeloproliferative neoplasms	family.Christensenellaceae.id.1866	Weighted mode	10	0.821829361	0.52897343	0.154691701	-0.214958562	1.858617284	2.274657202	0.806574862	6.414860704
Myeloproliferative neoplasms	genus.RuminococcaceaeUCG013.id.11370	Weighted mode	11	-0.943699567	0.651165392	0.177892773	-2.219983736	0.332584602	0.389185352	0.108610875	1.394567878
Myeloproliferative neoplasms	genus.LachnospiraceaeUCG008.id.11328	Weighted mode	10	0.661430231	0.453201973	0.178442169	-0.226845636	1.549706098	1.937561514	0.797043807	4.710085678
Myeloproliferative neoplasms	genus.Ruminiclostridium9.id.11357	Weighted mode	7	1.118081834	0.779951125	0.201693815	-0.41062237	2.646786039	3.05898094	0.663237342	14.10862114
Myeloproliferative neoplasms	genus.Butyricicoccus.id.2055	Weighted mode	7	1.018469178	0.747347949	0.221880796	-0.446332802	2.483271159	2.768952745	0.639970753	11.98039015
Myeloproliferative neoplasms	order.NB1n.id.3953	Weighted mode	12	-0.444339341	0.361187936	0.2442765	-1.152267694	0.263589013	0.641247782	0.315919547	1.301553149
Myeloproliferative neoplasms	family.Prevotellaceae.id.960	Weighted mode	13	-0.97962705	0.803034977	0.245933733	-2.553575605	0.594321505	0.375451097	0.077802975	1.81180123
Myeloproliferative neoplasms	genus..Ruminococcustorquesgroup.id.14377	Weighted mode	10	0.838263521	0.682692967	0.250636631	-0.499814695	2.176341736	2.312348144	0.606643063	8.81400326
Myeloproliferative neoplasms	genus.Collinsella.id.815	Weighted mode	9	-0.935653559	0.78353995	0.266627612	-2.471391861	0.600084742	0.392329371	0.084467211	1.822273217
Myeloproliferative neoplasms	genus.Coprococcus3.id.11303	Weighted mode	5	-1.120067893	0.887301043	0.275412138	-2.859177938	0.619042151	0.326257643	0.057315858	1.857148323
Myeloproliferative neoplasms	order.Bifidobacteriales.id.432	Weighted mode	9	0.55165694	0.472186344	0.276319196	-0.37382829	1.47714218	1.736127304	0.688095055	4.380409357
Myeloproliferative neoplasms	family.Bifidobacteriaceae.id.433	Weighted mode	10	0.513057655	0.455522956	0.289171469	-0.379767338	1.405882649	1.670390874	0.684020536	4.079125588
Myeloproliferative neoplasms	genus.RuminococcaceaeUCG010.id.11367	Weighted mode	6	0.676533724	0.57473123	0.292121343	-0.449939487	1.803006935	1.967047572	0.637666738	6.067865738
Myeloproliferative neoplasms	genus.Coprobacter.id.949	Weighted mode	10	0.532910094	0.477499313	0.293313342	-0.40298856	1.468808748	1.703883563	0.668319745	4.344057192
Myeloproliferative neoplasms	genus.Senegalimassilia.id.11160	Weighted mode	5	-0.742193663	0.631025505	0.304746538	-1.979003653	0.494616327	0.476068435	0.138206871	1.639868945
Myeloproliferative neoplasms	genus.Slackia.id.825	Weighted mode	6	-0.588564999	0.523958466	0.312329618	-1.615523593	0.438393595	0.555123316	0.19878656	1.550214945
Myeloproliferative neoplasms	genus.DefluviitaleaceaeUCG011.id.11287	Weighted mode	8	-0.60175384	0.554597045	0.313857146	-1.688764048	0.485256368	0.547849952	0.184747722	1.62459145
Myeloproliferative neoplasms	genus.Lactococcus.id.1851	Weighted mode	9	0.481072642	0.448971823	0.315202761	-0.398912131	1.361057415	1.617808801	0.671049663	3.900315373
Myeloproliferative neoplasms	order.Clostridiales.id.1863	Weighted mode	12	-0.798767441	0.761399794	0.316645336	-2.291111037	0.693576155	0.44988313	0.101154014	2.000858133
Myeloproliferative neoplasms	family.BacteroidalesS24.7group.id.11173	Weighted mode	7	0.542055478	0.511356774	0.329924702	-0.460203798	1.544314755	1.719537705	0.631155004	4.684760319
Myeloproliferative neoplasms	family.Lachnospiraceae.id.1987	Weighted mode	15	-0.77333732	0.772493956	0.333772202	-2.287425474	0.740750834	0.461470421	0.101527511	2.097509806
Myeloproliferative neoplasms	genus.Victivallis.id.2256	Weighted mode	8	-0.342124585	0.330380502	0.334856728	-0.989670369	0.305421198	0.710259711	0.371699194	1.357196532
Myeloproliferative neoplasms	class.Melainabacteria.id.1589	Weighted mode	9	0.444582162	0.434974144	0.336649254	-0.407967161	1.297131484	1.5598383	0.665000717	3.658786315
Myeloproliferative neoplasms	genus.Dorea.id.1997	Weighted mode	10	-0.733096979	0.728517178	0.340564601	-2.160990648	0.69479669	0.480418837	0.115210931	2.003301742
Myeloproliferative neoplasms	family.Streptococcaceae.id.1850	Weighted mode	9	-0.695347121	0.688889705	0.342346461	-2.045570943	0.654876701	0.498901239	0.129306342	1.924905165
Myeloproliferative neoplasms	genus.Romboutsia.id.11347	Weighted mode	11	0.805250626	0.815991865	0.34699778	-0.794093429	2.404594682	2.237257144	0.451990811	11.07394091
Myeloproliferative neoplasms	class.Actinobacteria.id.419	Weighted mode	12	0.472535733	0.482449592	0.348407673	-0.473065467	1.418136933	1.6040565	0.623089278	4.129419885
Myeloproliferative neoplasms	genus.Parasutterella.id.2892	Weighted mode	13	0.449673837	0.463063369	0.350673419	-0.457930366	1.35727804	1.567800743	0.632591525	3.885602439
Myeloproliferative neoplasms	genus.RuminococcaceaeNK4A214group.id.11358	Weighted mode	11	-0.733233789	0.759873264	0.357328766	-2.222585386	0.756117809	0.480353115	0.108328675	2.129991116
Myeloproliferative neoplasms	order.MollicutesRF9.id.11579	Weighted mode	11	0.693391238	0.72015596	0.358334852	-0.718114444	2.10489692	2.000488174	0.48767092	8.206257062
Myeloproliferative neoplasms	genus.Oscillibacter.id.2063	Weighted mode	13	-0.649354049	0.685416949	0.362130765	-1.992771268	0.69406317	0.522383102	0.13631713	2.001832818
Myeloproliferative neoplasms	family.Veillonellaceae.id.2172	Weighted mode	18	-0.379840074	0.406999668	0.363748222	-1.177559424	0.417879275	0.683970785	0.308029592	1.518737314
Myeloproliferative neoplasms	order.Methanobacteriales.id.120	Weighted mode	8	-0.312569681	0.324438154	0.367430628	-0.948468463	0.3233291	0.731564651	0.387333785	1.38172
Myeloproliferative neoplasms	genus.Eggerthella.id.819	Weighted mode	10	0.417968167	0.441776868	0.368801079	-0.447914494	1.283850828	1.518872324	0.638959317	3.610516469
Myeloproliferative neoplasms	genus.RuminococcaceaeUCG009.id.11366	Weighted mode	10	0.486626718	0.517378334	0.371486473	-0.527434817	1.500688253	1.626819234	0.590116787	4.48477467
Myeloproliferative neoplasms	genus.Streptococcus.id.1853	Weighted mode	10	-0.587241576	0.634835196	0.379085352	-1.83151856	0.657035409	0.555858466	0.160170155	1.92906496
Myeloproliferative neoplasms	class.Methanobacteria.id.119	Weighted mode	8	-0.312569681	0.334633256	0.381361548	-0.968450862	0.343311499	0.731564651	0.379670745	1.409607786
Myeloproliferative neoplasms	genus.RuminococcaceaeUCG005.id.11363	Weighted mode	13	-0.596260281	0.656705675	0.381773518	-1.883403404	0.690882843	0.55086788	0.152071663	1.995476448
Myeloproliferative neoplasms	genus.Enterorhabdus.id.820	Weighted mode	6	0.487787268	0.510293226	0.383024251	-0.512387455	1.487961992	1.628708335	0.599063632	4.428061891
Myeloproliferative neoplasms	genus.CandidatusSoleaferrea.id.11350	Weighted mode	8	-0.402368834	0.433655466	0.384360611	-1.252333547	0.447595878	0.668734048	0.285837004	1.5645463
Myeloproliferative neoplasms	family.Methanobacteriaceae.id.121	Weighted mode	8	-0.312569681	0.340545171	0.389226383	-0.980038217	0.354898854	0.731564651	0.375296756	1.426036409
Myeloproliferative neoplasms	genus.LachnospiraceaeUCG001.id.11321	Weighted mode	11	-0.470864078	0.524639696	0.390548411	-1.499157883	0.557429727	0.624462451	0.223318141	1.746178572
Myeloproliferative neoplasms	genus.RuminococcaceaeUCG004.id.11362	Weighted mode	9	-0.461920963	0.517042305	0.397725953	-1.475323881	0.551481954	0.630072137	0.228704642	1.735823524
Myeloproliferative neoplasms	genus..Eubacteriumoxidoreducensgroup.id.11339	Weighted mode	3	-0.536329766	0.502733945	0.39777464	-1.521688299	0.449028766	0.584891004	0.218342947	1.566789727
Myeloproliferative neoplasms	class.Clostridia.id.1859	Weighted mode	11	-0.703966161	0.84742944	0.425527673	-2.364927862	0.956995541	0.494619667	0.093956078	2.603861514
Myeloproliferative neoplasms	genus.Alloprevotella.id.961	Weighted mode	5	-0.304333705	0.346578322	0.429465609	-0.983627216	0.374959806	0.73761468	0.37395223	1.454932935
Myeloproliferative neoplasms	genus.Sutterella.id.2896	Weighted mode	12	-0.510852213	0.634091949	0.437517619	-1.753672433	0.731968008	0.599984047	0.173136941	2.079168404
Myeloproliferative neoplasms	genus.Ruminiclostridium6.id.11356	Weighted mode	12	0.430802118	0.545100059	0.446046235	-0.637593999	1.499198234	1.53849108	0.528562618	4.478097245
Myeloproliferative neoplasms	family.Acidaminococcaceae.id.2166	Weighted mode	6	-0.460626004	0.56127102	0.449169214	-1.560717202	0.639465195	0.630888583	0.209985415	1.895466903
Myeloproliferative neoplasms	genus.RuminococcaceaeUCG003.id.11361	Weighted mode	14	-0.620214494	0.800580307	0.452374438	-2.189351896	0.948922909	0.537829064	0.111989306	2.582926113
Myeloproliferative neoplasms	genus.LachnospiraceaeFCS020group.id.11314	Weighted mode	11	-0.503467919	0.654973805	0.459849659	-1.787216577	0.78028074	0.604430904	0.167425538	2.182084777
Myeloproliferative neoplasms	genus.Holdemania.id.2157	Weighted mode	12	-0.434164809	0.568651829	0.461228103	-1.548722394	0.680392777	0.647805483	0.212519316	1.974653178
Myeloproliferative neoplasms	genus..Eubacteriumfissicatenagroup.id.14373	Weighted mode	8	0.308303133	0.397797931	0.463696555	-0.471380812	1.087987079	1.361113525	0.624139853	2.968293115
Myeloproliferative neoplasms	genus.LachnospiraceaeNC2004group.id.11316	Weighted mode	8	-0.333198662	0.432182177	0.465947296	-1.180275729	0.513878404	0.716627813	0.307194025	1.671762409
Myeloproliferative neoplasms	phylum.Euryarchaeota.id.55	Weighted mode	11	-0.22930327	0.307979709	0.473683267	-0.8329435	0.37433696	0.795087371	0.434767662	1.454027017
Myeloproliferative neoplasms	genus.Erysipelatoclostridium.id.11381	Weighted mode	14	0.459404298	0.634424454	0.481816443	-0.784067633	1.702876229	1.58313063	0.456545171	5.48971438
Myeloproliferative neoplasms	family.ClostridialesvadinBB60group.id.11286	Weighted mode	14	0.308824095	0.426984672	0.482327127	-0.528065863	1.145714053	1.361822798	0.589744514	3.144686028
Myeloproliferative neoplasms	genus.Anaerostipes.id.1991	Weighted mode	12	0.567528857	0.783480635	0.483961791	-0.968093187	2.103150901	1.763902805	0.379806568	8.191941282
Myeloproliferative neoplasms	genus.Subdoligranulum.id.2070	Weighted mode	10	-0.545307651	0.757207335	0.489708223	-2.029434028	0.938818726	0.579663422	0.131409874	2.556959165
Myeloproliferative neoplasms	order.Verrucomicrobiales.id.4030	Weighted mode	10	-0.529442767	0.736538517	0.490489019	-1.97305826	0.914172727	0.588933051	0.139031012	2.494710591
Myeloproliferative neoplasms	phylum.Proteobacteria.id.2375	Weighted mode	10	-0.49929618	0.724632854	0.508174557	-1.919576573	0.920984214	0.606957699	0.146669053	2.511761285
Myeloproliferative neoplasms	class.Verrucomicrobiae.id.4029	Weighted mode	10	-0.529442767	0.785100096	0.51702576	-2.068238954	1.009353421	0.588933051	0.126408196	2.74382634
Myeloproliferative neoplasms	genus.Peptococcus.id.2037	Weighted mode	11	0.273901925	0.40792977	0.517147479	-0.525640425	1.073444275	1.315085818	0.591176639	2.92543818
Myeloproliferative neoplasms	genus.Akkermansia.id.4037	Weighted mode	10	-0.530277651	0.789115974	0.51846684	-2.076944961	1.016389659	0.588441566	0.125312462	2.763200637
Myeloproliferative neoplasms	genus.Butyricimonas.id.945	Weighted mode	13	-0.376153637	0.565598995	0.518593928	-1.484727668	0.732420394	0.686496854	0.226564033	2.080109204
Myeloproliferative neoplasms	genus.Ruminococcus2.id.11374	Weighted mode	13	0.259331391	0.390577919	0.519264428	-0.506201331	1.024864113	1.296063237	0.602781001	2.786716756
Myeloproliferative neoplasms	genus.Anaerotruncus.id.2054	Weighted mode	13	-0.423321286	0.638958654	0.520161666	-1.675680248	0.829037676	0.654868199	0.187180807	2.291112885
Myeloproliferative neoplasms	genus.LachnospiraceaeNK4A136group.id.11319	Weighted mode	13	-0.296056648	0.447282782	0.52054356	-1.172730901	0.580617605	0.743745295	0.309520516	1.787141838
Myeloproliferative neoplasms	class.Deltaproteobacteria.id.3087	Weighted mode	11	0.43722727	0.659706524	0.522466058	-0.855797518	1.730252057	1.548407943	0.424944155	5.642075855
Myeloproliferative neoplasms	phylum.Bacteroidetes.id.905	Weighted mode	10	-0.379529707	0.572813045	0.524207902	-1.502243275	0.743183861	0.6841831	0.222630179	2.102619315
Myeloproliferative neoplasms	family.Peptostreptococcaceae.id.2042	Weighted mode	12	-0.318621038	0.48595435	0.525518431	-1.271091565	0.633849488	0.727151059	0.280525243	1.884852348
Myeloproliferative neoplasms	genus.Faecalibacterium.id.2057	Weighted mode	10	-0.277115901	0.419960385	0.525866781	-1.100238256	0.546006454	0.757966643	0.332791785	1.726344995
Myeloproliferative neoplasms	genus.Tyzzerella3.id.11335	Weighted mode	12	-0.261537451	0.405611629	0.532275242	-1.056536245	0.533461343	0.769867043	0.347657929	1.704823085
Myeloproliferative neoplasms	genus.Oxalobacter.id.2978	Weighted mode	10	0.23774336	0.36933179	0.535818839	-0.486146948	0.961633668	1.268383633	0.614991429	2.615966606
Myeloproliferative neoplasms	family.Verrucomicrobiaceae.id.4036	Weighted mode	10	-0.529210434	0.823619408	0.53654395	-2.143504474	1.085083605	0.589069895	0.117243246	2.959687254
Myeloproliferative neoplasms	genus.RuminococcaceaeUCG002.id.11360	Weighted mode	17	0.435379024	0.690401477	0.537194972	-0.917807871	1.788565919	1.545548748	0.399393604	5.980869265
Myeloproliferative neoplasms	genus.Gordonibacter.id.821	Weighted mode	10	0.243111047	0.379934625	0.538199088	-0.501560818	0.987782912	1.275210225	0.605584714	2.685274379
Myeloproliferative neoplasms	genus.Blautia.id.1992	Weighted mode	11	-0.486872998	0.767705809	0.540190915	-1.991576384	1.017830387	0.614545076	0.136480111	2.767184528
Myeloproliferative neoplasms	phylum.Verrucomicrobia.id.3982	Weighted mode	12	-0.379681902	0.601743992	0.540949043	-1.559100126	0.799736323	0.684078979	0.210325252	2.224954183
Myeloproliferative neoplasms	genus.Ruminiclostridium5.id.11355	Weighted mode	10	0.441434743	0.712154459	0.550720193	-0.954387997	1.837257483	1.554936553	0.385047723	6.279293557
Myeloproliferative neoplasms	phylum.Firmicutes.id.1672	Weighted mode	13	-0.397333265	0.652398167	0.553856692	-1.676033673	0.881367142	0.672109997	0.187114664	2.414198004
Myeloproliferative neoplasms	family.Rikenellaceae.id.967	Weighted mode	16	0.394661954	0.657616811	0.557371762	-0.894266996	1.683590904	1.483882486	0.40890722	5.384857803
Myeloproliferative neoplasms	genus.Bifidobacterium.id.436	Weighted mode	12	0.238148258	0.39479728	0.558597128	-0.535654411	1.011950927	1.268897303	0.585286147	2.750962711
Myeloproliferative neoplasms	genus.Terrisporobacter.id.11348	Weighted mode	5	-0.332491354	0.527489424	0.562696353	-1.366370624	0.701387917	0.717134869	0.255030885	2.016549569
Myeloproliferative neoplasms	family.Coriobacteriaceae.id.811	Weighted mode	12	0.38516898	0.653971666	0.567780087	-0.896615485	1.666953444	1.469862677	0.407948032	5.29600861
Myeloproliferative neoplasms	genus.Intestinimonas.id.2062	Weighted mode	14	0.362558857	0.618596157	0.56784588	-0.849889611	1.575007325	1.437001796	0.427462116	4.830777005
Myeloproliferative neoplasms	order.Coriobacteriales.id.810	Weighted mode	12	0.38516898	0.658918582	0.570646716	-0.906311441	1.676649401	1.469862677	0.4040117	5.347608227
Myeloproliferative neoplasms	family.Defluviitaleaceae.id.1924	Weighted mode	10	0.330772115	0.562107584	0.570700788	-0.770958751	1.432502981	1.39204253	0.462569367	4.189171495
Myeloproliferative neoplasms	genus.RuminococcaceaeUCG014.id.11371	Weighted mode	10	-0.401474817	0.685186881	0.572318173	-1.744441104	0.941491469	0.669332175	0.174742625	2.563802401
Myeloproliferative neoplasms	class.Coriobacteriia.id.809	Weighted mode	12	0.38516898	0.663418079	0.573223624	-0.915130454	1.685468413	1.469862677	0.40046338	5.394977421
Myeloproliferative neoplasms	genus..Eubacteriumventriosumgroup.id.11341	Weighted mode	13	-0.337900823	0.584226516	0.573707001	-1.482984794	0.807183147	0.713266024	0.22695925	2.241584871
Myeloproliferative neoplasms	genus.Alistipes.id.968	Weighted mode	12	0.479638007	0.827440161	0.573821007	-1.142144709	2.101420723	1.615489501	0.319133838	8.177780022
Myeloproliferative neoplasms	genus.FamilyXIIIAD3011group.id.11293	Weighted mode	12	-0.407070536	0.710575824	0.578251939	-1.79979915	0.985658078	0.66559724	0.165332092	2.679574673
Myeloproliferative neoplasms	family.Desulfovibrionaceae.id.3169	Weighted mode	8	0.435622249	0.752009972	0.580557186	-1.038317297	1.909561795	1.54592471	0.354049942	6.750130211
Myeloproliferative neoplasms	phylum.Tenericutes.id.3919	Weighted mode	10	0.326240288	0.56937538	0.580689898	-0.789735457	1.442216033	1.385748307	0.453964872	4.23005939
Myeloproliferative neoplasms	genus.Veillonella.id.2198	Weighted mode	6	0.459335506	0.784344858	0.583559133	-1.077980416	1.996651428	1.583021727	0.34028206	7.364354691
Myeloproliferative neoplasms	genus..Ruminococcusgauvreauiigroup.id.11342	Weighted mode	9	-0.3023982	0.530102754	0.584033301	-1.341399597	0.736603197	0.739043719	0.261479446	2.088828112
Myeloproliferative neoplasms	genus..Eubacteriumxylanophilumgroup.id.14375	Weighted mode	7	-0.385951009	0.670440759	0.585766871	-1.700014896	0.928112878	0.679803829	0.182680803	2.529730761
Myeloproliferative neoplasms	order.Desulfovibrionales.id.3156	Weighted mode	10	0.409013909	0.734614034	0.591255717	-1.030829598	1.848857416	1.505332658	0.356710911	6.352557044
Myeloproliferative neoplasms	genus.Catenibacterium.id.2153	Weighted mode	4	0.275984949	0.462514775	0.59273433	-0.63054401	1.182513908	1.31782803	0.532302145	3.262565692
Myeloproliferative neoplasms	class.Mollicutes.id.3920	Weighted mode	10	0.326240288	0.593472324	0.595890735	-0.836965468	1.489446044	1.385748307	0.433022553	4.434638242
Myeloproliferative neoplasms	genus.Flavonifractor.id.2059	Weighted mode	4	0.40838545	0.698498325	0.599838669	-0.960671268	1.777442167	1.504386915	0.382635949	5.91470822
Myeloproliferative neoplasms	genus.Dialister.id.2183	Weighted mode	11	0.327285902	0.609271118	0.602892587	-0.86688549	1.521457294	1.387198023	0.420258412	4.578893129
Myeloproliferative neoplasms	order.Enterobacteriales.id.3468	Weighted mode	5	-0.420638596	0.807033194	0.629741785	-2.002423656	1.161146465	0.656627366	0.135007674	3.193592519
Myeloproliferative neoplasms	genus.Odoribacter.id.952	Weighted mode	5	0.529128352	1.07983593	0.649788831	-1.587350071	2.645606774	1.697452082	0.204466717	14.09199314
Myeloproliferative neoplasms	family.Enterobacteriaceae.id.3469	Weighted mode	5	-0.420638596	0.866856429	0.652880304	-2.119677196	1.278400005	0.656627366	0.120070381	3.59088972
Myeloproliferative neoplasms	genus.Lachnoclostridium.id.11308	Weighted mode	13	0.313935645	0.68489543	0.654876716	-1.028459399	1.656330688	1.368801644	0.35755739	5.240048154
Myeloproliferative neoplasms	family.Oxalobacteraceae.id.2966	Weighted mode	14	0.163794089	0.359525385	0.656205419	-0.540875666	0.868463844	1.177971733	0.582238183	2.383247
Myeloproliferative neoplasms	genus.Anaerofilum.id.2053	Weighted mode	9	-0.186990462	0.405699905	0.657135584	-0.982162276	0.608181351	0.829451647	0.374500449	1.837087343
Myeloproliferative neoplasms	family.Alcaligenaceae.id.2875	Weighted mode	11	-0.271043059	0.593140224	0.657463434	-1.433597898	0.891511779	0.762583659	0.23844946	2.438813814
Myeloproliferative neoplasms	genus.Oscillospira.id.2064	Weighted mode	7	0.340467836	0.734486666	0.659312564	-1.099126029	1.780061701	1.405605029	0.33316213	5.93022231
Myeloproliferative neoplasms	genus..Ruminococcusgnavusgroup.id.14376	Weighted mode	11	-0.211262734	0.465909214	0.659911262	-1.124444793	0.701919326	0.80956134	0.324832767	2.017621466
Myeloproliferative neoplasms	genus..Eubacteriumruminantiumgroup.id.11340	Weighted mode	16	0.166791346	0.37716287	0.664635093	-0.57244788	0.906030571	1.181507713	0.564142793	2.474480738
Myeloproliferative neoplasms	family.Ruminococcaceae.id.2050	Weighted mode	7	-0.249228661	0.552875103	0.667975888	-1.332863863	0.83440654	0.779401734	0.263720918	2.30344664
Myeloproliferative neoplasms	genus.Actinomyces.id.423	Weighted mode	5	-0.270621851	0.591858614	0.67122991	-1.430664735	0.889421032	0.762904933	0.239149898	2.433720196
Myeloproliferative neoplasms	genus.LachnospiraceaeUCG004.id.11324	Weighted mode	12	0.382257947	0.88699263	0.674821376	-1.356247607	2.120763501	1.465590081	0.257625678	8.337500752
Myeloproliferative neoplasms	genus.Eisenbergiella.id.11304	Weighted mode	11	-0.190930638	0.441913166	0.674869403	-1.057080427	0.675219167	0.826189893	0.347468786	1.964463474
Myeloproliferative neoplasms	class.Betaproteobacteria.id.2867	Weighted mode	10	0.279545811	0.646624594	0.67568558	-0.987838394	1.546930016	1.322528998	0.372380762	4.697028223
Myeloproliferative neoplasms	order.Pasteurellales.id.3688	Weighted mode	10	-0.192859654	0.462499839	0.686398349	-1.099395288	0.71360408	0.824568051	0.333072436	2.041335151
Myeloproliferative neoplasms	genus..Eubacteriumbrachygroup.id.11296	Weighted mode	6	0.166845798	0.392581862	0.688508758	-0.602614652	0.936306249	1.18157201	0.547378559	2.550542926
Myeloproliferative neoplasms	family.Pasteurellaceae.id.3689	Weighted mode	10	-0.192859654	0.470678352	0.691517575	-1.115425174	0.729633865	0.824568051	0.327775888	2.074321195
Myeloproliferative neoplasms	class.Gammaproteobacteria.id.3303	Weighted mode	6	0.402583517	0.982905663	0.699066993	-1.523911582	2.329078617	1.495683836	0.217858048	10.26847597
Myeloproliferative neoplasms	class.Alphaproteobacteria.id.2379	Weighted mode	6	0.207063863	0.50598868	0.699313762	-0.784673951	1.198801676	1.230061124	0.456268443	3.31614073
Myeloproliferative neoplasms	family.Clostridiaceae1.id.1869	Weighted mode	7	-0.281006643	0.705946	0.704365356	-1.664660803	1.102647516	0.75502332	0.189254842	3.012130141

Myeloproliferative neoplasms	genus.Coprococcus1.id.11301	Weighted mode	9	-0.339387922	0.883745036	0.710959361	-2.071528193	1.392752349	0.712206115	0.125993093	4.025915542
Myeloproliferative neoplasms	genus.Bilophila.id.3170	Weighted mode	13	-0.256135362	0.675118338	0.711023936	-1.579367634	1.06709625	0.774036931	0.206105391	2.906926245
Myeloproliferative neoplasms	genus.Holdemanella.id.11393	Weighted mode	9	-0.148921994	0.404557398	0.722340118	-0.941854494	0.644010506	0.861636325	0.38990409	1.904101999
Myeloproliferative neoplasms	family.Peptococcaceae.id.2024	Weighted mode	8	-0.179468641	0.491572839	0.725825137	-1.142951406	0.784014123	0.835714157	0.318876498	2.190246563
Myeloproliferative neoplasms	genus.ErysipelotrichaceaeUCG003.id.11384	Weighted mode	12	0.200663324	0.566496737	0.729872265	-0.909670281	1.310996929	1.222213213	0.402656966	3.709870346
Myeloproliferative neoplasms	genus.Parabacteroides.id.954	Weighted mode	5	0.288033091	0.787557586	0.733087372	-1.255579777	1.831645959	1.333801441	0.284910616	6.24415583
Myeloproliferative neoplasms	genus.Roseburia.id.2012	Weighted mode	11	-0.215566148	0.627243993	0.738209401	-1.444964373	1.013832078	0.806084948	0.235754476	2.756142557
Myeloproliferative neoplasms	genus.Marvinbryantia.id.2005	Weighted mode	9	0.216741793	0.627263827	0.738602173	-1.012695307	1.446178893	1.242023361	0.363238619	4.24685578
Myeloproliferative neoplasms	order.Erysipelotrichales.id.2148	Weighted mode	11	-0.274126433	0.81004426	0.742052563	-1.861813183	1.313560317	0.76023595	0.155390623	3.719392385
Myeloproliferative neoplasms	class.Erysipelotrichia.id.2147	Weighted mode	11	-0.274126433	0.812180367	0.74270327	-1.865999953	1.317747087	0.76023595	0.154741398	3.734997268
Myeloproliferative neoplasms	genus..Eubacteriumcoprostanoligenesgroup.id.11375	Weighted mode	12	-0.240499619	0.718163491	0.744013401	-1.648100062	1.167100824	0.786234945	0.192415138	3.212665042
Myeloproliferative neoplasms	family.Erysipelotrichaceae.id.2149	Weighted mode	11	-0.274126433	0.818706081	0.744671409	-1.878790352	1.330537487	0.76023595	0.152774798	3.783076194
Myeloproliferative neoplasms	genus.ChristensenellaceaeR.7group.id.11283	Weighted mode	6	-0.293535905	0.878917217	0.751950939	-2.016213651	1.429141841	0.745622451	0.133158697	4.175114743
Myeloproliferative neoplasms	genus..Eubacteriumnodatumgroup.id.11297	Weighted mode	11	-0.108100742	0.347406307	0.762064752	-0.789017103	0.572815619	0.897537172	0.454291097	1.773252833
Myeloproliferative neoplasms	genus.Coprococcus2.id.11302	Weighted mode	6	-0.258910483	0.833409207	0.768589115	-1.892392528	1.374571562	0.771892117	0.150710797	3.953382582
Myeloproliferative neoplasms	genus.Olsenella.id.822	Weighted mode	9	-0.086781419	0.288879742	0.77152538	-0.652985714	0.479422875	0.916877485	0.520489422	1.615141995
Myeloproliferative neoplasms	genus.FamilyXIIIUCG001.id.11294	Weighted mode	7	-0.199440894	0.705189004	0.786819227	-1.581611342	1.182729555	0.819188639	0.205643469	3.26326933
Myeloproliferative neoplasms	genus.Sellimonas.id.14369	Weighted mode	7	0.094445895	0.334106646	0.786919483	-0.560403132	0.749294922	1.099049697	0.570978838	2.115507892
Myeloproliferative neoplasms	genus.Butyrivibrio.id.1993	Weighted mode	13	0.088399628	0.321921939	0.788294206	-0.542567372	0.719366628	1.092424598	0.58125404	2.053132401
Myeloproliferative neoplasms	phylum.Actinobacteria.id.400	Weighted mode	14	0.150222642	0.550209062	0.789120601	-0.92818712	1.228632403	1.162092945	0.395269638	3.416553873
Myeloproliferative neoplasms	genus.Howardella.id.2000	Weighted mode	9	0.105622077	0.389492797	0.793119639	-0.657783806	0.869027959	1.111401772	0.517998047	2.384591807
Myeloproliferative neoplasms	order.Actinomycetales.id.420	Weighted mode	3	-0.205159846	0.717303508	0.801769989	-1.61107472	1.200755029	0.814517109	0.199672906	3.322624655
Myeloproliferative neoplasms	family.Actinomycetaceae.id.421	Weighted mode	3	-0.203050222	0.73541841	0.808384331	-1.644470306	1.238369861	0.816237247	0.193114828	3.449984924
Myeloproliferative neoplasms	genus.Intestinibacter.id.11345	Weighted mode	14	-0.108445882	0.453282898	0.814645764	-0.996880362	0.779988599	0.89722745	0.369028884	2.181447394
Myeloproliferative neoplasms	genus.Barnesiella.id.944	Weighted mode	10	0.155146799	0.651932375	0.817226265	-1.122640656	1.432934254	1.167829384	0.325419338	4.190978562
Myeloproliferative neoplasms	family.FamilyXIII.id.1957	Weighted mode	5	0.235320254	1.022368243	0.829250441	-1.768521504	2.239162011	1.265313925	0.170585012	9.385463072
Myeloproliferative neoplasms	genus.Ruminococcus1.id.11373	Weighted mode	8	-0.142471286	0.648606917	0.832406058	-1.413740844	1.128798272	0.867212455	0.243231687	3.091938598
Myeloproliferative neoplasms	order.Bacillales.id.1674	Weighted mode	8	-0.082193013	0.38801075	0.838273962	-0.842694083	0.678308057	0.921094159	0.430549025	1.970540868
Myeloproliferative neoplasms	genus.Fusicatenibacter.id.11305	Weighted mode	18	0.122000842	0.589274209	0.838441529	-1.032976607	1.276978291	1.129755053	0.355945871	3.58578813
Myeloproliferative neoplasms	order.Burkholderiales.id.2874	Weighted mode	10	0.127956223	0.657102218	0.849930647	-1.159964125	1.41587657	1.136503248	0.313497427	4.120096437
Myeloproliferative neoplasms	family.Victivallaceae.id.2255	Weighted mode	9	-0.066816996	0.345393205	0.851426421	-0.743787679	0.610153686	0.935366361	0.475310179	1.84071427
Myeloproliferative neoplasms	genus.LachnospiraceaeND3007group.id.11317	Weighted mode	3	0.179751334	0.847962459	0.851763322	-1.482255085	1.841757753	1.196919693	0.227124925	6.307615754
Myeloproliferative neoplasms	class.Bacteroidia.id.912	Weighted mode	12	-0.098184932	0.532613904	0.85709704	-1.142108183	0.94573832	0.906481251	0.319145495	2.57471364
Myeloproliferative neoplasms	phylum.Lentisphaerae.id.2238	Weighted mode	9	0.071689746	0.39295813	0.859778792	-0.698508189	0.841887681	1.07432198	0.497326668	2.320743669
Myeloproliferative neoplasms	order.Bacteroidales.id.913	Weighted mode	12	-0.098184932	0.557728825	0.863458291	-1.19133343	0.994963566	0.906481251	0.303815877	2.7046258
Myeloproliferative neoplasms	genus.Clostridiumsensustricto1.id.1873	Weighted mode	5	0.111996969	0.648874928	0.871345874	-1.159797889	1.383791828	1.118509471	0.313549546	3.990002381
Myeloproliferative neoplasms	order.Gastranaerophilales.id.1591	Weighted mode	9	0.071337208	0.447023924	0.877165437	-0.804829684	0.9475041	1.073943308	0.447164079	2.579264035
Myeloproliferative neoplasms	genus.Bacteroides.id.918	Weighted mode	7	-0.116066746	0.720036754	0.877230129	-1.527338783	1.295205291	0.89041579	0.217112683	3.651745569
Myeloproliferative neoplasms	genus.RikenellaceaeRC9gutgroup.id.11191	Weighted mode	10	0.051662271	0.335725831	0.881097793	-0.606360359	0.7096849	1.053020047	0.545332075	2.033350449
Myeloproliferative neoplasms	family.Bacteroidaceae.id.917	Weighted mode	7	-0.116066746	0.745993212	0.881461161	-1.57821344	1.346079949	0.89041579	0.206343414	3.842333825
Myeloproliferative neoplasms	family.Lactobacillaceae.id.1836	Weighted mode	7	0.072947599	0.507378446	0.890386597	-0.921514154	1.067409353	1.07567417	0.397916078	2.907836556
Myeloproliferative neoplasms	genus.Turicibacter.id.2162	Weighted mode	8	-0.075497528	0.530678426	0.890878048	-1.115627242	0.964632187	0.927282023	0.327709661	2.623822403
Myeloproliferative neoplasms	class.Bacilli.id.1673	Weighted mode	14	0.071786603	0.52123708	0.892569243	-0.949838073	1.093411279	1.07442604	0.386803652	2.984437477
Myeloproliferative neoplasms	genus.Prevotella9.id.11183	Weighted mode	13	-0.074086941	0.554492321	0.895923824	-1.160891889	1.012718008	0.928590958	0.313206711	2.753073731
Myeloproliferative neoplasms	family.Porphyromonadaceae.id.943	Weighted mode	9	0.095569614	0.805771471	0.908511331	-1.483742468	1.674881697	1.100285415	0.226787354	5.338163589
Myeloproliferative neoplasms	order.Lactobacillales.id.1800	Weighted mode	11	0.066491844	0.618727852	0.916545287	-1.146214746	1.279198434	1.068752247	0.317837591	3.593757938
Myeloproliferative neoplasms	genus.Allisonella.id.2174	Weighted mode	8	-0.039001606	0.373013282	0.91965921	-0.770107638	0.692104425	0.961749164	0.462963233	1.997915577
Myeloproliferative neoplasms	genus.Hungatella.id.11306	Weighted mode	4	0.064879083	0.637529337	0.925362605	-1.184678418	1.314436584	1.067029995	0.305844518	3.722652993
Myeloproliferative neoplasms	order.Selenomonadales.id.2165	Weighted mode	12	-0.053578758	0.691854339	0.93966242	-1.409613262	1.302455746	0.947831289	0.244237721	3.678318603
Myeloproliferative neoplasms	genus.RuminococcaceaeUCG011.id.11368	Weighted mode	8	-0.026760653	0.356476117	0.942259375	-0.725453842	0.671932536	0.973594241	0.484104812	1.958017607
Myeloproliferative neoplasms	class.Negativicutes.id.2164	Weighted mode	12	-0.053578758	0.729450424	0.942765976	-1.483301589	1.376144074	0.947831289	0.226887362	3.959604212
Myeloproliferative neoplasms	genus..Eubacteriumrectalegroup.id.14374	Weighted mode	8	0.047456423	0.657213215	0.944455798	-1.24068148	1.335594325	1.048600505	0.289187076	3.802255049
Myeloproliferative neoplasms	phylum.Cyanobacteria.id.1500	Weighted mode	7	-0.039843276	0.555857997	0.945187041	-1.129324951	1.049638398	0.960940029	0.323251393	2.856617974
Myeloproliferative neoplasms	genus.Lachnospira.id.2004	Weighted mode	5	-0.068596958	0.945376257	0.945639248	-1.921534421	1.784340505	0.933702926	0.146382178	5.955650931
Myeloproliferative neoplasms	genus.Haemophilus.id.3698	Weighted mode	8	-0.032106392	0.490275258	0.949617695	-0.993045898	0.928833114	0.968403546	0.370446628	2.531553419
Myeloproliferative neoplasms	genus.Lactobacillus.id.1837	Weighted mode	7	0.027444751	0.47280774	0.955596628	-0.89925842	0.954147921	1.027824827	0.406871275	2.596457255
Myeloproliferative neoplasms	genus.Adlercreutzia.id.812	Weighted mode	7	0.027143319	0.611012075	0.966008354	-1.170440348	1.224726986	1.027515055	0.310230302	3.403236825
Myeloproliferative neoplasms	order.Rhodospirillales.id.2667	Weighted mode	12	0.021691413	0.518420455	0.967374987	-0.994412678	1.037795505	1.021928382	0.369940655	2.822986888
Myeloproliferative neoplasms	genus.Escherichia.Shigella.id.3504	Weighted mode	8	-0.026827237	0.648073898	0.968136699	-1.297052077	1.243397604	0.973529417	0.273336381	3.467374238
Myeloproliferative neoplasms	genus..Eubacteriumeligensgroup.id.14372	Weighted mode	6	0.030111372	0.725155925	0.968485293	-1.39119424	1.451416985	1.030569305	0.248778026	4.269159566
Myeloproliferative neoplasms	family.FamilyXI.id.1936	Weighted mode	8	0.011137836	0.288442656	0.970276558	-0.554209769	0.576485441	1.011200093	0.57452609	1.779772312
Myeloproliferative neoplasms	genus.LachnospiraceaeUCG010.id.11330	Weighted mode	10	0.021046236	0.555408688	0.970600016	-1.067554792	1.109647264	1.02126927	0.343848271	3.033288255
Myeloproliferative neoplasms	family.Rhodospirillaceae.id.2717	Weighted mode	13	-0.016231851	0.469676763	0.972999116	-0.936798306	0.904334603	0.983899175	0.39188051	2.470287655
Myeloproliferative neoplasms	genus..Clostridiuminnocuumgroup.id.14397	Weighted mode	6	-0.008272665	0.352059668	0.982162045	-0.698309668	0.681764285	0.991761459	0.497425434	1.977363289
Myeloproliferative neoplasms	genus.Methanobrevibacter.id.123	Weighted mode	6	0.00664037	0.417602377	0.987928223	-0.811860289	0.825141029	1.006662466	0.444031271	2.282202599
Myeloproliferative neoplasms	genus.Desulfovibrio.id.3173	Weighted mode	8	0.007674133	0.532912401	0.988912404	-1.036834173	1.052182439	1.007703654	0.354575431	2.863894577
Myeloproliferative neoplasms	order.Victivallales.id.2254	Weighted mode	8	0.004594613	0.398156988	0.991114851	-0.775793083	0.78498231	1.004605185	0.460338549	2.192368158
Myeloproliferative neoplasms	class.Lentisphaeria.id.2250	Weighted mode	8	0.004594613	0.413917308	0.991453146	-0.80668331	0.815872537	1.004605185	0.446335972	2.261147746
Myeloproliferative neoplasms	genus.Prevotella7.id.11182	Weighted mode	9	0.00348708	0.374152116	0.992792084	-0.729851067	0.736825227	1.003493167	0.481980768	2.089291946
Myeloproliferative neoplasms	genus.Paraprevotella.id.962	Weighted mode	13	-0.000740692	0.401248075	0.998557463	-0.787186919	0.785705535	0.999259582	0.455123295	2.193954306

Supplementary Table 5. Five Mendelian randomization models estimate the causal effects of myeloproliferative neoplasms on gut microbiota

Outcome	Exposure	Method	Number of	Beta	Standard error	P-value	lo_ci	up_ci	OR	OR_lci95	OR_uci95
family.Lachnospiraceae.id.1987	Myeloproliferative neoplasms	Inverse variance weighted	13	-0.011696906	0.010550076	0.267558124	-0.032375056	0.008981243	0.988371237	0.968143406	1.009021696
family.Desulfovibrionaceae.id.3169	Myeloproliferative neoplasms	Inverse variance weighted	13	-0.020874749	0.01031053	0.052907945	-0.041083388	-0.000666111	0.97934162	0.959749095	0.999334111
genus.Streptococcus.id.1853	Myeloproliferative neoplasms	Inverse variance weighted	13	0.004607614	0.009917538	0.642223261	-0.01483076	0.024045989	1.004618246	0.985278674	1.024337425
genus.Romboutsia.id.11347	Myeloproliferative neoplasms	Inverse variance weighted	13	-0.008355369	0.010661976	0.433239388	-0.029252842	0.012542105	0.99167944	0.97117088	1.012621087
genus.Collinsella.id.815	Myeloproliferative neoplasms	Inverse variance weighted	13	-0.018680311	0.010568783	0.077144719	-0.039395125	0.002034503	0.981493084	0.961370772	1.002036574
genus..Eubacteriumhalliigroup.id.11	Myeloproliferative neoplasms	Inverse variance weighted	13	-0.010131684	0.010208462	0.320963947	-0.03014027	0.009876902	0.989919469	0.970309419	1.00992584
genus..Eubacteriumxylanophilumgr	Myeloproliferative neoplasms	Inverse variance weighted	13	0.015181865	0.010997917	0.167454002	-0.006374053	0.036737783	1.015297695	0.993646219	1.037420955
family.Lachnospiraceae.id.1987	Myeloproliferative neoplasms	MR Egger	13	-0.060201364	0.02856899	0.058858921	-0.116196584	-0.004206143	0.941574915	0.890300187	0.99580269
family.Desulfovibrionaceae.id.3169	Myeloproliferative neoplasms	MR Egger	13	-0.000553346	0.030429349	0.985817231	-0.06019487	0.059088179	0.999446807	0.941581029	1.060868783
genus.Streptococcus.id.1853	Myeloproliferative neoplasms	MR Egger	13	0.02564717	0.02927166	0.399662962	-0.031725283	0.083019623	1.025978888	0.968772684	1.08656313
genus.Romboutsia.id.11347	Myeloproliferative neoplasms	MR Egger	13	-0.009953131	0.031483239	0.757813616	-0.071660279	0.051754018	0.990096238	0.930847071	1.053116663
genus.Collinsella.id.815	Myeloproliferative neoplasms	MR Egger	13	-0.034117546	0.031203683	0.297592065	-0.095276765	0.027041672	0.966457894	0.909121287	1.027410617
genus..Eubacteriumhalliigroup.id.11	Myeloproliferative neoplasms	MR Egger	13	-0.015758585	0.031438174	0.62607018	-0.077377406	0.045860237	0.984364932	0.925540483	1.046928079
genus..Eubacteriumxylanophilumgr	Myeloproliferative neoplasms	MR Egger	13	0.04156832	0.032452605	0.226577875	-0.022038786	0.105175427	1.04244438	0.978202294	1.110905476
family.Lachnospiraceae.id.1987	Myeloproliferative neoplasms	Simple mode	13	0.017758859	0.027647961	0.532744668	-0.036431145	0.071948862	1.017917485	0.964224483	1.07460039
family.Desulfovibrionaceae.id.3169	Myeloproliferative neoplasms	Simple mode	13	-0.014354227	0.023663653	0.555423021	-0.060734987	0.032026533	0.985748304	0.941072603	1.032544902
genus.Streptococcus.id.1853	Myeloproliferative neoplasms	Simple mode	13	-0.015630377	0.022957834	0.508903709	-0.060627731	0.029366978	0.984491144	0.941173544	1.02980244
genus.Romboutsia.id.11347	Myeloproliferative neoplasms	Simple mode	13	-0.011665006	0.023440393	0.627727647	-0.057608177	0.034278166	0.988402767	0.944019764	1.034872433
genus.Collinsella.id.815	Myeloproliferative neoplasms	Simple mode	13	-0.000817142	0.022845717	0.972055568	-0.045594748	0.043960464	0.999183191	0.955429073	1.044941041
genus..Eubacteriumhalliigroup.id.11	Myeloproliferative neoplasms	Simple mode	13	-0.005639679	0.02316338	0.811750694	-0.051039903	0.039760546	0.994376194	0.950240752	1.040561577
genus..Eubacteriumxylanophilumgr	Myeloproliferative neoplasms	Simple mode	13	0.007430295	0.023089741	0.753140196	-0.037825599	0.052686188	1.007457968	0.962880854	1.054098804
family.Lachnospiraceae.id.1987	Myeloproliferative neoplasms	Weighted median	13	-0.028697218	0.013883849	0.038738887	-0.055909562	-0.001484875	0.971710636	0.945624652	0.998516227
family.Desulfovibrionaceae.id.3169	Myeloproliferative neoplasms	Weighted median	13	-0.010965136	0.014466444	0.448468708	-0.039319367	0.017389094	0.989094762	0.961443607	1.017541165
genus.Streptococcus.id.1853	Myeloproliferative neoplasms	Weighted median	13	0.001042621	0.014319111	0.941954722	-0.027022837	0.029108079	1.001043165	0.973339013	1.02953586
genus.Romboutsia.id.11347	Myeloproliferative neoplasms	Weighted median	13	-0.01273431	0.014728871	0.387268491	-0.041602897	0.016134276	0.987346428	0.959250627	1.016265137
genus.Collinsella.id.815	Myeloproliferative neoplasms	Weighted median	13	-0.026958298	0.014365884	0.060579816	-0.055115431	0.001198835	0.973401833	0.9463759	1.001199554
genus..Eubacteriumhalliigroup.id.11	Myeloproliferative neoplasms	Weighted median	13	-0.018379509	0.013651761	0.178202399	-0.045136961	0.008377942	0.981788364	0.955866557	1.008413135
genus..Eubacteriumxylanophilumgr	Myeloproliferative neoplasms	Weighted median	13	0.013569043	0.015656663	0.386126952	-0.017118016	0.044256102	1.01366152	0.983027664	1.045250011
family.Lachnospiraceae.id.1987	Myeloproliferative neoplasms	Weighted mode	13	-0.030431762	0.016373191	0.087768556	-0.062523217	0.001659692	0.970026622	0.939391253	1.00166107
family.Desulfovibrionaceae.id.3169	Myeloproliferative neoplasms	Weighted mode	13	-0.009393366	0.016057076	0.569385535	-0.040865235	0.022078503	0.990650614	0.95995849	1.022324037
genus.Streptococcus.id.1853	Myeloproliferative neoplasms	Weighted mode	13	-0.002670888	0.015847183	0.86896571	-0.033731367	0.028389591	0.997332676	0.966831192	1.028796416
genus.Romboutsia.id.11347	Myeloproliferative neoplasms	Weighted mode	13	-0.01541657	0.018860291	0.429625823	-0.052382741	0.0215496	0.984701657	0.94896559	1.021783469
genus.Collinsella.id.815	Myeloproliferative neoplasms	Weighted mode	13	-0.028171741	0.01812408	0.146059218	-0.063694937	0.007351455	0.972221382	0.938291193	1.007378543
genus..Eubacteriumhalliigroup.id.11	Myeloproliferative neoplasms	Weighted mode	13	-0.015812155	0.016992077	0.370423859	-0.049116626	0.017492316	0.984312201	0.952070087	1.017646202
genus..Eubacteriumxylanophilumgr	Myeloproliferative neoplasms	Weighted mode	13	0.011363384	0.017439676	0.526952993	-0.022818381	0.04554515	1.011428193	0.977439989	1.046598258

Supplementary Table 6. Pleiotropy of all Mendelian randomization results

Outcome	Exposure	Egger_intercept	Standard e	P-value
Myeloproliferative neoplasms	genus.Streptococcus.id.1853	0.025469136	0.094895	0.795187314
Myeloproliferative neoplasms	genus.Romboutsia.id.11347	0.008004978	0.081347	0.92376701
Myeloproliferative neoplasms	family.Lachnospiraceae.id.1987	-0.033837939	0.074502	0.657184825
Myeloproliferative neoplasms	family.Desulfovibrionaceae.id.3169	-0.003741765	0.138435	0.979313115
Myeloproliferative neoplasms	genus.Collinsella.id.815	0.102640186	0.095842	0.319731058
Myeloproliferative neoplasms	genus..Eubacteriumhalliigroup.id.11338	0.086870556	0.049232	0.105358922
Myeloproliferative neoplasms	genus..Eubacteriumxylanophilumgroup.id.14375	-0.155045721	0.113356	0.22965097
Lysine	family.Desulfovibrionaceae.id.3169	0.005528168	0.004493	0.285978141
X_11423	family.Desulfovibrionaceae.id.3169	0.001690044	0.010596	0.881001585
Oleoylcarnitine	family.Desulfovibrionaceae.id.3169	-0.007686874	0.010077	0.488097562
Acetylcarnitine	family.Desulfovibrionaceae.id.3169	-0.005509693	0.006886	0.468470182
X_12798	family.Desulfovibrionaceae.id.3169	0.014678888	0.009637	0.202382546
X_12850	family.Desulfovibrionaceae.id.3169	0.002419938	0.015393	0.882692306
Epiandrosterone sulfate	family.Desulfovibrionaceae.id.3169	-0.009400286	0.016826	0.606187913
Stearoylcarnitine	family.Desulfovibrionaceae.id.3169	-0.001196692	0.009372	0.904558801
Betaine	family.Desulfovibrionaceae.id.3169	0.006714435	0.00921	0.506376162
X_12092	family.Desulfovibrionaceae.id.3169	-0.008686544	0.015505	0.605205618
3-dehydrocarnitine	family.Desulfovibrionaceae.id.3169	-1.28E-05	0.006723	0.998574037
Succinylcarnitine	family.Desulfovibrionaceae.id.3169	3.60E-05	0.006113	0.995577611

X_11440	family.Desulfovibrionaceae.id.3169	-0.00173063	0.0143	0.909510041
4-androsten-3beta,17beta-diol disulf	family.Desulfovibrionaceae.id.3169	-0.008301854	0.018406	0.675325965
Propionylcarnitine	family.Desulfovibrionaceae.id.3169	-0.000572333	0.006952	0.93834532
X_12442	family.Desulfovibrionaceae.id.3169	-0.002550703	0.016092	0.881734027
Palmitoylcarnitine	family.Desulfovibrionaceae.id.3169	0.004710015	0.012795	0.731444436
Lysine	genus..Eubacteriumhalliigroup.id.11338	0.003300698	0.002185	0.19129189
X_11423	genus..Eubacteriumhalliigroup.id.11338	0.003140258	0.002937	0.333826388
Oleoylcarnitine	genus..Eubacteriumhalliigroup.id.11338	0.004840212	0.007132	0.527498904
Acetylcarnitine	genus..Eubacteriumhalliigroup.id.11338	0.000849375	0.003332	0.808937685
X_12798	genus..Eubacteriumhalliigroup.id.11338	0.000100044	0.004608	0.983516558
X_12850	genus..Eubacteriumhalliigroup.id.11338	0.009021903	0.008148	0.318568568
Epiandrosterone sulfate	genus..Eubacteriumhalliigroup.id.11338	-0.00220685	0.007996	0.793595033
Stearoylcarnitine	genus..Eubacteriumhalliigroup.id.11338	-0.0019816	0.004431	0.673453315
Betaine	genus..Eubacteriumhalliigroup.id.11338	0.003609782	0.003082	0.294336174
X_12092	genus..Eubacteriumhalliigroup.id.11338	0.00805125	0.007656	0.341100509
3-dehydrocarnitine	genus..Eubacteriumhalliigroup.id.11338	0.000170208	0.004064	0.968213003
Succinylcarnitine	genus..Eubacteriumhalliigroup.id.11338	-0.000236215	0.002844	0.937030655
X_11440	genus..Eubacteriumhalliigroup.id.11338	-0.005135153	0.006761	0.481776364
4-androsten-3beta,17beta-diol disulf	genus..Eubacteriumhalliigroup.id.11338	0.003582802	0.008753	0.699249677
Propionylcarnitine	genus..Eubacteriumhalliigroup.id.11338	0.001489467	0.00289	0.628233319
X_12442	genus..Eubacteriumhalliigroup.id.11338	0.001337616	0.005598	0.820644226
Palmitoylcarnitine	genus..Eubacteriumhalliigroup.id.11338	-0.001119588	0.007482	0.886904811
Lysine	genus.Collinsella.id.815	-0.004653	0.008469	0.680180017
X_11423	genus.Collinsella.id.815	0.004052584	0.00517	0.576739451
Oleoylcarnitine	genus.Collinsella.id.815	0.009330691	0.011186	0.557401428
Acetylcarnitine	genus.Collinsella.id.815	-0.001261663	0.007298	0.891012471
X_12798	genus.Collinsella.id.815	-0.002360553	0.010423	0.858208403
X_12850	genus.Collinsella.id.815	0.001331518	0.045465	0.981361031
Epiandrosterone sulfate	genus.Collinsella.id.815	-0.009694589	0.024001	0.755610874
Stearoylcarnitine	genus.Collinsella.id.815	0.006456299	0.027568	0.85354566
Betaine	genus.Collinsella.id.815	-0.009103279	0.006029	0.3724019
X_12092	genus.Collinsella.id.815	-0.00981065	0.015254	0.636140659
3-dehydrocarnitine	genus.Collinsella.id.815	0.003384528	0.007297	0.723535291
Succinylcarnitine	genus.Collinsella.id.815	-0.008044531	0.006217	0.418867421
X_11440	genus.Collinsella.id.815	0.003241281	0.015506	0.868817058
4-androsten-3beta,17beta-diol disulf	genus.Collinsella.id.815	0.011021405	0.02008	0.680429671
Propionylcarnitine	genus.Collinsella.id.815	-0.000447757	0.006125	0.953542169
X_12442	genus.Collinsella.id.815	-0.0063204	0.012794	0.70789462
Palmitoylcarnitine	genus.Collinsella.id.815	0.012254331	0.010168	0.440933752
Lysine	genus.Streptococcus.id.1853	0.0078142	0.005292	0.236287142
X_11423	genus.Streptococcus.id.1853	0.002993046	0.00629	0.666685524
Oleoylcarnitine	genus.Streptococcus.id.1853	-0.008202446	0.012267	0.551552298
Acetylcarnitine	genus.Streptococcus.id.1853	-0.000905568	0.007913	0.916113001
X_12798	genus.Streptococcus.id.1853	-0.003991823	0.01219	0.764835774
X_12850	genus.Streptococcus.id.1853	-0.012071553	0.033575	0.743012616
Epiandrosterone sulfate	genus.Streptococcus.id.1853	-0.009309989	0.019853	0.671078385
Stearoylcarnitine	genus.Streptococcus.id.1853	-0.014018087	0.011196	0.299287523
Betaine	genus.Streptococcus.id.1853	-0.004865433	0.008618	0.611822472
X_12092	genus.Streptococcus.id.1853	0.047719219	0.024473	0.1462906
3-dehydrocarnitine	genus.Streptococcus.id.1853	0.013954707	0.008287	0.190788902
Succinylcarnitine	genus.Streptococcus.id.1853	0.00220859	0.007967	0.79961177
X_11440	genus.Streptococcus.id.1853	0.00132182	0.017431	0.944327509
4-androsten-3beta,17beta-diol disulf	genus.Streptococcus.id.1853	0.009698665	0.02207	0.690064244
Propionylcarnitine	genus.Streptococcus.id.1853	0.002205147	0.007913	0.798588434
X_12442	genus.Streptococcus.id.1853	-0.007396449	0.014058	0.635202368
Palmitoylcarnitine	genus.Streptococcus.id.1853	-0.00866434	0.010701	0.477406133
Lysine	family.Lachnospiraceae.id.1987	0.0014627	0.006676	0.837294212
X_11423	family.Lachnospiraceae.id.1987	0.015411825	0.007961	0.124950874
Oleoylcarnitine	family.Lachnospiraceae.id.1987	-0.018068952	0.026598	0.534199889
Acetylcarnitine	family.Lachnospiraceae.id.1987	-0.013574554	0.012024	0.322037439
X_12798	family.Lachnospiraceae.id.1987	-0.004136953	0.014524	0.789903234
X_12850	family.Lachnospiraceae.id.1987	-0.029559107	0.034757	0.442984231
Epiandrosterone sulfate	family.Lachnospiraceae.id.1987	0.028191089	0.034166	0.455665207
Stearoylcarnitine	family.Lachnospiraceae.id.1987	-0.022818499	0.014257	0.184731427
Betaine	family.Lachnospiraceae.id.1987	-0.004690911	0.01324	0.74100346
X_12092	family.Lachnospiraceae.id.1987	-0.001764216	0.024498	0.946046524
3-dehydrocarnitine	family.Lachnospiraceae.id.1987	-0.002512504	0.010261	0.818619948
Succinylcarnitine	family.Lachnospiraceae.id.1987	-0.00545525	0.010701	0.637030179
X_11440	family.Lachnospiraceae.id.1987	-0.024097112	0.021546	0.326026937
4-androsten-3beta,17beta-diol disulf	family.Lachnospiraceae.id.1987	-0.052608212	0.02857	0.139370682
Propionylcarnitine	family.Lachnospiraceae.id.1987	0.022704659	0.016657	0.244555908
X_12442	family.Lachnospiraceae.id.1987	-0.021617174	0.029115	0.499036903
Palmitoylcarnitine	family.Lachnospiraceae.id.1987	-0.020465798	0.016225	0.275732793
Lysine	genus..Eubacteriumxylanophilumgroup.id.14375	0.000188889	0.003171	0.957918785
X_11423	genus..Eubacteriumxylanophilumgroup.id.14375	-0.001235393	0.003851	0.778773095
Oleoylcarnitine	genus..Eubacteriumxylanophilumgroup.id.14375	-0.00329934	0.008628	0.738972199
Acetylcarnitine	genus..Eubacteriumxylanophilumgroup.id.14375	-0.004369417	0.006608	0.576451533
X_12798	genus..Eubacteriumxylanophilumgroup.id.14375	0.01074549	0.006775	0.253622733
X_12850	genus..Eubacteriumxylanophilumgroup.id.14375	-0.015209748	0.011021	0.301570233
Epiandrosterone sulfate	genus..Eubacteriumxylanophilumgroup.id.14375	-0.027077531	0.011904	0.150755022
Stearoylcarnitine	genus..Eubacteriumxylanophilumgroup.id.14375	0.003773779	0.006553	0.622877671
Betaine	genus..Eubacteriumxylanophilumgroup.id.14375	0.003586843	0.004718	0.526469351
X_12092	genus..Eubacteriumxylanophilumgroup.id.14375	0.013689253	0.01127	0.348428721
3-dehydrocarnitine	genus..Eubacteriumxylanophilumgroup.id.14375	0.006324258	0.006703	0.445020874
Succinylcarnitine	genus..Eubacteriumxylanophilumgroup.id.14375	-0.006048357	0.004263	0.291760691
X_11440	genus..Eubacteriumxylanophilumgroup.id.14375	-0.023362681	0.010214	0.149453253
4-androsten-3beta,17beta-diol disulf	genus..Eubacteriumxylanophilumgroup.id.14375	-0.017452389	0.013046	0.312797851
Propionylcarnitine	genus..Eubacteriumxylanophilumgroup.id.14375	-0.004232659	0.004628	0.456929629
X_12442	genus..Eubacteriumxylanophilumgroup.id.14375	-0.009930457	0.011285	0.471690144
Palmitoylcarnitine	genus..Eubacteriumxylanophilumgroup.id.14375	-0.006314551	0.006477	0.432441821
Lysine	genus.Romboutsia.id.11347	0.004630004	0.01435	0.777570559
X_11423	genus.Romboutsia.id.11347	0.009405015	0.009578	0.429682447
Oleoylcarnitine	genus.Romboutsia.id.11347	0.01751672	0.020982	0.491641072
Acetylcarnitine	genus.Romboutsia.id.11347	0.003268835	0.01244	0.817319692
X_12798	genus.Romboutsia.id.11347	-0.004637659	0.01749	0.815710146
X_12850	genus.Romboutsia.id.11347	-0.004457729	0.029083	0.89225025
Epiandrosterone sulfate	genus.Romboutsia.id.11347	-0.042452686	0.030443	0.297869369
Stearoylcarnitine	genus.Romboutsia.id.11347	0.007252301	0.017474	0.71840447
Betaine	genus.Romboutsia.id.11347	-0.004708149	0.011716	0.726673816
X_12092	genus.Romboutsia.id.11347	0.000127692	0.02638	0.996577313
3-dehydrocarnitine	genus.Romboutsia.id.11347	-0.001211293	0.016818	0.949138548
Succinylcarnitine	genus.Romboutsia.id.11347	0.015669377	0.013466	0.364629691
X_11440	genus.Romboutsia.id.11347	-0.007294247	0.02626	0.807267807
4-androsten-3beta,17beta-diol disulf	genus.Romboutsia.id.11347	-0.036454475	0.033907	0.394796016
Propionylcarnitine	genus.Romboutsia.id.11347	-0.015455291	0.012409	0.339076064
X_12442	genus.Romboutsia.id.11347	0.032411562	0.021846	0.276165019
Palmitoylcarnitine	genus.Romboutsia.id.11347	0.022685829	0.017071	0.315204604
Myeloproliferative neoplasms	lysine	NA	NA	NA
Myeloproliferative neoplasms	betaine	-0.306394763	0.223237	0.400854053
Myeloproliferative neoplasms	palmitoylcarnitine	0.506960836	2.005202	0.812866299
Myeloproliferative neoplasms	acetylcarnitine	-0.034042959	0.077529	0.669925098
Myeloproliferative neoplasms	propionylcarnitine	-0.026260078	0.05219	0.623957581
Myeloproliferative neoplasms	3-dehydrocarnitine*	0.036992059	0.10275	0.725650277
Myeloproliferative neoplasms	X-11423	NA	NA	NA
Myeloproliferative neoplasms	X-11440	-0.008702935	0.063302	0.89729154
Myeloproliferative neoplasms	X-12092	0.020442266	0.036794	0.608102334
Myeloproliferative neoplasms	X-12442	0.060269855	1.136069	0.966258158
Myeloproliferative neoplasms	epiandrosterone sulfate	-0.090394888	0.051243	0.175920472
Myeloproliferative neoplasms	stearoylcarnitine	0.639584234	0.900616	0.516813032
Myeloproliferative neoplasms	X-12798	0.042498403	0.034323	0.250740823
Myeloproliferative neoplasms	X-12850	-0.154192423	0.086763	0.150177397
Myeloproliferative neoplasms	oleoylcarnitine	0.255017653	0.409749	0.567417116
Myeloproliferative neoplasms	succinylcarnitine	-0.008991523	0.04637	0.851755
Myeloproliferative neoplasms	4-androsten-3beta,17beta-diol disulfate 1*	0.202724149	0.131251	0.220158206

Supplementary Table 7. Heterogeneity of all Mendelian randomization results

Outcome	Exposure	Method	Q	Q_df	Q_pval
Myeloproliferative neoplasms	genus.Streptococcus.id.1853	MR Egger	5.439868	8	0.709692579
Myeloproliferative neoplasms	genus.Streptococcus.id.1853	Inverse variance weighted	5.511904	9	0.787598962
Myeloproliferative neoplasms	genus.Romboutsia.id.11347	MR Egger	12.62829	9	0.180159609

Myeloproliferative neoplasms	genus.Romboutsia.id.11347	Inverse variance weighted	12.64188	10	0.244389719
Myeloproliferative neoplasms	family.Lachnospiraceae.id.1987	MR Egger	18.11875	13	0.153062609
Myeloproliferative neoplasms	family.Lachnospiraceae.id.1987	Inverse variance weighted	18.40626	14	0.188899237
Myeloproliferative neoplasms	family.Desulfovibrionaceae.id.3169	MR Egger	8.672024	6	0.192880607
Myeloproliferative neoplasms	family.Desulfovibrionaceae.id.3169	Inverse variance weighted	8.67308	7	0.276987295
Myeloproliferative neoplasms	genus.Collinsella.id.815	MR Egger	6.394816	7	0.49447718
Myeloproliferative neoplasms	genus.Collinsella.id.815	Inverse variance weighted	7.541703	8	0.479466461
Myeloproliferative neoplasms	genus..Eubacteriumhalliigroup.id.11338	MR Egger	11.98924	11	0.364452906
Myeloproliferative neoplasms	genus..Eubacteriumhalliigroup.id.11338	Inverse variance weighted	15.38271	12	0.221171396
Myeloproliferative neoplasms	genus..Eubacteriumxylanophilumgroup.id.14375	MR Egger	3.714871	5	0.591152444
Myeloproliferative neoplasms	genus..Eubacteriumxylanophilumgroup.id.14375	Inverse variance weighted	5.585695	6	0.471160368
Lysine	family.Desulfovibrionaceae.id.3169	MR Egger	1.803217	4	0.77189367
Lysine	family.Desulfovibrionaceae.id.3169	Inverse variance weighted	3.316947	5	0.65124815
X_11423	family.Desulfovibrionaceae.id.3169	MR Egger	18.22544	4	0.00111497
X_11423	family.Desulfovibrionaceae.id.3169	Inverse variance weighted	18.34137	5	0.002547646
Oleoylcarnitine	family.Desulfovibrionaceae.id.3169	MR Egger	2.690233	4	0.610924717
Oleoylcarnitine	family.Desulfovibrionaceae.id.3169	Inverse variance weighted	3.272067	5	0.658120914
Acetylcarnitine	family.Desulfovibrionaceae.id.3169	MR Egger	3.581983	4	0.465522619
Acetylcarnitine	family.Desulfovibrionaceae.id.3169	Inverse variance weighted	4.222159	5	0.51789392
X_12798	family.Desulfovibrionaceae.id.3169	MR Egger	2.020874	4	0.731919341
X_12798	family.Desulfovibrionaceae.id.3169	Inverse variance weighted	4.340943	5	0.501442367
X_12850	family.Desulfovibrionaceae.id.3169	MR Egger	1.604227	4	0.808032248
X_12850	family.Desulfovibrionaceae.id.3169	Inverse variance weighted	1.628943	5	0.897727281
Epiandrosterone sulfate	family.Desulfovibrionaceae.id.3169	MR Egger	2.597455	4	0.627273966
Epiandrosterone sulfate	family.Desulfovibrionaceae.id.3169	Inverse variance weighted	2.909558	5	0.713927025
Stearoylcarnitine	family.Desulfovibrionaceae.id.3169	MR Egger	2.174403	4	0.703718185
Stearoylcarnitine	family.Desulfovibrionaceae.id.3169	Inverse variance weighted	2.190707	5	0.822177168
Betaine	family.Desulfovibrionaceae.id.3169	MR Egger	8.109148	4	0.087660815
Betaine	family.Desulfovibrionaceae.id.3169	Inverse variance weighted	9.186617	5	0.10184817
X_12092	family.Desulfovibrionaceae.id.3169	MR Egger	4.711148	4	0.318239503
X_12092	family.Desulfovibrionaceae.id.3169	Inverse variance weighted	5.080822	5	0.406096525
3-dehydrocarnitine	family.Desulfovibrionaceae.id.3169	MR Egger	2.255866	4	0.688815405
3-dehydrocarnitine	family.Desulfovibrionaceae.id.3169	Inverse variance weighted	2.25587	5	0.812726091
Succinylcarnitine	family.Desulfovibrionaceae.id.3169	MR Egger	1.952479	4	0.744499025
Succinylcarnitine	family.Desulfovibrionaceae.id.3169	Inverse variance weighted	1.952514	5	0.855675871
X_11440	family.Desulfovibrionaceae.id.3169	MR Egger	0.460495	4	0.977231065
X_11440	family.Desulfovibrionaceae.id.3169	Inverse variance weighted	0.475141	5	0.99300526
4-androsten-3beta,17beta-diol disulf	family.Desulfovibrionaceae.id.3169	MR Egger	2.556236	4	0.634594933
4-androsten-3beta,17beta-diol disulf	family.Desulfovibrionaceae.id.3169	Inverse variance weighted	2.75968	5	0.736976553
Propionylcarnitine	family.Desulfovibrionaceae.id.3169	MR Egger	4.827623	4	0.305446028
Propionylcarnitine	family.Desulfovibrionaceae.id.3169	Inverse variance weighted	4.835802	5	0.436246148
X_12442	family.Desulfovibrionaceae.id.3169	MR Egger	7.869752	4	0.096467579
X_12442	family.Desulfovibrionaceae.id.3169	Inverse variance weighted	7.919186	5	0.160746061
Palmitoylcarnitine	family.Desulfovibrionaceae.id.3169	MR Egger	8.31955	4	0.080549605
Palmitoylcarnitine	family.Desulfovibrionaceae.id.3169	Inverse variance weighted	8.601384	5	0.126059478
Lysine	genus..Eubacteriumhalliigroup.id.11338	MR Egger	3.806408	5	0.577611433
Lysine	genus..Eubacteriumhalliigroup.id.11338	Inverse variance weighted	6.08819	6	0.41338395
X_11423	genus..Eubacteriumhalliigroup.id.11338	MR Egger	7.11209	5	0.212436606
X_11423	genus..Eubacteriumhalliigroup.id.11338	Inverse variance weighted	8.738409	6	0.188832988
Oleoylcarnitine	genus..Eubacteriumhalliigroup.id.11338	MR Egger	10.85169	5	0.054401089
Oleoylcarnitine	genus..Eubacteriumhalliigroup.id.11338	Inverse variance weighted	11.85125	6	0.065370735
Acetylcarnitine	genus..Eubacteriumhalliigroup.id.11338	MR Egger	1.39372	5	0.924998987
Acetylcarnitine	genus..Eubacteriumhalliigroup.id.11338	Inverse variance weighted	1.458705	6	0.962190322
X_12798	genus..Eubacteriumhalliigroup.id.11338	MR Egger	2.317421	5	0.803704792
X_12798	genus..Eubacteriumhalliigroup.id.11338	Inverse variance weighted	2.317893	6	0.888265789
X_12850	genus..Eubacteriumhalliigroup.id.11338	MR Egger	5.982968	5	0.307879772
X_12850	genus..Eubacteriumhalliigroup.id.11338	Inverse variance weighted	7.450131	6	0.281215599
Epiandrosterone sulfate	genus..Eubacteriumhalliigroup.id.11338	MR Egger	5.095336	5	0.404356522
Epiandrosterone sulfate	genus..Eubacteriumhalliigroup.id.11338	Inverse variance weighted	5.172962	6	0.521828717
Stearoylcarnitine	genus..Eubacteriumhalliigroup.id.11338	MR Egger	2.758537	5	0.737151928
Stearoylcarnitine	genus..Eubacteriumhalliigroup.id.11338	Inverse variance weighted	2.958504	6	0.814036715
Betaine	genus..Eubacteriumhalliigroup.id.11338	MR Egger	4.282709	5	0.509471301
Betaine	genus..Eubacteriumhalliigroup.id.11338	Inverse variance weighted	5.654123	6	0.463027955
X_12092	genus..Eubacteriumhalliigroup.id.11338	MR Egger	6.486537	5	0.261710965
X_12092	genus..Eubacteriumhalliigroup.id.11338	Inverse variance weighted	7.921359	6	0.243921559
3-dehydrocarnitine	genus..Eubacteriumhalliigroup.id.11338	MR Egger	7.772081	5	0.16925209
3-dehydrocarnitine	genus..Eubacteriumhalliigroup.id.11338	Inverse variance weighted	7.774808	6	0.255070085
Succinylcarnitine	genus..Eubacteriumhalliigroup.id.11338	MR Egger	0.96759	5	0.965137256
Succinylcarnitine	genus..Eubacteriumhalliigroup.id.11338	Inverse variance weighted	0.974488	6	0.986560946
X_11440	genus..Eubacteriumhalliigroup.id.11338	MR Egger	2.28022	5	0.80916759
X_11440	genus..Eubacteriumhalliigroup.id.11338	Inverse variance weighted	2.857143	6	0.826551526
4-androsten-3beta,17beta-diol disulf	genus..Eubacteriumhalliigroup.id.11338	MR Egger	4.666381	5	0.457935399
4-androsten-3beta,17beta-diol disulf	genus..Eubacteriumhalliigroup.id.11338	Inverse variance weighted	4.833923	6	0.565283566
Propionylcarnitine	genus..Eubacteriumhalliigroup.id.11338	MR Egger	4.741357	5	0.448253494
Propionylcarnitine	genus..Eubacteriumhalliigroup.id.11338	Inverse variance weighted	5.007011	6	0.542914239
X_12442	genus..Eubacteriumhalliigroup.id.11338	MR Egger	2.482392	5	0.779144525
X_12442	genus..Eubacteriumhalliigroup.id.11338	Inverse variance weighted	2.539479	6	0.864023385
Palmitoylcarnitine	genus..Eubacteriumhalliigroup.id.11338	MR Egger	13.81334	5	0.016839637
Palmitoylcarnitine	genus..Eubacteriumhalliigroup.id.11338	Inverse variance weighted	13.87519	6	0.031061872
Lysine	genus.Collinsella.id.815	MR Egger	2.969648	1	0.084840314
Lysine	genus.Collinsella.id.815	Inverse variance weighted	3.865963	2	0.14471605
X_11423	genus.Collinsella.id.815	MR Egger	0.149976	1	0.698557907
X_11423	genus.Collinsella.id.815	Inverse variance weighted	0.7645	2	0.682324451
Oleoylcarnitine	genus.Collinsella.id.815	MR Egger	0.570942	1	0.449884669
Oleoylcarnitine	genus.Collinsella.id.815	Inverse variance weighted	1.266783	2	0.530788564
Acetylcarnitine	genus.Collinsella.id.815	MR Egger	0.923888	1	0.336456135
Acetylcarnitine	genus.Collinsella.id.815	Inverse variance weighted	0.953779	2	0.620711126
X_12798	genus.Collinsella.id.815	MR Egger	0.265323	1	0.606486502
X_12798	genus.Collinsella.id.815	Inverse variance weighted	0.316618	2	0.853586113
X_12850	genus.Collinsella.id.815	MR Egger	7.267076	1	0.007023003
X_12850	genus.Collinsella.id.815	Inverse variance weighted	7.273309	2	0.026340316
Epiandrosterone sulfate	genus.Collinsella.id.815	MR Egger	1.768793	1	0.18353152
Epiandrosterone sulfate	genus.Collinsella.id.815	Inverse variance weighted	2.057381	2	0.357474695
Stearoylcarnitine	genus.Collinsella.id.815	MR Egger	7.351528	1	0.006700578
Stearoylcarnitine	genus.Collinsella.id.815	Inverse variance weighted	7.754743	2	0.020705177
Betaine	genus.Collinsella.id.815	MR Egger	0.027135	1	0.869158764
Betaine	genus.Collinsella.id.815	Inverse variance weighted	2.306961	2	0.315536703
X_12092	genus.Collinsella.id.815	MR Egger	0.020463	1	0.886252824
X_12092	genus.Collinsella.id.815	Inverse variance weighted	0.434112	2	0.804884826
3-dehydrocarnitine	genus.Collinsella.id.815	MR Egger	0.170294	1	0.679850961
3-dehydrocarnitine	genus.Collinsella.id.815	Inverse variance weighted	0.385403	2	0.824728045
Succinylcarnitine	genus.Collinsella.id.815	MR Egger	0.824611	1	0.363835368
Succinylcarnitine	genus.Collinsella.id.815	Inverse variance weighted	2.498889	2	0.286663936
X_11440	genus.Collinsella.id.815	MR Egger	0.783757	1	0.375994616
X_11440	genus.Collinsella.id.815	Inverse variance weighted	0.82745	2	0.661182807
4-androsten-3beta,17beta-diol disulf	genus.Collinsella.id.815	MR Egger	0.780577	1	0.376964723
4-androsten-3beta,17beta-diol disulf	genus.Collinsella.id.815	Inverse variance weighted	1.081842	2	0.582211799
Propionylcarnitine	genus.Collinsella.id.815	MR Egger	0.507611	1	0.476174815
Propionylcarnitine	genus.Collinsella.id.815	Inverse variance weighted	0.512956	2	0.77377219
X_12442	genus.Collinsella.id.815	MR Egger	0.243369	1	0.621783399
X_12442	genus.Collinsella.id.815	Inverse variance weighted	0.487407	2	0.783719831
Palmitoylcarnitine	genus.Collinsella.id.815	MR Egger	0.737745	1	0.390384295
Palmitoylcarnitine	genus.Collinsella.id.815	Inverse variance weighted	2.190225	2	0.334502002
Lysine	genus.Streptococcus.id.1853	MR Egger	1.0555	3	0.787825878
Lysine	genus.Streptococcus.id.1853	Inverse variance weighted	3.235719	4	0.519181109
X_11423	genus.Streptococcus.id.1853	MR Egger	0.439334	3	0.932002328
X_11423	genus.Streptococcus.id.1853	Inverse variance weighted	0.665768	4	0.955482348
Oleoylcarnitine	genus.Streptococcus.id.1853	MR Egger	1.444767	3	0.6950755
Oleoylcarnitine	genus.Streptococcus.id.1853	Inverse variance weighted	1.8919	4	0.755632786
Acetylcarnitine	genus.Streptococcus.id.1853	MR Egger	1.798817	3	0.615192353
Acetylcarnitine	genus.Streptococcus.id.1853	Inverse variance weighted	1.811915	4	0.770301639
X_12798	genus.Streptococcus.id.1853	MR Egger	3.565819	3	0.31232539
X_12798	genus.Streptococcus.id.1853	Inverse variance weighted	3.693278	4	0.449104304
X_12850	genus.Streptococcus.id.1853	MR Egger	8.337487	3	0.039528264
X_12850	genus.Streptococcus.id.1853	Inverse variance weighted	8.696738	4	0.069143075
Epiandrosterone sulfate	genus.Streptococcus.id.1853	MR Egger	1.710137	3	0.634682379
Epiandrosterone sulfate	genus.Streptococcus.id.1853	Inverse variance weighted	1.930054	4	0.74862204
Stearoylcarnitine	genus.Streptococcus.id.1853	MR Egger	1.86485	3	0.600925467
Stearoylcarnitine	genus.Streptococcus.id.1853	Inverse variance weighted	3.43241	4	0.488229761

Betaine	genus.Streptococcus.id.1853	MR Egger	4.219605	3	0.238706351
Betaine	genus.Streptococcus.id.1853	Inverse variance weighted	4.667929	4	0.323097097
X_12092	genus.Streptococcus.id.1853	MR Egger	6.11251	3	0.106262864
X_12092	genus.Streptococcus.id.1853	Inverse variance weighted	13.85918	4	0.007758324
3-dehydrocarnitine	genus.Streptococcus.id.1853	MR Egger	1.690006	3	0.639158074
3-dehydrocarnitine	genus.Streptococcus.id.1853	Inverse variance weighted	4.525539	4	0.33953
Succinylcarnitine	genus.Streptococcus.id.1853	MR Egger	2.248504	3	0.522457848
Succinylcarnitine	genus.Streptococcus.id.1853	Inverse variance weighted	2.325359	4	0.676156032
X_11440	genus.Streptococcus.id.1853	MR Egger	0.65914	3	0.882769878
X_11440	genus.Streptococcus.id.1853	Inverse variance weighted	0.66489	4	0.955587022
4-androsten-3beta,17beta-diol disulf	genus.Streptococcus.id.1853	MR Egger	0.675203	3	0.879020253
4-androsten-3beta,17beta-diol disulf	genus.Streptococcus.id.1853	Inverse variance weighted	0.86832	4	0.929061167
Propionylcarnitine	genus.Streptococcus.id.1853	MR Egger	1.170872	3	0.759998666
Propionylcarnitine	genus.Streptococcus.id.1853	Inverse variance weighted	1.24854	4	0.870043964
X_12442	genus.Streptococcus.id.1853	MR Egger	0.042654	3	0.997686814
X_12442	genus.Streptococcus.id.1853	Inverse variance weighted	0.319487	4	0.988521766
Palmitoylcarnitine	genus.Streptococcus.id.1853	MR Egger	0.579232	3	0.901169911
Palmitoylcarnitine	genus.Streptococcus.id.1853	Inverse variance weighted	1.234747	4	0.872345211
Lysine	family.Lachnospiraceae.id.1987	MR Egger	4.481993	4	0.344688038
Lysine	family.Lachnospiraceae.id.1987	Inverse variance weighted	4.535784	5	0.475109931
X_11423	family.Lachnospiraceae.id.1987	MR Egger	1.466415	4	0.832573572
X_11423	family.Lachnospiraceae.id.1987	Inverse variance weighted	5.214498	5	0.390267455
Oleoylcarnitine	family.Lachnospiraceae.id.1987	MR Egger	10.78977	4	0.029031185
Oleoylcarnitine	family.Lachnospiraceae.id.1987	Inverse variance weighted	12.03465	5	0.034316112
Acetylcarnitine	family.Lachnospiraceae.id.1987	MR Egger	5.261374	4	0.261514632
Acetylcarnitine	family.Lachnospiraceae.id.1987	Inverse variance weighted	6.937836	5	0.225304757
X_12798	family.Lachnospiraceae.id.1987	MR Egger	1.111894	4	0.892380575
X_12798	family.Lachnospiraceae.id.1987	Inverse variance weighted	1.193029	5	0.945544341
X_12850	family.Lachnospiraceae.id.1987	MR Egger	8.652737	4	0.070390382
X_12850	family.Lachnospiraceae.id.1987	Inverse variance weighted	10.21731	5	0.069306851
Epiandrosterone sulfate	family.Lachnospiraceae.id.1987	MR Egger	7.16892	4	0.127226346
Epiandrosterone sulfate	family.Lachnospiraceae.id.1987	Inverse variance weighted	8.389128	5	0.136053952
Stearoylcarnitine	family.Lachnospiraceae.id.1987	MR Egger	2.059517	4	0.724813008
Stearoylcarnitine	family.Lachnospiraceae.id.1987	Inverse variance weighted	4.621247	5	0.46382662
Betaine	family.Lachnospiraceae.id.1987	MR Egger	7.394278	4	0.116462563
Betaine	family.Lachnospiraceae.id.1987	Inverse variance weighted	7.626325	5	0.178067676
X_12092	family.Lachnospiraceae.id.1987	MR Egger	4.953328	4	0.292119898
X_12092	family.Lachnospiraceae.id.1987	Inverse variance weighted	4.959751	5	0.420812007
3-dehydrocarnitine	family.Lachnospiraceae.id.1987	MR Egger	3.602383	4	0.462482425
3-dehydrocarnitine	family.Lachnospiraceae.id.1987	Inverse variance weighted	3.662335	5	0.598978895
Succinylcarnitine	family.Lachnospiraceae.id.1987	MR Egger	5.772321	4	0.216808885
Succinylcarnitine	family.Lachnospiraceae.id.1987	Inverse variance weighted	6.147376	5	0.292141604
X_11440	family.Lachnospiraceae.id.1987	MR Egger	1.516092	4	0.823787224
X_11440	family.Lachnospiraceae.id.1987	Inverse variance weighted	2.7669	5	0.735868894
4-androsten-3beta,17beta-diol disulf	family.Lachnospiraceae.id.1987	MR Egger	1.148294	4	0.886536041
4-androsten-3beta,17beta-diol disulf	family.Lachnospiraceae.id.1987	Inverse variance weighted	4.539078	5	0.47467207
Propionylcarnitine	family.Lachnospiraceae.id.1987	MR Egger	10.22967	4	0.036731639
Propionylcarnitine	family.Lachnospiraceae.id.1987	Inverse variance weighted	14.98099	5	0.010443859
X_12442	family.Lachnospiraceae.id.1987	MR Egger	10.74061	4	0.029639227
X_12442	family.Lachnospiraceae.id.1987	Inverse variance weighted	12.22083	5	0.031884098
Palmitoylcarnitine	family.Lachnospiraceae.id.1987	MR Egger	5.177523	4	0.269562721
Palmitoylcarnitine	family.Lachnospiraceae.id.1987	Inverse variance weighted	7.236865	5	0.203611932
Lysine	genus..Eubacteriumxylanophilumgroup.id.14375	MR Egger	0.227588	2	0.89244198
Lysine	genus..Eubacteriumxylanophilumgroup.id.14375	Inverse variance weighted	0.231135	3	0.972413028
X_11423	genus..Eubacteriumxylanophilumgroup.id.14375	MR Egger	1.070351	2	0.585566581
X_11423	genus..Eubacteriumxylanophilumgroup.id.14375	Inverse variance weighted	1.17327	3	0.75942203
Oleoylcarnitine	genus..Eubacteriumxylanophilumgroup.id.14375	MR Egger	2.799912	2	0.246607843
Oleoylcarnitine	genus..Eubacteriumxylanophilumgroup.id.14375	Inverse variance weighted	3.004634	3	0.390911248
Acetylcarnitine	genus..Eubacteriumxylanophilumgroup.id.14375	MR Egger	3.74747	2	0.153549047
Acetylcarnitine	genus..Eubacteriumxylanophilumgroup.id.14375	Inverse variance weighted	4.566707	3	0.206416781
X_12798	genus..Eubacteriumxylanophilumgroup.id.14375	MR Egger	0.870229	2	0.647190653
X_12798	genus..Eubacteriumxylanophilumgroup.id.14375	Inverse variance weighted	3.385707	3	0.33589087
X_12850	genus..Eubacteriumxylanophilumgroup.id.14375	MR Egger	0.816672	2	0.664755587
X_12850	genus..Eubacteriumxylanophilumgroup.id.14375	Inverse variance weighted	2.721428	3	0.436598111
Epiandrosterone sulfate	genus..Eubacteriumxylanophilumgroup.id.14375	MR Egger	0.07736	2	0.96205829
Epiandrosterone sulfate	genus..Eubacteriumxylanophilumgroup.id.14375	Inverse variance weighted	5.2514	3	0.154287247
Stearoylcarnitine	genus..Eubacteriumxylanophilumgroup.id.14375	MR Egger	0.370734	2	0.830799168
Stearoylcarnitine	genus..Eubacteriumxylanophilumgroup.id.14375	Inverse variance weighted	0.702338	3	0.872653901
Betaine	genus..Eubacteriumxylanophilumgroup.id.14375	MR Egger	2.169189	2	0.338038839
Betaine	genus..Eubacteriumxylanophilumgroup.id.14375	Inverse variance weighted	2.79618	3	0.424129149
X_12092	genus..Eubacteriumxylanophilumgroup.id.14375	MR Egger	2.397446	2	0.301579049
X_12092	genus..Eubacteriumxylanophilumgroup.id.14375	Inverse variance weighted	4.166176	3	0.244070209
3-dehydrocarnitine	genus..Eubacteriumxylanophilumgroup.id.14375	MR Egger	3.95074	2	0.138709993
3-dehydrocarnitine	genus..Eubacteriumxylanophilumgroup.id.14375	Inverse variance weighted	5.709177	3	0.126649277
Succinylcarnitine	genus..Eubacteriumxylanophilumgroup.id.14375	MR Egger	0.832457	2	0.659529604
Succinylcarnitine	genus..Eubacteriumxylanophilumgroup.id.14375	Inverse variance weighted	2.845321	3	0.416093441
X_11440	genus..Eubacteriumxylanophilumgroup.id.14375	MR Egger	0.335733	2	0.845466489
X_11440	genus..Eubacteriumxylanophilumgroup.id.14375	Inverse variance weighted	5.56717	3	0.134675866
4-androsten-3beta,17beta-diol disulf	genus..Eubacteriumxylanophilumgroup.id.14375	MR Egger	0.579052	2	0.7486183
4-androsten-3beta,17beta-diol disulf	genus..Eubacteriumxylanophilumgroup.id.14375	Inverse variance weighted	2.368702	3	0.49948729
Propionylcarnitine	genus..Eubacteriumxylanophilumgroup.id.14375	MR Egger	0.681573	2	0.711210592
Propionylcarnitine	genus..Eubacteriumxylanophilumgroup.id.14375	Inverse variance weighted	1.518153	3	0.678087058
X_12442	genus..Eubacteriumxylanophilumgroup.id.14375	MR Egger	3.553308	2	0.16920338
X_12442	genus..Eubacteriumxylanophilumgroup.id.14375	Inverse variance weighted	4.929066	3	0.177065606
Palmitoylcarnitine	genus..Eubacteriumxylanophilumgroup.id.14375	MR Egger	0.306299	2	0.858001293
Palmitoylcarnitine	genus..Eubacteriumxylanophilumgroup.id.14375	Inverse variance weighted	1.256684	3	0.739443668
Lysine	genus.Romboutsia.id.11347	MR Egger	6.146808	2	0.046263399
Lysine	genus.Romboutsia.id.11347	Inverse variance weighted	6.46675	3	0.090983073
X_11423	genus.Romboutsia.id.11347	MR Egger	0.431991	2	0.805738749
X_11423	genus.Romboutsia.id.11347	Inverse variance weighted	1.396105	3	0.706447898
Oleoylcarnitine	genus.Romboutsia.id.11347	MR Egger	2.589147	2	0.274014711
Oleoylcarnitine	genus.Romboutsia.id.11347	Inverse variance weighted	3.491434	3	0.321874791
Acetylcarnitine	genus.Romboutsia.id.11347	MR Egger	0.918669	2	0.631703901
Acetylcarnitine	genus.Romboutsia.id.11347	Inverse variance weighted	0.987717	3	0.804223926
X_12798	genus.Romboutsia.id.11347	MR Egger	1.377102	2	0.502303315
X_12798	genus.Romboutsia.id.11347	Inverse variance weighted	1.447416	3	0.694458801
X_12850	genus.Romboutsia.id.11347	MR Egger	1.158168	2	0.560411352
X_12850	genus.Romboutsia.id.11347	Inverse variance weighted	1.181661	3	0.757405993
Epiandrosterone sulfate	genus.Romboutsia.id.11347	MR Egger	0.321363	2	0.851563046
Epiandrosterone sulfate	genus.Romboutsia.id.11347	Inverse variance weighted	2.266039	3	0.519058172
Stearoylcarnitine	genus.Romboutsia.id.11347	MR Egger	0.375672	2	0.828750574
Stearoylcarnitine	genus.Romboutsia.id.11347	Inverse variance weighted	0.547923	3	0.908243616
Betaine	genus.Romboutsia.id.11347	MR Egger	1.044885	2	0.593070185
Betaine	genus.Romboutsia.id.11347	Inverse variance weighted	1.206363	3	0.751478601
X_12092	genus.Romboutsia.id.11347	MR Egger	0.521719	2	0.770389111
X_12092	genus.Romboutsia.id.11347	Inverse variance weighted	0.521743	3	0.914089355
3-dehydrocarnitine	genus.Romboutsia.id.11347	MR Egger	3.356897	2	0.186663355
3-dehydrocarnitine	genus.Romboutsia.id.11347	Inverse variance weighted	3.365603	3	0.338615735
Succinylcarnitine	genus.Romboutsia.id.11347	MR Egger	2.328515	2	0.312154395
Succinylcarnitine	genus.Romboutsia.id.11347	Inverse variance weighted	3.904908	3	0.271917078
X_11440	genus.Romboutsia.id.11347	MR Egger	0.185891	2	0.911243257
X_11440	genus.Romboutsia.id.11347	Inverse variance weighted	0.263048	3	0.966821375
4-androsten-3beta,17beta-diol disulf	genus.Romboutsia.id.11347	MR Egger	0.337073	2	0.844900635
4-androsten-3beta,17beta-diol disulf	genus.Romboutsia.id.11347	Inverse variance weighted	1.493	3	0.683886888
Propionylcarnitine	genus.Romboutsia.id.11347	MR Egger	1.66953	2	0.433976446
Propionylcarnitine	genus.Romboutsia.id.11347	Inverse variance weighted	3.220795	3	0.358819459
X_12442	genus.Romboutsia.id.11347	MR Egger	0.215865	2	0.897688401
X_12442	genus.Romboutsia.id.11347	Inverse variance weighted	2.41699	3	0.4904798
Palmitoylcarnitine	genus.Romboutsia.id.11347	MR Egger	1.76025	2	0.414731101
Palmitoylcarnitine	genus.Romboutsia.id.11347	Inverse variance weighted	3.526336	3	0.317362432
Myeloproliferative neoplasms	lysine	Inverse variance weighted	0.063873	1	0.800475603
Myeloproliferative neoplasms	betaine	MR Egger	0.492344	1	0.482883415
Myeloproliferative neoplasms	betaine	Inverse variance weighted	2.376117	2	0.304812446
Myeloproliferative neoplasms	palmitoylcarnitine	MR Egger	0.748668	4	0.945194253
Myeloproliferative neoplasms	palmitoylcarnitine	Inverse variance weighted	0.812588	5	0.976223513
Myeloproliferative neoplasms	acetylcarnitine	MR Egger	1.895913	10	0.997077423
Myeloproliferative neoplasms	acetylcarnitine	Inverse variance weighted	2.088721	11	0.998159101
Myeloproliferative neoplasms	propionylcarnitine	MR Egger	2.311498	12	0.99875737
Myeloproliferative neoplasms	propionylcarnitine	Inverse variance weighted	2.564671	13	0.999103783

Myeloproliferative neoplasms	3-dehydrocarnitine*	MR Egger	4.117254	11	0.966360562
Myeloproliferative neoplasms	3-dehydrocarnitine*	Inverse variance weighted	4.246868	12	0.978558394
Myeloproliferative neoplasms	X-11423	Inverse variance weighted	0.179885	1	0.671472208
Myeloproliferative neoplasms	X-11440	MR Egger	0.91679	4	0.922139729
Myeloproliferative neoplasms	X-11440	Inverse variance weighted	0.935692	5	0.96758413
Myeloproliferative neoplasms	X-12092	MR Egger	2.541067	4	0.637297448
Myeloproliferative neoplasms	X-12092	Inverse variance weighted	2.849741	5	0.723137858
Myeloproliferative neoplasms	X-12442	MR Egger	0.041826	1	0.837951528
Myeloproliferative neoplasms	X-12442	Inverse variance weighted	0.044641	2	0.977926964
Myeloproliferative neoplasms	epiandrosterone sulfate	MR Egger	0.165059	3	0.983022546
Myeloproliferative neoplasms	epiandrosterone sulfate	Inverse variance weighted	3.276857	4	0.512607464
Myeloproliferative neoplasms	stearoylcarnitine	MR Egger	0.244176	4	0.99312699
Myeloproliferative neoplasms	stearoylcarnitine	Inverse variance weighted	0.748507	5	0.98020082
Myeloproliferative neoplasms	X-12798	MR Egger	3.988865	8	0.858126658
Myeloproliferative neoplasms	X-12798	Inverse variance weighted	5.522022	9	0.786637776
Myeloproliferative neoplasms	X-12850	MR Egger	0.201052	4	0.995273441
Myeloproliferative neoplasms	X-12850	Inverse variance weighted	3.359414	5	0.64475836
Myeloproliferative neoplasms	oleoylcarnitine	MR Egger	0.137731	4	0.997734861
Myeloproliferative neoplasms	oleoylcarnitine	Inverse variance weighted	0.525082	5	0.991176078
Myeloproliferative neoplasms	succinylcarnitine	MR Egger	2.168987	7	0.949898093
Myeloproliferative neoplasms	succinylcarnitine	Inverse variance weighted	2.206587	8	0.974014298
Myeloproliferative neoplasms	4-androsten-3beta,17beta-diol disulfate 1*	MR Egger	1.892683	3	0.594976554
Myeloproliferative neoplasms	4-androsten-3beta,17beta-diol disulfate 1*	Inverse variance weighted	4.27832	4	0.369647747

Supplementary Table 8. Five Mendelian randomization models estimate the causal effects of plasma metabolites on Myeloproliferative neoplasms

Notations: Green back means P-value < 0.05 in inverse-variance-weighted method

Outcome	Exposure	Method	Number of	Beta	Standard error	P-value	lo_ci	up_ci	OR	OR_lci95	OR_uci95
Myeloproliferative neoplasms	3-dehydrocarnitine*	Inverse variance weighted	13	-1.985847923	0.742570445	0.00748879	-3.441285994	-0.530409851	0.137264175	0.032023477	0.588363779
Myeloproliferative neoplasms	X-11440	Inverse variance weighted	6	-0.653224727	0.245571646	0.007813673	-1.134545152	-0.171904302	0.520365033	0.321568353	0.842059753
Myeloproliferative neoplasms	epiandrosterone sulfate	Inverse variance weighted	5	0.518481191	0.212276081	0.014586527	0.102420073	0.934542309	1.67947491	1.107848751	2.546047888
Myeloproliferative neoplasms	betaine	Inverse variance weighted	3	4.497525854	1.882032403	0.016861335	0.808742344	8.186309365	89.7946911	2.245082668	3591.443052
Myeloproliferative neoplasms	propionylcarnitine	Inverse variance weighted	14	-2.016033342	0.855519852	0.018448021	-3.692852252	-0.339214431	0.133182709	0.024900877	0.712329687
Myeloproliferative neoplasms	X-12850	Inverse variance weighted	6	-1.216778368	0.522370769	0.019841138	-2.240625075	-0.192931661	0.296182824	0.106391981	0.82453832
Myeloproliferative neoplasms	acetylcarnitine	Inverse variance weighted	12	-2.193125925	0.953535999	0.021448628	-4.062056483	-0.324195368	0.111567451	0.017213583	0.723108957
Myeloproliferative neoplasms	X-12442	Inverse variance weighted	3	1.957787923	0.857165954	0.022370099	0.277742654	3.637833192	7.083640162	1.320146418	38.00938839
Myeloproliferative neoplasms	4-androsten-3beta,17beta-diol disulfate 1*	Inverse variance weighted	5	-0.53212986	0.235498932	0.023847018	-0.993707767	-0.070551952	0.587352658	0.370201522	0.931879325
Myeloproliferative neoplasms	X-12092	Inverse variance weighted	6	-0.336581644	0.150907063	0.025721545	-0.632359487	-0.040803801	0.71420757	0.531336639	0.960017466
Myeloproliferative neoplasms	lysine	Inverse variance weighted	2	6.86861376	3.138268031	0.028621282	0.717608418	13.0196191	961.6146125	2.049525736	451178.8491
Myeloproliferative neoplasms	X-12798	Inverse variance weighted	10	-0.744711503	0.345762305	0.031253897	-1.422405621	-0.067017385	0.474871279	0.241133243	0.935178943
Myeloproliferative neoplasms	succinylcarnitine	Inverse variance weighted	9	-2.244075736	1.076621358	0.037126653	-4.354253598	-0.133897875	0.106025491	0.012852029	0.874679387
Myeloproliferative neoplasms	oleoylcarnitine	Inverse variance weighted	6	-2.238196984	1.07779655	0.037834665	-4.350678222	-0.125715746	0.106650624	0.012898062	0.881865485
Myeloproliferative neoplasms	stearoylcarnitine	Inverse variance weighted	6	-2.241827	1.108884034	0.043207713	-4.415239707	-0.068414293	0.106264182	0.012091655	0.933873496
Myeloproliferative neoplasms	palmitoylcarnitine	Inverse variance weighted	6	-2.302259032	1.147809107	0.044879123	-4.551964882	-0.052553183	0.100032611	0.010546461	0.94880386
Myeloproliferative neoplasms	X-11423	Inverse variance weighted	2	-6.742946197	3.376183507	0.045802416	-13.36026587	-0.125626524	0.001179168	1.58E-06	0.881944171
Myeloproliferative neoplasms	2-tetradecenoyl carnitine	Inverse variance weighted	4	-2.228974344	1.140408859	0.050637429	-4.464175709	0.00622702	0.107638774	0.011514183	1.006246448
Myeloproliferative neoplasms	pyroglutamine*	Inverse variance weighted	5	-1.630719723	0.902039511	0.070635443	-3.398717166	0.137277719	0.19578861	0.03341611	1.147146689
Myeloproliferative neoplasms	dihomo-linolenate (20:3n3 or n6)	Inverse variance weighted	10	1.637911677	0.929104938	0.077918704	-0.183134	3.458957355	5.144415088	0.832656572	31.78381999
Myeloproliferative neoplasms	1-linoleoylglycerophosphoethanolamine*	Inverse variance weighted	12	0.770774112	0.437983255	0.078437394	-0.087673068	1.629221293	2.161438803	0.916060317	5.099901844
Myeloproliferative neoplasms	1-arachidonoylglycerophosphocholine*	Inverse variance weighted	13	-0.611587201	0.348185363	0.079003262	-1.294030513	0.07085611	0.542489146	0.274163534	1.07342676
Myeloproliferative neoplasms	docosapentaenoate (n3 DPA; 22:5n3)	Inverse variance weighted	8	-1.411427027	0.807260241	0.080391954	-2.9936571	0.170803047	0.243795133	0.050103866	1.186257089
Myeloproliferative neoplasms	kynurenine	Inverse variance weighted	6	2.336486509	1.345113051	0.082383872	-0.299935071	4.97290809	10.34482619	0.740866323	144.4463401
Myeloproliferative neoplasms	X-12093	Inverse variance weighted	5	-0.541919994	0.313441641	0.08382086	-1.156265611	0.072425623	0.581630452	0.314659049	1.075112839
Myeloproliferative neoplasms	arachidonate (20:4n6)	Inverse variance weighted	11	-0.677673054	0.399130478	0.089531644	-1.459968791	0.104622684	0.507797236	0.232243523	1.110291601
Myeloproliferative neoplasms	X-18601	Inverse variance weighted	11	0.766025299	0.461970251	0.09728312	-0.139436393	1.671486991	2.151198867	0.86984835	5.320072821
Myeloproliferative neoplasms	hexanoylcarnitine	Inverse variance weighted	17	-0.725075591	0.444896043	0.103151368	-1.597071835	0.146920653	0.48428796	0.202488571	1.158262055
Myeloproliferative neoplasms	isovalerylcarnitine	Inverse variance weighted	11	-0.81159819	0.498989817	0.103847059	-1.789618231	0.166421851	0.444147666	0.167023922	1.181071232
Myeloproliferative neoplasms	carnitine	Inverse variance weighted	26	-1.824246848	1.142080726	0.110198596	-4.06272507	0.414231375	0.161339111	0.017202078	1.513207204
Myeloproliferative neoplasms	1-eicosatrienoylglycerophosphocholine*	Inverse variance weighted	11	1.225103829	0.779686613	0.116118265	-0.303081932	2.753289591	3.404519553	0.738538584	15.69417447
Myeloproliferative neoplasms	X-09789	Inverse variance weighted	2	1.567061101	0.998797622	0.116660156	-0.390582238	3.524704439	4.792542676	0.676662781	33.94373968
Myeloproliferative neoplasms	adrenate (22:4n6)	Inverse variance weighted	9	-0.884417094	0.587932222	0.132508231	-2.036764249	0.267930061	0.412954817	0.130450133	1.307255708
Myeloproliferative neoplasms	asparagine	Inverse variance weighted	2	-3.107079187	2.067861386	0.132953371	-7.160087504	0.94592913	0.044731417	0.000776987	2.575204968
Myeloproliferative neoplasms	X-12712	Inverse variance weighted	2	0.744272491	0.501677898	0.13792398	-0.23901619	1.727561172	2.104909537	0.787402134	5.626914085
Myeloproliferative neoplasms	X-12696	Inverse variance weighted	3	-1.833321022	1.271988515	0.149499065	-4.326418512	0.659776468	0.159881714	0.013214791	1.934359896
Myeloproliferative neoplasms	X-11261	Inverse variance weighted	5	-1.111896628	0.773191041	0.150416473	-2.627351069	0.403557813	0.328934503	0.072269646	1.497141783
Myeloproliferative neoplasms	eicosapentaenoate (EPA; 20:5n3)	Inverse variance weighted	9	-0.79113983	0.556654647	0.15524701	-1.882182937	0.299903278	0.453327784	0.152257374	1.349728252
Myeloproliferative neoplasms	androsterone sulfate	Inverse variance weighted	9	0.27582651	0.199271748	0.166305586	-0.114746117	0.666399137	1.317619251	0.891592476	1.947213034
Myeloproliferative neoplasms	stearidonate (18:4n3)	Inverse variance weighted	9	-0.81393257	0.589850211	0.167618343	-1.970038983	0.342173844	0.443112066	0.13945142	1.40800505
Myeloproliferative neoplasms	creatine	Inverse variance weighted	2	-1.665526296	1.216194602	0.170856446	-4.049267717	0.718215125	0.189091114	0.017435137	2.050769574
Myeloproliferative neoplasms	valine	Inverse variance weighted	2	7.52497873	5.531754587	0.173727405	-3.317260261	18.36721772	1853.773796	0.036252017	94794099.19
Myeloproliferative neoplasms	1,5-anhydroglucitol (1,5-AG)	Inverse variance weighted	5	-1.276378001	0.949960802	0.179074136	-3.138301174	0.585545171	0.279046177	0.04335639	1.79596983
Myeloproliferative neoplasms	glycine	Inverse variance weighted	2	-0.986148098	0.746462141	0.186469379	-2.449213895	0.476917698	0.373010728	0.086361449	1.611100842
Myeloproliferative neoplasms	X-13435	Inverse variance weighted	3	-1.772412598	1.361495746	0.192980385	-4.44094426	0.896119065	0.169922539	0.011784805	2.45007605
Myeloproliferative neoplasms	caprylate (8:0)	Inverse variance weighted	2	5.306962208	4.192094041	0.205532627	-2.909542113	13.52346653	201.7364632	0.054500679	746735.6574
Myeloproliferative neoplasms	X-10395	Inverse variance weighted	2	-3.706118418	2.944018556	0.208079527	-9.476394789	2.064157952	0.024572719	7.66E-05	7.878660894
Myeloproliferative neoplasms	X-12844	Inverse variance weighted	3	2.104883258	1.676397687	0.209261395	-1.180856209	5.390622724	8.206144949	0.307015756	219.3399314
Myeloproliferative neoplasms	X-11315	Inverse variance weighted	4	2.432514019	1.951895856	0.212679433	-1.393201859	6.258229897	11.38747445	0.248279076	522.2936078
Myeloproliferative neoplasms	1-eicosadienoylglycerophosphocholine*	Inverse variance weighted	3	1.978754171	1.676112249	0.237776828	-1.306425837	5.26393418	7.233725386	0.270786163	193.2402396
Myeloproliferative neoplasms	X-14208	Inverse variance weighted	3	0.694222638	0.607911657	0.253462549	-0.49728421	1.885729487	2.002152072	0.608180108	6.591160852
Myeloproliferative neoplasms	palmitoyl sphingomyelin	Inverse variance weighted	2	-4.118615693	3.616253908	0.25473747	-11.20647335	2.969241966	0.016267017	1.36E-05	19.47714966
Myeloproliferative neoplasms	X-11204	Inverse variance weighted	3	3.503689615	3.09638587	0.257827178	-2.565226691	9.572605921	33.23786091	0.076901747	14365.80374
Myeloproliferative neoplasms	1-palmitoylglycerophosphoethanolamine	Inverse variance weighted	6	1.134542231	1.066816025	0.287562323	-0.956417179	3.22550164	3.109749669	0.384267183	25.16619536
Myeloproliferative neoplasms	1-stearoylglycerophosphoethanolamine	Inverse variance weighted	3	1.359249707	1.285831196	0.290466821	-1.160979437	3.879478851	3.89327111	0.313179291	48.3989854
Myeloproliferative neoplasms	X-11381	Inverse variance weighted	4	-1.78436045	1.688276273	0.290551482	-5.093381945	1.524661045	0.16790441	0.006137229	4.593586285
Myeloproliferative neoplasms	1-arachidonoylglycerophosphoethanolamine*	Inverse variance weighted	18	-0.49152422	0.468735079	0.294353792	-1.410244974	0.427196535	0.611693328	0.244083482	1.532953911
Myeloproliferative neoplasms	3-methyl-2-oxovalerate	Inverse variance weighted	4	2.577240159	2.460412059	0.294876802	-2.245167477	7.399647795	13.16076637	0.105909801	1635.40833
Myeloproliferative neoplasms	X-13215	Inverse variance weighted	3	2.752091981	2.67372609	0.303334205	-2.488411154	7.992595116	15.67539023	0.083041802	2958.965865
Myeloproliferative neoplasms	X-12644	Inverse variance weighted	7	1.142152335	1.135502	0.314484491	-1.083431586	3.367736256	3.133505466	0.338432172	29.01277516
Myeloproliferative neoplasms	X-08988	Inverse variance weighted	2	-1.761346159	1.767896565	0.319106928	-5.226423425	1.703731108	0.17181342	0.005372707	5.494409429
Myeloproliferative neoplasms	X-12189	Inverse variance weighted	2	0.368098146	0.369905381	0.319680661	-0.3569164	1.093112691	1.444983851	0.699831001	2.983546494
Myeloproliferative neoplasms	citrulline	Inverse variance weighted	4	2.909182566	2.951569732	0.324310239	-2.875894109	8.694259241	18.34179923	0.05636572	5968.549673
Myeloproliferative neoplasms	uridine	Inverse variance weighted	3	4.815460794	4.915133682	0.327223746	-4.818201224	14.44912281	123.4036629	0.008081311	1884405.25
Myeloproliferative neoplasms	N-acetylglycine	Inverse variance weighted	3	-0.605853183	0.628449416	0.335023632	-1.837614037	0.625907672	0.545608724	0.159196811	1.869942482
Myeloproliferative neoplasms	X-08402	Inverse variance weighted	3	-0.788664328	0.823801883	0.338392099	-2.403316018	0.825987363	0.454451388	0.090417629	2.284134923
Myeloproliferative neoplasms	X-12556	Inverse variance weighted	2	2.015190079	2.157843458	0.350360243	-2.214183097	6.244563256	7.502153251	0.109242718	515.2041636
Myeloproliferative neoplasms	2-aminobutyrate	Inverse variance weighted	2	2.461847415	2.654928436	0.353784035	-2.74181232	7.665507149	11.72645517	0.064453431	2133.474497
Myeloproliferative neoplasms	HWESASXX*	Inverse variance weighted	2	-0.928413031	1.015994313	0.360823277	-2.919761885	1.062935823	0.395180352	0.053946531	2.894857314
Myeloproliferative neoplasms	X-11530	Inverse variance weighted	2	0.317281378	0.368506463	0.389242058	-0.404991289	1.039554045	1.373388959	0.666982621	2.827955593
Myeloproliferative neoplasms	X-11442	Inverse variance weighted	2	0.322951347	0.375093394	0.389244012	-0.412231705	1.0581344	1.38119815	0.66217083	2.880991195
Myeloproliferative neoplasms	bilirubin (E,Z or Z,E)*	Inverse variance weighted	2	0.439231441	0.51015196	0.389248187	-0.5606664	1.439129282	1.55151433	0.570828537	4.21702238
Myeloproliferative neoplasms	X-11441	Inverse variance weighted	2	0.323392961	0.375609774	0.389248388	-0.412802197	1.059588118	1.381808241	0.661793175	2.885182391
Myeloproliferative neoplasms	bilirubin (E,E)*	Inverse variance weighted	2	0.27873897	0.323751737	0.389257226	-0.355814435	0.913292375	1.321462357	0.700602615	2.492515334
Myeloproliferative neoplasms	biliverdin	Inverse variance weighted	2	0.315821225	0.36682603	0.389262046	-0.403157793	1.034800244	1.371385065	0.668206652	2.814543959
Myeloproliferative neoplasms	bilirubin (Z,Z)	Inverse variance weighted	2	0.213971098	0.248540938	0.389288236	-0.27316914	0.701111336	1.238586857	0.760964067	2.015991907
Myeloproliferative neoplasms	X-14205	Inverse variance weighted	3	0.659512857	0.775360989	0.394998183	-0.860194681	2.179220395	1.933850044	0.423079709	8.839412325
Myeloproliferative neoplasms	1-oleoylglycerophosphoethanolamine	Inverse variance weighted	2	1.346700751	1.74611219	0.440554784	-2.075679142	4.769080644	3.844719893	0.125471186	117.810882
Myeloproliferative neoplasms	isobutyrylcarnitine	Inverse variance weighted	5	-0.773875095	1.012265717	0.44457054	-2.7579159	1.21016571	0.46122232	0.063423812	3.354040403
Myeloproliferative neoplasms	bradykinin, des-arg(9)	Inverse variance weighted	6	-0.172054264	0.225427659	0.445323299	-0.613892475	0.269783947	0.841933485	0.54124	1.30968146
Myeloproliferative neoplasms	1-arachidonoylglycerophosphoinositol*	Inverse variance weighted	17	-0.370495727	0.487895913	0.447628548	-1.326771717	0.585780262	0.690392	0.265332448	1.796392096
Myeloproliferative neoplasms	5alpha-androstan-3beta,17beta-diol disulfate	Inverse variance weighted	10	0.244586529	0.325083949	0.451822816	-0.392578011	0.88175107	1.277093163	0.675313662	2.415125059
Myeloproliferative neoplasms	levulinate (4-oxovalerate)	Inverse variance weighted	2	2.847515829	3.797832877	0.453390913	-4.59623661	10.29126827	17.24488933	0.010089736	29474.13174
Myeloproliferative neoplasms	caprate (10:0)	Inverse variance weighted	2	3.300168406	4.579216802	0.471103942	-5.675096526	12.27543334	27.11720523	0.003430338	214364.5476
Myeloproliferative neoplasms	glutaroyl carnitine	Inverse variance weighted	11	-0.552729547	0.767367925	0.471344772	-2.05677068	0.951311585	0.575377146	0.127866225	2.589103263
Myeloproliferative neoplasms	3-(4-hydroxyphenyl)lactate	Inverse variance weighted	2	1.59945082	2.246910926	0.476561174	-2.804494595	6.003396235	4.950313064	0.060537359	404.8012617
Myeloproliferative neoplasms	X-14662	Inverse variance weighted	6	0.374061251	0.532724565	0.482575442	-0.670078896	1.418201398	1.453626184	0.511668208	4.129686095
Myeloproliferative neoplasms	X-03094	Inverse variance weighted	6	1.116953082	1.595524798	0.483893752	-2.010275522	4.244181686	3.055530056	0.133951763	69.69870138
Myeloproliferative neoplasms	X-11793	Inverse variance weighted	3	0.296892513	0.433683916	0.493607278	-0.553127963	1.146912988	1.345670649	0.575147952	3.148458563
Myeloproliferative neoplasms	10-undecenoate (11:1n1)	Inverse variance weighted	3	1.005321045	1.469904699	0.494015449	-1.875692164	3.886334255	2.73278448	0.153248857	48.7319199
Myeloproliferative neoplasms	malate	Inverse variance weighted	2	1.756129555	2.572778707	0.494871719	-3.286516711	6.798775821	5.789984154	0.037383842	896.7488384
Myeloproliferative neoplasms	X-13429	Inverse variance weighted	16	-0.11429087	0.169803212	0.50089743	-0.447105165	0.218523425	0.891998463	0.639476654	1.244238163
Myeloproliferative neoplasms	X-14086	Inverse variance weighted	2	0.779535726	1.167526094	0.504336838	-1.508815418	3.067886871	2.180459701	0.221171819	21.49642992
Myeloproliferative neoplasms	aspartylphenylalanine	Inverse variance weighted	2	0.593806082	0.895458422	0.50724677	-1.161292425	2.348904588	1.810867626	0.313081285	10.47409
Myeloproliferative neoplasms	cis-4-decenoyl carnitine	Inverse variance weighted	6	-0.371448174	0.582971042	0.52401801	-1.514071415	0.771175068	0.689734751	0.22001239	2.162305617
Myeloproliferative neoplasms	alanine	Inverse variance weighted	2	2.853913444	4.806693548	0.552687804	-6.567205909	12.2750328	17.35556917	0.00140572	214278.7031
Myeloproliferative neoplasms	butyrylcarnitine	Inverse variance weighted	12	0.204243074	0.347975366	0.557239346	-0.477788643	0.886274791	1.226596271	0.620153257	2.42607516
Myeloproliferative neoplasms	tryptophan	Inverse variance weighted	18	1.749245081	3.025932621	0.563206997	-4.181582856	7.680073017	5.750260054	0.015274311	2164.777832
Myeloproliferative neoplasms	X-12524	Inverse variance weighted	2	3.008390815	5.235373329	0.565543078	-7.25294091	13.26972254	20.25478	0.000708089	579385.0376
Myeloproliferative neoplasms	leucine	Inverse variance weighted	12	-1.685341804	2.9568509	0.568692197	-7.480769567	4.110085959	0.185381057	0.000563823	60.95195671
Myeloproliferative neoplasms	gamma-glutamyltyrosine	Inverse variance weighted	5	2.30369818	4.042325985	0.568749451	-5.619260751	10.22665711	10.01113707	0.003627322	27629.99152
Myeloproliferative neoplasms	X-11327	Inverse variance weighted	2	-2.858124637	5.042154796	0.570818901	-12.74074804	7.024498764	0.057376261	2.93E-06	1123.831113
Myeloproliferative neoplasms	X-13431	Inverse variance weighted	4	-0.339431568	0.603285687	0.57368128	-1.521871514	0.843008378	0.712175031	0.218302947	2.323345976
Myeloproliferative neoplasms	X-13548	Inverse variance weighted	2	-5.260274667	9.382900384	0.575054212	-23.65075942	13.13021009	0.005193878	5.35E-11	503938.7871
Myeloproliferative neoplasms	4-androsten-3beta,17beta-diol disulfate 2*	Inverse variance weighted	11	0.264493774	0.474010223	0.576850046	-0.664566263	1.193553811	1.302771312	0.514496636	3.298783653
Myeloproliferative neoplasms	hexadecanedioate	Inverse variance weighted	15	-0.140885243	0.256153067	0.582316545	-0.642945254	0.361174768	0.868588983	0.525741699	1.435014234
Myeloproliferative neoplasms	mannose	Inverse variance weighted	2	0.68635615	1.267834931	0.588258911	-1.798600314	3.171312614	1.986463953	0.165530417	23.83875491
Myeloproliferative neoplasms	octanoylcarnitine	Inverse variance weighted	7	-0.230735243	0.428733762	0.590453902	-1.071053416	0.60958293	0.793949642	0.342647377	1.83966397
Myeloproliferative neoplasms	myo-inositol	Inverse variance weighted	3	1.2880952	2.502953795	0.606811573	-3.617694239	6.193884639	3.625873409	0.026844502	489.7448977
Myeloproliferative neoplasms	X-12100	Inverse variance weighted	4	1.196843564	2.351401027	0.610758055	-3.411902449	5.805589576	3.309653707	0.032978401	332.1509639
Myeloproliferative neoplasms	1-linoleoylglycerol (1-monolinolein)	Inverse variance weighted	2	0.537267445	1.065853779	0.614210519	-1.551805962	2.626340852	1.71132418	0.211865008	13.82309649
Myeloproliferative neoplasms	X-12040	Inverse variance weighted	2	-0.095759549	0.191805494	0.617600864	-0.471698317	0.280179219	0.908682483	0.623941717	1.323366963

Myeloproliferative neoplasms	X-10510	Inverse variance weighted	4	-0.669223595	1.37475677	0.626404195	-3.363746865	2.025299675	0.512106025	0.034605354	7.578381655
Myeloproliferative neoplasms	X-11538	Inverse variance weighted	15	-0.100848131	0.20717374	0.626413911	-0.50690866	0.305212399	0.904070323	0.602354787	1.356913179
Myeloproliferative neoplasms	tetradecanedioate	Inverse variance weighted	16	-0.109480688	0.23039687	0.634656506	-0.561058553	0.342097177	0.896299474	0.570604729	1.407897107
Myeloproliferative neoplasms	serine	Inverse variance weighted	3	-0.84802737	1.821618429	0.641548286	-4.41839949	2.72234475	0.428258896	0.012053509	15.21595807
Myeloproliferative neoplasms	X-13496	Inverse variance weighted	2	4.851529159	10.47127446	0.643136499	-15.67216879	25.37522711	127.9358747	1.56E-07	1.0479E+11
Myeloproliferative neoplasms	X-12510	Inverse variance weighted	9	0.161882734	0.353062941	0.646586394	-0.530120629	0.853886098	1.175722361	0.588533971	2.348756638
Myeloproliferative neoplasms	4-methyl-2-oxopentanoate	Inverse variance weighted	3	1.544770514	3.614373833	0.669090599	-5.539402198	8.628943227	4.686895928	0.003928875	5591.166533
Myeloproliferative neoplasms	X-11593	Inverse variance weighted	6	-0.441605302	1.047859632	0.673437064	-2.495410181	1.612199578	0.643003378	0.08246262	5.013827411
Myeloproliferative neoplasms	X-11820	Inverse variance weighted	2	0.756042439	1.846991981	0.682292254	-2.864061843	4.376146721	2.129830585	0.057036615	79.53098713
Myeloproliferative neoplasms	X-02269	Inverse variance weighted	13	-0.147652099	0.362887471	0.684095225	-0.858911542	0.563607344	0.862731208	0.423622927	1.756999182
Myeloproliferative neoplasms	decanoylcarnitine	Inverse variance weighted	7	-0.190861861	0.483029985	0.692743553	-1.137600631	0.755876909	0.826246717	0.320587307	2.129478063
Myeloproliferative neoplasms	dehydroisoandrosterone sulfate (DHEA-S)	Inverse variance weighted	7	0.337447974	0.855408647	0.693221193	-1.339152975	2.014048922	1.401366699	0.262067552	7.493596995
Myeloproliferative neoplasms	cholesterol	Inverse variance weighted	3	-1.525365585	4.023357822	0.704593187	-9.411146916	6.360415746	0.217541512	8.18E-05	578.48681
Myeloproliferative neoplasms	N-acetylornithine	Inverse variance weighted	4	0.072879774	0.201833846	0.718033687	-0.322714565	0.468474112	1.075601213	0.724180531	1.597554643
Myeloproliferative neoplasms	ADpSGEGDFXAEGGGVR*	Inverse variance weighted	2	0.415259245	1.178091884	0.724474867	-1.893800848	2.724319337	1.514763384	0.150498698	15.24603299
Myeloproliferative neoplasms	erythronate*	Inverse variance weighted	3	2.022788577	5.901459411	0.731778466	-9.544071869	13.58964902	7.559375471	7.16E-05	797828.5654
Myeloproliferative neoplasms	ADSGEGDFXAEGGGVR*	Inverse variance weighted	2	-0.1987651	0.59346651	0.737683786	-1.36195946	0.96442926	0.819742428	0.256158353	2.623290012
Myeloproliferative neoplasms	X-03056	Inverse variance weighted	3	0.451221933	1.369491228	0.741791825	-2.232980874	3.135424741	1.57022973	0.107208379	22.99840213
Myeloproliferative neoplasms	X-12038	Inverse variance weighted	4	-0.741941222	2.259827845	0.742671549	-5.171203799	3.687321355	0.47618863	0.00567773	39.93772456
Myeloproliferative neoplasms	laurylcarnitine	Inverse variance weighted	3	0.384196939	1.203532935	0.749556444	-1.974727614	2.743121493	1.468434606	0.138799114	15.53540313
Myeloproliferative neoplasms	4-acetamidobutanoate	Inverse variance weighted	2	-0.621327587	1.978365055	0.753474602	-4.498923095	3.256267922	0.537230744	0.011120966	25.95249942
Myeloproliferative neoplasms	X-12456	Inverse variance weighted	12	-0.091568005	0.302892683	0.762414453	-0.685237665	0.502101655	0.91249926	0.503970439	1.652189957
Myeloproliferative neoplasms	X-11792	Inverse variance weighted	4	-0.101433812	0.351111324	0.772662806	-0.789612007	0.586744382	0.903540981	0.454020918	1.798124869
Myeloproliferative neoplasms	X-13477	Inverse variance weighted	3	0.459146484	1.704113162	0.787595518	-2.880915313	3.799208281	1.58272253	0.056083405	44.66580773
Myeloproliferative neoplasms	X-14625	Inverse variance weighted	2	-0.69265892	2.618128526	0.791346426	-5.824190832	4.438872992	0.50024419	0.002955194	84.67945344
Myeloproliferative neoplasms	5-oxoproline	Inverse variance weighted	2	-0.316697743	1.201095083	0.792031005	-2.670844106	2.03744862	0.728550931	0.069193794	7.671012543
Myeloproliferative neoplasms	X-12244	Inverse variance weighted	3	0.412260329	1.587173351	0.795060485	-2.698599438	3.523120096	1.510227541	0.067299704	33.89000374
Myeloproliferative neoplasms	citrate	Inverse variance weighted	6	0.545219119	2.20026402	0.804291372	-3.76729836	4.857736598	1.724986318	0.023114426	128.7324987
Myeloproliferative neoplasms	octadecanedioate	Inverse variance weighted	10	-0.161384727	0.694245026	0.816180161	-1.522104979	1.199335525	0.850964619	0.218251987	3.317911519
Myeloproliferative neoplasms	hydroxyisovaleroyl carnitine	Inverse variance weighted	2	0.377700018	1.625457333	0.816254233	-2.808196356	3.563596391	1.458925226	0.060313679	35.28988543
Myeloproliferative neoplasms	X-11787	Inverse variance weighted	6	0.197518303	0.908275536	0.82784584	-1.582701747	1.977738353	1.218375365	0.205419357	7.226380969
Myeloproliferative neoplasms	X-11491	Inverse variance weighted	9	0.061701237	0.285785013	0.829065009	-0.498437389	0.621839863	1.063644519	0.607479172	1.862351363
Myeloproliferative neoplasms	X-14626	Inverse variance weighted	10	0.102425617	0.476458994	0.8297887	-0.831434011	1.036285245	1.107854894	0.435424435	2.818726664
Myeloproliferative neoplasms	scyllo-inositol	Inverse variance weighted	2	0.228912094	1.140866778	0.840974195	-2.00718679	2.465010978	1.257231516	0.134366144	11.7636113
Myeloproliferative neoplasms	X-12855	Inverse variance weighted	6	0.222553947	1.112419183	0.84143129	-1.957787652	2.402895547	1.249263212	0.141170394	11.05514076
Myeloproliferative neoplasms	X-11247	Inverse variance weighted	3	0.146588898	0.733339129	0.841564757	-1.290755795	1.58393359	1.157877859	0.275062813	4.874090827
Myeloproliferative neoplasms	tyrosine	Inverse variance weighted	5	0.85626944	4.291318574	0.841844051	-7.554714964	9.267253844	2.354361205	0.000523635	10585.64211
Myeloproliferative neoplasms	alpha-hydroxyisovalerate	Inverse variance weighted	3	0.455770014	2.432862153	0.85139483	-4.312639805	5.224179834	1.577387526	0.013398134	185.708796
Myeloproliferative neoplasms	2-hydroxyisobutyrate	Inverse variance weighted	3	-0.526231473	2.924637219	0.857207121	-6.258520423	5.206057476	0.590827328	0.001914076	182.3736266
Myeloproliferative neoplasms	X-12728	Inverse variance weighted	3	-0.021986906	0.147650916	0.881623538	-0.311382701	0.267408889	0.978253044	0.732433519	1.306574581
Myeloproliferative neoplasms	X-11469	Inverse variance weighted	10	-0.062542914	0.428490574	0.883952173	-0.902384439	0.77729861	0.93937275	0.405601374	2.175587211
Myeloproliferative neoplasms	X-12063	Inverse variance weighted	20	-0.018553942	0.188649669	0.921653337	-0.388307294	0.35119941	0.981617123	0.678203903	1.420770614
Myeloproliferative neoplasms	laurate (12:0)	Inverse variance weighted	2	-0.394006796	4.061392574	0.922716282	-8.354336241	7.566322649	0.674349473	0.000235374	1932.022477
Myeloproliferative neoplasms	X-11445	Inverse variance weighted	9	0.028227026	0.336879396	0.933223632	-0.63205659	0.688510641	1.028629183	0.531497604	1.990748386
Myeloproliferative neoplasms	X-14189	Inverse variance weighted	3	0.050971636	0.627842091	0.935294479	-1.179598863	1.281542135	1.052293045	0.307402024	3.602190508
Myeloproliferative neoplasms	X-02249	Inverse variance weighted	3	0.109417536	1.352209385	0.935507484	-2.540912859	2.759747931	1.115628068	0.078794439	15.7958608
Myeloproliferative neoplasms	urate	Inverse variance weighted	2	0.115382631	1.571285751	0.941462366	-2.964337441	3.195102703	1.122302783	0.051594642	24.41268081
Myeloproliferative neoplasms	3-carboxy-4-methyl-5-propyl-2-furanpropanoate (CMP	Inverse variance weighted	5	-0.040245175	0.554004665	0.942089326	-1.126094318	1.045603969	0.960553907	0.324297388	2.845116366
Myeloproliferative neoplasms	histidine	Inverse variance weighted	2	-0.388751468	5.549728069	0.944154865	-11.26621848	10.48871555	0.67790273	1.28E-05	35908.00562
Myeloproliferative neoplasms	X-11529	Inverse variance weighted	14	-0.005760825	0.084857273	0.945874467	-0.172081081	0.160559431	0.994255737	0.841910907	1.174167553
Myeloproliferative neoplasms	stearate (18:0)	Inverse variance weighted	2	0.182126017	3.160696083	0.954049628	-6.012838305	6.37709034	1.199765376	0.002447133	588.2137132
Myeloproliferative neoplasms	X-14304	Inverse variance weighted	3	0.029743356	0.68455921	0.965343685	-1.311992697	1.371479408	1.030190107	0.269282922	3.941176994
Myeloproliferative neoplasms	gamma-glutamylphenylalanine	Inverse variance weighted	2	-0.23703589	8.288942229	0.977186293	-16.48336266	16.00929088	0.788962972	6.94E-08	8969055.019
Myeloproliferative neoplasms	gamma-glutamylglutamine	Inverse variance weighted	3	-0.067664051	2.375050009	0.977271721	-4.722762069	4.587433967	0.93457439	0.008890588	98.24201419
Myeloproliferative neoplasms	X-11905	Inverse variance weighted	11	-0.007786108	0.295905518	0.979007832	-0.587760923	0.572188708	0.992244126	0.555569857	1.77214151
Myeloproliferative neoplasms	X-11317	Inverse variance weighted	3	0.028032439	2.50490066	0.99107103	-4.881572854	4.937637733	1.028429046	0.007585074	139.4404646
Myeloproliferative neoplasms	tryptophan	MR Egger	18	-3.79283796	42.13102077	0.929384818	-86.36963868	78.78396276	0.022531567	3.09E-38	1.64E+34
Myeloproliferative neoplasms	leucine	MR Egger	12	25.89533625	17.96702415	0.180079706	-9.320031086	61.11070358	1.76279E+11	8.96E-05	3.47E+26
Myeloproliferative neoplasms	cholesterol	MR Egger	3	-13.79424439	7.899173182	0.331081047	-29.27662383	1.688135047	1.02E-06	1.93E-13	5.409383049
Myeloproliferative neoplasms	uridine	MR Egger	3	-7.4756909	31.26645327	0.850591546	-68.75793931	53.8065575	0.000566694	1.38E-30	2.33E+23
Myeloproliferative neoplasms	arachidonate (20:4n6)	MR Egger	11	1.497640622	2.169947204	0.507490387	-2.755455897	5.750737141	4.471127537	0.063580027	314.4223486
Myeloproliferative neoplasms	tyrosine	MR Egger	5	-55.70601117	18.61888272	0.058042107	-92.1990213	-19.21300103	6.41E-25	9.09E-41	4.53E-09
Myeloproliferative neoplasms	citrate	MR Egger	6	-4.643554394	7.863894781	0.586609785	-20.05678816	10.76967938	0.009623431	1.95E-09	47556.76722
Myeloproliferative neoplasms	citrulline	MR Egger	4	1.076075385	50.65752165	0.984981216	-98.21266705	100.3648178	2.933145464	2.22E-43	3.87E+43
Myeloproliferative neoplasms	gamma-glutamylglutamine	MR Egger	3	2.756809002	9.88443858	0.826844482	-16.61669061	22.13030862	15.74950604	6.07E-08	4083860659
Myeloproliferative neoplasms	gamma-glutamyltyrosine	MR Egger	5	-51.25100695	25.62486297	0.139319219	-101.4757384	-1.026275534	5.52E-23	8.50E-45	0.3583391
Myeloproliferative neoplasms	betaine	MR Egger	3	18.0441359	10.01987599	0.322703719	-1.594821036	37.68309284	68622834.65	0.202944845	2.32038E+16
Myeloproliferative neoplasms	X-03094	MR Egger	6	1.183670316	6.210488924	0.858127456	-10.98888797	13.35622861	3.266340733	1.69E-05	631737.101
Myeloproliferative neoplasms	kynurenine	MR Egger	6	1.198022587	2.599126374	0.668794445	-3.896265106	6.292310281	3.313558168	0.020317654	540.4003646
Myeloproliferative neoplasms	carnitine	MR Egger	26	-2.243500359	1.8373469	0.233925842	-5.844700282	1.357699565	0.106086513	0.002895202	3.887240665
Myeloproliferative neoplasms	N-acetylornithine	MR Egger	4	-0.324785056	0.593358825	0.639046319	-1.487768353	0.838198242	0.722682673	0.225876169	2.312197201
Myeloproliferative neoplasms	3-methyl-2-oxovalerate	MR Egger	4	13.37212783	19.64467948	0.566297101	-25.13144396	51.87569962	641861.5017	1.22E-11	3.38E+22
Myeloproliferative neoplasms	X-18601	MR Egger	11	4.9737353	1.964386019	0.032132181	1.123538703	8.823931897	144.565877	3.075719025	6794.929132
Myeloproliferative neoplasms	eicosapentaenoate (EPA; 20:5n3)	MR Egger	9	13.95684243	13.08460844	0.321525785	-11.68899012	39.60267498	1151806.84	8.39E-06	1.58206E+17
Myeloproliferative neoplasms	myo-inositol	MR Egger	3	-1.317742638	7.256767078	0.885643491	-15.54100611	12.90552084	0.267739004	1.78E-07	402528.361
Myeloproliferative neoplasms	1,5-anhydroglucitol (1,5-AG)	MR Egger	5	-2.879150921	2.854220393	0.387406234	-8.473422892	2.71512105	0.056182446	0.000208948	15.1064386
Myeloproliferative neoplasms	X-08402	MR Egger	3	0.50207451	1.803965308	0.827191135	-3.033697494	4.037846514	1.65214511	0.048137321	56.70409974
Myeloproliferative neoplasms	2-hydroxyisobutyrate	MR Egger	3	-0.263202168	10.12277584	0.983450986	-20.10384281	19.57743848	0.768586498	1.86E-09	317960741.2
Myeloproliferative neoplasms	4-methyl-2-oxopentanoate	MR Egger	3	159.7965068	164.4735368	0.509181494	-162.5716254	482.164639	2.50E+69	2.49E-71	2.52E+209
Myeloproliferative neoplasms	palmitoylcarnitine	MR Egger	6	-27.37388524	99.17344085	0.796206174	-221.7538293	167.0060588	1.29E-12	4.94E-97	3.39E+72
Myeloproliferative neoplasms	X-10510	MR Egger	4	-0.75909351	3.913603452	0.864119638	-8.429756276	6.911569257	0.468090554	0.000218275	1003.82126
Myeloproliferative neoplasms	N-acetylglycine	MR Egger	3	-1.294411725	1.657331229	0.577882109	-4.542780934	1.953957485	0.274059039	0.010643766	7.056558616
Myeloproliferative neoplasms	androsterone sulfate	MR Egger	9	0.864148384	0.227074141	0.006666172	0.419083068	1.3092137	2.372984353	1.52056666	3.703260692
Myeloproliferative neoplasms	3-carboxy-4-methyl-5-propyl-2-furanpropanoate (CMP	MR Egger	5	7.405627158	12.73306482	0.601651019	-17.55117988	32.3624342	1645.216323	2.39E-08	1.13456E+14
Myeloproliferative neoplasms	acetylcarnitine	MR Egger	12	-0.492094436	3.98953266	0.904276727	-8.31157845	7.327389578	0.61134463	0.000245656	1521.405074
Myeloproliferative neoplasms	serine	MR Egger	3	-17.6205232	20.46535212	0.547464246	-57.73261335	22.49156695	2.23E-08	8.45E-26	5860887911
Myeloproliferative neoplasms	hexanoylcarnitine	MR Egger	17	-0.16854532	0.87093128	0.849145499	-1.875570629	1.538479989	0.844892972	0.153267483	4.657505429
Myeloproliferative neoplasms	butyrylcarnitine	MR Egger	12	1.169813033	0.575817131	0.069621653	0.041211456	2.29841461	3.221390289	1.042072435	9.958382015
Myeloproliferative neoplasms	dehydroisoandrosterone sulfate (DHEA-S)	MR Egger	7	7.134711619	1.952627799	0.014688203	3.307561133	10.96186211	1254.775083	27.31841817	57633.66312
Myeloproliferative neoplasms	propionylcarnitine	MR Egger	14	-0.75528749	2.647668918	0.780304921	-5.944718569	4.434143589	0.469875511	0.002619639	84.27991573
Myeloproliferative neoplasms	10-undecenoate (11:1n1)	MR Egger	3	-3.489607944	2.06948767	0.340775165	-7.545803778	0.56658789	0.030512833	0.000528322	1.762243811
Myeloproliferative neoplasms	docosapentaenoate (n3 DPA; 22:5n3)	MR Egger	8	-14.89484454	31.63303763	0.654369272	-76.8955983	47.10590921	3.40E-07	4.02E-34	2.87E+20
Myeloproliferative neoplasms	X-11204	MR Egger	3	3.737681205	24.30693326	0.902867676	-43.90390799	51.3792704	42.00048666	8.57E-20	2.06E+22
Myeloproliferative neoplasms	X-02269	MR Egger	13	-0.060508968	2.83365825	0.983345965	-5.614479138	5.493461202	0.941285327	0.003644708	243.0971607
Myeloproliferative neoplasms	X-11247	MR Egger	3	1.97442433	2.287590936	0.546693969	-2.509253905	6.458102565	7.202472213	0.081328896	637.8496296
Myeloproliferative neoplasms	X-11261	MR Egger	5	-1.283273391	4.036536153	0.771394086	-9.194884251	6.628337469	0.277128664	0.000101558	756.2238795
Myeloproliferative neoplasms	X-02249	MR Egger	3	9.188538698	11.39243426	0.567913076	-13.14063246	31.51770985	9784.342648	1.96E-06	4.87492E+13
Myeloproliferative neoplasms	X-11315	MR Egger	4	11.98060852	8.687980721	0.30186656	-5.047833693	29.00905073	159629.1387	0.006423233	3.96708E+12
Myeloproliferative neoplasms	X-11317	MR Egger	3	13.93924203	10.09405022	0.399000409	-5.845096396	33.72358045	1131711.929	0.002894056	4.42553E+14
Myeloproliferative neoplasms	1-linoleoylglycerophosphoethanolamine*	MR Egger	12	-1.093219646	2.710427863	0.695188329	-6.405658257	4.219218965	0.335135736	0.001652182	67.98036847
Myeloproliferative neoplasms	3-dehydrocarnitine*	MR Egger	13	-3.519579271	4.32437844	0.432972001	-11.99536101	4.956202471	0.029611891	6.17E-06	142.0533187
Myeloproliferative neoplasms	pyroglutamine*	MR Egger	5	-3.732293561	3.286887773	0.338672295	-10.17459359	2.710006474	0.02393787	3.81E-05	15.02937281
Myeloproliferative neoplasms	X-11381	MR Egger	4	29.31215332	104.823192	0.806024373	-176.141303	234.7656097	5.37163E+12	3.18E-77	9.07E+101
Myeloproliferative neoplasms	X-03056	MR Egger	3	2.454740019	13.04110071	0.881554298	-23.10581737	28.01529741	11.64340609	9.23E-11	1.46855E+12
Myeloproliferative neoplasms	X-11440	MR Egger	6	-0.590552382	0.51779188	0.317720533	-1.605424467	0.424319703	0.554021169	0.200804302	1.528550198
Myeloproliferative neoplasms	X-11445	MR Egger	9	4.008736314	5.791081819	0.511097491	-7.34178405	15.35925668	55.07722625	0.000647894	4682097.158
Myeloproliferative neoplasms	X-11469	MR Egger	10	2.878040412	5.585763008	0.620312775	-8.070055084	13.82613591	17.77939872	0.000312766	1010682
Myeloproliferative neoplasms	X-11491	MR Egger	9	2.705437423	1.507496032	0.115786312	-0.249254799	5.660129645	14.96085948	0.779381362	287.1858723
Myeloproliferative neoplasms	X-11529	MR Egger	14	0.415253517	0.190935277	0.050356947	0.041020374	0.789486661	1.514754709	1.041873332	2.202265627
Myeloproliferative neoplasms	X-11538	MR Egger	15	0.858736134	0.646444236	0.206895552	-0.408294568	2.125766836	2.360175862	0.664783026	8.379320594
Myeloproliferative neoplasms	X-11593	MR Egger	6	0.526845609	1.71292181	0.773757704	-2.830481138	3.884172357	1.693581656	0.058984467	48.62668026
Myeloproliferative neoplasms	adrenate (22:4n6)	MR Egger	9	-13.00299395	14.26205161	0.392231434	-40.9566151	14.9506272	2.25E-06	1.63E-18	3111536.467
Myeloproliferative neoplasms	X-11787	MR Egger	6	-0.686947475	5.372349399	0.904424838	-11.2167523	9.842857348	0.503109482	1.34E-05	18823.42432
Myeloproliferative neoplasms	X-11792	MR Egger	4	2.076297305	1.540257656	0.310036396	-0.942607701	5.09520231	7.974885607	0.389610522	163.2368657
Myeloproliferative neoplasms	X-11793	MR Egger	3	1.258028606	1.118682276	0.462717724	-0.934588655	3.450645867	3.518478338	0.392747387	31.52074393
Myeloproliferative neoplasms	1-arachidonoylglycerophosphocholine*	MR Egger	13	-0.13907786	1.101114694	0.901768465	-2.297262661	2.019106941	0.870160275	0.100533662	7.531595769
Myeloproliferative neoplasms	X-11905	MR Egger	11	2.154354283	1.173298994	0.099518489	-0.145311746	4.454020311	8.622320799	0.864752668	85.97188388
Myeloproliferative neoplasms	X-12038	MR Egger	4	18.25829096	16.09703665	0.374332302	-13.29190087	49.80848278	85010977.7	1.69E-06	4.28E+21
Myeloproliferative neoplasms	X-12063	MR Egger	20	0.639460918	0.374194329	0.104654539	-0.093959967	1.372881803	1.895458796	0.910319205	3.946707957
Myeloproliferative neoplasms	isobutyrylcarnitine	MR Egger	5	-0.177763381	2.749343969	0.952514657	-5.566477561	5.210950798	0.837140483	0.003823926	183.2682264
Myeloproliferative neoplasms	erythronate*	MR Egger	3	-32.93963053	57.14253835	0.667098234	-144.9390057	79.05974464	4.95E-15	1.13E-63	2.16E+34
Myeloproliferative neoplasms	X-12092	MR Egger	6	-0.425169936	0.219539176	0.124857501	-0.855466722	0.005126849	0.653658695	0.425084748	1.005140014
Myeloproliferative neoplasms	X-12093	MR Egger	5	0.134029678	2.373802226	0.958523849	-4.518622685	4.78668204	1.143426754	0.010904032	119.902875
Myeloproliferative neoplasms	X-12100	MR Egger	4	4.472472823	14.13845641	0.78171237	-23.23890173	32.18384738	87.57300801	8.08E-11	9.49002E+13
Myeloproliferative neoplasms	X-12244	MR Egger	3	9.198527999	17.19966717	0.687352783	-24.51281965	42.90987565	9882.571189	2.26E-11	4.32038E+18
Myeloproliferative neoplasms	1-eicosatrienoylglycerophosphocholine*	MR Egger	11	3.801264736	4.036302686	0.370900953	-4.109888528	11.712418	44.75775544	0.016409604	122078.3095
Myeloproliferative neoplasms	1-eicosadienoylglycerophosphocholine*	MR Egger	3	-4.417808219	82.13699715	0.965791826	-165.4063226	156.5707062	0.012060638	1.46E-72	9.95E+67
Myeloproliferative neoplasms	X-12442	MR Egger	3	0.521026086	27.09608846	0.987760055	-52.5873073	53.62935947	1.683754441	1.45E-23	1.95E+23
Myeloproliferative neoplasms	X-12456	MR Egger	12	2.576145911	1.212443409	0.059544915	0.199756829	4.952534994	13.14637311	1.221105784	141.5332955
Myeloproliferative neoplasms	octanoylcarnitine	MR Egger	7	-0.66966629	1.293537712	0.626748772	-3.205000206	1.865667626	0.511879369	0.040558893	6.460247477
Myeloproliferative neoplasms	alpha-hydroxyisovalerate	MR Egger	3	3.712275073	11.04539397	0.793587608	-17.93669711	25.36124725	40.94685774	1.62E-08	1.03336E+11
Myeloproliferative neoplasms	decanoylcarnitine	MR Egger	7	-0.149321115	1.99179453	0.943147082	-4.053238395	3.754596164	0.861292496	0.017366045	42.71696567
Myeloproliferative neoplasms	stearidonate (18:4n3)	MR Egger	9	8.853006328	7.887365236	0.298699027	-6.606229535	24.31224219	6995.387822	0.00135192	36197004053
Myeloproliferative neoplasms	epiandrosterone sulfate	MR Egger	5	1.02538324	0.357258834	0.06404293	0.325155925	1.725610555	2.788163791	1.384246467	5.615948826
Myeloproliferative neoplasms	X-12510	MR Egger	9	0.185938524	0.563713149	0.751167452	-0.918939248	1.290816297	1.20434822	0.398941995	3.635753199
Myeloproliferative neoplasms	1-arachidonoylglycerophosphoinositol*	MR Egger	17	4.926206349	2.357685755	0.054120655	0.305142269	9.547270429	137.8555433	1.356818022	14006.41095
Myeloproliferative neoplasms	X-12644	MR Egger	7	4.621405964	8.624533137	0.615035136	-12.28267899	21.52549091	101.6368294	4.63E-06	2230498439
Myeloproliferative neoplasms	X-12696	MR Egger	3	-5.530980807	11.59295612	0.716602947	-28.25317479	17.19121318	0.003962101	5.37E-13	29244824.57
Myeloproliferative neoplasms	X-12728	MR Egger	3	-0.192120199	0.57147862	0.793537093	-1.312218294	0.927977896	0.825207673	0.26922218	2.529389314
Myeloproliferative neoplasms	isovalerylcarnitine	MR Egger	11	-4.072545847	5.042302016	0.440101162	-13.9554578	5.810366104	0.017033967	8.69E-07	333.7412874
Myeloproliferative neoplasms	stearoylcarnitine	MR Egger	6	-32.79154166	43.03217837	0.488501539	-117.1346113	51.55152795	5.74E-15	1.35E-51	2.45E+22
Myeloproliferative neoplasms	1-stearoylglycerophosphoethanolamine	MR Egger	3	-7.130523115	80.86294633	0.944007473	-165.6218979	151.3608517	0.000800301	1.18E-72	5.43E+65

Myeloproliferative neoplasms	bradykinin, des-arg(9)	MR Egger	6	1.301052024	0.628944779	0.10739711	0.068320257	2.533783792	3.673158888	1.070708155	12.60109597
Myeloproliferative neoplasms	X-12798	MR Egger	10	-1.204847104	0.5075912	0.044988924	-2.199725856	-0.209968352	0.299737825	0.110833539	0.8106099
Myeloproliferative neoplasms	X-12844	MR Egger	3	2.264770895	16.58860835	0.913619108	-30.24890147	34.77844326	9.628918315	7.30E-14	1.27083E+15
Myeloproliferative neoplasms	X-12850	MR Egger	6	1.041653322	1.373970466	0.490583832	-1.651328792	3.734635436	2.83389849	0.191794884	41.87275747
Myeloproliferative neoplasms	laurylcarnitine	MR Egger	3	-4.655402971	29.19183547	0.899322088	-61.87140049	52.56059454	0.00951008	1.35E-27	6.71E+22
Myeloproliferative neoplasms	X-12855	MR Egger	6	-31.91819325	37.30346626	0.440425603	-105.0329871	41.19660062	1.37E-14	2.43E-46	7.78854E+17
Myeloproliferative neoplasms	X-13215	MR Egger	3	-4.708333333	13.00458787	0.778856937	-30.19732555	20.78065888	0.009019798	7.68E-14	1059071993
Myeloproliferative neoplasms	oleoylcarnitine	MR Egger	6	-14.00186765	18.93196513	0.500603104	-51.10851931	23.10478402	8.30E-07	6.37E-23	10821319127
Myeloproliferative neoplasms	1-arachidonoylglycerophosphoethanolamine*	MR Egger	18	-1.604244545	2.581008746	0.542988413	-6.663021688	3.454532598	0.201041375	0.001277281	31.643495
Myeloproliferative neoplasms	X-13429	MR Egger	16	1.178472306	0.479368306	0.027594847	0.238910427	2.118034185	3.24940631	1.269864786	8.314776096
Myeloproliferative neoplasms	X-13431	MR Egger	4	1.702629318	1.027381598	0.239319231	-0.311038615	3.71629725	5.488359077	0.732685583	41.11188491
Myeloproliferative neoplasms	X-13435	MR Egger	3	-2.73787089	4.39383581	0.645247689	-11.34978908	5.874047298	0.064707971	1.18E-05	355.6856367
Myeloproliferative neoplasms	X-13477	MR Egger	3	6.11344965	21.5729706	0.824200912	-36.16957272	48.39647202	451.8949089	1.96E-16	1.04E+21
Myeloproliferative neoplasms	glutaroyl carnitine	MR Egger	11	-0.266041187	2.867540669	0.928113314	-5.886420899	5.354338525	0.766407561	0.002776898	211.5240121
Myeloproliferative neoplasms	2-tetradecenoyl carnitine	MR Egger	4	3.555555556	41.24318125	0.939153601	-77.2810797	84.39219081	35.00726305	2.74E-34	4.48E+36
Myeloproliferative neoplasms	1-palmitoylglycerophosphoethanolamine	MR Egger	6	2.692176682	4.778503267	0.603251192	-6.673689721	12.05804309	14.76377702	0.001263727	172481.1244
Myeloproliferative neoplasms	tetradecanedioate	MR Egger	16	1.445545145	0.688531129	0.05438722	0.096024131	2.795066159	4.244165198	1.100785627	16.36371132
Myeloproliferative neoplasms	hexadecanedioate	MR Egger	15	1.868821951	0.896828864	0.057475128	0.111037377	3.626606525	6.480657369	1.117436672	37.58505603
Myeloproliferative neoplasms	dihomo-linolenate (20:3n3 or n6)	MR Egger	10	5.671948319	6.156723322	0.383854682	-6.395229393	17.73912603	290.6001651	0.001669503	50582996.02
Myeloproliferative neoplasms	X-14189	MR Egger	3	9.070481432	6.427302605	0.392457954	-3.527031673	21.66799454	8694.808754	0.029392032	2572115468
Myeloproliferative neoplasms	X-14205	MR Egger	3	0.876785142	2.252417857	0.763675877	-3.537953858	5.291524142	2.403161451	0.029072753	198.6459594
Myeloproliferative neoplasms	X-14208	MR Egger	3	3.834786611	3.577098951	0.477875669	-3.176327332	10.84590055	46.28355022	0.041738666	51323.32234
Myeloproliferative neoplasms	X-14304	MR Egger	3	13.47395653	9.61014686	0.394421492	-5.361931315	32.30984438	710665.0711	0.004691836	1.07643E+14
Myeloproliferative neoplasms	X-14626	MR Egger	10	2.442942889	1.629953989	0.172312792	-0.751766929	5.637652706	11.50685436	0.471532652	280.8028176
Myeloproliferative neoplasms	X-14662	MR Egger	6	1.643685886	19.28109292	0.936160178	-36.14725625	39.43462802	5.174205943	2.00E-16	1.33734E+17
Myeloproliferative neoplasms	octadecanedioate	MR Egger	10	-4.255085091	4.835500151	0.404534524	-13.73266539	5.222495205	0.014191883	1.09E-06	185.3962089
Myeloproliferative neoplasms	succinylcarnitine	MR Egger	9	-1.809116028	2.488116592	0.490759042	-6.685824549	3.067592492	0.163798866	0.001248485	21.49010276
Myeloproliferative neoplasms	5alpha-androstan-3beta,17beta-diol disulfate	MR Egger	10	1.277672583	0.665872395	0.091285029	-0.027437312	2.582782477	3.588278577	0.972935672	13.23391003
Myeloproliferative neoplasms	4-androsten-3beta,17beta-diol disulfate 1*	MR Egger	5	-1.847864871	0.88176576	0.12709167	-3.576125761	-0.119603981	0.157573247	0.027983905	0.887271744
Myeloproliferative neoplasms	4-androsten-3beta,17beta-diol disulfate 2*	MR Egger	11	1.291225205	2.080105363	0.550158835	-2.785781307	5.368231716	3.637240191	0.061680879	214.4832649
Myeloproliferative neoplasms	cis-4-decenoyl carnitine	MR Egger	6	-1.354175917	1.633045685	0.45358935	-4.55494546	1.846593625	0.258159952	0.010515074	6.338192447
Myeloproliferative neoplasms	tryptophan	Simple mode	18	0.02773935	7.288103028	0.997007475	-14.25694258	14.31242129	1.028127668	6.43E-07	1643635.78
Myeloproliferative neoplasms	leucine	Simple mode	12	-6.17126483	7.460914831	0.425734095	-20.7946579	8.45212824	0.002088593	9.31E-10	4685.033006
Myeloproliferative neoplasms	cholesterol	Simple mode	3	3.920518344	6.468357472	0.606072069	-8.757462302	16.59849899	50.42657633	0.000157283	16167263.6
Myeloproliferative neoplasms	uridine	Simple mode	3	0.007988946	5.750334567	0.999017616	-11.2626668	11.2786447	1.008020943	1.28E-05	79113.96584
Myeloproliferative neoplasms	arachidonate (20:4n6)	Simple mode	11	-0.634916231	0.733256335	0.406847284	-2.072098647	0.802266185	0.529979882	0.12592124	2.230590135
Myeloproliferative neoplasms	tyrosine	Simple mode	5	7.398172487	7.805151301	0.396874434	-7.899924063	22.69626904	1632.997377	0.000370772	7192243836
Myeloproliferative neoplasms	citrate	Simple mode	6	-0.02661367	4.383553314	0.995390656	-8.618378166	8.565150825	0.973737352	0.000180753	5245.631036
Myeloproliferative neoplasms	citrulline	Simple mode	4	5.704740517	4.948947492	0.332540673	-3.995196567	15.4046776	300.2875504	0.018403828	4899666.017
Myeloproliferative neoplasms	gamma-glutamylglutamine	Simple mode	3	1.168709619	3.849628167	0.790111011	-6.376561588	8.713980827	3.217837722	0.001700962	6087.42731
Myeloproliferative neoplasms	gamma-glutamyltyrosine	Simple mode	5	8.192231125	5.772286327	0.228837	-3.121450076	19.50591233	3612.77381	0.044093184	296012525.1
Myeloproliferative neoplasms	betaine	Simple mode	3	2.855173883	2.632475518	0.39143907	-2.304478133	8.014825899	17.37745859	0.099810875	3025.482613
Myeloproliferative neoplasms	X-03094	Simple mode	6	0.226352231	2.857007853	0.939925181	-5.373383162	5.826087624	1.254017291	0.004638412	339.0296693
Myeloproliferative neoplasms	kynurenine	Simple mode	6	0.898381094	1.901106965	0.656436605	-2.827788559	4.624550746	2.455624465	0.059143502	101.9569582
Myeloproliferative neoplasms	carnitine	Simple mode	26	-3.620383003	2.450162825	0.152000824	-8.422702141	1.181936134	0.026772421	0.00021982	3.260681212
Myeloproliferative neoplasms	N-acetylornithine	Simple mode	4	0.072664172	0.240079648	0.781916311	-0.397891938	0.543220282	1.075369337	0.671734613	1.721541795
Myeloproliferative neoplasms	3-methyl-2-oxovalerate	Simple mode	4	0.744506613	3.877196461	0.859987677	-6.85479845	8.343811676	2.1054024	0.001054384	4204.083846
Myeloproliferative neoplasms	X-18601	Simple mode	11	0.452907819	0.960976837	0.647540654	-1.430606782	2.33642242	1.57287919	0.239163758	10.34416322
Myeloproliferative neoplasms	eicosapentaenoate (EPA; 20:5n3)	Simple mode	9	-0.89027414	1.04116829	0.417381951	-2.930963989	1.150415709	0.410543191	0.053345589	3.159506071
Myeloproliferative neoplasms	myo-inositol	Simple mode	3	0.623603207	3.577075294	0.877653852	-6.387464369	7.634670782	1.865638225	0.001682517	2068.689889
Myeloproliferative neoplasms	1,5-anhydroglucitol (1,5-AG)	Simple mode	5	-1.172608576	1.465677589	0.468503654	-4.045336651	1.700119499	0.309558381	0.017503811	5.47460156
Myeloproliferative neoplasms	X-08402	Simple mode	3	-0.583240299	1.115382345	0.65319697	-2.769389696	1.602909098	0.558087065	0.062700259	4.967462259
Myeloproliferative neoplasms	2-hydroxyisobutyrate	Simple mode	3	0.6727997	2.906819224	0.838485012	-5.024565978	6.370165379	1.959716265	0.006574439	584.1544275
Myeloproliferative neoplasms	4-methyl-2-oxopentanoate	Simple mode	3	4.160666016	3.790454919	0.386851351	-3.268625625	11.58995766	64.11420948	0.038058698	108007.6846
Myeloproliferative neoplasms	palmitoylcarnitine	Simple mode	6	-2.837376827	1.881786576	0.191968096	-6.525678516	0.850924862	0.058579128	0.001465325	2.341811704
Myeloproliferative neoplasms	X-10510	Simple mode	4	-1.210217859	1.66861628	0.520733757	-4.480705768	2.060270051	0.298132322	0.011325417	7.848088907
Myeloproliferative neoplasms	N-acetylglycine	Simple mode	3	-1.149070357	0.93696951	0.344850677	-2.985530596	0.687389882	0.316931265	0.050512695	1.988518486
Myeloproliferative neoplasms	androsterone sulfate	Simple mode	9	-1.411964446	0.393314276	0.007085374	-2.182860427	-0.641068464	0.243664148	0.112718646	0.526729332
Myeloproliferative neoplasms	3-carboxy-4-methyl-5-propyl-2-furanpropanoate (CMP	Simple mode	5	0.046174062	0.831683782	0.958387639	-1.583926152	1.676274275	1.047256682	0.205167994	5.345602579
Myeloproliferative neoplasms	acetylcarnitine	Simple mode	12	-3.418496969	1.880632998	0.096415678	-7.104537645	0.267543708	0.03276164	0.000821369	1.306750744
Myeloproliferative neoplasms	serine	Simple mode	3	-0.623722161	2.708657144	0.839291036	-5.932690164	4.685245842	0.535945844	0.00265134	108.3369028
Myeloproliferative neoplasms	hexanoylcarnitine	Simple mode	17	-0.355569874	0.894635202	0.696290034	-2.109054871	1.397915122	0.700773976	0.121352606	4.046754177
Myeloproliferative neoplasms	butyrylcarnitine	Simple mode	12	-2.183940961	1.07861331	0.06785833	-4.298023048	-0.069858873	0.112596915	0.01359541	0.932525415
Myeloproliferative neoplasms	dehydroisoandrosterone sulfate (DHEA-S)	Simple mode	7	-2.376109873	2.275643714	0.336647876	-6.836371553	2.084151806	0.092911312	0.001073993	8.037771
Myeloproliferative neoplasms	propionylcarnitine	Simple mode	14	-3.412036216	1.834760984	0.085707712	-7.008167746	0.184095313	0.03297399	0.000904464	1.202130396
Myeloproliferative neoplasms	10-undecenoate (11:1n1)	Simple mode	3	0.450234401	0.987623721	0.693193185	-1.485508093	2.385976895	1.568679842	0.226387286	10.86967601
Myeloproliferative neoplasms	docosapentaenoate (n3 DPA; 22:5n3)	Simple mode	8	-1.328269703	1.49018733	0.402341793	-4.24903687	1.592497463	0.264935282	0.014277979	4.916011168
Myeloproliferative neoplasms	X-11204	Simple mode	3	3.565241434	4.338430533	0.497578689	-4.93808241	12.06856528	35.34798659	0.007168331	174305.5859
Myeloproliferative neoplasms	X-02269	Simple mode	13	-0.32256397	0.617329591	0.610822835	-1.532529968	0.887402027	0.724289597	0.215988531	2.428811462
Myeloproliferative neoplasms	X-11247	Simple mode	3	-0.419733167	1.130454403	0.746060338	-2.635423798	1.795957463	0.657222165	0.071688581	6.025240908
Myeloproliferative neoplasms	X-11261	Simple mode	5	-0.702246515	1.194614215	0.588221924	-3.043690378	1.639197347	0.495470969	0.047658686	5.151033359
Myeloproliferative neoplasms	X-02249	Simple mode	3	-0.461324091	1.844948361	0.825890504	-4.07742288	3.154774697	0.630448321	0.016951094	23.44775365
Myeloproliferative neoplasms	X-11315	Simple mode	4	4.101866132	2.61762318	0.215098702	-1.0286753	9.232407563	60.45299565	0.357480201	10223.12473
Myeloproliferative neoplasms	X-11317	Simple mode	3	1.587071313	3.676449739	0.708050524	-5.618770175	8.792912801	4.889408394	0.003629102	6587.392021
Myeloproliferative neoplasms	1-linoleoylglycerophosphoethanolamine*	Simple mode	12	0.7674148	0.790756145	0.352662107	-0.782467244	2.317296845	2.154190038	0.457276406	10.14820502
Myeloproliferative neoplasms	3-dehydrocarnitine*	Simple mode	13	-2.224517358	1.41363713	0.141559229	-4.995246132	0.546211417	0.108119589	0.006770055	1.726698868
Myeloproliferative neoplasms	pyroglutamine*	Simple mode	5	-2.553502867	1.564264482	0.177932239	-5.619461253	0.512455518	0.077808635	0.003626594	1.669385372
Myeloproliferative neoplasms	X-11381	Simple mode	4	-1.244862663	2.578720828	0.66231333	-6.299155485	3.809430159	0.287980456	0.001837856	45.12471764
Myeloproliferative neoplasms	X-03056	Simple mode	3	1.108686046	2.263063377	0.672668963	-3.326918173	5.544290265	3.030374006	0.035903583	255.7729828
Myeloproliferative neoplasms	X-11440	Simple mode	6	-0.741583325	0.332426746	0.076086879	-1.393139747	-0.090026903	0.476359087	0.248294498	0.913906598
Myeloproliferative neoplasms	X-11445	Simple mode	9	0.275986701	0.597266969	0.65632864	-0.894656558	1.44662996	1.317830338	0.408747956	4.248771831
Myeloproliferative neoplasms	X-11469	Simple mode	10	0.137276239	0.78515423	0.865075527	-1.401626052	1.67617853	1.147144991	0.24619631	5.345090788
Myeloproliferative neoplasms	X-11491	Simple mode	9	0.208427318	0.399389737	0.615901437	-0.574376567	0.991231203	1.231739402	0.563055787	2.694549969
Myeloproliferative neoplasms	X-11529	Simple mode	14	-0.127824233	0.123104171	0.318039049	-0.369108409	0.113459943	0.880008042	0.691350458	1.120147018
Myeloproliferative neoplasms	X-11538	Simple mode	15	-0.206375107	0.348599557	0.563281783	-0.889630239	0.476880025	0.813527859	0.410807625	1.611040148
Myeloproliferative neoplasms	X-11593	Simple mode	6	0.891566333	2.36951571	0.722157197	-3.752684459	5.535817126	2.438946865	0.023454698	253.6149383
Myeloproliferative neoplasms	adrenate (22:4n6)	Simple mode	9	-0.930198771	1.058564626	0.40516359	-3.004985438	1.144587896	0.394475292	0.049539476	3.14114661
Myeloproliferative neoplasms	X-11787	Simple mode	6	0.500311195	1.435892392	0.741706854	-2.314037893	3.314660283	1.649234425	0.098861253	27.51304575
Myeloproliferative neoplasms	X-11792	Simple mode	4	0.140714759	0.432663943	0.766369695	-0.70730657	0.988736087	1.151096261	0.492970192	2.687835135
Myeloproliferative neoplasms	X-11793	Simple mode	3	0.382003579	0.543423323	0.554889415	-0.683106133	1.447113292	1.46521733	0.505045814	4.250825894
Myeloproliferative neoplasms	1-arachidonoylglycerophosphocholine*	Simple mode	13	-0.547108616	0.544988906	0.335246071	-1.615286872	0.52106964	0.578620408	0.198833622	1.683827776
Myeloproliferative neoplasms	X-11905	Simple mode	11	0.231342216	0.443495434	0.613281939	-0.637908834	1.100593266	1.260290457	0.528396234	3.005948821
Myeloproliferative neoplasms	X-12038	Simple mode	4	1.365282226	3.924366003	0.750895791	-6.32647514	9.057039593	3.916828328	0.001788326	8578.716532
Myeloproliferative neoplasms	X-12063	Simple mode	20	-0.369312595	0.318880342	0.261152366	-0.994318065	0.255692876	0.691209308	0.369975658	1.291356061
Myeloproliferative neoplasms	isobutyrylcarnitine	Simple mode	5	-1.434640451	1.501569466	0.393466034	-4.377716604	1.508435703	0.238200994	0.012553992	4.519655177
Myeloproliferative neoplasms	erythronate*	Simple mode	3	6.082232533	4.875661248	0.338487871	-3.474063512	15.63852858	438.0059667	0.030990843	6190513.378
Myeloproliferative neoplasms	X-12092	Simple mode	6	0.064243165	0.377894591	0.871672448	-0.676430233	0.804916563	1.066351667	0.508428729	2.236509883
Myeloproliferative neoplasms	X-12093	Simple mode	5	-0.113509247	0.51207723	0.835431897	-1.117180618	0.890162124	0.892695942	0.327201	2.435524476
Myeloproliferative neoplasms	X-12100	Simple mode	4	1.560166411	3.397106639	0.677278832	-5.098162602	8.218495423	4.75961323	0.006107959	3708.91783
Myeloproliferative neoplasms	X-12244	Simple mode	3	0.184438098	2.316448244	0.943788417	-4.355800461	4.724676657	1.202542539	0.012832164	112.6940536
Myeloproliferative neoplasms	1-eicosatrienoylglycerophosphocholine*	Simple mode	11	1.541153268	1.482842043	0.323130929	-1.365217136	4.447523671	4.669972895	0.25532523	85.41516588
Myeloproliferative neoplasms	1-eicosadienoylglycerophosphocholine*	Simple mode	3	2.047259267	2.412404513	0.485455058	-2.681053579	6.775572113	7.746640512	0.068490955	876.1804943
Myeloproliferative neoplasms	X-12442	Simple mode	3	2.088184182	1.089058081	0.195220081	-0.046369656	4.22273802	8.070247749	0.95468899	68.22001656
Myeloproliferative neoplasms	X-12456	Simple mode	12	0.203776897	0.499178645	0.690942593	-0.774613248	1.182167042	1.226024594	0.460881994	3.261434216
Myeloproliferative neoplasms	octanoylcarnitine	Simple mode	7	-0.419486382	0.682761893	0.561512335	-1.757699692	0.918726929	0.657384378	0.172441076	2.506097917
Myeloproliferative neoplasms	alpha-hydroxyisovalerate	Simple mode	3	2.866004172	1.818941047	0.255798515	-0.69912028	6.431128623	17.56668432	0.497022352	620.8742859
Myeloproliferative neoplasms	decanoylcarnitine	Simple mode	7	-0.508765889	0.784195519	0.540505696	-2.045789106	1.028257328	0.601237114	0.129278136	2.796188746
Myeloproliferative neoplasms	stearidonate (18:4n3)	Simple mode	9	-0.9674026	1.066436535	0.390840324	-3.057618209	1.12281301	0.380068948	0.046999505	3.073487805
Myeloproliferative neoplasms	epiandrosterone sulfate	Simple mode	5	0.616188485	0.272889974	0.086871418	0.081324137	1.151052834	1.851856196	1.084722438	3.161519713
Myeloproliferative neoplasms	X-12510	Simple mode	9	0.118424407	0.599870025	0.848425074	-1.057320843	1.294169656	1.125721774	0.347385264	3.647965651
Myeloproliferative neoplasms	1-arachidonoylglycerophosphoinositol*	Simple mode	17	-0.062879779	0.957782381	0.948468661	-1.940133247	1.814373688	0.939056361	0.143684803	6.137231155
Myeloproliferative neoplasms	X-12644	Simple mode	7	0.889296526	1.801687658	0.639140421	-2.642011283	4.420604336	2.433417204	0.071217886	83.14651858
Myeloproliferative neoplasms	X-12696	Simple mode	3	-2.007705062	1.683969602	0.355444052	-5.308285481	1.292875357	0.134296524	0.004950407	3.643247147
Myeloproliferative neoplasms	X-12728	Simple mode	3	0.067803777	0.291861047	0.837900879	-0.504243876	0.63985143	1.070155299	0.603962073	1.89619914
Myeloproliferative neoplasms	isovalerylcarnitine	Simple mode	11	-1.498377946	0.991509023	0.161671613	-3.441735632	0.444979739	0.223492383	0.032009081	1.560458579
Myeloproliferative neoplasms	stearoylcarnitine	Simple mode	6	-2.711214326	1.864876764	0.205736052	-6.366372784	0.943944132	0.066456058	0.001718381	2.57009826
Myeloproliferative neoplasms	1-stearoylglycerophosphoethanolamine	Simple mode	3	1.216506882	1.736960139	0.55620703	-2.18793499	4.620948755	3.375376529	0.112148097	101.5903708
Myeloproliferative neoplasms	bradykinin, des-arg(9)	Simple mode	6	0.050878389	0.263490293	0.854483972	-0.465562585	0.567319363	1.052194927	0.627781825	1.763533317
Myeloproliferative neoplasms	X-12798	Simple mode	10	0.451514655	0.628624874	0.490822501	-0.780590098	1.683619408	1.570689437	0.458135586	5.385011294
Myeloproliferative neoplasms	X-12844	Simple mode	3	3.915425034	2.731410813	0.288125286	-1.43814016	9.268990228	50.17039111	0.237368817	10604.03882
Myeloproliferative neoplasms	X-12850	Simple mode	6	-1.855329568	1.10264416	0.153273131	-4.016512122	0.305852985	0.156401389	0.018015692	1.357782678
Myeloproliferative neoplasms	laurylcarnitine	Simple mode	3	0.543579714	1.61974275	0.769109534	-2.631116076	3.718275503	1.722160683	0.071998062	41.19329512
Myeloproliferative neoplasms	X-12855	Simple mode	6	-1.071188761	1.902748924	0.597762782	-4.800576652	2.65819913	0.342601004	0.008225003	14.27056651
Myeloproliferative neoplasms	X-13215	Simple mode	3	1.809315292	3.673550694	0.671107351	-5.390844069	9.009474654	6.106264995	0.004558124	8180.222696
Myeloproliferative neoplasms	oleoylcarnitine	Simple mode	6	-2.605932338	1.804993287	0.208413987	-6.14371918	0.931854505	0.073834267	0.002146924	2.539213798
Myeloproliferative neoplasms	1-arachidonoylglycerophosphoethanolamine*	Simple mode	18	0.199491439	0.979860569	0.841089751	-1.721035275	2.120018154	1.220781759	0.178880861	8.33128873
Myeloproliferative neoplasms	X-13429	Simple mode	16	-0.209028088	0.261784609	0.437055955	-0.722125922	0.304069746	0.811372445	0.485718558	1.355363584
Myeloproliferative neoplasms	X-13431	Simple mode	4	-1.918853162	1.410324695	0.266848515	-4.683089563	0.845383239	0.146775193	0.00925039	2.328870158
Myeloproliferative neoplasms	X-13435	Simple mode	3	-2.246605128	1.874516438	0.353474404	-5.920657347	1.427447091	0.105757649	0.002683436	4.168044957
Myeloproliferative neoplasms	X-13477	Simple mode	3	0.753218701	2.352064483	0.779149439	-3.856827686	5.363265088	2.123824985	0.02113494	213.4206473
Myeloproliferative neoplasms	glutaroyl carnitine	Simple mode	11	0.525530031	1.760896904	0.771473195	-2.925827902	3.976887964	1.691355081	0.053620281	53.35074615
Myeloproliferative neoplasms	2-tetradecenoyl carnitine	Simple mode	4	-2.311464995	1.665679581	0.259337911	-5.576196973	0.953266983	0.099115941	0.00378694	2.594170942
Myeloproliferative neoplasms	1-palmitoylglycerophosphoethanolamine	Simple mode	6	1.475840636	1.653552656	0.41299979	-1.765122569	4.716803842	4.374711768	0.171165806	111.8103175
Myeloproliferative neoplasms	tetradecanedioate	Simple mode	16	-0.531331331	0.35108546	0.150962188	-1.219458834	0.156796171	0.587821863	0.295389979	1.169757159
Myeloproliferative neoplasms	hexadecanedioate	Simple mode	15	-0.361337398	0.440636573	0.425937944	-1.224985081	0.502310285	0.696743879	0.293762083	1.65253469
Myeloproliferative neoplasms	dihomo-linolenate (20:3n3 or n6)	Simple mode	10	1.584805923	1.686738854	0.371969695	-1.721202231	4.890814078	4.878344513	0.178850998	133.0618527
Myeloproliferative neoplasms	X-14189	Simple mode	3	0.453444969	0.89599387	0.663070761	-1.302703015	2.209592954	1.57372429	0.27179613	9.11200663
Myeloproliferative neoplasms	X-14205	Simple mode	3	0.811961471	0.990872879	0.498649699	-1.130149371	2.754072313	2.25232152	0.322985008	15.70646346
Myeloproliferative neoplasms	X-14208	Simple mode	3	0.445670858	0.851111709	0.652772532	-1.222508092	2.113849807	1.561537414	0.29449063	8.280056627
Myeloproliferative neoplasms	X-14304	Simple mode	3	0.504841552	1.056349977	0.679852016	-1.565604403	2.575287508	1.656722996	0.208961678	13.13509305
Myeloproliferative neoplasms	X-14626	Simple mode	10	0.352303033	0.750180841	0.649793449	-1.118051415	1.822657482	1.422339475	0.326916198	6.188281865
Myeloproliferative neoplasms	X-14662	Simple mode	6	0.361602608	0.839202877	0.684493088	-1.28323503	2.006440247	1.435628322	0.277139295	7.436797012

Myeloproliferative neoplasms octadecanedioate	Simple mode	10	0.630979273	1.380529018	0.658462566	-2.074857602	3.336816147	1.879450173	0.125574308	28.12942404
Myeloproliferative neoplasms succinylcarnitine	Simple mode	9	-2.555573545	2.040748278	0.245839344	-6.55544017	1.444293079	0.077647685	0.001422357	4.238854549
Myeloproliferative neoplasms 5alpha-androstan-3beta,17beta-diol disulfate	Simple mode	10	0.640033113	0.728948107	0.402762138	-0.788705176	2.068771402	1.896543679	0.454432825	7.915092674
Myeloproliferative neoplasms 4-androsten-3beta,17beta-diol disulfate 1*	Simple mode	5	-0.596019992	0.314795182	0.131240805	-1.213018549	0.020978565	0.551000263	0.297298514	1.021200162
Myeloproliferative neoplasms 4-androsten-3beta,17beta-diol disulfate 2*	Simple mode	11	0.397358844	0.819960511	0.638394143	-1.209763758	2.004481447	1.487889755	0.298267734	7.422244069
Myeloproliferative neoplasms cis-4-decenoyl carnitine	Simple mode	6	-0.492397981	0.84151633	0.583868853	-2.141769988	1.156974026	0.611159088	0.11744678	3.180295211
Myeloproliferative neoplasms glutamine	Wald ratio	1	2.386666667	4.893333333	0.625735135	-7.204266667	11.9776	10.87717619	0.000743407	159149.6128
Myeloproliferative neoplasms phenylalanine	Wald ratio	1	1.026666667	7.666666667	0.893471336	-14	16.05333333	2.791744493	8.32E-07	9372902.143
Myeloproliferative neoplasms deoxycholate	Wald ratio	1	-2.323287671	1.583561644	0.142341231	-5.427068493	0.780493151	0.097951025	0.004395964	2.182548325
Myeloproliferative neoplasms margarate (17:0)	Wald ratio	1	1.544520548	3.02739726	0.609924665	-4.389178082	7.478219178	4.685724508	0.012410926	1769.087539
Myeloproliferative neoplasms inosine	Wald ratio	1	0.323181049	0.82571912	0.695506439	-1.295228426	1.941590525	1.38151545	0.273835306	6.969827841
Myeloproliferative neoplasms palmitate (16:0)	Wald ratio	1	2.986754967	5.854304636	0.609924665	-8.487682119	14.46119205	19.82125738	0.00020599	1907286.393
Myeloproliferative neoplasms nonadecanoate (19:0)	Wald ratio	1	2.478021978	4.857142857	0.609924665	-7.041978022	11.99802198	11.91766768	0.000874395	162433.177
Myeloproliferative neoplasms oleate (18:1n9)	Wald ratio	1	-2.51744186	4.238372093	0.552535968	-10.82465116	5.789767442	0.080665697	1.99E-05	326.9369837
Myeloproliferative neoplasms pentadecanoate (15:0)	Wald ratio	1	-3.352112676	4.816901408	0.486487429	-12.79323944	6.089014085	0.03501031	2.78E-06	440.9864214
Myeloproliferative neoplasms pipecolate	Wald ratio	1	-1.736507937	2.263492063	0.442973952	-6.172952381	2.699936508	0.1761344	0.002085071	14.87878701
Myeloproliferative neoplasms pantothenate	Wald ratio	1	-3.906976744	3.587209302	0.27609171	-10.93790698	3.123953488	0.02010118	1.78E-05	22.73608906
Myeloproliferative neoplasms N-acetylalanine	Wald ratio	1	-5.175257732	9.216494845	0.574442246	-23.23958763	12.88907216	0.005654759	8.08E-11	395961.4611
Myeloproliferative neoplasms heptanoate (7:0)	Wald ratio	1	-2.732673267	5.683168317	0.630632894	-13.87168317	8.406336634	0.065045174	9.45E-07	4475.335651
Myeloproliferative neoplasms cortisone	Wald ratio	1	5.818181818	5.747474747	0.311393543	-5.446868687	17.08323232	336.3599338	0.004309779	26251464.17
Myeloproliferative neoplasms proline	Wald ratio	1	-2.393258427	1.762172285	0.174422549	-5.847116105	1.060599251	0.091331601	0.002888216	2.888101168
Myeloproliferative neoplasms hypoxanthine	Wald ratio	1	0.975247525	2.801980198	0.727797724	-4.516633663	6.467128713	2.651823522	0.010925742	643.6330161
Myeloproliferative neoplasms pelargonate (9:0)	Wald ratio	1	-1.86	6.49	0.774422626	-14.5804	10.8604	0.15567263	4.65E-07	52072.90318
Myeloproliferative neoplasms beta-hydroxyisovalerate	Wald ratio	1	-6.79245283	4.080188679	0.095964662	-14.78962264	1.204716981	0.001122213	3.78E-07	3.335814846
Myeloproliferative neoplasms acetylphosphate	Wald ratio	1	7.459183673	7.255102041	0.303888965	-6.760816327	21.67918367	1735.730556	0.001158283	2601056833
Myeloproliferative neoplasms N1-methyladenosine	Wald ratio	1	18.25	10.22222222	0.07420844	-1.785555556	38.28555556	84309069.23	0.167703866	4.23843E+16
Myeloproliferative neoplasms 3-phenylpropionate (hydrocinnamate)	Wald ratio	1	1.01650165	2.04950495	0.619912589	-3.000528053	5.033531353	2.763510109	0.049760785	153.4740279
Myeloproliferative neoplasms X-04499	Wald ratio	1	-6.28959276	3.43438914	0.067047268	-13.02099548	0.441809955	0.001855515	2.21E-06	1.555520093
Myeloproliferative neoplasms indolelactate	Wald ratio	1	3.612565445	3.554973822	0.309534016	-3.355183246	10.58031414	37.06100894	0.034902974	39352.4745
Myeloproliferative neoplasms estrone 3-sulfate	Wald ratio	1	0.371033968	0.417319895	0.373955972	-0.446913027	1.188980963	1.4492323	0.639599534	3.283733256
Myeloproliferative neoplasms X-06246	Wald ratio	1	3.304545455	2.727272727	0.225640004	-2.040909091	8.65	27.2361587	0.129910557	5710.146734
Myeloproliferative neoplasms glucose	Wald ratio	1	-5.050505051	6.212121212	0.416213397	-17.22626263	7.125252525	0.006406097	3.30E-08	1242.962007
Myeloproliferative neoplasms 3-methyl-2-oxobutyrate	Wald ratio	1	3.5	4.680327869	0.454574252	-5.673442623	12.67344262	33.11545196	0.003436016	319158.3402
Myeloproliferative neoplasms 1,6-anhydroglucose	Wald ratio	1	2.667355372	1.54338843	0.083944073	-0.35768595	5.692396694	14.4018313	0.699292653	296.6036377
Myeloproliferative neoplasms 1-oleoylglycerol (1-monoolein)	Wald ratio	1	0.083521445	1.803611738	0.963064855	-3.451557562	3.618600451	1.087108528	0.031696229	37.28534863
Myeloproliferative neoplasms phenyllactate (PLA)	Wald ratio	1	3.627507163	2.936962751	0.216785121	-2.128939828	9.383954155	37.61892181	0.118963349	11895.96035
Myeloproliferative neoplasms homocitrulline	Wald ratio	1	0.929230769	1.858461538	0.617075077	-2.713353846	4.571815385	2.532560305	0.066314026	96.71953365
Myeloproliferative neoplasms X-10429	Wald ratio	1	-3.061728395	3.586419753	0.393270948	-10.09111111	3.967654321	0.046806725	4.14E-05	52.86039179
Myeloproliferative neoplasms indoleacetate	Wald ratio	1	-1.821052632	3.38245614	0.590313528	-8.450666667	4.808561404	0.161855287	0.000213758	122.5551832
Myeloproliferative neoplasms N-(2-furoyl)glycine	Wald ratio	1	0.409718056	0.910617876	0.652757942	-1.375092981	2.194529094	1.506393007	0.252816088	8.975773318
Myeloproliferative neoplasms glycochenodeoxycholate	Wald ratio	1	-1.73512252	1.470245041	0.23793709	-4.6168028	1.14655776	0.176378589	0.009884348	3.14734034
Myeloproliferative neoplasms dodecanedioate	Wald ratio	1	-0.994444444	2.494444444	0.69014102	-5.883555556	3.894666667	0.369928904	0.002784866	49.13967074
Myeloproliferative neoplasms gamma-glutamylvaline	Wald ratio	1	-0.208633094	5	0.966716634	-10.00863309	9.591366906	0.811692996	4.50E-05	14637.86446
Myeloproliferative neoplasms indolepropionate	Wald ratio	1	0.900584795	1.815789474	0.619912589	-2.658362573	4.459532164	2.461041896	0.070062851	86.44705657
Myeloproliferative neoplasms myristoleate (14:1n5)	Wald ratio	1	-1.225543478	2.402173913	0.609924665	-5.933804348	3.482717391	0.293598093	0.002648387	32.5480478
Myeloproliferative neoplasms X-11334	Wald ratio	1	3.009950249	3.288557214	0.360044719	-3.435621891	9.455522388	20.28639063	0.032205376	12778.53882
Myeloproliferative neoplasms X-11412	Wald ratio	1	-3	5.575221239	0.5905113	-13.92743363	7.927433628	0.049787068	8.94E-07	2772.302907
Myeloproliferative neoplasms X-01911	Wald ratio	1	-0.146788991	1.300458716	0.910129832	-2.695688073	2.402110092	0.86347616	0.067495924	11.04646085
Myeloproliferative neoplasms X-11444	Wald ratio	1	4.353658537	2.085365854	0.036823163	0.266341463	8.44097561	77.76243987	1.305180654	4633.072851
Myeloproliferative neoplasms X-11452	Wald ratio	1	-0.185840708	1.740412979	0.914963783	-3.597050147	3.225368732	0.830405861	0.027404442	25.16285078
Myeloproliferative neoplasms X-11470	Wald ratio	1	1.064738292	2.159779614	0.622023402	-3.168429752	5.297906336	2.900079911	0.042069606	199.9178108
Myeloproliferative neoplasms X-11478	Wald ratio	1	0.762376238	1.537128713	0.619912589	-2.25039604	3.775148515	2.143363315	0.105357491	43.60398367
Myeloproliferative neoplasms X-11483	Wald ratio	1	-2.567990373	1.208182912	0.03354508	-4.936028881	-0.199951865	0.076689508	0.007183067	0.818770163
Myeloproliferative neoplasms X-11521	Wald ratio	1	-3.398104265	3.004739336	0.258090942	-9.287393365	2.491184834	0.033436597	9.26E-05	12.0755752
Myeloproliferative neoplasms X-11550	Wald ratio	1	-3.725274725	6.923076923	0.5905113	-17.29450549	9.843956044	0.024106477	3.08E-08	18844.11691
Myeloproliferative neoplasms X-11786	Wald ratio	1	0.450331126	1.419426049	0.751044179	-2.331743929	3.232406181	1.56883158	0.097126218	25.34055764
Myeloproliferative neoplasms X-11799	Wald ratio	1	-0.522237197	0.533692722	0.327809615	-1.568274933	0.523800539	0.593191975	0.208404384	1.688432425
Myeloproliferative neoplasms X-11858	Wald ratio	1	0.085749537	0.43923504	0.845217028	-0.775151141	0.946650216	1.089533407	0.460634155	2.577062581
Myeloproliferative neoplasms 1-palmitoleoylglycerophosphocholine*	Wald ratio	1	-1.645985401	3.226277372	0.609924665	-7.969489051	4.677518248	0.192822462	0.000345856	107.5029456
Myeloproliferative neoplasms X-12013	Wald ratio	1	0.265663598	0.36626993	0.468254581	-0.452225466	0.983552661	1.304296216	0.63621071	2.673938987
Myeloproliferative neoplasms palmitoleate (16:1n7)	Wald ratio	1	-1.228882834	2.408719346	0.609924665	-5.949972752	3.492207084	0.2926193	0.002605912	32.85838897
Myeloproliferative neoplasms alpha-ketoglutarate	Wald ratio	1	-1.263473054	1.129341317	0.263238323	-3.476982036	0.950035928	0.28267059	0.030900527	2.585802561
Myeloproliferative neoplasms X-12094	Wald ratio	1	-0.676470588	2.833333333	0.811296089	-6.229803922	4.876862745	0.508408211	0.001969838	131.218352
Myeloproliferative neoplasms X-12095	Wald ratio	1	-0.762430939	3.193370166	0.811296089	-7.021436464	5.496574586	0.466530939	0.000892542	243.8551949
Myeloproliferative neoplasms X-12435	Wald ratio	1	-0.793206198	0.401519666	0.048210278	-1.580184744	-0.006227652	0.452392009	0.205937049	0.9937917
Myeloproliferative neoplasms X-12441	Wald ratio	1	0.290379523	0.508384819	0.567877257	-0.706054722	1.286813769	1.33693479	0.493587702	3.621230078
Myeloproliferative neoplasms 5-dodecenoate (12:1n7)	Wald ratio	1	-1.464285714	2.87012987	0.609924665	-7.08974026	4.161168831	0.231243106	0.000833614	64.14645518
Myeloproliferative neoplasms 10-nonadecenoate (19:1n9)	Wald ratio	1	-1.86637931	3.142241379	0.552535968	-8.025172414	4.292413793	0.154682707	0.000327124	73.1428072
Myeloproliferative neoplasms X-12740	Wald ratio	1	-0.920077973	1.115009747	0.409272344	-3.105497076	1.265341131	0.398487969	0.044802243	3.5443016
Myeloproliferative neoplasms X-12749	Wald ratio	1	-9.751004016	5.995983936	0.103895237	-21.50313253	2.001124498	5.82E-05	4.58E-10	7.397369751
Myeloproliferative neoplasms X-12990	Wald ratio	1	0.705989111	1.156079855	0.541414734	-1.559927405	2.971905626	2.025849484	0.210151327	19.52909932
Myeloproliferative neoplasms X-13069	Wald ratio	1	2.803108808	2.727979275	0.304166091	-2.54373057	8.149948187	16.49584957	0.078572731	3463.19962
Myeloproliferative neoplasms phenylacetylglutamine	Wald ratio	1	-0.541871921	1.674876847	0.746294337	-3.824630542	2.7408867	0.581658414	0.021826498	15.50072349
Myeloproliferative neoplasms pro-hydroxy-pro	Wald ratio	1	-0.01459854	4.131386861	0.997180626	-8.112116788	8.082919708	0.985507502	0.000299883	3238.675432
Myeloproliferative neoplasms N2,N2-dimethylguanosine	Wald ratio	1	3.853211009	5.449541284	0.479522539	-6.827889908	14.53431193	47.14420091	0.001083141	2051972.178
Myeloproliferative neoplasms cysteine-glutathione disulfide	Wald ratio	1	-0.837398374	1.221544715	0.493013241	-3.231626016	1.556829268	0.432835135	0.03949323	4.743756196
Myeloproliferative neoplasms 2-stearoylglycerophosphocholine*	Wald ratio	1	2.938566553	3.638225256	0.419267733	-4.192354949	10.06948805	18.88875087	0.015110658	23611.47389
Myeloproliferative neoplasms 2-linoleoylglycerophosphocholine*	Wald ratio	1	2.853658537	3.463414634	0.40997157	-3.934634146	9.64195122	17.35114566	0.019552852	15397.35807
Myeloproliferative neoplasms 2-methylbutyroylcarnitine	Wald ratio	1	-4.304812834	4.267379679	0.313084026	-12.66887701	4.059251337	0.013503413	3.15E-06	57.93092409
Myeloproliferative neoplasms X-13658	Wald ratio	1	-0.205250597	0.908114558	0.821187112	-1.985155131	1.574653938	0.814443194	0.137359304	4.829070171
Myeloproliferative neoplasms X-13671	Wald ratio	1	-8.320224719	3.241573034	0.010266416	-14.67370787	-1.966741573	0.000243541	4.24E-07	0.139912007
Myeloproliferative neoplasms X-13741	Wald ratio	1	-0.442367601	1.387850467	0.749921416	-3.162554517	2.277819315	0.642513403	0.042317502	9.755383775
Myeloproliferative neoplasms 2-hydroxypalmitate	Wald ratio	1	-1.457142857	6	0.808116067	-13.21714286	10.30285714	0.232900756	1.82E-06	29817.69067
Myeloproliferative neoplasms X-13859	Wald ratio	1	4.055555556	5.259259259	0.4406318	-6.252592593	14.3637037	57.71721921	0.001925456	1730124.11
Myeloproliferative neoplasms X-14473	Wald ratio	1	1.947743468	1.77672209	0.272966459	-1.534631829	5.430118765	7.012845003	0.21553503	228.1763431
Myeloproliferative neoplasms dimethylarginine (SDMA + ADMA)	Wald ratio	1	14.48979592	6.285714286	0.021155666	2.169795918	26.80979592	1962629.902	8.756496818	4.39892E+11
Myeloproliferative neoplasms tryptophan betaine	Wald ratio	1	-0.611550633	0.651107595	0.347604118	-1.887721519	0.664620253	0.542508984	0.151416416	1.943752247
Myeloproliferative neoplasms tryptophan	Weighted median	18	1.495852673	3.630925344	0.680357302	-5.620761001	8.612466346	4.46314053	0.003621884	5499.796367
Myeloproliferative neoplasms leucine	Weighted median	12	-2.603950048	3.970999716	0.511990343	-10.38710949	5.179209396	0.073980773	3.08E-05	177.5423897
Myeloproliferative neoplasms cholesterol	Weighted median	3	-1.494982879	3.778163987	0.692333505	-8.900184293	5.910218535	0.224252444	0.000136364	368.7867393
Myeloproliferative neoplasms uridine	Weighted median	3	3.111344105	4.193389792	0.45810917	-5.107699887	11.3303881	22.45120091	0.006049983	83315.3511
Myeloproliferative neoplasms arachidonate (20:4n6)	Weighted median	11	-0.705260618	0.487367706	0.147874198	-1.660501321	0.249980086	0.493979818	0.190043683	1.283999847
Myeloproliferative neoplasms tyrosine	Weighted median	5	2.223296904	3.937757822	0.572339155	-5.494708426	9.941302235	9.23773665	0.004108454	20770.77511
Myeloproliferative neoplasms citrate	Weighted median	6	0.462361056	2.651899995	0.861589727	-4.735362934	5.660085047	1.587818492	0.008779262	287.1730646
Myeloproliferative neoplasms citrulline	Weighted median	4	3.346013775	3.413704277	0.32700174	-3.344846609	10.03687416	28.38934148	0.035265624	22853.83369
Myeloproliferative neoplasms gamma-glutamylglutamine	Weighted median	3	1.184247839	2.711992455	0.662350129	-4.131257374	6.499753051	3.268227662	0.016062669	664.9773974
Myeloproliferative neoplasms gamma-glutamyltyrosine	Weighted median	5	5.073462385	3.852490879	0.187861953	-2.477419738	12.62434451	159.7264053	0.083959584	303866.7334
Myeloproliferative neoplasms betaine	Weighted median	3	2.966823987	2.12781627	0.163225646	-1.203695902	7.137343875	19.4301112	0.300083082	1258.082324
Myeloproliferative neoplasms X-03094	Weighted median	6	0.524710651	1.881064975	0.780288265	-3.162176701	4.211598003	1.689969786	0.042333493	67.46426178
Myeloproliferative neoplasms kynurenine	Weighted median	6	1.214407943	1.543755947	0.431482105	-1.811353714	4.2401696	3.36829925	0.163432746	69.41962442
Myeloproliferative neoplasms carnitine	Weighted median	26	-1.126690287	1.550538379	0.46744372	-4.165745509	1.912364935	0.324104175	0.015518142	6.769078313
Myeloproliferative neoplasms N-acetylornithine	Weighted median	4	0.083270611	0.234264561	0.722248429	-0.37588793	0.542429151	1.086835878	0.686679285	1.720180369
Myeloproliferative neoplasms 3-methyl-2-oxovalerate	Weighted median	4	2.382434028	2.895876526	0.410679321	-3.293483964	8.05835202	10.83123433	0.037124284	3160.078094
Myeloproliferative neoplasms X-18601	Weighted median	11	0.503182784	0.62576915	0.421337554	-0.72332475	1.729690317	1.653977153	0.485136614	5.638907367
Myeloproliferative neoplasms eicosapentaenoate (EPA; 20:5n3)	Weighted median	9	-0.925795428	0.702083626	0.187289554	-2.301879336	0.45028848	0.396216132	0.100070601	1.568764677
Myeloproliferative neoplasms myo-inositol	Weighted median	3	0.787394707	2.920284856	0.787445366	-4.936363611	6.511153026	2.197663405	0.007180663	672.6014974
Myeloproliferative neoplasms 1,5-anhydroglucitol (1,5-AG)	Weighted median	5	-1.61212241	1.123893075	0.151455563	-3.814952837	0.590708017	0.19946382	0.022038754	1.805266122
Myeloproliferative neoplasms X-08402	Weighted median	3	-0.581678122	0.888605289	0.512727426	-2.323344489	1.159988244	0.558959577	0.09794546	3.189895776
Myeloproliferative neoplasms 2-hydroxyisobutyrate	Weighted median	3	-0.202673719	2.017301952	0.919973012	-4.156585544	3.751238106	0.816544621	0.01566094	42.57376023
Myeloproliferative neoplasms 4-methyl-2-oxopentanoate	Weighted median	3	3.510522834	3.07340762	0.253360532	-2.513356102	9.53440177	33.46576024	0.080995952	13827.32201
Myeloproliferative neoplasms palmitoylcarnitine	Weighted median	6	-2.803009925	1.372648093	0.041147014	-5.493400186	-0.112619664	0.060627304	0.004113833	0.893490422
Myeloproliferative neoplasms X-10510	Weighted median	4	-0.893338689	1.384664127	0.518819395	-3.607280378	1.820602999	0.409286987	0.027125518	6.175581198
Myeloproliferative neoplasms N-acetylglycine	Weighted median	3	-0.741656069	0.615985452	0.228583564	-1.948987554	0.465675416	0.476324436	0.142418189	1.593089824
Myeloproliferative neoplasms androsterone sulfate	Weighted median	9	0.448314477	0.172121835	0.00919723	0.110955681	0.785673274	1.565670986	1.117345386	2.193883527
Myeloproliferative neoplasms 3-carboxy-4-methyl-5-propyl-2-furanpropanoate (CMPI	Weighted median	5	0.050544068	0.652942546	0.938297647	-1.229223322	1.330311459	1.051843215	0.292519683	3.782221209
Myeloproliferative neoplasms acetylcarnitine	Weighted median	12	-2.387943567	1.222245366	0.050732563	-4.783544485	0.007657351	0.091818308	0.008366292	1.007686744
Myeloproliferative neoplasms serine	Weighted median	3	-0.930616845	2.067003936	0.652548212	-4.98194456	3.120710869	0.394310407	0.006860709	22.66248398
Myeloproliferative neoplasms hexanoylcarnitine	Weighted median	17	-0.455328217	0.563981999	0.419467571	-1.560732934	0.6500765	0.634239765	0.209982112	1.915687373
Myeloproliferative neoplasms butyrylcarnitine	Weighted median	12	0.440955839	0.403855116	0.27489182	-0.350600187	1.232511866	1.554192066	0.704265272	3.429834007
Myeloproliferative neoplasms dehydroisoandrosterone sulfate (DHEA-S)	Weighted median	7	1.734072778	0.75579084	0.021768575	0.252722732	3.215422824	5.663673886	1.287526238	24.91382385
Myeloproliferative neoplasms propionylcarnitine	Weighted median	14	-1.981292435	1.061487372	0.061967987	-4.061807684	0.099222814	0.137890907	0.017217867	1.104312329
Myeloproliferative neoplasms 10-undecenoate (11:1n1)	Weighted median	3	0.460917908	0.916900005	0.615181134	-1.336206102	2.258041918	1.585528687	0.262840971	9.56434305
Myeloproliferative neoplasms docosapentaenoate (n3 DPA; 22:5n3)	Weighted median	8	-1.389962088	0.971439338	0.152479669	-3.29398319	0.514059014	0.249084748	0.037105755	1.672064372
Myeloproliferative neoplasms X-11204	Weighted median	3	3.512663796	3.56193615	0.324051183	-3.468731057	10.49405865	33.53748592	0.031156541	36100.37923
Myeloproliferative neoplasms X-02269	Weighted median	13	-0.276000401	0.438134655	0.528731068	-1.134744326	0.582743523	0.758812626	0.321504311	1.790945196
Myeloproliferative neoplasms X-11247	Weighted median	3	-0.00867133	0.834712054	0.991711398	-1.644706956	1.627364297	0.991366158	0.193069132	5.090440131
Myeloproliferative neoplasms X-11261	Weighted median	5	-0.899297767	0.933941663	0.33559484	-2.729823427	0.931227892	0.406855266	0.065230807	2.537623193
Myeloproliferative neoplasms X-02249	Weighted median	3	-0.430093258	1.595719292	0.787522324	-3.55770307	2.697516553	0.650448432	0.028504222	14.84282455
Myeloproliferative neoplasms X-11315	Weighted median	4	3.972966056	1.88133029	0.03470485	0.285558688	7.660373424	53.14191921	1.330505157	2122.549892
Myeloproliferative neoplasms X-11317	Weighted median	3	1.559982805	2.850142458	0.584148583	-4.026296414	7.146262024	4.758739418	0.017840281	1269.352267
Myeloproliferative neoplasms 1-linoleoylglycerophosphoethanolamine*	Weighted median	12	0.820708064	0.567226332	0.147930719	-0.291055546	1.932471675	2.272108067	0.747474158	6.906559932
Myeloproliferative neoplasms 3-dehydrocarnitine*	Weighted median	13	-2.177100477	0.962364778	0.023682555	-4.063335441	-0.290865513	0.113369773	0.017191582	0.747616216
Myeloproliferative neoplasms pyroglutamine*	Weighted median	5	-2.433513787	1.008325872	0.015803598	-4.409832497	-0.457195078	0.087728033	0.012157215	0.633056833
Myeloproliferative neoplasms X-11381	Weighted median	4	-1.279972542	1.882091127	0.496453805	-4.968871152	2.408926067	0.278044935	0.00695099	11.12201043
Myeloproliferative neoplasms X-03056	Weighted median	3	0.954134985	1.616882399	0.555118351	-2.214954517	4.123224487	2.596423665	0.109158479	61.75805959
Myeloproliferative neoplasms X-11440	Weighted median	6	-0.652025782	0.287474606	0.023322131	-1.215476011	-0.088575554	0.520989296	0.296568811	0.915233958
Myeloproliferative neoplasms X-11445	Weighted median	9	0.28113762	0.420543781	0.503808257	-0.543128191	1.105403431	1.324635888	0.580928153	3.020442763
Myeloproliferative neoplasms X-11469	Weighted median	10	0.053264707	0.511902451	0.917127721	-0.950064097	1.05659351	1.054708797	0.386716235	2.876555324
Myeloproliferative neoplasms X-11491	Weighted median	9	0.209821876	0.334486792	0.530465911	-0.445772235	0.865415988	1.233458332	0.6403296	2.375994265
Myeloproliferative neoplasms X-11529	Weighted median	14	0.062021195	0.10820526	0.566522176	-0.150061115	0.274103504	1.063984895	0.860655376	1.31535094
Myeloproliferative neoplasms X-11538	Weighted median	15	0.153347067	0.259673808	0.554830492	-0.355613596	0.662307731	1.165729495	0.700743338	1.93926247
Myeloproliferative neoplasms X-11593	Weighted median	6	-0.16143333	1.110399036	0.884408353	-2.33781544	2.014948781	0.850923261	0.096538302	7.500343208

Myeloproliferative neoplasms adrenate (22:4n6)	Weighted median	9	-0.993555555	0.718613247	0.166787174	-2.402037519	0.41492641	0.370257876	0.090533302	1.514259302
Myeloproliferative neoplasms X-11787	Weighted median	6	0.474539153	1.029217888	0.644750014	-1.542727907	2.491806213	1.60727332	0.213797087	12.08308104
Myeloproliferative neoplasms X-11792	Weighted median	4	0.140878384	0.400368264	0.724934254	-0.643843413	0.925600181	1.151284625	0.525269711	2.523382292
Myeloproliferative neoplasms X-11793	Weighted median	3	0.381334558	0.440804999	0.386991185	-0.482643241	1.245312357	1.464237395	0.617149958	3.474019763
Myeloproliferative neoplasms 1-arachidonoylglycerophosphocholine*	Weighted median	13	-0.613356314	0.442456494	0.16566989	-1.480571042	0.253858415	0.54153027	0.227507735	1.28898929
Myeloproliferative neoplasms X-11905	Weighted median	11	0.218358453	0.358952095	0.542973938	-0.485187653	0.921904559	1.244032915	0.61558167	2.514074035
Myeloproliferative neoplasms X-12038	Weighted median	4	-0.199403446	2.533478883	0.937265388	-5.165022057	4.766215164	0.819219316	0.005712937	117.4737805
Myeloproliferative neoplasms X-12063	Weighted median	20	0.15887141	0.241777935	0.511118936	-0.315013342	0.632756162	1.172187205	0.729779137	1.882792716
Myeloproliferative neoplasms isobutyrylcarnitine	Weighted median	5	-1.096993708	0.963868842	0.255072413	-2.986176639	0.792189223	0.333873297	0.050480072	2.208225436
Myeloproliferative neoplasms erythronate*	Weighted median	3	5.974552795	4.015976387	0.136831031	-1.896760923	13.84586651	393.2921796	0.150053869	1030821.396
Myeloproliferative neoplasms X-12092	Weighted median	6	-0.343741836	0.155835002	0.027397562	-0.64917844	-0.038305232	0.709111972	0.522474845	0.962419135
Myeloproliferative neoplasms X-12093	Weighted median	5	-0.377956302	0.37955431	0.319352298	-1.121882748	0.365970145	0.685260445	0.32566607	1.441912194
Myeloproliferative neoplasms X-12100	Weighted median	4	1.355476026	2.647582279	0.60867377	-3.833785241	6.544737293	3.878606834	0.021627595	695.5739229
Myeloproliferative neoplasms X-12244	Weighted median	3	0.464701111	1.885291526	0.80530475	-3.230470279	4.159872502	1.591538425	0.0395389	64.06335414
Myeloproliferative neoplasms 1-eicosatrienoylglycerophosphocholine*	Weighted median	11	1.28577156	0.991917559	0.194889916	-0.658386856	3.229929976	3.617457966	0.517685763	25.27788685
Myeloproliferative neoplasms 1-eicosadienoylglycerophosphocholine*	Weighted median	3	2.000664014	2.028765657	0.324060294	-1.975716673	5.977044701	7.393964165	0.138661901	394.2734488
Myeloproliferative neoplasms X-12442	Weighted median	3	2.034737293	0.942633357	0.030883772	0.187175914	3.882298672	7.650242092	1.205839391	48.5356545
Myeloproliferative neoplasms X-12456	Weighted median	12	0.224879086	0.388841142	0.563040323	-0.537249552	0.987007724	1.252171302	0.584353277	2.683193593
Myeloproliferative neoplasms octanoylcarnitine	Weighted median	7	-0.411400068	0.500935834	0.411496476	-1.393234304	0.570434167	0.662721745	0.248271021	1.769034941
Myeloproliferative neoplasms alpha-hydroxyisovalerate	Weighted median	3	1.295163943	1.263274	0.305248126	-1.180853097	3.771180983	3.651594579	0.307016712	43.43132623
Myeloproliferative neoplasms decanoylcarnitine	Weighted median	7	-0.36796386	0.550517859	0.503880935	-1.446978863	0.711051143	0.692142195	0.235280029	2.036130398
Myeloproliferative neoplasms stearidonate (18:4n3)	Weighted median	9	-0.958942266	0.748827139	0.200336907	-2.426643459	0.508758928	0.383298099	0.088332828	1.66322573
Myeloproliferative neoplasms epiandrosterone sulfate	Weighted median	5	0.60936163	0.241405726	0.01159547	0.136206407	1.082516853	1.839256898	1.145918395	2.952100212
Myeloproliferative neoplasms X-12510	Weighted median	9	0.153419778	0.412450945	0.709914464	-0.654984074	0.96182363	1.165814259	0.519450335	2.616463586
Myeloproliferative neoplasms 1-arachidonoylglycerophosphoinositol*	Weighted median	17	0.384400493	0.619902836	0.535193285	-0.830609066	1.599410053	1.468733542	0.435783785	4.950111257
Myeloproliferative neoplasms X-12644	Weighted median	7	1.074183834	1.365367348	0.431436345	-1.601936168	3.750303836	2.927602516	0.201505991	42.53400338
Myeloproliferative neoplasms X-12696	Weighted median	3	-1.982836768	1.355247656	0.143445282	-4.639122175	0.673448639	0.137678122	0.009666179	1.960988413
Myeloproliferative neoplasms X-12728	Weighted median	3	-0.053870892	0.101474342	0.595500619	-0.252760603	0.145018819	0.947554435	0.776653788	1.156061326
Myeloproliferative neoplasms isovalerylcarnitine	Weighted median	11	-1.369012344	0.605413515	0.023741631	-2.555622833	-0.182401854	0.254358054	0.077643858	0.833266422
Myeloproliferative neoplasms stearoylcarnitine	Weighted median	6	-2.676707382	1.291931828	0.038278102	-5.208893765	-0.144520999	0.068789279	0.005467719	0.865436739
Myeloproliferative neoplasms 1-stearoylglycerophosphoethanolamine	Weighted median	3	1.281838748	1.493828206	0.390842879	-1.646064536	4.209742032	3.603259123	0.192807203	67.3391662
Myeloproliferative neoplasms bradykinin, des-arg(9)	Weighted median	6	0.063716501	0.224964268	0.777000971	-0.377213464	0.504646465	1.065790205	0.685769671	1.656399822
Myeloproliferative neoplasms X-12798	Weighted median	10	-0.969171692	0.429480515	0.024032212	-1.810953502	-0.127389882	0.379397166	0.163498167	0.880390357
Myeloproliferative neoplasms X-12844	Weighted median	3	2.336190146	1.956459604	0.232442505	-1.498470678	6.170850971	10.34176082	0.223471659	478.5932017
Myeloproliferative neoplasms X-12850	Weighted median	6	-1.278367395	0.684868801	0.061959245	-2.620710245	0.063975456	0.278491596	0.072751173	1.066066233
Myeloproliferative neoplasms laurylcarnitine	Weighted median	3	0.47018664	1.44672177	0.745179992	-2.365388029	3.305761308	1.600292843	0.093912852	27.26929403
Myeloproliferative neoplasms X-12855	Weighted median	6	0.616232935	1.304348962	0.636609306	-1.940291032	3.172756901	1.851938512	0.143662133	23.8732098
Myeloproliferative neoplasms X-13215	Weighted median	3	1.898678962	3.107950112	0.541258917	-4.192903256	7.990261181	6.677067956	0.015102375	2952.067883
Myeloproliferative neoplasms oleoylcarnitine	Weighted median	6	-2.569270048	1.331614163	0.053676223	-5.179233807	0.040693711	0.076591433	0.00563232	1.041533047
Myeloproliferative neoplasms 1-arachidonoylglycerophosphoethanolamine*	Weighted median	18	-0.569952841	0.620503887	0.358340275	-1.78614046	0.646234778	0.565552109	0.167605804	1.908341955
Myeloproliferative neoplasms X-13429	Weighted median	16	0.125457717	0.218404352	0.565677771	-0.302614813	0.553530247	1.133667233	0.73888365	1.739382643
Myeloproliferative neoplasms X-13431	Weighted median	4	-0.333928069	0.508337711	0.51124392	-1.330269983	0.662413845	0.71610529	0.264405866	1.939468265
Myeloproliferative neoplasms X-13435	Weighted median	3	-2.007772506	1.51178032	0.184150085	-4.970861934	0.955316921	0.134287466	0.006937166	2.599494287
Myeloproliferative neoplasms X-13477	Weighted median	3	0.740102312	1.894520083	0.696052824	-2.973157051	4.453361676	2.096149965	0.051141599	85.91527838
Myeloproliferative neoplasms glutaroyl carnitine	Weighted median	11	0.214668171	1.018870324	0.833127353	-1.782317663	2.211654005	1.239450543	0.168247753	9.130806314
Myeloproliferative neoplasms 2-tetradecenoyl carnitine	Weighted median	4	-2.285674785	1.27447884	0.072905924	-4.783653311	0.212303741	0.10170541	0.008365382	1.236523411
Myeloproliferative neoplasms 1-palmitoylglycerophosphoethanolamine	Weighted median	6	1.24200147	1.284218291	0.333480827	-1.27506638	3.759069319	3.462536697	0.27941242	42.90847331
Myeloproliferative neoplasms tetradecanedioate	Weighted median	16	0.168609442	0.297483919	0.57086022	-0.41445904	0.751677924	1.183657762	0.660697595	2.120555164
Myeloproliferative neoplasms hexadecanedioate	Weighted median	15	0.187952013	0.319925764	0.556877128	-0.439102485	0.815006511	1.206775605	0.644614713	2.259190383
Myeloproliferative neoplasms dihomo-linolenate (20:3n3 or n6)	Weighted median	10	1.76069672	1.154410585	0.127211446	-0.501948026	4.023341466	5.816488446	0.605350272	55.88754047
Myeloproliferative neoplasms X-14189	Weighted median	3	0.385393354	0.675736882	0.568453558	-0.939050935	1.709837642	1.470192513	0.390998743	5.528063881
Myeloproliferative neoplasms X-14205	Weighted median	3	0.783265575	0.901449371	0.384904125	-0.983575191	2.550106341	2.188607671	0.373971686	12.80846578
Myeloproliferative neoplasms X-14208	Weighted median	3	0.516385689	0.695572368	0.457851321	-0.846936151	1.87970753	1.675959252	0.428726475	6.551588437
Myeloproliferative neoplasms X-14304	Weighted median	3	0.411897996	0.823516124	0.616955435	-1.202193607	2.0259896	1.509680436	0.300534234	7.583611972
Myeloproliferative neoplasms X-14626	Weighted median	10	0.34201176	0.559652237	0.541123588	-0.754906624	1.438930144	1.407776853	0.470054504	4.216182695
Myeloproliferative neoplasms X-14662	Weighted median	6	0.369169765	0.616923701	0.549570231	-0.84000069	1.578340219	1.446533153	0.431710226	4.846904331
Myeloproliferative neoplasms octadecanedioate	Weighted median	10	0.494267728	0.883295289	0.575771086	-1.236991039	2.225526494	1.639297387	0.290256275	9.258355993
Myeloproliferative neoplasms succinylcarnitine	Weighted median	9	-2.162593317	1.311252094	0.099094741	-4.732647421	0.407460786	0.115026434	0.008803135	1.502996506
Myeloproliferative neoplasms 5alpha-androstan-3beta,17beta-diol disulfate	Weighted median	10	0.76949556	0.335054311	0.021639924	0.11278911	1.42620201	2.158677056	1.119395838	4.162858636
Myeloproliferative neoplasms 4-androsten-3beta,17beta-diol disulfate 1*	Weighted median	5	-0.610143386	0.261630151	0.019696293	-1.122938481	-0.09734829	0.543272966	0.325322435	0.907239968
Myeloproliferative neoplasms 4-androsten-3beta,17beta-diol disulfate 2*	Weighted median	11	0.376907296	0.571060502	0.509245553	-0.742371288	1.496185881	1.457769163	0.475983881	4.464627931
Myeloproliferative neoplasms cis-4-decenoyl carnitine	Weighted median	6	-0.503925629	0.680161867	0.458759691	-1.837042888	0.82919163	0.604154313	0.159287762	2.29146564
Myeloproliferative neoplasms tryptophan	Weighted mode	18	0.02773935	6.381371213	0.996582269	-12.47974823	12.53522693	1.028127668	3.80E-06	277958.4516
Myeloproliferative neoplasms leucine	Weighted mode	12	-6.288517599	7.034736072	0.390502017	-20.0766003	7.499565103	0.001857511	1.91E-09	1807.256273
Myeloproliferative neoplasms cholesterol	Weighted mode	3	-4.964502209	3.999509354	0.340340082	-12.80354054	2.874536126	0.006981425	2.75E-06	17.71720366
Myeloproliferative neoplasms uridine	Weighted mode	3	1.434060137	5.247688151	0.810275295	-8.851408638	11.71952891	4.195699774	0.00014318	122949.4917
Myeloproliferative neoplasms arachidonate (20:4n6)	Weighted mode	11	-0.62307693	0.690389855	0.388013001	-1.976241047	0.730087186	0.536291764	0.13858921	2.075261534
Myeloproliferative neoplasms tyrosine	Weighted mode	5	7.159236079	7.519664407	0.394975292	-7.579306159	21.89777832	1285.92821	0.000510916	3236564671
Myeloproliferative neoplasms citrate	Weighted mode	6	-0.459756497	4.680076999	0.925560812	-9.632707415	8.713194422	0.631437384	6.55E-05	6082.642013
Myeloproliferative neoplasms citrulline	Weighted mode	4	5.368258858	4.532674496	0.321559985	-3.515783153	14.25230087	214.4890865	0.029724515	1547731.502
Myeloproliferative neoplasms gamma-glutamylglutamine	Weighted mode	3	1.339223447	2.992484375	0.698295379	-4.526045928	7.204492823	3.816078968	0.010823388	1345.462128
Myeloproliferative neoplasms gamma-glutamyltyrosine	Weighted mode	5	7.991682953	5.566667555	0.224443506	-2.918985454	18.90235136	2956.268035	0.053988433	161877649.8
Myeloproliferative neoplasms betaine	Weighted mode	3	2.838729947	2.653496526	0.396703773	-2.362123244	8.039583137	17.0940414	0.094219959	3101.320095
Myeloproliferative neoplasms X-03094	Weighted mode	6	0.367699961	2.390289779	0.883759292	-4.317268005	5.052667928	1.444408595	0.013336268	156.4392769
Myeloproliferative neoplasms kynurenine	Weighted mode	6	1.023958035	1.67434504	0.567565419	-2.257758244	4.305674314	2.784192917	0.104584676	74.11917819
Myeloproliferative neoplasms carnitine	Weighted mode	26	-1.720615352	1.541196687	0.274860214	-4.741360859	1.300130155	0.178955993	0.008726762	3.669774277
Myeloproliferative neoplasms N-acetylornithine	Weighted mode	4	0.078319228	0.225987272	0.751802125	-0.364615825	0.521254282	1.081467839	0.694463395	1.68413871
Myeloproliferative neoplasms 3-methyl-2-oxovalerate	Weighted mode	4	2.163399524	3.727068786	0.60233402	-5.141655297	9.468454344	8.700665558	0.005848002	12944.86346
Myeloproliferative neoplasms X-18601	Weighted mode	11	0.452907819	0.840598163	0.601817518	-1.194664581	2.100480219	1.57287919	0.302805504	8.170092408
Myeloproliferative neoplasms eicosapentaenoate (EPA; 20:5n3)	Weighted mode	9	-0.879577315	1.074229741	0.436611193	-2.985067607	1.225912978	0.414958271	0.050536087	3.40727543
Myeloproliferative neoplasms myo-inositol	Weighted mode	3	0.614925258	3.327594235	0.87043117	-5.907159443	7.137009958	1.849518357	0.002719902	1257.662298
Myeloproliferative neoplasms 1,5-anhydroglucitol (1,5-AG)	Weighted mode	5	-1.492711325	1.232872229	0.292632499	-3.909140893	0.923718244	0.224762425	0.020057725	2.518637912
Myeloproliferative neoplasms X-08402	Weighted mode	3	-0.583240299	1.008821643	0.621592428	-2.560530719	1.394050121	0.558087065	0.077263724	4.031143656
Myeloproliferative neoplasms 2-hydroxyisobutyrate	Weighted mode	3	-0.110583882	2.084685292	0.962517286	-4.196567055	3.975399292	0.895311227	0.015047144	53.27138347
Myeloproliferative neoplasms 4-methyl-2-oxopentanoate	Weighted mode	3	4.104815447	3.484977073	0.360024437	-2.725739615	10.93537051	60.63155377	0.065497742	56126.90177
Myeloproliferative neoplasms palmitoylcarnitine	Weighted mode	6	-2.837376827	1.919670731	0.199444004	-6.59993146	0.925177805	0.058579128	0.001360461	2.522316702
Myeloproliferative neoplasms X-10510	Weighted mode	4	-0.960239312	1.408457028	0.544303153	-3.720815086	1.800336462	0.382801266	0.024214223	6.051683283
Myeloproliferative neoplasms N-acetylglycine	Weighted mode	3	-0.779535383	0.61406562	0.33200285	-1.983103999	0.424033233	0.458619044	0.137641335	1.528112377
Myeloproliferative neoplasms androsterone sulfate	Weighted mode	9	0.436249233	0.161830016	0.027253871	0.119062402	0.753436063	1.546894283	1.126440209	2.124286674
Myeloproliferative neoplasms 3-carboxy-4-methyl-5-propyl-2-furanpropanoate (CMP	Weighted mode	5	0.046174062	0.88190409	0.960754482	-1.682357954	1.774706077	1.047256682	0.185935032	5.898547165
Myeloproliferative neoplasms acetylcarnitine	Weighted mode	12	-3.321750403	1.885541805	0.105851076	-7.017412342	0.373911535	0.036089605	0.000896141	1.45340857
Myeloproliferative neoplasms serine	Weighted mode	3	-1.952617216	2.722213563	0.547656198	-7.2881558	3.382921367	0.141902196	0.000683588	29.45669938
Myeloproliferative neoplasms hexanoylcarnitine	Weighted mode	17	-0.448678697	0.589085964	0.457348176	-1.603287187	0.705929794	0.638471209	0.201233936	2.02572932
Myeloproliferative neoplasms butyrylcarnitine	Weighted mode	12	0.518157749	0.414498215	0.237209533	-0.294258753	1.33057425	1.678931784	0.745083674	3.783215274
Myeloproliferative neoplasms dehydroisoandrosterone sulfate (DHEA-S)	Weighted mode	7	1.836411558	0.824281917	0.067461231	0.220819	3.452004116	6.27398399	1.247097685	31.56358605
Myeloproliferative neoplasms propionylcarnitine	Weighted mode	14	-0.793352791	1.666491817	0.641931581	-4.059676753	2.47297117	0.452325696	0.017254596	11.85762559
Myeloproliferative neoplasms 10-undecenoate (11:1n1)	Weighted mode	3	0.450234401	0.863680349	0.654136075	-1.242579083	2.143047885	1.568679842	0.288638834	8.525382453
Myeloproliferative neoplasms docosapentaenoate (n3 DPA; 22:5n3)	Weighted mode	8	-1.328269703	1.381973799	0.368498132	-4.036938348	1.380398942	0.264935282	0.017651432	3.976487698
Myeloproliferative neoplasms X-11204	Weighted mode	3	3.533397217	3.926904526	0.463193343	-4.163335654	11.23013009	34.24009128	0.015555583	75367.39914
Myeloproliferative neoplasms X-02269	Weighted mode	13	-0.327203389	0.693593085	0.645566399	-1.686645834	1.032239057	0.720937098	0.185139472	2.807344608
Myeloproliferative neoplasms X-11247	Weighted mode	3	-0.419733167	1.18780891	0.757584343	-2.74783863	1.908372296	0.657222165	0.064066182	6.742105707
Myeloproliferative neoplasms X-11261	Weighted mode	5	-0.708660396	1.230044129	0.595394154	-3.119546888	1.702226096	0.492303247	0.044177181	5.486146499
Myeloproliferative neoplasms X-02249	Weighted mode	3	-0.461324091	1.771514164	0.818905312	-3.933491853	3.01084367	0.630448321	0.019575199	20.30452301
Myeloproliferative neoplasms X-11315	Weighted mode	4	4.101866132	2.664013901	0.221260473	-1.119601115	9.323333378	60.45299565	0.326409969	11196.24103
Myeloproliferative neoplasms X-11317	Weighted mode	3	1.587071313	2.981159014	0.647694933	-4.256000354	7.430142981	4.889408394	0.0141789	1686.048629
Myeloproliferative neoplasms 1-linoleoylglycerophosphoethanolamine*	Weighted mode	12	0.728701619	0.820250001	0.393343093	-0.878988383	2.336391621	2.072388111	0.415202725	10.34384463
Myeloproliferative neoplasms 3-dehydrocarnitine*	Weighted mode	13	-2.203772938	1.212076293	0.094069794	-4.579442473	0.171896596	0.110385893	0.010260615	1.187555029
Myeloproliferative neoplasms pyroglutamine*	Weighted mode	5	-2.613294783	1.313079952	0.11742351	-5.186931489	-0.039658077	0.073292662	0.005589131	0.961118011
Myeloproliferative neoplasms X-11381	Weighted mode	4	-1.244862663	2.427280817	0.64342307	-6.002333064	3.512607738	0.287980456	0.002472976	33.53560593
Myeloproliferative neoplasms X-03056	Weighted mode	3	1.536523802	2.173504786	0.552873979	-2.72354558	5.796593183	4.648403381	0.065641604	329.1762044
Myeloproliferative neoplasms X-11440	Weighted mode	6	-0.625839335	0.334625262	0.120371876	-1.281704849	0.03002618	0.534812354	0.277563693	1.030481511
Myeloproliferative neoplasms X-11445	Weighted mode	9	0.270459564	0.563053202	0.643842106	-0.833124712	1.37404384	1.310566602	0.434688884	3.951296845
Myeloproliferative neoplasms X-11469	Weighted mode	10	0.151414359	0.746818918	0.843843113	-1.31235072	1.615179438	1.163478656	0.26918653	5.028790196
Myeloproliferative neoplasms X-11491	Weighted mode	9	0.200224552	0.39495469	0.625861069	-0.57388664	0.974335745	1.221677058	0.563331711	2.649406744
Myeloproliferative neoplasms X-11529	Weighted mode	14	0.051393048	0.105243253	0.633452317	-0.154883727	0.257669823	1.052736588	0.856514761	1.293911528
Myeloproliferative neoplasms X-11538	Weighted mode	15	0.080944705	0.299455701	0.790868706	-0.505988468	0.667877878	1.084310938	0.602909324	1.950094588
Myeloproliferative neoplasms X-11593	Weighted mode	6	-0.115960963	1.199882134	0.926763619	-2.467729947	2.23580802	0.890509985	0.084777089	9.354037052
Myeloproliferative neoplasms adrenate (22:4n6)	Weighted mode	9	-0.962710312	1.062562897	0.391392667	-3.04533359	1.119912967	0.381856532	0.047580437	3.064587471
Myeloproliferative neoplasms X-11787	Weighted mode	6	0.491020596	1.299725236	0.721094331	-2.056440868	3.038482059	1.633983005	0.127908404	20.87353438
Myeloproliferative neoplasms X-11792	Weighted mode	4	0.144787874	0.432724872	0.759957748	-0.703352874	0.992928623	1.155794371	0.494923104	2.699127635
Myeloproliferative neoplasms X-11793	Weighted mode	3	0.382003579	0.494857477	0.520881238	-0.587917076	1.351924234	1.46521733	0.55548311	3.864855267
Myeloproliferative neoplasms 1-arachidonoylglycerophosphocholine*	Weighted mode	13	-0.547108616	0.537616691	0.328921064	-1.60083733	0.506620098	0.578620408	0.201727535	1.659672175
Myeloproliferative neoplasms X-11905	Weighted mode	11	0.222289049	0.386727329	0.57813124	-0.535696516	0.980274614	1.248932329	0.585261504	2.66518804
Myeloproliferative neoplasms X-12038	Weighted mode	4	1.477473365	3.606917578	0.709575621	-5.592085089	8.547031819	4.381860321	0.003727248	5151.441301
Myeloproliferative neoplasms X-12063	Weighted mode	20	0.146772234	0.222850329	0.51804632	-0.290014412	0.583558879	1.158090159	0.748252784	1.792406049
Myeloproliferative neoplasms isobutyrylcarnitine	Weighted mode	5	-1.164976025	0.995677625	0.306944425	-3.116504171	0.78655212	0.31193014	0.044311804	2.195812461
Myeloproliferative neoplasms erythronate*	Weighted mode	3	6.521295516	3.84875156	0.232273615	-1.022257542	14.06484857	679.4580641	0.3597818	1283175.694
Myeloproliferative neoplasms X-12092	Weighted mode	6	-0.335160735	0.14733364	0.07200118	-0.623934669	-0.046386801	0.715223116	0.535831963	0.954672623
Myeloproliferative neoplasms X-12093	Weighted mode	5	-0.140485439	0.545558303	0.809491657	-1.209779713	0.928808834	0.868936317	0.298262976	2.531491954
Myeloproliferative neoplasms X-12100	Weighted mode	4	1.593004848	3.509516376	0.680719246	-5.28564725	8.471656945	4.918506107	0.005063754	4777.424847
Myeloproliferative neoplasms X-12244	Weighted mode	3	0.760125397	2.315064738	0.773844709	-3.777401489	5.297652283	2.138544372	0.022882074	199.8670276
Myeloproliferative neoplasms 1-eicosatrienoylglycerophosphocholine*	Weighted mode	11	1.180380639	1.50409081	0.450773716	-1.767637347	4.128398626	3.255613182	0.170735902	62.07843253
Myeloproliferative neoplasms 1-eicosadienoylglycerophosphocholine*	Weighted mode	3	2.043189503	2.320738691	0.47150352	-2.505458331	6.591837337	7.715177582	0.081638172	729.1192781
Myeloproliferative neoplasms X-12442	Weighted mode	3	2.080667248	1.135304775	0.208305575	-0.144530111	4.305864606	8.009811659	0.865428853	74.13328385
Myeloproliferative neoplasms X-12456	Weighted mode	12	0.226266461	0.435716988	0.613841922	-0.627738836	1.080271757	1.253909738	0.533797441	2.945479897
Myeloproliferative neoplasms octanoylcarnitine	Weighted mode	7	-0.436888673	0.626578079	0.511737735	-1.664981708	0.791204362	0.64604335	0.189194119	2.206051711
Myeloproliferative neoplasms alpha-hydroxyisovalerate	Weighted mode	3	2.267815359	1.533767711	0.277335924	-0.738369354	5.274000073	9.658277884	0.477892554	195.1951979
Myeloproliferative neoplasms decanoylcarnitine	Weighted mode	7	-0.552773095	0.776635445	0.503338261	-2.074978567	0.969432378	0.57535209	0.125559119	2.636447528
Myeloproliferative neoplasms stearidonate (18:4n3)	Weighted mode	9	-0.999570212	1.120793287	0.398507575	-3.196325055	1.197184631	0.368037585	0.040912278	3.310782713
Myeloproliferative neoplasms epiandrosterone sulfate	Weighted mode	5	0.612164702	0.246508524	0.067967354	0.129007996	1.095321409	1.8444197	1.137699221	2.990143589
Myeloproliferative neoplasms X-12510	Weighted mode	9	0.153824017	0.394229017	0.706579982	-0.618864857	0.92651289	1.166285622	0.538555428	2.525686458
Myeloproliferative neoplasms 1-arachidonoylglycerophosphoinositol*	Weighted mode	17	0.302205321	0.849049182	0.72653974	-1.361931074	1.966341717	1.352838965	0.256165624	7.14449205

Myeloproliferative neoplasms	X-12644	Weighted mode	7	0.917000645	1.787727877	0.626323625	-2.586945994	4.420947283	2.501775412	0.075249502	83.17503835
Myeloproliferative neoplasms	X-12696	Weighted mode	3	-2.003527822	1.634985845	0.345143276	-5.208100079	1.201044434	0.134858686	0.00547206	3.323586378
Myeloproliferative neoplasms	X-12728	Weighted mode	3	-0.069142075	0.092106953	0.531151354	-0.249671704	0.111387553	0.933194087	0.779056502	1.117828041
Myeloproliferative neoplasms	isovalerylcarnitine	Weighted mode	11	-1.461799936	0.869737825	0.123732214	-3.166486073	0.242886201	0.23181864	0.042151455	1.274923531
Myeloproliferative neoplasms	stearoylcarnitine	Weighted mode	6	-2.711214326	1.762476827	0.184590045	-6.165668907	0.743240254	0.066456058	0.002100313	2.102737893
Myeloproliferative neoplasms	1-stearoylglycerophosphoethanolamine	Weighted mode	3	1.209542587	1.663597162	0.542773958	-2.051107851	4.470193025	3.351951075	0.128592363	87.37358666
Myeloproliferative neoplasms	bradykinin, des-arg(9)	Weighted mode	6	0.057027703	0.231969711	0.815577936	-0.397632931	0.511688338	1.058685139	0.671908619	1.668105144
Myeloproliferative neoplasms	X-12798	Weighted mode	10	-1.046256707	0.403095469	0.028948478	-1.836323826	-0.256189587	0.351250124	0.159402341	0.773995215
Myeloproliferative neoplasms	X-12844	Weighted mode	3	3.306072218	2.590218376	0.329998843	-1.770755798	8.382900234	27.27777364	0.1702043	4371.669423
Myeloproliferative neoplasms	X-12850	Weighted mode	6	-0.162692047	0.801547869	0.847157961	-1.733725871	1.408341778	0.849852863	0.1766251	4.089169027
Myeloproliferative neoplasms	laurylcarnitine	Weighted mode	3	0.543579714	1.608800128	0.767623612	-2.609668536	3.696827964	1.722160683	0.073558922	40.31920732
Myeloproliferative neoplasms	X-12855	Weighted mode	6	1.17941302	1.925059724	0.566889552	-2.593704039	4.952530079	3.25246451	0.074742677	141.5325999
Myeloproliferative neoplasms	X-13215	Weighted mode	3	1.809315292	3.674446467	0.67117886	-5.392599783	9.011230368	6.106264995	0.004550129	8194.597447
Myeloproliferative neoplasms	oleoylcarnitine	Weighted mode	6	-2.605932338	1.767959847	0.200489857	-6.071133637	0.859268962	0.073834267	0.002308555	2.361433766
Myeloproliferative neoplasms	1-arachidonoylglycerophosphoethanolamine*	Weighted mode	18	-0.661230881	0.867402037	0.45632132	-2.361338874	1.038877112	0.516215543	0.094293891	2.826041904
Myeloproliferative neoplasms	X-13429	Weighted mode	16	0.073348769	0.245867679	0.769546935	-0.408551882	0.555249421	1.076105784	0.66461199	1.742375515
Myeloproliferative neoplasms	X-13431	Weighted mode	4	0.282894543	0.524597084	0.627157705	-0.745315741	1.311104827	1.326965217	0.47458443	3.710270657
Myeloproliferative neoplasms	X-13435	Weighted mode	3	-2.153435094	1.691879878	0.331031223	-5.469519655	1.162649466	0.11608471	0.004213255	3.198396102
Myeloproliferative neoplasms	X-13477	Weighted mode	3	0.753218701	2.288523308	0.773328508	-3.732286983	5.238724385	2.123824985	0.023938027	188.4295855
Myeloproliferative neoplasms	glutaroyl carnitine	Weighted mode	11	0.552984371	1.533676675	0.725927243	-2.453021912	3.558990653	1.738433414	0.086033209	35.12772318
Myeloproliferative neoplasms	2-tetradecenoyl carnitine	Weighted mode	4	-2.307611931	1.648187733	0.255983002	-5.538059887	0.922836025	0.099498578	0.003934152	2.516416902
Myeloproliferative neoplasms	1-palmitoylglycerophosphoethanolamine	Weighted mode	6	1.402517035	1.603658879	0.421820479	-1.740654367	4.545688437	4.065419904	0.175405583	94.22527308
Myeloproliferative neoplasms	tetradecanedioate	Weighted mode	16	0.133857685	0.308922686	0.670959752	-0.471630778	0.739346149	1.14323011	0.623983859	2.094565533
Myeloproliferative neoplasms	hexadecanedioate	Weighted mode	15	0.103179888	0.382377965	0.791222002	-0.646280923	0.852640698	1.108690831	0.52399092	2.345833318
Myeloproliferative neoplasms	dihomo-linolenate (20:3n3 or n6)	Weighted mode	10	1.47775561	1.652160121	0.394377653	-1.760478227	4.715989446	4.383097253	0.171962607	111.7192968
Myeloproliferative neoplasms	X-14189	Weighted mode	3	0.462649385	0.757484496	0.603516036	-1.022020226	1.947318996	1.588276371	0.359867192	7.009868882
Myeloproliferative neoplasms	X-14205	Weighted mode	3	0.816909249	0.96807038	0.487593087	-1.080508695	2.714327193	2.263493121	0.339422819	15.094451
Myeloproliferative neoplasms	X-14208	Weighted mode	3	0.452826695	0.790448387	0.624551925	-1.096452143	2.002105532	1.572751597	0.33405416	7.404630386
Myeloproliferative neoplasms	X-14304	Weighted mode	3	0.504841552	0.951860474	0.64885137	-1.360804976	2.370488081	1.656722996	0.256454254	10.70261475
Myeloproliferative neoplasms	X-14626	Weighted mode	10	0.345167554	0.644020034	0.604978408	-0.917111712	1.60744682	1.412226524	0.399671743	4.99005444
Myeloproliferative neoplasms	X-14662	Weighted mode	6	0.364121797	0.852205856	0.686957825	-1.30620168	2.034445274	1.439249499	0.270846868	7.648008396
Myeloproliferative neoplasms	octadecanedioate	Weighted mode	10	0.585392043	1.32805144	0.669762827	-2.017588778	3.188372864	1.795694837	0.132975713	24.24893901
Myeloproliferative neoplasms	succinylcarnitine	Weighted mode	9	-2.099289146	1.430582303	0.180438294	-4.90323046	0.704652168	0.122543508	0.007422566	2.023142849
Myeloproliferative neoplasms	5alpha-androstan-3beta,17beta-diol disulfate	Weighted mode	10	0.844959309	0.369867337	0.048206953	0.120019329	1.569899289	2.327883088	1.127518645	4.806164136
Myeloproliferative neoplasms	4-androsten-3beta,17beta-diol disulfate 1*	Weighted mode	5	-0.581567744	0.291699502	0.116952138	-1.153298768	-0.00983672	0.559021277	0.315593979	0.990211502
Myeloproliferative neoplasms	4-androsten-3beta,17beta-diol disulfate 2*	Weighted mode	11	0.392818103	0.819840846	0.642144608	-1.214069956	1.999706162	1.481148949	0.296986096	7.386885233
Myeloproliferative neoplasms	cis-4-decenoyl carnitine	Weighted mode	6	-0.531866965	0.798743541	0.534972243	-2.097404304	1.033670375	0.58750709	0.122774701	2.811365687

Supplementary Table 9.Five Mendelian randomization models estimate the causal effects of gut microbiota on blood metabolites

Notations: Green back means P-value < 0.05 in inverse variance weighted method

Outcome	Exposure	Method	nsnp	Beta	Standard error	P-value	lo_ci	up_ci	OR	OR_lci95	OR_uci95
4-androsten-3beta,17beta-diol disulf	family.Desulfovibrionaceae.id.3169	Inverse variance weighted	6	0.022949591	0.049881938	0.645460108	-0.074819007	0.120718189	1.023214959	0.927911416	1.128306899
4-androsten-3beta,17beta-diol disulf	genus..Eubacteriumhalliigroup.id.11338	Inverse variance weighted	7	-0.014146094	0.047755821	0.767064236	-0.107747503	0.079455315	0.985953492	0.897854273	1.082697178
4-androsten-3beta,17beta-diol disulf	genus.Collinsella.id.815	Inverse variance weighted	3	-0.076996098	0.076423552	0.313698526	-0.22678626	0.072794065	0.925893467	0.797091134	1.075509029
4-androsten-3beta,17beta-diol disulf	genus.Streptococcus.id.1853	Inverse variance weighted	5	0.007804486	0.06011165	0.896698424	-0.110014349	0.125623321	1.00783502	0.895821281	1.133854988
4-androsten-3beta,17beta-diol disulf	family.Lachnospiraceae.id.1987	Inverse variance weighted	6	0.037545865	0.055060129	0.495298015	-0.070371988	0.145463719	1.038259616	0.932047045	1.156575772
4-androsten-3beta,17beta-diol disulf	genus..Eubacteriumxylanophilumgroup.id.14375	Inverse variance weighted	4	-0.004296942	0.059692971	0.942614599	-0.121295164	0.112701281	0.995712277	0.885772472	1.119297527
4-androsten-3beta,17beta-diol disulf	genus.Romboutsia.id.11347	Inverse variance weighted	4	-0.07535923	0.067861	0.266786237	-0.20836679	0.057648331	0.927410273	0.811909182	1.059342392
Oleoylcarnitine	family.Desulfovibrionaceae.id.3169	Inverse variance weighted	6	0.011990381	0.027491777	0.662732061	-0.041893501	0.065874263	1.012062554	0.958971904	1.06809241
Oleoylcarnitine	genus..Eubacteriumhalliigroup.id.11338	Inverse variance weighted	7	0.050986138	0.03697692	0.167936745	-0.021488626	0.123460901	1.052308306	0.978740609	1.131405767
Oleoylcarnitine	genus.Collinsella.id.815	Inverse variance weighted	3	-0.020116121	0.042734768	0.637840513	-0.103876266	0.063644024	0.980084858	0.901336816	1.065712963
Oleoylcarnitine	genus.Streptococcus.id.1853	Inverse variance weighted	5	0.031326525	0.033286995	0.346651547	-0.033915985	0.096569035	1.031822365	0.966652715	1.101385613
Oleoylcarnitine	family.Lachnospiraceae.id.1987	Inverse variance weighted	6	0.026979048	0.046626697	0.562846928	-0.064409278	0.118367374	1.027346278	0.937621173	1.125657573
Oleoylcarnitine	genus..Eubacteriumxylanophilumgroup.id.14375	Inverse variance weighted	4	-0.010554696	0.033509738	0.752781394	-0.076233782	0.05512439	0.989500809	0.926599559	1.056672046
Oleoylcarnitine	genus.Romboutsia.id.11347	Inverse variance weighted	4	0.049418852	0.040449179	0.221801545	-0.029861538	0.128699242	1.05066033	0.970579913	1.137348007
Stearoylcarnitine	family.Desulfovibrionaceae.id.3169	Inverse variance weighted	6	-0.01028209	0.024696897	0.677167619	-0.058688009	0.038123828	0.98977059	0.943000931	1.038859865
Stearoylcarnitine	genus..Eubacteriumhalliigroup.id.11338	Inverse variance weighted	7	0.046035643	0.024076553	0.05586941	-0.001154401	0.093225688	1.047111733	0.998846265	1.097709447
Stearoylcarnitine	genus.Collinsella.id.815	Inverse variance weighted	3	-0.002563773	0.075716598	0.972988692	-0.150968306	0.145840759	0.99743951	0.859874951	1.15701193
Stearoylcarnitine	genus.Streptococcus.id.1853	Inverse variance weighted	5	0.014731886	0.030520309	0.62931498	-0.04508792	0.074551692	1.014840935	0.955913434	1.077401035
Stearoylcarnitine	family.Lachnospiraceae.id.1987	Inverse variance weighted	6	0.020479289	0.028397144	0.470802305	-0.035179113	0.076137691	1.020690429	0.96543248	1.079111148
Stearoylcarnitine	genus..Eubacteriumxylanophilumgroup.id.14375	Inverse variance weighted	4	0.027212346	0.029801298	0.361176185	-0.031198199	0.085622891	1.027585983	0.969283443	1.08939543
Stearoylcarnitine	genus.Romboutsia.id.11347	Inverse variance weighted	4	0.014586293	0.034455583	0.672050034	-0.052946649	0.082119235	1.014693192	0.948430611	1.085585242
Succinylcarnitine	family.Desulfovibrionaceae.id.3169	Inverse variance weighted	6	0.015396225	0.016185112	0.341473174	-0.016326594	0.047119043	1.015515357	0.983805963	1.048246789
Succinylcarnitine	genus..Eubacteriumhalliigroup.id.11338	Inverse variance weighted	7	-0.013032421	0.015377011	0.396701837	-0.043171362	0.01710652	0.987052133	0.957747255	1.017253675
Succinylcarnitine	genus.Collinsella.id.815	Inverse variance weighted	3	-0.029357834	0.027268308	0.281646312	-0.082803717	0.024088049	0.97106892	0.920531814	1.024380509
Succinylcarnitine	genus.Streptococcus.id.1853	Inverse variance weighted	5	0.021148057	0.021561972	0.32668967	-0.021113408	0.063409522	1.021373262	0.979107919	1.065463081
Succinylcarnitine	family.Lachnospiraceae.id.1987	Inverse variance weighted	6	0.007407415	0.019408815	0.702719435	-0.030633862	0.045448692	1.007434918	0.9698306	1.046497309
Succinylcarnitine	genus..Eubacteriumxylanophilumgroup.id.14375	Inverse variance weighted	4	0.045962998	0.019506372	0.018457661	0.00773051	0.084195486	1.047035668	1.007760467	1.087841531
Succinylcarnitine	genus.Romboutsia.id.11347	Inverse variance weighted	4	0.032667777	0.026673876	0.220684304	-0.01961302	0.084948574	1.033207227	0.980578064	1.088661079
X_12798	family.Desulfovibrionaceae.id.3169	Inverse variance weighted	6	-0.018469005	0.025467676	0.468333047	-0.068385651	0.031447641	0.981700502	0.933900245	1.031947342
X_12798	genus..Eubacteriumhalliigroup.id.11338	Inverse variance weighted	7	-0.037069946	0.024764352	0.134417306	-0.085608075	0.011468183	0.963608733	0.91795393	1.011534195
X_12798	genus.Collinsella.id.815	Inverse variance weighted	3	-0.037939217	0.039720796	0.339503138	-0.115791977	0.039913543	0.962771459	0.890660482	1.040720793
X_12798	genus.Streptococcus.id.1853	Inverse variance weighted	5	-0.012461278	0.030758544	0.68538031	-0.072748023	0.047825468	0.987616043	0.929835098	1.048987557
X_12798	family.Lachnospiraceae.id.1987	Inverse variance weighted	6	-0.03351855	0.029354009	0.253506819	-0.091052406	0.024015307	0.967036973	0.912969864	1.024305997
X_12798	genus..Eubacteriumxylanophilumgroup.id.14375	Inverse variance weighted	4	-0.042068227	0.032740492	0.19882757	-0.106239592	0.022103138	0.958804362	0.899209179	1.022349222
X_12798	genus.Romboutsia.id.11347	Inverse variance weighted	4	-0.01137008	0.034480635	0.741586871	-0.078952124	0.056211963	0.988694315	0.924084165	1.057821879
X_12850	family.Desulfovibrionaceae.id.3169	Inverse variance weighted	6	0.008044662	0.041595473	0.846643871	-0.073482465	0.08957179	1.008077108	0.929152438	1.093705848
X_12850	genus..Eubacteriumhalliigroup.id.11338	Inverse variance weighted	7	0.040410747	0.045171359	0.370995632	-0.048125116	0.12894661	1.041238372	0.953014542	1.137629384
X_12850	genus.Collinsella.id.815	Inverse variance weighted	3	-0.154806894	0.126148189	0.219753874	-0.402057344	0.092443556	0.856580572	0.668942384	1.09685123
X_12850	genus.Streptococcus.id.1853	Inverse variance weighted	5	0.0288249	0.077536814	0.710073337	-0.123147256	0.180797056	1.029244358	0.884133459	1.198171993
X_12850	family.Lachnospiraceae.id.1987	Inverse variance weighted	6	-0.04410351	0.065537717	0.500980042	-0.172557434	0.084350415	0.956854909	0.841509956	1.088010082
X_12850	genus..Eubacteriumxylanophilumgroup.id.14375	Inverse variance weighted	4	-0.034136507	0.050572749	0.499677012	-0.133259095	0.064986082	0.96643957	0.875238293	1.067144172
X_12850	genus.Romboutsia.id.11347	Inverse variance weighted	4	0.022247188	0.056620932	0.694382603	-0.08872984	0.133224215	1.022496502	0.915092762	1.142506137
Epiandrosterone sulfate	family.Desulfovibrionaceae.id.3169	Inverse variance weighted	6	-0.01815361	0.044939827	0.686246765	-0.106235671	0.069928452	0.982010175	0.899212705	1.072431448
Epiandrosterone sulfate	genus..Eubacteriumhalliigroup.id.11338	Inverse variance weighted	7	-0.016767434	0.043176897	0.697762411	-0.101394152	0.067859283	0.983372357	0.903576816	1.070214701
Epiandrosterone sulfate	genus.Collinsella.id.815	Inverse variance weighted	3	-0.043621053	0.069456995	0.529984507	-0.179756762	0.092514656	0.957316661	0.835473405	1.096929218
Epiandrosterone sulfate	genus.Streptococcus.id.1853	Inverse variance weighted	5	0.001512153	0.054867003	0.978012816	-0.106027173	0.109051479	1.001513297	0.899400209	1.115219759
Epiandrosterone sulfate	family.Lachnospiraceae.id.1987	Inverse variance weighted	6	0.075057086	0.064211478	0.242442105	-0.05079741	0.200911583	1.077945685	0.950471207	1.222516676
Epiandrosterone sulfate	genus..Eubacteriumxylanophilumgroup.id.14375	Inverse variance weighted	4	-0.051717387	0.072485667	0.475546114	-0.193789295	0.090354521	0.949597198	0.823831472	1.09456226
Epiandrosterone sulfate	genus.Romboutsia.id.11347	Inverse variance weighted	4	-0.077930854	0.06159262	0.205777114	-0.198652389	0.04279068	0.925028386	0.819834827	1.043719401
X_12442	family.Desulfovibrionaceae.id.3169	Inverse variance weighted	6	0.029501556	0.03917033	0.45135331	-0.047272291	0.106275402	1.029941038	0.953827644	1.112128117
X_12442	genus..Eubacteriumhalliigroup.id.11338	Inverse variance weighted	7	0.041629921	0.030414798	0.17108086	-0.017983082	0.101242925	1.042508597	0.982177648	1.106545416
X_12442	genus.Collinsella.id.815	Inverse variance weighted	3	0.05871506	0.048945151	0.230291094	-0.037217435	0.154647556	1.060473027	0.963466621	1.1672465
X_12442	genus.Streptococcus.id.1853	Inverse variance weighted	5	0.03685508	0.03735626	0.323846722	-0.036363189	0.110073349	1.037542649	0.96429001	1.116359952
X_12442	family.Lachnospiraceae.id.1987	Inverse variance weighted	6	0.073980821	0.05481112	0.177099047	-0.033448974	0.181410617	1.076786154	0.967104257	1.19890737
X_12442	genus..Eubacteriumxylanophilumgroup.id.14375	Inverse variance weighted	4	-0.008077607	0.049483911	0.870331812	-0.105066072	0.088910858	0.99195493	0.900265039	1.092983222
X_12442	genus.Romboutsia.id.11347	Inverse variance weighted	4	0.023714933	0.043702124	0.587371195	-0.06194123	0.109371097	1.023998368	0.939938125	1.11557626
X_12092	family.Desulfovibrionaceae.id.3169	Inverse variance weighted	6	-0.016582911	0.038634123	0.667756112	-0.092305793	0.059139971	0.983553829	0.911826277	1.060923729
X_12092	genus..Eubacteriumhalliigroup.id.11338	Inverse variance weighted	7	-0.043827442	0.042068131	0.297494892	-0.126280978	0.038626094	0.957119101	0.881367167	1.03938178
X_12092	genus.Collinsella.id.815	Inverse variance weighted	3	0.01962164	0.05864301	0.73793085	-0.09531866	0.13456194	1.01981541	0.9090832	1.144035518
X_12092	genus.Streptococcus.id.1853	Inverse variance weighted	5	0.003810574	0.085928044	0.964628521	-0.164608391	0.17222954	1.003817844	0.848225812	1.187950484
X_12092	family.Lachnospiraceae.id.1987	Inverse variance weighted	6	-0.027458871	0.043069831	0.523771183	-0.111875739	0.056957997	0.972914697	0.89415536	1.058611345
X_12092	genus..Eubacteriumxylanophilumgroup.id.14375	Inverse variance weighted	4	0.034385244	0.055857344	0.538164895	-0.07509515	0.143865637	1.034983251	0.927655216	1.154728946
X_12092	genus.Romboutsia.id.11347	Inverse variance weighted	4	0.047038445	0.052399293	0.36934958	-0.055664169	0.149741059	1.048162305	0.945856731	1.161533436
X_11440	family.Desulfovibrionaceae.id.3169	Inverse variance weighted	6	0.026837918	0.038609125	0.486981035	-0.048835968	0.102511803	1.027201298	0.952337331	1.107950379
X_11440	genus..Eubacteriumhalliigroup.id.11338	Inverse variance weighted	7	-0.032319431	0.036891762	0.380996909	-0.104627284	0.039988422	0.96819726	0.90066015	1.040798724
X_11440	genus.Collinsella.id.815	Inverse variance weighted	3	-0.016283334	0.059339948	0.783771394	-0.132589632	0.100022965	0.983848523	0.875824428	1.105196299
X_11440	genus.Streptococcus.id.1853	Inverse variance weighted	5	-0.000437294	0.047108716	0.992593611	-0.092770379	0.09189579	0.999562801	0.911402754	1.096250576
X_11440	family.Lachnospiraceae.id.1987	Inverse variance weighted	6	0.052948066	0.042213117	0.209731666	-0.029789643	0.135685775	1.054374885	0.970649695	1.145321948
X_11440	genus..Eubacteriumxylanophilumgroup.id.14375	Inverse variance weighted	4	0.04820861	0.063609189	0.448518327	-0.0764654	0.172882619	1.049389545	0.926384967	1.188726564
X_11440	genus.Romboutsia.id.11347	Inverse variance weighted	4	-0.019552484	0.053422468	0.714367018	-0.124260522	0.085155553	0.980637426	0.883149731	1.088886433
X_11423	family.Desulfovibrionaceae.id.3169	Inverse variance weighted	6	-0.012423606	0.025730876	0.629217339	-0.062856123	0.03800891	0.987653248	0.939078576	1.038740488
X_11423	genus..Eubacteriumhalliigroup.id.11338	Inverse variance weighted	7	0.008262889	0.016022213	0.606053764	-0.023140648	0.039666426	1.008297121	0.977125043	1.040463645
X_11423	genus.Collinsella.id.815	Inverse variance weighted	3	0.026567117	0.020004408	0.184157244	-0.012641522	0.065775756	1.026923169	0.987438046	1.0679872
X_11423	genus.Streptococcus.id.1853	Inverse variance weighted	5	-0.021335534	0.01645362	0.19473181	-0.05358463	0.010913561	0.978890458	0.947825723	1.010973331
X_11423	family.Lachnospiraceae.id.1987	Inverse variance weighted	6	0.01996189	0.014854863	0.179014703	-0.009153642	0.049077422	1.020162461	0.990888125	1.050301664
X_11423	genus..Eubacteriumxylanophilumgroup.id.14375	Inverse variance weighted	4	-0.002654419	0.017681511	0.88066679	-0.037310181	0.032001343	0.997349101	0.963377268	1.032518892
X_11423	genus.Romboutsia.id.11347	Inverse variance weighted	4	0.012399464	0.019187017	0.518122405	-0.025207091	0.050006018	1.012476656	0.975107955	1.051277423
3-dehydrocarnitine	family.Desulfovibrionaceae.id.3169	Inverse variance weighted	6	0.010782304	0.018196863	0.553491444	-0.024883548	0.046448156	1.010840643	0.975423496	1.047543769
3-dehydrocarnitine	genus..Eubacteriumhalliigroup.id.11338	Inverse variance weighted	7	-0.026321376	0.019957977	0.187222646	-0.065439011	0.012796259	0.974022012	0.93665617	1.012878481
3-dehydrocarnitine	genus.Collinsella.id.815	Inverse variance weighted	3	-0.040454592	0.027597803	0.142685671	-0.094546286	0.013637101	0.960352771	0.909785623	1.013730511
3-dehydrocarnitine	genus.Streptococcus.id.1853	Inverse variance weighted	5	-0.001128787	0.023428179	0.961572204	-0.047048018	0.044790444	0.99887185	0.954041585	1.045808682
3-dehydrocarnitine	family.Lachnospiraceae.id.1987	Inverse variance weighted	6	-0.037841339	0.021044524	0.072152274	-0.079088605	0.003405928	0.962865698	0.923958053	1.003411734
3-dehydrocarnitine	genus..Eubacteriumxylanophilumgroup.id.14375	Inverse variance weighted	4	-0.034687134	0.030724264	0.258905515	-0.094906691	0.025532423	0.965907568	0.909457791	1.025861167
3-dehydrocarnitine	genus.Romboutsia.id.11347	Inverse variance weighted	4	0.015089031	0.027336097	0.580960754	-0.038489719	0.068667781	1.015203445	0.962241597	1.071080317
Propionylcarnitine	family.Desulfovibrionaceae.id.3169	Inverse variance weighted	6	0.011198662	0.016500836	0.497345446	-0.021142976	0.0435403	1.011261602	0.97907897	1.044502087
Propionylcarnitine	genus..Eubacteriumhalliigroup.id.11338	Inverse variance weighted	7	0.002215262	0.01552295	0.886520125	-0.02820972	0.032640245	1.002217718	0.972184459	1.033178781
Propionylcarnitine	genus.Collinsella.id.815	Inverse variance weighted	3	0.033448317	0.024073605	0.164705455	-0.013735948	0.080632582	1.034014002	0.98635796	1.083972553
Propionylcarnitine	genus.Streptococcus.id.1853	Inverse variance weighted	5	-0.005041749	0.021419277	0.813910923	-0.047023532	0.036940034	0.994970939	0.954064946	1.037630797
Propionylcarnitine	family.Lachnospiraceae.id.1987	Inverse variance weighted	6	-0.006467895	0.034222227	0.850095231	-0.07354346	0.060607669	0.993552976	0.929095767	1.062481988
Propionylcarnitine	genus..Eubacteriumxylanophilumgroup.id.14375	Inverse variance weighted	4	-0.003718592	0.021226716	0.860934639	-0.045322955	0.03788577	0.996288313	0.955688788	1.038612586
Propionylcarnitine	genus.Romboutsia.id.11347	Inverse variance weighted	4	0.01504152	0.02383633	0.52801869	-0.031677687	0.061760728	1.015155213	0.968818795	1.063707799
Acetylcarnitine	family.Desulfovibrionaceae.id.3169	Inverse variance weighted	6	0.022420045	0.018624508	0.228669752	-0.014083991	0.05892408	1.022673263	0.986014725	1.06069471

Acetylcarnitine	genus..Eubacteriumhalliigroup.id.11338	Inverse variance weighted	7	0.01932546	0.017852999	0.279040641	-0.015666417	0.054317338	1.019513406	0.984455662	1.0558196
Acetylcarnitine	genus.Collinsella.id.815	Inverse variance weighted	3	0.006345748	0.027775285	0.81928287	-0.048093811	0.060785307	1.006365925	0.953044377	1.062670742
Acetylcarnitine	genus.Streptococcus.id.1853	Inverse variance weighted	5	0.038356344	0.021419277	0.073335117	-0.003625439	0.080338128	1.039101445	0.996381125	1.083653419
Acetylcarnitine	family.Lachnospiraceae.id.1987	Inverse variance weighted	6	0.025077846	0.025198103	0.319625627	-0.024310437	0.074466128	1.02539494	0.975982682	1.077308853
Acetylcarnitine	genus..Eubacteriumxylanophilumgroup.id.14375	Inverse variance weighted	4	-0.011608864	0.026769696	0.664537584	-0.064077469	0.04085974	0.988458258	0.937932336	1.041705986
Acetylcarnitine	genus.Romboutsia.id.11347	Inverse variance weighted	4	0.007689492	0.025004789	0.758447013	-0.041319894	0.056698879	1.007719132	0.959522135	1.058337075
Palmitoylcarnitine	family.Desulfovibrionaceae.id.3169	Inverse variance weighted	6	0.012899306	0.03190098	0.685952031	-0.049626615	0.075425226	1.012982861	0.951584666	1.078342593
Palmitoylcarnitine	genus..Eubacteriumhalliigroup.id.11338	Inverse variance weighted	7	0.031909991	0.036878989	0.386894949	-0.040372827	0.10419281	1.032424574	0.960431298	1.109814417
Palmitoylcarnitine	genus.Collinsella.id.815	Inverse variance weighted	3	-0.018220417	0.040591406	0.653522738	-0.097779573	0.06133874	0.981944572	0.906848775	1.063259022
Palmitoylcarnitine	genus.Streptococcus.id.1853	Inverse variance weighted	5	0.012413442	0.028977821	0.668376402	-0.044383086	0.069209971	1.012490809	0.956587432	1.071661204
Palmitoylcarnitine	family.Lachnospiraceae.id.1987	Inverse variance weighted	6	0.008064376	0.034350816	0.814390987	-0.059263223	0.075391975	1.008096981	0.942458659	1.078306737
Palmitoylcarnitine	genus..Eubacteriumxylanophilumgroup.id.14375	Inverse variance weighted	4	-0.021662107	0.029796191	0.467220266	-0.080062641	0.036738426	0.978570831	0.923058523	1.037421623
Palmitoylcarnitine	genus.Romboutsia.id.11347	Inverse variance weighted	4	0.031424624	0.036528093	0.389631146	-0.040170438	0.103019687	1.031923591	0.960625698	1.108513232
Betaine	family.Desulfovibrionaceae.id.3169	Inverse variance weighted	6	0.012197243	0.023645284	0.605964403	-0.034147514	0.058542001	1.012271933	0.966428932	1.060289518
Betaine	genus..Eubacteriumhalliigroup.id.11338	Inverse variance weighted	7	0.02988117	0.016458956	0.069447709	-0.002378384	0.062140724	1.030332092	0.997624442	1.06411208
Betaine	genus.Collinsella.id.815	Inverse variance weighted	3	0.000541174	0.02516025	0.982839547	-0.048772916	0.049855265	1.000541321	0.952397379	1.051118951
Betaine	genus.Streptococcus.id.1853	Inverse variance weighted	5	-0.002044097	0.021723983	0.925034477	-0.044623103	0.040534909	0.997957991	0.956357863	1.041367662
Betaine	family.Lachnospiraceae.id.1987	Inverse variance weighted	6	-0.02306359	0.023368641	0.323669028	-0.068866126	0.022738945	0.977200341	0.933451636	1.022999446
Betaine	genus..Eubacteriumxylanophilumgroup.id.14375	Inverse variance weighted	4	-0.021856751	0.020406933	0.284149045	-0.06185434	0.018140838	0.978380377	0.9400198	1.018306383
Betaine	genus.Romboutsia.id.11347	Inverse variance weighted	4	0.031835208	0.023555236	0.176531141	-0.014333053	0.07800347	1.032347369	0.985769176	1.08112641
Lysine	family.Desulfovibrionaceae.id.3169	Inverse variance weighted	6	0.005062312	0.011794194	0.667762631	-0.018054308	0.028178932	1.005075147	0.982107695	1.028579714
Lysine	genus..Eubacteriumhalliigroup.id.11338	Inverse variance weighted	7	0.002149294	0.011507548	0.851838948	-0.020405499	0.024704088	1.002151606	0.979801284	1.025011763
Lysine	genus.Collinsella.id.815	Inverse variance weighted	3	0.00270661	0.026196673	0.917709917	-0.04863887	0.05405209	1.002710276	0.952525053	1.055539584
Lysine	genus.Streptococcus.id.1853	Inverse variance weighted	5	0.009954847	0.014638345	0.496471064	-0.01873631	0.038646003	1.010004561	0.981438124	1.039402473
Lysine	family.Lachnospiraceae.id.1987	Inverse variance weighted	6	6.52E-05	0.01392815	0.996265566	-0.027233985	0.027364364	1.000065192	0.973133517	1.027742207
Lysine	genus..Eubacteriumxylanophilumgroup.id.14375	Inverse variance weighted	4	-0.02869583	0.014619143	0.049658471	-0.057349349	-4.23E-05	0.971711985	0.944264134	0.99995769
Lysine	genus.Romboutsia.id.11347	Inverse variance weighted	4	0.004274455	0.022473925	0.849155399	-0.039774437	0.048323347	1.004283603	0.961006182	1.049509956
4-androsten-3beta,17beta-diol disulf	family.Desulfovibrionaceae.id.3169	MR Egger	6	0.15817738	0.303929428	0.63023499	-0.437524299	0.753879059	1.171373955	0.645632838	2.125227932
4-androsten-3beta,17beta-diol disulf	genus..Eubacteriumhalliigroup.id.11338	MR Egger	7	-0.062565893	0.1275694	0.644581823	-0.312601916	0.187470131	0.939351164	0.731541069	1.206194221
4-androsten-3beta,17beta-diol disulf	genus.Collinsella.id.815	MR Egger	3	-0.234113286	0.296278746	0.574277168	-0.814819628	0.346593057	0.791272171	0.442719174	1.414241093
4-androsten-3beta,17beta-diol disulf	genus.Streptococcus.id.1853	MR Egger	5	-0.13217462	0.324154609	0.710809344	-0.767517654	0.503168414	0.876187982	0.464163855	1.653953386
4-androsten-3beta,17beta-diol disulf	family.Lachnospiraceae.id.1987	MR Egger	6	1.060397955	0.558195008	0.130285303	-0.033664261	2.154460171	2.887519864	0.966896075	8.623233854
4-androsten-3beta,17beta-diol disulf	genus..Eubacteriumxylanophilumgroup.id.14375	MR Egger	4	0.220604796	0.178398994	0.341753479	-0.129057231	0.570266824	1.246830581	0.878923662	1.76873893
4-androsten-3beta,17beta-diol disulf	genus.Romboutsia.id.11347	MR Egger	4	0.422373355	0.467893754	0.461954978	-0.494698403	1.339445112	1.525578	0.60975478	3.816924953
Oleoylcarnitine	family.Desulfovibrionaceae.id.3169	MR Egger	6	0.137438397	0.166743582	0.456108794	-0.189379024	0.464255818	1.147331025	0.827472816	1.590829881
Oleoylcarnitine	genus..Eubacteriumhalliigroup.id.11338	MR Egger	7	-0.015191326	0.104935633	0.890548557	-0.220865165	0.190482514	0.984923481	0.801824787	1.209833219
Oleoylcarnitine	genus.Collinsella.id.815	MR Egger	3	-0.154449699	0.166612292	0.524105147	-0.481009792	0.172110393	0.856886593	0.618158865	1.187808952
Oleoylcarnitine	genus.Streptococcus.id.1853	MR Egger	5	0.148561462	0.178454826	0.466208202	-0.201209997	0.498332922	1.160164102	0.817740691	1.645975013
Oleoylcarnitine	family.Lachnospiraceae.id.1987	MR Egger	6	0.379281923	0.520938709	0.506900794	-0.641757945	1.400321792	1.461234934	0.526366287	4.056505109
Oleoylcarnitine	genus..Eubacteriumxylanophilumgroup.id.14375	MR Egger	4	0.032141585	0.118472161	0.811596895	-0.200063851	0.26434702	1.032663704	0.818678478	1.302580139
Oleoylcarnitine	genus.Romboutsia.id.11347	MR Egger	4	-0.190621129	0.290671966	0.579313286	-0.760338182	0.379095924	0.826445645	0.467508297	1.46096317
Stearoylcarnitine	family.Desulfovibrionaceae.id.3169	MR Egger	6	0.009247378	0.15492933	0.955267407	-0.294414108	0.312908864	1.009290267	0.74496793	1.367396907
Stearoylcarnitine	genus..Eubacteriumhalliigroup.id.11338	MR Egger	7	0.072531966	0.063957289	0.308193107	-0.05282432	0.197888252	1.075227177	0.948546639	1.218826185
Stearoylcarnitine	genus.Collinsella.id.815	MR Egger	3	-0.093681883	0.402794587	0.854521442	-0.883159275	0.695795508	0.910572384	0.413474566	2.005303675
Stearoylcarnitine	genus.Streptococcus.id.1853	MR Egger	5	0.218923596	0.165920733	0.278681001	-0.106281041	0.544128232	1.24473617	0.899171908	1.723105579
Stearoylcarnitine	family.Lachnospiraceae.id.1987	MR Egger	6	0.462831104	0.277831515	0.171068736	-0.081718665	1.007380873	1.588565018	0.921531181	2.738419346
Stearoylcarnitine	genus..Eubacteriumxylanophilumgroup.id.14375	MR Egger	4	-0.021226965	0.089240889	0.834136271	-0.196139108	0.153685177	0.978996741	0.821897895	1.166123706
Stearoylcarnitine	genus.Romboutsia.id.11347	MR Egger	4	-0.084275201	0.240681628	0.759662186	-0.556011191	0.387460789	0.919178263	0.573492058	1.473235186
Succinylcarnitine	family.Desulfovibrionaceae.id.3169	MR Egger	6	0.01480069	0.10228559	0.891946061	-0.185679066	0.215280446	1.014910763	0.830540101	1.24020966
Succinylcarnitine	genus..Eubacteriumhalliigroup.id.11338	MR Egger	7	-0.009608015	0.044004858	0.835796056	-0.095857538	0.076641507	0.990437994	0.908593447	1.079654958
Succinylcarnitine	genus.Collinsella.id.815	MR Egger	3	0.091312264	0.096395842	0.517236949	-0.097623585	0.280248114	1.095611072	0.906990244	1.32345814
Succinylcarnitine	genus.Streptococcus.id.1853	MR Egger	5	-0.009858969	0.113906814	0.936480169	-0.233116325	0.213398386	0.990189471	0.792061432	1.237877706
Succinylcarnitine	family.Lachnospiraceae.id.1987	MR Egger	6	0.112833594	0.20786429	0.616082541	-0.294580415	0.520247603	1.119445635	0.744844047	1.682444177
Succinylcarnitine	genus..Eubacteriumxylanophilumgroup.id.14375	MR Egger	4	0.126086331	0.059748288	0.169287903	0.008979687	0.243192975	1.134380096	1.009020126	1.275314704
Succinylcarnitine	genus.Romboutsia.id.11347	MR Egger	4	-0.179398839	0.183986397	0.432367222	-0.540012177	0.181214498	0.835772494	0.582741156	1.198672265
X_12798	family.Desulfovibrionaceae.id.3169	MR Egger	6	-0.258056147	0.159342736	0.180652434	-0.570367909	0.054255615	0.772551855	0.565317415	1.055754435
X_12798	genus..Eubacteriumhalliigroup.id.11338	MR Egger	7	-0.038405242	0.066295772	0.587504573	-0.168344956	0.091534472	0.962322888	0.845062275	1.095854552
X_12798	genus.Collinsella.id.815	MR Egger	3	-0.004709612	0.152001603	0.980281305	-0.302632755	0.293213531	0.995301461	0.738870393	1.340729047
X_12798	genus.Streptococcus.id.1853	MR Egger	5	0.044666533	0.177647809	0.817718567	-0.303523173	0.392856238	1.045679102	0.738212783	1.481205433
X_12798	family.Lachnospiraceae.id.1987	MR Egger	6	0.046589697	0.282763814	0.8771199	-0.507627378	0.600806772	1.047692049	0.601922019	1.823589428
X_12798	genus..Eubacteriumxylanophilumgroup.id.14375	MR Egger	4	-0.180468733	0.092544894	0.190471447	-0.361856725	0.000919258	0.834878784	0.696382135	1.000919681
X_12798	genus.Romboutsia.id.11347	MR Egger	4	0.051397056	0.239206112	0.849791181	-0.417446924	0.520241036	1.052740808	0.658726454	1.682433128
X_12850	family.Desulfovibrionaceae.id.3169	MR Egger	6	-0.031353111	0.254027371	0.9077245	-0.529246758	0.466540536	0.969133301	0.589048499	1.594468634
X_12850	genus..Eubacteriumhalliigroup.id.11338	MR Egger	7	-0.082997215	0.119947023	0.519780686	-0.318093379	0.152098949	0.92035371	0.727534849	1.164275435
X_12850	genus.Collinsella.id.815	MR Egger	3	-0.173770707	0.671635573	0.838823201	-1.490176431	1.142635016	0.840489594	0.225332896	3.135018316
X_12850	genus.Streptococcus.id.1853	MR Egger	5	0.200389605	0.485169268	0.707330642	-0.750542161	1.151321371	1.221878716	0.472110524	3.162368812
X_12850	family.Lachnospiraceae.id.1987	MR Egger	6	0.530337815	0.67880913	0.478294701	-0.800128079	1.86080371	1.699506331	0.449271418	6.428901666
X_12850	genus..Eubacteriumxylanophilumgroup.id.14375	MR Egger	4	0.163279549	0.151718622	0.394418395	-0.134088951	0.460648049	1.177365775	0.874512272	1.585100875
X_12850	genus.Romboutsia.id.11347	MR Egger	4	0.084362672	0.409195394	0.855742621	-0.7176603	0.886385645	1.088023418	0.487892444	2.426344114
Epiandrosterone sulfate	family.Desulfovibrionaceae.id.3169	MR Egger	6	0.135782438	0.279185155	0.652161123	-0.411420465	0.682985342	1.145432664	0.662708227	1.979779237
Epiandrosterone sulfate	genus..Eubacteriumhalliigroup.id.11338	MR Egger	7	0.01322476	0.117084245	0.914464377	-0.216260361	0.242709881	1.013312594	0.805525547	1.274698756
Epiandrosterone sulfate	genus.Collinsella.id.815	MR Egger	3	0.093947868	0.352547928	0.834204806	-0.597046071	0.784941807	1.098502477	0.550435183	2.192279362
Epiandrosterone sulfate	genus.Streptococcus.id.1853	MR Egger	5	0.135686591	0.291328321	0.673123127	-0.435316918	0.706690101	1.145322883	0.64705957	2.027270081
Epiandrosterone sulfate	family.Lachnospiraceae.id.1987	MR Egger	6	-0.472776857	0.667248412	0.517714324	-1.780583745	0.83503003	0.623269134	0.168539734	2.304883264
Epiandrosterone sulfate	genus..Eubacteriumxylanophilumgroup.id.14375	MR Egger	4	0.297509609	0.163012303	0.209538977	-0.021994505	0.617013723	1.346501314	0.97824561	1.853385048
Epiandrosterone sulfate	genus.Romboutsia.id.11347	MR Egger	4	0.500505287	0.419341436	0.355031825	-0.321403928	1.322414502	1.649554558	0.725130291	3.752470795
X_12442	family.Desulfovibrionaceae.id.3169	MR Egger	6	0.071054278	0.265752401	0.802403822	-0.449820429	0.591928985	1.073639499	0.637742662	1.80747164
X_12442	genus..Eubacteriumhalliigroup.id.11338	MR Egger	7	0.023484341	0.081809083	0.785580346	-0.136861463	0.183830144	1.023762269	0.872091035	1.201811671
X_12442	genus.Collinsella.id.815	MR Egger	3	0.148578446	0.188378437	0.574848172	-0.22064329	0.517800181	1.160183806	0.802002712	1.678331561
X_12442	genus.Streptococcus.id.1853	MR Egger	5	0.144045458	0.207122974	0.536798261	-0.26191557	0.550006486	1.154936609	0.769575996	1.733264261
X_12442	family.Lachnospiraceae.id.1987	MR Egger	6	0.493532089	0.567988579	0.433923953	-0.619725525	1.606789704	1.638091901	0.53809211	4.986776474
X_12442	genus..Eubacteriumxylanophilumgroup.id.14375	MR Egger	4	0.119314135	0.153640818	0.518670995	-0.181821868	0.420450138	1.126723806	0.833749845	1.522646802
X_12442	genus.Romboutsia.id.11347	MR Egger	4	-0.421116545	0.302996854	0.299066564	-1.014990379	0.172757288	0.656313607	0.362405917	1.188577588
X_12092	family.Desulfovibrionaceae.id.3169	MR Egger	6	0.125160907	0.256400515	0.650996606	-0.377384102	0.627705916	1.133330799	0.685652662	1.87330812
X_12092	genus..Eubacteriumhalliigroup.id.11338	MR Egger	7	-0.151882943	0.110887126	0.229087818	-0.36922171	0.065455823	0.859088837	0.691272132	1.067645571
X_12092	genus.Collinsella.id.815	MR Egger	3	0.157383746	0.22207965	0.6075056	-0.277892368	0.59265986	1.170444681	0.757378335	1.808793159
X_12092	genus.Streptococcus.id.1853	MR Egger	5	-0.68258866	0.358135008	0.152733135	-1.384533274	0.019355955	0.505307229	0.25044066	1.019544496
X_12092	family.Lachnospiraceae.id.1987	MR Egger	6	0.006800401	0.478127922	0.98933322	-0.930330326	0.943931127	1.006823576	0.3944234	2.570064838
X_12092	genus..Eubacteriumxylanophilumgroup.id.14375	MR Egger	4	-0.141772324	0.154026525	0.454510908	-0.443664313	0.160119665	0.867818815	0.641680788	1.173651308
X_12092	genus.Romboutsia.id.11347	MR Egger	4	0.045290018	0.364993435	0.912594826	-0.670097115	0.760677151	1.046331271	0.511658886	2.139724646
X_11440	family.Desulfovibrionaceae.id.3169	MR Egger	6	0.05513319	0.236970793	0.827446588	-0.409329564	0.519595943	1.056681344	0.664095335	1.681348152
X_11440	genus..Eubacteriumhalliigroup.id.11338	MR Egger	7	0.03730119	0.098805491	0.721280339	-0.156357573	0.230959954	1.038005611	0.85525332	1.259808788
X_11440	genus.Collinsella.id.815	MR Egger	3	-0.062714285	0.229916795	0.830473669	-0.513351203	0.387922632	0.939211782	0.598486565	1.473915746
X_11440	genus.Streptococcus.id.1853	MR Egger	5	-0.019463711	0.255290375	0.944026777	-0.519832846	0.480905424	0.980724484	0.594619933	1.617538298
X_11440	family.Lachnospiraceae.id.1987	MR Egger	6	0.520834613	0.420479548	0.283190254	-0.303305301	1.344974527	1.683432078	0.738373636	3.838088774
X_11440	genus..Eubacteriumxylanophilumgroup.id.14375	MR Egger	4	0.350289427	0.140083972	0.129563835	0.075724842	0.624854013	1.419478325	1.07866573	1.867973237
X_11440	genus.Romboutsia.id.11347	MR Egger	4	0.080658383	0.36470017	0.845491722	-0.63415395	0.795470715	1.08400052	0.53038403	2.215483614
X_11423	family.Desulfovibrionaceae.id.3169	MR Egger	6	-0.040179894	0.176362921	0.830954042	-0.38585122	0.305491431	0.960616614	0.67987167	1.357291855
X_11423	genus..Eubacteriumhalliigroup.id.11338	MR Egger	7	-0.035731605	0.044085936	0.454485192	-0.12214004	0.05067683	0.964899233	0.885024421	1.051982869
X_11423	genus.Collinsella.id.815	MR Egger	3	-0.030242149	0.075178952	0.756517937	-0.177592895	0.117108597	0.97021057	0.837283216	1.124241512
X_11423	genus.Streptococcus.id.1853	MR Egger	5	-0.064770679	0.092750004	0.535231376	-0.246560687	0.117019329	0.937282377	0.781483934	1.124141158
X_11423	family.Lachnospiraceae.id.1987	MR Egger	6	-0.280879601	0.15607292	0.146294711	-0.586782525	0.025023323	0.755119245	0.556113691	1.025339034
X_11423	genus..Eubacteriumxylanophilumgroup.id.14375	MR Egger	4	0.013712801	0.053995356	0.823248695	-0.092118097	0.119543699	1.013807252	0.911997439	1.126982491
X_11423	genus.Romboutsia.id.11347	MR Egger	4	-0.118439464	0.134626	0.471779686	-0.382306424	0.145427495	0.888305588	0.682285953	1.156533877
3-dehydrocarnitine	family.Desulfovibrionaceae.id.3169	MR Egger	6	0.010989852	0.110668224	0.925674246	-0.205919866	0.227899571	1.011050462	0.813898294	1.255959184
3-dehydrocarnitine	genus..Eubacteriumhalliigroup.id.11338	MR Egger	7	-0.02856414	0.057838008	0.642329786	-0.141926635	0.084798356	0.971839959	0.867684912	1.088497555
3-dehydrocarnitine	genus.Collinsella.id.815	MR Egger	3	-0.086712157	0.103484075	0.555993683	-0.289540944	0.116116631	0.916940993	0.748607141	1.123126856
3-dehydrocarnitine	genus.Streptococcus.id.1853	MR Egger	5	-0.201096818	0.120778001	0.194499593	-0.437821699	0.035628064	0.817833247	0.645440856	1.036270348
3-dehydrocarnitine	family.Lachnospiraceae.id.1987	MR Egger	6	0.01075101	0.199569572	0.959621169	-0.380405351	0.40190737	1.01080901	0.683584262	1.494672875
3-dehydrocarnitine	genus..Eubacteriumxylanophilumgroup.id.14375	MR Egger	4	-0.11608614	0.091777186	0.333345578	-0.295969425	0.063797145	0.890398521	0.743810169	1.065876158
3-dehydrocarnitine	genus.Romboutsia.id.11347	MR Egger	4	0.031614275	0.231870107	0.904034657	-0.422851134	0.486079685	1.032119314	0.655176159	1.625929553
Propionylcarnitine	family.Desulfovibrionaceae.id.3169	MR Egger	6	0.020814597	0.118206704	0.868781384	-0.210870542	0.252499736	1.021032731	0.809878905	1.287239156
Propionylcarnitine	genus..Eubacteriumhalliigroup.id.11338	MR Egger	7	-0.018881675	0.043776473	0.684199151	-0.104683562	0.066920213	0.981295467	0.900609464	1.069210165
Propionylcarnitine	genus.Collinsella.id.815	MR Egger	3	0.040117575	0.094350595	0.744055064	-0.144809591	0.225044741	1.040933155	0.865187017	1.252378748
Propionylcarnitine	genus.Streptococcus.id.1853	MR Egger	5	-0.036070068	0.113377619	0.77123828	-0.2582902	0.186150065	0.964572706	0.772371058	1.204603015
Propionylcarnitine	family.Lachnospiraceae.id.1987	MR Egger	6	-0.44787243	0.325379581	0.240705763	-1.085616409	0.189871549	0.638986194	0.337693564	1.209094278
Propionylcarnitine	genus..Eubacteriumxylanophilumgroup.id.14375	MR Egger	4	0.04875021	0.061166375	0.50903005	-0.071135884	0.168636305	1.049958049	0.93133533	1.183689559
Propionylcarnitine	genus.Romboutsia.id.11347	MR Egger	4	0.224120562	0.169436794	0.31690774	-0.107975554	0.556216678	1.25122186	0.89764954	1.744061655
Acetylcarnitine	family.Desulfovibrionaceae.id.3169	MR Egger	6	0.11204896	0.11355843	0.379645742	-0.110525562	0.334623482	1.118567625	0.895363443	1.397414135
Acetylcarnitine	genus..Eubacteriumhalliigroup.id.11338	MR Egger	7	0.008071813	0.047619038	0.872041654	-0.0852615	0.101405127	1.008104478	0.918272125	1.106724915
Acetylcarnitine	genus.Collinsella.id.815	MR Egger	3	0.023586461	0.103516838	0.857380094	-0.179306541	0.226479463	1.023866821	0.835849638	1.254176853
Acetylcarnitine	genus.Streptococcus.id.1853	MR Egger	5	0.051098464	0.113377619	0.68279203	-0.171121669	0.273318596	1.052426514	0.842719035	1.314318915
Acetylcarnitine	family.Lachnospiraceae.id.1987	MR Egger	6	0.287773364	0.233978535	0.286120595	-0.170824565	0.746371293	1.333455061	0.842969447	2.109331965
Acetylcarnitine	genus..Eubacteriumxylanophilumgroup.id.14375	MR Egger	4	0.04376328	0.088852294	0.67109857	-0.130387217	0.217913777	1.044735016	0.877755483	1.243479847
Acetylcarnitine	genus.Romboutsia.id.11347	MR Egger	4	-0.036349313	0.169449069	0.850030672	-0.368469487	0.295770862	0.964303391	0.691792318	1.344162123
Palmitoylcarnitine	family.Desulfovibrionaceae.id.3169	MR Egger	6	-0.063008334	0.209171719	0.77825061	-0.472984902	0.346968235	0.938935649	0.623139479	1.414771784
Palmitoylcarnitine	genus..Eubacteriumhalliigroup.id.11338	MR Egger	7	0.046823289	0.107510288	0.681341978	-0.163896877	0.257543454	1.04793681	0.848829551	1.293748028
Palmitoylcarnitine	genus.Collinsella.id.815	MR Egger	3	-0.1912243	0.148697445	0.420765594	-0.482671293	0.100222693	0.825947307	0.617132646	1.105417059
Palmitoylcarnitine	genus.Streptococcus.id.1853	MR Egger	5	0.135444067	0.154695649	0.445722663	-0.167759405	0.43864754	1.145045149	0.845557247	1.550608663

Palmitoylcarnitine	family.Lachnospiraceae.id.1987	MR Egger	6	0.404697478	0.316126412	0.269682286	-0.214910289	1.024305246	1.498848997	0.806613799	2.785159788
Palmitoylcarnitine	genus..Eubacteriumxylanophilumgroup.id.14375	MR Egger	4	0.059037599	0.08797864	0.571311069	-0.113400536	0.231475734	1.060815125	0.892792992	1.260458739
Palmitoylcarnitine	genus.Romboutsia.id.11347	MR Egger	4	-0.27575319	0.233587247	0.359173996	-0.733584194	0.182077814	0.759000237	0.480184826	1.199707545
Betaine	family.Desulfovibrionaceae.id.3169	MR Egger	6	-0.09682827	0.151597432	0.557744316	-0.393959237	0.200302696	0.907711874	0.674381545	1.221772528
Betaine	genus..Eubacteriumhalliigroup.id.11338	MR Egger	7	-0.016957875	0.043250776	0.711152152	-0.101729397	0.067813646	0.9831851	0.903273947	1.07016586
Betaine	genus.Collinsella.id.815	MR Egger	3	0.134921053	0.092030254	0.381089702	-0.045458244	0.31530035	1.14444643	0.955559502	1.37064054
Betaine	genus.Streptococcus.id.1853	MR Egger	5	0.066786385	0.124226726	0.628162444	-0.176697999	0.310270768	1.069067085	0.838032833	1.363794335
Betaine	family.Lachnospiraceae.id.1987	MR Egger	6	0.06797634	0.258242675	0.805378933	-0.438179304	0.574131984	1.070339984	0.645210084	1.775588618
Betaine	genus..Eubacteriumxylanophilumgroup.id.14375	MR Egger	4	-0.067242712	0.063363596	0.399797394	-0.19143536	0.056949935	0.934968246	0.825773002	1.05860281
Betaine	genus.Romboutsia.id.11347	MR Egger	4	0.094909206	0.158719331	0.610554651	-0.216180684	0.405999095	1.099559017	0.805589732	1.500801194
Lysine	family.Desulfovibrionaceae.id.3169	MR Egger	6	-0.084630196	0.073848651	0.315695568	-0.229373553	0.06011316	0.918852017	0.795031492	1.061956711
Lysine	genus..Eubacteriumhalliigroup.id.11338	MR Egger	7	-0.041953438	0.031351747	0.238474134	-0.103402863	0.019495987	0.958914428	0.901763613	1.019687275
Lysine	genus.Collinsella.id.815	MR Egger	3	0.068294749	0.123721187	0.678900782	-0.174198777	0.310788275	1.070680844	0.840129882	1.364500292
Lysine	genus.Streptococcus.id.1853	MR Egger	5	-0.101676925	0.077006895	0.278411981	-0.25261044	0.04925659	0.903321345	0.776770422	1.050489861
Lysine	family.Lachnospiraceae.id.1987	MR Egger	6	-0.027998118	0.128927223	0.838708988	-0.280695476	0.224699239	0.972390196	0.755258295	1.251946123
Lysine	genus..Eubacteriumxylanophilumgroup.id.14375	MR Egger	4	-0.031098196	0.042899877	0.543850746	-0.115181954	0.052985562	0.969380379	0.891203971	1.054414421
Lysine	genus.Romboutsia.id.11347	MR Egger	4	-0.058723886	0.197091115	0.793841235	-0.445022472	0.327574701	0.9429671	0.640809876	1.387598702
4-androsten-3beta,17beta-diol disulf	family.Desulfovibrionaceae.id.3169	Simple mode	6	0.056966635	0.092464492	0.564796878	-0.12426377	0.23819704	1.058620489	0.883146863	1.268959204
4-androsten-3beta,17beta-diol disulf	genus..Eubacteriumhalliigroup.id.11338	Simple mode	7	0.03069605	0.101625976	0.772810763	-0.168490863	0.229882964	1.031172032	0.844938983	1.258452717
4-androsten-3beta,17beta-diol disulf	genus.Collinsella.id.815	Simple mode	3	-0.06183042	0.103356951	0.610414666	-0.264410043	0.140749204	0.940042286	0.767658702	1.151135911
4-androsten-3beta,17beta-diol disulf	genus.Streptococcus.id.1853	Simple mode	5	0.028057878	0.092484287	0.776725122	-0.153211325	0.209327081	1.028455207	0.857948396	1.232848174
4-androsten-3beta,17beta-diol disulf	family.Lachnospiraceae.id.1987	Simple mode	6	-0.00909062	0.103881952	0.93366346	-0.212699246	0.194518006	0.990950575	0.80839923	1.214725355
4-androsten-3beta,17beta-diol disulf	genus..Eubacteriumxylanophilumgroup.id.14375	Simple mode	4	0.000736396	0.106361702	0.994910545	-0.207732539	0.209205332	1.000736668	0.812424299	1.232698085
4-androsten-3beta,17beta-diol disulf	genus.Romboutsia.id.11347	Simple mode	4	-0.078735689	0.102748922	0.499279883	-0.280123577	0.122652198	0.92428419	0.75569035	1.130491166
Oleoylcarnitine	family.Desulfovibrionaceae.id.3169	Simple mode	6	-0.018894345	0.051782385	0.73012632	-0.120387821	0.08259913	0.981283034	0.886576537	1.086106334
Oleoylcarnitine	genus..Eubacteriumhalliigroup.id.11338	Simple mode	7	0.025685557	0.062529814	0.695506077	-0.096872878	0.148243993	1.026018274	0.907671384	1.159795844
Oleoylcarnitine	genus.Collinsella.id.815	Simple mode	3	-0.049784314	0.067388443	0.536982899	-0.181865663	0.082297035	0.951434614	0.833713332	1.085778276
Oleoylcarnitine	genus.Streptococcus.id.1853	Simple mode	5	0.003183955	0.057011581	0.958141564	-0.108558744	0.114926653	1.003189029	0.897126192	1.121791155
Oleoylcarnitine	family.Lachnospiraceae.id.1987	Simple mode	6	0.085902423	0.052730753	0.164225623	-0.017449852	0.189254698	1.089699994	0.982701515	1.208348678
Oleoylcarnitine	genus..Eubacteriumxylanophilumgroup.id.14375	Simple mode	4	0.017480243	0.055366393	0.772904942	-0.091037887	0.125998374	1.017633917	0.91298312	1.134280323
Oleoylcarnitine	genus.Romboutsia.id.11347	Simple mode	4	0.015166265	0.069253288	0.84070556	-0.120570178	0.150902709	1.015281857	0.886414878	1.162883514
Stearoylcarnitine	family.Desulfovibrionaceae.id.3169	Simple mode	6	-0.003887668	0.043007287	0.931482419	-0.08818195	0.080406614	0.996119879	0.91559427	1.083727637
Stearoylcarnitine	genus..Eubacteriumhalliigroup.id.11338	Simple mode	7	0.065083781	0.04667654	0.212652957	-0.026402237	0.156569798	1.067248435	0.973943254	1.169492388
Stearoylcarnitine	genus.Collinsella.id.815	Simple mode	3	0.041735822	0.051018938	0.499289034	-0.058261297	0.141732941	1.042619006	0.943403407	1.152268884
Stearoylcarnitine	genus.Streptococcus.id.1853	Simple mode	5	0.014482888	0.049886715	0.786003873	-0.083295074	0.112260849	1.014588273	0.920079616	1.118804662
Stearoylcarnitine	family.Lachnospiraceae.id.1987	Simple mode	6	0.019983047	0.051250511	0.712659627	-0.080467954	0.120434049	1.020184045	0.922684471	1.127986346
Stearoylcarnitine	genus..Eubacteriumxylanophilumgroup.id.14375	Simple mode	4	0.011207291	0.042704711	0.809974234	-0.072493943	0.094908525	1.011270328	0.93007138	1.099558268
Stearoylcarnitine	genus.Romboutsia.id.11347	Simple mode	4	-0.006273871	0.053119396	0.913445585	-0.110387888	0.097840145	0.993745768	0.89548672	1.102786485
Succinylcarnitine	family.Desulfovibrionaceae.id.3169	Simple mode	6	0.024855615	0.03077012	0.455910077	-0.03545382	0.085165051	1.025167091	0.965167304	1.088896775
Succinylcarnitine	genus..Eubacteriumhalliigroup.id.11338	Simple mode	7	-0.016752767	0.025405867	0.53411117	-0.066548266	0.033042732	0.98338678	0.935617756	1.033594706
Succinylcarnitine	genus.Collinsella.id.815	Simple mode	3	-0.067051393	0.043012008	0.25935854	-0.151354929	0.017252144	0.935147141	0.859542568	1.017401822
Succinylcarnitine	genus.Streptococcus.id.1853	Simple mode	5	-0.0089378	0.038525909	0.827927913	-0.084488582	0.066572982	0.991102023	0.919018908	1.068838967
Succinylcarnitine	family.Lachnospiraceae.id.1987	Simple mode	6	0.035234386	0.037392383	0.389309885	-0.038054685	0.108523457	1.035862472	0.962660297	1.114631054
Succinylcarnitine	genus..Eubacteriumxylanophilumgroup.id.14375	Simple mode	4	0.035000789	0.034168473	0.381051953	-0.031969419	0.101970997	1.035620526	0.968536201	1.107351355
Succinylcarnitine	genus.Romboutsia.id.11347	Simple mode	4	0.059536495	0.039710713	0.230762709	-0.018296503	0.137369492	1.061344494	0.981869862	1.147251971
X_12798	family.Desulfovibrionaceae.id.3169	Simple mode	6	-0.05203712	0.04905603	0.337323332	-0.148186938	0.044112698	0.949293629	0.862269909	1.045100129
X_12798	genus..Eubacteriumhalliigroup.id.11338	Simple mode	7	-0.045391526	0.043101971	0.332837451	-0.129871389	0.039088336	0.955623257	0.878208371	1.039862337
X_12798	genus.Collinsella.id.815	Simple mode	3	-0.046580455	0.051448259	0.460824727	-0.147419043	0.054258133	0.954487764	0.862932296	1.055757093
X_12798	genus.Streptococcus.id.1853	Simple mode	5	-0.072656303	0.060247307	0.294283216	-0.190741025	0.045428418	0.929920386	0.826346564	1.046476093
X_12798	family.Lachnospiraceae.id.1987	Simple mode	6	-0.01007993	0.05310317	0.856917334	-0.114162143	0.094002283	0.989970702	0.892113295	1.098562253
X_12798	genus..Eubacteriumxylanophilumgroup.id.14375	Simple mode	4	-0.078859227	0.055891665	0.253086068	-0.18840689	0.030688437	0.924170014	0.828277621	1.031164181
X_12798	genus.Romboutsia.id.11347	Simple mode	4	-0.017758812	0.049691547	0.744469265	-0.115154244	0.07963662	0.982397946	0.891228667	1.082893494
X_12850	family.Desulfovibrionaceae.id.3169	Simple mode	6	-0.011284684	0.069468739	0.877318167	-0.147443412	0.124874045	0.98877875	0.862911267	1.133005736
X_12850	genus..Eubacteriumhalliigroup.id.11338	Simple mode	7	0.061068141	0.085224262	0.500574063	-0.105971412	0.228107694	1.062971344	0.899450361	1.256220605
X_12850	genus.Collinsella.id.815	Simple mode	3	-0.102669584	0.07926052	0.324564187	-0.258020202	0.052681035	0.9024251	0.772579624	1.054093372
X_12850	genus.Streptococcus.id.1853	Simple mode	5	0.020729813	0.102952039	0.850246571	-0.181056183	0.22251581	1.020946168	0.834388479	1.249215569
X_12850	family.Lachnospiraceae.id.1987	Simple mode	6	-0.037480823	0.100675387	0.724932477	-0.234804581	0.159842935	0.963212889	0.790725357	1.173326568
X_12850	genus..Eubacteriumxylanophilumgroup.id.14375	Simple mode	4	0.012424327	0.086325686	0.894684812	-0.156774017	0.181622671	1.01250183	0.854897229	1.199161631
X_12850	genus.Romboutsia.id.11347	Simple mode	4	-0.048740131	0.088386812	0.61974607	-0.221978281	0.12449802	0.952428605	0.800932759	1.132579778
Epiandrosterone sulfate	family.Desulfovibrionaceae.id.3169	Simple mode	6	0.01677194	0.086467651	0.853835244	-0.152704657	0.186248537	1.016913379	0.858383202	1.204721641
Epiandrosterone sulfate	genus..Eubacteriumhalliigroup.id.11338	Simple mode	7	0.012258622	0.078319494	0.880756401	-0.141247585	0.16576483	1.012334067	0.868274313	1.180295499
Epiandrosterone sulfate	genus.Collinsella.id.815	Simple mode	3	-0.081372002	0.092127012	0.47027046	-0.261940945	0.099196942	0.921850698	0.769556468	1.104283758
Epiandrosterone sulfate	genus.Streptococcus.id.1853	Simple mode	5	0.039164946	0.085242178	0.669768669	-0.127909723	0.206239615	1.039942004	0.879932813	1.229047667
Epiandrosterone sulfate	family.Lachnospiraceae.id.1987	Simple mode	6	-0.053927624	0.128625859	0.69242974	-0.306034308	0.19817906	0.947500681	0.736361356	1.219180681
Epiandrosterone sulfate	genus..Eubacteriumxylanophilumgroup.id.14375	Simple mode	4	-0.158784473	0.132013117	0.315326136	-0.417530182	0.099961235	0.853180222	0.658671612	1.105128077
Epiandrosterone sulfate	genus.Romboutsia.id.11347	Simple mode	4	-0.130856256	0.108744956	0.315145986	-0.34399637	0.082283857	0.877343878	0.708931502	1.085763968
X_12442	family.Desulfovibrionaceae.id.3169	Simple mode	6	-0.018558217	0.075570459	0.815772847	-0.166676316	0.129559882	0.981612926	0.846473557	1.138327274
X_12442	genus..Eubacteriumhalliigroup.id.11338	Simple mode	7	0.013952236	0.053571992	0.803232675	-0.091048869	0.118953341	1.014050023	0.912973094	1.126317364
X_12442	genus.Collinsella.id.815	Simple mode	3	0.029862166	0.065172036	0.691774476	-0.097875025	0.157599358	1.030312513	0.906762219	1.17069707
X_12442	genus.Streptococcus.id.1853	Simple mode	5	0.030575048	0.054675559	0.605847744	-0.076589047	0.137739143	1.031047265	0.926270429	1.147676132
X_12442	family.Lachnospiraceae.id.1987	Simple mode	6	0.160273303	0.071948562	0.07639411	0.019254122	0.301292484	1.173831639	1.019440678	1.351604607
X_12442	genus..Eubacteriumxylanophilumgroup.id.14375	Simple mode	4	0.034983309	0.075694204	0.675417533	-0.113377331	0.18334395	1.035602424	0.89281371	1.201227499
X_12442	genus.Romboutsia.id.11347	Simple mode	4	0.020389911	0.080894958	0.817280684	-0.138164206	0.178944029	1.020599206	0.870955664	1.195953803
X_12092	family.Desulfovibrionaceae.id.3169	Simple mode	6	0.02647072	0.082321023	0.760797486	-0.134878486	0.187819926	1.026824182	0.873822087	1.206616216
X_12092	genus..Eubacteriumhalliigroup.id.11338	Simple mode	7	-0.015964097	0.081377581	0.850950332	-0.175464156	0.143535962	0.984162654	0.839067472	1.154343323
X_12092	genus.Collinsella.id.815	Simple mode	3	0.048983023	0.085403565	0.624172245	-0.118407964	0.21637401	1.050202522	0.888333571	1.241566651
X_12092	genus.Streptococcus.id.1853	Simple mode	5	0.086831113	0.072126341	0.295006837	-0.054536516	0.228198741	1.090712457	0.94692393	1.256334986
X_12092	family.Lachnospiraceae.id.1987	Simple mode	6	-0.036251541	0.089533847	0.702297769	-0.211737881	0.139234799	0.964397677	0.80917677	1.149393944
X_12092	genus..Eubacteriumxylanophilumgroup.id.14375	Simple mode	4	0.061818879	0.094999737	0.561617734	-0.124380605	0.248018362	1.063769656	0.883043686	1.281483463
X_12092	genus.Romboutsia.id.11347	Simple mode	4	0.035415914	0.072413052	0.658309521	-0.106513669	0.177345497	1.036050527	0.89896276	1.19404356
X_11440	family.Desulfovibrionaceae.id.3169	Simple mode	6	0.0234485	0.063082644	0.725340302	-0.100193482	0.147090481	1.023725577	0.904662365	1.158458777
X_11440	genus..Eubacteriumhalliigroup.id.11338	Simple mode	7	-0.056999988	0.072433283	0.461265381	-0.198969223	0.084969248	0.944594081	0.819575117	1.088683587
X_11440	genus.Collinsella.id.815	Simple mode	3	0.004418227	0.073652367	0.957620491	-0.139940412	0.148776865	1.004428001	0.86941004	1.160414031
X_11440	genus.Streptococcus.id.1853	Simple mode	5	0.025155291	0.069513087	0.735749926	-0.111090359	0.161400942	1.025474355	0.894857886	1.175156044
X_11440	family.Lachnospiraceae.id.1987	Simple mode	6	0.006316885	0.076100461	0.937066532	-0.142840018	0.155473788	1.006336879	0.866892745	1.168211315
X_11440	genus..Eubacteriumxylanophilumgroup.id.14375	Simple mode	4	-0.035807488	0.085487209	0.703497369	-0.203362418	0.131747442	0.964826016	0.815982461	1.140820159
X_11440	genus.Romboutsia.id.11347	Simple mode	4	-0.035458615	0.077222788	0.677336816	-0.186815278	0.115898049	0.965162677	0.829596967	1.122881387
X_11423	family.Desulfovibrionaceae.id.3169	Simple mode	6	-0.025539737	0.025628185	0.364736405	-0.075770979	0.024691506	0.974783643	0.927028491	1.024998856
X_11423	genus..Eubacteriumhalliigroup.id.11338	Simple mode	7	-0.02459377	0.035142716	0.510244855	-0.093473494	0.044285954	0.975706193	0.910762158	1.045281215
X_11423	genus.Collinsella.id.815	Simple mode	3	0.046299879	0.029190781	0.253604564	-0.010914051	0.10351381	1.047388454	0.989145291	1.109061109
X_11423	genus.Streptococcus.id.1853	Simple mode	5	-0.026246332	0.023616286	0.328707328	-0.072534253	0.020041588	0.974095109	0.93003389	1.020243769
X_11423	family.Lachnospiraceae.id.1987	Simple mode	6	0.021669458	0.025564701	0.435329231	-0.028437355	0.071776272	1.021905946	0.971963181	1.07441494
X_11423	genus..Eubacteriumxylanophilumgroup.id.14375	Simple mode	4	-0.002720531	0.028984936	0.931137636	-0.059531006	0.054089945	0.997283167	0.942206319	1.055579542
X_11423	genus.Romboutsia.id.11347	Simple mode	4	0.022065877	0.031530009	0.534415361	-0.03973294	0.083864695	1.022311129	0.961046062	1.087481742
3-dehydrocarnitine	family.Desulfovibrionaceae.id.3169	Simple mode	6	0.016462623	0.032693515	0.635976948	-0.047616667	0.080541913	1.016598879	0.953499225	1.083874274
3-dehydrocarnitine	genus..Eubacteriumhalliigroup.id.11338	Simple mode	7	-0.012654196	0.036549901	0.740997332	-0.084292001	0.058983609	0.987425531	0.91916282	1.060757854
3-dehydrocarnitine	genus.Collinsella.id.815	Simple mode	3	-0.023124208	0.038218042	0.606647319	-0.098031571	0.051783154	0.977141107	0.906620281	1.053147347
3-dehydrocarnitine	genus.Streptococcus.id.1853	Simple mode	5	0.014338284	0.038791772	0.730400962	-0.061693588	0.090370156	1.01444157	0.940170922	1.094579374
3-dehydrocarnitine	family.Lachnospiraceae.id.1987	Simple mode	6	-0.037165549	0.038833872	0.382499845	-0.113279937	0.03894884	0.963516613	0.892900669	1.03971729
3-dehydrocarnitine	genus..Eubacteriumxylanophilumgroup.id.14375	Simple mode	4	-0.058516292	0.037475378	0.21633849	-0.131968032	0.014935448	0.943162874	0.87636901	1.015047539
3-dehydrocarnitine	genus.Romboutsia.id.11347	Simple mode	4	0.014984168	0.042291843	0.746551669	-0.067907844	0.09787618	1.015096993	0.934346575	1.102826225
Propionylcarnitine	family.Desulfovibrionaceae.id.3169	Simple mode	6	-0.016124639	0.031354006	0.628974006	-0.077578491	0.045329213	0.984004667	0.925354389	1.046372282
Propionylcarnitine	genus..Eubacteriumhalliigroup.id.11338	Simple mode	7	0.017063401	0.029053188	0.578407277	-0.039880848	0.074007649	1.017209812	0.960903926	1.076815042
Propionylcarnitine	genus.Collinsella.id.815	Simple mode	3	0.038535582	0.031340354	0.343872158	-0.022891512	0.099962676	1.039287708	0.977368511	1.10512967
Propionylcarnitine	genus.Streptococcus.id.1853	Simple mode	5	-0.008616719	0.032864249	0.806122928	-0.073030646	0.055797209	0.991420299	0.929572342	1.057383234
Propionylcarnitine	family.Lachnospiraceae.id.1987	Simple mode	6	-0.041540348	0.044316007	0.391603292	-0.128399721	0.045319026	0.959310629	0.879501754	1.046361623
Propionylcarnitine	genus..Eubacteriumxylanophilumgroup.id.14375	Simple mode	4	0.009241541	0.035238391	0.810100077	-0.059825706	0.078308788	1.009284376	0.941928691	1.081456548
Propionylcarnitine	genus.Romboutsia.id.11347	Simple mode	4	-0.002013607	0.033873954	0.956336612	-0.068406557	0.064379344	0.997988419	0.93388072	1.066496891
Acetylcarnitine	family.Desulfovibrionaceae.id.3169	Simple mode	6	0.020245765	0.038767184	0.623806734	-0.055737916	0.096229445	1.0204521	0.945786979	1.101011656
Acetylcarnitine	genus..Eubacteriumhalliigroup.id.11338	Simple mode	7	0.015892109	0.031117107	0.627788601	-0.045097421	0.076881638	1.01601906	0.955904352	1.079914248
Acetylcarnitine	genus.Collinsella.id.815	Simple mode	3	0.014100408	0.03787811	0.745445329	-0.060140687	0.088341502	1.014200287	0.941632049	1.092361102
Acetylcarnitine	genus.Streptococcus.id.1853	Simple mode	5	0.012163313	0.036343957	0.754685697	-0.059070843	0.083397469	1.012237587	0.942639987	1.086973761
Acetylcarnitine	family.Lachnospiraceae.id.1987	Simple mode	6	0.041597546	0.042929034	0.377056689	-0.042543361	0.125738453	1.042474846	0.958348909	1.133985538
Acetylcarnitine	genus..Eubacteriumxylanophilumgroup.id.14375	Simple mode	4	-0.038794298	0.041771254	0.421533192	-0.120665955	0.04307736	0.961948564	0.886329984	1.044018657
Acetylcarnitine	genus.Romboutsia.id.11347	Simple mode	4	0.002912733	0.035136238	0.939153937	-0.065954295	0.07177976	1.002916979	0.936173651	1.074418688
Palmitoylcarnitine	family.Desulfovibrionaceae.id.3169	Simple mode	6	0.063311133	0.060728882	0.344930677	-0.055717475	0.182339742	1.065358256	0.945806312	1.200021823
Palmitoylcarnitine	genus..Eubacteriumhalliigroup.id.11338	Simple mode	7	0.087235767	0.078866102	0.311039369	-0.067341792	0.241813326	1.091153907	0.934875613	1.273556431
Palmitoylcarnitine	genus.Collinsella.id.815	Simple mode	3	-0.067418542	0.064032125	0.402825634	-0.192921506	0.058084423	0.934803865	0.824546693	1.059804464
Palmitoylcarnitine	genus.Streptococcus.id.1853	Simple mode	5	-0.007757937	0.045737081	0.873541593	-0.097402616	0.081886742	0.992272078	0.907190683	1.085332881
Palmitoylcarnitine	family.Lachnospiraceae.id.1987	Simple mode	6	0.045612385	0.06963316	0.541376438	-0.090868609	0.18209338	1.046668628	0.913137681	1.199726219
Palmitoylcarnitine	genus..Eubacteriumxylanophilumgroup.id.14375	Simple mode	4	0.001818114	0.046342893	0.971170375	-0.089013956	0.092650185	1.001819768	0.914832806	1.097077893
Palmitoylcarnitine	genus.Romboutsia.id.11347	Simple mode	4	0.099284646	0.064508072	0.221404398	-0.027151175	0.225720467	1.104380613	0.973214105	1.253225799
Betaine	family.Desulfovibrionaceae.id.3169	Simple mode	6	0.045597212	0.043139231	0.338892035	-0.038955681	0.130150106	1.046652747	0.961793334	1.138999341
Betaine	genus..Eubacteriumhalliigroup.id.11338	Simple mode	7	0.002698558	0.035247969	0.941463228	-0.06638746	0.071784577	1.002702203	0.935768221	1.074423863
Betaine	genus.Collinsella.id.815	Simple mode	3	-0.010640864	0.047702013	0.844192389	-0.104136809	0.082855082	0.98941555	0.90110201	1.08638436
Betaine	genus.Streptococcus.id.1853	Simple mode	5	-0.005480277	0.037245419	0.890140224	-0.078481299	0.067520745	0.994534713	0.924519349	1.069852454
Betaine	family.Lachnospiraceae.id.1987	Simple mode	6	-0.002259176	0.039145689	0.956213445	-0.078984725	0.074466374	0.997743374	0.924054039	1.077309117
Betaine	genus..Eubacteriumxylanophilumgroup.id.14375	Simple mode	4	-0.001722658	0.037719495	0.966443131	-0.075652868	0.072207552	0.998278825	0.92713799	1.074878414
Betaine	genus.Romboutsia.id.11347	Simple mode	4	0.027657874	0.034062365	0.476249687	-0.039104362	0.094420109	1.028043903	0.961650344	1.099021358

Lysine	family.Desulfovibrionaceae.id.3169	Simple mode	6	0.000953763	0.020996999	0.965527875	-0.040200355	0.042107882	1.000954218	0.960596959	1.043006994
Lysine	genus..Eubacteriumhalliigroup.id.11338	Simple mode	7	-0.013854195	0.026021583	0.61357098	-0.064856498	0.037148109	0.986241333	0.937201944	1.037846723
Lysine	genus.Collinsella.id.815	Simple mode	3	0.005764876	0.028245158	0.857158457	-0.049595634	0.061125387	1.005781525	0.951614147	1.063032196
Lysine	genus.Streptococcus.id.1853	Simple mode	5	0.030517119	0.029536576	0.359883904	-0.02737457	0.088408807	1.030987539	0.972996718	1.092434626
Lysine	family.Lachnospiraceae.id.1987	Simple mode	6	-0.004648317	0.027245432	0.871220154	-0.058049364	0.04875273	0.99536247	0.943603366	1.049960695
Lysine	genus..Eubacteriumxylanophilumgroup.id.14375	Simple mode	4	-0.024219329	0.023161628	0.372547201	-0.069616119	0.021177461	0.976071606	0.932751817	1.021403295
Lysine	genus.Romboutsia.id.11347	Simple mode	4	-0.005653928	0.028684272	0.856340746	-0.061875101	0.050567245	0.994362025	0.940000284	1.051867594
4-androsten-3beta,17beta-diol disulf	family.Desulfovibrionaceae.id.3169	Weighted median	6	0.040035715	0.061459393	0.514777111	-0.080424695	0.160496125	1.040847947	0.922724387	1.174093223
4-androsten-3beta,17beta-diol disulf	genus..Eubacteriumhalliigroup.id.11338	Weighted median	7	0.002442623	0.063020722	0.969082499	-0.121077993	0.125963239	1.002445609	0.885964858	1.134240471
4-androsten-3beta,17beta-diol disulf	genus.Collinsella.id.815	Weighted median	3	-0.061471648	0.095768208	0.520951436	-0.249177336	0.12623404	0.940379607	0.779441738	1.134547666
4-androsten-3beta,17beta-diol disulf	genus.Streptococcus.id.1853	Weighted median	5	0.022255791	0.073228675	0.761187464	-0.121272412	0.165783995	1.022505299	0.885792626	1.180318119
4-androsten-3beta,17beta-diol disulf	family.Lachnospiraceae.id.1987	Weighted median	6	0.011137907	0.067487175	0.868914551	-0.121136955	0.143412769	1.011200165	0.885912621	1.154206124
4-androsten-3beta,17beta-diol disulf	genus..Eubacteriumxylanophilumgroup.id.14375	Weighted median	4	-0.002150851	0.070211128	0.97556139	-0.139764661	0.135462959	0.997851461	0.869562853	1.145066781
4-androsten-3beta,17beta-diol disulf	genus.Romboutsia.id.11347	Weighted median	4	-0.073058281	0.084526313	0.387408888	-0.238729855	0.092613292	0.929546653	0.787627627	1.097037421
Oleoylcarnitine	family.Desulfovibrionaceae.id.3169	Weighted median	6	-0.002602353	0.033446017	0.937981134	-0.068156546	0.06295184	0.99740103	0.934114231	1.064975549
Oleoylcarnitine	genus..Eubacteriumhalliigroup.id.11338	Weighted median	7	0.038962394	0.039624264	0.325461612	-0.038701164	0.116625952	1.039731383	0.962038158	1.123699034
Oleoylcarnitine	genus.Collinsella.id.815	Weighted median	3	-0.017339043	0.051648532	0.737087891	-0.118570165	0.083892079	0.982810413	0.888189493	1.087511522
Oleoylcarnitine	genus.Streptococcus.id.1853	Weighted median	5	0.014534811	0.041669963	0.727233564	-0.067138317	0.096207938	1.014640955	0.935065857	1.100987978
Oleoylcarnitine	family.Lachnospiraceae.id.1987	Weighted median	6	0.070056097	0.039891957	0.079063494	-0.008132138	0.148244332	1.072568348	0.991900839	1.159796237
Oleoylcarnitine	genus..Eubacteriumxylanophilumgroup.id.14375	Weighted median	4	0.008957945	0.040884752	0.826570329	-0.071176169	0.089092059	1.008998187	0.931297812	1.093181289
Oleoylcarnitine	genus.Romboutsia.id.11347	Weighted median	4	0.023010089	0.046169257	0.618212339	-0.067481655	0.113501834	1.023276864	0.934744868	1.120193943
Stearoylcarnitine	family.Desulfovibrionaceae.id.3169	Weighted median	6	-0.004171103	0.030252841	0.890339397	-0.063466673	0.055124466	0.995837584	0.938505397	1.056672126
Stearoylcarnitine	genus..Eubacteriumhalliigroup.id.11338	Weighted median	7	0.045988321	0.032857888	0.1616293	-0.01841314	0.110389782	1.047062182	0.981755346	1.116713261
Stearoylcarnitine	genus.Collinsella.id.815	Weighted median	3	0.033472605	0.047884832	0.484537578	-0.060381666	0.127326876	1.034039116	0.941405163	1.135788219
Stearoylcarnitine	genus.Streptococcus.id.1853	Weighted median	5	0.020683416	0.037567667	0.581932395	-0.052949212	0.094316044	1.0208988	0.94842818	1.098906994
Stearoylcarnitine	family.Lachnospiraceae.id.1987	Weighted median	6	0.008319307	0.036367363	0.819057391	-0.062960724	0.079599338	1.008354009	0.938980353	1.082853123
Stearoylcarnitine	genus..Eubacteriumxylanophilumgroup.id.14375	Weighted median	4	0.014365671	0.034313889	0.675468511	-0.052889552	0.081620894	1.014469353	0.948484765	1.085044385
Stearoylcarnitine	genus.Romboutsia.id.11347	Weighted median	4	0.016018266	0.040867593	0.695091316	-0.064082217	0.096118749	1.016147246	0.937927883	1.100889785
Succinylcarnitine	family.Desulfovibrionaceae.id.3169	Weighted median	6	0.016549079	0.019491671	0.39586273	-0.021654596	0.054752754	1.016686774	0.978578181	1.056279422
Succinylcarnitine	genus..Eubacteriumhalliigroup.id.11338	Weighted median	7	-0.009954546	0.018962063	0.599602859	-0.047120189	0.027211096	0.990094836	0.953972733	1.027584699
Succinylcarnitine	genus.Collinsella.id.815	Weighted median	3	-0.027328305	0.03102645	0.378422451	-0.088140146	0.033483536	0.973041735	0.915632546	1.034050419
Succinylcarnitine	genus.Streptococcus.id.1853	Weighted median	5	0.021604894	0.027483661	0.431809154	-0.032263081	0.075472869	1.02183997	0.96825182	1.07839397
Succinylcarnitine	family.Lachnospiraceae.id.1987	Weighted median	6	0.032431719	0.023489803	0.167379756	-0.013608294	0.078471732	1.032963359	0.98648388	1.081632779
Succinylcarnitine	genus..Eubacteriumxylanophilumgroup.id.14375	Weighted median	4	0.036504646	0.024792925	0.140917868	-0.012089487	0.085098779	1.037179123	0.987983297	1.088824614
Succinylcarnitine	genus.Romboutsia.id.11347	Weighted median	4	0.044969919	0.031226243	0.149829998	-0.016233518	0.106173355	1.045996395	0.983897535	1.112014634
X_12798	family.Desulfovibrionaceae.id.3169	Weighted median	6	-0.043344041	0.03193797	0.174739275	-0.105942463	0.019254381	0.957581886	0.8994764	1.019440942
X_12798	genus..Eubacteriumhalliigroup.id.11338	Weighted median	7	-0.041521614	0.029879411	0.164638333	-0.100085259	0.017042032	0.959328601	0.904760276	1.017188075
X_12798	genus.Collinsella.id.815	Weighted median	3	-0.04298225	0.045985418	0.34994652	-0.133113669	0.047149169	0.957928394	0.875365585	1.048278369
X_12798	genus.Streptococcus.id.1853	Weighted median	5	-0.002920672	0.036876245	0.936871965	-0.075198113	0.069356769	0.997083589	0.927559707	1.071818533
X_12798	family.Lachnospiraceae.id.1987	Weighted median	6	-0.022726919	0.03722261	0.541485891	-0.095683235	0.050229397	0.977529392	0.908751831	1.051512282
X_12798	genus..Eubacteriumxylanophilumgroup.id.14375	Weighted median	4	-0.052989288	0.03844355	0.168090272	-0.128338646	0.02236007	0.948390172	0.879555471	1.022611931
X_12798	genus.Romboutsia.id.11347	Weighted median	4	-0.015651106	0.040582461	0.699747129	-0.09519273	0.063890517	0.984470736	0.909197688	1.065975686
X_12850	family.Desulfovibrionaceae.id.3169	Weighted median	6	-0.000534597	0.050158115	0.991496123	-0.098844502	0.097775308	0.999465546	0.905883561	1.102714986
X_12850	genus..Eubacteriumhalliigroup.id.11338	Weighted median	7	0.060016984	0.053798998	0.264602767	-0.045429051	0.165463019	1.061854581	0.955587398	1.179939327
X_12850	genus.Collinsella.id.815	Weighted median	3	-0.10223675	0.075840455	0.177642196	-0.250884042	0.046410541	0.902815785	0.778112595	1.047504367
X_12850	genus.Streptococcus.id.1853	Weighted median	5	0.009477783	0.066611372	0.886855084	-0.121080505	0.140036071	1.009522839	0.885962632	1.150315291
X_12850	family.Lachnospiraceae.id.1987	Weighted median	6	-0.048783123	0.064991623	0.452889615	-0.176166705	0.078600458	0.952387658	0.838478193	1.081772023
X_12850	genus..Eubacteriumxylanophilumgroup.id.14375	Weighted median	4	-0.014887999	0.062903718	0.812905702	-0.138179286	0.108403288	0.985222279	0.87094253	1.114497118
X_12850	genus.Romboutsia.id.11347	Weighted median	4	0.029276996	0.072297114	0.68551142	-0.112425348	0.170979341	1.029709781	0.893664058	1.186466237
Epiandrosterone sulfate	family.Desulfovibrionaceae.id.3169	Weighted median	6	-0.013661155	0.054555611	0.802271932	-0.120590153	0.093267843	0.986431735	0.886397172	1.097755722
Epiandrosterone sulfate	genus..Eubacteriumhalliigroup.id.11338	Weighted median	7	-0.003267846	0.057252326	0.954483094	-0.115482405	0.108946713	0.996737488	0.890936249	1.115102928
Epiandrosterone sulfate	genus.Collinsella.id.815	Weighted median	3	-0.06837508	0.07852659	0.383904218	-0.222287196	0.085537036	0.933910117	0.800685378	1.089301903
Epiandrosterone sulfate	genus.Streptococcus.id.1853	Weighted median	5	0.037366144	0.065423375	0.567902284	-0.090863671	0.165595959	1.038073035	0.91314219	1.180096198
Epiandrosterone sulfate	family.Lachnospiraceae.id.1987	Weighted median	6	0.056381367	0.068307511	0.409141644	-0.077501354	0.190264088	1.058001094	0.925425772	1.209568988
Epiandrosterone sulfate	genus..Eubacteriumxylanophilumgroup.id.14375	Weighted median	4	-0.048250145	0.067213433	0.47284077	-0.179988474	0.083488184	0.952895395	0.835279839	1.08707237
Epiandrosterone sulfate	genus.Romboutsia.id.11347	Weighted median	4	-0.118804879	0.076586929	0.120843645	-0.26891526	0.031305502	0.887981048	0.764208012	1.031800673
X_12442	family.Desulfovibrionaceae.id.3169	Weighted median	6	0.006835345	0.044602006	0.87819958	-0.080584587	0.094255277	1.006858759	0.922576862	1.098840219
X_12442	genus..Eubacteriumhalliigroup.id.11338	Weighted median	7	0.024972183	0.038370321	0.515162506	-0.050233645	0.100178011	1.0252866	0.9510072	1.105367668
X_12442	genus.Collinsella.id.815	Weighted median	3	0.058767925	0.060505791	0.331410013	-0.059823425	0.177359275	1.06052909	0.941930841	1.194060012
X_12442	genus.Streptococcus.id.1853	Weighted median	5	0.031396823	0.044816714	0.483577288	-0.056443937	0.119237583	1.031894902	0.94511947	1.126637556
X_12442	family.Lachnospiraceae.id.1987	Weighted median	6	0.0886689	0.051468008	0.084925349	-0.012208397	0.189546196	1.092718797	0.987865824	1.20870096
X_12442	genus..Eubacteriumxylanophilumgroup.id.14375	Weighted median	4	0.007103006	0.048640327	0.883896741	-0.088232034	0.102438046	1.007128292	0.915548414	1.107868663
X_12442	genus.Romboutsia.id.11347	Weighted median	4	0.038966723	0.058434286	0.504869925	-0.075564477	0.153497923	1.039735884	0.927219944	1.165905366
X_12092	family.Desulfovibrionaceae.id.3169	Weighted median	6	-0.00910635	0.048135259	0.849949765	-0.103451458	0.085238758	0.990934987	0.901719793	1.088977038
X_12092	genus..Eubacteriumhalliigroup.id.11338	Weighted median	7	-0.029186625	0.053046812	0.582178731	-0.133158376	0.074785127	0.971235191	0.87532645	1.077652567
X_12092	genus.Collinsella.id.815	Weighted median	3	0.04667243	0.068137062	0.49335742	-0.086876212	0.180221072	1.047778732	0.916790577	1.197482063
X_12092	genus.Streptococcus.id.1853	Weighted median	5	0.085798706	0.061692279	0.16430042	-0.035118161	0.206715572	1.089586978	0.965491326	1.22963278
X_12092	family.Lachnospiraceae.id.1987	Weighted median	6	-0.029418184	0.05865332	0.615976762	-0.144378692	0.085542323	0.971010318	0.865559905	1.089307664
X_12092	genus..Eubacteriumxylanophilumgroup.id.14375	Weighted median	4	0.040633057	0.061306273	0.50746639	-0.079527237	0.160793351	1.041469875	0.923552865	1.174442247
X_12092	genus.Romboutsia.id.11347	Weighted median	4	0.039795263	0.061116536	0.514957784	-0.079993147	0.159583673	1.040597704	0.923122673	1.173022408
X_11440	family.Desulfovibrionaceae.id.3169	Weighted median	6	0.025220966	0.044217685	0.568418722	-0.061445697	0.11188763	1.025541706	0.940404011	1.11838718
X_11440	genus..Eubacteriumhalliigroup.id.11338	Weighted median	7	-0.041501491	0.045315895	0.359757974	-0.130320645	0.047317663	0.959347905	0.877813919	1.048455011
X_11440	genus.Collinsella.id.815	Weighted median	3	0.001040396	0.070402709	0.988209469	-0.136948914	0.139029705	1.001040937	0.872014772	1.149158236
X_11440	genus.Streptococcus.id.1853	Weighted median	5	0.012155677	0.055786262	0.827509113	-0.097185397	0.121496752	1.012229858	0.907387764	1.129185698
X_11440	family.Lachnospiraceae.id.1987	Weighted median	6	0.02162281	0.052453303	0.680171059	-0.081185663	0.124431283	1.021858277	0.92202249	1.132504196
X_11440	genus..Eubacteriumxylanophilumgroup.id.14375	Weighted median	4	0.009558868	0.059764612	0.872926817	-0.107579773	0.126697508	1.009604699	0.898004883	1.135073615
X_11440	genus.Romboutsia.id.11347	Weighted median	4	-0.029460582	0.065177321	0.651264196	-0.157208132	0.098286968	0.97096915	0.854526186	1.103279345
X_11423	family.Desulfovibrionaceae.id.3169	Weighted median	6	-0.021925265	0.019258927	0.254933935	-0.059672763	0.015822232	0.978313346	0.942072764	1.015948066
X_11423	genus..Eubacteriumhalliigroup.id.11338	Weighted median	7	-0.000486111	0.018309141	0.97881849	-0.036372028	0.035399805	0.999514007	0.964281487	1.036033838
X_11423	genus.Collinsella.id.815	Weighted median	3	0.026999439	0.023991239	0.260424877	-0.020023388	0.074022267	1.027367227	0.980175748	1.076830783
X_11423	genus.Streptococcus.id.1853	Weighted median	5	-0.025277278	0.019731194	0.200164862	-0.063950419	0.013395863	0.975039517	0.938051508	1.013485989
X_11423	family.Lachnospiraceae.id.1987	Weighted median	6	0.019238778	0.018816559	0.306573315	-0.017641678	0.056119234	1.019425036	0.982513025	1.057723793
X_11423	genus..Eubacteriumxylanophilumgroup.id.14375	Weighted median	4	-0.006556737	0.021584552	0.761303212	-0.048862459	0.035748985	0.993464712	0.952312103	1.036395663
X_11423	genus.Romboutsia.id.11347	Weighted median	4	0.020676759	0.022955458	0.367729844	-0.02431594	0.065669457	1.020892004	0.975977311	1.067873681
3-dehydrocarnitine	family.Desulfovibrionaceae.id.3169	Weighted median	6	0.014250155	0.022108366	0.51921275	-0.029082243	0.057582554	1.014352173	0.971336576	1.059272714
3-dehydrocarnitine	genus..Eubacteriumhalliigroup.id.11338	Weighted median	7	-0.016147406	0.023424684	0.490613778	-0.062059788	0.029764975	0.983982264	0.939826695	1.03021238
3-dehydrocarnitine	genus.Collinsella.id.815	Weighted median	3	-0.040538287	0.033376636	0.224529836	-0.105956493	0.024879919	0.960272398	0.899463781	1.025192007
3-dehydrocarnitine	genus.Streptococcus.id.1853	Weighted median	5	0.006681267	0.027731788	0.809613638	-0.047673038	0.061035572	1.006703637	0.953445476	1.062936725
3-dehydrocarnitine	family.Lachnospiraceae.id.1987	Weighted median	6	-0.037437608	0.026780976	0.162137703	-0.089928322	0.015053106	0.963254515	0.913996697	1.015166974
3-dehydrocarnitine	genus..Eubacteriumxylanophilumgroup.id.14375	Weighted median	4	-0.05618892	0.028805839	0.05110361	-0.112648364	0.000270524	0.945360521	0.893464779	1.000270561
3-dehydrocarnitine	genus.Romboutsia.id.11347	Weighted median	4	0.018689507	0.033570601	0.577717069	-0.04710887	0.084487884	1.018865249	0.953983532	1.08815966
Propionylcarnitine	family.Desulfovibrionaceae.id.3169	Weighted median	6	0.001252305	0.021782117	0.954153005	-0.041440644	0.043945254	1.001253089	0.95940628	1.044925148
Propionylcarnitine	genus..Eubacteriumhalliigroup.id.11338	Weighted median	7	0.014165708	0.020722065	0.494224613	-0.026449539	0.054780954	1.014266517	0.973897186	1.056309209
Propionylcarnitine	genus.Collinsella.id.815	Weighted median	3	0.037653136	0.028280334	0.183049302	-0.017776319	0.09308259	1.038370997	0.982380748	1.097552379
Propionylcarnitine	genus.Streptococcus.id.1853	Weighted median	5	-0.006573915	0.025491037	0.796490857	-0.056536347	0.043388518	0.993447646	0.945032134	1.044343562
Propionylcarnitine	family.Lachnospiraceae.id.1987	Weighted median	6	-0.038701989	0.031258998	0.215676337	-0.099969626	0.022565648	0.962037364	0.904864902	1.022822178
Propionylcarnitine	genus..Eubacteriumxylanophilumgroup.id.14375	Weighted median	4	0.005633796	0.025257475	0.823493025	-0.043870856	0.055138447	1.005649695	0.957077551	1.056686899
Propionylcarnitine	genus.Romboutsia.id.11347	Weighted median	4	-0.005284848	0.028545546	0.853121267	-0.061234118	0.050664422	0.994729092	0.940603002	1.051969816
Acetylcarnitine	family.Desulfovibrionaceae.id.3169	Weighted median	6	0.022886614	0.024072826	0.341744486	-0.024296125	0.070069353	1.023150522	0.97599665	1.072582565
Acetylcarnitine	genus..Eubacteriumhalliigroup.id.11338	Weighted median	7	0.017782794	0.021994429	0.418795004	-0.025326287	0.060891875	1.017941849	0.974991733	1.062783995
Acetylcarnitine	genus.Collinsella.id.815	Weighted median	3	0.014044681	0.031227869	0.652891937	-0.047161942	0.075251304	1.014143771	0.953932903	1.078155062
Acetylcarnitine	genus.Streptococcus.id.1853	Weighted median	5	0.02358299	0.026399668	0.371693157	-0.028160359	0.07532634	1.023863268	0.972232448	1.078235965
Acetylcarnitine	family.Lachnospiraceae.id.1987	Weighted median	6	0.028748778	0.027097356	0.288715341	-0.02436204	0.081859595	1.029166012	0.97593232	1.085303417
Acetylcarnitine	genus..Eubacteriumxylanophilumgroup.id.14375	Weighted median	4	-0.035258533	0.031384783	0.261255909	-0.096772709	0.026255642	0.965355807	0.907762309	1.026603358
Acetylcarnitine	genus.Romboutsia.id.11347	Weighted median	4	0.002550712	0.029882106	0.931975834	-0.056018215	0.06111964	1.002553968	0.945521913	1.063026087
Palmitoylcarnitine	family.Desulfovibrionaceae.id.3169	Weighted median	6	0.018541567	0.031544728	0.556675573	-0.043286101	0.080369235	1.018714529	0.95763737	1.083687129
Palmitoylcarnitine	genus..Eubacteriumhalliigroup.id.11338	Weighted median	7	0.042858704	0.040356363	0.288232912	-0.036239768	0.121957177	1.043790401	0.964409032	1.129705723
Palmitoylcarnitine	genus.Collinsella.id.815	Weighted median	3	-0.018947636	0.046457123	0.683382253	-0.110003596	0.072108325	0.981230742	0.895830914	1.074771762
Palmitoylcarnitine	genus.Streptococcus.id.1853	Weighted median	5	-0.001828651	0.035912034	0.959389036	-0.072216238	0.068558935	0.99817302	0.930329701	1.070963741
Palmitoylcarnitine	family.Lachnospiraceae.id.1987	Weighted median	6	-0.001562273	0.038729821	0.967823879	-0.077472722	0.074348176	0.998438947	0.925452269	1.077181789
Palmitoylcarnitine	genus..Eubacteriumxylanophilumgroup.id.14375	Weighted median	4	-0.008279541	0.035286623	0.814490629	-0.077441321	0.06088224	0.99175464	0.925481329	1.062773754
Palmitoylcarnitine	genus.Romboutsia.id.11347	Weighted median	4	-0.003564596	0.045515045	0.937576002	-0.092774084	0.085644892	0.99644175	0.911399377	1.089419398
Betaine	family.Desulfovibrionaceae.id.3169	Weighted median	6	0.025734009	0.024872694	0.300842223	-0.02301647	0.074484489	1.026067987	0.977246388	1.077328632
Betaine	genus..Eubacteriumhalliigroup.id.11338	Weighted median	7	0.012893873	0.022192952	0.561247512	-0.030604314	0.056392059	1.012977357	0.969859257	1.058012406
Betaine	genus.Collinsella.id.815	Weighted median	3	-0.006869184	0.031296833	0.826272299	-0.068210976	0.054472608	0.993154355	0.934063388	1.055983551
Betaine	genus.Streptococcus.id.1853	Weighted median	5	0.002035287	0.025068916	0.935292697	-0.047099789	0.051170363	1.00203736	0.953992195	1.052502186
Betaine	family.Lachnospiraceae.id.1987	Weighted median	6	-0.003328748	0.02523226	0.895044183	-0.052783978	0.046126482	0.996676786	0.948584906	1.047206856
Betaine	genus..Eubacteriumxylanophilumgroup.id.14375	Weighted median	4	-0.010616242	0.026751932	0.69148529	-0.063050028	0.041817544	0.989439911	0.938896501	1.042704213
Betaine	genus.Romboutsia.id.11347	Weighted median	4	0.029118294	0.028688119	0.310108252	-0.027110418	0.085347007	1.029546377	0.973253771	1.089094925
Lysine	family.Desulfovibrionaceae.id.3169	Weighted median	6	0.000688093	0.015100425	0.963654762	-0.028908741	0.030284926	1.000688329	0.971505119	1.03074818
Lysine	genus..Eubacteriumhalliigroup.id.11338	Weighted median	7	-0.006199828	0.015712903	0.693160808	-0.036997118	0.024597461	0.993819351	0.963678913	1.024902474
Lysine	genus.Collinsella.id.815	Weighted median	3	0.008952617	0.022693248	0.693207676	-0.035526149	0.053431383	1.008992812	0.965097498	1.054884606
Lysine	genus.Streptococcus.id.1853	Weighted median	5	0.007914052	0.019254835	0.681060565	-0.029825426	0.045653529	1.007945451	0.970614963	1.046711693
Lysine	family.Lachnospiraceae.id.1987	Weighted median	6	1.52E-05	0.018121541	0.99932988	-0.035503001	0.035533441	1.00001522	0.965119837	1.036172298
Lysine	genus..Eubacteriumxylanophilumgroup.id.14375	Weighted median	4	-0.025495621	0.01790314	0.15442141	-0.060585775	0.009594533	0.974826648	0.941213033	1.009640708
Lysine	genus.Romboutsia.id.11347	Weighted median	4	-0.009660136	0.022876437	0.672824676	-0.054497952	0.03517768	0.990386374	0.946960449	1.035803734
4-androsten-3beta,17beta-diol disulf	family.Desulfovibrionaceae.id.3169	Weighted mode	6	0.052469693	0.089825753	0.584494144	-0.123588783	0.228528169	1.053870622	0.883743177	1.256748926
4-androsten-3beta,17beta-diol disulf	genus..Eubacteriumhalliigroup.id.11338	Weighted mode	7	0.019713453	0.091292025	0.83618972	-0.159218916	0.198645823	1.019909047	0.852809645	1.219749882
4-androsten-3beta,17beta-diol disulf	genus.Collinsella.id.815	Weighted mode	3	-0.052596482	0.102190834	0.658004968	-0.252890517	0.147697552	0.948762778	0.776552897	1.159162258

4-androsten-3beta,17beta-diol disulf	genus.Streptococcus.id.1853	Weighted mode	5	0.023453611	0.094574565	0.816352153	-0.161912537	0.208819759	1.02373081	0.85051559	1.232222882
4-androsten-3beta,17beta-diol disulf	family.Lachnospiraceae.id.1987	Weighted mode	6	-0.00565813	0.094229459	0.954444896	-0.190347869	0.179031609	0.994357847	0.826671511	1.19605855
4-androsten-3beta,17beta-diol disulf	genus..Eubacteriumxylanophilumgroup.id.14375	Weighted mode	4	0.002214219	0.09858159	0.98349082	-0.191005698	0.195434135	1.002216672	0.826127881	1.21583871
4-androsten-3beta,17beta-diol disulf	genus.Romboutsia.id.11347	Weighted mode	4	-0.08149347	0.092772286	0.444384754	-0.263327151	0.100340211	0.921738729	0.768490444	1.105546973
Oleoylcarnitine	family.Desulfovibrionaceae.id.3169	Weighted mode	6	-0.020186531	0.055005972	0.728647747	-0.127998236	0.087625175	0.980015853	0.879854931	1.091578894
Oleoylcarnitine	genus..Eubacteriumhalliigroup.id.11338	Weighted mode	7	0.034594447	0.065024132	0.61382301	-0.092852852	0.162041746	1.035199795	0.911327591	1.175909329
Oleoylcarnitine	genus.Collinsella.id.815	Weighted mode	3	-0.013055147	0.054925985	0.834255117	-0.120710077	0.094599783	0.987029701	0.886290878	1.099218841
Oleoylcarnitine	genus.Streptococcus.id.1853	Weighted mode	5	0.001106983	0.053111196	0.984369362	-0.102990962	0.105204927	1.001107596	0.902135127	1.110938248
Oleoylcarnitine	family.Lachnospiraceae.id.1987	Weighted mode	6	0.079097332	0.052416234	0.191669055	-0.023638488	0.181833151	1.08230966	0.976638713	1.199414056
Oleoylcarnitine	genus..Eubacteriumxylanophilumgroup.id.14375	Weighted mode	4	0.016879555	0.048995773	0.753198896	-0.079152161	0.11291127	1.017022819	0.923899333	1.119532592
Oleoylcarnitine	genus.Romboutsia.id.11347	Weighted mode	4	0.013954137	0.060002298	0.831065804	-0.103650368	0.131558641	1.01405195	0.90154045	1.140604792
Stearoylcarnitine	family.Desulfovibrionaceae.id.3169	Weighted mode	6	-0.003047793	0.043325784	0.94664521	-0.08796633	0.081870743	0.996956846	0.915791711	1.085315516
Stearoylcarnitine	genus..Eubacteriumhalliigroup.id.11338	Weighted mode	7	0.059298504	0.044900757	0.23475173	-0.02870698	0.147303988	1.061091934	0.971701151	1.158706142
Stearoylcarnitine	genus.Collinsella.id.815	Weighted mode	3	0.039290363	0.04462794	0.471506329	-0.048180399	0.126761125	1.040072438	0.952961858	1.135145827
Stearoylcarnitine	genus.Streptococcus.id.1853	Weighted mode	5	0.014482888	0.044777911	0.762566929	-0.073281818	0.102247593	1.014588273	0.929338889	1.107657686
Stearoylcarnitine	family.Lachnospiraceae.id.1987	Weighted mode	6	0.012955869	0.049520701	0.804045764	-0.084104705	0.110016442	1.013040159	0.919334992	1.116296425
Stearoylcarnitine	genus..Eubacteriumxylanophilumgroup.id.14375	Weighted mode	4	0.011036152	0.044498222	0.820131092	-0.076180363	0.098252667	1.011097275	0.926649058	1.103241503
Stearoylcarnitine	genus.Romboutsia.id.11347	Weighted mode	4	0.020265906	0.049432476	0.709345333	-0.076621748	0.117153559	1.020472653	0.92624014	1.124292061
Succinylcarnitine	family.Desulfovibrionaceae.id.3169	Weighted mode	6	0.025796462	0.028866321	0.412451709	-0.030781527	0.082374451	1.026132071	0.9696874	1.085862336
Succinylcarnitine	genus..Eubacteriumhalliigroup.id.11338	Weighted mode	7	-0.014817635	0.024918311	0.573802848	-0.063657525	0.034022255	0.985291606	0.938326298	1.034607631
Succinylcarnitine	genus.Collinsella.id.815	Weighted mode	3	-0.032185069	0.0412787	0.51718575	-0.113091321	0.048721184	0.968327358	0.8930691	1.049927573
Succinylcarnitine	genus.Streptococcus.id.1853	Weighted mode	5	0.038559424	0.034054477	0.320788902	-0.028187351	0.105306199	1.039312487	0.972206206	1.11105076
Succinylcarnitine	family.Lachnospiraceae.id.1987	Weighted mode	6	0.037070982	0.03233375	0.303471632	-0.026303168	0.100445132	1.037766681	0.974039747	1.105662975
Succinylcarnitine	genus..Eubacteriumxylanophilumgroup.id.14375	Weighted mode	4	0.033653941	0.028665677	0.325106005	-0.022530785	0.089838668	1.034226641	0.977721137	1.093997772
Succinylcarnitine	genus.Romboutsia.id.11347	Weighted mode	4	0.044492742	0.041285496	0.360110575	-0.036426831	0.125412315	1.045497389	0.964228643	1.133615764
X_12798	family.Desulfovibrionaceae.id.3169	Weighted mode	6	-0.051548462	0.046980564	0.322550314	-0.143630369	0.040533444	0.949757621	0.866207866	1.041366136
X_12798	genus..Eubacteriumhalliigroup.id.11338	Weighted mode	7	-0.048046759	0.045259173	0.329271349	-0.136754739	0.040661221	0.95308922	0.872184113	1.041499207
X_12798	genus.Collinsella.id.815	Weighted mode	3	-0.045092174	0.052278094	0.479296207	-0.14755724	0.057372891	0.955909367	0.86281305	1.059050647
X_12798	genus.Streptococcus.id.1853	Weighted mode	5	0.037488489	0.056832001	0.545531799	-0.073902233	0.148879211	1.038200046	0.928762492	1.160532801
X_12798	family.Lachnospiraceae.id.1987	Weighted mode	6	-0.010434568	0.05070405	0.845068835	-0.109814507	0.08894537	0.989619683	0.896000322	1.093020943
X_12798	genus..Eubacteriumxylanophilumgroup.id.14375	Weighted mode	4	-0.074344065	0.052416339	0.251126661	-0.17708009	0.02839196	0.928352225	0.837712689	1.028798853
X_12798	genus.Romboutsia.id.11347	Weighted mode	4	-0.017170797	0.045953728	0.733505238	-0.107240105	0.072898511	0.982975781	0.898309959	1.075621367
X_12850	family.Desulfovibrionaceae.id.3169	Weighted mode	6	-0.013446219	0.072738268	0.860605055	-0.156013223	0.129120786	0.986643778	0.855547877	1.137827549
X_12850	genus..Eubacteriumhalliigroup.id.11338	Weighted mode	7	0.057975695	0.080364131	0.497791542	-0.099538002	0.215489393	1.05968924	0.905255548	1.240468825
X_12850	genus.Collinsella.id.815	Weighted mode	3	-0.102669584	0.07308769	0.295272227	-0.245921455	0.040582288	0.9024251	0.781983643	1.041417002
X_12850	genus.Streptococcus.id.1853	Weighted mode	5	0.038608668	0.087622703	0.682251147	-0.133131829	0.210349165	1.039363667	0.875349688	1.234108892
X_12850	family.Lachnospiraceae.id.1987	Weighted mode	6	-0.043213177	0.088736112	0.646865996	-0.217135956	0.130709601	0.957707207	0.804820542	1.139636784
X_12850	genus..Eubacteriumxylanophilumgroup.id.14375	Weighted mode	4	0.003345061	0.081627464	0.969886917	-0.156644768	0.16333489	1.003350662	0.855007731	1.177430933
X_12850	genus.Romboutsia.id.11347	Weighted mode	4	0.049315161	0.077901598	0.571666501	-0.10337197	0.202002293	1.050551392	0.901791472	1.223850814
Epiandrosterone sulfate	family.Desulfovibrionaceae.id.3169	Weighted mode	6	0.021982116	0.092294853	0.821200016	-0.158915795	0.202880028	1.022225503	0.853068189	1.224925502
Epiandrosterone sulfate	genus..Eubacteriumhalliigroup.id.11338	Weighted mode	7	0.009650704	0.076730447	0.904019338	-0.140740971	0.160042379	1.009697422	0.868714305	1.173560604
Epiandrosterone sulfate	genus.Collinsella.id.815	Weighted mode	3	-0.078358229	0.093168542	0.488855608	-0.260968571	0.104252113	0.924633137	0.770305129	1.109880234
Epiandrosterone sulfate	genus.Streptococcus.id.1853	Weighted mode	5	0.038588125	0.081874128	0.661975347	-0.121885167	0.199061417	1.039342317	0.885250019	1.220256908
Epiandrosterone sulfate	family.Lachnospiraceae.id.1987	Weighted mode	6	-0.033235717	0.118343403	0.790082412	-0.265188788	0.198717354	0.967310521	0.767061125	1.219837135
Epiandrosterone sulfate	genus..Eubacteriumxylanophilumgroup.id.14375	Weighted mode	4	0.041403227	0.105033228	0.719789071	-0.164461899	0.247268353	1.042272293	0.848350079	1.280522699
Epiandrosterone sulfate	genus.Romboutsia.id.11347	Weighted mode	4	-0.13378125	0.093723813	0.24875212	-0.317479923	0.049917424	0.874781403	0.727981296	1.05118429
X_12442	family.Desulfovibrionaceae.id.3169	Weighted mode	6	-0.018558217	0.07519178	0.814867219	-0.165934105	0.128817671	0.981612926	0.847102052	1.137482709
X_12442	genus..Eubacteriumhalliigroup.id.11338	Weighted mode	7	0.017038933	0.057064907	0.775324964	-0.094808284	0.128886149	1.017184923	0.909547292	1.137560605
X_12442	genus.Collinsella.id.815	Weighted mode	3	0.062478345	0.063978288	0.431779869	-0.062919098	0.187875789	1.064471408	0.939019439	1.206683622
X_12442	genus.Streptococcus.id.1853	Weighted mode	5	0.031191761	0.05394176	0.594090618	-0.074534089	0.136917611	1.031683321	0.928175833	1.146733666
X_12442	family.Lachnospiraceae.id.1987	Weighted mode	6	0.145427529	0.067069346	0.082317869	0.013971611	0.276883447	1.156533916	1.01406967	1.319012627
X_12442	genus..Eubacteriumxylanophilumgroup.id.14375	Weighted mode	4	0.020277598	0.059846485	0.757069781	-0.097021513	0.137576708	1.020484585	0.907536482	1.147489724
X_12442	genus.Romboutsia.id.11347	Weighted mode	4	0.063990256	0.076723798	0.465458665	-0.086388388	0.214368901	1.066082011	0.917237918	1.239079668
X_12092	family.Desulfovibrionaceae.id.3169	Weighted mode	6	0.02530406	0.082293877	0.770870288	-0.135991939	0.186600059	1.025626925	0.872849668	1.205145202
X_12092	genus..Eubacteriumhalliigroup.id.11338	Weighted mode	7	-0.010708846	0.090231131	0.909400754	-0.187561862	0.16614417	0.98934829	0.828977835	1.180743317
X_12092	genus.Collinsella.id.815	Weighted mode	3	0.051118093	0.076522997	0.572896209	-0.098866982	0.201103168	1.052447172	0.905863196	1.222750914
X_12092	genus.Streptococcus.id.1853	Weighted mode	5	0.089090586	0.068061901	0.260680099	-0.04431074	0.222491912	1.093179679	0.95665664	1.249185716
X_12092	family.Lachnospiraceae.id.1987	Weighted mode	6	-0.031021076	0.089589431	0.743243461	-0.206616362	0.144574209	0.96945514	0.813331615	1.155547444
X_12092	genus..Eubacteriumxylanophilumgroup.id.14375	Weighted mode	4	0.048995934	0.089144397	0.620847779	-0.125727084	0.223718952	1.050216081	0.881855486	1.250719458
X_12092	genus.Romboutsia.id.11347	Weighted mode	4	0.036661797	0.070102372	0.637159775	-0.100738852	0.174062445	1.037342129	0.904169124	1.190129881
X_11440	family.Desulfovibrionaceae.id.3169	Weighted mode	6	0.020042791	0.061986887	0.759523856	-0.101451507	0.141537089	1.020244996	0.903524993	1.152043232
X_11440	genus..Eubacteriumhalliigroup.id.11338	Weighted mode	7	-0.063266225	0.076188564	0.438104346	-0.21259581	0.086063361	0.938693537	0.808482852	1.089875381
X_11440	genus.Collinsella.id.815	Weighted mode	3	0.003819798	0.068079245	0.960356764	-0.129615523	0.137255118	1.003827102	0.878433103	1.147120763
X_11440	genus.Streptococcus.id.1853	Weighted mode	5	0.016757656	0.069464613	0.821230523	-0.119392986	0.152908298	1.016898853	0.887458973	1.165218122
X_11440	family.Lachnospiraceae.id.1987	Weighted mode	6	0.006316885	0.074950393	0.936103575	-0.140585886	0.153219656	1.006336879	0.86884904	1.165580978
X_11440	genus..Eubacteriumxylanophilumgroup.id.14375	Weighted mode	4	-0.029450643	0.074789623	0.720062801	-0.176038304	0.117137018	0.970978801	0.838585861	1.124273464
X_11440	genus.Romboutsia.id.11347	Weighted mode	4	-0.031705169	0.072302142	0.690674951	-0.173417368	0.11000703	0.96879217	0.840786624	1.116285918
X_11423	family.Desulfovibrionaceae.id.3169	Weighted mode	6	-0.024250902	0.022993038	0.339834894	-0.069317256	0.020815452	0.976040788	0.933030623	1.021033604
X_11423	genus..Eubacteriumhalliigroup.id.11338	Weighted mode	7	-0.026542051	0.030535236	0.418132647	-0.086391113	0.033307011	0.973807093	0.917235419	1.033867899
X_11423	genus.Collinsella.id.815	Weighted mode	3	0.009395703	0.024121748	0.73446138	-0.037882923	0.056674329	1.009439981	0.962825659	1.058311093
X_11423	genus.Streptococcus.id.1853	Weighted mode	5	-0.024475439	0.021993657	0.32814324	-0.067583007	0.018632129	0.975821656	0.934650135	1.01880679
X_11423	family.Lachnospiraceae.id.1987	Weighted mode	6	0.021236914	0.024698674	0.429167235	-0.027172487	0.069646316	1.021464022	0.973193364	1.072128919
X_11423	genus..Eubacteriumxylanophilumgroup.id.14375	Weighted mode	4	-0.012109951	0.025849009	0.671375002	-0.062774009	0.038554106	0.987963079	0.93915569	1.03930696
X_11423	genus.Romboutsia.id.11347	Weighted mode	4	0.027323342	0.02899571	0.415545293	-0.02950825	0.084154935	1.027700048	0.970922867	1.087797418
3-dehydrocarnitine	family.Desulfovibrionaceae.id.3169	Weighted mode	6	0.017407661	0.030567864	0.593663023	-0.042505353	0.077320675	1.017560058	0.958385335	1.080388475
3-dehydrocarnitine	genus..Eubacteriumhalliigroup.id.11338	Weighted mode	7	-0.016373931	0.03079011	0.613974704	-0.076722546	0.043974684	0.983759394	0.926146782	1.044955901
3-dehydrocarnitine	genus.Collinsella.id.815	Weighted mode	3	-0.045923423	0.032566409	0.293912062	-0.109753586	0.017906739	0.955115099	0.896054909	1.018068026
3-dehydrocarnitine	genus.Streptococcus.id.1853	Weighted mode	5	0.011312068	0.038907577	0.785700254	-0.064946783	0.08757092	1.011376292	0.937117333	1.091519672
3-dehydrocarnitine	family.Lachnospiraceae.id.1987	Weighted mode	6	-0.037966477	0.039634322	0.382096811	-0.115649748	0.039716795	0.962745215	0.890787168	1.040516053
3-dehydrocarnitine	genus..Eubacteriumxylanophilumgroup.id.14375	Weighted mode	4	-0.05891622	0.033859127	0.180228728	-0.12528011	0.00744767	0.942785752	0.882249741	1.007475473
3-dehydrocarnitine	genus.Romboutsia.id.11347	Weighted mode	4	0.01583454	0.036611182	0.69457912	-0.055923377	0.087592457	1.015960571	0.945611589	1.091543181
Propionylcarnitine	family.Desulfovibrionaceae.id.3169	Weighted mode	6	-0.015608957	0.030978559	0.63576802	-0.076326932	0.045109018	0.984512232	0.926513251	1.046141902
Propionylcarnitine	genus..Eubacteriumhalliigroup.id.11338	Weighted mode	7	0.017063401	0.026489694	0.543300764	-0.034856399	0.0689832	1.017209812	0.965744088	1.071418209
Propionylcarnitine	genus.Collinsella.id.815	Weighted mode	3	0.038104999	0.027885157	0.305129217	-0.016549909	0.092759906	1.038840304	0.983586288	1.097198273
Propionylcarnitine	genus.Streptococcus.id.1853	Weighted mode	5	-0.010787295	0.030217006	0.739133308	-0.070012627	0.048438036	0.989270679	0.932382047	1.049630331
Propionylcarnitine	family.Lachnospiraceae.id.1987	Weighted mode	6	-0.041540348	0.05440843	0.479625989	-0.14818087	0.065100175	0.959310629	0.862275141	1.067265932
Propionylcarnitine	genus..Eubacteriumxylanophilumgroup.id.14375	Weighted mode	4	0.011790088	0.031991	0.736939227	-0.050912272	0.074492448	1.011859865	0.95036204	1.077337207
Propionylcarnitine	genus.Romboutsia.id.11347	Weighted mode	4	-0.004045041	0.029674298	0.900205898	-0.062206664	0.054116583	0.99596313	0.939688667	1.055607661
Acetylcarnitine	family.Desulfovibrionaceae.id.3169	Weighted mode	6	0.020245765	0.038047314	0.617428739	-0.054326972	0.094818501	1.0204521	0.947122374	1.099459286
Acetylcarnitine	genus..Eubacteriumhalliigroup.id.11338	Weighted mode	7	0.016665922	0.030715811	0.606965841	-0.043537067	0.076868911	1.016805573	0.957397066	1.079900504
Acetylcarnitine	genus.Collinsella.id.815	Weighted mode	3	0.015318769	0.033067703	0.688705314	-0.049493929	0.080131467	1.015436703	0.951710936	1.083429494
Acetylcarnitine	genus.Streptococcus.id.1853	Weighted mode	5	0.015976322	0.036334066	0.682862609	-0.055238448	0.087191091	1.016104625	0.946259487	1.091105161
Acetylcarnitine	family.Lachnospiraceae.id.1987	Weighted mode	6	0.035450182	0.041774703	0.43483614	-0.046428236	0.1173286	1.036086031	0.954633066	1.124488876
Acetylcarnitine	genus..Eubacteriumxylanophilumgroup.id.14375	Weighted mode	4	-0.038409786	0.049532166	0.494589186	-0.135492831	0.058673258	0.962318515	0.873285424	1.060428698
Acetylcarnitine	genus.Romboutsia.id.11347	Weighted mode	4	0.000474602	0.034312177	0.989832534	-0.066777265	0.06772647	1.000474715	0.935403526	1.070072571
Palmitoylcarnitine	family.Desulfovibrionaceae.id.3169	Weighted mode	6	0.04692027	0.054776504	0.430807956	-0.060441678	0.154282219	1.048038446	0.941348669	1.166820139
Palmitoylcarnitine	genus..Eubacteriumhalliigroup.id.11338	Weighted mode	7	0.097251947	0.089284539	0.3178527	-0.07774575	0.272249645	1.102138019	0.925199628	1.312914722
Palmitoylcarnitine	genus.Collinsella.id.815	Weighted mode	3	-0.030987734	0.064082867	0.676463668	-0.156590152	0.094614685	0.969487465	0.855054429	1.099235222
Palmitoylcarnitine	genus.Streptococcus.id.1853	Weighted mode	5	-0.005018923	0.046707569	0.919602649	-0.096565757	0.086527911	0.994993651	0.907950191	1.090381801
Palmitoylcarnitine	family.Lachnospiraceae.id.1987	Weighted mode	6	-0.01148234	0.057177795	0.848754078	-0.123550818	0.100586138	0.98858333	0.883776728	1.10581889
Palmitoylcarnitine	genus..Eubacteriumxylanophilumgroup.id.14375	Weighted mode	4	0.002729261	0.048990468	0.959075477	-0.093292057	0.098750579	1.002732989	0.910927419	1.103790956
Palmitoylcarnitine	genus.Romboutsia.id.11347	Weighted mode	4	-0.013281202	0.053101016	0.818650209	-0.117359193	0.090796789	0.986806604	0.889265717	1.095046458
Betaine	family.Desulfovibrionaceae.id.3169	Weighted mode	6	0.043486448	0.045174458	0.37994272	-0.04505549	0.132028386	1.04444584	0.955944435	1.141140711
Betaine	genus..Eubacteriumhalliigroup.id.11338	Weighted mode	7	0.00172098	0.034401322	0.961725011	-0.065705611	0.069147571	1.001722462	0.936406492	1.071594334
Betaine	genus.Collinsella.id.815	Weighted mode	3	-0.0274791	0.037850333	0.543306908	-0.101665753	0.046707553	0.972895016	0.903331437	1.047815534
Betaine	genus.Streptococcus.id.1853	Weighted mode	5	0.011908353	0.036220222	0.758817516	-0.059083282	0.082899988	1.01197954	0.942628262	1.086433147
Betaine	family.Lachnospiraceae.id.1987	Weighted mode	6	-0.002700211	0.041935026	0.951154479	-0.084892863	0.07949244	0.997303431	0.918610697	1.082737374
Betaine	genus..Eubacteriumxylanophilumgroup.id.14375	Weighted mode	4	0.000720394	0.030200555	0.982467254	-0.058472694	0.059913483	1.000720654	0.943203995	1.061744683
Betaine	genus.Romboutsia.id.11347	Weighted mode	4	0.027963485	0.029884823	0.418449771	-0.030610768	0.086537739	1.028358134	0.969852998	1.090392517
Lysine	family.Desulfovibrionaceae.id.3169	Weighted mode	6	0.000953763	0.020510717	0.964711285	-0.039247242	0.041154769	1.000954218	0.961512953	1.042013364
Lysine	genus..Eubacteriumhalliigroup.id.11338	Weighted mode	7	-0.016763614	0.026115304	0.544661566	-0.067949611	0.034422382	0.983376113	0.934307552	1.035021689
Lysine	genus.Collinsella.id.815	Weighted mode	3	0.013075448	0.024422968	0.645953307	-0.034793568	0.060944465	1.013161306	0.965804768	1.062839888
Lysine	genus.Streptococcus.id.1853	Weighted mode	5	-0.014155755	0.02849655	0.645424537	-0.070008993	0.041697483	0.985943967	0.932385435	1.042579033
Lysine	family.Lachnospiraceae.id.1987	Weighted mode	6	-0.000603483	0.028347735	0.983838899	-0.056165044	0.054958079	0.999396699	0.945383093	1.056496324
Lysine	genus..Eubacteriumxylanophilumgroup.id.14375	Weighted mode	4	-0.024338568	0.022427457	0.357245582	-0.068296383	0.019619247	0.975955227	0.933983615	1.01981297
Lysine	genus.Romboutsia.id.11347	Weighted mode	4	-0.011323304	0.023515277	0.663082367	-0.057413248	0.034766639	0.988740563	0.944203799	1.035378064

Supplementary Table 10.Five Mendelian randomization models estimate the causal effects of blood metabolites on gut microbiota

Notations: Green back means P-value < 0.05 in inverse variance weighted method

Outcome	Exposure	Method	nsnp	Beta	Standard error	P-value	lo_ci	up_ci	OR	OR_lci95	OR_uci95
genus.Collinsella.id.815	succinylcarnitine	MR Egger	6	0.838493591	0.641437384	0.261220088	-0.418723682	2.095710864	2.312880208	0.657885956	8.131219108
genus.Collinsella.id.815	succinylcarnitine	Weighted median	6	0.736211743	0.287809328	0.010528085	0.17210546	1.300318026	2.088010592	1.187803092	3.670463786
genus.Collinsella.id.815	succinylcarnitine	Inverse variance weighted	6	0.691065786	0.283775	0.01488111	0.134866785	1.247264786	1.995841539	1.144384325	3.480809168
genus.Collinsella.id.815	succinylcarnitine	Simple mode	6	0.749624353	0.460704094	0.164638599	-0.153355671	1.652604376	2.116204921	0.857824565	5.220558437
genus.Collinsella.id.815	succinylcarnitine	Weighted mode	6	0.712848663	0.315528944	0.073423612	0.094411933	1.331285392	2.039793674	1.099012372	3.785906635
genus.Collinsella.id.815	lysine	Wald ratio	1	1.295968931	0.935021221	0.165737264	-0.536672662	3.128610525	3.654535252	0.584690482	22.84221878
genus.Collinsella.id.815	oleoylcarnitine	MR Egger	3	1.077687789	15.52015881	0.95586528	-29.34182348	31.49719906	2.937878695	1.81E-13	4.77595E+13
genus.Collinsella.id.815	oleoylcarnitine	Weighted median	3	-0.539399469	0.37042243	0.145344557	-1.265427432	0.186628494	0.583098316	0.282118684	1.205179471
genus.Collinsella.id.815	oleoylcarnitine	Inverse variance weighted	3	-0.512243866	0.318024537	0.10724393	-1.135571959	0.111084227	0.599149658	0.321238333	1.117489026
genus.Collinsella.id.815	oleoylcarnitine	Simple mode	3	-0.543547633	0.445704143	0.346944871	-1.417127753	0.330032487	0.580684538	0.242409277	1.391013317

genus.Collinsella.id.815	oleoylcarnitine	Weighted mode	3	-0.543547633	0.437241794	0.339785151	-1.400541549	0.313446283	0.580684538	0.246463456	1.368131969
genus.Collinsella.id.815	epiandrosterone sulfate	MR Egger	3	0.350123844	0.134145282	0.232929647	0.087199091	0.613048598	1.419243303	1.091113889	1.846050695
genus.Collinsella.id.815	epiandrosterone sulfate	Weighted median	3	0.195211388	0.083518632	0.019421544	0.031514869	0.358907908	1.215567916	1.032016721	1.431764941
genus.Collinsella.id.815	epiandrosterone sulfate	Inverse variance weighted	3	0.152922621	0.097083365	0.115217643	-0.037360774	0.343206017	1.165234811	0.963328529	1.409459104
genus.Collinsella.id.815	epiandrosterone sulfate	Simple mode	3	0.195092268	0.099693878	0.189495247	-0.000307732	0.390492269	1.215423126	0.999692315	1.477708045
genus.Collinsella.id.815	epiandrosterone sulfate	Weighted mode	3	0.195092268	0.088680634	0.15881544	0.021278225	0.368906312	1.215423126	1.02150622	1.44615211
genus.Collinsella.id.815	X-12798	MR Egger	6	0.150908129	0.110939683	0.245355262	-0.066533649	0.368349907	1.162889817	0.935631432	1.445347687
genus.Collinsella.id.815	X-12798	Weighted median	6	0.111096374	0.080515236	0.16764268	-0.046713488	0.268906237	1.1175026	0.954360794	1.308532443
genus.Collinsella.id.815	X-12798	Inverse variance weighted	6	0.051061707	0.077419764	0.509546783	-0.100681031	0.202804444	1.052387831	0.904221406	1.224832922
genus.Collinsella.id.815	X-12798	Simple mode	6	-0.345037507	0.171304171	0.100110809	-0.680793681	-0.009281333	0.708193791	0.506215059	0.990761606
genus.Collinsella.id.815	X-12798	Weighted mode	6	0.104770465	0.075880092	0.225889344	-0.043954514	0.253495444	1.110455693	0.956997486	1.28852151
genus.Collinsella.id.815	X-12850	MR Egger	4	2.229379298	2.004910593	0.381906403	-1.700245465	6.159004061	9.294095426	0.182638687	472.9568042
genus.Collinsella.id.815	X-12850	Weighted median	4	0.179830067	0.170115054	0.290462079	-0.153595438	0.513255572	1.197013933	0.85761891	1.670721505
genus.Collinsella.id.815	X-12850	Inverse variance weighted	4	0.102980542	0.152359897	0.499102105	-0.195644855	0.401605939	1.10846984	0.82230422	1.494222402
genus.Collinsella.id.815	X-12850	Simple mode	4	0.192240029	0.237818431	0.478045872	-0.273884095	0.658364153	1.211961388	0.760420207	1.931629898
genus.Collinsella.id.815	X-12850	Weighted mode	4	0.191025358	0.218610732	0.446533237	-0.237451677	0.619502393	1.210490147	0.788634999	1.858003256
genus.Collinsella.id.815	4-androsten-3beta,17beta-diol disulfate 1*	MR Egger	5	-0.246322037	0.168551278	0.24005454	-0.576682542	0.084038468	0.781670458	0.56175889	1.087670733
genus.Collinsella.id.815	4-androsten-3beta,17beta-diol disulfate 1*	Weighted median	5	0.063579479	0.064077827	0.321088866	-0.062013061	0.189172019	1.065644179	0.939870611	1.208248777
genus.Collinsella.id.815	4-androsten-3beta,17beta-diol disulfate 1*	Inverse variance weighted	5	0.088275222	0.057540152	0.124992764	-0.024503476	0.20105392	1.092288703	0.975794297	1.222690698
genus.Collinsella.id.815	4-androsten-3beta,17beta-diol disulfate 1*	Simple mode	5	0.060887147	0.067359213	0.417156165	-0.071136912	0.192911205	1.062778969	0.931334373	1.2127751
genus.Collinsella.id.815	4-androsten-3beta,17beta-diol disulfate 1*	Weighted mode	5	0.060887147	0.058269538	0.355045126	-0.053321148	0.175095441	1.062778969	0.948075491	1.191359916
genus.Collinsella.id.815	propionylcarnitine	MR Egger	8	1.332982176	0.588409842	0.064071375	0.179698886	2.486265467	3.792335955	1.196856918	12.01631688
genus.Collinsella.id.815	propionylcarnitine	Weighted median	8	0.505656112	0.306859726	0.099385254	-0.095788951	1.107101175	1.658073045	0.908655766	3.025575057
genus.Collinsella.id.815	propionylcarnitine	Inverse variance weighted	8	0.079124658	0.240189471	0.741833172	-0.391646704	0.549896021	1.082339236	0.67594288	1.733072805
genus.Collinsella.id.815	propionylcarnitine	Simple mode	8	0.450779057	0.578328022	0.461248259	-0.682743867	1.584301981	1.569534466	0.505228808	4.875886727
genus.Collinsella.id.815	propionylcarnitine	Weighted mode	8	0.50218983	0.32581141	0.16713261	-0.136400534	1.140780194	1.652335646	0.8724931	3.129208803
genus.Collinsella.id.815	X-12092	MR Egger	4	0.061218266	0.062898592	0.433070434	-0.062062974	0.184499505	1.063130934	0.9398237	1.202616386
genus.Collinsella.id.815	X-12092	Weighted median	4	0.017478661	0.047111268	0.710631508	-0.074859424	0.109816747	1.017632307	0.927873914	1.116073528
genus.Collinsella.id.815	X-12092	Inverse variance weighted	4	0.013756461	0.046801359	0.768809147	-0.077974203	0.105487125	1.013851517	0.924988288	1.111251797
genus.Collinsella.id.815	X-12092	Simple mode	4	-0.289601629	0.174423525	0.195430614	-0.631471737	0.05226848	0.748561714	0.531808542	1.053658591
genus.Collinsella.id.815	X-12092	Weighted mode	4	0.024238447	0.046895546	0.640942412	-0.067676823	0.116153718	1.024534586	0.934562454	1.12316851
genus.Collinsella.id.815	acetylcarnitine	MR Egger	7	2.64912168	0.924429749	0.035171803	0.837239373	4.461003987	14.14161233	2.309981169	86.57438506
genus.Collinsella.id.815	acetylcarnitine	Weighted median	7	0.430643051	0.330827373	0.19301373	-0.217778601	1.079064702	1.538246376	0.804303494	2.941926686
genus.Collinsella.id.815	acetylcarnitine	Inverse variance weighted	7	0.026796337	0.288701098	0.926049035	-0.539057815	0.59265049	1.027158588	0.583297568	1.808776211
genus.Collinsella.id.815	acetylcarnitine	Simple mode	7	-0.743881652	0.621025068	0.276150955	-1.961090784	0.473327481	0.475265515	0.140704859	1.605327011
genus.Collinsella.id.815	acetylcarnitine	Weighted mode	7	0.577727137	0.423489951	0.221456019	-0.252313167	1.40776744	1.781983619	0.777001369	4.086821137
genus.Collinsella.id.815	X-11423	Inverse variance weighted	2	-0.961080041	1.693465139	0.570359055	-4.280271714	2.358111632	0.382479569	0.013838901	10.57097071
genus.Collinsella.id.815	X-11440	MR Egger	5	0.041147796	0.109591405	0.732289934	-0.173651358	0.25594695	1.042006098	0.840589912	1.291684202
genus.Collinsella.id.815	X-11440	Weighted median	5	0.067754959	0.066669179	0.309493177	-0.062916633	0.19842655	1.070103057	0.939021754	1.219482453
genus.Collinsella.id.815	X-11440	Inverse variance weighted	5	0.06317107	0.058309426	0.278641275	-0.051115404	0.177457545	1.065209049	0.950169011	1.194177358
genus.Collinsella.id.815	X-11440	Simple mode	5	0.064887418	0.072565964	0.421763277	-0.077341871	0.207116707	1.067038888	0.925573373	1.230126128
genus.Collinsella.id.815	X-11440	Weighted mode	5	0.064887418	0.06955888	0.403709957	-0.071447987	0.201222823	1.067038888	0.931044703	1.222897231
genus.Collinsella.id.815	X-12442	Wald ratio	1	0.213395456	0.279527236	0.445215571	-0.334477926	0.761268838	1.237874079	0.715711643	2.140991068
genus.Collinsella.id.815	3-dehydrocarnitine*	MR Egger	6	-0.98271538	3.13816038	0.769815234	-7.133509724	5.168078965	0.374293569	0.000797914	175.5772234
genus.Collinsella.id.815	3-dehydrocarnitine*	Weighted median	6	-0.452124884	0.28201291	0.108889108	-1.004870188	0.100620421	0.636274704	0.366092155	1.105856802
genus.Collinsella.id.815	3-dehydrocarnitine*	Inverse variance weighted	6	-0.324369816	0.230332224	0.159051322	-0.775820974	0.127081343	0.722982823	0.46032571	1.13550938
genus.Collinsella.id.815	3-dehydrocarnitine*	Simple mode	6	-0.470099632	0.362715396	0.25155343	-1.181021809	0.240822545	0.624940001	0.306964919	1.27229524
genus.Collinsella.id.815	3-dehydrocarnitine*	Weighted mode	6	-0.464022965	0.323574119	0.211013195	-1.098228239	0.170182309	0.628749115	0.333461375	1.185520962
genus.Collinsella.id.815	palmitoylcarnitine	MR Egger	3	3.220976884	29.99271718	0.931893176	-55.56474878	62.00670255	25.05258171	7.39E-25	8.50E+26
genus.Collinsella.id.815	palmitoylcarnitine	Weighted median	3	-0.579469623	0.423194362	0.17091321	-1.408930573	0.249991327	0.560195402	0.244404516	1.284014281
genus.Collinsella.id.815	palmitoylcarnitine	Inverse variance weighted	3	-0.561017458	0.348217672	0.107155446	-1.243524095	0.121489179	0.570628178	0.288366195	1.129177147
genus.Collinsella.id.815	palmitoylcarnitine	Simple mode	3	-0.593330102	0.484073645	0.345054087	-1.542114447	0.355454243	0.552484388	0.213928283	1.426828633
genus.Collinsella.id.815	palmitoylcarnitine	Weighted mode	3	-0.592875953	0.48174356	0.343536746	-1.537093331	0.351341426	0.552735356	0.215005143	1.4209724
genus.Collinsella.id.815	betaine	MR Egger	4	-0.364196544	1.541628619	0.83523515	-3.385788638	2.65739555	0.694754632	0.033850936	14.25910357
genus.Collinsella.id.815	betaine	Weighted median	4	-0.436419983	0.405306276	0.281584856	-1.230820284	0.357980317	0.646346214	0.292052913	1.430437465
genus.Collinsella.id.815	betaine	Inverse variance weighted	4	-0.376674374	0.361747678	0.297753629	-1.085699823	0.332351074	0.686139462	0.337665397	1.394242245
genus.Collinsella.id.815	betaine	Simple mode	4	-0.696780658	0.699503902	0.392615142	-2.067808307	0.67424699	0.498186558	0.126462646	1.962554597
genus.Collinsella.id.815	betaine	Weighted mode	4	-0.752628055	0.665562281	0.340364577	-2.057130125	0.551874015	0.471126777	0.127820272	1.736504205
genus.Collinsella.id.815	stearoylcarnitine	MR Egger	3	-6.209256604	56.89153424	0.93079197	-117.7166637	105.2981505	0.002010732	7.52E-52	5.38E+45
genus.Collinsella.id.815	stearoylcarnitine	Weighted median	3	-0.552692911	0.385330091	0.151476336	-1.307939889	0.202554068	0.575398226	0.270376488	1.224526291
genus.Collinsella.id.815	stearoylcarnitine	Inverse variance weighted	3	-0.538062066	0.333562109	0.106727324	-1.191843801	0.115719668	0.583878675	0.303660858	1.122681104
genus.Collinsella.id.815	stearoylcarnitine	Simple mode	3	-0.5613234	0.438164162	0.328639111	-1.420125159	0.297478358	0.570453626	0.241683766	1.346459235
genus.Collinsella.id.815	stearoylcarnitine	Weighted mode	3	-0.5613234	0.445171143	0.334505579	-1.433858841	0.31121204	0.570453626	0.238387247	1.365078642
family.Desulfovibrionaceae.id.3169	succinylcarnitine	MR Egger	6	0.268471007	0.53157202	0.640079288	-0.773410153	1.310352167	1.307963054	0.461436812	3.707479134
family.Desulfovibrionaceae.id.3169	succinylcarnitine	Weighted median	6	0.075077269	0.293056105	0.79780617	-0.499312696	0.649467234	1.077967441	0.606947674	1.914520566
family.Desulfovibrionaceae.id.3169	succinylcarnitine	Inverse variance weighted	6	-0.177525845	0.258749667	0.492655659	-0.684675194	0.329623503	0.837339358	0.504253988	1.390444531
family.Desulfovibrionaceae.id.3169	succinylcarnitine	Simple mode	6	-0.082894719	0.695591563	0.909779485	-1.446254182	1.280464743	0.920448047	0.235450593	3.598311628
family.Desulfovibrionaceae.id.3169	succinylcarnitine	Weighted mode	6	0.149320655	0.324204838	0.664441594	-0.486120828	0.784762138	1.161045224	0.615007492	2.191885513
family.Desulfovibrionaceae.id.3169	lysine	Wald ratio	1	0.641318269	0.911105259	0.481501014	-1.144448039	2.427084576	1.898982599	0.318399613	11.32581435
family.Desulfovibrionaceae.id.3169	oleoylcarnitine	MR Egger	3	3.997709591	15.08688859	0.835098821	-25.57259204	33.56801122	54.47324106	7.83E-12	3.78793E+14
family.Desulfovibrionaceae.id.3169	oleoylcarnitine	Weighted median	3	0.828269807	0.368124302	0.024450648	0.106746176	1.549793439	2.289354288	1.112651801	4.710497075
family.Desulfovibrionaceae.id.3169	oleoylcarnitine	Inverse variance weighted	3	0.881007934	0.309054166	0.004362878	0.275261768	1.486754099	2.413330959	1.316875346	4.422716496
family.Desulfovibrionaceae.id.3169	oleoylcarnitine	Simple mode	3	0.816650154	0.424365082	0.194191162	-0.015105407	1.648405715	2.262906738	0.985008107	5.198685032
family.Desulfovibrionaceae.id.3169	oleoylcarnitine	Weighted mode	3	0.81606615	0.398174803	0.176928637	0.035643535	1.596488764	2.261585577	1.036286381	4.935671656
family.Desulfovibrionaceae.id.3169	epiandrosterone sulfate	MR Egger	3	0.130583632	0.131494465	0.502212157	-0.12714552	0.388312783	1.139493234	0.880605518	1.474490908
family.Desulfovibrionaceae.id.3169	epiandrosterone sulfate	Weighted median	3	0.135449179	0.080099282	0.090833719	-0.021545414	0.292443772	1.145051002	0.978685031	1.339697406
family.Desulfovibrionaceae.id.3169	epiandrosterone sulfate	Inverse variance weighted	3	0.136167312	0.074981941	0.069370153	-0.010797293	0.283131917	1.145873596	0.989260789	1.327280241
family.Desulfovibrionaceae.id.3169	epiandrosterone sulfate	Simple mode	3	0.13532272	0.099479855	0.306763657	-0.059657795	0.330303236	1.144906209	0.942086865	1.391389983
family.Desulfovibrionaceae.id.3169	epiandrosterone sulfate	Weighted mode	3	0.135124426	0.095320522	0.292053213	-0.051703797	0.321952649	1.144679204	0.949610103	1.379819439
family.Desulfovibrionaceae.id.3169	X-12798	MR Egger	6	-0.134758456	0.10537583	0.270108422	-0.341295083	0.071778172	0.873926978	0.710849118	1.074416982
family.Desulfovibrionaceae.id.3169	X-12798	Weighted median	6	-0.133596452	0.075676382	0.077502406	-0.28192216	0.014729256	0.874943075	0.754332399	1.014838266
family.Desulfovibrionaceae.id.3169	X-12798	Inverse variance weighted	6	-0.170722397	0.068944359	0.013277636	-0.305853342	-0.035591453	0.843055575	0.736494625	0.965034475
family.Desulfovibrionaceae.id.3169	X-12798	Simple mode	6	-0.457036527	0.130247945	0.017119434	-0.712322498	-0.201750555	0.633157213	0.49050368	0.817298773
family.Desulfovibrionaceae.id.3169	X-12798	Weighted mode	6	-0.139898482	0.077732941	0.131803004	-0.292255046	0.012458081	0.869446495	0.7465781	1.012536006
family.Desulfovibrionaceae.id.3169	X-12850	MR Egger	4	-1.556161931	1.938283052	0.506304197	-5.355196713	2.242872851	0.210944138	0.00472354	9.420355729
family.Desulfovibrionaceae.id.3169	X-12850	Weighted median	4	0.106708944	0.164017701	0.515308559	-0.21476575	0.428183638	1.112610375	0.806730394	1.534467842
family.Desulfovibrionaceae.id.3169	X-12850	Inverse variance weighted	4	0.165325269	0.148126434	0.264375414	-0.125002541	0.45565308	1.179776801	0.88249466	1.577203086
family.Desulfovibrionaceae.id.3169	X-12850	Simple mode	4	0.101467253	0.240507747	0.701514867	-0.369927932	0.572862437	1.106793673	0.690784113	1.773335856
family.Desulfovibrionaceae.id.3169	X-12850	Weighted mode	4	0.101467253	0.217045537	0.672010357	-0.323942	0.526876506	1.106793673	0.723292192	1.693633982
family.Desulfovibrionaceae.id.3169	4-androsten-3beta,17beta-diol disulfate 1*	MR Egger	5	-0.181918162	0.164642596	0.349848549	-0.504617649	0.140781325	0.833669563	0.60373637	1.151172888
family.Desulfovibrionaceae.id.3169	4-androsten-3beta,17beta-diol disulfate 1*	Weighted median	5	0.036294322	0.062354168	0.560521353	-0.085919847	0.158508491	1.036961002	0.917667782	1.171761873
family.Desulfovibrionaceae.id.3169	4-androsten-3beta,17beta-diol disulfate 1*	Inverse variance weighted	5	0.05435029	0.053017344	0.305296336	-0.049563704	0.158264284	1.055854392	0.951644532	1.171475756
family.Desulfovibrionaceae.id.3169	4-androsten-3beta,17beta-diol disulfate 1*	Simple mode	5	0.035038832	0.061209251	0.597659809	-0.0849313	0.155008964	1.035659925	0.918575389	1.167668428
family.Desulfovibrionaceae.id.3169	4-androsten-3beta,17beta-diol disulfate 1*	Weighted mode	5	0.035038832	0.061746789	0.600734572	-0.085984873	0.156062538	1.035659925	0.917608111	1.168899301
family.Desulfovibrionaceae.id.3169	propionylcarnitine	MR Egger	8	0.561632506	0.573986192	0.365626466	-0.563380431	1.686645443	1.753532817	0.569281391	5.401331205
family.Desulfovibrionaceae.id.3169	propionylcarnitine	Weighted median	8	0.681131604	0.259059808	0.008557478	0.17337438	1.188888829	1.976112645	1.189311276	3.283430725
family.Desulfovibrionaceae.id.3169	propionylcarnitine	Inverse variance weighted	8	0.798766499	0.210712878	0.000150174	0.385769259	1.211763739	2.222797414	1.47074527	3.359404545
family.Desulfovibrionaceae.id.3169	propionylcarnitine	Simple mode	8	0.716554495	0.348299105	0.078678463	0.033888249	1.399220741	2.047366832	1.034468998	4.052041148
family.Desulfovibrionaceae.id.3169	propionylcarnitine	Weighted mode	8	0.691807401	0.329205162	0.073713464	0.046565283	1.337049518	1.997322235	1.047666472	3.807792094
family.Desulfovibrionaceae.id.3169	X-12092	MR Egger	4	-0.049083733	0.061150494	0.506390139	-0.168938701	0.070771234	0.952101404	0.844560673	1.073335655
family.Desulfovibrionaceae.id.3169	X-12092	Weighted median	4	-0.076976424	0.045286946	0.089178218	-0.165738839	0.01178599	0.925911682	0.847267479	1.011855718
family.Desulfovibrionaceae.id.3169	X-12092	Inverse variance weighted	4	-0.080515894	0.045495085	0.076765254	-0.169686262	0.008654473	0.922640239	0.843929548	1.008692031
family.Desulfovibrionaceae.id.3169	X-12092	Simple mode	4	-0.284611977	0.144211071	0.142947718	-0.567265676	-0.001958279	0.75230611	0.567073885	0.998043637
family.Desulfovibrionaceae.id.3169	X-12092	Weighted mode	4	-0.074201247	0.047135851	0.213509143	-0.166587515	0.018185021	0.92848482	0.846548728	1.018351376
family.Desulfovibrionaceae.id.3169	acetylcarnitine	MR Egger	7	0.45382542	0.902105633	0.636284942	-1.314301621	2.221952461	1.574323128	0.268661885	9.225325382
family.Desulfovibrionaceae.id.3169	acetylcarnitine	Weighted median	7	0.800242388	0.287838717	0.005432939	0.236078503	1.364406273	2.226080438	1.266273712	3.913398872
family.Desulfovibrionaceae.id.3169	acetylcarnitine	Inverse variance weighted	7	0.905693257	0.233778653	0.000106998	0.447487098	1.363899417	2.473646202	1.564376118	3.911415844
family.Desulfovibrionaceae.id.3169	acetylcarnitine	Simple mode	7	0.786641775	0.410136784	0.103553092	-0.017226323	1.590509872	2.196009335	0.982921202	4.906249851
family.Desulfovibrionaceae.id.3169	acetylcarnitine	Weighted mode	7	0.781727794	0.379995749	0.085386564	0.036936127	1.526519462	2.185244659	1.037626742	4.602131022
family.Desulfovibrionaceae.id.3169	X-11423	Inverse variance weighted	2	0.603556715	0.585275859	0.30243082	-0.543583969	1.750697399	1.828611096	0.580663439	5.758617329
family.Desulfovibrionaceae.id.3169	X-11440	MR Egger	5	0.030757348	0.106713309	0.791941353	-0.178400739	0.239915434	1.031235242	0.836607095	1.27114165
family.Desulfovibrionaceae.id.3169	X-11440	Weighted median	5	0.037962637	0.063564553	0.550354056	-0.086623887	0.16254916	1.038692423	0.917021935	1.176506154
family.Desulfovibrionaceae.id.3169	X-11440	Inverse variance weighted	5	0.033736321	0.056776163	0.552379235	-0.077544958	0.145017601	1.034311845	0.92538542	1.156059917
family.Desulfovibrionaceae.id.3169	X-11440	Simple mode	5	0.037263291	0.075711715	0.64838598	-0.11113167	0.185658251	1.037966272	0.89482092	1.204010721
family.Desulfovibrionaceae.id.3169	X-11440	Weighted mode	5	0.037263291	0.068025962	0.612974892	-0.096067596	0.170594177	1.037966272	0.90840261	1.186009342
family.Desulfovibrionaceae.id.3169	X-12442	Wald ratio	1	-0.189832073	0.271973655	0.485189928	-0.722900437	0.343236291	0.827098014	0.485342507	1.409501775
family.Desulfovibrionaceae.id.3169	3-dehydrocarnitine*	MR Egger	6	4.285672947	3.350594074	0.270031376	-2.281491438	10.85283733	72.65142084	0.10213177	51680.57849
family.Desulfovibrionaceae.id.3169	3-dehydrocarnitine*	Weighted median	6	0.691882846	0.274792457	0.011807767	0.153289631	1.230476061	1.997472929	1.165662542	3.422858639
family.Desulfovibrionaceae.id.3169	3-dehydrocarnitine*	Inverse variance weighted	6	0.468983669	0.281088032	0.095224777	-0.081948868	1.019916212	1.598368895	0.921319061	2.772962413
family.Desulfovibrionaceae.id.3169	3-dehydrocarnitine*	Simple mode	6	0.690983489	0.314595317	0.079451334	0.074376667	1.307590311	1.995677295	1.077212479	3.697253739
family.Desulfovibrionaceae.id.3169	3-dehydrocarnitine*	Weighted mode	6	0.70853831	0.279853155	0.052413445	0.160026127	1.257050493	2.031020366	1.173541532	3.515038552
family.Desulfovibrionaceae.id.3169	palmitoylcarnitine	MR Egger	3	9.598277605	29.15439991	0.797525601	-47.54434623	66.74090144	14739.37266	2.25E-21	9.67E+28
family.Desulfovibrionaceae.id.3169	palmitoylcarnitine	Weighted median	3	0.898685953	0.413816698	0.029878257	0.087605224	1.709766681	2.456373199	1.091557117	5.527671619
family.Desulfovibrionaceae.id.3169	palmitoylcarnitine	Inverse variance weighted	3	0.964249505	0.338397593	0.004379388	0.300990223	1.627508787	2.622818506	1.351196131	5.091175705
family.Desulfovibrionaceae.id.3169	palmitoylcarnitine	Simple mode	3	0.891153114	0.449441109	0.185863912	0.010248541	1.772057688	2.437939253	1.010301237	5.882946182
family.Desulfovibrionaceae.id.3169	palmitoylcarnitine	Weighted mode	3	0.891153114	0.448189145	0.185099219	0.012702391	1.769603838	2.437939253	1.012783409	5.868528011
family.Desulfovibrionaceae.id.3169	betaine	MR Egger	4	1.025896884	1.146055696	0.465167171	-1.22037228	3.272166049	2.789596284	0.295120279	26.36839275
family.Desulfovibrionaceae.id.3169	betaine	Weighted median	4	-0.009404703	0.368241946	0.979624669	-0.731158917	0.712349511	0.990639383	0.481350821	2.038775762
family.Desulfovibrionaceae.id.3169	betaine	Inverse variance weighted	4	0.012556278	0.328043533	0.969467422	-0.630409047	0.655521604	1.012635439	0.532373991	1.92614694

family.Desulfovibrionaceae.id.3169	betaine	Simple mode	4	0.024632232	0.532946	0.96604029	-1.019941927	1.069206391	1.024938112	0.360615881	2.913066747
family.Desulfovibrionaceae.id.3169	betaine	Weighted mode	4	0.036693575	0.453903401	0.94066019	-0.852957091	0.92634424	1.037375094	0.426152894	2.525260537
family.Desulfovibrionaceae.id.3169	stearoylcarnitine	MR Egger	3	-16.98691539	55.28927304	0.810234448	-125.3538905	91.38005977	4.19E-08	3.63E-55	4.85E+39
family.Desulfovibrionaceae.id.3169	stearoylcarnitine	Weighted median	3	0.845715139	0.412333039	0.040262206	0.037542382	1.653887896	2.329643238	1.038255999	5.227263429
family.Desulfovibrionaceae.id.3169	stearoylcarnitine	Inverse variance weighted	3	0.921876895	0.324150552	0.004455419	0.286541813	1.557211978	2.514004487	1.331813853	4.745572023
family.Desulfovibrionaceae.id.3169	stearoylcarnitine	Simple mode	3	0.843063766	0.447370529	0.200172917	-0.033782472	1.719910003	2.323474665	0.966781784	5.584025895
family.Desulfovibrionaceae.id.3169	stearoylcarnitine	Weighted mode	3	0.843063766	0.426508541	0.186716541	0.007107025	1.679020507	2.323474665	1.007132339	5.360303018
genus.Streptococcus.id.1853	succinylcarnitine	MR Egger	6	0.933295078	0.492401653	0.130932813	-0.031812162	1.898402317	2.542874357	0.968688522	6.675221033
genus.Streptococcus.id.1853	succinylcarnitine	Weighted median	6	0.361803375	0.277037442	0.191561794	-0.181190012	0.904796761	1.435916577	0.834276821	2.471429582
genus.Streptococcus.id.1853	succinylcarnitine	Inverse variance weighted	6	0.284684787	0.241425995	0.238326373	-0.188510164	0.757879738	1.329342936	0.828192086	2.133747317
genus.Streptococcus.id.1853	succinylcarnitine	Simple mode	6	-0.404866959	0.604462763	0.532670934	-1.589613975	0.779880057	0.667065552	0.204004347	2.181210629
genus.Streptococcus.id.1853	succinylcarnitine	Weighted mode	6	0.593621879	0.295959852	0.101193625	0.01354057	1.173703188	1.81053409	1.013632659	3.233946404
genus.Streptococcus.id.1853	lysine	Wald ratio	1	0.458628867	0.873903254	0.5997187	-1.254221511	2.171479245	1.581903498	0.285297863	8.771249279
genus.Streptococcus.id.1853	oleoylcarnitine	MR Egger	3	2.933288533	14.50811533	0.872998599	-25.50261751	31.36919457	18.78931831	8.40E-12	4.20212E+13
genus.Streptococcus.id.1853	oleoylcarnitine	Weighted median	3	-0.440342902	0.359938516	0.221184819	-1.145822393	0.265136589	0.643815618	0.31796232	1.303609022
genus.Streptococcus.id.1853	oleoylcarnitine	Inverse variance weighted	3	-0.453172066	0.297552007	0.127758403	-1.036374	0.130029867	0.635608757	0.354738635	1.138862398
genus.Streptococcus.id.1853	oleoylcarnitine	Simple mode	3	-0.417607567	0.37778397	0.384162474	-1.158064148	0.322849015	0.658620642	0.314093631	1.381056816
genus.Streptococcus.id.1853	oleoylcarnitine	Weighted mode	3	-0.417607567	0.40042825	0.406487397	-1.202446937	0.367231804	0.658620642	0.30045811	1.443732543
genus.Streptococcus.id.1853	epiandrosterone sulfate	MR Egger	3	0.13243277	0.126436455	0.485256302	-0.115382683	0.380248222	1.141602263	0.891025099	1.462647606
genus.Streptococcus.id.1853	epiandrosterone sulfate	Weighted median	3	0.080775732	0.076839055	0.293151701	-0.069828816	0.23138028	1.084127734	0.932553445	1.260338429
genus.Streptococcus.id.1853	epiandrosterone sulfate	Inverse variance weighted	3	0.067433091	0.072021987	0.349126573	-0.073730003	0.208596185	1.069758681	0.928922467	1.231947419
genus.Streptococcus.id.1853	epiandrosterone sulfate	Simple mode	3	0.080969148	0.09587007	0.487271442	-0.10693619	0.268874486	1.084337442	0.89858301	1.308490897
genus.Streptococcus.id.1853	epiandrosterone sulfate	Weighted mode	3	0.081342514	0.090325854	0.462873586	-0.09569616	0.258381188	1.084742373	0.908740086	1.294832299
genus.Streptococcus.id.1853	X-12798	MR Egger	6	0.102006838	0.098874946	0.360516408	-0.091788055	0.295801732	1.107391044	0.912298486	1.344203617
genus.Streptococcus.id.1853	X-12798	Weighted median	6	0.114523369	0.075818823	0.130919125	-0.034081523	0.263128262	1.121338846	0.96649271	1.300993576
genus.Streptococcus.id.1853	X-12798	Inverse variance weighted	6	0.13499682	0.066032138	0.040912975	0.00557383	0.264419811	1.144533145	1.005589393	1.302674958
genus.Streptococcus.id.1853	X-12798	Simple mode	6	0.174730158	0.104986847	0.156934874	-0.031044063	0.380504379	1.190924812	0.969432856	1.463022322
genus.Streptococcus.id.1853	X-12798	Weighted mode	6	0.113821198	0.073599355	0.182653334	-0.030433538	0.258075934	1.12055175	0.9700249	1.294437106
genus.Streptococcus.id.1853	X-12850	MR Egger	4	-1.335811313	1.867242502	0.548608854	-4.995606616	2.323983991	0.262944758	0.006767615	10.21629496
genus.Streptococcus.id.1853	X-12850	Weighted median	4	-0.338243345	0.163581523	0.038664889	-0.65886313	-0.017623559	0.713021757	0.517439261	0.982530827
genus.Streptococcus.id.1853	X-12850	Inverse variance weighted	4	-0.305629895	0.142035882	0.031414727	-0.584020224	-0.027239567	0.73665921	0.557651969	0.973128084
genus.Streptococcus.id.1853	X-12850	Simple mode	4	-0.351879056	0.209024005	0.190879385	-0.761566106	0.057807994	0.703365185	0.466934585	1.059511544
genus.Streptococcus.id.1853	X-12850	Weighted mode	4	-0.351879056	0.21025402	0.192804684	-0.763976936	0.060218824	0.703365185	0.465810241	1.062068927
genus.Streptococcus.id.1853	4-androsten-3beta,17beta-diol disulfate 1*	MR Egger	5	-0.324723246	0.157979493	0.132067681	-0.634363051	-0.015083441	0.722727344	0.530273138	0.985029745
genus.Streptococcus.id.1853	4-androsten-3beta,17beta-diol disulfate 1*	Weighted median	5	-0.116890054	0.056948837	0.040116722	-0.228509775	-0.005270333	0.889683005	0.795718519	0.994743531
genus.Streptococcus.id.1853	4-androsten-3beta,17beta-diol disulfate 1*	Inverse variance weighted	5	-0.094742156	0.05077395	0.062046972	-0.194259099	0.004774786	0.909607441	0.823444523	1.004786203
genus.Streptococcus.id.1853	4-androsten-3beta,17beta-diol disulfate 1*	Simple mode	5	-0.11512177	0.063280291	0.143004001	-0.239151141	0.0089076	0.891257608	0.787295881	1.008947391
genus.Streptococcus.id.1853	4-androsten-3beta,17beta-diol disulfate 1*	Weighted mode	5	-0.11512177	0.058360056	0.119810509	-0.22950748	-0.00073606	0.891257608	0.794925022	0.99926421
genus.Streptococcus.id.1853	propionylcarnitine	MR Egger	8	-0.478578911	0.550565583	0.418121224	-1.557687454	0.600529633	0.619663363	0.210622583	1.82308411
genus.Streptococcus.id.1853	propionylcarnitine	Weighted median	8	-0.431193898	0.246003339	0.07963687	-0.913360444	0.050972647	0.649732916	0.401173834	1.052294109
genus.Streptococcus.id.1853	propionylcarnitine	Inverse variance weighted	8	-0.509390175	0.202211623	0.011765709	-0.905724955	-0.113055395	0.600861888	0.404248717	0.893101186
genus.Streptococcus.id.1853	propionylcarnitine	Simple mode	8	-0.67186367	0.324041341	0.076831415	-1.306984699	-0.036742641	0.51075581	0.270634873	0.963924178
genus.Streptococcus.id.1853	propionylcarnitine	Weighted mode	8	-0.402208773	0.29815868	0.219350364	-0.986599787	0.18218224	0.668841095	0.372842281	1.199832832
genus.Streptococcus.id.1853	X-12092	MR Egger	4	0.051570018	0.058782681	0.472848917	-0.063644036	0.166784073	1.052922908	0.938338955	1.18149912
genus.Streptococcus.id.1853	X-12092	Weighted median	4	0.003517004	0.045547987	0.938452226	-0.085757051	0.092791059	1.003523196	0.917817187	1.097232454
genus.Streptococcus.id.1853	X-12092	Inverse variance weighted	4	-0.006394681	0.043726613	0.883730096	-0.092098842	0.07930948	0.993625721	0.912014999	1.082539294
genus.Streptococcus.id.1853	X-12092	Simple mode	4	-0.07219169	0.185911238	0.723701331	-0.436577718	0.292194337	0.930352539	0.646244271	1.33936328
genus.Streptococcus.id.1853	X-12092	Weighted mode	4	0.001866293	0.044563134	0.969225969	-0.085477449	0.089210035	1.001868036	0.918073846	1.093310266
genus.Streptococcus.id.1853	acetylcarnitine	MR Egger	7	-0.342823802	0.865442486	0.708351883	-2.039091075	1.35344347	0.709763259	0.130146951	3.870731357
genus.Streptococcus.id.1853	acetylcarnitine	Weighted median	7	-0.463910359	0.280378143	0.098008064	-1.013451519	0.085630801	0.62881992	0.362964038	1.089404047
genus.Streptococcus.id.1853	acetylcarnitine	Inverse variance weighted	7	-0.575408466	0.224402223	0.010341887	-1.015236823	-0.135580109	0.56247507	0.362316615	0.873209208
genus.Streptococcus.id.1853	acetylcarnitine	Simple mode	7	-0.456928553	0.381844407	0.276582513	-1.20534359	0.291486484	0.633225581	0.299589046	1.338415543
genus.Streptococcus.id.1853	acetylcarnitine	Weighted mode	7	-0.434614648	0.376656342	0.292433658	-1.172861078	0.303631782	0.64751414	0.309480226	1.354770114
genus.Streptococcus.id.1853	X-11423	Inverse variance weighted	2	-0.880994682	0.560411365	0.115938958	-1.979400958	0.217411594	0.41437054	0.138151971	1.242855549
genus.Streptococcus.id.1853	X-11440	MR Egger	5	-0.163754751	0.102417287	0.20814085	-0.364492633	0.036983132	0.848950201	0.694548953	1.037675517
genus.Streptococcus.id.1853	X-11440	Weighted median	5	-0.125668367	0.064934572	0.052952567	-0.252940127	0.001603394	0.881907268	0.776514372	1.00160468
genus.Streptococcus.id.1853	X-11440	Inverse variance weighted	5	-0.111241353	0.054377271	0.040782693	-0.217820804	-0.004661901	0.894722779	0.804269551	0.995348949
genus.Streptococcus.id.1853	X-11440	Simple mode	5	-0.122343096	0.069087299	0.151289914	-0.257754202	0.013068009	0.884844729	0.772785158	1.013153768
genus.Streptococcus.id.1853	X-11440	Weighted mode	5	-0.122343096	0.063312129	0.125471501	-0.246434869	0.001748676	0.884844729	0.781582265	1.001750206
genus.Streptococcus.id.1853	X-12442	Wald ratio	1	-0.164344481	0.261372328	0.529496129	-0.676634244	0.347945282	0.848449696	0.508325014	1.416154758
genus.Streptococcus.id.1853	3-dehydrocarnitine*	MR Egger	6	-1.932092675	2.717340324	0.516333641	-7.25807971	3.393894361	0.144844768	0.00070446	29.78170745
genus.Streptococcus.id.1853	3-dehydrocarnitine*	Weighted median	6	-0.402042479	0.262879327	0.126170179	-0.91728596	0.113201002	0.668952329	0.399602107	1.119857003
genus.Streptococcus.id.1853	3-dehydrocarnitine*	Inverse variance weighted	6	-0.235383118	0.214604661	0.272719201	-0.656008253	0.185242017	0.790268026	0.518918597	1.203509675
genus.Streptococcus.id.1853	3-dehydrocarnitine*	Simple mode	6	-0.430818592	0.307136978	0.219647358	-1.032807068	0.171169884	0.649976811	0.356006223	1.186692332
genus.Streptococcus.id.1853	3-dehydrocarnitine*	Weighted mode	6	-0.426020674	0.278379236	0.186483843	-0.971643977	0.119602628	0.65310284	0.378460346	1.127048905
genus.Streptococcus.id.1853	palmitoylcarnitine	MR Egger	3	0.132512026	28.0442364	0.996991926	-54.83419133	55.09921538	1.141692746	1.53E-24	8.50E+23
genus.Streptococcus.id.1853	palmitoylcarnitine	Weighted median	3	-0.488644397	0.380544849	0.199119144	-1.234512301	0.257223507	0.613457436	0.290976637	1.293334164
genus.Streptococcus.id.1853	palmitoylcarnitine	Inverse variance weighted	3	-0.49759023	0.325794929	0.126683656	-1.13614829	0.14096783	0.607994021	0.321053247	1.151387607
genus.Streptococcus.id.1853	palmitoylcarnitine	Simple mode	3	-0.466059577	0.445025306	0.404882631	-1.338309177	0.406190024	0.6274699	0.262288778	1.501087768
genus.Streptococcus.id.1853	palmitoylcarnitine	Weighted mode	3	-0.470860793	0.421279116	0.379942685	-1.29656786	0.354846273	0.624464502	0.273468767	1.425961429
genus.Streptococcus.id.1853	betaine	MR Egger	4	-0.119241327	1.102150697	0.923721232	-2.279456693	2.040974039	0.887593575	0.102339794	7.698103798
genus.Streptococcus.id.1853	betaine	Weighted median	4	-0.698595971	0.382019138	0.067445515	-1.447353481	0.05016154	0.497283014	0.235191905	1.051440932
genus.Streptococcus.id.1853	betaine	Inverse variance weighted	4	-0.591399315	0.315410236	0.060790489	-1.209603378	0.026804748	0.553552149	0.298315574	1.027167226
genus.Streptococcus.id.1853	betaine	Simple mode	4	-0.737724742	0.583747252	0.2955846	-1.881869355	0.406419872	0.478200709	0.152305127	1.501432829
genus.Streptococcus.id.1853	betaine	Weighted mode	4	-0.852569076	0.532326091	0.207564418	-1.895928213	0.190790062	0.42631828	0.150178872	1.210205357
genus.Streptococcus.id.1853	stearoylcarnitine	MR Egger	3	5.29979434	53.22951087	0.936823187	-99.03004696	109.6296356	200.2956129	9.81E-44	4.09E+47
genus.Streptococcus.id.1853	stearoylcarnitine	Weighted median	3	-0.478948066	0.369765351	0.195225146	-1.203688154	0.245792021	0.619434653	0.300085407	1.278633617
genus.Streptococcus.id.1853	stearoylcarnitine	Inverse variance weighted	3	-0.476524135	0.312094373	0.12679605	-1.088229107	0.135180836	0.620937941	0.336812425	1.144743776
genus.Streptococcus.id.1853	stearoylcarnitine	Simple mode	3	-0.489827381	0.44903515	0.389238867	-1.369936275	0.390281514	0.612732154	0.254123153	1.477396643
genus.Streptococcus.id.1853	stearoylcarnitine	Weighted mode	3	-0.489827381	0.414459142	0.358748015	-1.302167299	0.322512538	0.612732154	0.271941775	1.3805922
genus.Romboutsia.id.11347	succinylcarnitine	MR Egger	6	-0.521491302	0.529130499	0.380144073	-1.558587081	0.515604476	0.593634599	0.210433186	1.674650481
genus.Romboutsia.id.11347	succinylcarnitine	Weighted median	6	-0.077762648	0.284253177	0.784416425	-0.634898875	0.479373578	0.925183994	0.529989081	1.615062375
genus.Romboutsia.id.11347	succinylcarnitine	Inverse variance weighted	6	-0.043565136	0.259444797	0.866648745	-0.552076939	0.464946666	0.957370193	0.575752764	1.591929283
genus.Romboutsia.id.11347	succinylcarnitine	Simple mode	6	0.411253554	0.514129768	0.460065781	-0.596440791	1.418947899	1.508707847	0.550768452	4.132770062
genus.Romboutsia.id.11347	succinylcarnitine	Weighted mode	6	-0.20358687	0.325134625	0.558679839	-0.840850734	0.433676994	0.815799333	0.431343409	1.542920415
genus.Romboutsia.id.11347	lysine	Wald ratio	1	-2.250428891	0.938715405	0.016514164	-4.090311086	-0.410546697	0.105354029	0.016734027	0.663287534
genus.Romboutsia.id.11347	oleoylcarnitine	MR Egger	3	-0.138361262	15.58664076	0.99434893	-30.68817715	30.41145463	0.870784053	4.70E-14	1.6126E+13
genus.Romboutsia.id.11347	oleoylcarnitine	Weighted median	3	-0.467454097	0.370763742	0.207385607	-1.194151031	0.259242836	0.626595491	0.30296105	1.29594847
genus.Romboutsia.id.11347	oleoylcarnitine	Inverse variance weighted	3	-0.478896134	0.319925038	0.134419	-1.105949209	0.148156941	0.619466823	0.330896643	1.159694886
genus.Romboutsia.id.11347	oleoylcarnitine	Simple mode	3	-0.457483336	0.426182905	0.395401908	-1.29280183	0.377835157	0.632874375	0.274500601	1.459122397
genus.Romboutsia.id.11347	oleoylcarnitine	Weighted mode	3	-0.455813115	0.38431893	0.357415198	-1.209078219	0.297451989	0.633932299	0.298472279	1.346423731
genus.Romboutsia.id.11347	epiandrosterone sulfate	MR Egger	3	-0.144082586	0.136263396	0.482248503	-0.411158843	0.12299367	0.86581624	0.662881629	1.130877263
genus.Romboutsia.id.11347	epiandrosterone sulfate	Weighted median	3	-0.167823479	0.083299973	0.043937322	-0.331091426	-0.004555531	0.845503071	0.718139509	0.99545483
genus.Romboutsia.id.11347	epiandrosterone sulfate	Inverse variance weighted	3	-0.172565954	0.077594349	0.026151807	-0.324650878	-0.02048103	0.841502787	0.722779648	0.979727282
genus.Romboutsia.id.11347	epiandrosterone sulfate	Simple mode	3	-0.166088982	0.101372402	0.243000577	-0.364778891	0.032600926	0.846970865	0.694350161	1.033138159
genus.Romboutsia.id.11347	epiandrosterone sulfate	Weighted mode	3	-0.166427149	0.09443435	0.220066173	-0.351518474	0.018664176	0.846684496	0.703618851	1.01883944
genus.Romboutsia.id.11347	X-12798	MR Egger	6	-0.128163344	0.106310291	0.294424356	-0.336531515	0.080204827	0.879709672	0.714243374	1.083508977
genus.Romboutsia.id.11347	X-12798	Weighted median	6	-0.130924001	0.077672201	0.09187352	-0.283161515	0.021313514	0.877284445	0.753398093	1.021542269
genus.Romboutsia.id.11347	X-12798	Inverse variance weighted	6	-0.123991614	0.071003299	0.080762373	-0.26315808	0.015174851	0.883387249	0.768620384	1.015290573
genus.Romboutsia.id.11347	X-12798	Simple mode	6	-0.132994828	0.112873531	0.291708486	-0.35422695	0.088237293	0.87546962	0.701715695	1.092247274
genus.Romboutsia.id.11347	X-12798	Weighted mode	6	-0.13276783	0.082915902	0.17022423	-0.295282998	0.029747338	0.875668373	0.744320916	1.03019421
genus.Romboutsia.id.11347	X-12850	MR Egger	4	2.672986881	1.998902858	0.312947284	-1.244862721	6.590836483	14.48316414	0.287980439	728.3899012
genus.Romboutsia.id.11347	X-12850	Weighted median	4	0.057971536	0.182066007	0.750174377	-0.298877838	0.41482091	1.059684832	0.741650005	1.514099556
genus.Romboutsia.id.11347	X-12850	Inverse variance weighted	4	-0.032019615	0.152365499	0.833550575	-0.330655993	0.266616763	0.968487585	0.718452279	1.30554002
genus.Romboutsia.id.11347	X-12850	Simple mode	4	0.071500536	0.230615327	0.776818513	-0.380505504	0.523506577	1.074118727	0.683515802	1.687936163
genus.Romboutsia.id.11347	X-12850	Weighted mode	4	0.069898625	0.207656446	0.758584242	-0.337108008	0.476905259	1.072399462	0.713831736	1.611080802
genus.Romboutsia.id.11347	4-androsten-3beta,17beta-diol disulfate 1*	MR Egger	5	0.330654958	0.170014451	0.147010278	-0.002573366	0.663883282	1.391879453	0.997429942	1.942320287
genus.Romboutsia.id.11347	4-androsten-3beta,17beta-diol disulfate 1*	Weighted median	5	0.023150303	0.063056007	0.71351553	-0.100439471	0.146740077	1.023420351	0.904439855	1.15805292
genus.Romboutsia.id.11347	4-androsten-3beta,17beta-diol disulfate 1*	Inverse variance weighted	5	-0.004900689	0.057148133	0.931661869	-0.116911029	0.107109652	0.9951113	0.889664344	1.113056296
genus.Romboutsia.id.11347	4-androsten-3beta,17beta-diol disulfate 1*	Simple mode	5	0.022847562	0.061640201	0.729685127	-0.097967232	0.143662356	1.023110567	0.906678613	1.154494235
genus.Romboutsia.id.11347	4-androsten-3beta,17beta-diol disulfate 1*	Weighted mode	5	0.022847562	0.05661145	0.707161385	-0.088110881	0.133806005	1.023110567	0.915659342	1.143171028
genus.Romboutsia.id.11347	propionylcarnitine	MR Egger	8	0.589027485	0.591717137	0.357950884	-0.570738104	1.748793074	1.802234863	0.565108176	5.747661488
genus.Romboutsia.id.11347	propionylcarnitine	Weighted median	8	0.174488161	0.279249815	0.532072062	-0.372841476	0.721817798	1.190636647	0.688774412	2.058171152
genus.Romboutsia.id.11347	propionylcarnitine	Inverse variance weighted	8	-0.072290875	0.217366936	0.739454887	-0.498330069	0.353748319	0.930260267	0.60754437	1.424396647
genus.Romboutsia.id.11347	propionylcarnitine	Simple mode	8	-0.610279832	0.420291112	0.189791943	-1.434050412	0.213490748	0.543198843	0.238341583	1.237992044
genus.Romboutsia.id.11347	propionylcarnitine	Weighted mode	8	0.192625927	0.339876319	0.588585013	-0.473531657	0.858783512	1.212429172	0.622798868	2.360287684
genus.Romboutsia.id.11347	X-12092	MR Egger	4	0.049583433	0.063097657	0.514287965	-0.074087974	0.17325484	1.050833263	0.928589998	1.189169114
genus.Romboutsia.id.11347	X-12092	Weighted median	4	0.04296172	0.047650445	0.367268511	-0.050433152	0.136356592	1.043897934	0.950817487	1.146090507
genus.Romboutsia.id.11347	X-12092	Inverse variance weighted	4	0.041121215	0.046925759	0.380864968	-0.050853272	0.133095702	1.041978401	0.950418113	1.142359319
genus.Romboutsia.id.11347	X-12092	Simple mode	4	0.074589256	0.13394508	0.616465412	-0.187943101	0.337121613	1.077441507	0.828661856	1.400909422
genus.Romboutsia.id.11347	X-12092	Weighted mode	4	0.043535467	0.047508356	0.427045524	-0.049580911	0.136651846	1.044497039	0.951628158	1.146428945
genus.Romboutsia.id.11347	acetylcarnitine	MR Egger	7	1.357577061	0.930536559	0.204398788	-0.466274595	3.181428717	3.88676449	0.627334998	24.0811341
genus.Romboutsia.id.11347	acetylcarnitine	Weighted median	7	0.193391153	0.289937491	0.504765862	-0.37488633	0.761668636	1.213357309	0.687367408	2.141847204
genus.Romboutsia.id.11347	acetylcarnitine	Inverse variance weighted	7	-0.12575419	0.241236007	0.60216398	-0.598576763	0.347068383	0.881831583	0.549593281	1.414913478
genus.Romboutsia.id.11347	acetylcarnitine	Simple mode	7	-0.606486369	0.471089181	0.245377712	-1.529821163	0.316848425	0.545263361	0.216574395	1.372794475
genus.Romboutsia.id.11347	acetylcarnitine	Weighted mode	7	0.218582033	0.422583281	0.623488123	-0.609681197	1.046845264	1.244311087	0.543524118	2.848650189
genus.Romboutsia.id.11347	X-11423	Inverse variance weighted	2	0.69363154	0.60252432	0.249646181	-0.487316127	1.874579206	2.000968953	0.614272814	6.518075778
genus.Romboutsia.id.11347	X-11440	MR Egger	5	0.002261365	0.109992809	0.984888241	-0.213324541	0.217847271	1.002263924	0.8078939	1.24339715
genus.Romboutsia.id.11347	X-11440	Weighted median	5	0.02550131	0.063989048	0.690242378	-0.099917225	0.150919845	1.02582925	0.904912319	1.162903441

genus.Romboutsia.id.11347	X-11440	Inverse variance weighted	5	0.025875252	0.058369539	0.657548316	-0.088529045	0.140279549	1.026212923	0.915276526	1.150595402
genus.Romboutsia.id.11347	X-11440	Simple mode	5	0.018116644	0.080313333	0.832588908	-0.139297489	0.175530777	1.018281746	0.869969184	1.191878671
genus.Romboutsia.id.11347	X-11440	Weighted mode	5	0.02443549	0.069542038	0.743033018	-0.111866905	0.160737885	1.024736483	0.894163258	1.174377106
genus.Romboutsia.id.11347	X-12442	Wald ratio	1	0.269686508	0.280880353	0.336981093	-0.280838983	0.820212	1.309553852	0.755149917	2.270981234
genus.Romboutsia.id.11347	3-dehydrocarnitine*	MR Egger	6	0.358507523	2.832274545	0.905381037	-5.192750584	5.909765631	1.431191799	0.005556702	368.6197522
genus.Romboutsia.id.11347	3-dehydrocarnitine*	Weighted median	6	-0.390903666	0.268031182	0.144723136	-0.916244782	0.13443745	0.676445318	0.400018381	1.143893107
genus.Romboutsia.id.11347	3-dehydrocarnitine*	Inverse variance weighted	6	-0.490075548	0.230797887	0.033720596	-0.942439406	-0.037711689	0.612580113	0.389676097	0.962990542
genus.Romboutsia.id.11347	3-dehydrocarnitine*	Simple mode	6	-0.391877083	0.376834284	0.346027158	-1.130472279	0.346718113	0.675787175	0.322880731	1.414417963
genus.Romboutsia.id.11347	3-dehydrocarnitine*	Weighted mode	6	-0.393612479	0.313316266	0.264515538	-1.00771236	0.220487403	0.674615434	0.365053135	1.24668422
genus.Romboutsia.id.11347	palmitoylcarnitine	MR Egger	3	2.035345175	30.13969327	0.957073993	-57.03845363	61.10914398	7.65489395	1.69E-25	3.46E+26
genus.Romboutsia.id.11347	palmitoylcarnitine	Weighted median	3	-0.502599931	0.42625953	0.238360863	-1.33806861	0.332868748	0.60495577	0.262351883	1.394964195
genus.Romboutsia.id.11347	palmitoylcarnitine	Inverse variance weighted	3	-0.524077485	0.350284449	0.134615011	-1.210635004	0.162480034	0.592101335	0.298007983	1.17642483
genus.Romboutsia.id.11347	palmitoylcarnitine	Simple mode	3	-0.493315152	0.457165718	0.393396068	-1.38935996	0.402729655	0.610598807	0.249234774	1.495902427
genus.Romboutsia.id.11347	palmitoylcarnitine	Weighted mode	3	-0.493315152	0.463232315	0.398453496	-1.401250491	0.414620186	0.610598807	0.246288789	1.51379567
genus.Romboutsia.id.11347	betaine	MR Egger	4	-1.94699513	1.984942323	0.430078704	-5.837482083	1.943491824	0.14270223	0.002916176	6.983092174
genus.Romboutsia.id.11347	betaine	Weighted median	4	-0.439893114	0.446156514	0.324152041	-1.314359881	0.434573654	0.644105263	0.268646234	1.544304511
genus.Romboutsia.id.11347	betaine	Inverse variance weighted	4	-0.550978889	0.521877719	0.291076767	-1.573859217	0.47190144	0.576385317	0.207243838	1.60303938
genus.Romboutsia.id.11347	betaine	Simple mode	4	0.303615096	0.889462292	0.755352361	-1.439730997	2.04696119	1.354747509	0.236991502	7.744331757
genus.Romboutsia.id.11347	betaine	Weighted mode	4	-1.236567181	0.757823088	0.20123544	-2.721900433	0.248766071	0.290379329	0.065749683	1.282441998
genus.Romboutsia.id.11347	stearoylcarnitine	MR Egger	3	-5.509903914	57.23848969	0.938905813	-117.6973437	106.6775359	0.004046496	7.67E-52	2.14E+46
genus.Romboutsia.id.11347	stearoylcarnitine	Weighted median	3	-0.477578847	0.398435411	0.23066969	-1.258512253	0.303354559	0.620283376	0.284076346	1.354394592
genus.Romboutsia.id.11347	stearoylcarnitine	Inverse variance weighted	3	-0.502529607	0.335562708	0.13424426	-1.160232515	0.1551733	0.604998314	0.313413299	1.167860334
genus.Romboutsia.id.11347	stearoylcarnitine	Simple mode	3	-0.476019704	0.442685233	0.394738954	-1.34368276	0.391643353	0.621251241	0.260883127	1.479409989
genus.Romboutsia.id.11347	stearoylcarnitine	Weighted mode	3	-0.476019704	0.4472947	0.398713749	-1.352717316	0.400677908	0.621251241	0.258536779	1.49283636
family.Lachnospiraceae.id.1987	succinylcarnitine	MR Egger	6	-0.5145854	0.466143796	0.331568379	-1.428227239	0.39905644	0.59774837	0.239733536	1.490417735
family.Lachnospiraceae.id.1987	succinylcarnitine	Weighted median	6	-0.526757844	0.246928824	0.032905243	-1.010738338	-0.042777349	0.590516416	0.363950162	0.958124694
family.Lachnospiraceae.id.1987	succinylcarnitine	Inverse variance weighted	6	-0.591196754	0.228556377	0.009691225	-1.039167253	-0.143226255	0.553664288	0.353749143	0.866557983
family.Lachnospiraceae.id.1987	succinylcarnitine	Simple mode	6	-0.695902124	0.475762609	0.203408098	-1.628396838	0.23659259	0.498624424	0.196243933	1.266924855
family.Lachnospiraceae.id.1987	succinylcarnitine	Weighted mode	6	-0.539200301	0.276843964	0.10899505	-1.08181447	0.003413867	0.583214462	0.338979899	1.003419701
family.Lachnospiraceae.id.1987	lysine	Wald ratio	1	1.354294636	0.828020712	0.101927387	-0.268625959	2.977215232	3.874027394	0.76442913	19.6330669
family.Lachnospiraceae.id.1987	oleoylcarnitine	MR Egger	3	-3.439394954	13.71758023	0.843605028	-30.3258522	23.4470623	0.032084092	6.76E-14	15238063245
family.Lachnospiraceae.id.1987	oleoylcarnitine	Weighted median	3	0.668819378	0.347374418	0.054184851	-0.012034481	1.349673237	1.951931467	0.988037644	3.856165273
family.Lachnospiraceae.id.1987	oleoylcarnitine	Inverse variance weighted	3	0.612419957	0.281193553	0.029411227	0.061280593	1.16355932	1.844890556	1.063197198	3.2013075
family.Lachnospiraceae.id.1987	oleoylcarnitine	Simple mode	3	0.700050373	0.400063278	0.222248717	-0.084073652	1.484174399	2.01385415	0.919363541	4.411321917
family.Lachnospiraceae.id.1987	oleoylcarnitine	Weighted mode	3	0.701069731	0.380629786	0.206833612	-0.044964649	1.447104111	2.015908035	0.956031278	4.250786869
family.Lachnospiraceae.id.1987	epiandrosterone sulfate	MR Egger	3	-0.296561242	0.119708562	0.2442415	-0.531190024	-0.06193246	0.7433701	0.587904932	0.939946368
family.Lachnospiraceae.id.1987	epiandrosterone sulfate	Weighted median	3	-0.161609915	0.072393191	0.025589126	-0.303500571	-0.01971926	0.850773013	0.738229468	0.980473893
family.Lachnospiraceae.id.1987	epiandrosterone sulfate	Inverse variance weighted	3	-0.128280252	0.083274297	0.123449646	-0.291497875	0.03493737	0.879606833	0.747143601	1.03555485
family.Lachnospiraceae.id.1987	epiandrosterone sulfate	Simple mode	3	-0.164077707	0.08481193	0.192700865	-0.330309089	0.002153676	0.848676072	0.718701556	1.002155997
family.Lachnospiraceae.id.1987	epiandrosterone sulfate	Weighted mode	3	-0.164077707	0.07358099	0.1555146	-0.308296447	-0.019858966	0.848676072	0.734697487	0.980336925
family.Lachnospiraceae.id.1987	X-12798	MR Egger	6	-0.13973761	0.093758979	0.210374978	-0.32350521	0.044029989	0.869586376	0.723608188	1.045013693
family.Lachnospiraceae.id.1987	X-12798	Weighted median	6	-0.114505148	0.069859329	0.101196244	-0.251429434	0.022419137	0.891807347	0.777688334	1.022672334
family.Lachnospiraceae.id.1987	X-12798	Inverse variance weighted	6	-0.074942972	0.062638875	0.231529157	-0.197715168	0.047829223	0.927796395	0.820603554	1.048991497
family.Lachnospiraceae.id.1987	X-12798	Simple mode	6	0.182522845	0.129503089	0.217770383	-0.07130321	0.436348901	1.200241571	0.931179506	1.547048467
family.Lachnospiraceae.id.1987	X-12798	Weighted mode	6	-0.110055179	0.070703195	0.180298928	-0.248633442	0.028523083	0.895784705	0.779865787	1.028933762
family.Lachnospiraceae.id.1987	X-12850	MR Egger	4	0.544606868	1.759062347	0.786143962	-2.903155332	3.992369069	1.723930518	0.054849877	54.18310092
family.Lachnospiraceae.id.1987	X-12850	Weighted median	4	-0.074844895	0.15394423	0.626838219	-0.376575586	0.226885796	0.927887395	0.686207248	1.25468657
family.Lachnospiraceae.id.1987	X-12850	Inverse variance weighted	4	-0.073697892	0.134720898	0.584350375	-0.337750851	0.190355068	0.928952296	0.713373002	1.20967904
family.Lachnospiraceae.id.1987	X-12850	Simple mode	4	-0.077635132	0.197270659	0.720218026	-0.464285624	0.309015361	0.925301978	0.62858399	1.362083293
family.Lachnospiraceae.id.1987	X-12850	Weighted mode	4	-0.069270654	0.205703636	0.758486827	-0.472449781	0.333908472	0.933074105	0.623473024	1.396415326
family.Lachnospiraceae.id.1987	4-androsten-3beta,17beta-diol disulfate 1*	MR Egger	5	0.258952735	0.149765465	0.182241613	-0.034587575	0.552493046	1.295572569	0.966003738	1.737579488
family.Lachnospiraceae.id.1987	4-androsten-3beta,17beta-diol disulfate 1*	Weighted median	5	-0.023127431	0.055426299	0.676484897	-0.131762976	0.085508114	0.977137958	0.876548733	1.0892704
family.Lachnospiraceae.id.1987	4-androsten-3beta,17beta-diol disulfate 1*	Inverse variance weighted	5	-0.043787099	0.05231789	0.402624533	-0.146330163	0.058755965	0.957157716	0.863872438	1.060516406
family.Lachnospiraceae.id.1987	4-androsten-3beta,17beta-diol disulfate 1*	Simple mode	5	-0.018529456	0.06022189	0.773676295	-0.136564361	0.099505449	0.981641159	0.872350173	1.10462449
family.Lachnospiraceae.id.1987	4-androsten-3beta,17beta-diol disulfate 1*	Weighted mode	5	-0.019888923	0.051941491	0.721264783	-0.121694245	0.0819164	0.980307557	0.885419048	1.085365069
family.Lachnospiraceae.id.1987	propionylcarnitine	MR Egger	8	-0.850325946	0.521966796	0.154418878	-1.873380867	0.172728975	0.42727564	0.15360347	1.188543936
family.Lachnospiraceae.id.1987	propionylcarnitine	Weighted median	8	-0.270075458	0.255086209	0.289708411	-0.770044427	0.229893512	0.763321894	0.462992498	1.258465991
family.Lachnospiraceae.id.1987	propionylcarnitine	Inverse variance weighted	8	0.07425928	0.206351409	0.718945806	-0.33018948	0.478708041	1.077086037	0.718787524	1.613987849
family.Lachnospiraceae.id.1987	propionylcarnitine	Simple mode	8	-0.32927696	0.444129588	0.482578221	-1.199770951	0.541217032	0.719443732	0.301263208	1.718096569
family.Lachnospiraceae.id.1987	propionylcarnitine	Weighted mode	8	-0.29207776	0.305187458	0.370422735	-0.890245178	0.306089657	0.746710469	0.410555081	1.358104065
family.Lachnospiraceae.id.1987	X-12092	MR Egger	4	0.041896435	0.05554631	0.529404905	-0.066974332	0.150767202	1.042786477	0.935219206	1.162725947
family.Lachnospiraceae.id.1987	X-12092	Weighted median	4	0.062602692	0.042425072	0.140049689	-0.02055045	0.145755834	1.06460378	0.979659271	1.156913675
family.Lachnospiraceae.id.1987	X-12092	Inverse variance weighted	4	0.065805086	0.041318001	0.111238882	-0.015178196	0.146788368	1.068018525	0.984936413	1.158108844
family.Lachnospiraceae.id.1987	X-12092	Simple mode	4	0.070036157	0.128478447	0.623585007	-0.1817816	0.321853914	1.07254696	0.833783419	1.379683208
family.Lachnospiraceae.id.1987	X-12092	Weighted mode	4	0.061123588	0.041820865	0.240016892	-0.020845309	0.143092484	1.063030284	0.979370453	1.153836508
family.Lachnospiraceae.id.1987	acetylcarnitine	MR Egger	7	-1.97429435	0.82054633	0.061160031	-3.582565158	-0.366023543	0.138859264	0.027804284	0.693486474
family.Lachnospiraceae.id.1987	acetylcarnitine	Weighted median	7	-0.193766974	0.288053894	0.501153366	-0.758352605	0.370818657	0.82384986	0.468437493	1.448920298
family.Lachnospiraceae.id.1987	acetylcarnitine	Inverse variance weighted	7	0.14807162	0.237772058	0.533451386	-0.317961612	0.614104853	1.159595944	0.72763072	1.848001626
family.Lachnospiraceae.id.1987	acetylcarnitine	Simple mode	7	0.84925914	0.553669057	0.175955699	-0.235932213	1.934450492	2.337914143	0.789834213	6.920240285
family.Lachnospiraceae.id.1987	acetylcarnitine	Weighted mode	7	-0.331527761	0.384056276	0.421173261	-1.084278062	0.421222539	0.717826228	0.338145818	1.523823351
family.Lachnospiraceae.id.1987	X-11423	Inverse variance weighted	2	0.152972963	0.532397721	0.773860911	-0.890526569	1.196472496	1.165293473	0.410439571	3.30842583
family.Lachnospiraceae.id.1987	X-11440	MR Egger	5	0.066618703	0.097047678	0.541725172	-0.123594746	0.256832152	1.068887836	0.883737906	1.29282811
family.Lachnospiraceae.id.1987	X-11440	Weighted median	5	-0.0237719	0.060548557	0.69460839	-0.142447073	0.094903272	0.976508425	0.867233453	1.099552493
family.Lachnospiraceae.id.1987	X-11440	Inverse variance weighted	5	-0.027811578	0.051667887	0.590386597	-0.129080637	0.073457481	0.972571604	0.87890309	1.076222776
family.Lachnospiraceae.id.1987	X-11440	Simple mode	5	-0.021756609	0.062237049	0.744285223	-0.143741225	0.100228006	0.978478358	0.866111847	1.105422933
family.Lachnospiraceae.id.1987	X-11440	Weighted mode	5	-0.021756609	0.059370372	0.73258586	-0.138122538	0.094609319	0.978478358	0.870991956	1.099229323
family.Lachnospiraceae.id.1987	X-12442	Wald ratio	1	0.210322135	0.24718901	0.394849613	-0.274168325	0.694812595	1.234075535	0.760204103	2.003333604
family.Lachnospiraceae.id.1987	3-dehydrocarnitine*	MR Egger	6	2.421413267	2.994285971	0.464052996	-3.447387236	8.29021377	11.26176396	0.031828689	3984.685912
family.Lachnospiraceae.id.1987	3-dehydrocarnitine*	Weighted median	6	0.568290742	0.248205358	0.022044572	0.081808241	1.054773244	1.765247209	1.085247683	2.87132399
family.Lachnospiraceae.id.1987	3-dehydrocarnitine*	Inverse variance weighted	6	0.344691761	0.231000755	0.135655305	-0.108069718	0.79745324	1.411554756	0.897565018	2.219880222
family.Lachnospiraceae.id.1987	3-dehydrocarnitine*	Simple mode	6	0.579255728	0.2736989	0.087905988	0.042805883	1.115705572	1.784709626	1.043735269	3.051720629
family.Lachnospiraceae.id.1987	3-dehydrocarnitine*	Weighted mode	6	0.574618336	0.276829722	0.092565183	0.032032081	1.117204591	1.776452389	1.03255063	3.056298646
family.Lachnospiraceae.id.1987	palmitoylcarnitine	MR Egger	3	-10.34432759	26.51205063	0.763172621	-62.30794682	41.61929163	3.22E-05	8.71E-28	1.18858E+18
family.Lachnospiraceae.id.1987	palmitoylcarnitine	Weighted median	3	0.71761231	0.367402351	0.050795244	-0.002496297	1.437720917	2.049533712	0.997506816	4.211087453
family.Lachnospiraceae.id.1987	palmitoylcarnitine	Inverse variance weighted	3	0.67084051	0.307887373	0.029342787	0.06738126	1.274299761	1.955880568	1.069703235	3.576196341
family.Lachnospiraceae.id.1987	palmitoylcarnitine	Simple mode	3	0.764300565	0.430393092	0.217748803	-0.079269896	1.607871026	2.147491818	0.923790563	4.992171701
family.Lachnospiraceae.id.1987	palmitoylcarnitine	Weighted mode	3	0.764300565	0.44705684	0.229461718	-0.111930842	1.640531972	2.147491818	0.89410609	5.157912646
family.Lachnospiraceae.id.1987	betaine	MR Egger	4	0.468948772	1.045435501	0.697658954	-1.58010481	2.518002353	1.598313118	0.205953511	12.4037935
family.Lachnospiraceae.id.1987	betaine	Weighted median	4	0.056338342	0.370999659	0.879300899	-0.67082099	0.783497674	1.057955574	0.511288643	2.189115704
family.Lachnospiraceae.id.1987	betaine	Inverse variance weighted	4	-0.074793418	0.298902217	0.802411772	-0.660641763	0.511054926	0.927935161	0.516519745	1.667048882
family.Lachnospiraceae.id.1987	betaine	Simple mode	4	0.100501809	0.565413833	0.870244494	-1.007709304	1.208712921	1.105725642	0.365054251	3.349171229
family.Lachnospiraceae.id.1987	betaine	Weighted mode	4	0.104795621	0.488590037	0.843920334	-0.852840851	1.062432093	1.110483628	0.426202433	2.893399455
family.Lachnospiraceae.id.1987	stearoylcarnitine	MR Egger	3	19.99225057	50.30277908	0.759169249	-78.60119642	118.5856976	481419971.2	7.31E-35	3.17E+51
family.Lachnospiraceae.id.1987	stearoylcarnitine	Weighted median	3	0.689850277	0.348579785	0.047812491	0.006633898	1.373066655	1.99341705	1.006655952	3.947437581
family.Lachnospiraceae.id.1987	stearoylcarnitine	Inverse variance weighted	3	0.64467252	0.294933751	0.028828671	0.066602368	1.222742672	1.905362959	1.068870377	3.396490431
family.Lachnospiraceae.id.1987	stearoylcarnitine	Simple mode	3	0.724390869	0.407623942	0.217529103	-0.074552057	1.523333794	2.06347379	0.928159156	4.587493489
family.Lachnospiraceae.id.1987	stearoylcarnitine	Weighted mode	3	0.723114288	0.400707828	0.212902438	-0.062273055	1.50850163	2.06084128	0.939626282	4.519953157
genus..Eubacteriumhalliigroup.id.11	succinylcarnitine	MR Egger	6	0.385199621	0.589027587	0.548828587	-0.76929445	1.539693692	1.469907716	0.463339862	4.663161689
genus..Eubacteriumhalliigroup.id.11	succinylcarnitine	Weighted median	6	0.336496222	0.272321806	0.216586257	-0.197254518	0.870246961	1.400033579	0.820981652	2.3875004
genus..Eubacteriumhalliigroup.id.11	succinylcarnitine	Inverse variance weighted	6	0.374336538	0.258399811	0.147429239	-0.132127091	0.880800167	1.454026403	0.876229627	2.4128296
genus..Eubacteriumhalliigroup.id.11	succinylcarnitine	Simple mode	6	-0.203568091	0.454448532	0.672934663	-1.094287214	0.687151031	0.815814653	0.334778147	1.988043584
genus..Eubacteriumhalliigroup.id.11	succinylcarnitine	Weighted mode	6	0.414349193	0.292192377	0.215372765	-0.158347867	0.987046253	1.513385498	0.853552808	2.683296975
genus..Eubacteriumhalliigroup.id.11	lysine	Wald ratio	1	0.436194827	0.871715559	0.616802852	-1.272367667	2.144757322	1.546810126	0.280167492	8.539968526
genus..Eubacteriumhalliigroup.id.11	oleoylcarnitine	MR Egger	3	3.468807291	14.44459157	0.849959676	-24.84259218	31.78020676	32.09843552	1.63E-11	6.33824E+13
genus..Eubacteriumhalliigroup.id.11	oleoylcarnitine	Weighted median	3	0.076907066	0.341111971	0.821621528	-0.591672397	0.74548653	1.079941709	0.553401004	2.107466531
genus..Eubacteriumhalliigroup.id.11	oleoylcarnitine	Inverse variance weighted	3	0.132721011	0.296296403	0.65420137	-0.448019939	0.713461961	1.141931368	0.638891945	2.04104506
genus..Eubacteriumhalliigroup.id.11	oleoylcarnitine	Simple mode	3	0.065185676	0.409037691	0.88802172	-0.736528199	0.866899551	1.067357188	0.478773239	2.379521818
genus..Eubacteriumhalliigroup.id.11	oleoylcarnitine	Weighted mode	3	0.065185676	0.40645501	0.887319198	-0.731466143	0.861837494	1.067357188	0.48120296	2.36750698
genus..Eubacteriumhalliigroup.id.11	epiandrosterone sulfate	MR Egger	3	-0.094660891	0.125683683	0.589045791	-0.34100091	0.151679127	0.909681363	0.711058261	1.163786748
genus..Eubacteriumhalliigroup.id.11	epiandrosterone sulfate	Weighted median	3	-0.085556205	0.075727936	0.258567283	-0.233982959	0.062870549	0.918001546	0.791375302	1.06488898
genus..Eubacteriumhalliigroup.id.11	epiandrosterone sulfate	Inverse variance weighted	3	-0.084059695	0.071653474	0.24073889	-0.224500504	0.056381113	0.919376372	0.798915174	1.058000825
genus..Eubacteriumhalliigroup.id.11	epiandrosterone sulfate	Simple mode	3	-0.086320054	0.093780947	0.454509898	-0.270130711	0.097490603	0.917300599	0.763279719	1.102401083
genus..Eubacteriumhalliigroup.id.11	epiandrosterone sulfate	Weighted mode	3	-0.086153019	0.090800149	0.44285853	-0.264121311	0.091815273	0.917453832	0.767880381	1.096162312
genus..Eubacteriumhalliigroup.id.11	X-12798	MR Egger	6	-0.083355298	0.098383369	0.444573082	-0.276186702	0.109476105	0.920024206	0.758671272	1.115693411
genus..Eubacteriumhalliigroup.id.11	X-12798	Weighted median	6	-0.002462609	0.074499889	0.973630564	-0.148482392	0.143557174	0.997540421	0.862015185	1.154372809
genus..Eubacteriumhalliigroup.id.11	X-12798	Inverse variance weighted	6	0.022502678	0.065701903	0.731977211	-0.106273052	0.151278407	1.022757773	0.899179092	1.16332049
genus..Eubacteriumhalliigroup.id.11	X-12798	Simple mode	6	-0.003319217	0.113521669	0.977805443	-0.225821688	0.219183254	0.996686286	0.797860357	1.245059417
genus..Eubacteriumhalliigroup.id.11	X-12798	Weighted mode	6	-0.003319217	0.073211076	0.965593205	-0.146812926	0.140174492	0.996686286	0.863455492	1.15047453
genus..Eubacteriumhalliigroup.id.11	X-12850	MR Egger	4	2.271130439	1.849160737	0.344294347	-1.353224606	5.895485483	9.690348971	0.258405659	363.3932144
genus..Eubacteriumhalliigroup.id.11	X-12850	Weighted median	4	0.106642924	0.167126779	0.523411212	-0.220925564	0.434211411	1.112536923	0.801776359	1.543745198
genus..Eubacteriumhalliigroup.id.11	X-12850	Inverse variance weighted	4	0.029773441	0.141744977	0.833629151	-0.248046714	0.307593596	1.030221102	0.78032349	1.360148108
genus..Eubacteriumhalliigroup.id.11	X-12850	Simple mode	4	0.134326618	0.237384378	0.611049431	-0.330946763	0.599599999	1.143766333	0.718243405	1.821390097
genus..Eubacteriumhalliigroup.id.11	X-12850	Weighted mode	4	0.132577807	0.188719694	0.532964036	-0.237312793	0.502468407	1.14176785	0.788744536	1.652796012
genus..Eubacteriumhalliigroup.id.11	4-androsten-3beta,17beta-diol disulfate 1*	MR Egger	5	0.218447117	0.15743543	0.259383236	-0.090126327	0.52702056	1.24414322	0.913815738	1.693877975
genus..Eubacteriumhalliigroup.id.11	4-androsten-3beta,17beta-diol disulfate 1*	Weighted median	5	0.04343958	0.058848545	0.460417866	-0.071903568	0.158782729	1.04439689	0.930620633	1.172083259
genus..Eubacteriumhalliigroup.id.11	4-androsten-3beta,17beta-diol disulfate 1*	Inverse variance weighted	5	0.022991892	0.050773653	0.65067033	-0.076524468	0.122508251	1.023258243	0.926330249	1.130328446
genus..Eubacteriumhalliigroup.id.11	4-androsten-3beta,17beta-diol disulfate 1*	Simple mode	5	0.038979899	0.066547005	0.589499427	-0.091452231	0.169412029	1.039749583	0.912604909	1.184608131
genus..Eubacteriumhalliigroup.id.11	4-androsten-3beta,17beta-diol disulfate 1*	Weighted mode	5	0.040037615	0.059777378	0.539678283	-0.077126045	0.157201276	1.040849925	0.925773157	1.170231129
genus..Eubacteriumhalliigroup.id.11	propionylcarnitine	MR Egger	8	-0.186569229	0.548138112	0.745179133	-1.260919928	0.88778147	0.829801114	0.283393205	2.429733233

genus..Eubacteriumhalliigroup.id.11	propionylcarnitine	Weighted median	8	0.015658351	0.241719108	0.948349868	-0.458111102	0.489427803	1.015781585	0.632477203	1.63138248
genus..Eubacteriumhalliigroup.id.11	propionylcarnitine	Inverse variance weighted	8	-0.045333765	0.201367992	0.82187896	-0.440015028	0.349347499	0.955678457	0.644026742	1.418141908
genus..Eubacteriumhalliigroup.id.11	propionylcarnitine	Simple mode	8	0.077931011	0.367427621	0.838073456	-0.642227125	0.798089148	1.081048076	0.526119385	2.221292309
genus..Eubacteriumhalliigroup.id.11	propionylcarnitine	Weighted mode	8	-0.092475007	0.306566626	0.771685154	-0.693345595	0.508395581	0.911671996	0.499900803	1.662621513
genus..Eubacteriumhalliigroup.id.11	X-12092	MR Egger	4	0.010696602	0.058442678	0.871650624	-0.103851047	0.125244251	1.010754016	0.901359548	1.13342526
genus..Eubacteriumhalliigroup.id.11	X-12092	Weighted median	4	0.001418714	0.045399361	0.975070447	-0.087564033	0.090401461	1.001419721	0.916160205	1.09461364
genus..Eubacteriumhalliigroup.id.11	X-12092	Inverse variance weighted	4	0.000325423	0.043476949	0.994027926	-0.084889397	0.085540243	1.000325476	0.918613881	1.089305397
genus..Eubacteriumhalliigroup.id.11	X-12092	Simple mode	4	0.003197555	0.134841823	0.982570353	-0.261092419	0.267487528	1.003202672	0.770209734	1.306677333
genus..Eubacteriumhalliigroup.id.11	X-12092	Weighted mode	4	0.003197555	0.044665691	0.947434684	-0.0843472	0.090742309	1.003202672	0.919112085	1.0949868
genus..Eubacteriumhalliigroup.id.11	acetylcarnitine	MR Egger	7	-0.944735243	0.862056991	0.323075874	-2.634366945	0.744896459	0.38878249	0.071764386	2.106223343
genus..Eubacteriumhalliigroup.id.11	acetylcarnitine	Weighted median	7	0.082920156	0.274198798	0.762340375	-0.454509489	0.6203498	1.086455058	0.634759248	1.859578409
genus..Eubacteriumhalliigroup.id.11	acetylcarnitine	Inverse variance weighted	7	0.008468525	0.223479663	0.969772245	-0.429551615	0.446488665	1.008504484	0.650800839	1.562814973
genus..Eubacteriumhalliigroup.id.11	acetylcarnitine	Simple mode	7	0.131097695	0.406581318	0.758059469	-0.665801689	0.927997079	1.140079156	0.513861405	2.529437836
genus..Eubacteriumhalliigroup.id.11	acetylcarnitine	Weighted mode	7	-0.213441585	0.361854938	0.576811016	-0.922677264	0.495794094	0.807799346	0.397453527	1.641801465
genus..Eubacteriumhalliigroup.id.11	X-11423	Inverse variance weighted	2	0.345434785	1.168156215	0.767452011	-1.944151397	2.635020966	1.412603965	0.143108614	13.94360481
genus..Eubacteriumhalliigroup.id.11	X-11440	MR Egger	5	0.064711434	0.102112927	0.571277639	-0.135429902	0.264852771	1.066851123	0.87334038	1.303239087
genus..Eubacteriumhalliigroup.id.11	X-11440	Weighted median	5	0.047979411	0.060803263	0.43005783	-0.071194985	0.167153807	1.049149054	0.931280289	1.181936042
genus..Eubacteriumhalliigroup.id.11	X-11440	Inverse variance weighted	5	0.043619365	0.054376142	0.422449788	-0.062957874	0.150196604	1.044584674	0.938983029	1.162062686
genus..Eubacteriumhalliigroup.id.11	X-11440	Simple mode	5	0.042755502	0.070975628	0.579393069	-0.096356729	0.181867732	1.043682685	0.908139998	1.199455534
genus..Eubacteriumhalliigroup.id.11	X-11440	Weighted mode	5	0.044247527	0.064477448	0.530267564	-0.082128271	0.170623325	1.045241048	0.921153794	1.186043912
genus..Eubacteriumhalliigroup.id.11	X-12442	Wald ratio	1	-0.296586315	0.260107461	0.254184112	-0.806396938	0.213224309	0.743351462	0.446463809	1.237662238
genus..Eubacteriumhalliigroup.id.11	3-dehydrocarnitine*	MR Egger	6	-1.045322824	2.623511616	0.710655568	-6.187405592	4.096759945	0.351578303	0.002055152	60.1450981
genus..Eubacteriumhalliigroup.id.11	3-dehydrocarnitine*	Weighted median	6	0.088338513	0.245375848	0.718837356	-0.392598148	0.569275175	1.092357837	0.675300063	1.766985832
genus..Eubacteriumhalliigroup.id.11	3-dehydrocarnitine*	Inverse variance weighted	6	0.149651774	0.213736256	0.483820969	-0.269271287	0.568574836	1.161429732	0.763935981	1.765748775
genus..Eubacteriumhalliigroup.id.11	3-dehydrocarnitine*	Simple mode	6	0.087847046	0.340968361	0.806952403	-0.580450942	0.756145033	1.091821111	0.559645942	2.130049104
genus..Eubacteriumhalliigroup.id.11	3-dehydrocarnitine*	Weighted mode	6	0.073865645	0.328411521	0.83094625	-0.569820936	0.717552227	1.076662141	0.565626713	2.049410574
genus..Eubacteriumhalliigroup.id.11	palmitoylcarnitine	MR Egger	3	8.769380967	27.92245557	0.806272003	-45.95863194	63.49739388	6434.188259	1.10E-20	3.77E+27
genus..Eubacteriumhalliigroup.id.11	palmitoylcarnitine	Weighted median	3	0.083965129	0.385078694	0.827392744	-0.670789111	0.838719369	1.087590968	0.511304942	2.313402464
genus..Eubacteriumhalliigroup.id.11	palmitoylcarnitine	Inverse variance weighted	3	0.144924316	0.324419046	0.655077596	-0.490937013	0.780785645	1.15595208	0.612052624	2.183186802
genus..Eubacteriumhalliigroup.id.11	palmitoylcarnitine	Simple mode	3	0.07110442	0.419056709	0.88087437	-0.75024673	0.89245557	1.073693335	0.47225002	2.441116631
genus..Eubacteriumhalliigroup.id.11	palmitoylcarnitine	Weighted mode	3	0.071617641	0.421001394	0.880573127	-0.753545092	0.896780374	1.074244519	0.470694935	2.451696844
genus..Eubacteriumhalliigroup.id.11	betaine	MR Egger	4	0.838393975	1.094878621	0.523856693	-1.307568122	2.984356072	2.312649818	0.270477024	19.77376525
genus..Eubacteriumhalliigroup.id.11	betaine	Weighted median	4	0.283922112	0.357814594	0.427492703	-0.417394493	0.985238716	1.328329465	0.658760992	2.678451196
genus..Eubacteriumhalliigroup.id.11	betaine	Inverse variance weighted	4	0.287816884	0.313636723	0.358788532	-0.326911093	0.902544862	1.333513094	0.721147855	2.465870433
genus..Eubacteriumhalliigroup.id.11	betaine	Simple mode	4	0.076009129	0.502922499	0.889460069	-0.909718968	1.061737226	1.078972424	0.402637362	2.891389627
genus..Eubacteriumhalliigroup.id.11	betaine	Weighted mode	4	0.42716387	0.452201092	0.414535869	-0.45915027	1.313478011	1.532903839	0.631820294	3.719086267
genus..Eubacteriumhalliigroup.id.11	stearoylcarnitine	MR Egger	3	-15.89879955	53.00449211	0.81448092	-119.7876041	87.99000499	1.25E-07	9.48E-53	1.64E+38
genus..Eubacteriumhalliigroup.id.11	stearoylcarnitine	Weighted median	3	0.072300963	0.384677666	0.850914225	-0.681667262	0.826269188	1.074978824	0.505773033	2.28477874
genus..Eubacteriumhalliigroup.id.11	stearoylcarnitine	Inverse variance weighted	3	0.137157182	0.310778534	0.658971103	-0.471968745	0.746283109	1.147008423	0.623773008	2.109145963
genus..Eubacteriumhalliigroup.id.11	stearoylcarnitine	Simple mode	3	0.06737823	0.400039229	0.88173844	-0.716698659	0.851455119	1.069699994	0.488361847	2.343053795
genus..Eubacteriumhalliigroup.id.11	stearoylcarnitine	Weighted mode	3	0.06737823	0.429052533	0.889634604	-0.773564734	0.908321194	1.069699994	0.461365488	2.480155338
genus..Eubacteriumxylanophilumgr	succinylcarnitine	MR Egger	6	-0.269236593	0.544932166	0.647156316	-1.337303638	0.798830452	0.763962486	0.262552652	2.222939573
genus..Eubacteriumxylanophilumgr	succinylcarnitine	Weighted median	6	-0.181999206	0.309714555	0.55677711	-0.789039733	0.425041321	0.833602002	0.454280817	1.529653625
genus..Eubacteriumxylanophilumgr	succinylcarnitine	Inverse variance weighted	6	-0.256951211	0.267098947	0.336045797	-0.780465147	0.266562725	0.773405947	0.458192835	1.305469472
genus..Eubacteriumxylanophilumgr	succinylcarnitine	Simple mode	6	-0.275875994	0.525056436	0.621748785	-1.304986609	0.753234621	0.758907034	0.271176166	2.123858796
genus..Eubacteriumxylanophilumgr	succinylcarnitine	Weighted mode	6	-0.149636811	0.331499215	0.670609861	-0.799375273	0.50010165	0.861020633	0.44960976	1.648888872
genus..Eubacteriumxylanophilumgr	lysine	Wald ratio	1	-1.385906847	0.963279509	0.150224765	-3.273934684	0.50212099	0.250096897	0.037857178	1.652221903
genus..Eubacteriumxylanophilumgr	oleoylcarnitine	MR Egger	3	-1.480432051	16.07185857	0.941523862	-32.98127484	30.02041074	0.227539359	4.75E-15	1.09068E+13
genus..Eubacteriumxylanophilumgr	oleoylcarnitine	Weighted median	3	0.632322957	0.375029254	0.091783662	-0.102734381	1.367380295	1.881977257	0.902366627	3.925054728
genus..Eubacteriumxylanophilumgr	oleoylcarnitine	Inverse variance weighted	3	0.624560153	0.329755772	0.058223915	-0.02176116	1.270881467	1.867424396	0.978473905	3.563992721
genus..Eubacteriumxylanophilumgr	oleoylcarnitine	Simple mode	3	0.662475113	0.429135275	0.262635255	-0.178630027	1.503580252	1.939587095	0.836415294	4.497763403
genus..Eubacteriumxylanophilumgr	oleoylcarnitine	Weighted mode	3	0.656657746	0.459158537	0.288946787	-0.243292987	1.556608478	1.928336561	0.784041766	4.742708937
genus..Eubacteriumxylanophilumgr	epiandrosterone sulfate	MR Egger	3	-0.253792078	0.139558201	0.32006708	-0.527326153	0.019741997	0.775853102	0.590180915	1.019938159
genus..Eubacteriumxylanophilumgr	epiandrosterone sulfate	Weighted median	3	-0.164530012	0.084369184	0.051161848	-0.329893612	0.000833588	0.848292298	0.719000223	1.000833936
genus..Eubacteriumxylanophilumgr	epiandrosterone sulfate	Inverse variance weighted	3	-0.141678423	0.079283874	0.073941281	-0.297074817	0.013717971	0.867900308	0.742988422	1.013812494
genus..Eubacteriumxylanophilumgr	epiandrosterone sulfate	Simple mode	3	-0.165055153	0.090836358	0.210846574	-0.343094414	0.012984107	0.847846941	0.709571215	1.013068767
genus..Eubacteriumxylanophilumgr	epiandrosterone sulfate	Weighted mode	3	-0.165055153	0.09324404	0.218722349	-0.347813471	0.017703164	0.847846941	0.706230596	1.017860794
genus..Eubacteriumxylanophilumgr	X-12798	MR Egger	6	0.121423577	0.109373497	0.329163876	-0.092948477	0.335795632	1.129103074	0.911240449	1.399053074
genus..Eubacteriumxylanophilumgr	X-12798	Weighted median	6	0.085791027	0.085766652	0.317172993	-0.082311612	0.253893666	1.089578612	0.920984924	1.289034728
genus..Eubacteriumxylanophilumgr	X-12798	Inverse variance weighted	6	0.053381621	0.073013039	0.46470312	-0.089723936	0.196487177	1.054832114	0.914183524	1.217119714
genus..Eubacteriumxylanophilumgr	X-12798	Simple mode	6	0.051451741	0.133758134	0.716304995	-0.210714201	0.313617683	1.052798378	0.810005533	1.368366486
genus..Eubacteriumxylanophilumgr	X-12798	Weighted mode	6	0.08415106	0.081239422	0.347751734	-0.075078207	0.243380327	1.087793203	0.927670933	1.275553659
genus..Eubacteriumxylanophilumgr	X-12850	MR Egger	4	0.329192969	2.080547071	0.8888123	-3.74867929	4.407065227	1.389846026	0.023548827	82.02837524
genus..Eubacteriumxylanophilumgr	X-12850	Weighted median	4	0.397662909	0.178175028	0.025623775	0.048439854	0.746885964	1.488342238	1.049632239	2.110417856
genus..Eubacteriumxylanophilumgr	X-12850	Inverse variance weighted	4	0.382997947	0.156084758	0.014136271	0.077071822	0.688924073	1.46667502	1.08011965	1.991571595
genus..Eubacteriumxylanophilumgr	X-12850	Simple mode	4	0.398307881	0.215929062	0.162289657	-0.024913082	0.821528843	1.489302487	0.975394688	2.273973731
genus..Eubacteriumxylanophilumgr	X-12850	Weighted mode	4	0.39863436	0.203160078	0.144546047	0.000440608	0.796828112	1.489788793	1.000440705	2.218492947
genus..Eubacteriumxylanophilumgr	4-androsten-3beta,17beta-diol disulfate 1*	MR Egger	5	0.494810974	0.173544948	0.065045014	0.154662875	0.834959073	1.640188171	1.167264381	2.304719721
genus..Eubacteriumxylanophilumgr	4-androsten-3beta,17beta-diol disulfate 1*	Weighted median	5	0.126386957	0.062645194	0.0436429	0.003602376	0.249171538	1.134721172	1.003608872	1.282962091
genus..Eubacteriumxylanophilumgr	4-androsten-3beta,17beta-diol disulfate 1*	Inverse variance weighted	5	0.094382888	0.068595949	0.168844826	-0.040065173	0.228830948	1.098980452	0.960726824	1.257129501
genus..Eubacteriumxylanophilumgr	4-androsten-3beta,17beta-diol disulfate 1*	Simple mode	5	0.128563096	0.067976925	0.131556669	-0.004671677	0.26179787	1.137193172	0.995339218	1.299263896
genus..Eubacteriumxylanophilumgr	4-androsten-3beta,17beta-diol disulfate 1*	Weighted mode	5	0.126926875	0.060590532	0.104260161	0.008169432	0.245684318	1.135333994	1.008202893	1.278495911
genus..Eubacteriumxylanophilumgr	propionylcarnitine	MR Egger	8	0.064400699	0.608334747	0.919140965	-1.127935406	1.256736803	1.066519666	0.323700878	3.513936094
genus..Eubacteriumxylanophilumgr	propionylcarnitine	Weighted median	8	0.288013893	0.27627346	0.297181921	-0.25348209	0.829509875	1.333775834	0.776093645	2.292195003
genus..Eubacteriumxylanophilumgr	propionylcarnitine	Inverse variance weighted	8	0.352839693	0.223415821	0.114268199	-0.085055316	0.790734702	1.423102992	0.918461477	2.205015862
genus..Eubacteriumxylanophilumgr	propionylcarnitine	Simple mode	8	0.52098448	0.398730452	0.232634006	-0.260527206	1.302496165	1.683684388	0.77064519	3.67846728
genus..Eubacteriumxylanophilumgr	propionylcarnitine	Weighted mode	8	0.291214068	0.329421722	0.406024506	-0.354452507	0.936880642	1.338050987	0.701557436	2.552008361
genus..Eubacteriumxylanophilumgr	X-12092	MR Egger	4	-0.050090568	0.076852019	0.58143661	-0.200720524	0.100539388	0.951143277	0.81814105	1.105767195
genus..Eubacteriumxylanophilumgr	X-12092	Weighted median	4	-0.019392727	0.048014789	0.686293096	-0.113501712	0.074716259	0.980794103	0.892702668	1.077578355
genus..Eubacteriumxylanophilumgr	X-12092	Inverse variance weighted	4	-0.018400962	0.050912472	0.71778291	-0.118189406	0.081387483	0.981767302	0.888527744	1.084791153
genus..Eubacteriumxylanophilumgr	X-12092	Simple mode	4	0.391952836	0.214136346	0.164605926	-0.027754403	0.811660075	1.479867913	0.972627212	2.251642781
genus..Eubacteriumxylanophilumgr	X-12092	Weighted mode	4	-0.024703587	0.052221086	0.668462093	-0.127056916	0.077649741	0.975599049	0.880683546	1.080744052
genus..Eubacteriumxylanophilumgr	acetylcarnitine	MR Egger	7	-0.432538703	0.95567636	0.669798307	-2.305664369	1.440586962	0.64885974	0.099692546	4.223173934
genus..Eubacteriumxylanophilumgr	acetylcarnitine	Weighted median	7	0.351333165	0.295732809	0.234829963	-0.228303142	0.930969471	1.420960661	0.795882958	2.536967503
genus..Eubacteriumxylanophilumgr	acetylcarnitine	Inverse variance weighted	7	0.444464381	0.247967739	0.073064393	-0.041552388	0.93048115	1.559654591	0.959299078	2.535728951
genus..Eubacteriumxylanophilumgr	acetylcarnitine	Simple mode	7	0.454158311	0.434948044	0.336640045	-0.398339855	1.306656477	1.574847293	0.671433799	3.693802727
genus..Eubacteriumxylanophilumgr	acetylcarnitine	Weighted mode	7	0.355869855	0.397211964	0.404804311	-0.422665595	1.134405304	1.427421764	0.655297731	3.109323891
genus..Eubacteriumxylanophilumgr	X-11423	Inverse variance weighted	2	-0.136531256	0.617619917	0.825045467	-1.347066294	1.074003782	0.872379053	0.260001912	2.927075442
genus..Eubacteriumxylanophilumgr	X-11440	MR Egger	5	0.211919657	0.155001195	0.264989451	-0.091882685	0.515722	1.236048574	0.91221216	1.674847304
genus..Eubacteriumxylanophilumgr	X-11440	Weighted median	5	0.136746645	0.072069333	0.057770971	-0.004509248	0.278002538	1.146537631	0.995500903	1.320489549
genus..Eubacteriumxylanophilumgr	X-11440	Inverse variance weighted	5	0.13776564	0.074461823	0.064291742	-0.008179533	0.283710813	1.147706542	0.991853829	1.32804882
genus..Eubacteriumxylanophilumgr	X-11440	Simple mode	5	0.13791635	0.071592297	0.126335573	-0.002404552	0.278237252	1.147879527	0.997598337	1.320799523
genus..Eubacteriumxylanophilumgr	X-11440	Weighted mode	5	0.134781193	0.066346113	0.112023123	0.004742811	0.264819574	1.144286379	1.004754076	1.303195824
genus..Eubacteriumxylanophilumgr	X-12442	Wald ratio	1	-0.369893973	0.289605864	0.201520236	-0.937521467	0.197733521	0.690807571	0.39159722	1.21863761
genus..Eubacteriumxylanophilumgr	3-dehydrocarnitine*	MR Egger	6	1.016167265	2.909658829	0.744519796	-4.68676404	6.71909857	2.762586186	0.009216462	828.0707273
genus..Eubacteriumxylanophilumgr	3-dehydrocarnitine*	Weighted median	6	0.52536664	0.277672443	0.058486002	-0.018871349	1.069604629	1.691078752	0.9813056	2.914227071
genus..Eubacteriumxylanophilumgr	3-dehydrocarnitine*	Inverse variance weighted	6	0.529796432	0.237601072	0.025762523	0.064098332	0.995494533	1.698586496	1.066197234	2.706062248
genus..Eubacteriumxylanophilumgr	3-dehydrocarnitine*	Simple mode	6	0.648632318	0.384476567	0.152399405	-0.104941753	1.402206389	1.912922769	0.900376965	4.064157192
genus..Eubacteriumxylanophilumgr	3-dehydrocarnitine*	Weighted mode	6	0.528605283	0.349346412	0.190661267	-0.156113685	1.213324251	1.69656443	0.855461931	3.364651026
genus..Eubacteriumxylanophilumgr	palmitoylcarnitine	MR Egger	3	-7.330728873	31.07682522	0.852523388	-68.24130631	53.57984856	0.000655096	2.31E-30	1.86E+23
genus..Eubacteriumxylanophilumgr	palmitoylcarnitine	Weighted median	3	0.679191657	0.431461479	0.115449256	-0.166472843	1.524856157	1.972282807	0.846645809	4.594482637
genus..Eubacteriumxylanophilumgr	palmitoylcarnitine	Inverse variance weighted	3	0.683623792	0.361050696	0.058300866	-0.024035572	1.391283156	1.981043631	0.976250982	4.020005039
genus..Eubacteriumxylanophilumgr	palmitoylcarnitine	Simple mode	3	0.68267641	0.511789557	0.313851187	-0.320431122	1.685783942	1.979167715	0.725836046	5.396679959
genus..Eubacteriumxylanophilumgr	palmitoylcarnitine	Weighted mode	3	0.664719529	0.492890398	0.30987666	-0.301345652	1.630784709	1.943945224	0.739822008	5.107881348
genus..Eubacteriumxylanophilumgr	betaine	MR Egger	4	-2.260686616	1.217523416	0.204468243	-4.647032512	0.125659279	0.104278861	0.009590018	1.133895761
genus..Eubacteriumxylanophilumgr	betaine	Weighted median	4	0.156517087	0.426961551	0.713929671	-0.680327554	0.993361727	1.169430744	0.506451075	2.700296891
genus..Eubacteriumxylanophilumgr	betaine	Inverse variance weighted	4	0.354591066	0.455265751	0.436058587	-0.537729806	1.246911939	1.42559756	0.584072707	3.47958119
genus..Eubacteriumxylanophilumgr	betaine	Simple mode	4	0.048961618	0.554092987	0.935155948	-1.037060637	1.134983873	1.050180042	0.354495142	3.111123371
genus..Eubacteriumxylanophilumgr	betaine	Weighted mode	4	0.048961618	0.479161327	0.92505929	-0.890194583	0.988117819	1.050180042	0.410575854	2.686173847
genus..Eubacteriumxylanophilumgr	stearoylcarnitine	MR Egger	3	16.02385908	59.00107859	0.831175212	-99.61825496	131.6659731	9100674.411	5.45E-44	1.52E+57
genus..Eubacteriumxylanophilumgr	stearoylcarnitine	Weighted median	3	0.649285086	0.404101448	0.108111891	-0.142753753	1.441323925	1.914171871	0.86696753	4.226287401
genus..Eubacteriumxylanophilumgr	stearoylcarnitine	Inverse variance weighted	3	0.656410917	0.345869561	0.057714941	-0.021493422	1.334315255	1.927860651	0.978735916	3.797394811
genus..Eubacteriumxylanophilumgr	stearoylcarnitine	Simple mode	3	0.633965329	0.475235067	0.313823135	-0.297495403	1.565426061	1.885070704	0.742675997	4.784713081
genus..Eubacteriumxylanophilumgr	stearoylcarnitine	Weighted mode	3	0.631137744	0.465724636	0.308125075	-0.281682544	1.543958031	1.879748034	0.754513171	4.683089449
